# The molecular sensory machinery of a Chagas disease vector: expression changes through imaginal moult and sexually dimorphic features

**DOI:** 10.1038/srep40049

**Published:** 2017-01-06

**Authors:** Jose Manuel Latorre-Estivalis, Hugh M. Robertson, Kimberly K. O. Walden, Jerônimo Ruiz, Leilane Oliveira Gonçalves, Alessandra A. Guarneri, Marcelo Gustavo Lorenzo

**Affiliations:** 1 Vector Behaviour and Pathogen Interaction Group, Centro de Pesquisas René Rachou - FIOCRUZ, Belo Horizonte, Minas Gerais, Brazil; 2 Department of Entomology, University of Illinois at Urbana-Champaign, Urbana, IL 61801, USA; 3 Grupo Informática de Biossistemas; Centro de Pesquisas René Rachou - FIOCRUZ, Belo Horizonte, Minas Gerais, Brazil

## Abstract

The triatomine bug *Rhodnius prolixus* is a main vector of Chagas disease, which affects several million people, mostly in Latin-America. Host searching, pheromone communication, and microclimatic preferences are aspects of its behaviour that depend on multimodal sensory inputs. The molecular bases of these sensory processes are largely unknown. The expression levels of genes transcribed in antennae were compared between 5^th^ instar larvae, and female and male adults by means of RNA-Seq. The antennae of *R. prolixus* showed increased expression of several chemosensory-related genes in imaginal bugs, while both sexes had similar expression patterns for most target genes. Few cases suggest involvement of target genes in sexually dimorphic functions. Most odorant and ionotropic receptor genes seemed to be expressed in all libraries. OBPs and CSPs showed very high expression levels. Other sensory-related genes such as TRPs, PPKs and mechanoreceptors had consistent levels of expression in all libraries. Our study characterises most of the sensory gene repertoire of these insects, opening an avenue for functional genetics studies. The increase in expression of chemosensory genes suggests an enhanced role in adult bugs. This knowledge allows developing new behaviour interfering strategies, increasing the options for translational research in the vector control field.

The haematophagous bug *Rhodnius prolixus* Stål, 1859 (Hemiptera, Reduviidae: Triatominae) is the second most important vector of Chagas disease, transmitting its etiological agent, i.e., *Trypanosoma cruzi*, to humans in Colombia and Venezuela^1^,^2^. Chagas disease is an important health problem in Latin America, being endemic to 22 countries in this region. It is considered that 90–100 million people are exposed to its transmission because they live in endemic areas. Eight million people are infected and 12,000–14,000 deaths are reported annually^3^. Vector control strategies are fundamental due to the absence of a vaccine or an effective treatment during the chronic phase of this disease^4^. The emergence of insecticide resistance^5^ requires improved vector control strategies or the development of new sustainable methods to avoid *T. cruzi* transmission.

Numerous neuroethological studies have improved our understanding of diverse aspects of triatomine olfactory-mediated behaviours, such as host searching^6^, mating^7^, and aggregation responses^8^. Aspects of triatomine sensory ecology such as the use of thermal^9^ and humidity^10^ cues, as well as vibratory signals^11^, have also been studied. Stimuli are detected by different sensory neurons mostly located in the eyes, antennae, mouthparts and legs. As in other insects, bug antennae are the main chemosensory structures. Triatomine antennae present four segments: a basal scape, a pedicel, and a distal flagellum, which is composed of two segments. Several morphological, ultrastructural and physiological studies on bug antennae have identified different types of sensilla: mechanosensory (8 types), chemosensory (5), mechano-chemosensory (1), thermo-hygrosensory (1), and one sensillum type with unknown function^12^,^13^. A three to five-fold increase in chemoreceptor sensilla numbers has been described to happen after imaginal molt^13^,^14^,^15^. This increase probably relates to the need of new sensory abilities for several activities performed exclusively by adult bugs (sex, oviposition and flight). Unlike them, host searching and aggregation in hiding places, both partly driven by volatile signals or cues, contact substances, mechanical, thermal and humidity cues, are pervasive to all triatomine stages. It is worth mentioning that no sexual dimorphism has been reported for the number or types of antennal chemosensilla^16^ or regarding the number of glomeruli in the antennal lobe of *R. prolixus* adult bugs^17^. Therefore, no evident dimorphic features are known in triatomines, besides clearly different male and female behavioural profiles shown during sexual interactions.

The *R. prolixus* genome has recently been sequenced^18^, making functional genetic and genomic studies more feasible with these insects. Insect odorant receptors (ORs) are expressed in olfactory sensory neurons (OSNs) housed in trichoid and basiconic sensilla^19^. A total of 116 ORs have been identified in the *R. prolixus* genome, including 5 pseudogenes^18^. Ionotropic receptors, another family of proteins mediating the detection of odours in insects, were characterized in *Drosophila melanogaster* OSNs located in coeloconic sensilla^20^. In the *R. prolixus* genome, 33 IRs have been identified. Other sensory-related genes annotated for this species include gustatory receptors (GRs = 28); odorant binding proteins (OBPs = 27); chemosensory proteins (CSPs = 19); sensory neuron membrane proteins (SNMPs = 1); secreted esterases belonging to the detoxification/pheromone hormone processing class (10); CYP4 clade members (22); transient receptor potential channels (TRPs = 9); and pickpocket receptors (PPKs = 6)^18^,^21^.

In triatomines, as in other hemimetabolous insects, the imaginal moult represents significant changes in physiology and behaviour. Our hypothesis is that the more complex behavioural repertoire of adult bugs should be correlated with changes in gene expression profiles at their sensory structures. To characterize these molecular changes at the peripheral level, we have sequenced the antennal transcriptomes of 5^th^ instar larvae, and female and male adults of *R. prolixus* and characterized transcript levels for the main sensory protein families mentioned above.

## Results

### Transcriptome assemblies

The sequencing yield was 353.6 M read-pairs, distributed as 103 M for larval, 125 M for female, and 125.6 M for male libraries. A total of 128,047 contigs, with an average length of 744 bp, were assembled using SOAPdenovo. The length of contigs ranged from 100 to 35,978 bp and N50 value was 2,518. Using Trinity, a total of 303,403 contigs with an average length of 368 bp were assembled. The length of contigs ranged from 101 to 29,400 bp and N50 value was 649.

### Gene Ontology annotation and functional enrichment analysis

A total of 239; 249 and 177 transcripts had FPKM values >1,000 in larvae, female and male antennal transcriptomes, respectively. After the annotation of these transcripts based on Gene Ontology terms, a functional enrichment analysis was performed for each antennal library comparing it to the *R. prolixus* genome. Those transcripts enriched in the antennal transcriptomes and their Gene Ontology annotations are detailed in Supplementary Table S1. The number of genes that shared a Gene Ontology annotation term in each library and those that were unique are detailed in Supplementary Table S2. The following terms deserve to be highlighted: GO:0005513 (detection of calcium ion); GO:0010880 (regulation of release of sequestered calcium ion into cytosol by sarcoplasmic reticulum); GO:0033173 (calcineurin-NFAT signalling cascade); and GO:0060316 (positive regulation of ryanodine-sensitive calcium-release channel activity) that were common to all libraries (Supplementary Table S2). Triggering Ca^2+^ flux activates a number of signalling pathways including but not restricted to nucleoside diphosphate kinase activity and nucleoside diphosphate phosphorylation, which appeared only in the male library (GO:0004550 and GO:0006165, respectively in Supplementary Table S2). This finding suggests the existence of signalling pathways that are unique for each library and that should be experimentally explored in further work.

The term GO:0005549 (odorant binding) was shared by all libraries (Supplementary Table S2). Additionally the ability to perceive environmental light is important for the normal growth and development of many organisms and the GO:0009785 (blue light signalling pathway) was also shared among larval, female and male libraries (Supplementary Table S2).

### Manual gene curation

The transcript sequences allowed us to manually improve the gene models for 16 ORs, two GRs, 12 IRs, seven OBPs, three CSPs, two ammonium transporters, four TRPs, two PPKs, and *Piezo* and *narrow abdomen* genes. In addition two IRs identified as pseudogenes in the genome (*Ir75d* and *Ir75g* with VectorBase codes RPRC000105 and RPRC017356, respectively) had coding sequences in the antennal transcriptome that allowed their modelling as intact genes.

Additional sensory genes were identified in the *R. prolixus* genome by tBLASTn searches against the VectorBase database using orthologous sequences from other insects^22^,^23^,^24^,^25^. The sequences of these genes were subsequently compared to our transcriptome assemblies and corrected or extended. Five CheB protein sequences were identified in the *R. prolixus* genome (VectorBase codes: RPRC004662, RPRC004663, RPRC004664, RPRC004665 and RPRC004666) and annotated as *CheB1*-*CheB5* respectively (Supplementary Fig. S1). The sequence of *CheB1* (RPRC004662) was manually edited. No sequence belonging to the CheA protein family was found in the bug genome.

Only one SNMP sequence (RPRC013907) had been annotated in the bug genome^18^, and our new searches of the genome identified four additional ones: *Snmp1b* (RPRC013910); *Snmp1c* (RPRC000399); *Snmp1d* (RPRC002720); and *Snmp*2 (RPRC002754) (Supplementary Fig. S2). Several other sequences discovered through tBLASTn searches against the VectorBase had to be manually edited according to our transcriptome assemblies. Another 13 members of the SCRB/CD36 protein family were identified and 9 of their models improved (Supplementary Fig. S2). A new ammonium transporter was identified: *Amt1* (RPRC006389). The previously identified ammonium transporter (*Amt*)^26^ was now annotated as *Amt3* according to tBLASTn results. The sequences of *Rh50* and both *Amt*s were manually edited after comparing them to our transcriptome assemblies.

In the case of TRP receptor genes, six new members and a new *TRPA1* isoform were identified and annotated (*TrpA5a*; *TrpA5b*; *TrpA5c*; *Pkd2*; *TRPM*; and *TRPML*), raising the number of *R. prolixus* TRPs to 14. The sequence described as *pyrexia* in the genome paper (identified as RPRC000570) was properly annotated as *TRPA5a* based on its sequence similarity to other TRPA5 members. Additionally, the sequence of *TRPML* needed to be manually edited after comparison with the originally described gene. Four new PPK receptors were identified, bringing the total of *R. prolixus* PPKs to ten. Finally, new orthologous sequences of known insect mechanoreceptor genes were identified: *mrityu* (RPRC014507), two type B chloride channels (RPRC0065358 and RPRC013530), and three NMDA receptors (RPRC000296; RPRC006099; and RPRC001831). The sequences of *mirtyu* and both *chloride channel-b* genes were manually edited after comparison to our transcriptome assemblies. The nucleotide sequences of all genes analysed in this work are included in Supplementary Dataset S1, and their VectorBase predicted protein codes and their functional annotations are detailed in Supplementary Table S3. An edited version of the *R. prolixus* genome generic file format (GFF) was created and all the adjustments included (see Supplementary Dataset S2).

### Mapping and transcript prediction

The Illumina reads were mapped to the modified version of the RproC1 *R. prolixus* genome assembly (see Material and Methods section) to analyse gene expression profiles. The BioProject PRJNA281760 in the Sequence Read Archive at NCBI contains all the RNA-Seq reads produced in this study. A high proportion of reads mapped to the *R. prolixus* genome, being 92% for larval, 94.6% for female and 94.3% for male libraries. A total of 17,190 genes and 17,353 isoforms were predicted by Cufflinks-Cuffmerge in the consensus transcriptome based on the three conditions studied.

### Pair-wise transcriptome comparisons

**Figure 1:**
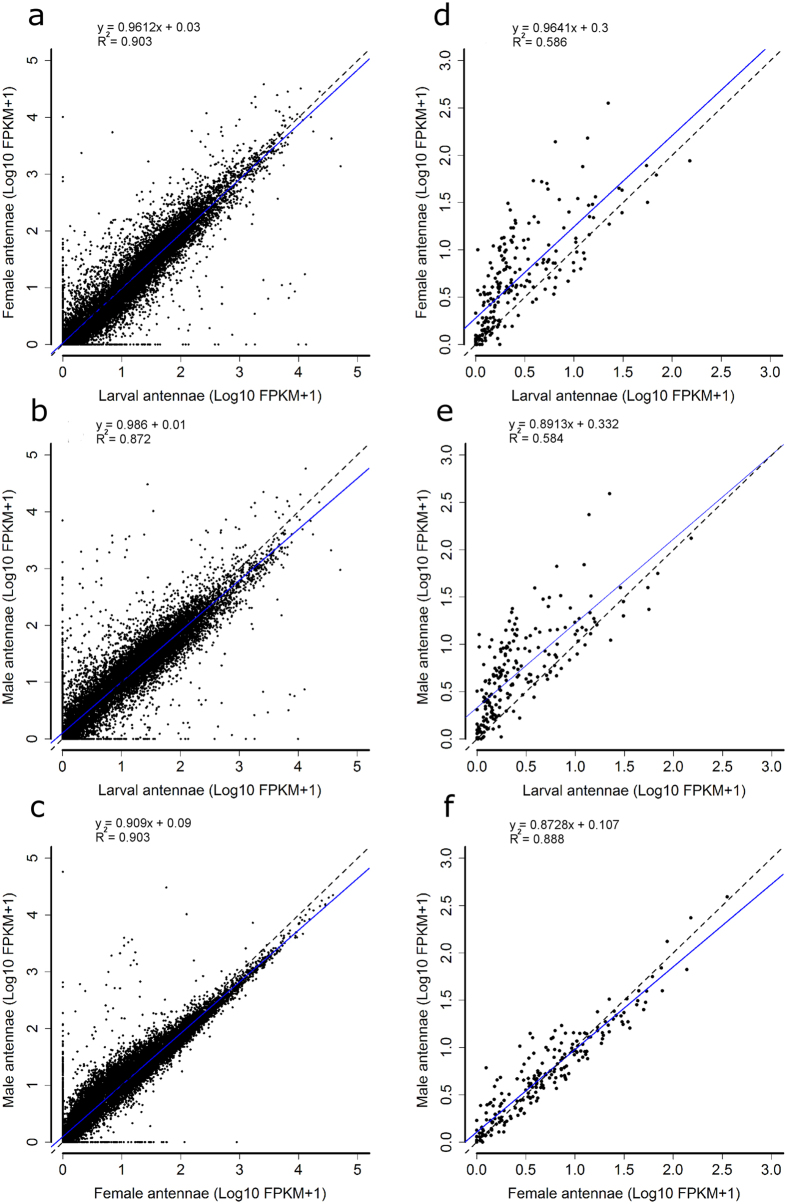
Pair-wise comparison of the three transcriptome libraries. On the left, comparison of expression levels of the 17,190 genes predicted: between larval *vs*. adult female transcriptomes (**a**); larval *vs*. adult male transcriptomes (**b**) and; female *vs*. male adult transcriptomes (**c**). On the right, comparisons of the expression levels of a set of 217 sensory receptor genes between the larval *vs.* adult female transcriptomes (**d**); larval *vs*. adult male transcriptomes (**e**) and; between female *vs*. male adult transcriptomes (**f**). Dotted lines indicate 1:1 gene expression relationship between the compared antennal transcriptomes, while linear regression analyses were marked with blue solid lines.

The expression levels of all transcripts (represented as Log10 of FPKM +1) were compared in pair-wise regression analyses and visualized using scatter plots (Fig. 1). The comparisons between larval *vs*. female (Fig. 1a, regression slope coefficient = 0.9612, R^2^ = 0.903) and larval *vs*. male (Fig. 1b, regression slope coefficient = 0.986, R^2^ = 0.872) antennal transcriptomes showed an overall similar gene expression for antennae of larvae and adults. Regarding male and female antennae, both transcriptomes showed a similar global expression profile (Fig. 1c, regression slope coefficient = 0.909, R^2^ = 0.918). Interestingly, several transcripts showed higher expression in the male than in the female transcriptome (those with Log10 FPKM +1 lower than 2.5, Fig. 1c).

### qRT-PCR validation of RNA-Seq

The expression levels of 25 genes and two reference genes (Supplementary Table S4) were evaluated by means of qRT-PCR to validate these RNA-Seq expression data. Transcript abundances obtained through both techniques were strongly correlated when larval *vs*. male results (Spearman Correlation; r = 0.88, p < 0.0001) and larval *vs*. female results (Spearman Correlation; r = 0.81, p < 0.0001) were compared (see Supplementary Fig. S6 and Table S5).

### Expression of sensory genes

#### Overall expression profiles

The expression levels of a set of 217 transcripts belonging to OR, IR, GR, PPK, TRP, CheB and SNMP protein families were compared in pair-wise regression analyses and visualized using scatter plots represented as Log10 FPKM +1 (Fig. 1). This set of transcripts showed higher expression levels in antennal transcriptomes of female (Fig. 1d, regression slope coefficient = 0.9641, R^2^ = 0.586) and male (Fig. 1e, regression slope coefficient = 0.893, R^2^ = 0.584) bugs, when compared to those seen in the larval transcriptome. As observed in the overall comparison, the expression levels of this set of sensory receptor genes were similar in the antennae of male and female adult bugs (Fig. 1f, regression slope coefficient = 0.8728, R^2^ = 0.888).

#### Odorant receptors

**Figure 2:**
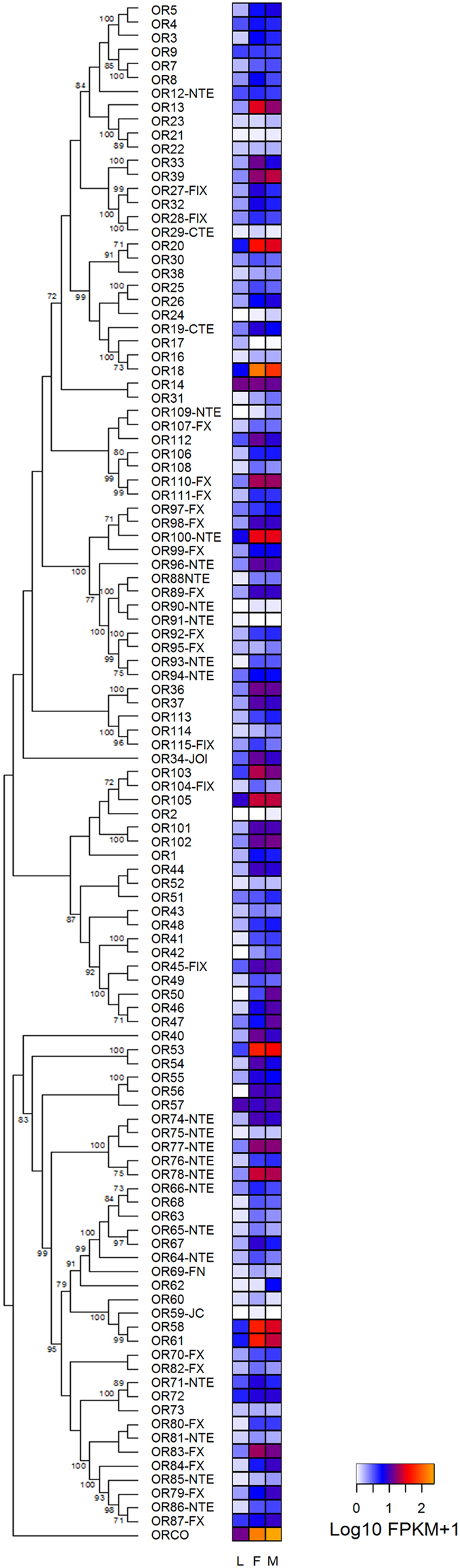
Phylogenetic relationship of odorant receptors (ORs) and heat map comparing their expression levels in antennae of larvae (L), female (F) and male (M) adults of *R. prolixus*. The OR evolutionary history was inferred using the Maximum Likelihood method in MEGA6.0. The bootstrap consensus tree (which topology is displayed) was inferred from 1,000 pseudo-replicates. Only bootstrap values higher than 70 are displayed. An initial tree was obtained by applying the Neighbor-Joining method to a matrix of pairwise distances estimated using JTT and F models. Expression levels (represented as Log10 FPKM +1) are depicted with a colour scale in which white and orange represent lowest and highest expression, respectively. Suffixes to gene names are explained in the Methods section.

The *R. prolixus* genome contains 116 ORs, including *Orco* and five pseudogenes. Our transcriptome analyses suggest that at least 83 ORs (75%) present transcripts in *R. prolixus* antennae (Table 1). As expected, the *Orco* gene had the highest expression in all life stages (Fig. 2), with FPKM values of 13, 152 and 233 in larvae, female and male antennae, respectively (Supplementary Table S6). The main feature seen in the expression pattern shown by the OR heat map is an apparent increase in expression from larval antennae to those of adult bugs (Fig. 2). According to the Cuffdiff output (FPKM values) 72 out of 111 odorant receptors (65%) seem to have increased expression after imaginal moult. Half of the ORs had no or very low expression in larval antennae, while most seem to be expressed in the antennae of adult bugs. Indeed, several OR genes with low expression in larval antennae (FPKM <1) seem to have increased expression in the antennae of adults (at least a fourth of the 111 ORs - Supplementary Fig. S3 and Table S6). In the case of ORs with FPKM values > 1 in larval antennae, a similar proportion of genes seem to have increased expression in adult antennae (see Supplementary Fig. S3 and Table S6). Consistently, statistical analysis based on the edgeR package showed that while 6 ORs increased their antennal expression significantly in both sexes after the imaginal moult (Supplementary Table S7), five (*Or18*; *Or33*; *Or40*; *Or54;* and *Or58*) did so exclusively in female antennae and, six (*Orco; Or39*; *Or46*; *Or62*; *Or84;* and *Or109*) augmented only in male antennae (Supplementary Table S7). Considering all these cases (all belonging to the highest fold-change category in Supplementary Fig. S3), almost a sixth of *R. prolixus* ORs had a significant increase in their expression in the antennae of adult bugs (FDR adjusted p-value < 0.05, Supplementary Table S7), while just as many others had borderline increased expression (FDR adjusted p-value < 0.1, Supplementary Table S7).

#### Ionotropic receptors

**Figure 3:**
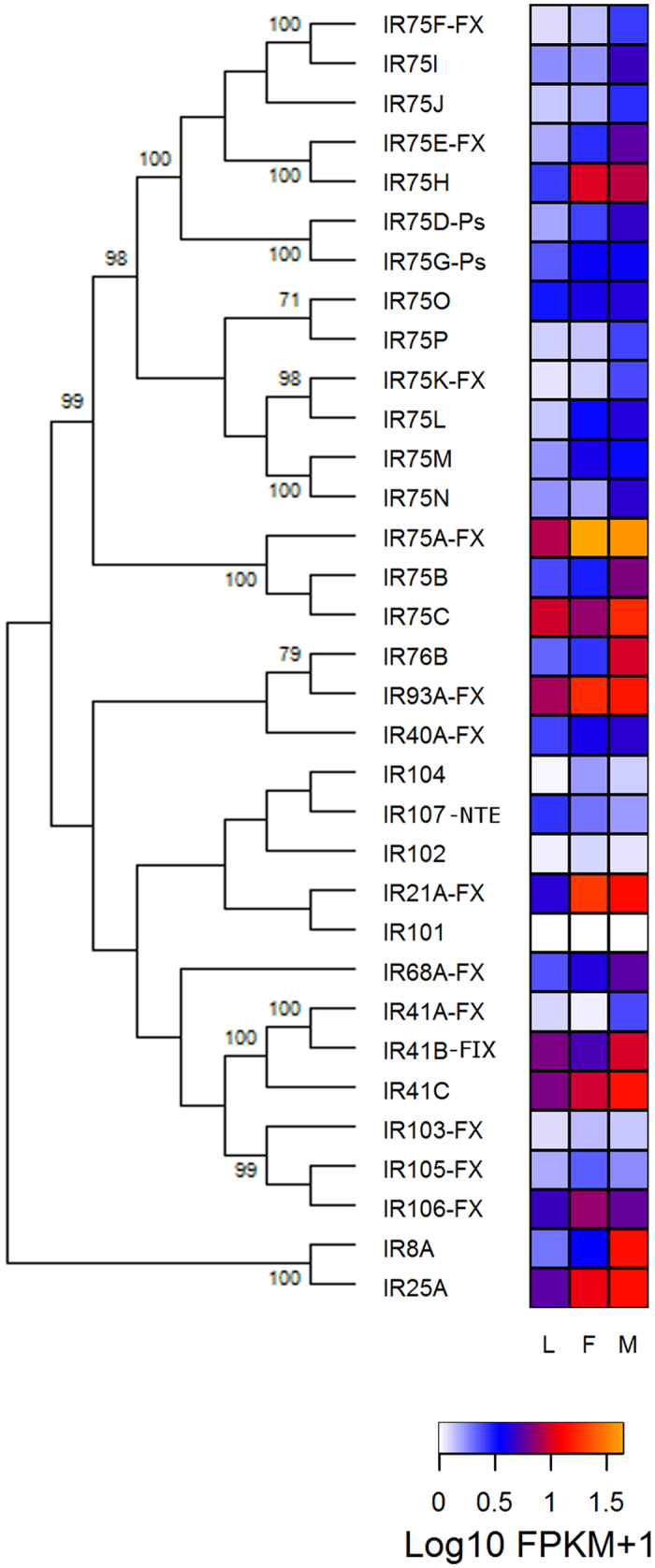
Phylogenetic relationship of ionotropic receptors (IRs) and heat map comparing their expression levels in antennae of larvae (L), female (F) and male (M) adults of *R. prolixus*. The IR evolutionary history was inferred using the Maximum Likelihood method in MEGA6.0. The bootstrap consensus tree (which topology is displayed) was inferred from 1,000 pseudo-replicates. Only bootstrap values higher than 70 were displayed. Initial tree was obtained by applying the Neighbor-Joining method to a matrix of pairwise distances estimated using the WAG and F models. Expression levels (represented as Log10 FPKM +1) were depicted with a colour scale in which white and orange represent lowest and highest expression, respectively. Suffixes to gene names are explained in the Methods section.

Considering our different expression criteria, almost all IR receptors seem to be expressed in bug antennae (Table 1). As seen for ORs, many IRs seem to have increased expression in the antennae of adult bugs (Fig. 3). A similar increase was observed for the three IR co-receptors, reinforcing the apparent effect of imaginal molt on IR gene expression. Interestingly, the most expressed IR gene was *Ir75a* (7.5, 43, and 39 FPKM in larvae, female and male antennae, respectively), and not the IR co-receptor genes, which showed a maximum FPKM of 13 (Supplementary Table S6). A subset of IRs (*Ir41c*; *Ir40*; *Ir93a*; *Ir106*; *Ir75g*; and *Ir75o*) showed expression profiles similar to that of *Ir25a* (Fig. 3). Co-receptors *Ir8a* and *Ir76b* showed similar expression profiles, with apparent increases in male antennae (Fig. 3 and Supplementary Fig. S4). Furthermore, 11 out of 16 genes belonging to the IR75 expansion also seem to have higher expression in male antennae (Fig. 3 and Supplementary Fig. S4).

Almost a third of the IR receptor genes do not seem to be expressed in larval antennae (FPKM value < 1) (Supplementary Table S6). Many of these seem to present increased expression in males compared to larvae, with a highlight for *Ir8a* which presented a 13.6-fold increase (Supplementary Table S6). The antennae of larvae presented 15 IR receptor genes with FPKM values > 1 (Supplementary Table S6), a third of which seem to have increased transcript abundance in male antennae (Supplementary Fig. S3).

#### Gustatory receptors

**Figure 4:**
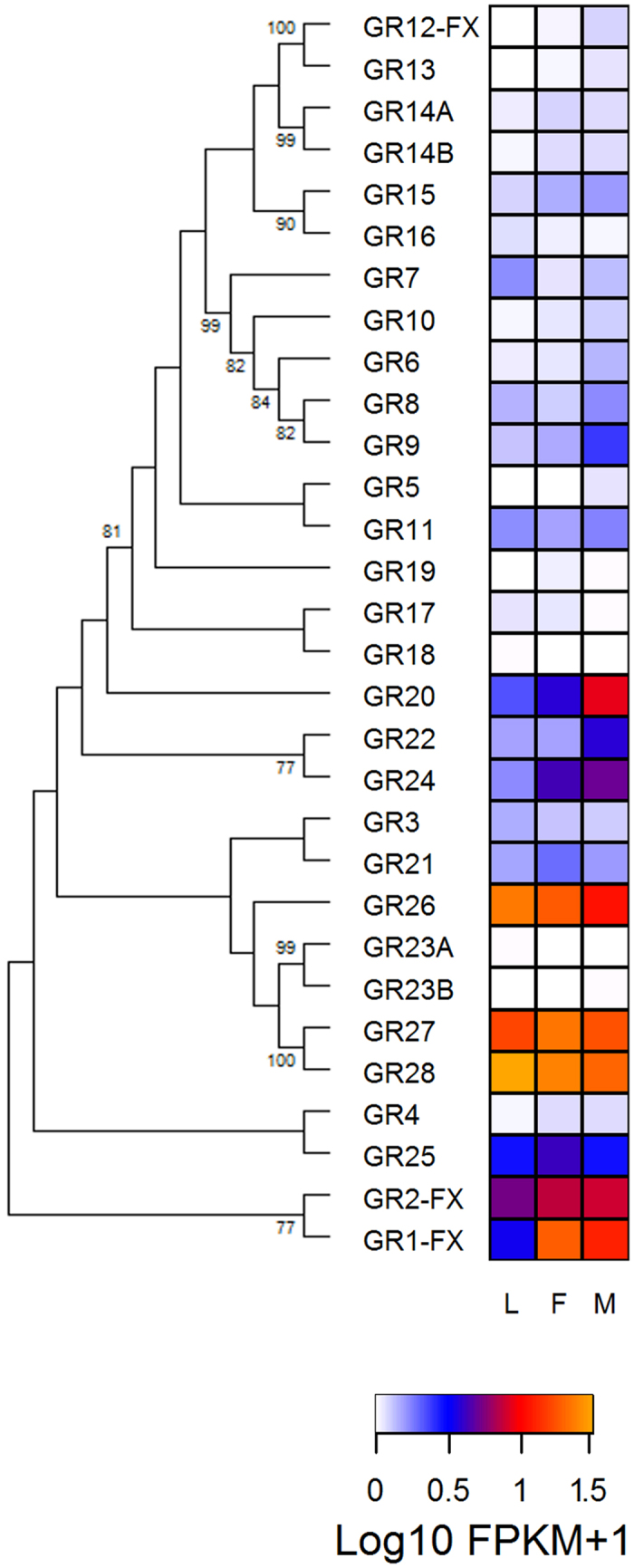
Phylogenetic relationship of gustatory receptors (GRs) and heat map comparing their expression levels in antennae of larvae (L), female (F) and male (M) adults of *R. prolixus*. The GR evolutionary history was inferred using the Maximum Likelihood method in MEGA6.0. The bootstrap consensus tree (which topology is displayed) was inferred from 1,000 pseudo-replicates. Only bootstrap values higher than 70 were displayed. Initial tree was obtained by applying the Neighbor-Joining method to a matrix of pairwise distances estimated using JTT and F models. Expression levels (represented as Log10 FPKM +1) were depicted with a colour scale in which white and orange represent lowest and highest expression, respectively. FX: gene model corrected based on the *de novo* transcriptome assembly.

BLASTn searches against our transcriptome assemblies suggest that only nine GRs were expressed in the antennae. Table 1 shows that this number can rise to 20 depending on the criteria used. The level of antennal expression seemed to relate to gene distribution in the phylogenetic tree (Fig. 4). The expression of *Gr26, Gr27*, and *Gr28* was relatively high (Fig. 4) in all conditions studied (Supplementary Dataset S3). The genes *Gr1* (orthologue of *D. melanogaster* fructose receptor), *Gr2, Gr20* and *Gr24* seemed to have increased expression in the antennae of adult bugs (Fig. 4,). Most GRs (22/28) showed FPKM values < 1 in the three conditions tested (Fig. 4 and Supplementary Dataset S3).

#### Odorant binding proteins and chemosensory proteins

**Figure 5:**
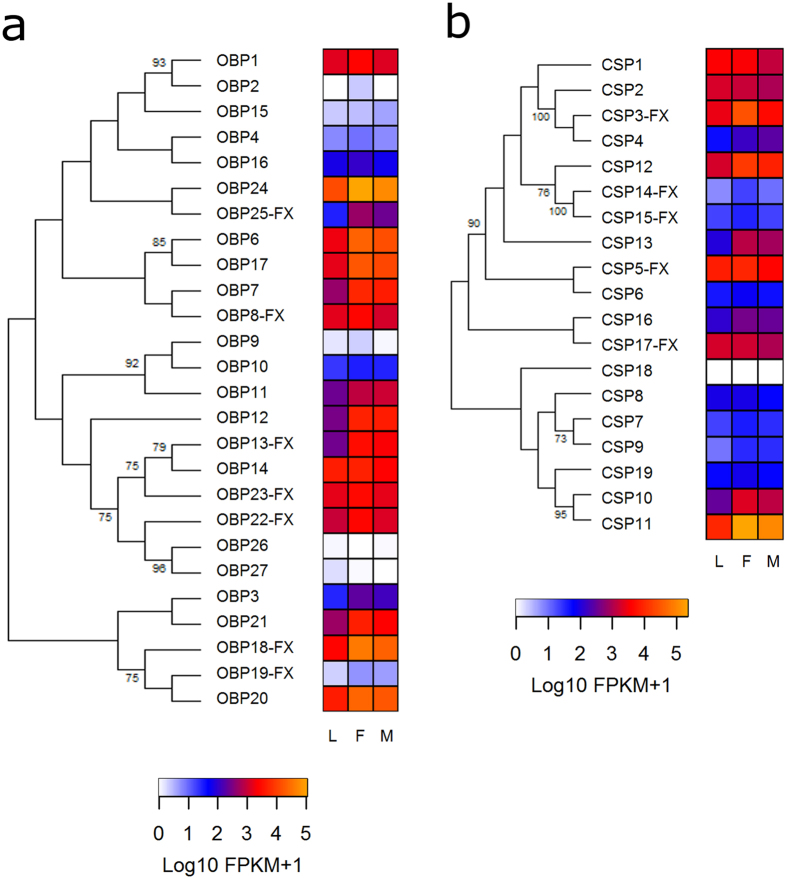
Phylogenetic relationship of odorant binding proteins (OBPs) and chemosensory proteins (CSPs) and their corresponding heat maps comparing their expression levels in antennae of larvae (L), female (F) and male (M) adults of *R. prolixus*. The OBP and CSP evolutionary histories were inferred using the Maximum Likelihood method in MEGA6.0. The bootstrap consensus trees (which topologies are displayed) were inferred from 1,000 pseudo-replicates. Only bootstrap values higher than 70 were displayed. Initial trees were obtained by applying the Neighbor-Joining method to a matrix of pairwise distances estimated using a LG model. Expression levels (represented as Log10 FPKM +1) were depicted with a colour scale in which white and orange represent lowest and highest expression, respectively. FX: gene model corrected based on the *de novo* transcriptome assembly.

A total of 27 OBPs and 19 CSPs have been annotated in the *R. prolixus* genome and most of them were expressed in bug antennae (Table 1). As expected from studies in other insects, most OBPs and CSPs showed very high levels of expression (Fig. 5), up to 100X higher than *Orco*, the most highly expressed odorant receptor. Expression patterns of both protein families seemed to correlate with their clusterization into the corresponding phylogenetic trees (Fig. 5). Many of these genes did not show relevant expression increases after imaginal moult, even though a few of them were more highly expressed in the antennae of adults (Fig. 5). This was the case for *Obp12* for which expression was significantly increased in the antennae of males and females (Supplementary Table S7). Besides, *Obp6, Obp18* and *Obp25* had increased expression in the antennae of female bugs when compared to those of larvae (Supplementary Table S7). A single CSP gene (*Csp11*) showed significantly increased expression in the antennae of females when compared to those of larvae (Supplementary Table S7).

#### CheB, SNMP/CD36 and ammonium transporter proteins

**Figure 6:**
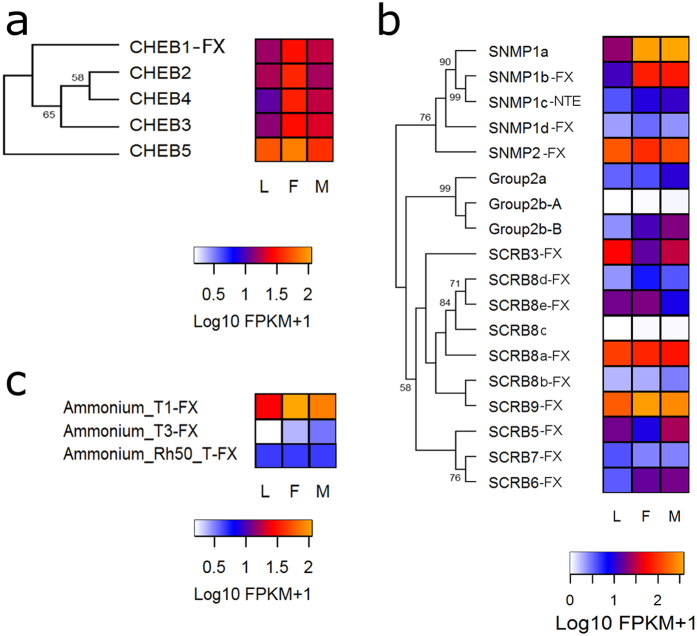
Phylogenetic relationships of CheB (**a**) and sensory neuron membrane protein/CD36 protein (**b**) families and their corresponding heat maps. Heat map of ammonium transport proteins (**c**). Heat maps compare expression levels in antennae of larvae (L), female (F) and male (M) adults of *R. prolixus*. The evolutionary history of CheB and SNMP/CD36 protein families were inferred using the Maximum Likelihood method in MEGA6.0. The topologies of the CheB (highest log likelihood −1123.0068) and SNMP/CD36 trees (highest log likelihood −6147.2094) are shown. The percentage of trees in which the associated taxa clustered together is shown next to the branches. The initial trees for the heuristic search were obtained by applying the Neighbor-Joining method to a matrix of pairwise distances estimated using WAG and F models models (for CheB proteins) and L and G models (for SNMP/CD36). Expression levels (represented as Log10 FPKM +1) were depicted with a colour scale in which white and orange represent lowest and highest expression, respectively. FX: gene model corrected based on the *de novo* transcriptome assembly; NTE: N-terminus region is missing.

All CheB genes are expressed in the antennae (Table 1) and interestingly, most of them showed higher expression in female antennae (Fig. 6a). In the case of the SNMP/CD36 protein family, all SNMPs were expressed in antennae (Table 1). *Snmp1a* and *Snmp1b* showed higher expression in adult antennae, while *Snmp2* presented high expression in antennae of all developmental stages studied (Fig. 6b and Supplementary Table S7). *SCRB8a* and *SCRB9* were the scavenger-like receptors showing highest expression (Fig. 6b). The three ammonium transporters were expressed in the antennae (Table 1 and Fig. 6c); especially *Amt1* with high expression in antennae of all developmental stages studied (Fig. 6c).

#### Pickpocket receptors

**Figure 7:**
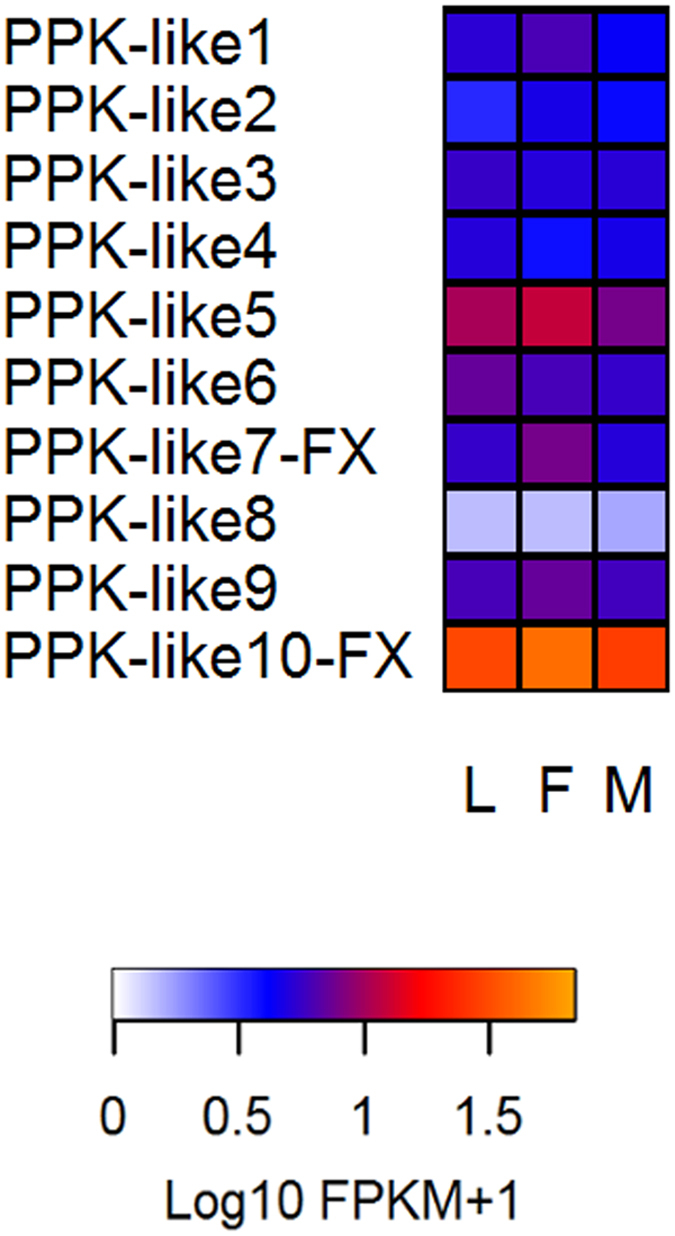
Heat map of pickpocket receptor (PPK) genes comparing their expression levels in antennae of larvae (L), female (F) and male (M) adults of *R. prolixus*. Expression levels (represented as Log10 FPKM +1) were depicted with a colour scale in which white and orange represent lowest and highest expression, respectively. FX: gene model corrected based on the *de novo* transcriptome assembly.

All *R. prolixus* PPKs (i.e., ten) were found to be expressed in the antennae (Table 1). The highest expression found for this gene set belonged to the gene named *ppk-like10* (Fig. 7 and Supplementary Dataset S3). Overall, the expression levels found for most members of this gene family tended to be lower than those obtained for other sensory receptors.

#### Transient potential receptor and mechanoreceptor genes

**Figure 8:**
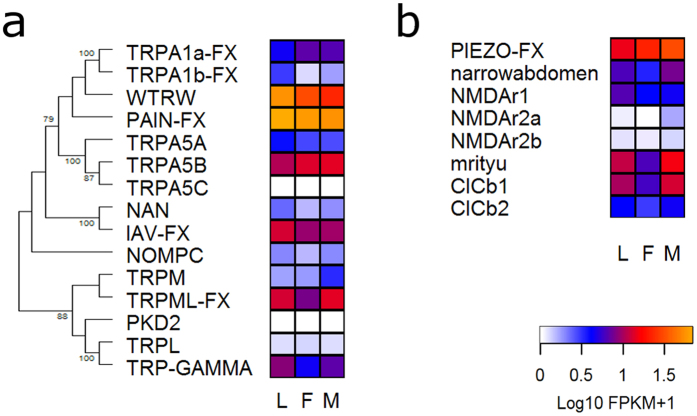
Phylogenetic relationship of transient potential receptor (TRP) genes and their corresponding heat map (**a**). Heat map of potential mechanoreceptor genes (**b**). Heat maps compare the expression levels in antennae of larvae (L), female (F) and male (M) adults of *R. prolixus*. The TRP evolutionary history was inferred using the Maximum Likelihood method in MEGA6.0. The bootstrap consensus tree (which topology is displayed) was inferred from 1,000 pseudo-replicates. Only bootstrap values higher than 70 were displayed. The initial tree was obtained by applying the Neighbor-Joining method to a matrix of pairwise distances estimated using WAG and F models. Expression levels (represented as Log10 FPKM +1) were depicted with a colour scale in which white and orange represent lowest and highest expression, respectively. FX: gene model corrected based on the *de novo* transcriptome assembly.

At least nine out of 15 TRPs showed expression in all libraries (Table 1), with *painless* and *waterwitch* showing the highest levels (Fig. 8a and Supplementary Dataset S3). Most other TRPs had low or intermediate expression with no clear tendency of increase in the antennae of any particular stage.

In the case of putative mechanoreceptors, *piezo* deserves mention as the gene showing the highest expression. Overall, males tended to have higher expression of mechanoreceptor genes compared to females (Fig. 8b).

#### Detoxification enzymes

Sixteen CYP4 genes showed FPKM values higher than 1 in at least one library (Supplementary Dataset S3). Five CYP4 genes presented high transcript abundances in all libraries (Supplementary Fig. S5). Besides, several genes belonging to this clade showed higher expression in libraries obtained from adult antennae (Supplementary Fig. S5). Intriguingly, some members of this clade had higher expression in larval or female antennae.

Seven secreted esterase genes presented FPKM values > 1 in at least one library (Supplementary Dataset S3). Few of these enzymes presented high expression in all libraries, while a small number presented higher expression profiles in the antennae of larvae, adults or females (Supplementary Fig. S5).

## Discussion

The present study represents the first antennal transcriptome sequenced for a Chagas disease vector and one of the few existing for hemimetabolous insects. It improves our characterization of the sensory repertoire of *R. prolixus*, which was initially described in the recently published genome paper^18^, allowing more robust functional studies.

Antennal transcriptomes of several insect species have been recently published, including those of tree-killing beetles^27^ or human disease vectors^28^,^29^. Most of these studies have been carried out with holometabolous insects and focused on imaginal antennae. In the case of hemimetabolous insects, antennal transcriptome analyses are fewer and many aspects of the molecular bases of their sensory physiology are still unknown. One of the main outcomes of this study is that these insects seem to increase the expression of several chemosensory genes after reaching the adult phase, as significantly increased expression was observed for diverse odorant receptor, OBP, CSP and SNMP genes which may serve to enhance adult chemosensory abilities. In contrast, antennal transcriptomes of male and female adults showed similar expression profiles (Fig. 1). A few exceptions showing higher expression in males or females may be related to functions that are differentially relevant for one of the sexes. These genes should be further studied as candidates mediating sexually dimorphic physiological activities, such as pheromone detection.

The number of olfactory receptors apparently expressed in the antennae of *R. prolixus* (88 ORs and 22 IRs, see Table 1) is much higher than that of glomeruli (22) identified in the antennal lobe of this species^17^. An opposite case has been observed for locusts^30^, suggesting that the prevailing view of “one receptor-to-one glomerulus” olfactory organization scheme may not be generalized, and alternative neuronal organization schemes for the olfactory system may co-exist in the diverse insect clades.

We characterized the antennal expression levels of all known *R. prolixus* sensory-related genes and compared their transcript abundances in the antennae of 5^th^ instar larvae and adults for the first time in a hemimetabolous insect. As said above, increased expression from larvae to adults was observed (Fig. 1d and e) for many chemosensory-related genes. Indeed, sexual behaviour, oviposition and flight are activities exclusively performed by adult bugs, and their incorporation into bug biology seems to correlate with these peripheral modifications happening at the molecular level. Consistently, this increase in receptor gene expression also seems to correlate with the greater number of chemosensilla reported for the antennae of adult insects of this species^13^,^14^,^15^. As observed in other insects^27^,^28^, *Orco* was the odorant receptor showing highest expression in bug antennae (Fig. 2) and the imaginal increase in *Orco* expression seen in our transcriptome was previously reported for *R. prolixus* antennae based on qRT-PCR^31^. Interestingly, only one OR presented differential expression between sexes (Supplementary Table S7), while the bulk of the chemosensory repertoire seemed to remain mostly similar. A similar case was observed in an antennal transcriptome from the blowfly *Calliphora stygia*^32^. Additional functional studies, such as RNAi or de-orphanization through heterologous gene expression would be necessary to understand the role of these genes in adult bug biology. In the case of ORs and other sensory receptor genes which presented similar expression in larval and adult antennae, a potential role in host, shelter and aggregation signal detection deserves to be considered in future genetic studies.

As seen in the results section, a parallel increase of IR transcript abundance also seemed to occur after imaginal moult (Fig. 3). Given that *Ir75a* was the most highly expressed IR in adult bug antennae (Fig. 3), even higher than *Ir25a*, it seems as a potential candidate for functional studies. Interestingly, the antennae of male bugs showed increased expression of some IR co-receptors (*Ir76b* and *Ir8a*), part of the *Ir75* gene subfamily, as well as other specific receptor genes like *Ir41a* and *Ir41b* (Fig. 3 and Supplementary Fig. S4). Whether the proteins encoded by these genes are related to male-enhanced functions needs to be explored.

As it might be expected given their primary role in gustation, most GRs showed low or no expression in bug antennae, in agreement with observations in other insects^28^,^33^,^34^. It is likely that these receptors are involved in chemoreception in bug tarsi or proboscis. Indeed, triatomines use contact chemo-signals to mark shelters^35^ and to recognize sexual partners^36^. Two groups of GRs, nevertheless, showed interesting expression patterns in antennae, these being *Gr1* and *Gr2* primarily being expressed in adults, and *Gr26–28* highly expressed in all stages studied (Fig. 4). These genes may relate to triatomine behaviours known to be based on contact chemostimuli, even though other unexpected roles cannot be discarded.

Antennal transcriptome studies performed with other insects showed similar expression patterns for OBPs and CSPs^28^,^33^. Bug OBP and CSP numbers (27 and 19, respectively) are much smaller than those of ORs and IRs (111 and 33, respectively). This suggests that their role as odour carriers is not necessarily linked to all specific receptors. The increase in expression observed for ORs and IRs at the adult stage had a correlate in the case of several odour-carrying proteins, making them potential candidates for mediating adult-related functions.

CheB proteins have been related to the detection of cuticular hydrocarbons in *Drosophila*^37^ and as said above, compounds of this type have been shown to mediate bug communication^35^,^36^. Nevertheless, no behavioural or functional evidence can be related to date to the increased expression observed for *R. prolixus* female bugs (Fig. 6). Two SNMPs with high expression in adults (*Snmp1a* and *Snmp1b*, Fig. 6) may have relevant roles for imaginal chemosensory physiology. *Snmp1* is important for sex pheromone responses in *Drosophila* and moths^38^,^39^; however, functional data on other insect models are scarce for this protein family.

The high expression of *ppk-like10* suggests a relevant role in the sensory ecology of these bugs. Similarly, special mention should be made for *painless* and *waterwitch* genes, the most expressed TRPs (Fig. 8a). This expression profile suggests their relation with the known capacity of these bugs to detect heat^9^ and water vapour^10^,^40^, as Drosophila *painless* and *waterwitch* genes encode for heat and water vapor receptors, respectively. The relatively high expression of several mechanoreceptors (Fig. 8) seems to correspond to the known capacity of triatomines to detected vibratory signals^11^. It is worth highlighting that several genes belonging to this category seemed to have more intense expression in the antennae of male bugs, which are known to detect vibratory sex signals^11^. The homogenous expression profile observed for PPKs and TRPs in larval and adult antennae, differently from ORs and IRs, reinforces the hypothesis of a major role in the detection of salts, substrate-borne vibrations, heat or water vapour, which are mostly relevant for all triatomine developmental stages. Several secreted esterases and CYP4 clade members presented high levels of antennal expression; therefore suggesting that they may be involved in odour/pheromone degradation processes. However, it would be important to compare the expression of these genes in other bug tissues and evaluate the corresponding levels of enzymatic activity to reinforce a potential role as ODEs.

The statistical comparison of larval *vs*. female and larval *vs*. male RNA-seq data with results obtained by means of qRT-PCR for 25 selected genes demonstrated similar trends for antennal transcript abundances reinforcing the significant increase observed in the expression of several chemosensory genes in bug antennae after imaginal moult. On the contrary, transcript abundances obtained by both techniques showed a low correlation when results from female and male samples were compared. In fact, gene expression differences between sexes detected by means of both techniques were small, i.e. fold-changes obtained for 22 genes were lower than 0.30. Coincidently, absence of sexual dimorphism has been reported for the number of antennal chemosensilla and glomeruli in the antennal lobe of *R. prolixus*^16^,^17^. The similarities in male and female bug chemosensory systems may explain, at least in part, the low correlation observed when results from both techniques were compared.

The number of transcripts predicted in the antennal transcriptome (17,190) is similar to that reported (16,857) in the last version of the *R. prolixus* genome (www.vectorbase.org/organisms/rhodnius-prolixus/cdc/RproC3.1.). More than 10,000 genes were expressed in antennae, meaning that at least 60% of all genome genes were expressed, similarly as seen for *D. melanogaster* antennae^41^. Our two *de novo* transcriptome assemblies and the subsequent manual curation process have allowed validating a large number of gene models, while many additional sensory-related genes have also been identified here. An edited version of the *R. prolixus* genome generic file format (GFF) was created including these adjustments (Supplementary Dataset S2), improving the potential of future RNA-Seq analyses with this insect species.

## Methods

### Insects and RNA isolation

*Rhodnius prolixus* were obtained from a colony held at the Centro de Pesquisas René Rachou - FIOCRUZ. Insects were reared under controlled conditions at 26 ± 1 °C, 65 ± 10% relative humidity and a 12 h:12 h light/dark cycle provided by artificial lights (4 fluorescent lamps, cold white light, 6400 K, 40 W). Colony insects were routinely fed *ad libitum* with citrated rabbit blood (2.5% buffered sodium citrate, provided by the Centro de Criação de Animais de Laboratório-CECAL, FIOCRUZ), using an artificial membrane feeder. This procedure does not require approval by the institutional ethics committee, as no live vertebrates are used. Experiments were performed using 5^th^ instar larvae and female and male adult insects, all being unfed and 21 days-old when antennae were excised (adult bugs were sorted by sex at 5^th^ instar and kept virgin until their antennae were excised). A total of sixty antennae were collected per sample and used for RNA extraction for subsequent RNA-Seq library preparation. Tissues were manually homogenized using sterilized pestles and total RNA was extracted using TRIzol^®^ Reagent (Life Technologies, Carlsbad, CA, USA) according to the manufacturer’s instructions. Then, extracted RNA was resuspended in 22 μL of DEPC-treated water (Life Technologies), and its concentration determined at 260 nm using a Nanodrop (Thermo Fisher Scientific, Waltham, MA, USA). Extraction yield for the larval antennae sample was 4.5 μg (total RNA), while it reached 2.8 μg for samples of male and female adult antennae. RNA integrity and quality were assessed by means of an agarose electrophoresis gel and a Bionalyzer using the RNA 6000 Nano Kit (Agilent Technologies, Santa Clara, CA, USA).

### Illumina sequencing

Library construction and Illumina HiSeq2000 sequencing services were hired at the W. M. Keck Centre for Comparative and Functional Genomics (University of Illinois at Urbana-Champaign, IL, USA). RNA-Seq libraries were constructed using the TruSeq Stranded RNA Sample Preparation Kit (Illumina, San Diego, CA, USA). Briefly, messenger RNA was selected from one microgram of high quality total RNA. First-strand cDNA was synthesized with a random hexamer and SuperScript II (Life Technologies). Double stranded DNA was blunt-ended, 3′-end A-tailed, and ligated to indexed adaptors. The adaptor-ligated double-stranded cDNA was amplified by PCR for 10 cycles with the Kapa HiFi polymerase (Kapa Biosystems, Woburn, MA, USA). The final libraries were quantified on a Qubit (Life Technologies), and the average size, 280 nt, was determined on an Agilent bioanalyzer DNA7500 DNA chip. Individual libraries were diluted to 10 nM, and the indexed libraries were pooled in equimolar concentration. The pooled libraries were further quantitated by qPCR on an ABI 7900 (Life Technologies). The multiplexed libraries were loaded onto one lane of an 8-lane flowcell for cluster formation and sequenced on an Illumina HiSeq2000 with TruSeq SBS sequencing kits version 3. The libraries were sequenced from both ends of the molecules to a total read length of 100 nt from each end. The raw.bcl files were converted into demultiplexed fastq files with Casava 1.8.2 (Illumina).The single lane of paired-end sequencing yielded 353,628,668 total raw reads. The larval sample produced 103,016,074 reads; the female 124,995,224 reads; and the male 125,617,370 reads.

The FASTQ software (www.bioinformatics.babraham.ac.uk/projects/fastqc/) was used to assess the reads bias for the sequencing lanes as a whole. Subsequently, FASTX Toolkit (http://hannonlab.cshl.edu/fastx_toolkit/index.html) was used to trim off biased reads at the 5′ end, and then using the quality score trimmer to remove low-quality reads at the 3′ end of the read (−t 20). After trimming processes, the final number of reads was 102,457,875; 124,386,666; and 124,742,367 for larvae, female and male libraries, respectively. Using all trimmed reads obtained from the three libraries, two *de novo* transcriptome assemblies were elaborated using SOAPdenovo v.1.02^42^ and Trinity (release 2012-03-17)^43^ softwares. In the assembly carried out using SOAPdenovo, different *K-mer* sizes were assessed; 49-mer yielded the best assembly for the desired application, and was chosen to construct the de Bruijn graph. Trinity was used with default parameters, except minimum reported contig length = 100.

### Gene ontology annotation and functional enrichment analysis

Genes with FPKM value >1000 were selected in each library. Subsequently, these genes were annotated according to GO annotation using Blast2GO^44^. The Perl library GO:TermFinder^45^ was used to identify the enrichment of functional terms in sequenced data. This algorithm was used to compare genes with FPKM value >1,000 in each library to the list of functionally annotated genes in the *R. prolixus* genome. The Fischer’s Exact Test was used to check for association strength and 0.05 was defined as the minimum adjusted p-value for the functional annotation of genes. The hypergeometric distribution and the Bonferroni correction for multiple hypotheses were employed to correct p-values. Finally, the GO terms of those genes which were enriched in each library were compared between the three libraries.

### Manual gene curation

The sequences of all target genes were compared to our SOAPdenovo and Trinity assemblies using BLASTn searches. Subsequently, incomplete or inconsistent sequences found in that genome version were manually corrected/extended based on our transcriptome assemblies. Suffixes were added to gene names according to their gene model characteristics: NTE – N-terminus missing in gap, CTE – C-terminus missing in gap, INT – internal exon missing in gap, FIX – genome assembly was repaired; JOI – gene model spans scaffolds; FX - gene model repaired based on *de novo* transcriptome assemblies. Following, new GFFs (Generic Feature Format) were created for these new models and these were subsequently included in the RproC1.2 version of the *R. prolixus* genome GFF file (downloaded from http://Vectorbase.org/downloads/) which was used in the subsequent read mapping analysis. The nucleotide sequences of all genes analysed in this work and the edited GFF file are included in the Supplementary Datasets S1 and S2.

### Read mapping and differential expression analysis

The filtered and trimmed Illumina reads from the three libraries were mapped independently to the *R. prolixus* genome assembly (version RproC1.2) by means of TopHat v. 2.0.11^46^. Based on the mapped reads, the transcriptome assemblies and the gene expression estimations (in Fragments Per Kilobase of transcript per Million mapped reads or FPKM) were obtained for the three conditions using Cufflinks (v. 2.1.0). Finally, Cuffmerge was used for merging the three predicted transcriptome assemblies and the FPKM values at each predicted gene locus in the three conditions tested were compared using Cuffdiff. The Cuffdiff output (in table format) is detailed in Supplementary Dataset S3. Raw read counts produced by HTSeq (v0.6.1.p2)^47^ were normalized using the Trimmed Mean of M-value (TMM) normalization method and were then used for differential expression analyses among stages and between sexes using the edgeR package (v3.6.8)^48^. In order to identify differentially expressed genes, the FDR adjusted p-value (False Discovery Rate) < 0.05 was set as threshold to define the significance level.

### Definition of expression criteria

Three different criteria were used to determine whether sensory genes were expressed in *R. prolixus* antennae (Table 1). The first criterion was based on BLASTn searches of sensory gene sequences against the *de novo* transcriptome assemblies. Those genes with a coverage >90% and sequence identity >95% against one transcript found in the *de novo* assemblies were considered as expressed. The second criterion was based on the raw read counts obtained by means of HTseq^47^: genes with more than 10 mapped reads in at least one library were considered as expressed. The last criterion was based on FPKM values obtained from TopHat^46^: genes with an FPKM value higher than 1 in at least one library were classified as expressed.

### Expression analysis

The Cufflinks-predicted transcripts and genes and their FPKM values from larvae, female and male libraries were used for gene expression comparison. R package was used to generate scatter plots to compare expression levels (represented as Log10 FPKM +1) of each transcript in pair-wise analyses: larval transcriptome *vs*. female transcriptome; larval transcriptome *vs*. male transcriptome, and male transcriptome *vs*. female transcriptome. The same comparisons were performed using the expression levels of 217 sensory receptor genes (including all ORs, GRs, IRs, TRPs, CheBs, PPKs, SNMP and mechanoreceptors). Subsequently, a linear regression analysis was performed to calculate the slope coefficient and R^2^ values for each comparison. Heat maps showing gene expression (expressed as FPKM value +1 following by Log10 transformation) of the different protein families in the three conditions were prepared using ggplot2 (www.ggplo2.org).

### qRT-PCR validation

In order to validate RNA-Seq data and expression profiles, the expression levels of 25 genes were determined by means of the qRT-PCR technique (Supplementary Table S4). *Rhodnius prolixus* insects from the same colony, reared in the same conditions and whose antennae were excised in the same developmental/physiological conditions of those used for RNA-Seq samples were utilized for qRT-PCR validation. Each treatment (i.e., 5^th^ instar larvae, female and male adults) was replicated 6 times using 60 antennae (i.e., 30 bugs) per sample. Total RNA was extracted using 500 μL of TRIzol^®^ Reagent (Life Technologies) according to the manufacturer’s instructions. Later, each RNA sample was resuspended in 30 μL of DEPC-treated water (Life Technologies), and its concentration determined using a Qubit^®^ 2.0 Fluorometer (Life Technologies). Subsequently, all samples were treated with RQ1 RNase-Free DNase (Promega, Fitchburg, WI, USA). All treated RNA (33 μL per sample) was immediately used to synthesize cDNA using the SuperScript III Reverse Transcriptase (Life Technologies) and a 1:1 mix of Random Hexamers and 10 μM Oligo(dT)_20_ primers in a final volume of 60 μL.

For qPCR reactions, 5 μL of SYBR Green PCR Master Mix (Life Technologies) were used in the reaction mixture that also contained 0.4 μL of a 10 μM primer solution and 1 μL of cDNA sample diluted 2-fold into a final volume of 10 μL. The reactions were conducted using a ViiA™ 7 Real-Time PCR System (Life Technologies) under the following conditions: one 10 min cycle at 95 °C, followed by 40 cycles of 15 s at 95 °C, 20 s at 60 °C and 30 s at 72 °C. Following the amplification step, a melting curve analysis was performed to confirm the specificity of the reaction. In all qPCR experiments, non-template controls (NTC) were included in triplicate for each primer set to verify the absence of exogenous DNA. The PCR efficiencies (E) and repeatability (R^2^) for each primer were determined using the slope of a linear regression model. Information about primers, amplicons and calibration curves is presented in Supplementary Table S4. A total of six biological replicates using averages from three technical replicates were performed for each stage studied. The relative gene expression in larvae and female and male adult antennae was calculated using the 2^−ΔΔCt^ method. First, the gene expression levels were normalized to the geometric mean of two reference genes, *α-tubulin* and *G6PDH*, for each condition. Subsequently, expression levels of each gene were normalized to the expression levels of larvae.

Regarding RNA-Seq expression data, FPKM values for each target gene from the female and male libraries were normalized by their corresponding FPKM values in the larval library (Supplementary Table S5). In the case of male *vs*. female comparisons, FPKM values from male library were normalized by their corresponding value in female library (Supplementary Table S5). Gene expression levels obtained by RNA-Seq and qRT-PCR techniques were compared using Spearman Correlation.

## Additional Information

**Accession codes:** Read sequences from the three libraries have been submitted to the Sequence Read Archive (SRA) and NCBI under the project accesion number PRJNA281760/SRP057515 and the SRA accession numbers for the three experiments can be accessed at SRS923612/SRX1011796/SRR2001242 (antennal library from larvae); SRS923595/SRX1011769/SRR2001240 (antennal library from female adult); and SRS923599/SRX1011778/SRR2001241 (antennal library from male adult).

**How to cite this article**: Latorre-Estivalis, J. M. *et al*. The molecular sensory machinery of a Chagas disease vector: expression changes through imaginal moult and sexually dimorphic features. *Sci. Rep.* **7**, 40049; doi: 10.1038/srep40049 (2017).

**Publisher's note:** Springer Nature remains neutral with regard to jurisdictional claims in published maps and institutional affiliations.

## Supplementary Material

Supplementary Information

Supplementary DataSet 1

Supplementary DataSet 2

Supplementary DataSet 3

## Figures and Tables

**Table 1:** Number of sensory genes expressed in *R. prolixus* antennae.

Gene Family	*De novo* transcriptome assemblies*	At least 10 mapped reads in one library**	FPKM >1 in at least one library
Odorant receptors (111)	83	101	95
Ionotropic receptors (33)	32	31	29
Gustatory receptors (30)	9	19	10
Odorant binding proteins (26)	21	25	23
Chemosensory proteins (19)	17	18	18
SNMPs (5)	5	5	5
Ammonium transporters (2)	3	3	3
CheBs (5)	2	5	5
TRP receptors (14)	9	11	11
PPK receptors (10)	9	6	10
Mechanoreceptors (9)	8	9	6

^*^Those sensory genes with a BLASTn to the *de novo* transcriptome assembly with a coverage <90% and/or sequence identity <95% were classified as not expressed in the *R. prolixus* antennae.

^**^Number of mapped reads were obtained using HTseq v0.6.1.p2^47^ and are detailed in the Supplementary Table S8.

Number of genes identified in the *R. prolixus* genome for each gene family are indicated between parentheses^18^.
