# Supplementary material for: The molecular sensory machinery of a Chagas disease vector: expression changes through imaginal moult and sexually dimorphic features
Source: Sci Rep. 2017 Jan 6;7:40049. doi: 10.1038/srep40049 (PMC5216343; doi:10.1038/srep40049)
Supplement: Supplementary Information [file srep40049-s1.pdf]

The molecular sensory machinery of a Chagas disease vector: expression changes through imaginal moult and sexually dimorphic features

Jose Manuel Latorre-Estivalis; Hugh M. Robertson; Kimberly K. O. Walden; Jerônimo Ruiz; Leilane Oliveira Gonçalves; Alessandra Guarneri and Marcelo Gustavo Lorenzo

DATASETS

Dataset S1 – Nucleotides sequences of all genes studied.

Dataset S2 – Edited Generic Feature Format (GFF) file of the *R. prolixus* genome used to perform the read mapping and gene expression analysis.

Dataset S3 – Cuffdiff output including VectorBase code, genome location and FPKM values for the *R. prolixus* transcripts in the three antennal libraries. In those genes with Test status = HIDATA (too many fragments in locus), raw read counts from HTseq (Supplementary Table S7) were used to calculate their FPKM values.

Figure S1 – Molecular phylogenetic analysis of CheA and CheB protein families of *R. prolixus* and other insects.

The evolutionary history was inferred by using the Maximum Likelihood method in MEGA6.0. The topology of the tree with the highest log likelihood (-8152.8619) is shown. The percentage of trees (higher than 70) in which the associated taxa clustered together is shown next to the branches. Initial tree(s) for the heuristic search were obtained by applying the Neighbor-Joining method to a matrix of pairwise distances estimated using a WAG and F models. The analysis involved 22 amino acid sequences from *R. prolixus* (identified with RPRC and their corresponding annotation), *D .melanogaster* (Dmel); and *A. aegypti* (identified by AAEL). Sequences of *D. melanogaster* and *A. aegypti* were obtained from Xu *et al*. (2002) and Bohbot *et al*. (2014), respectively.

Figure S2 – Molecular phylogenetic analysis of SMMP/CD36 protein family of *R. prolixus* and other insects.

The evolutionary history was inferred by using the Maximum Likelihood method in MEGA6.0. The topology of the tree with the highest log likelihood (-12121.1360) is shown. The percentage of trees (higher than 70) in which the associated taxa clustered together is shown next to the branches. Initial tree(s) for the heuristic search were obtained by applying the Neighbor-Joining method to a matrix of pairwise distances estimated using a LG and F models. The analysis involved 52 amino acid sequences from *R. prolixus* (identified with RPRC and their corresponding annotation), *D.melanogaster* (Dmel); *T. castaneum* (Tcas); *A. mellifera* (Amel); and *D.pseudoobscura* (Dpse). Sequences from other insects were obtained from Nichols and Vogt (2007).

Figure S3 – FPKM increase of odorant (A) and ionotropic (B) receptor expression in female and male adult antennae compared to larval antennae.

OR and IR genes with FPKM value >1 in at least one adult condition were included. OR pseudogenes (6), genes with FPKM value = 0 in larvae (5 ORs and 1 IR), genes with FPKM<1 in

adults (12 ORs and 3 IRs) and genes with lower expression in adults compared to larval antennae (2 ORs) were not included in the figure. The figure was created using Circos v0.63-9 (http://mkweb.bcgsc.ca). Fold changes calculated based on FPKM values for each gene are detailed in Supplementary Table S6.

Figure S4 – FPKM increase of ionotropic receptor expression in male compared to female adult antennae.

IR genes with FPKM value >1 in at least one adult condition were included. Four IR genes (*Ir101*-*Ir104*) with FPKM value <1 in both libraries were not included in the figure. The figure was created using Circos v0.63-9 (http://mkweb.bcgsc.ca). Fold changes calculated based on FPKM values for each gene are detailed in Supplementary Table S6.

Figure S5 – Heat maps of the secreted esterases belonging to the detoxification/pheromone hormone processing class (A) and CYP4 clade members of the CypP450 protein family (B), comparing their expression in *R. prolixus* larval (L), female (F) and male (M) antennae.

Expression levels (represented as Log10 FPKM +1) were depicted with a color scale in which white and orange represent lowest and highest expression, respectively. All gene sequences were obtained from Schama *et al*. (2015).

Figure S6 - Correlation of transcript abundances found for larval and adult expression data using RNA-Seq and qRT-PCR.

Spearman's rank correlation coefficients (R) for each library comparison are shown in the figure. Data used to create these graphs are detailed in Supplementary Table S5.

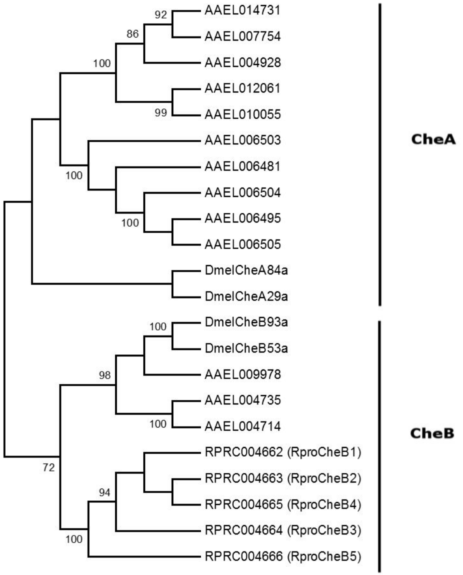

AAEL014731
AAEL007754
AAEL004928
AAEL012061
AAEL010055
AAEL006503
AAEL006481
AAEL006504
AAEL006495
AAEL006505
DmelCheA84a
DmelCheA29a
DmelCheB93a
DmelCheB53a
AAEL009978
AAEL004735
AAEL004714
RPRC004662 (RproCheB1)
RPRC004663 (RproCheB2)
RPRC004665 (RproCheB4)
RPRC004664 (RproCheB3)
RPRC004666 (RproCheB5)
92
86
100
99
100
100
100
98
100
72
94
100
CheA
CheB

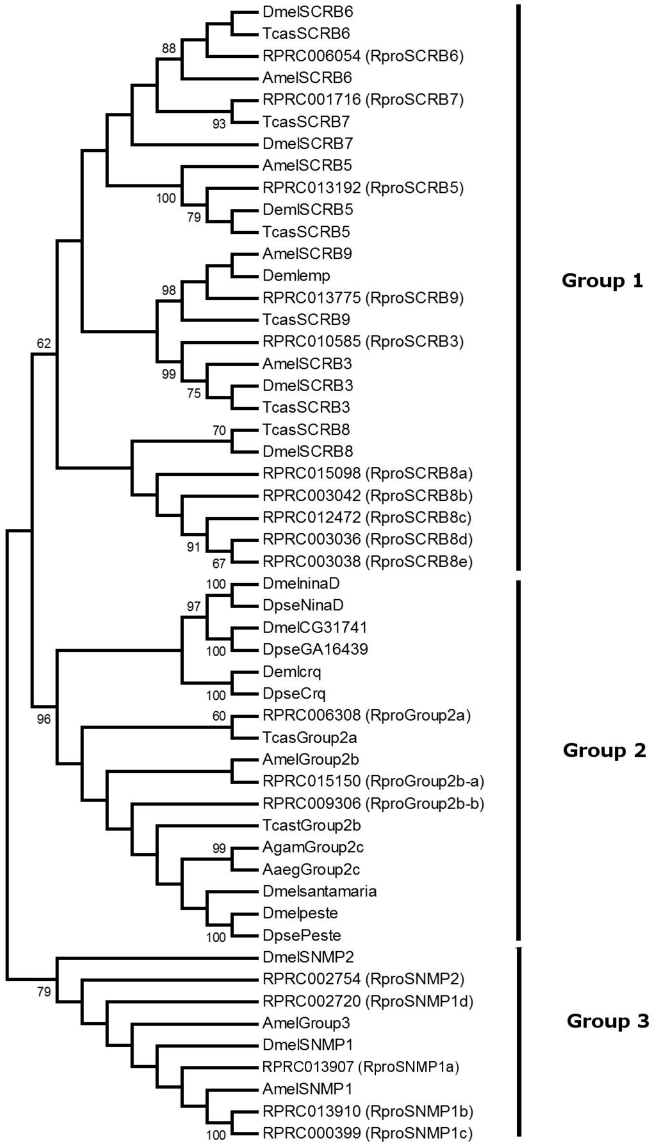
DmelSCRB6
TcasSCRB6
RPRC006054 (RproSCRB6)
AmelSCRB6
RPRC001716 (RproSCRB7)
TcasSCRB7
DmelSCRB7
AmelSCRB5
RPRC013192 (RproSCRB5)
DemlSCRB5
TcasSCRB5
AmelSCRB9
Demlemp
RPRC013775 (RproSCRB9)
TcasSCRB9
RPRC010585 (RproSCRB3)
AmelSCRB3
DmelSCRB3
TcasSCRB3
TcasSCRB8
DmelSCRB8
RPRC015098 (RproSCRB8a)
RPRC003042 (RproSCRB8b)
RPRC012472 (RproSCRB8c)
RPRC003036 (RproSCRB8d)
RPRC003038 (RproSCRB8e)
DmelninaD
DpseNinaD
DmelCG31741
DpseGA16439
Demlcrq
DpseCrq
RPRC006308 (RproGroup2a)
TcasGroup2a
AmelGroup2b
RPRC015150 (RproGroup2b-a)
RPRC009306 (RproGroup2b-b)
TcastGroup2b
AgamGroup2c
AaegGroup2c
Dmelsantamaria
Dmelpeste
DpsePeste
DmelSNMP2
RPRC002754 (RproSNMP2)
RPRC002720 (RproSNMP1d)
AmelGroup3
DmelSNMP1
RPRC013907 (RproSNMP1a)
AmelSNMP1
RPRC013910 (RproSNMP1b)
RPRC000399 (RproSNMP1c)
88
93
100
79
98
62
99
75
70
91
67
100
97
100
100
96
60
99
100
79
100
Group 1
Group 2
Group 3

a

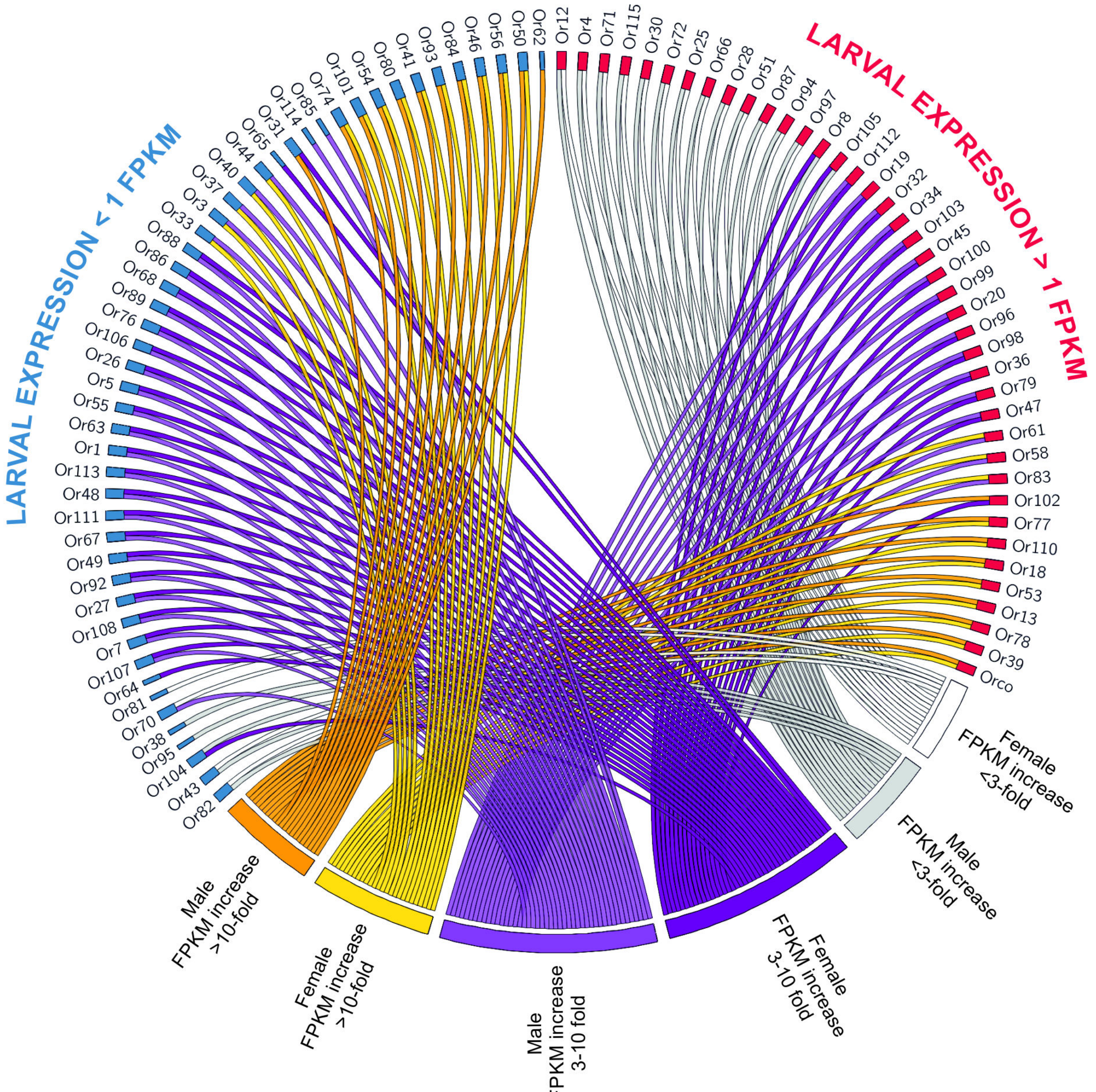
LARVAL EXPRESSION < 1 FPKM
LARVAL EXPRESSION > 1 FPKM
Or62 Or50 Or56 Or46 Or84 Or93 Or41 Or80 Or54 Or101 Or74 Or85 Or114 Or31 Or65 Or44 Or40 Or37 Or3 Or33 Or88 Or86 Or68 Or89 Or76 Or106 Or26 Or5 Or55 Or63 Or1 Or113 Or48 Or111 Or67 Or49 Or92 Or27 Or108 Or7 Or107 Or64 Or81 Or70 Or38 Or95 Or104 Or43 Or82
Or12 Or4 Or71 Or115 Or30 Or72 Or25 Or66 Or28 Or51 Or87 Or94 Or97 Or8 Or105 Or112 Or19 Or32 Or34 Or103 Or45 Or100 Or99 Or20 Or96 Or98 Or36 Or79 Or47 Or61 Or58 Or83 Or102 Or77 Or110 Or18 Or53 Or13 Or78 Or39 Orco
Male FPKM increase >10-fold
Female FPKM increase >10-fold
Male FPKM increase 3-10 fold
Female FPKM increase 3-10 fold
Male FPKM increase <3-fold
Female FPKM increase <3-fold

b

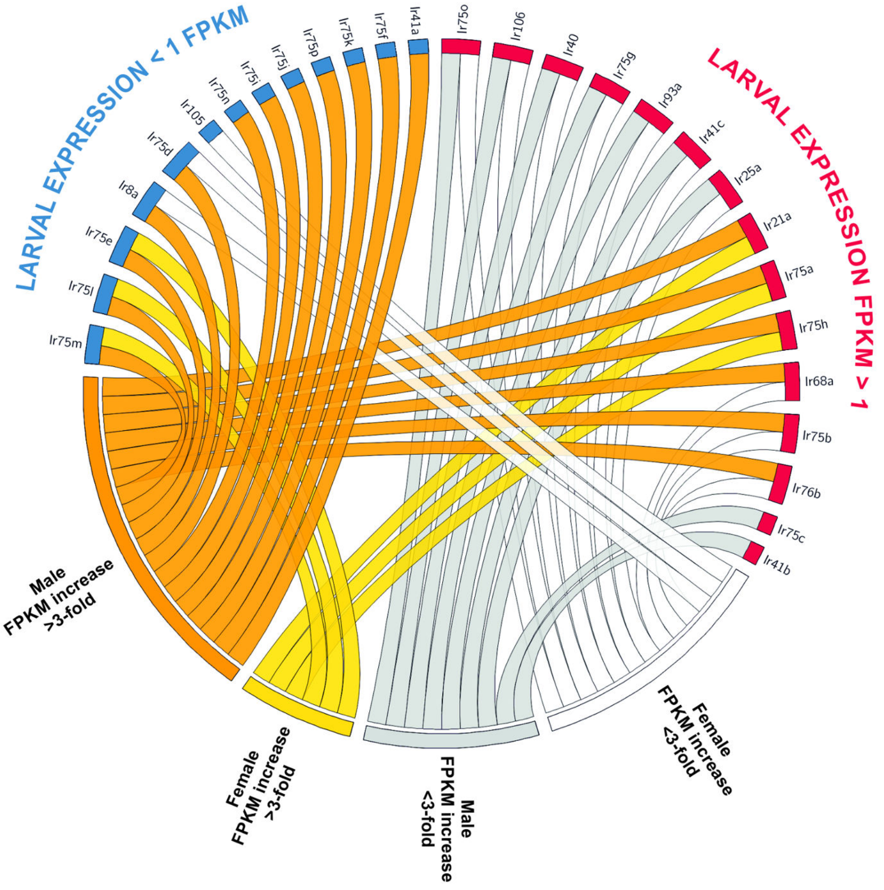
LARVAL EXPRESSION < 1 FPKM
LARVAL EXPRESSION FPKM > 1
Ir41a Ir75f Ir75k Ir75p Ir75j Ir75i Ir75n Ir105 Ir75d Ir8a Ir75e Ir75l Ir75m
Ir75o Ir106 Ir40 Ir75g Ir93a Ir41c Ir25a Ir21a Ir75a Ir75h Ir68a Ir75b Ir76b Ir75c Ir41b
Male FPKM increase >3-fold
Female FPKM increase >3-fold
Male FPKM increase <3-fold
Female FPKM increase <3-fold

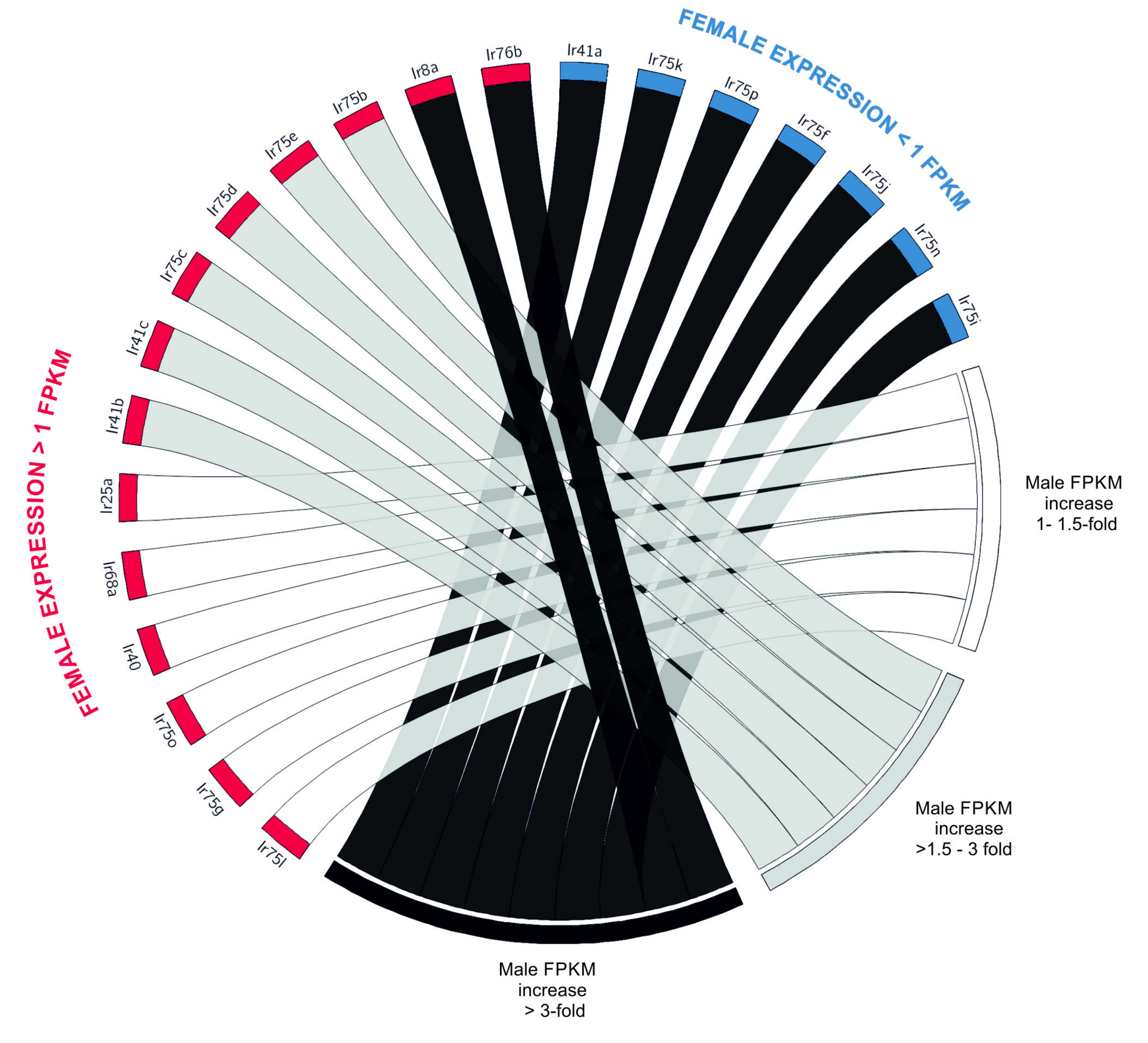
FEMALE EXPRESSION < 1 FPKM
FEMALE EXPRESSION > 1 FPKM
Ir75b
Ir8a
Ir76b
Ir41a
Ir75k
Ir75p
Ir75f
Ir75j
Ir75n
Ir75i
Ir75e
Ir75d
Ir75c
Ir41c
Ir41b
Ir25a
Ir68a
Ir40
Ir75o
Ir75g
Ir75l
Male FPKM increase 1- 1.5-fold
Male FPKM increase >1.5 - 3 fold
Male FPKM increase > 3-fold

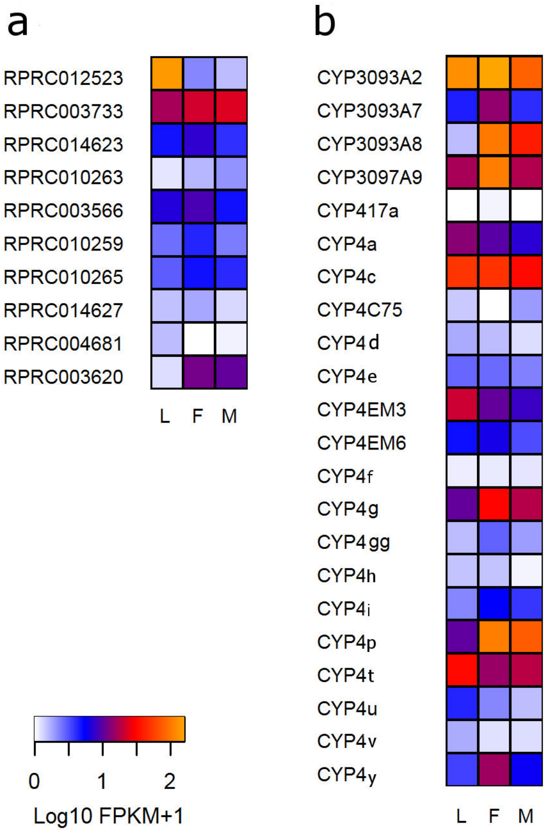
a
RPRC012523
RPRC003733
RPRC014623
RPRC010263
RPRC003566
RPRC010259
RPRC010265
RPRC014627
RPRC004681
RPRC003620
L F M
0 1 2
Log10 FPKM+1
b
CYP3093A2
CYP3093A7
CYP3093A8
CYP3097A9
CYP417a
CYP4a
CYP4c
CYP4C75
CYP4d
CYP4e
CYP4EM3
CYP4EM6
CYP4f
CYP4g
CYP4gg
CYP4h
CYP4i
CYP4p
CYP4t
CYP4u
CYP4v
CYP4y
L F M

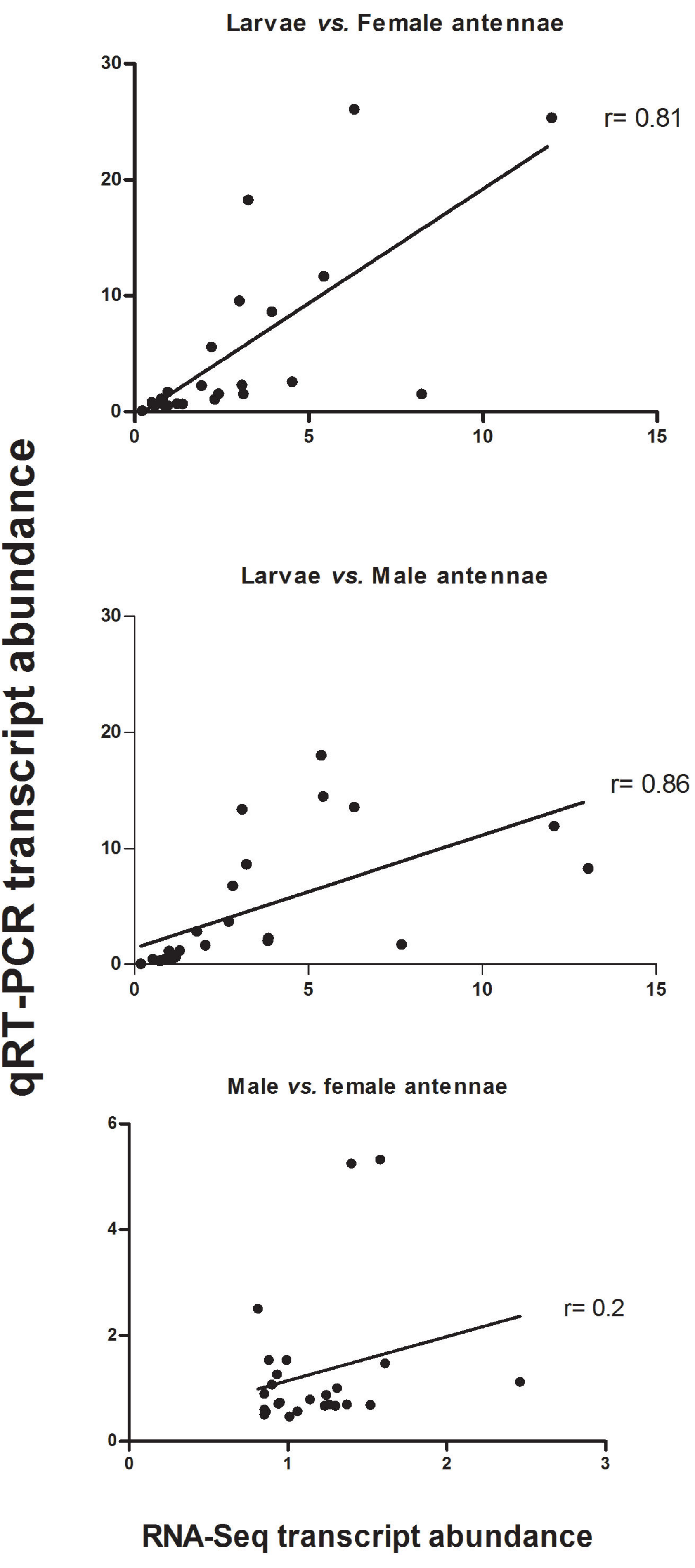
Larvae vs. Female antennae
r= 0.81
Larvae vs. Male antennae
r= 0.86
Male vs. female antennae
r= 0.2
qRT-PCR transcript abundance
RNA-Seq transcript abundance

Table S1– GO annotation terms for enriched transcripts in each library. Only those transcripts with an FPKM value >1000 were included in the functional enrichment analysis of each library. (*) P: Biological process, F: Molecular function and C: Cellular component; D.E: differentially expressed.

GO annotation terms for enriched transcripts in larval library

GO-ID	Term	Category*	FDR	P-Value	N° of genes in dataset D.E.	N° of genes in *R. prolixus* genome	Over/Under represented	VectorBase code of genes dataset D.E.	VectorBase code of genes in *R. prolixus* genome
GO:0003735	structural constituent of ribosome	F	2.26E-69	3.84E-73	63	29	over	RPRC012633, RPRC010853, RPRC003327, RPRC009716, RPRC012784, RPRC004269, RPRC013146, RPRC002520, RPRC007451, RPRC002698, RPRC010146, RPRC003768, RPRC014587, RPRC009189, RPRC013169, RPRC007328, RPRC013077, RPRC007856, RPRC012158, RPRC010378, RPRC012542, RPRC005701, RPRC010286, RPRC014419, RPRC003510, RPRC005980, RPRC014363, RPRC005724, RPRC012711, RPRC002544, RPRC006329, RPRC005430, RPRC002470, RPRC013786, RPRC009702, RPRC013851, RPRC011260, RPRC006974, RPRC011456, RPRC014620, RPRC013371, RPRC014845, RPRC011937, RPRC009337, RPRC012162, RPRC012353, RPRC012387, RPRC011596, RPRC002869, RPRC005389, RPRC013504, RPRC013167, RPRC002923, RPRC015127, RPRC015001, RPRC010405, RPRC007825, RPRC010192, RPRC000499, RPRC012354, RPRC006392, RPRC007230, RPRC006309	RPRC008799, RPRC014365, RPRC002822, RPRC009039, RPRC004296, RPRC011999, RPRC015409, RPRC012941, RPRC002505, RPRC012363, RPRC000922, RPRC006118, RPRC014793, RPRC006758, RPRC008479, RPRC010054, RPRC000972, RPRC003014, RPRC005011, RPRC009871, RPRC012072, RPRC006051, RPRC010872, RPRC003980, RPRC009873, RPRC002897, RPRC004303, RPRC001328, RPRC010809
GO:0022627	cytosolic small ribosomal subunit	C	1.05E+02	7.86E-01	4	0	over	RPRC010146, RPRC013504, RPRC002923, RPRC000499	
GO:0015986	ATP synthesis coupled proton transport	P	1.53E+02	1.20E+00	5	3	over	RPRC012073, RPRC011931, RPRC008498, RPRC010550, RPRC005917	RPRC003410, RPRC008312, RPRC005494
GO:0005200	structural constituent of cytoskeleton	F	3.29E+01	2.68E+00	6	9	over	RPRC003672, RPRC009875, RPRC005383, RPRC010283, RPRC007667, RPRC004762	RPRC014380, RPRC014377, RPRC005328, RPRC011694, RPRC008372, RPRC008343, RPRC003842, RPRC015390, RPRC000816

GO:0003924	GTPase activity	F	8.70E+02	7.98E+00	10	49	over	RPRC012142, RPRC003672, RPRC005383, RPRC010283, RPRC007667, RPRC015041, RPRC014951, RPRC011075, RPRC012140, RPRC004762	RPRC014380, RPRC013503, RPRC015134, RPRC004755, RPRC005628, RPRC011076, RPRC005457, RPRC014377, RPRC005906, RPRC013292, RPRC009003, RPRC011142, RPRC014184, RPRC008979, RPRC001144, RPRC001275, RPRC008122, RPRC001582, RPRC003793, RPRC011694, RPRC015352, RPRC009331, RPRC006970, RPRC007358, RPRC008372, RPRC001448, RPRC003416, RPRC000800, RPRC012714, RPRC003842, RPRC014240, RPRC006334, RPRC002143, RPRC005936, RPRC005351, RPRC003442, RPRC005505, RPRC013790, RPRC000348, RPRC004368, RPRC008854, RPRC013366, RPRC000816, RPRC015292, RPRC008666, RPRC002028, RPRC011274, RPRC012204, RPRC007250
GO:0046933	proton-transporting ATP synthase activity, rotational mechanism	F	1.18E+03	1.12E+01	4	2	over	RPRC012073, RPRC011931, RPRC008498, RPRC005917	RPRC003410, RPRC005494
GO:0070062	extracellular exosome	C	2.39E+03	2.44E+01	5	8	over	RPRC008809, RPRC007515, RPRC001811, RPRC005383, RPRC012247	RPRC015027, RPRC012892, RPRC007468, RPRC009243, RPRC011182, RPRC006358, RPRC000881, RPRC000267
GO:0019843	rRNA binding	F	4.40E+03	5.00E+01	4	4	over	RPRC012633. RPRC012542. RPRC013371. RPRC011596	RPRC006783. RPRC014793. RPRC008479. RPRC011272
GO:0005549	odorant binding	F	1.14E+04	1.43E+02	4	6	over	RPRC000174. RPRC009905. RPRC010284. RPRC004407	RPRC000274. RPRC000476. RPRC010357. RPRC000378. RPRC014985. RPRC004408
GO:0051258	protein polymerization	P	2.22E+01	3.17E+02	5	16	over	RPRC003672. RPRC005383. RPRC010283. RPRC007667. RPRC004762	RPRC014380. RPRC011530. RPRC014377. RPRC011608. RPRC011142. RPRC013158. RPRC005737. RPRC005939. RPRC011694. RPRC011753. RPRC008372. RPRC004637. RPRC003842. RPRC003459. RPRC015390. RPRC000816

GO:0005525	GTP binding	F	3.17E+04	4.79E+02	12	118	over	RPRC012142. RPRC015257. RPRC003672. RPRC001811. RPRC005383. RPRC010283. RPRC007667. RPRC015041. RPRC014951. RPRC011075. RPRC012140. RPRC004762	RPRC014380. RPRC011062. RPRC015453. RPRC013503. RPRC015134. RPRC005498. RPRC008142. RPRC004755. RPRC005852. RPRC013261. RPRC007642. RPRC005628. RPRC011076. RPRC005457. RPRC003955. RPRC010209. RPRC014377. RPRC010918. RPRC003772. RPRC006830. RPRC013168. RPRC005906. RPRC013292. RPRC009003. RPRC008853. RPRC012492. RPRC015391. RPRC013377. RPRC007644. RPRC010913. RPRC010340. RPRC011142. RPRC003248. RPRC003065. RPRC004007. RPRC007066. RPRC005518. RPRC014184. RPRC008979. RPRC001144. RPRC008553. RPRC010076. RPRC001275. RPRC014716. RPRC008122. RPRC001582. RPRC003793. RPRC011694. RPRC005832. RPRC011936. RPRC015352. RPRC009331. RPRC006970. RPRC014014. RPRC009243. RPRC007358. RPRC008372. RPRC001448. RPRC000800. RPRC012714. RPRC010563. RPRC003842. RPRC015449. RPRC009576. RPRC014240. RPRC008314. RPRC007034. RPRC005889. RPRC006334. RPRC002143. RPRC007838. RPRC013286. RPRC007643. RPRC007802. RPRC004106. RPRC014052. RPRC004254. RPRC005936. RPRC005351. RPRC012940. RPRC006938. RPRC009742. RPRC011124. RPRC003442. RPRC002735. RPRC005505. RPRC013790. RPRC015125. RPRC005482. RPRC005553. RPRC000348. RPRC004368. RPRC006886. RPRC008854. RPRC003337. RPRC009223. RPRC015392. RPRC013366. RPRC000816. RPRC011864. RPRC013915. RPRC015292. RPRC004710. RPRC004646. RPRC008666. RPRC014910. RPRC008081. RPRC014929. RPRC002028. RPRC013508. RPRC011274. RPRC006184. RPRC004629. RPRC012204. RPRC000267. RPRC003970. RPRC003344. RPRC007250
GO:0022625	cytosolic large ribosomal subunit	C	5.45E+04	8.98E+02	2	0	over	RPRC014620. RPRC015127	
GO:0005874	microtubule	C	1.04E+04	1.84E+03	6	38	over	RPRC003672. RPRC007515. RPRC005383. RPRC010283. RPRC007667. RPRC004762	RPRC014380. RPRC004943. RPRC011176. RPRC014377. RPRC010393. RPRC013227. RPRC002789. RPRC004988. RPRC011142. RPRC010423. RPRC014833. RPRC002641. RPRC010968. RPRC005737. RPRC001777. RPRC006473. RPRC011694. RPRC003935. RPRC004459. RPRC008372. RPRC012400. RPRC005456. RPRC002928. RPRC011282. RPRC003842. RPRC011177. RPRC015106. RPRC009316. RPRC005789. RPRC001728. RPRC005417. RPRC015439. RPRC010046. RPRC000816. RPRC004870. RPRC012381. RPRC013987. RPRC001778
GO:0003746	translation elongation factor activity	F	1.18E+05	2.14E+03	4	15	over	RPRC012142. RPRC007684. RPRC015041. RPRC012140	RPRC012040. RPRC012111. RPRC013273. RPRC011076. RPRC009039. RPRC003914. RPRC007456. RPRC001582. RPRC005473. RPRC011027. RPRC002143. RPRC005936. RPRC002752. RPRC005351. RPRC011916
GO:0006448	regulation of translational elongation	P	2.24E+05	5.22E+03	4	20	over	RPRC012142. RPRC007684. RPRC015041. RPRC012140	RPRC012040. RPRC014027. RPRC012111. RPRC013273. RPRC011076. RPRC009039. RPRC003914. RPRC015450. RPRC007456. RPRC001582. RPRC005473. RPRC011027. RPRC002143. RPRC005280. RPRC005936. RPRC002752. RPRC005351. RPRC011916. RPRC012971. RPRC008081

GO:0008233	peptidase activity	F	5.36E+05	1.46E+04	1	219	under	RPRC006290	RPRC009154. RPRC003097. RPRC003320. RPRC015015. RPRC009301. RPRC014463. RPRC014770. RPRC006557. RPRC010196. RPRC003168. RPRC001252. RPRC007074. RPRC011388. RPRC008278. RPRC013356. RPRC001240. RPRC012649. RPRC007157. RPRC012785. RPRC013734. RPRC012868. RPRC008963. RPRC012613. RPRC012920. RPRC003351. RPRC005414. RPRC015289. RPRC001944. RPRC010398. RPRC009977. RPRC002249. RPRC012513. RPRC001697. RPRC000913. RPRC013717. RPRC011176. RPRC000890. RPRC010309. RPRC000973. RPRC012508. RPRC009789. RPRC014294. RPRC008515. RPRC008037. RPRC013001. RPRC008587. RPRC013665. RPRC005262. RPRC006216. RPRC009127. RPRC010640. RPRC008989. RPRC004798. RPRC015124. RPRC010795. RPRC005321. RPRC009800. RPRC005067. RPRC006028. RPRC014950. RPRC012970. RPRC014407. RPRC002948. RPRC011018. RPRC006554. RPRC009294. RPRC008510. RPRC003153. RPRC008753. RPRC012487. RPRC007632. RPRC011066. RPRC013584. RPRC007692. RPRC013731. RPRC002919. RPRC003360. RPRC014196. RPRC014184. RPRC008099. RPRC002884. RPRC002310. RPRC014012. RPRC008063. RPRC011427. RPRC014338. RPRC010708. RPRC006644. RPRC000644. RPRC002696. RPRC000905. RPRC002377. RPRC015393. RPRC008831. RPRC008448. RPRC003155. RPRC008034. RPRC003864. RPRC012075. RPRC007220. RPRC000308. RPRC008253. RPRC002519. RPRC012465. RPRC000717. RPRC008082. RPRC014262. RPRC001796. RPRC007936. RPRC006929. RPRC001700. RPRC015288. RPRC002472. RPRC006917. RPRC003357. RPRC002798. RPRC003102. RPRC011748. RPRC010532. RPRC015188. RPRC011322. RPRC015323. RPRC007429. RPRC009226. RPRC001679. RPRC006558. RPRC010834. RPRC013528. RPRC008598. RPRC014045. RPRC009433. RPRC012786. RPRC008733. RPRC011672. RPRC006605. RPRC006753. RPRC010965. RPRC012212. RPRC005829. RPRC011129. RPRC006092. RPRC001372. RPRC002326. RPRC014897. RPRC006907. RPRC010280. RPRC005463. RPRC013126. RPRC004585. RPRC000405. RPRC013990. RPRC003247. RPRC002593. RPRC000886. RPRC003478. RPRC015385. RPRC000294. RPRC007479. RPRC011762. RPRC009595. RPRC004620. RPRC011379. RPRC013495. RPRC003708. RPRC009021. RPRC012374. RPRC000205. RPRC013009. RPRC012350. RPRC005322. RPRC007219. RPRC003608. RPRC001374. RPRC005405. RPRC009004. RPRC006898. RPRC014325. RPRC003668. RPRC004113. RPRC008281. RPRC009383. RPRC015415. RPRC007881. RPRC003230. RPRC010205. RPRC006957. RPRC000816. RPRC014468. RPRC003154. RPRC001652. RPRC003556. RPRC001747. RPRC003715. RPRC013661. RPRC007007. RPRC014699. RPRC013490. RPRC005596. RPRC005607. RPRC011776. RPRC003946. RPRC013732. RPRC015299. RPRC013349. RPRC011752. RPRC000219. RPRC014822. RPRC011433. RPRC004789. RPRC014810. RPRC009668. RPRC014173. RPRC010407. RPRC009349. RPRC009720. RPRC006893. RPRC014013. RPRC003090. RPRC000107

GO:0000910	cytokinesis	P	5.44E+05	2.23E+04	2	6	over	RPRC007515. RPRC009875	RPRC006338. RPRC010698. RPRC003092. RPRC000800. RPRC007538. RPRC009554
GO:0008061	chitin binding	F	5.44E+05	2.70E+04	3	19	over	RPRC000381. RPRC013059. RPRC000042	RPRC010255. RPRC004089. RPRC003005. RPRC005523. RPRC014196. RPRC008641. RPRC008796. RPRC014976. RPRC002707. RPRC009212. RPRC010254. RPRC000663. RPRC013840. RPRC007943. RPRC012296. RPRC001762. RPRC001569. RPRC010253. RPRC014929
GO:0005125	cytokine activity	F	5.44E+05	3.01E+03	1	0	over	RPRC002698	
GO:0006892	post-Golgi vesicle-mediated transport	P	5.44E+05	3.01E+03	1	0	over	RPRC014951	
GO:0000275	mitochondrial proton-transporting ATP synthase complex. catalytic core F(1)	C	5.44E+05	3.01E+03	1	0	over	RPRC012073	
GO:0035690	cellular response to drug	P	5.44E+05	3.01E+03	1	0	over	RPRC007515	
GO:0043274	phospholipase binding	F	5.44E+05	3.01E+03	1	0	over	RPRC007515	
GO:0001837	epithelial to mesenchymal transition	P	5.44E+05	3.01E+03	1	0	over	RPRC007515	
GO:0055117	regulation of cardiac muscle contraction	P	5.44E+05	3.01E+03	1	0	over	RPRC007515	
GO:0000028	ribosomal small subunit assembly	P	5.44E+05	3.01E+03	1	0	over	RPRC000499	
GO:0060079	regulation of excitatory postsynaptic membrane potential	P	5.44E+05	3.01E+03	1	0	over	RPRC007515	
GO:0003958	NADPH-hemoprotein reductase activity	F	5.44E+05	3.01E+03	1	0	over	RPRC005729	
GO:0015886	heme transport	P	5.44E+05	3.01E+03	1	0	over	RPRC009283	
GO:0043024	ribosomal small subunit binding	F	5.44E+05	3.01E+03	1	0	over	RPRC001811	
GO:0010992	ubiquitin homeostasis	P	5.44E+05	3.01E+03	1	0	over	RPRC012247	
GO:0033192	calmodulin-dependent protein phosphatase activity	F	5.44E+05	3.01E+03	1	0	over	RPRC007515	
GO:0006407	rRNA export from nucleus	P	5.44E+05	3.01E+03	1	0	over	RPRC010146	
GO:0033173	calcineurin-NFAT signaling cascade	P	5.44E+05	3.01E+03	1	0	over	RPRC007515	
GO:0035307	positive regulation of protein dephosphorylation	P	5.44E+05	3.01E+03	1	0	over	RPRC007515	
GO:0001569	patterning of blood vessels	P	5.44E+05	3.01E+03	1	0	over	RPRC007515	
GO:0010880	regulation of release of sequestered calcium ion into cytosol by	P	5.44E+05	3.01E+03	1	0	over	RPRC007515	

	sarcoplasmic reticulum							
GO:0071333	cellular response to glucose stimulus	P	5.44E+05	3.01E+03	1	0	over	RPRC007515
GO:0008537	proteasome activator complex	C	5.44E+05	3.01E+03	1	0	over	RPRC009319
GO:0006336	DNA replication-independent nucleosome assembly	P	5.44E+05	3.01E+03	1	0	over	RPRC008809
GO:0042383	sarcolemma	C	5.44E+05	3.01E+03	1	0	over	RPRC007515
GO:0032587	ruffle membrane	C	5.44E+05	3.01E+03	1	0	over	RPRC001811
GO:0032516	positive regulation of phosphoprotein phosphatase activity	P	5.44E+05	3.01E+03	1	0	over	RPRC007515
GO:0046676	negative regulation of insulin secretion	P	5.44E+05	3.01E+03	1	0	over	RPRC007515
GO:0031997	N-terminal myristoylation domain binding	F	5.44E+05	3.01E+03	1	0	over	RPRC007515
GO:0031996	thioesterase binding	F	5.44E+05	3.01E+03	1	0	over	RPRC007515
GO:0031932	TORC2 complex	C	5.44E+05	3.01E+03	1	0	over	RPRC012706
GO:0031929	TOR signaling	P	5.44E+05	3.01E+03	1	0	over	RPRC012706
GO:0015439	heme-transporting ATPase activity	F	5.44E+05	3.01E+03	1	0	over	RPRC009283
GO:0048741	skeletal muscle fiber development	P	5.44E+05	3.01E+03	1	0	over	RPRC007515
GO:0000788	nuclear nucleosome	C	5.44E+05	3.01E+03	1	0	over	RPRC008809
GO:0005513	detection of calcium ion	P	5.44E+05	3.01E+03	1	0	over	RPRC007515
GO:0014883	transition between fast and slow fiber	P	5.44E+05	3.01E+03	1	0	over	RPRC007515
GO:0045955	negative regulation of calcium ion-dependent exocytosis	P	5.44E+05	3.01E+03	1	0	over	RPRC007515
GO:0051343	positive regulation of cyclic-nucleotide phosphodiesterase activity	P	5.44E+05	3.01E+03	1	0	over	RPRC007515
GO:0009882	blue light photoreceptor activity	F	5.44E+05	3.01E+03	1	0	over	RPRC005966
GO:0051298	centrosome duplication	P	5.44E+05	3.01E+03	1	0	over	RPRC015127
GO:1902036	regulation of hematopoietic stem cell differentiation	P	5.44E+05	3.01E+03	1	0	over	RPRC014951
GO:0009785	blue light signaling pathway	P	5.44E+05	3.01E+03	1	0	over	RPRC005966
GO:0072542	protein phosphatase	F	5.44E+05	3.01E+03	1	0	over	RPRC007515

	activator activity							
GO:0007595	lactation	P	5.44E+05	3.01E+03	1	0	over	RPRC001811
GO:0004838	L-tyrosine:2-oxoglutarate aminotransferase activity	F	5.44E+05	3.01E+03	1	0	over	RPRC000370
GO:0021762	substantia nigra development	P	5.44E+05	3.01E+03	1	0	over	RPRC007515
GO:0060487	lung epithelial cell differentiation	P	5.44E+05	3.01E+03	1	0	over	RPRC007515
GO:0050679	positive regulation of epithelial cell proliferation	P	5.44E+05	3.01E+03	1	0	over	RPRC001811
GO:0071949	FAD binding	F	5.44E+05	3.01E+03	1	0	over	RPRC005966
GO:0031492	nucleosomal DNA binding	F	5.44E+05	3.01E+03	1	0	over	RPRC008809
GO:0031432	titin binding	F	5.44E+05	3.01E+03	1	0	over	RPRC007515
GO:0060316	positive regulation of ryanodine-sensitive calcium-release channel activity	P	5.44E+05	3.01E+03	1	0	over	RPRC007515

GO:0006468	protein phosphorylation	P	5.78E+05	3.24E+04	1	203	under	RPRC000798	RPRC000167. RPRC007014. RPRC001021. RPRC014610. RPRC014789. RPRC001666. RPRC015063. RPRC002513. RPRC014924. RPRC001790. RPRC004860. RPRC003853. RPRC006286. RPRC012318. RPRC005491. RPRC003777. RPRC012932. RPRC010391. RPRC007535. RPRC004772. RPRC008485. RPRC012856. RPRC001637. RPRC002018. RPRC013487. RPRC005633. RPRC000333. RPRC004767. RPRC013865. RPRC002851. RPRC000321. RPRC009634. RPRC006659. RPRC008054. RPRC011471. RPRC008213. RPRC007813. RPRC005852. RPRC000961. RPRC001330. RPRC013013. RPRC004886. RPRC011932. RPRC012556. RPRC003051. RPRC011920. RPRC001792. RPRC008734. RPRC005019. RPRC012934. RPRC004319. RPRC015112. RPRC007939. RPRC001692. RPRC008168. RPRC004857. RPRC015100. RPRC012994. RPRC001149. RPRC007035. RPRC007915. RPRC007732. RPRC000839. RPRC009931. RPRC004881. RPRC003862. RPRC013175. RPRC011946. RPRC015450. RPRC006442. RPRC000863. RPRC014526. RPRC008334. RPRC013655. RPRC011910. RPRC013400. RPRC010713. RPRC011822. RPRC011917. RPRC001610. RPRC007432. RPRC010785. RPRC006668. RPRC008636. RPRC008784. RPRC006390. RPRC015014. RPRC002282. RPRC001741. RPRC012099. RPRC013105. RPRC012489. RPRC002914. RPRC014191. RPRC014923. RPRC009431. RPRC000699. RPRC007546. RPRC014676. RPRC009184. RPRC008241. RPRC005266. RPRC008336. RPRC005349. RPRC006894. RPRC011919. RPRC014321. RPRC002957. RPRC004754. RPRC007812. RPRC012412. RPRC011796. RPRC013036. RPRC006941. RPRC000736. RPRC004483. RPRC005095. RPRC012077. RPRC012957. RPRC002438. RPRC001555. RPRC007441. RPRC001141. RPRC012200. RPRC007991. RPRC009026. RPRC006251. RPRC003276. RPRC011265. RPRC009002. RPRC006429. RPRC006577. RPRC001750. RPRC013126. RPRC006860. RPRC002309. RPRC013778. RPRC014223. RPRC007336. RPRC000814. RPRC010180. RPRC012179. RPRC007631. RPRC006548. RPRC004230. RPRC006377. RPRC000862. RPRC002038. RPRC013495. RPRC003442. RPRC001940. RPRC010394. RPRC007591. RPRC005800. RPRC008233. RPRC001693. RPRC011502. RPRC010636. RPRC001226. RPRC007981. RPRC003744. RPRC015332. RPRC002559. RPRC010134. RPRC001286. RPRC004208. RPRC008854. RPRC007119. RPRC005317. RPRC001031. RPRC012588. RPRC010991. RPRC014715. RPRC015085. RPRC004344. RPRC001740. RPRC000881. RPRC013601. RPRC014787. RPRC001102. RPRC003727. RPRC004097. RPRC015156. RPRC006467. RPRC011545. RPRC009939. RPRC000864. RPRC007788. RPRC006124. RPRC009342. RPRC013497. RPRC004717. RPRC013140. RPRC011911. RPRC006207. RPRC004705. RPRC001221. RPRC003273. RPRC000184-PB. RPRC000184. RPRC011516. RPRC008406. RPRC006669
GO:0046961	proton-transporting ATPase activity. rotational mechanism	F	6.04E+05	3.45E+04	2	8	over	RPRC012073. RPRC008498	RPRC003694. RPRC003410. RPRC002938. RPRC008833. RPRC007341. RPRC005494. RPRC009992. RPRC003113

GO:0006351	transcription. DNA-templated	P	6.08E+05	3.49E+04	3	299	under	RPRC007515. RPRC001993. RPRC000303	RPRC001204. RPRC001595. RPRC015417. RPRC003870. RPRC004092. RPRC009544. RPRC013615. RPRC009946. RPRC015246. RPRC013846. RPRC003475. RPRC014120. RPRC011695. RPRC000954. RPRC012076. RPRC011535. RPRC010976. RPRC005757. RPRC009929. RPRC007388. RPRC015365. RPRC012040. RPRC005005. RPRC007842. RPRC008887. RPRC001701. RPRC001318. RPRC010462. RPRC005391. RPRC001625. RPRC014334. RPRC005645. RPRC012111. RPRC000026. RPRC002066. RPRC003748. RPRC007347. RPRC006647. RPRC005947. RPRC011554. RPRC001915. RPRC007801. RPRC003819. RPRC010274. RPRC003712. RPRC000737. RPRC005100. RPRC002527. RPRC000133. RPRC003051. RPRC001899. RPRC011283. RPRC006459. RPRC008085. RPRC002817. RPRC003110. RPRC000583-PB. RPRC000583. RPRC006116. RPRC000264. RPRC002475. RPRC009163. RPRC001680. RPRC002941. RPRC002220. RPRC010293. RPRC006801. RPRC010281. RPRC007266. RPRC015255. RPRC009234. RPRC001910. RPRC011792. RPRC009636. RPRC011887. RPRC003791. RPRC003886. RPRC008192. RPRC001515. RPRC006478. RPRC012392. RPRC012403. RPRC015314. RPRC000951. RPRC009536. RPRC004402. RPRC014602. RPRC010188. RPRC006844. RPRC009755. RPRC015055. RPRC005264. RPRC002505. RPRC001794. RPRC007468. RPRC014514. RPRC014662. RPRC010885. RPRC005973. RPRC002577. RPRC007456. RPRC006183. RPRC008087. RPRC008489. RPRC000437. RPRC015114. RPRC008075. RPRC009962. RPRC008636. RPRC015186. RPRC010625. RPRC011161. RPRC007344. RPRC004528. RPRC005785. RPRC007025. RPRC004262. RPRC007173. RPRC000230. RPRC001180. RPRC001127. RPRC001996. RPRC005932. RPRC012907. RPRC007256. RPRC012429. RPRC003072. RPRC005920. RPRC007244. RPRC002110. RPRC004883. RPRC012003. RPRC007327. RPRC005673. RPRC012796. RPRC005188. RPRC001429. RPRC005513. RPRC012700. RPRC005176. RPRC003374. RPRC003681. RPRC007546. RPRC001967. RPRC003362. RPRC000853. RPRC009438. RPRC007458. RPRC010875. RPRC000936. RPRC012843. RPRC001317. RPRC014174. RPRC011665. RPRC007032. RPRC000587. RPRC006332. RPRC000025. RPRC003823. RPRC011027. RPRC000900. RPRC006499. RPRC001122. RPRC004754. RPRC003811. RPRC001058. RPRC001938. RPRC007824. RPRC013788. RPRC008212. RPRC007346. RPRC002774. RPRC000824. RPRC001205. RPRC008698. RPRC007334. RPRC015259. RPRC003002. RPRC010273. RPRC002750. RPRC007565. RPRC002845. RPRC009521. RPRC000239. RPRC009923. RPRC013000. RPRC013664. RPRC010332. RPRC001336. RPRC005261. RPRC002414. RPRC003216. RPRC006848. RPRC008243. RPRC011448. RPRC013416. RPRC005006. RPRC001236. RPRC004868. RPRC011914. RPRC012136. RPRC009026. RPRC002486. RPRC014104. RPRC007938. RPRC008019. RPRC010782. RPRC006092. RPRC002781. RPRC000767. RPRC009416. RPRC011667. RPRC003985. RPRC005805. RPRC001614. RPRC015106. RPRC002693. RPRC010841. RPRC011791. RPRC012172. RPRC006151. RPRC006405. RPRC010617. RPRC003790.

									RPRC003801. RPRC001598. RPRC011714. RPRC012964. RPRC009706. RPRC008357. RPRC010410. RPRC009766. RPRC009045. RPRC011455. RPRC000282. RPRC007691. RPRC004875. RPRC008747. RPRC015149. RPRC006660. RPRC009188. RPRC003111. RPRC011916. RPRC014254. RPRC013311. RPRC001852. RPRC001788. RPRC004922. RPRC006094. RPRC007981. RPRC012581. RPRC000496. RPRC002559. RPRC015268. RPRC007586. RPRC014154. RPRC007267. RPRC001031. RPRC012906. RPRC009064. RPRC000881. RPRC007000. RPRC010289. RPRC001186. RPRC005115. RPRC003461. RPRC015156. RPRC002606. RPRC010679. RPRC007940. RPRC009271. RPRC003703. RPRC004953. RPRC000455. RPRC012157. RPRC004327. RPRC014273. RPRC013330. RPRC006379. RPRC008411. RPRC001871. RPRC004156. RPRC005003. RPRC008737. RPRC002164. RPRC008152. RPRC005484. RPRC013325. RPRC006502. RPRC002968. RPRC011971. RPRC004936. RPRC007274. RPRC011345. RPRC015422. RPRC013620. RPRC004192. RPRC004351. RPRC014392
GO:0006030	chitin metabolic process	P	6.46E+05	3.77E+04	3	22	over	RPRC000381. RPRC013059. RPRC000042	RPRC010255. RPRC015300. RPRC004089. RPRC003005. RPRC005523. RPRC014196. RPRC008641. RPRC008796. RPRC014976. RPRC002707. RPRC009212. RPRC010254. RPRC011446. RPRC011263. RPRC000663. RPRC013840. RPRC007943. RPRC012296. RPRC001762. RPRC001569. RPRC010253. RPRC014929
GO:0033177	proton-transporting two-sector ATPase complex. proton-transporting domain	C	6.99E+05	4.13E+03	2	9	over	RPRC006543. RPRC010550	RPRC013834. RPRC005881. RPRC008403. RPRC015179. RPRC001199. RPRC008312. RPRC005989. RPRC007426. RPRC001198
GO:0019904	protein domain specific binding	F	6.99E+05	4.13E+03	2	9	over	RPRC007515. RPRC011742	RPRC011237. RPRC011822. RPRC008500. RPRC003362. RPRC008219. RPRC001496. RPRC010679. RPRC013325. RPRC012128

GO:0019752	carboxylic acid metabolic process	P	7.19E+05	4.29E+04	5	385	under	RPRC012101. RPRC000370. RPRC000798. RPRC013371. RPRC012369	RPRC015027. RPRC000509. RPRC001671. RPRC013946. RPRC012423. RPRC007493. RPRC006550. RPRC000391. RPRC002708. RPRC000167. RPRC003658. RPRC001276. RPRC000633. RPRC003403. RPRC001021. RPRC015405. RPRC001985. RPRC014789. RPRC009934. RPRC014948. RPRC001666. RPRC003634. RPRC010184. RPRC015063. RPRC002756. RPRC007783. RPRC004819. RPRC011281. RPRC014924. RPRC001790. RPRC013492. RPRC007559. RPRC003853. RPRC014180. RPRC006286. RPRC012318. RPRC005491. RPRC005070. RPRC012932. RPRC010391. RPRC011204. RPRC008242. RPRC000706. RPRC007535. RPRC004772. RPRC008485. RPRC003924. RPRC012856. RPRC009810. RPRC013965. RPRC013487. RPRC001371. RPRC007045. RPRC012271. RPRC011985. RPRC014027. RPRC007511. RPRC000830. RPRC011093. RPRC006747. RPRC004365. RPRC006481. RPRC001708. RPRC004619. RPRC000333. RPRC004767. RPRC000269. RPRC002851. RPRC000321. RPRC004755. RPRC009634. RPRC006659. RPRC013853. RPRC008054. RPRC011471. RPRC014329. RPRC007654. RPRC002213. RPRC007813. RPRC005852. RPRC007961. RPRC000961. RPRC003819. RPRC017364. RPRC002846. RPRC001330. RPRC001266. RPRC004898. RPRC009777. RPRC009522. RPRC006547. RPRC002420. RPRC010918. RPRC011932. RPRC003317. RPRC012556. RPRC011040. RPRC014110. RPRC003294. RPRC004312. RPRC013824. RPRC004460. RPRC005262. RPRC006216. RPRC001166. RPRC008734. RPRC003270. RPRC009972. RPRC006997. RPRC012297. RPRC004319. RPRC013417. RPRC007939. RPRC015196. RPRC000678. RPRC004709. RPRC004857. RPRC004538. RPRC007035. RPRC007915. RPRC001042. RPRC011497. RPRC011963. RPRC005878. RPRC003507. RPRC007732. RPRC003241. RPRC011951. RPRC005281. RPRC000720. RPRC004881. RPRC014626. RPRC003862. RPRC005830. RPRC013175. RPRC003384. RPRC015450. RPRC007958. RPRC004569. RPRC000863. RPRC014526. RPRC011858. RPRC000123. RPRC015309. RPRC007468. RPRC000366. RPRC012853. RPRC013655. RPRC007934. RPRC007213. RPRC013484. RPRC009424. RPRC010713. RPRC015369. RPRC011822. RPRC006023. RPRC014669. RPRC010383. RPRC000751. RPRC008784. RPRC010466. RPRC003650. RPRC006561. RPRC001056. RPRC013543. RPRC012677. RPRC011161. RPRC006390. RPRC000758. RPRC000810. RPRC002282. RPRC008684. RPRC012027. RPRC008270. RPRC005454. RPRC000734. RPRC005920. RPRC009774. RPRC015074. RPRC009709. RPRC006225. RPRC000805. RPRC002914. RPRC011132. RPRC014191. RPRC014923. RPRC002838. RPRC007546. RPRC007705. RPRC014676. RPRC001246. RPRC013089. RPRC012146. RPRC009184. RPRC008241. RPRC005266. RPRC005880. RPRC002909. RPRC011848. RPRC007936. RPRC008472. RPRC010727. RPRC001370. RPRC012270. RPRC001317. RPRC010142. RPRC011103. RPRC006894. RPRC009816. RPRC014321. RPRC002957. RPRC004447. RPRC000741. RPRC011736. RPRC012986. RPRC000001. RPRC011470. RPRC012572. RPRC007812. RPRC005059. RPRC008762. RPRC008698. RPRC007907. RPRC013935.

									RPRC002762. RPRC015259. RPRC013036. RPRC007884. RPRC006941. RPRC006156. RPRC011234. RPRC004961. RPRC004483. RPRC003878. RPRC001496. RPRC004566. RPRC012077. RPRC006056. RPRC005261. RPRC012957. RPRC002438. RPRC000700. RPRC009433. RPRC006670. RPRC012065. RPRC008881. RPRC000617. RPRC010965. RPRC000605. RPRC009250. RPRC000689. RPRC007441. RPRC004466. RPRC001714. RPRC008402. RPRC009588. RPRC012200. RPRC001691. RPRC013393. RPRC007672. RPRC009014. RPRC003276. RPRC014028. RPRC011265. RPRC006919. RPRC011519. RPRC014790. RPRC007034. RPRC009002. RPRC000589. RPRC009404. RPRC006429. RPRC003666. RPRC012609. RPRC006015. RPRC015095. RPRC011336. RPRC005315. RPRC002704. RPRC007572. RPRC002067. RPRC008628. RPRC013126. RPRC004278. RPRC006310. RPRC000826. RPRC014478. RPRC015071. RPRC005280. RPRC014223. RPRC007336. RPRC013364. RPRC002286. RPRC005617. RPRC004644. RPRC006548. RPRC010103. RPRC004230. RPRC001255. RPRC006377. RPRC013423. RPRC000862. RPRC002038. RPRC003932. RPRC004556. RPRC009021. RPRC003283. RPRC001940. RPRC013009. RPRC010394. RPRC015125. RPRC015273. RPRC007591. RPRC005641. RPRC005800. RPRC005884. RPRC003927. RPRC008233. RPRC004858. RPRC005310. RPRC005553. RPRC001055. RPRC003744. RPRC013785. RPRC014739. RPRC015332. RPRC014473. RPRC013318. RPRC000757. RPRC013466. RPRC001202. RPRC001286. RPRC012588. RPRC002281. RPRC010991. RPRC008535. RPRC014715. RPRC012174. RPRC004344. RPRC001740. RPRC000881. RPRC013601. RPRC014787. RPRC015232. RPRC014946. RPRC007402. RPRC001102. RPRC003727. RPRC004097. RPRC004817. RPRC006053. RPRC000467. RPRC015292. RPRC009939. RPRC000698. RPRC013987. RPRC009927. RPRC000864. RPRC011764. RPRC008081. RPRC001488. RPRC009342. RPRC008483. RPRC011604. RPRC005003. RPRC013497. RPRC013140. RPRC005826. RPRC014101. RPRC006207. RPRC000840. RPRC002885. RPRC012364. RPRC006491. RPRC000184-PB. RPRC000184. RPRC008406. RPRC000923. RPRC013461. RPRC004924. RPRC006160. RPRC000574. RPRC004605. RPRC012678. RPRC010212
GO:0016209	antioxidant activity	F	7.58E+05	4.60E+04	3	24	over	RPRC002923. RPRC015388. RPRC006756	RPRC009934. RPRC007595. RPRC007347. RPRC014110. RPRC012922. RPRC007468. RPRC007213. RPRC000982. RPRC011108. RPRC007344. RPRC009148. RPRC004883. RPRC005064. RPRC007907. RPRC011796. RPRC015195. RPRC011500. RPRC000422-PB. RPRC015401. RPRC007484. RPRC015008. RPRC004965. RPRC004717. RPRC011126
GO:0004672	protein kinase activity	F	7.88E+05	4.79E+04	2	232	under	RPRC000798. RPRC013371	RPRC000167. RPRC007014. RPRC001021. RPRC014610. RPRC014789. RPRC001666. RPRC015063. RPRC002513. RPRC007221. RPRC014924. RPRC013917. RPRC001790. RPRC003853. RPRC004234. RPRC006286. RPRC012318. RPRC005491. RPRC003777. RPRC012932. RPRC010391. RPRC011204. RPRC007535. RPRC004772. RPRC008485.

RPRC012856. RPRC009810. RPRC001637. RPRC002018.
RPRC013487. RPRC008863. RPRC005633. RPRC000333.
RPRC004767. RPRC000576. RPRC008787. RPRC013865.
RPRC002851. RPRC000321. RPRC009634. RPRC006659.
RPRC003907. RPRC008054. RPRC011471. RPRC008213.
RPRC000404. RPRC007813. RPRC005852. RPRC000961.
RPRC001330. RPRC013013. RPRC004886. RPRC002420.
RPRC011932. RPRC011211. RPRC012556. RPRC003051.
RPRC005528. RPRC011920. RPRC001792. RPRC008734.
RPRC005019. RPRC012934. RPRC004319. RPRC008551.
RPRC015112. RPRC007939. RPRC001692. RPRC004857.
RPRC012994. RPRC004538. RPRC001149. RPRC007035.
RPRC007915. RPRC013044. RPRC007732. RPRC000839.
RPRC009931. RPRC004881. RPRC000803. RPRC003862.
RPRC013175. RPRC014709. RPRC006868. RPRC011946.
RPRC000625. RPRC015450. RPRC002358. RPRC006442.
RPRC000863. RPRC014526. RPRC008334. RPRC013655.
RPRC011910. RPRC010713. RPRC011822. RPRC011917.
RPRC001610. RPRC010542. RPRC007432. RPRC010785.
RPRC006668. RPRC008784. RPRC010466. RPRC006390.
RPRC005057. RPRC012505. RPRC002282. RPRC008684.
RPRC001741. RPRC012099. RPRC013105. RPRC014214.
RPRC012489. RPRC002914. RPRC014191. RPRC014923.
RPRC009431. RPRC013438. RPRC000699. RPRC007546.
RPRC008560. RPRC014676. RPRC009184. RPRC008241.
RPRC005266. RPRC008336. RPRC009893. RPRC001329.
RPRC005349. RPRC006894. RPRC011919. RPRC014321.
RPRC002957. RPRC011736. RPRC007812. RPRC012412.
RPRC013036. RPRC006941. RPRC000736. RPRC004483.
RPRC005095. RPRC012077. RPRC003304. RPRC012957.
RPRC002438. RPRC002012. RPRC014583. RPRC002498.
RPRC001555. RPRC007441. RPRC008402. RPRC001141.
RPRC012200. RPRC007991. RPRC011277. RPRC006251.
RPRC003276. RPRC011265. RPRC001869. RPRC009002.
RPRC006429. RPRC001857. RPRC005941. RPRC001750.
RPRC008628. RPRC013126. RPRC006860. RPRC015071.
RPRC014223. RPRC007336. RPRC000814. RPRC010180.
RPRC012179. RPRC007631. RPRC006548. RPRC004230.
RPRC001255. RPRC013098. RPRC006377. RPRC000862.
RPRC002038. RPRC014525. RPRC014832. RPRC013495.
RPRC001940. RPRC010394. RPRC007591. RPRC005429.
RPRC005800. RPRC008233. RPRC001693. RPRC010636.
RPRC001226. RPRC003744. RPRC015332. RPRC010612.
RPRC001202. RPRC010134. RPRC001286. RPRC004208.
RPRC007119. RPRC012588. RPRC010991. RPRC014715.
RPRC004344. RPRC001740. RPRC000881. RPRC013601.
RPRC000248. RPRC014787. RPRC001102. RPRC003727.
RPRC004097. RPRC011545. RPRC000467. RPRC009939.
RPRC007079. RPRC000864. RPRC007788. RPRC001488.
RPRC009342. RPRC013497. RPRC013140. RPRC011911.
RPRC006207. RPRC015139. RPRC004705. RPRC003273.
RPRC000184-PB. RPRC000184. RPRC008406. RPRC006669

GO annotation terms for enriched transcripts in female library

GO-ID	Term	Category*	FDR	P-Value	N° of genes in dataset D.E.	N° of genes in *R. prolixus* genome	Over/Under represented	VectorBase code of genes dataset D.E.	VectorBase code of genes in *R. prolixus* genome
GO:0003735	structural constituent of ribosome	F	1.09E-66	1.85E-70	62	30	over	RPRC012633, RPRC010853, RPRC003327, RPRC009716, RPRC012784, RPRC004269, RPRC013146, RPRC002520, RPRC007451, RPRC002698, RPRC010146, RPRC003768, RPRC014587, RPRC009189, RPRC013169, RPRC007328, RPRC013077, RPRC007856, RPRC012158, RPRC010378, RPRC012542, RPRC005701, RPRC010286, RPRC014419, RPRC003510, RPRC005980, RPRC014363, RPRC005724, RPRC012711, RPRC002544, RPRC006329, RPRC005430, RPRC002470, RPRC013786, RPRC009702, RPRC013851, RPRC011260, RPRC006974, RPRC011456, RPRC014620, RPRC013371, RPRC014845, RPRC011937, RPRC009337, RPRC012162, RPRC012353, RPRC012387, RPRC011596, RPRC005389, RPRC013504, RPRC013167, RPRC002923, RPRC015127, RPRC015001, RPRC010405, RPRC007825, RPRC010192, RPRC000499, RPRC012354, RPRC006392, RPRC007230, RPRC006309	RPRC008799, RPRC014365, RPRC002822, RPRC009039, RPRC004296, RPRC011999, RPRC015409, RPRC012941, RPRC002505, RPRC012363, RPRC000922, RPRC006118, RPRC014793, RPRC006758, RPRC008479, RPRC010054, RPRC000972, RPRC003014, RPRC002869, RPRC005011, RPRC009871, RPRC012072, RPRC006051, RPRC010872, RPRC003980, RPRC009873, RPRC002897, RPRC004303, RPRC001328, RPRC010809
GO:0022627	cytosolic small ribosomal subunit	C	1.22E+02	8.48E-01	4	0	over	RPRC010146, RPRC013504, RPRC002923, RPRC000499	
GO:0005200	structural constituent of cytoskeleton	F	4.20E+02	2.99E-01	6	9	over	RPRC003672, RPRC009875, RPRC005383, RPRC010283, RPRC007667, RPRC004762	RPRC014380, RPRC014377, RPRC005328, RPRC011694, RPRC008372, RPRC008343, RPRC003842, RPRC015390, RPRC000816
GO:0070062	extracellular exosome	C	2.86E+03	2.67E+01	5	8	over	RPRC008809, RPRC007515, RPRC001811, RPRC005383, RPRC012247	RPRC015027, RPRC012892, RPRC007468, RPRC009243, RPRC011182, RPRC006358, RPRC000881, RPRC000267

GO:0051258	protein polymerization	P	2.93E+03	2.78E+01	6	15	over	RPRC003672, RPRC005383, RPRC010283, RPRC007667, RPRC011753, RPRC004762	RPRC014380, RPRC011530, RPRC014377, RPRC011608, RPRC011142, RPRC013158, RPRC005737, RPRC005939, RPRC011694, RPRC008372, RPRC004637, RPRC003842, RPRC003459, RPRC015390, RPRC000816
GO:0015986	ATP synthesis coupled proton transport	P	4.96E+03	5.39E+01	4	4	over	RPRC011931, RPRC008498, RPRC010550, RPRC005917	RPRC003410, RPRC012073, RPRC008312, RPRC005494
GO:0019843	rRNA binding	F	4.96E+03	5.39E+01	4	4	over	RPRC012633, RPRC012542, RPRC013371, RPRC011596	RPRC006783, RPRC014793, RPRC008479, RPRC011272
GO:0003924	GTPase activity	F	2.55E+04	3.81E+02	8	51	over	RPRC012142, RPRC003672, RPRC005383, RPRC010283, RPRC007667, RPRC015041, RPRC012140, RPRC004762	RPRC014380, RPRC013503, RPRC015134, RPRC004755, RPRC005628, RPRC011076, RPRC005457, RPRC014377, RPRC005906, RPRC013292, RPRC009003, RPRC011142, RPRC014184, RPRC008979, RPRC001144, RPRC001275, RPRC008122, RPRC001582, RPRC003793, RPRC011694, RPRC015352, RPRC009331, RPRC006970, RPRC007358, RPRC008372, RPRC001448, RPRC003416, RPRC011075, RPRC000800, RPRC012714, RPRC003842, RPRC014240, RPRC006334, RPRC002143, RPRC005936, RPRC005351, RPRC003442, RPRC005505, RPRC013790, RPRC000348, RPRC014951, RPRC004368, RPRC008854, RPRC013366, RPRC000816, RPRC015292, RPRC008666, RPRC002028, RPRC011274, RPRC012204, RPRC007250
GO:0046933	proton-transporting ATP synthase activity, rotational mechanism	F	3.32E+04	5.27E+02	3	3	over	RPRC011931, RPRC008498, RPRC005917	RPRC003410, RPRC012073, RPRC005494
GO:0022625	cytosolic large ribosomal subunit	C	5.23E+04	9.32E+02	2	0	over	RPRC014620, RPRC015127	
GO:0005874	microtubule	C	9.45E+04	2.02E+03	6	38	over	RPRC003672, RPRC007515, RPRC005383, RPRC010283, RPRC007667, RPRC004762	RPRC014380, RPRC004943, RPRC011176, RPRC014377, RPRC010393, RPRC013227, RPRC002789, RPRC004988, RPRC011142, RPRC010423, RPRC014833, RPRC002641, RPRC010968, RPRC005737, RPRC001777, RPRC006473, RPRC011694, RPRC003935, RPRC004459, RPRC008372, RPRC012400, RPRC005456, RPRC002928, RPRC011282, RPRC003842, RPRC011177, RPRC015106, RPRC009316, RPRC005789, RPRC001728, RPRC005417, RPRC015439, RPRC010046, RPRC000816, RPRC004870, RPRC012381, RPRC013987, RPRC001778

GO:0003746	translation elongation factor activity	F	1.04E+05	2.29E+03	4	15	over	RPRC012142, RPRC007684, RPRC015041, RPRC012140	RPRC012040, RPRC012111, RPRC013273, RPRC011076, RPRC009039, RPRC003914, RPRC007456, RPRC001582, RPRC005473, RPRC011027, RPRC002143, RPRC005936, RPRC002752, RPRC005351, RPRC011916
GO:0006468	protein phosphorylation	P	1.19E+05	2.88E+03	0	204	under		RPRC000167, RPRC007014, RPRC001021, RPRC014610, RPRC014789, RPRC001666, RPRC015063, RPRC002513, RPRC014924, RPRC001790, RPRC004860, RPRC003853, RPRC006286, RPRC012318, RPRC005491, RPRC003777, RPRC012932, RPRC010391, RPRC007535, RPRC004772, RPRC008485, RPRC012856, RPRC001637, RPRC002018, RPRC013487, RPRC005633, RPRC000333, RPRC004767, RPRC013865, RPRC002851, RPRC000321, RPRC009634, RPRC006659, RPRC008054, RPRC011471, RPRC008213, RPRC007813, RPRC005852, RPRC000961, RPRC001330, RPRC013013, RPRC004886, RPRC011932, RPRC012556, RPRC003051, RPRC011920, RPRC001792, RPRC008734, RPRC005019, RPRC012934, RPRC004319, RPRC015112, RPRC007939, RPRC001692, RPRC008168, RPRC004857, RPRC015100, RPRC012994, RPRC001149, RPRC007035, RPRC007915, RPRC007732, RPRC000839, RPRC009931, RPRC004881, RPRC003862, RPRC013175, RPRC011946, RPRC015450, RPRC006442, RPRC000863, RPRC014526, RPRC008334, RPRC013655, RPRC011910, RPRC013400, RPRC010713, RPRC011822, RPRC011917, RPRC001610, RPRC007432, RPRC010785, RPRC006668, RPRC008636, RPRC008784, RPRC006390, RPRC015014, RPRC002282, RPRC001741, RPRC012099, RPRC013105, RPRC012489, RPRC002914, RPRC014191, RPRC014923, RPRC009431, RPRC000699, RPRC007546, RPRC014676, RPRC009184, RPRC008241, RPRC005266, RPRC008336, RPRC005349, RPRC006894, RPRC011919, RPRC014321, RPRC002957, RPRC004754, RPRC007812, RPRC012412, RPRC011796, RPRC013036, RPRC006941, RPRC000736, RPRC004483, RPRC005095, RPRC012077, RPRC012957, RPRC002438, RPRC001555, RPRC007441, RPRC001141, RPRC012200, RPRC007991, RPRC009026, RPRC006251, RPRC003276, RPRC011265, RPRC009002, RPRC006429, RPRC006577, RPRC001750, RPRC013126, RPRC006860, RPRC002309, RPRC013778, RPRC014223, RPRC007336, RPRC000814, RPRC010180, RPRC012179, RPRC007631, RPRC006548, RPRC004230, RPRC006377, RPRC000862, RPRC000798, RPRC002038, RPRC013495, RPRC003442, RPRC001940, RPRC010394, RPRC007591, RPRC005800, RPRC008233, RPRC001693, RPRC011502, RPRC010636, RPRC001226, RPRC007981, RPRC003744, RPRC015332, RPRC002559, RPRC010134, RPRC001286, RPRC004208, RPRC008854, RPRC007119, RPRC005317, RPRC001031, RPRC012588, RPRC010991, RPRC014715, RPRC015085, RPRC004344, RPRC001740, RPRC000881, RPRC013601, RPRC014787, RPRC001102, RPRC003727, RPRC004097, RPRC015156, RPRC006467, RPRC011545, RPRC009939, RPRC000864, RPRC007788, RPRC006124, RPRC009342, RPRC013497, RPRC004717, RPRC013140, RPRC011911, RPRC006207,

									RPRC004705, RPRC001221, RPRC003273, RPRC000184-PB, RPRC000184, RPRC011516, RPRC008406, RPRC006669
GO:0045277	respiratory chain complex IV	C	1.19E+05	2.89E+03	3	7	over	RPRC012256, RPRC005409, RPRC011839	RPRC007811, RPRC000780, RPRC010093, RPRC005506, RPRC003685, RPRC005092, RPRC010094
GO:0004129	cytochrome-c oxidase activity	F	1.19E+05	2.89E+03	3	7	over	RPRC012256, RPRC005409, RPRC011839	RPRC007811, RPRC000780, RPRC010093, RPRC005506, RPRC003685, RPRC005092, RPRC010094
GO:0006123	mitochondrial electron transport, cytochrome c to oxygen	P	1.19E+05	2.89E+03	3	7	over	RPRC012256, RPRC005409, RPRC011839	RPRC007811, RPRC000780, RPRC010093, RPRC005506, RPRC003685, RPRC005092, RPRC010094
GO:0005549	odorant binding	F	1.19E+05	2.89E+03	3	7	over	RPRC000174, RPRC010284, RPRC004407	RPRC000274, RPRC009905, RPRC000476, RPRC010357, RPRC000378, RPRC014985, RPRC004408
GO:0006448	regulation of translational elongation	P	1.97E+04	5.58E+03	4	20	over	RPRC012142, RPRC007684, RPRC015041, RPRC012140	RPRC012040, RPRC014027, RPRC012111, RPRC013273, RPRC011076, RPRC009039, RPRC003914, RPRC015450, RPRC007456, RPRC001582, RPRC005473, RPRC011027, RPRC002143, RPRC005280, RPRC005936, RPRC002752, RPRC005351, RPRC011916, RPRC012971, RPRC008081
GO:0005525	GTP binding	F	2.14E+05	6.20E+03	10	120	over	RPRC012142, RPRC015257, RPRC003672, RPRC001811, RPRC005383, RPRC010283, RPRC007667, RPRC015041, RPRC012140, RPRC004762	RPRC014380, RPRC011062, RPRC015453, RPRC013503, RPRC015134, RPRC005498, RPRC008142, RPRC004755, RPRC005852, RPRC013261, RPRC007642, RPRC005628, RPRC011076, RPRC005457, RPRC003955, RPRC010209, RPRC014377, RPRC010918, RPRC003772, RPRC006830, RPRC013168, RPRC005906, RPRC013292, RPRC009003, RPRC008853, RPRC012492, RPRC015391, RPRC013377, RPRC007644, RPRC010913, RPRC010340, RPRC011142, RPRC003248, RPRC003065, RPRC004007, RPRC007066, RPRC005518, RPRC014184, RPRC008979, RPRC001144, RPRC008553, RPRC010076, RPRC001275, RPRC014716, RPRC008122, RPRC001582, RPRC003793, RPRC011694, RPRC005832, RPRC011936, RPRC015352, RPRC009331, RPRC006970, RPRC014014, RPRC009243, RPRC007358, RPRC008372, RPRC001448, RPRC011075, RPRC000800, RPRC012714, RPRC010563, RPRC003842, RPRC015449,

									RPRC009576, RPRC014240, RPRC008314, RPRC007034, RPRC005889, RPRC006334, RPRC002143, RPRC007838, RPRC013286, RPRC007643, RPRC007802, RPRC004106, RPRC014052, RPRC004254, RPRC005936, RPRC005351, RPRC012940, RPRC006938, RPRC009742, RPRC011124, RPRC003442, RPRC002735, RPRC005505, RPRC013790, RPRC015125, RPRC005482, RPRC005553, RPRC000348, RPRC014951, RPRC004368, RPRC006886, RPRC008854, RPRC003337, RPRC009223, RPRC015392, RPRC013366, RPRC000816, RPRC011864, RPRC013915, RPRC015292, RPRC004710, RPRC004646, RPRC008666, RPRC014910, RPRC008081, RPRC014929, RPRC002028, RPRC013508, RPRC011274, RPRC006184, RPRC004629, RPRC012204, RPRC000267, RPRC003970, RPRC003344, RPRC007250
GO:0098800	inner mitochondrial membrane protein complex	C	2.18E+04	6.49E+03	4	21	over	RPRC012256, RPRC008827, RPRC010550, RPRC006976	RPRC005212, RPRC013687, RPRC008337, RPRC011666, RPRC013972, RPRC013180, RPRC012073, RPRC010093, RPRC007427, RPRC004174, RPRC006787, RPRC000615, RPRC008312, RPRC004785, RPRC005989, RPRC007507, RPRC003690, RPRC005736, RPRC004687, RPRC013690, RPRC010094
GO:0045261	proton-transporting ATP synthase complex, catalytic core F(1)	C	2.73E+05	8.77E+02	2	3	over	RPRC011931, RPRC008498	RPRC003410, RPRC012073, RPRC005494
GO:0004672	protein kinase activity	F	3.06E+05	1.01E+04	1	233	under	RPRC013371	RPRC000167, RPRC007014, RPRC001021, RPRC014610, RPRC014789, RPRC001666, RPRC015063, RPRC002513, RPRC007221, RPRC014924, RPRC013917, RPRC001790, RPRC003853, RPRC004234, RPRC006286, RPRC012318, RPRC005491, RPRC003777, RPRC012932, RPRC010391, RPRC011204, RPRC007535, RPRC004772, RPRC008485, RPRC012856, RPRC009810, RPRC001637, RPRC002018, RPRC013487, RPRC008863, RPRC005633, RPRC000333, RPRC004767, RPRC000576, RPRC008787, RPRC013865, RPRC002851, RPRC000321, RPRC009634, RPRC006659, RPRC003907, RPRC008054, RPRC011471, RPRC008213, RPRC000404, RPRC007813, RPRC005852, RPRC000961, RPRC001330, RPRC013013, RPRC004886, RPRC002420, RPRC011932, RPRC011211, RPRC012556, RPRC003051, RPRC005528, RPRC011920, RPRC001792, RPRC008734, RPRC005019, RPRC012934, RPRC004319, RPRC008551, RPRC015112, RPRC007939, RPRC001692, RPRC004857, RPRC012994, RPRC004538, RPRC001149, RPRC007035, RPRC007915, RPRC013044, RPRC007732, RPRC000839, RPRC009931, RPRC004881, RPRC000803, RPRC003862, RPRC013175, RPRC014709, RPRC006868, RPRC011946, RPRC000625, RPRC015450, RPRC002358, RPRC006442, RPRC000863, RPRC014526, RPRC008334, RPRC013655, RPRC011910, RPRC010713, RPRC011822, RPRC011917, RPRC001610, RPRC010542, RPRC007432, RPRC010785, RPRC006668, RPRC008784, RPRC010466, RPRC006390, RPRC005057, RPRC012505, RPRC002282, RPRC008684, RPRC001741, RPRC012099, RPRC013105, RPRC014214,

									RPRC012489, RPRC002914, RPRC014191, RPRC014923, RPRC009431, RPRC013438, RPRC000699, RPRC007546, RPRC008560, RPRC014676, RPRC009184, RPRC008241, RPRC005266, RPRC008336, RPRC009893, RPRC001329, RPRC005349, RPRC006894, RPRC011919, RPRC014321, RPRC002957, RPRC011736, RPRC007812, RPRC012412, RPRC013036, RPRC006941, RPRC000736, RPRC004483, RPRC005095, RPRC012077, RPRC003304, RPRC012957, RPRC002438, RPRC002012, RPRC014583, RPRC002498, RPRC001555, RPRC007441, RPRC008402, RPRC001141, RPRC012200, RPRC007991, RPRC011277, RPRC006251, RPRC003276, RPRC011265, RPRC001869, RPRC009002, RPRC006429, RPRC001857, RPRC005941, RPRC001750, RPRC008628, RPRC013126, RPRC006860, RPRC015071, RPRC014223, RPRC007336, RPRC000814, RPRC010180, RPRC012179, RPRC007631, RPRC006548, RPRC004230, RPRC001255, RPRC013098, RPRC006377, RPRC000862, RPRC000798, RPRC002038, RPRC014525, RPRC014832, RPRC013495, RPRC001940, RPRC010394, RPRC007591, RPRC005429, RPRC005800, RPRC008233, RPRC001693, RPRC010636, RPRC001226, RPRC003744, RPRC015332, RPRC010612, RPRC001202, RPRC010134, RPRC001286, RPRC004208, RPRC007119, RPRC012588, RPRC010991, RPRC014715, RPRC004344, RPRC001740, RPRC000881, RPRC013601, RPRC000248, RPRC014787, RPRC001102, RPRC003727, RPRC004097, RPRC011545, RPRC000467, RPRC009939, RPRC007079, RPRC000864, RPRC007788, RPRC001488, RPRC009342, RPRC013497, RPRC013140, RPRC011911, RPRC006207, RPRC015139, RPRC004705, RPRC003273, RPRC000184-PB, RPRC000184, RPRC008406, RPRC006669
GO:0008233	peptidase activity	F	4.16E+05	1.47E+04	1	219	under	RPRC006290	RPRC009154, RPRC003097, RPRC003320, RPRC015015, RPRC009301, RPRC014463, RPRC014770, RPRC006557, RPRC010196, RPRC003168, RPRC001252, RPRC007074, RPRC011388, RPRC008278, RPRC013356, RPRC001240, RPRC012649, RPRC007157, RPRC012785, RPRC013734, RPRC012868, RPRC008963, RPRC012613, RPRC012920, RPRC003351, RPRC005414, RPRC015289, RPRC001944, RPRC010398, RPRC009977, RPRC002249, RPRC012513, RPRC001697, RPRC000913, RPRC013717, RPRC011176, RPRC000890, RPRC010309, RPRC000973, RPRC012508, RPRC009789, RPRC014294, RPRC008515, RPRC008037, RPRC013001, RPRC008587, RPRC013665, RPRC005262, RPRC006216, RPRC009127, RPRC010640, RPRC008989, RPRC004798, RPRC015124, RPRC010795, RPRC005321, RPRC009800, RPRC005067, RPRC006028, RPRC014950, RPRC012970, RPRC014407, RPRC002948, RPRC011018, RPRC006554, RPRC009294, RPRC008510, RPRC003153, RPRC008753, RPRC012487, RPRC007632, RPRC011066, RPRC013584, RPRC007692, RPRC013731, RPRC002919, RPRC003360, RPRC014196, RPRC014184, RPRC008099, RPRC002884, RPRC002310, RPRC014012, RPRC008063, RPRC011427, RPRC014338, RPRC010708, RPRC006644, RPRC000644, RPRC002696, RPRC000905, RPRC002377, RPRC015393, RPRC008831, RPRC008448, RPRC003155, RPRC008034, RPRC003864, RPRC012075, RPRC007220,

								RPRC000308, RPRC008253, RPRC002519, RPRC012465, RPRC000717, RPRC008082, RPRC014262, RPRC001796, RPRC007936, RPRC006929, RPRC001700, RPRC015288, RPRC002472, RPRC006917, RPRC003357, RPRC002798, RPRC003102, RPRC011748, RPRC010532, RPRC015188, RPRC011322, RPRC015323, RPRC007429, RPRC009226, RPRC001679, RPRC006558, RPRC010834, RPRC013528, RPRC008598, RPRC014045, RPRC009433, RPRC012786, RPRC008733, RPRC011672, RPRC006605, RPRC006753, RPRC010965, RPRC012212, RPRC005829, RPRC011129, RPRC006092, RPRC001372, RPRC002326, RPRC014897, RPRC006907, RPRC010280, RPRC005463, RPRC013126, RPRC004585, RPRC000405, RPRC013990, RPRC003247, RPRC002593, RPRC000886, RPRC003478, RPRC015385, RPRC000294, RPRC007479, RPRC011762, RPRC009595, RPRC004620, RPRC011379, RPRC013495, RPRC003708, RPRC009021, RPRC012374, RPRC000205, RPRC013009, RPRC012350, RPRC005322, RPRC007219, RPRC003608, RPRC001374, RPRC005405, RPRC009004, RPRC006898, RPRC014325, RPRC003668, RPRC004113, RPRC008281, RPRC009383, RPRC015415, RPRC007881, RPRC003230, RPRC010205, RPRC006957, RPRC000816, RPRC014468, RPRC003154, RPRC001652, RPRC003556, RPRC001747, RPRC003715, RPRC013661, RPRC007007, RPRC014699, RPRC013490, RPRC005596, RPRC005607, RPRC011776, RPRC003946, RPRC013732, RPRC015299, RPRC013349, RPRC011752, RPRC000219, RPRC014822, RPRC011433, RPRC004789, RPRC014810, RPRC009668, RPRC014173, RPRC010407, RPRC009349, RPRC009720, RPRC006893, RPRC014013, RPRC003090, RPRC000107
GO:0005811	lipid particle	C	4.58E+04	1.64E+04	3	15	over	RPRC009875, RPRC010550, RPRC012662	RPRC005070, RPRC003924, RPRC013670, RPRC014760, RPRC003294, RPRC011461, RPRC009022, RPRC008122, RPRC003793, RPRC005989, RPRC007507, RPRC012740, RPRC009418, RPRC015232, RPRC013987
GO:0000910	cytokinesis	P	4.89E+05	2.31E+04	2	6	over	RPRC007515, RPRC009875	RPRC006338, RPRC010698, RPRC003092, RPRC000800, RPRC007538, RPRC009554
GO:0005506	iron ion binding	F	4.89E+05	2.33E+04	4	32	over	RPRC010050, RPRC005729, RPRC013804, RPRC003270	RPRC003227, RPRC007511, RPRC014860, RPRC012297, RPRC009967, RPRC004769, RPRC009370, RPRC003886, RPRC012677, RPRC005454, RPRC000592, RPRC009881, RPRC005615, RPRC003942, RPRC004701, RPRC007672, RPRC007034, RPRC012024, RPRC013830, RPRC001745, RPRC013447, RPRC001881, RPRC007077, RPRC007300, RPRC005553, RPRC006400, RPRC014149, RPRC010679, RPRC004244, RPRC000626, RPRC014101, RPRC015287

GO:0005125	cytokine activity	F	4.89E+05	3.06E+04	1	0	over	RPRC002698
GO:0035690	cellular response to drug	P	4.89E+05	3.06E+04	1	0	over	RPRC007515
GO:0043274	phospholipase binding	F	4.89E+05	3.06E+04	1	0	over	RPRC007515
GO:0001837	epithelial to mesenchymal transition	P	4.89E+05	3.06E+04	1	0	over	RPRC007515
GO:0055117	regulation of cardiac muscle contraction	P	4.89E+05	3.06E+04	1	0	over	RPRC007515
GO:0000028	ribosomal small subunit assembly	P	4.89E+05	3.06E+04	1	0	over	RPRC000499
GO:0060079	regulation of excitatory postsynaptic membrane potential	P	4.89E+05	3.06E+04	1	0	over	RPRC007515
GO:0003958	NADPH-hemoprotein reductase activity	F	4.89E+05	3.06E+04	1	0	over	RPRC005729

GO:0015886	heme transport	P	4.89E+05	3.06E+04	1	0	over	RPRC009283
GO:0043024	ribosomal small subunit binding	F	4.89E+05	3.06E+04	1	0	over	RPRC001811
GO:0010992	ubiquitin homeostasis	P	4.89E+05	3.06E+04	1	0	over	RPRC012247
GO:0033192	calmodulin-dependent protein phosphatase activity	F	4.89E+05	3.06E+04	1	0	over	RPRC007515
GO:0006407	rRNA export from nucleus	P	4.89E+05	3.06E+04	1	0	over	RPRC010146
GO:0033173	calcineurin-NFAT signaling cascade	P	4.89E+05	3.06E+04	1	0	over	RPRC007515
GO:0035307	positive regulation of protein dephosphorylation	P	4.89E+05	3.06E+04	1	0	over	RPRC007515
GO:0001569	patterning of blood vessels	P	4.89E+05	3.06E+04	1	0	over	RPRC007515

GO:0010880	regulation of release of sequestered calcium ion into cytosol by sarcoplasmic reticulum	P	4.89E+05	3.06E+04	1	0	over	RPRC007515
GO:0071333	cellular response to glucose stimulus	P	4.89E+05	3.06E+04	1	0	over	RPRC007515
GO:0006336	DNA replication-independent nucleosome assembly	P	4.89E+05	3.06E+04	1	0	over	RPRC008809
GO:0042383	sarcolemma	C	4.89E+05	3.06E+04	1	0	over	RPRC007515
GO:0032587	ruffle membrane	C	4.89E+05	3.06E+04	1	0	over	RPRC001811
GO:0032516	positive regulation of phosphoprotein phosphatase activity	P	4.89E+05	3.06E+04	1	0	over	RPRC007515
GO:0046676	negative regulation of insulin secretion	P	4.89E+05	3.06E+04	1	0	over	RPRC007515
GO:0031997	N-terminal myristoylation domain binding	F	4.89E+05	3.06E+04	1	0	over	RPRC007515

GO:0031996	thioesterase binding	F	4.89E+05	3.06E+04	1	0	over	RPRC007515
GO:0031932	TORC2 complex	C	4.89E+05	3.06E+04	1	0	over	RPRC012706
GO:0031929	TOR signaling	P	4.89E+05	3.06E+04	1	0	over	RPRC012706
GO:0015439	heme-transporting ATPase activity	F	4.89E+05	3.06E+04	1	0	over	RPRC009283
GO:0048741	skeletal muscle fiber development	P	4.89E+05	3.06E+04	1	0	over	RPRC007515
GO:0000788	nuclear nucleosome	C	4.89E+05	3.06E+04	1	0	over	RPRC008809
GO:0005513	detection of calcium ion	P	4.89E+05	3.06E+04	1	0	over	RPRC007515
GO:0014883	transition between fast and slow fiber	P	4.89E+05	3.06E+04	1	0	over	RPRC007515

GO:0045955	negative regulation of calcium ion-dependent exocytosis	P	4.89E+05	3.06E+04	1	0	over	RPRC007515
GO:0051343	positive regulation of cyclic-nucleotide phosphodiesterase activity	P	4.89E+05	3.06E+04	1	0	over	RPRC007515
GO:0009882	blue light photoreceptor activity	F	4.89E+05	3.06E+04	1	0	over	RPRC005966
GO:0051298	centrosome duplication	P	4.89E+05	3.06E+04	1	0	over	RPRC015127
GO:0009785	blue light signaling pathway	P	4.89E+05	3.06E+04	1	0	over	RPRC005966
GO:0072542	protein phosphatase activator activity	F	4.89E+05	3.06E+04	1	0	over	RPRC007515
GO:0007595	lactation	P	4.89E+05	3.06E+04	1	0	over	RPRC001811
GO:0004838	L-tyrosine:2-oxoglutarate aminotransferase activity	F	4.89E+05	3.06E+04	1	0	over	RPRC000370

GO:0021762	substantia nigra development	P	4.89E+05	3.06E+04	1	0	over	RPRC007515	
GO:0060487	lung epithelial cell differentiation	P	4.89E+05	3.06E+04	1	0	over	RPRC007515	
GO:0050679	positive regulation of epithelial cell proliferation	P	4.89E+05	3.06E+04	1	0	over	RPRC001811	
GO:0071949	FAD binding	F	4.89E+05	3.06E+04	1	0	over	RPRC005966	
GO:0031492	nucleosomal DNA binding	F	4.89E+05	3.06E+04	1	0	over	RPRC008809	
GO:0031432	titin binding	F	4.89E+05	3.06E+04	1	0	over	RPRC007515	
GO:0060316	positive regulation of ryanodine-sensitive calcium-release channel activity	P	4.89E+05	3.06E+04	1	0	over	RPRC007515	
GO:0044255	cellular lipid metabolic process	P	5.31E+05	3.35E+04	0	125	under		RPRC000509, RPRC014327, RPRC000391, RPRC003013, RPRC009385, RPRC003658, RPRC003953, RPRC005070, RPRC004370, RPRC003924, RPRC007045, RPRC007511, RPRC000269, RPRC002703, RPRC013853, RPRC011566, RPRC001513, RPRC010847, RPRC000062, RPRC010421, RPRC004484, RPRC003294, RPRC004460, RPRC011537, RPRC001166, RPRC006830, RPRC004077, RPRC011992, RPRC005747, RPRC009972, RPRC015196, RPRC008232, RPRC000104, RPRC005328, RPRC008853, RPRC003507,

									RPRC013377, RPRC014850, RPRC015462, RPRC005288, RPRC000123, RPRC012853, RPRC008015, RPRC009424, RPRC006183, RPRC010649, RPRC014669, RPRC010944, RPRC011734, RPRC007894, RPRC008855, RPRC000006, RPRC006715, RPRC009774, RPRC008619, RPRC008205, RPRC000544, RPRC006225, RPRC000710, RPRC002838, RPRC007705, RPRC002909, RPRC001317, RPRC004440, RPRC002726, RPRC011103, RPRC011410, RPRC004193, RPRC000741, RPRC015176, RPRC000001, RPRC006889, RPRC007884, RPRC002821, RPRC003718, RPRC009995, RPRC000879, RPRC008881, RPRC000617, RPRC010817, RPRC008657, RPRC012307, RPRC004052, RPRC009588, RPRC013818, RPRC001691, RPRC014188, RPRC011010, RPRC004123, RPRC003666, RPRC000482, RPRC006015, RPRC006310, RPRC009771, RPRC009293, RPRC004106, RPRC014052, RPRC008911, RPRC002528, RPRC012474, RPRC004644, RPRC002835, RPRC012940, RPRC009447, RPRC008109, RPRC005310, RPRC012369, RPRC011669, RPRC002771, RPRC000757, RPRC006886, RPRC008854, RPRC015097, RPRC004037, RPRC011633, RPRC015232, RPRC009454, RPRC014149, RPRC011776, RPRC013987, RPRC009927, RPRC009513, RPRC011764, RPRC013508, RPRC000923
GO:0006355	regulation of transcription, DNA-templated	P	5.38E+05	3.40E+04	2	238	under	RPRC007515, RPRC000303	RPRC001204, RPRC001595, RPRC003870, RPRC004092, RPRC009544, RPRC009946, RPRC003475, RPRC014120, RPRC011695, RPRC000954, RPRC012076, RPRC010976, RPRC005757, RPRC007388, RPRC015365, RPRC012040, RPRC005005, RPRC007842, RPRC008887, RPRC001701, RPRC010462, RPRC005391, RPRC001625, RPRC014334, RPRC005645, RPRC012111, RPRC000026, RPRC002066, RPRC003748, RPRC007347, RPRC006647, RPRC005947, RPRC011554, RPRC001915, RPRC007801, RPRC003819, RPRC010274, RPRC003712, RPRC000737, RPRC005100, RPRC000133, RPRC003051, RPRC001899, RPRC006459, RPRC002817, RPRC003110, RPRC000583-PB, RPRC000583, RPRC000264, RPRC009163, RPRC002220, RPRC010293, RPRC006801, RPRC010281, RPRC007266, RPRC015255, RPRC009234, RPRC001910, RPRC011792, RPRC009636, RPRC011887, RPRC003791, RPRC003886, RPRC008192, RPRC001515, RPRC006478, RPRC012392, RPRC012403, RPRC000951, RPRC004402, RPRC014602, RPRC010188, RPRC006844, RPRC005264, RPRC007468, RPRC014662, RPRC010885, RPRC005973, RPRC002577, RPRC007456, RPRC006183, RPRC008087, RPRC008489, RPRC000437, RPRC015114, RPRC009962, RPRC008636, RPRC015186, RPRC010625, RPRC011161, RPRC007344, RPRC004528, RPRC005785, RPRC007025, RPRC004262, RPRC007173, RPRC001180, RPRC001127, RPRC005932, RPRC012907, RPRC007256, RPRC012429, RPRC003072, RPRC005920, RPRC007244, RPRC002110, RPRC004883, RPRC012796, RPRC005188, RPRC001429, RPRC005513, RPRC012700, RPRC005176, RPRC003681, RPRC007546, RPRC003362, RPRC000853, RPRC009438, RPRC007458, RPRC000936, RPRC012843, RPRC001317, RPRC014174, RPRC007032, RPRC000587, RPRC000025, RPRC011027, RPRC000900, RPRC006499, RPRC001122, RPRC004754, RPRC003811,

									RPRC001058, RPRC001938, RPRC007824, RPRC013788, RPRC008212, RPRC007346, RPRC002774, RPRC000824, RPRC001205, RPRC008698, RPRC015259, RPRC010273, RPRC002750, RPRC007565, RPRC002845, RPRC000239, RPRC009923, RPRC013000, RPRC001336, RPRC005261, RPRC003216, RPRC006848, RPRC008243, RPRC011448, RPRC005006, RPRC001236, RPRC004868, RPRC011914, RPRC002486, RPRC014104, RPRC007938, RPRC008019, RPRC010782, RPRC006092, RPRC002781, RPRC000767, RPRC009416, RPRC011667, RPRC003985, RPRC005805, RPRC001614, RPRC002693, RPRC010841, RPRC001993, RPRC012172, RPRC006151, RPRC010617, RPRC003790, RPRC011714, RPRC012964, RPRC009706, RPRC008357, RPRC010410, RPRC009766, RPRC011455, RPRC000282, RPRC007691, RPRC004875, RPRC015149, RPRC006660, RPRC009188, RPRC003111, RPRC011916, RPRC014254, RPRC013311, RPRC001852, RPRC001788, RPRC006094, RPRC007981, RPRC000496, RPRC002559, RPRC015268, RPRC014154, RPRC007267, RPRC001031, RPRC012906, RPRC000881, RPRC007000, RPRC010289, RPRC001186, RPRC005115, RPRC003461, RPRC015156, RPRC002606, RPRC010679, RPRC009271, RPRC004953, RPRC012157, RPRC004327, RPRC014273, RPRC013330, RPRC006379, RPRC008411, RPRC001871, RPRC004156, RPRC005003, RPRC008737, RPRC002164, RPRC005484, RPRC006502, RPRC011971, RPRC007274, RPRC015422, RPRC004192, RPRC004351, RPRC014392
GO:0000162	tryptophan biosynthetic process	P	6.47E+05	4.28E+04	2	9	over	RPRC000370, RPRC003270	RPRC009777, RPRC004312, RPRC015309, RPRC013484, RPRC000810, RPRC012146, RPRC015259, RPRC007672, RPRC000840
GO:0006571	tyrosine biosynthetic process	P	6.47E+05	4.28E+04	2	9	over	RPRC000370, RPRC003270	RPRC009777, RPRC004312, RPRC015309, RPRC013484, RPRC000810, RPRC012146, RPRC015259, RPRC007672, RPRC000840
GO:0033177	proton-transporting two-sector ATPase complex, proton-transporting domain	C	6.47E+05	4.28E+04	2	9	over	RPRC006543, RPRC010550	RPRC013834, RPRC005881, RPRC008403, RPRC015179, RPRC001199, RPRC008312, RPRC005989, RPRC007426, RPRC001198
GO:0009094	L-phenylalanine biosynthetic process	P	6.47E+05	4.28E+04	2	9	over	RPRC000370, RPRC003270	RPRC009777, RPRC004312, RPRC015309, RPRC013484, RPRC000810, RPRC012146, RPRC015259, RPRC007672, RPRC000840

GO:0019904	protein domain specific binding	F	6.47E+05	4.28E+04	2	9	over	RPRC007515, RPRC011742	RPRC011237, RPRC011822, RPRC008500, RPRC003362, RPRC008219, RPRC001496, RPRC010679, RPRC013325, RPRC012128
GO:0006446	regulation of translational initiation	P	6.65E+05	4.45E+04	4	40	over	RPRC013802, RPRC002700, RPRC006840, RPRC010378	RPRC009699, RPRC009587, RPRC002208, RPRC006459, RPRC001018, RPRC006116, RPRC003914, RPRC013955, RPRC013696, RPRC000566, RPRC014786, RPRC006478, RPRC001101, RPRC012870, RPRC006844, RPRC004935, RPRC006903, RPRC004362, RPRC011953, RPRC010218, RPRC003864, RPRC010290, RPRC006499, RPRC013852, RPRC001448, RPRC007334, RPRC015147, RPRC010634, RPRC007926, RPRC007607, RPRC008557, RPRC003801, RPRC004922, RPRC004846, RPRC015292, RPRC007462, RPRC003851, RPRC007923, RPRC014905, RPRC002383
GO:0003743	translation initiation factor activity	F	6.65E+05	4.45E+04	4	40	over	RPRC013802, RPRC002700, RPRC006840, RPRC010378	RPRC009699, RPRC009587, RPRC002208, RPRC006459, RPRC001018, RPRC006116, RPRC003914, RPRC013955, RPRC013696, RPRC000566, RPRC014786, RPRC006478, RPRC001101, RPRC012870, RPRC006844, RPRC004935, RPRC006903, RPRC004362, RPRC011953, RPRC010218, RPRC003864, RPRC010290, RPRC006499, RPRC013852, RPRC001448, RPRC007334, RPRC015147, RPRC010634, RPRC007926, RPRC007607, RPRC008557, RPRC003801, RPRC004922, RPRC004846, RPRC015292, RPRC007462, RPRC003851, RPRC007923, RPRC014905, RPRC002383
GO:0016209	antioxidant activity	F	7.11E+05	4.82E+03	3	24	over	RPRC002923, RPRC015388, RPRC006756	RPRC009934, RPRC007595, RPRC007347, RPRC014110, RPRC012922, RPRC007468, RPRC007213, RPRC000982, RPRC011108, RPRC007344, RPRC009148, RPRC004883, RPRC005064, RPRC007907, RPRC011796, RPRC015195, RPRC011500, RPRC000422-PB, RPRC015401, RPRC007484, RPRC015008, RPRC004965, RPRC004717, RPRC011126

GO annotation terms for enriched transcripts in male library

GO-ID	Term	Category*	FDR	P-Value	N° of genes in dataset D.E.	N° of genes in *R. prolixus* genome	Over/Under represented	VectorBase code of genes dataset D.E.	VectorBase code of genes in *R. prolixus* genome
GO:0003735	structural constituent of ribosome	F	1.26E-27	4.28E-31	34	58	over	RPRC013077, RPRC010378, RPRC010853, RPRC005980, RPRC002470, RPRC003510, RPRC010146, RPRC007328, RPRC005430, RPRC014363, RPRC005701, RPRC013167, RPRC012162, RPRC012542, RPRC000499, RPRC006329, RPRC012387, RPRC011937, RPRC009189, RPRC014845, RPRC014419, RPRC010286, RPRC011596, RPRC009337, RPRC007230, RPRC012158, RPRC013146, RPRC009702, RPRC015127, RPRC003327, RPRC012354, RPRC013504, RPRC002923, RPRC002544	RPRC006309, RPRC008799, RPRC007825, RPRC002520, RPRC014365, RPRC002822, RPRC007856, RPRC009039, RPRC004296, RPRC011999, RPRC015409, RPRC012941, RPRC011456, RPRC002505, RPRC012363, RPRC000922, RPRC006118, RPRC014793, RPRC013786, RPRC004269, RPRC012784, RPRC007451, RPRC006758, RPRC012353, RPRC008479, RPRC010054, RPRC006392, RPRC000972, RPRC003014, RPRC002869, RPRC009716, RPRC002698, RPRC005011, RPRC009871, RPRC010192, RPRC012072, RPRC006051, RPRC012633, RPRC013169, RPRC010872, RPRC011260, RPRC010405, RPRC003768, RPRC003980, RPRC005724, RPRC006974, RPRC009873, RPRC013371, RPRC014620, RPRC015001, RPRC005389, RPRC012711, RPRC002897, RPRC004303, RPRC001328, RPRC014587, RPRC010809, RPRC013851
GO:0022627	cytosolic small ribosomal subunit	C	2.23E+01	1.21E-02	4	0	over	RPRC010146, RPRC000499, RPRC013504, RPRC002923	
GO:0005549	odorant binding	F	8.94E+00	5.16E-01	5	5	over	RPRC009905, RPRC000174, RPRC004407, RPRC004408, RPRC010284	RPRC000274, RPRC000476, RPRC010357, RPRC000378, RPRC014985
GO:0070062	extracellular exosome	C	3.91E+02	2.52E+00	5	8	over	RPRC012247, RPRC001811, RPRC008809, RPRC005383, RPRC007515	RPRC015027, RPRC012892, RPRC007468, RPRC009243, RPRC011182, RPRC006358, RPRC000881, RPRC000267
GO:0005200	structural constituent of cytoskeleton	F	8.00E+02	5.71E+00	5	10	over	RPRC005383, RPRC003672, RPRC010283, RPRC009875, RPRC007667	RPRC014380, RPRC014377, RPRC004762, RPRC005328, RPRC011694, RPRC008372, RPRC008343, RPRC003842, RPRC015390, RPRC000816
GO:0003924	GTPase activity	F	1.33E+04	1.08E+02	7	52	over	RPRC015041, RPRC005383, RPRC003672, RPRC010283, RPRC012140, RPRC012142, RPRC007667	RPRC014380, RPRC013503, RPRC015134, RPRC004755, RPRC005628, RPRC011076, RPRC005457, RPRC014377, RPRC005906, RPRC013292, RPRC004762, RPRC009003, RPRC011142, RPRC014184, RPRC008979, RPRC001144, RPRC001275, RPRC008122, RPRC001582, RPRC003793, RPRC011694, RPRC015352, RPRC009331, RPRC006970, RPRC007358, RPRC008372, RPRC001448, RPRC003416, RPRC011075, RPRC000800, RPRC012714, RPRC003842, RPRC014240, RPRC006334, RPRC002143, RPRC005936, RPRC005351, RPRC003442, RPRC005505, RPRC013790, RPRC000348, RPRC014951, RPRC004368, RPRC008854, RPRC013366, RPRC000816, RPRC015292, RPRC008666, RPRC002028, RPRC011274, RPRC012204, RPRC007250

GO:0003746	translation elongation factor activity	F	3.94E+04	3.78E+02	4	15	over	RPRC015041, RPRC007684, RPRC012140, RPRC012142	RPRC012040, RPRC012111, RPRC013273, RPRC011076, RPRC009039, RPRC003914, RPRC007456, RPRC001582, RPRC005473, RPRC011027, RPRC002143, RPRC005936, RPRC002752, RPRC005351, RPRC011916
GO:0051258	protein polymerization	P	5.56E+04	5.67E+02	4	17	over	RPRC005383, RPRC003672, RPRC010283, RPRC007667	RPRC014380, RPRC011530, RPRC014377, RPRC011608, RPRC004762, RPRC011142, RPRC013158, RPRC005737, RPRC005939, RPRC011694, RPRC011753, RPRC008372, RPRC004637, RPRC003842, RPRC003459, RPRC015390, RPRC000816
GO:0005525	GTP binding	F	6.79E+04	7.24E+02	9	121	over	RPRC015041, RPRC001811, RPRC005383, RPRC003672, RPRC015257, RPRC010283, RPRC012140, RPRC012142, RPRC007667	RPRC014380, RPRC011062, RPRC015453, RPRC013503, RPRC015134, RPRC005498, RPRC008142, RPRC004755, RPRC005852, RPRC013261, RPRC007642, RPRC005628, RPRC011076, RPRC005457, RPRC003955, RPRC010209, RPRC014377, RPRC010918, RPRC003772, RPRC006830, RPRC013168, RPRC005906, RPRC013292, RPRC004762, RPRC009003, RPRC008853, RPRC012492, RPRC015391, RPRC013377, RPRC007644, RPRC010913, RPRC010340, RPRC011142, RPRC003248, RPRC003065, RPRC004007, RPRC007066, RPRC005518, RPRC014184, RPRC008979, RPRC001144, RPRC008553, RPRC010076, RPRC001275, RPRC014716, RPRC008122, RPRC001582, RPRC003793, RPRC011694, RPRC005832, RPRC011936, RPRC015352, RPRC009331, RPRC006970, RPRC014014, RPRC009243, RPRC007358, RPRC008372, RPRC001448, RPRC011075, RPRC000800, RPRC012714, RPRC010563, RPRC003842, RPRC015449, RPRC009576, RPRC014240, RPRC008314, RPRC007034, RPRC005889, RPRC006334, RPRC002143, RPRC007838, RPRC013286, RPRC007643, RPRC007802, RPRC004106, RPRC014052, RPRC004254, RPRC005936, RPRC005351, RPRC012940, RPRC006938, RPRC009742, RPRC011124, RPRC003442, RPRC002735, RPRC005505, RPRC013790, RPRC015125, RPRC005482, RPRC005553, RPRC000348, RPRC014951, RPRC004368, RPRC006886, RPRC008854, RPRC003337, RPRC009223, RPRC015392, RPRC013366, RPRC000816, RPRC011864, RPRC013915, RPRC015292, RPRC004710, RPRC004646, RPRC008666, RPRC014910, RPRC008081, RPRC014929, RPRC002028, RPRC013508, RPRC011274, RPRC006184, RPRC004629, RPRC012204, RPRC000267, RPRC003970, RPRC003344, RPRC007250
GO:0006448	regulation of translational elongation	P	8.22E+04	9.64E+02	4	20	over	RPRC015041, RPRC007684, RPRC012140, RPRC012142	RPRC012040, RPRC014027, RPRC012111, RPRC013273, RPRC011076, RPRC009039, RPRC003914, RPRC015450, RPRC007456, RPRC001582, RPRC005473, RPRC011027, RPRC002143, RPRC005280, RPRC005936, RPRC002752, RPRC005351, RPRC011916, RPRC012971, RPRC008081
GO:0005874	microtubule	C	1.09E+05	1.34E+03	5	39	over	RPRC005383, RPRC003672, RPRC010283, RPRC007515, RPRC007667	RPRC014380, RPRC004943, RPRC011176, RPRC014377, RPRC010393, RPRC004762, RPRC013227, RPRC002789, RPRC004988, RPRC011142, RPRC010423, RPRC014833, RPRC002641, RPRC010968, RPRC005737, RPRC001777, RPRC006473, RPRC011694, RPRC003935, RPRC004459, RPRC008372, RPRC012400, RPRC005456, RPRC002928, RPRC011282, RPRC003842, RPRC011177, RPRC015106, RPRC009316, RPRC005789, RPRC001728, RPRC005417, RPRC015439, RPRC010046, RPRC000816, RPRC004870, RPRC012381, RPRC013987, RPRC001778

GO:0019843	rRNA binding	F	4.89E+04	9.24E+03	2	6	over	RPRC012542, RPRC011596	RPRC006783, RPRC014793, RPRC008479, RPRC012633, RPRC011272, RPRC013371
GO:0031966	mitochondrial membrane	C	4.89E+04	1.49E+04	5	72	over	RPRC012468, RPRC009600, RPRC008679, RPRC014245, RPRC008827	RPRC002932, RPRC007811, RPRC005212, RPRC013687, RPRC010550, RPRC007228, RPRC014199, RPRC008337, RPRC005338, RPRC011666, RPRC005457, RPRC001573, RPRC014843, RPRC009203, RPRC013972, RPRC014986, RPRC002403, RPRC004686, RPRC014579, RPRC007118, RPRC013180, RPRC011887, RPRC012256, RPRC011461, RPRC010901, RPRC012073, RPRC014200, RPRC010093, RPRC014686, RPRC014196, RPRC009424, RPRC004840, RPRC006892, RPRC007427, RPRC001996, RPRC007829, RPRC004174, RPRC006787, RPRC006213, RPRC000054, RPRC014752, RPRC000615, RPRC008312, RPRC007996, RPRC012755, RPRC002614, RPRC004785, RPRC005989, RPRC001714, RPRC006310, RPRC007507, RPRC014920, RPRC003690, RPRC013678, RPRC005912, RPRC015468, RPRC005505, RPRC005736, RPRC004687, RPRC013690, RPRC003118, RPRC007669, RPRC008997, RPRC005092, RPRC003301, RPRC010094, RPRC008335, RPRC009254, RPRC009413, RPRC006976, RPRC009230, RPRC002637
GO:0019904	protein domain specific binding	F	4.89E+04	1.75E+04	2	9	over	RPRC007515, RPRC011742	RPRC011237, RPRC011822, RPRC008500, RPRC003362, RPRC008219, RPRC001496, RPRC010679, RPRC013325, RPRC012128
GO:0035690	cellular response to drug	P	4.89E+04	1.90E+02	1	0	over	RPRC007515	
GO:0043274	phospholipase binding	F	4.89E+04	1.90E+02	1	0	over	RPRC007515	
GO:0001837	epithelial to mesenchymal transition	P	4.89E+04	1.90E+02	1	0	over	RPRC007515	
GO:0055117	regulation of cardiac muscle contraction	P	4.89E+04	1.90E+02	1	0	over	RPRC007515	
GO:0000028	ribosomal small subunit assembly	P	4.89E+04	1.90E+02	1	0	over	RPRC000499	
GO:0060079	regulation of excitatory postsynaptic membrane potential	P	4.89E+04	1.90E+02	1	0	over	RPRC007515	
GO:0003958	NADPH-hemoprotein reductase activity	F	4.89E+04	1.90E+02	1	0	over	RPRC005729	

GO:0043024	ribosomal small subunit binding	F	4.89E+04	1.90E+02	1	0	over	RPRC001811
GO:0010992	ubiquitin homeostasis	P	4.89E+04	1.90E+02	1	0	over	RPRC012247
GO:0033192	calmodulin-dependent protein phosphatase activity	F	4.89E+04	1.90E+02	1	0	over	RPRC007515
GO:0006407	rRNA export from nucleus	P	4.89E+04	1.90E+02	1	0	over	RPRC010146
GO:0033173	calcineurin-NFAT signaling cascade	P	4.89E+04	1.90E+02	1	0	over	RPRC007515
GO:0035307	positive regulation of protein dephosphorylation	P	4.89E+04	1.90E+02	1	0	over	RPRC007515
GO:0001569	patterning of blood vessels	P	4.89E+04	1.90E+02	1	0	over	RPRC007515
GO:0010880	regulation of release of sequestered calcium ion into cytosol by sarcoplasmic reticulum	P	4.89E+04	1.90E+02	1	0	over	RPRC007515
GO:0071333	cellular response to glucose stimulus	P	4.89E+04	1.90E+02	1	0	over	RPRC007515
GO:0006336	DNA replication-independent nucleosome assembly	P	4.89E+04	1.90E+02	1	0	over	RPRC008809
GO:0042383	sarcolemma	C	4.89E+04	1.90E+02	1	0	over	RPRC007515
GO:0032587	ruffle membrane	C	4.89E+04	1.90E+02	1	0	over	RPRC001811
GO:0032516	positive regulation of phosphoprotein phosphatase activity	P	4.89E+04	1.90E+02	1	0	over	RPRC007515

GO:0046676	negative regulation of insulin secretion	P	4.89E+04	1.90E+02	1	0	over	RPRC007515
GO:0031997	N-terminal myristoylation domain binding	F	4.89E+04	1.90E+02	1	0	over	RPRC007515
GO:0031996	thioesterase binding	F	4.89E+04	1.90E+02	1	0	over	RPRC007515
GO:0048741	skeletal muscle fiber development	P	4.89E+04	1.90E+02	1	0	over	RPRC007515
GO:0000788	nuclear nucleosome	C	4.89E+04	1.90E+02	1	0	over	RPRC008809
GO:0005513	detection of calcium ion	P	4.89E+04	1.90E+02	1	0	over	RPRC007515
GO:0014883	transition between fast and slow fiber	P	4.89E+04	1.90E+02	1	0	over	RPRC007515
GO:0045955	negative regulation of calcium ion-dependent exocytosis	P	4.89E+04	1.90E+02	1	0	over	RPRC007515
GO:0051343	positive regulation of cyclic-nucleotide phosphodiesterase activity	P	4.89E+04	1.90E+02	1	0	over	RPRC007515
GO:0009882	blue light photoreceptor activity	F	4.89E+04	1.90E+02	1	0	over	RPRC005966
GO:0051298	centrosome duplication	P	4.89E+04	1.90E+02	1	0	over	RPRC015127
GO:0009785	blue light signaling pathway	P	4.89E+04	1.90E+02	1	0	over	RPRC005966
GO:0072542	protein phosphatase activator activity	F	4.89E+04	1.90E+02	1	0	over	RPRC007515

GO:0007595	lactation	P	4.89E+04	1.90E+02	1	0	over	RPRC001811	
GO:0004838	L-tyrosine:2-oxoglutarate aminotransferase activity	F	4.89E+04	1.90E+02	1	0	over	RPRC000370	
GO:0021762	substantia nigra development	P	4.89E+04	1.90E+02	1	0	over	RPRC007515	
GO:0060487	lung epithelial cell differentiation	P	4.89E+04	1.90E+02	1	0	over	RPRC007515	
GO:0050679	positive regulation of epithelial cell proliferation	P	4.89E+04	1.90E+02	1	0	over	RPRC001811	
GO:0071949	FAD binding	F	4.89E+04	1.90E+02	1	0	over	RPRC005966	
GO:0031492	nucleosomal DNA binding	F	4.89E+04	1.90E+02	1	0	over	RPRC008809	
GO:0031432	titin binding	F	4.89E+04	1.90E+02	1	0	over	RPRC007515	
GO:0060316	positive regulation of ryanodine-sensitive calcium-release channel activity	P	4.89E+04	1.90E+02	1	0	over	RPRC007515	
GO:0015405	P-P-bond-hydrolysis-driven transmembrane transporter activity	F	5.29E+05	2.10E+04	4	52	over	RPRC003672, RPRC011931, RPRC010283, RPRC008827	RPRC005058, RPRC000735, RPRC007564, RPRC009520, RPRC006226, RPRC000681, RPRC005207, RPRC003694, RPRC013230, RPRC010550, RPRC006811, RPRC011093, RPRC003410, RPRC014158, RPRC010031, RPRC010516, RPRC014377, RPRC005604, RPRC006069, RPRC007159, RPRC002303, RPRC014874, RPRC014798, RPRC012073, RPRC001782, RPRC000668, RPRC002063, RPRC002938, RPRC000444, RPRC004866, RPRC004849, RPRC003978, RPRC014771, RPRC008833, RPRC005592, RPRC005917, RPRC008498, RPRC005989, RPRC000180, RPRC005499, RPRC007341, RPRC002302, RPRC002676, RPRC013435, RPRC001633, RPRC001781, RPRC005494, RPRC010075, RPRC009283, RPRC009992, RPRC015363, RPRC003113
GO:0004672	protein kinase activity	F	5.70E+05	2.28E+03	0	234	under		RPRC000167, RPRC007014, RPRC001021, RPRC014610, RPRC014789, RPRC001666, RPRC015063, RPRC002513, RPRC007221, RPRC014924, RPRC013917, RPRC001790, RPRC003853, RPRC004234, RPRC006286, RPRC012318, RPRC005491, RPRC003777, RPRC012932, RPRC010391,

RPRC011204, RPRC007535, RPRC004772, RPRC008485,
RPRC012856, RPRC009810, RPRC001637, RPRC002018,
RPRC013487, RPRC008863, RPRC005633, RPRC000333,
RPRC004767, RPRC000576, RPRC008787, RPRC013865,
RPRC002851, RPRC000321, RPRC009634, RPRC006659,
RPRC003907, RPRC008054, RPRC011471, RPRC008213,
RPRC000404, RPRC007813, RPRC005852, RPRC000961,
RPRC001330, RPRC013013, RPRC004886, RPRC002420,
RPRC011932, RPRC011211, RPRC012556, RPRC003051,
RPRC005528, RPRC011920, RPRC001792, RPRC008734,
RPRC005019, RPRC012934, RPRC004319, RPRC008551,
RPRC015112, RPRC007939, RPRC001692, RPRC004857,
RPRC012994, RPRC004538, RPRC001149, RPRC007035,
RPRC007915, RPRC013044, RPRC007732, RPRC000839,
RPRC009931, RPRC004881, RPRC000803, RPRC003862,
RPRC013175, RPRC014709, RPRC006868, RPRC011946,
RPRC000625, RPRC015450, RPRC002358, RPRC006442,
RPRC000863, RPRC014526, RPRC008334, RPRC013655,
RPRC011910, RPRC010713, RPRC011822, RPRC011917,
RPRC001610, RPRC010542, RPRC007432, RPRC010785,
RPRC006668, RPRC008784, RPRC010466, RPRC006390,
RPRC005057, RPRC012505, RPRC002282, RPRC008684,
RPRC001741, RPRC012099, RPRC013105, RPRC014214,
RPRC012489, RPRC002914, RPRC014191, RPRC014923,
RPRC009431, RPRC013438, RPRC000699, RPRC007546,
RPRC008560, RPRC014676, RPRC009184, RPRC008241,
RPRC005266, RPRC008336, RPRC009893, RPRC001329,
RPRC005349, RPRC006894, RPRC011919, RPRC014321,
RPRC002957, RPRC011736, RPRC007812, RPRC012412,
RPRC013036, RPRC006941, RPRC000736, RPRC004483,
RPRC005095, RPRC012077, RPRC003304, RPRC012957,
RPRC002438, RPRC002012, RPRC014583, RPRC002498,
RPRC001555, RPRC007441, RPRC008402, RPRC001141,
RPRC012200, RPRC007991, RPRC011277, RPRC006251,
RPRC003276, RPRC011265, RPRC001869, RPRC009002,
RPRC006429, RPRC001857, RPRC005941, RPRC001750,
RPRC008628, RPRC013126, RPRC006860, RPRC015071,
RPRC014223, RPRC007336, RPRC000814, RPRC010180,
RPRC012179, RPRC007631, RPRC006548, RPRC004230,
RPRC001255, RPRC013098, RPRC006377, RPRC000862,
RPRC000798, RPRC002038, RPRC014525, RPRC014832,
RPRC013495, RPRC001940, RPRC010394, RPRC007591,
RPRC005429, RPRC005800, RPRC008233, RPRC001693,
RPRC010636, RPRC001226, RPRC003744, RPRC015332,
RPRC013371, RPRC010612, RPRC001202, RPRC010134,
RPRC001286, RPRC004208, RPRC007119, RPRC012588,
RPRC010991, RPRC014715, RPRC004344, RPRC001740,
RPRC000881, RPRC013601, RPRC000248, RPRC014787,
RPRC001102, RPRC003727, RPRC004097, RPRC011545,
RPRC000467, RPRC009939, RPRC007079, RPRC000864,
RPRC007788, RPRC001488, RPRC009342, RPRC013497,
RPRC013140, RPRC011911, RPRC006207, RPRC015139,
RPRC004705, RPRC003273, RPRC000184-PB, RPRC000184,
RPRC008406, RPRC006669

GO:0005506	iron ion binding	F	6.76E+05	3.00E+04	3	33	over	RPRC005729, RPRC013804, RPRC010050	RPRC003227, RPRC007511, RPRC014860, RPRC003270, RPRC012297, RPRC009967, RPRC004769, RPRC009370, RPRC003886, RPRC012677, RPRC005454, RPRC000592, RPRC009881, RPRC005615, RPRC003942, RPRC004701, RPRC007672, RPRC007034, RPRC012024, RPRC013830, RPRC001745, RPRC013447, RPRC001881, RPRC007077, RPRC007300, RPRC005553, RPRC006400, RPRC014149, RPRC010679, RPRC004244, RPRC000626, RPRC014101, RPRC015287
GO:0019752	carboxylic acid metabolic process	P	6.76E+05	3.24E+04	2	388	under	RPRC000370, RPRC012101	RPRC015027, RPRC000509, RPRC001671, RPRC013946, RPRC012423, RPRC007493, RPRC006550, RPRC000391, RPRC002708, RPRC000167, RPRC003658, RPRC001276, RPRC000633, RPRC003403, RPRC001021, RPRC015405, RPRC001985, RPRC014789, RPRC009934, RPRC014948, RPRC001666, RPRC003634, RPRC010184, RPRC015063, RPRC002756, RPRC007783, RPRC004819, RPRC011281, RPRC014924, RPRC001790, RPRC013492, RPRC007559, RPRC003853, RPRC014180, RPRC006286, RPRC012318, RPRC005491, RPRC005070, RPRC012932, RPRC010391, RPRC011204, RPRC008242, RPRC000706, RPRC007535, RPRC004772, RPRC008485, RPRC003924, RPRC012856, RPRC009810, RPRC013965, RPRC013487, RPRC001371, RPRC007045, RPRC012271, RPRC011985, RPRC014027, RPRC007511, RPRC000830, RPRC011093, RPRC006747, RPRC004365, RPRC006481, RPRC001708, RPRC004619, RPRC000333, RPRC004767, RPRC000269, RPRC002851, RPRC000321, RPRC004755, RPRC009634, RPRC006659, RPRC013853, RPRC008054, RPRC011471, RPRC014329, RPRC007654, RPRC002213, RPRC007813, RPRC005852, RPRC007961, RPRC000961, RPRC003819, RPRC017364, RPRC002846, RPRC001330, RPRC001266, RPRC004898, RPRC009777, RPRC009522, RPRC006547, RPRC002420, RPRC010918, RPRC011932, RPRC003317, RPRC012556, RPRC011040, RPRC014110, RPRC003294, RPRC004312, RPRC013824, RPRC004460, RPRC005262, RPRC006216, RPRC001166, RPRC008734, RPRC003270, RPRC009972, RPRC006997, RPRC012297, RPRC004319, RPRC013417, RPRC007939, RPRC015196, RPRC000678, RPRC004709, RPRC004857, RPRC004538, RPRC007035, RPRC007915, RPRC001042, RPRC011497, RPRC011963, RPRC005878, RPRC003507, RPRC007732, RPRC003241, RPRC011951, RPRC005281, RPRC000720, RPRC004881, RPRC014626, RPRC003862, RPRC005830, RPRC013175, RPRC003384, RPRC015450, RPRC007958, RPRC004569, RPRC000863, RPRC014526, RPRC011858, RPRC000123, RPRC015309, RPRC007468, RPRC000366, RPRC012853, RPRC013655, RPRC007934, RPRC007213, RPRC013484, RPRC009424, RPRC010713, RPRC015369, RPRC011822, RPRC006023, RPRC014669, RPRC010383, RPRC000751, RPRC008784, RPRC010466, RPRC003650, RPRC006561, RPRC001056, RPRC013543, RPRC012677, RPRC011161, RPRC006390, RPRC000758, RPRC000810, RPRC002282, RPRC008684, RPRC012027, RPRC008270, RPRC005454, RPRC000734, RPRC005920, RPRC009774, RPRC015074, RPRC009709, RPRC006225, RPRC000805, RPRC002914, RPRC011132, RPRC014191, RPRC014923, RPRC002838, RPRC007546,

								RPRC007705, RPRC014676, RPRC001246, RPRC013089, RPRC012146, RPRC009184, RPRC008241, RPRC005266, RPRC005880, RPRC002909, RPRC011848, RPRC007936, RPRC008472, RPRC010727, RPRC001370, RPRC012270, RPRC001317, RPRC010142, RPRC011103, RPRC006894, RPRC009816, RPRC014321, RPRC002957, RPRC004447, RPRC000741, RPRC011736, RPRC012986, RPRC000001, RPRC011470, RPRC012572, RPRC007812, RPRC005059, RPRC008762, RPRC008698, RPRC007907, RPRC013935, RPRC002762, RPRC015259, RPRC013036, RPRC007884, RPRC006941, RPRC006156, RPRC011234, RPRC004961, RPRC004483, RPRC003878, RPRC001496, RPRC004566, RPRC012077, RPRC006056, RPRC005261, RPRC012957, RPRC002438, RPRC000700, RPRC009433, RPRC006670, RPRC012065, RPRC008881, RPRC000617, RPRC010965, RPRC000605, RPRC009250, RPRC000689, RPRC007441, RPRC004466, RPRC001714, RPRC008402, RPRC009588, RPRC012200, RPRC001691, RPRC013393, RPRC007672, RPRC009014, RPRC003276, RPRC014028, RPRC011265, RPRC006919, RPRC011519, RPRC014790, RPRC007034, RPRC009002, RPRC000589, RPRC009404, RPRC006429, RPRC003666, RPRC012609, RPRC006015, RPRC015095, RPRC011336, RPRC005315, RPRC002704, RPRC007572, RPRC002067, RPRC008628, RPRC013126, RPRC004278, RPRC006310, RPRC000826, RPRC014478, RPRC015071, RPRC005280, RPRC014223, RPRC007336, RPRC013364, RPRC002286, RPRC005617, RPRC004644, RPRC006548, RPRC010103, RPRC004230, RPRC001255, RPRC006377, RPRC013423, RPRC000862, RPRC000798, RPRC002038, RPRC003932, RPRC004556, RPRC009021, RPRC003283, RPRC001940, RPRC013009, RPRC010394, RPRC015125, RPRC015273, RPRC007591, RPRC005641, RPRC005800, RPRC005884, RPRC003927, RPRC008233, RPRC004858, RPRC005310, RPRC012369, RPRC005553, RPRC001055, RPRC003744, RPRC013785, RPRC014739, RPRC015332, RPRC013371, RPRC014473, RPRC013318, RPRC000757, RPRC013466, RPRC001202, RPRC001286, RPRC012588, RPRC002281, RPRC010991, RPRC008535, RPRC014715, RPRC012174, RPRC004344, RPRC001740, RPRC000881, RPRC013601, RPRC014787, RPRC015232, RPRC014946, RPRC007402, RPRC001102, RPRC003727, RPRC004097, RPRC004817, RPRC006053, RPRC000467, RPRC015292, RPRC009939, RPRC000698, RPRC013987, RPRC009927, RPRC000864, RPRC011764, RPRC008081, RPRC001488, RPRC009342, RPRC008483, RPRC011604, RPRC005003, RPRC013497, RPRC013140, RPRC005826, RPRC014101, RPRC006207, RPRC000840, RPRC002885, RPRC012364, RPRC006491, RPRC000184-PB, RPRC000184, RPRC008406, RPRC000923, RPRC013461, RPRC004924, RPRC006160, RPRC000574, RPRC004605, RPRC012678, RPRC010212
GO:0006468	protein phosphorylation	P	6.76E+05	3.33E+04	0	204	under	RPRC000167, RPRC007014, RPRC001021, RPRC014610, RPRC014789, RPRC001666, RPRC015063, RPRC002513, RPRC014924, RPRC001790, RPRC004860, RPRC003853, RPRC006286, RPRC012318, RPRC005491, RPRC003777, RPRC012932, RPRC010391, RPRC007535, RPRC004772, RPRC008485, RPRC012856, RPRC001637, RPRC002018,

									RPRC013487, RPRC005633, RPRC000333, RPRC004767, RPRC013865, RPRC002851, RPRC000321, RPRC009634, RPRC006659, RPRC008054, RPRC011471, RPRC008213, RPRC007813, RPRC005852, RPRC000961, RPRC001330, RPRC013013, RPRC004886, RPRC011932, RPRC012556, RPRC003051, RPRC011920, RPRC001792, RPRC008734, RPRC005019, RPRC012934, RPRC004319, RPRC015112, RPRC007939, RPRC001692, RPRC008168, RPRC004857, RPRC015100, RPRC012994, RPRC001149, RPRC007035, RPRC007915, RPRC007732, RPRC000839, RPRC009931, RPRC004881, RPRC003862, RPRC013175, RPRC011946, RPRC015450, RPRC006442, RPRC000863, RPRC014526, RPRC008334, RPRC013655, RPRC011910, RPRC013400, RPRC010713, RPRC011822, RPRC011917, RPRC001610, RPRC007432, RPRC010785, RPRC006668, RPRC008636, RPRC008784, RPRC006390, RPRC015014, RPRC002282, RPRC001741, RPRC012099, RPRC013105, RPRC012489, RPRC002914, RPRC014191, RPRC014923, RPRC009431, RPRC000699, RPRC007546, RPRC014676, RPRC009184, RPRC008241, RPRC005266, RPRC008336, RPRC005349, RPRC006894, RPRC011919, RPRC014321, RPRC002957, RPRC004754, RPRC007812, RPRC012412, RPRC011796, RPRC013036, RPRC006941, RPRC000736, RPRC004483, RPRC005095, RPRC012077, RPRC012957, RPRC002438, RPRC001555, RPRC007441, RPRC001141, RPRC012200, RPRC007991, RPRC009026, RPRC006251, RPRC003276, RPRC011265, RPRC009002, RPRC006429, RPRC006577, RPRC001750, RPRC013126, RPRC006860, RPRC002309, RPRC013778, RPRC014223, RPRC007336, RPRC000814, RPRC010180, RPRC012179, RPRC007631, RPRC006548, RPRC004230, RPRC006377, RPRC000862, RPRC000798, RPRC002038, RPRC013495, RPRC003442, RPRC001940, RPRC010394, RPRC007591, RPRC005800, RPRC008233, RPRC001693, RPRC011502, RPRC010636, RPRC001226, RPRC007981, RPRC003744, RPRC015332, RPRC002559, RPRC010134, RPRC001286, RPRC004208, RPRC008854, RPRC007119, RPRC005317, RPRC001031, RPRC012588, RPRC010991, RPRC014715, RPRC015085, RPRC004344, RPRC001740, RPRC000881, RPRC013601, RPRC014787, RPRC001102, RPRC003727, RPRC004097, RPRC015156, RPRC006467, RPRC011545, RPRC009939, RPRC000864, RPRC007788, RPRC006124, RPRC009342, RPRC013497, RPRC004717, RPRC013140, RPRC011911, RPRC006207, RPRC004705, RPRC001221, RPRC003273, RPRC000184-PB, RPRC000184, RPRC011516, RPRC008406, RPRC006669
GO:0044822	poly(A) RNA binding	F	6.76E+05	3.59E+04	2	14	over	RPRC012247, RPRC001811	RPRC014058, RPRC004312, RPRC008256, RPRC013641, RPRC007920, RPRC014200, RPRC013650, RPRC002774, RPRC015311, RPRC011731, RPRC010044, RPRC009259, RPRC000704, RPRC005412
GO:0043388	positive regulation of DNA binding	P	6.76E+05	3.75E+04	1	1	over	RPRC001811	RPRC006379

GO:0033555	multicellular organismal response to stress	P	6.76E+05	3.75E+04	1	1	over	RPRC007515	RPRC007025
GO:0004550	nucleoside diphosphate kinase activity	F	6.76E+05	3.75E+04	1	1	over	RPRC001811	RPRC009259
GO:0004478	methionine adenosyltransferase activity	F	6.76E+05	3.75E+04	1	1	over	RPRC012101	RPRC004605
GO:0000022	mitotic spindle elongation	P	6.76E+05	3.75E+04	1	1	over	RPRC015127	RPRC004459
GO:0006556	S-adenosylmethionine biosynthetic process	P	6.76E+05	3.75E+04	1	1	over	RPRC012101	RPRC004605
GO:0002181	cytoplasmic translation	P	6.76E+05	3.75E+04	1	1	over	RPRC010146	RPRC007370
GO:0002027	regulation of heart rate	P	6.76E+05	3.75E+04	1	1	over	RPRC007515	RPRC003117
GO:0002020	protease binding	F	6.76E+05	3.75E+04	1	1	over	RPRC012247	RPRC009942
GO:0005853	eukaryotic translation elongation factor 1 complex	C	6.76E+05	3.75E+04	1	1	over	RPRC007684	RPRC009039
GO:0000980	RNA polymerase II distal enhancer sequence-specific DNA binding	F	6.76E+05	3.75E+04	1	1	over	RPRC008809	RPRC005115
GO:0030307	positive regulation of cell growth	P	6.76E+05	3.75E+04	1	1	over	RPRC008809	RPRC005417
GO:0000979	RNA polymerase II core promoter sequence-specific DNA binding	F	6.76E+05	3.75E+04	1	1	over	RPRC008809	RPRC003362
GO:0006228	UTP biosynthetic process	P	6.76E+05	3.75E+04	1	1	over	RPRC001811	RPRC009259

GO:0032465	regulation of cytokinesis	P	6.76E+05	3.75E+04	1	1	over	RPRC007515	RPRC010698
GO:0022625	cytosolic large ribosomal subunit	C	6.76E+05	3.75E+04	1	1	over	RPRC015127	RPRC014620
GO:0006183	GTP biosynthetic process	P	6.76E+05	3.75E+04	1	1	over	RPRC001811	RPRC009259
GO:0006165	nucleoside diphosphate phosphorylation	P	6.76E+05	3.75E+04	1	1	over	RPRC001811	RPRC009259
GO:0008308	voltage-gated anion channel activity	F	6.76E+05	3.75E+04	1	1	over	RPRC008679	RPRC002738
GO:0030018	Z disc	C	6.76E+05	3.75E+04	1	1	over	RPRC007515	RPRC005070
GO:0044070	regulation of anion transport	P	6.76E+05	3.75E+04	1	1	over	RPRC008679	RPRC002738
GO:0090304	nucleic acid metabolic process	P	6.91E+05	3.85E+04	5	600	under	RPRC001811, RPRC007515, RPRC000303, RPRC001993, RPRC002923	RPRC002530, RPRC005809, RPRC011257, RPRC002052, RPRC004677, RPRC002868, RPRC006645, RPRC001204, RPRC003097, RPRC001352, RPRC012104, RPRC001595, RPRC002708, RPRC001181, RPRC015417, RPRC003870, RPRC004092, RPRC009544, RPRC013615, RPRC000633, RPRC011381, RPRC009946, RPRC015246, RPRC014239, RPRC012578, RPRC005200, RPRC007818, RPRC013846, RPRC003475, RPRC014948, RPRC015382, RPRC000723, RPRC011559, RPRC001252, RPRC014120, RPRC002756, RPRC009444, RPRC011695, RPRC000954, RPRC012076, RPRC006362, RPRC001578, RPRC011535, RPRC010976, RPRC005757, RPRC010498, RPRC012318, RPRC009929, RPRC007388, RPRC009208, RPRC000445, RPRC015365, RPRC004772, RPRC012040, RPRC005005, RPRC002242, RPRC007842, RPRC002485, RPRC011276, RPRC008887, RPRC013965, RPRC001701, RPRC003594, RPRC013551, RPRC009575, RPRC010876, RPRC003996, RPRC001318, RPRC014027, RPRC010462, RPRC004377, RPRC005391, RPRC001625, RPRC014334, RPRC001052, RPRC008249, RPRC005645, RPRC012111, RPRC001295, RPRC006895, RPRC004365, RPRC000588, RPRC000026, RPRC002078, RPRC004767, RPRC003824, RPRC014967, RPRC002066, RPRC010776, RPRC013030, RPRC003748, RPRC012892, RPRC004436, RPRC000659, RPRC007347, RPRC006488, RPRC001768, RPRC002934, RPRC006647, RPRC008042, RPRC005947, RPRC011554, RPRC015419, RPRC001915, RPRC006783, RPRC007801, RPRC003819, RPRC010274,

RPRC003712, RPRC000737, RPRC002846, RPRC005100,
RPRC004962, RPRC002527, RPRC015384, RPRC009522,
RPRC010250, RPRC007076, RPRC008970, RPRC014058,
RPRC000133, RPRC003772, RPRC003051, RPRC001899,
RPRC004312, RPRC014046, RPRC011283, RPRC006459,
RPRC001230, RPRC012703, RPRC013232, RPRC013960,
RPRC008256, RPRC010818, RPRC006033, RPRC006913,
RPRC008085, RPRC006997, RPRC002817, RPRC003110,
RPRC005155, RPRC000583-PB, RPRC000583, RPRC006116,
RPRC009960, RPRC002073, RPRC000264, RPRC004709,
RPRC010878, RPRC007832, RPRC004284, RPRC002475,
RPRC011987, RPRC009163, RPRC001680, RPRC002941,
RPRC002220, RPRC010293, RPRC012515, RPRC006801,
RPRC010281, RPRC008789, RPRC007266, RPRC015255,
RPRC009234, RPRC008948, RPRC001910, RPRC002789,
RPRC011792, RPRC005559, RPRC002227, RPRC009636,
RPRC011887, RPRC014077, RPRC015391, RPRC009317,
RPRC003791, RPRC001834, RPRC003017, RPRC006649,
RPRC003886, RPRC014850, RPRC007899, RPRC008192,
RPRC001515, RPRC006478, RPRC011863, RPRC013039,
RPRC012392, RPRC012403, RPRC001587, RPRC015314,
RPRC000951, RPRC009536, RPRC005618, RPRC005038,
RPRC004402, RPRC015450, RPRC014602, RPRC010188,
RPRC007290, RPRC004474, RPRC006844, RPRC009755,
RPRC015055, RPRC014112, RPRC001487, RPRC005264,
RPRC002505, RPRC001794, RPRC001073, RPRC015309,
RPRC007468, RPRC014514, RPRC014662, RPRC010885,
RPRC005973, RPRC002577, RPRC007456, RPRC006597,
RPRC006183, RPRC007137, RPRC008087, RPRC010649,
RPRC009560, RPRC005790, RPRC008489, RPRC012044,
RPRC000437, RPRC012924, RPRC015114, RPRC008075,
RPRC009962, RPRC006987, RPRC001132, RPRC004202,
RPRC008636, RPRC015186, RPRC005395, RPRC010625,
RPRC006561, RPRC013543, RPRC011161, RPRC008772,
RPRC007344, RPRC004528, RPRC007439, RPRC005785,
RPRC007025, RPRC004262, RPRC015014, RPRC007173,
RPRC000230, RPRC001180, RPRC001127, RPRC001996,
RPRC012027, RPRC005932, RPRC012907, RPRC007256,
RPRC009945, RPRC017361, RPRC012429, RPRC012641,
RPRC003072, RPRC011061, RPRC005920, RPRC005856,
RPRC007244, RPRC002110, RPRC010820, RPRC013010,
RPRC004883, RPRC012003, RPRC001919, RPRC009698,
RPRC000692, RPRC007327, RPRC001812, RPRC014521,
RPRC005673, RPRC012796, RPRC010987, RPRC005188,
RPRC001429, RPRC005513, RPRC012700, RPRC013502,
RPRC001163, RPRC013650, RPRC005176, RPRC014688,
RPRC003374, RPRC003681, RPRC000137, RPRC007546,
RPRC001967, RPRC011044, RPRC010899, RPRC013089,
RPRC003362, RPRC015057, RPRC014114, RPRC014209,
RPRC004559, RPRC000853, RPRC015459, RPRC009438,
RPRC008738, RPRC009331, RPRC007458, RPRC007670,
RPRC010727, RPRC001943, RPRC010875, RPRC008631,
RPRC000936, RPRC012843, RPRC011900, RPRC001317,
RPRC014174, RPRC005485, RPRC011665, RPRC004778,
RPRC004057, RPRC007032, RPRC014162, RPRC000587,
RPRC006332, RPRC000025, RPRC004671, RPRC012596,

RPRC014416, RPRC001134, RPRC003823, RPRC004045,
RPRC011027, RPRC015093, RPRC014150, RPRC000900,
RPRC006499, RPRC001122, RPRC000320, RPRC004754,
RPRC003811, RPRC001058, RPRC001938, RPRC007824,
RPRC013788, RPRC008212, RPRC007346, RPRC002774,
RPRC000824, RPRC001205, RPRC013112, RPRC008698,
RPRC006953, RPRC007334, RPRC002762, RPRC015259,
RPRC003002, RPRC004093, RPRC010273, RPRC009788,
RPRC014293, RPRC007258, RPRC002750, RPRC007565,
RPRC002845, RPRC004081, RPRC013172, RPRC001519,
RPRC009298, RPRC012631, RPRC009521, RPRC005023,
RPRC000239, RPRC004164, RPRC009923, RPRC007382,
RPRC004630, RPRC013000, RPRC013664, RPRC001400,
RPRC010332, RPRC001336, RPRC010989, RPRC005261,
RPRC007370, RPRC011441, RPRC006670, RPRC011672,
RPRC002107, RPRC000594, RPRC002414, RPRC003216,
RPRC006848, RPRC008243, RPRC011448, RPRC002084,
RPRC013416, RPRC005006, RPRC005154, RPRC001236,
RPRC004868, RPRC005882, RPRC011914, RPRC012136,
RPRC009026, RPRC014104, RPRC002486, RPRC007938,
RPRC008019, RPRC006358, RPRC010782, RPRC006092,
RPRC006972, RPRC008728, RPRC014028, RPRC002781,
RPRC004601, RPRC010558, RPRC000767, RPRC009416,
RPRC011731, RPRC011667, RPRC004844, RPRC003985,
RPRC005805, RPRC007277, RPRC001614, RPRC009404,
RPRC015095, RPRC011909, RPRC015106, RPRC002693,
RPRC002704, RPRC012586, RPRC010841, RPRC011791,
RPRC012172, RPRC006151, RPRC008628, RPRC005239,
RPRC000731, RPRC006405, RPRC010617, RPRC003790,
RPRC003801, RPRC005125, RPRC005280, RPRC001598,
RPRC008286, RPRC013364, RPRC007579, RPRC013459,
RPRC011714, RPRC012964, RPRC009706, RPRC002031,
RPRC008369, RPRC010900, RPRC001331, RPRC008983,
RPRC013849, RPRC008357, RPRC012633, RPRC012007,
RPRC010410, RPRC009766, RPRC009045, RPRC005996,
RPRC003318, RPRC011455, RPRC000282, RPRC007691,
RPRC010896, RPRC004875, RPRC011857, RPRC008747,
RPRC015149, RPRC002895, RPRC009742, RPRC003442,
RPRC005346, RPRC011526, RPRC006660, RPRC012776,
RPRC011833, RPRC004946, RPRC014171, RPRC009188,
RPRC008393, RPRC002086, RPRC003111, RPRC002404,
RPRC011916, RPRC014254, RPRC013311, RPRC001852,
RPRC001788, RPRC006731, RPRC004190, RPRC011745,
RPRC004922, RPRC007833, RPRC006094, RPRC007981,
RPRC012581, RPRC002062, RPRC009259, RPRC006814,
RPRC000496, RPRC013785, RPRC015332, RPRC002559,
RPRC015268, RPRC014473, RPRC007586, RPRC008611,
RPRC009076, RPRC014154, RPRC007267, RPRC009235,
RPRC001031, RPRC006631, RPRC012895, RPRC012906,
RPRC009064, RPRC000881, RPRC007000, RPRC010289,
RPRC007402, RPRC014373, RPRC008288, RPRC001186,
RPRC003875, RPRC005115, RPRC003461, RPRC010500,
RPRC015156, RPRC002606, RPRC004499, RPRC014456,
RPRC010679, RPRC004870, RPRC007940, RPRC008021,
RPRC005926, RPRC000626, RPRC009271, RPRC012476,
RPRC003703, RPRC004953, RPRC000455, RPRC012157,

									RPRC004327, RPRC014273, RPRC013330, RPRC006379, RPRC008411, RPRC001871, RPRC010029, RPRC000443, RPRC004156, RPRC008081, RPRC001552, RPRC013975, RPRC001647, RPRC002081, RPRC005003, RPRC008737, RPRC014037, RPRC000674, RPRC005412, RPRC000840, RPRC002164, RPRC006609, RPRC008873, RPRC008152, RPRC000609, RPRC006491, RPRC005484, RPRC013325, RPRC006502, RPRC002968, RPRC011971, RPRC005738, RPRC004936, RPRC006172, RPRC007274, RPRC011345, RPRC000752, RPRC015422, RPRC013620, RPRC004192, RPRC004351, RPRC006107, RPRC014392, RPRC009780
GO:0070469	respiratory chain	C	8.15E+04	4.72E+04	3	40	over	RPRC012468, RPRC013804, RPRC014245	RPRC007811, RPRC005212, RPRC013687, RPRC005845, RPRC005338, RPRC005409, RPRC014986, RPRC012922, RPRC011839, RPRC014579, RPRC007118, RPRC013180, RPRC012256, RPRC010901, RPRC000780, RPRC010093, RPRC005506, RPRC004840, RPRC007427, RPRC004174, RPRC006787, RPRC006213, RPRC000054, RPRC012755, RPRC002614, RPRC004701, RPRC004785, RPRC007507, RPRC003690, RPRC003685, RPRC004687, RPRC013690, RPRC003118, RPRC007669, RPRC008997, RPRC005092, RPRC003301, RPRC010094, RPRC006976, RPRC002637
GO:0015992	proton transport	P	8.25E+05	4.89E+04	4	69	over	RPRC012468, RPRC006543, RPRC011931, RPRC014245	RPRC002932, RPRC007811, RPRC004960, RPRC013687, RPRC009520, RPRC005845, RPRC013834, RPRC003694, RPRC010550, RPRC007228, RPRC005881, RPRC013237, RPRC002473, RPRC005338, RPRC003410, RPRC005409, RPRC006718, RPRC007956, RPRC014986, RPRC012922, RPRC011839, RPRC014579, RPRC007118, RPRC015179, RPRC012256, RPRC010901, RPRC012073, RPRC015291, RPRC000780, RPRC010093, RPRC001392, RPRC005506, RPRC004840, RPRC009396, RPRC007427, RPRC002938, RPRC001199, RPRC006213, RPRC012879, RPRC001151, RPRC008312, RPRC012755, RPRC004849, RPRC002614, RPRC008833, RPRC005917, RPRC000044, RPRC008498, RPRC005989, RPRC007341, RPRC007507, RPRC003690, RPRC005494, RPRC003685, RPRC007686, RPRC008169, RPRC013690, RPRC003118, RPRC007426, RPRC007669, RPRC001198, RPRC003568, RPRC008997, RPRC005092, RPRC003301, RPRC009992, RPRC010094, RPRC003113, RPRC002637
GO:0006446	regulation of translational initiation	P	8.25E+05	5.00E+04	3	41	over	RPRC010378, RPRC013802, RPRC002700	RPRC009699, RPRC006840, RPRC009587, RPRC002208, RPRC006459, RPRC001018, RPRC006116, RPRC003914, RPRC013955, RPRC013696, RPRC000566, RPRC014786, RPRC006478, RPRC001101, RPRC012870, RPRC006844, RPRC004935, RPRC006903, RPRC004362, RPRC011953, RPRC010218, RPRC003864, RPRC010290, RPRC006499, RPRC013852, RPRC001448, RPRC007334, RPRC015147, RPRC010634, RPRC007926, RPRC007607, RPRC008557, RPRC003801, RPRC004922, RPRC004846, RPRC015292, RPRC007462, RPRC003851, RPRC007923, RPRC014905, RPRC002383
GO:0003743	translation initiation factor activity	F	8.25E+05	5.00E+04	3	41	over	RPRC010378, RPRC013802, RPRC002700	RPRC009699, RPRC006840, RPRC009587, RPRC002208, RPRC006459, RPRC001018, RPRC006116, RPRC003914, RPRC013955, RPRC013696, RPRC000566, RPRC014786, RPRC006478, RPRC001101, RPRC012870, RPRC006844, RPRC004935, RPRC006903, RPRC004362, RPRC011953, RPRC010218, RPRC003864, RPRC010290, RPRC006499,

RPRC013852, RPRC001448, RPRC007334, RPRC015147,
RPRC010634, RPRC007926, RPRC007607, RPRC008557,
RPRC003801, RPRC004922, RPRC004846, RPRC015292,
RPRC007462, RPRC003851, RPRC007923, RPRC014905,
RPRC002383

Table S2- Comparison of GO annotation terms for enriched transcripts between libraries. (*) P: Biological process, F: Molecular function and C: Cellular component. (**) Fields with 0 mean that this function is underrepresented in differentially expressed genes dataset.

GO ID	GO category*	GO term	Genes in each sample**		
			Larvae	Female	Male
GO:0000028	P	ribosomal small subunit assembly	1	1	1
GO:0000788	C	nuclear nucleosome	1	1	1
GO:0001569	P	patterning of blood vessels	1	1	1
GO:0001837	P	epithelial to mesenchymal transition	1	1	1
GO:0003735	F	structural constituent of ribosome	63	62	34
GO:0003746	F	translation elongation factor activity	4	4	4
GO:0003924	F	GTPase activity	10	8	7
GO:0003958	F	NADPH-hemoprotein reductase activity	1	1	1
GO:0004672	F	protein kinase activity	2	1	0
GO:0004838	F	L-tyrosine:2-oxoglutarate aminotransferase activity	1	1	1
GO:0005200	F	structural constituent of cytoskeleton	6	6	5
GO:0005513	P	detection of calcium ion	1	1	1
GO:0005525	F	GTP binding	12	10	9
GO:0005549	F	odorant binding	4	3	5
GO:0005874	C	microtubule	6	6	5
GO:0006336	P	DNA replication-independent nucleosome assembly	1	1	1
GO:0006407	P	rRNA export from nucleus	1	1	1
GO:0006448	P	regulation of translational elongation	4	4	4
GO:0006468	P	protein phosphorylation	1	0	0
GO:0007595	P	lactation	1	1	1
GO:0009785	P	blue light signaling pathway	1	1	1
GO:0009882	F	blue light photoreceptor activity	1	1	1
GO:0010880	P	regulation of release of sequestered calcium ion into cytosol by sarcoplasmic reticulum	1	1	1
GO:0010992	P	ubiquitin homeostasis	1	1	1
GO:0014883	P	transition between fast and slow fiber	1	1	1
GO:0019843	F	rRNA binding	4	4	2
GO:0019904	F	protein domain specific binding	2	2	2
GO:0021762	P	substantia nigra development	1	1	1
GO:0022625	C	cytosolic large ribosomal subunit	2	2	1
GO:0022627	C	cytosolic small ribosomal subunit	4	4	4
GO:0031432	F	titin binding	1	1	1

GO ID	GO category*	GO term	Genes in each sample**		
			Larvae	Female	Male
GO:0031492	F	nucleosomal DNA binding	1	1	1
GO:0031996	F	thioesterase binding	1	1	1
GO:0031997	F	N-terminal myristoylation domain binding	1	1	1
GO:0032516	P	positive regulation of phosphoprotein phosphatase activity	1	1	1
GO:0032587	C	ruffle membrane	1	1	1
GO:0033173	P	calcineurin-NFAT signaling cascade	1	1	1
GO:0033192	F	calmodulin-dependent protein phosphatase activity	1	1	1
GO:0035307	P	positive regulation of protein dephosphorylation	1	1	1
GO:0035690	P	cellular response to drug	1	1	1
GO:0042383	C	sarcolemma	1	1	1
GO:0043024	F	ribosomal small subunit binding	1	1	1
GO:0043274	F	phospholipase binding	1	1	1
GO:0045955	P	negative regulation of calcium ion-dependent exocytosis	1	1	1
GO:0046676	P	negative regulation of insulin secretion	1	1	1
GO:0048741	P	skeletal muscle fiber development	1	1	1
GO:0050679	P	positive regulation of epithelial cell proliferation	1	1	1
GO:0051258	P	protein polymerization	5	6	4
GO:0051298	P	centrosome duplication	1	1	1
GO:0051343	P	positive regulation of cyclic-nucleotide phosphodiesterase activity	1	1	1
GO:0055117	P	regulation of cardiac muscle contraction	1	1	1
GO:0060079	P	regulation of excitatory postsynaptic membrane potential	1	1	1
GO:0060316	P	positive regulation of ryanodine-sensitive calcium-release channel activity	1	1	1
GO:0060487	P	lung epithelial cell differentiation	1	1	1
GO:0070062	C	extracellular exosome	5	5	5
GO:0071333	P	cellular response to glucose stimulus	1	1	1
GO:0071949	F	FAD binding	1	1	1
GO:0072542	F	protein phosphatase activator activity	1	1	1
GO:0015986	P	ATP synthesis coupled proton transport	5	4	-
GO:0046933	F	proton-transporting ATP synthase activity, rotational mechanism	4	3	-
GO:0008233	F	peptidase activity	1	1	-
GO:0000910	P	cytokinesis	2	2	-
GO:0005125	F	cytokine activity	1	1	-
GO:0015886	P	heme transport	1	1	-
GO:0031932	C	TORC2 complex	1	1	-
GO:0031929	P	TOR signaling	1	1	-

GO ID	GO category*	GO term	Genes in each sample**		
			Larvae	Female	Male
GO:0033177	C	proton-transporting two-sector ATPase complex, proton-transporting domain	2	2	-
GO:0016209	F	antioxidant activity	3	3	-
GO:0005506	F	iron ion binding	-	4	3
GO:0006446	P	regulation of translational initiation	-	4	3
GO:0003743	F	translation initiation factor activity	-	4	3
GO:0019752	P	carboxylic acid metabolic process	5	-	2
GO:0008061	F	chitin binding	3	-	-
GO:0006892	P	post-Golgi vesicle-mediated transport	1	-	-
GO:0000275	C	mitochondrial proton-transporting ATP synthase complex, catalytic core F(1)	1	-	-
GO:0008537	C	proteasome activator complex	1	-	-
GO:1902036	P	regulation of hematopoietic stem cell differentiation	1	-	-
GO:0046961	F	proton-transporting ATPase activity, rotational mechanism	2	-	-
GO:0006351	P	transcription, DNA-templated	3	-	-
GO:0006030	P	chitin metabolic process	3	-	-
GO:0045277	C	respiratory chain complex IV	-	3	-
GO:0004129	F	cytochrome-c oxidase activity	-	3	-
GO:0006123	P	mitochondrial electron transport, cytochrome c to oxygen	-	3	-
GO:0098800	C	inner mitochondrial membrane protein complex	-	4	-
GO:0045261	C	proton-transporting ATP synthase complex, catalytic core F(1)	-	2	-
GO:0005811	C	lipid particle	-	3	-
GO:0044255	P	cellular lipid metabolic process	-	0	-
GO:0006355	P	regulation of transcription, DNA-templated	-	2	-
GO:0000162	P	tryptophan biosynthetic process	-	2	-
GO:0006571	P	tyrosine biosynthetic process	-	2	-
GO:0009094	P	L-phenylalanine biosynthetic process	-	2	-
GO:0031966	C	mitochondrial membrane	-	-	5
GO:0015405	F	P-P-bond-hydrolysis-driven transmembrane transporter activity	-	-	4
GO:0044822	F	poly(A) RNA binding	-	-	2
GO:0043388	P	positive regulation of DNA binding	-	-	1
GO:0033555	P	multicellular organismal response to stress	-	-	1
GO:0004550	F	nucleoside diphosphate kinase activity	-	-	1
GO:0004478	F	methionine adenosyltransferase activity	-	-	1
GO:0000022	P	mitotic spindle elongation	-	-	1
GO:0006556	P	S-adenosylmethionine biosynthetic process	-	-	1
GO:0002181	P	cytoplasmic translation	-	-	1

GO ID	GO category*	GO term	Genes in each sample**		
			Larvae	Female	Male
GO:0002027	P	regulation of heart rate	-	-	1
GO:0002020	F	protease binding	-	-	1
GO:0005853	C	eukaryotic translation elongation factor 1 complex	-	-	1
GO:0000980	F	RNA polymerase II distal enhancer sequence-specific DNA binding	-	-	1
GO:0030307	P	positive regulation of cell growth	-	-	1
GO:0000979	F	RNA polymerase II core promoter sequence-specific DNA binding	-	-	1
GO:0006228	P	UTP biosynthetic process	-	-	1
GO:0032465	P	regulation of cytokinesis	-	-	1
GO:0006183	P	GTP biosynthetic process	-	-	1
GO:0006165	P	nucleoside diphosphate phosphorylation	-	-	1
GO:0008308	F	voltage-gated anion channel activity	-	-	1
GO:0030018	C	Z disc	-	-	1
GO:0044070	P	regulation of anion transport	-	-	1
GO:0090304	P	nucleic acid metabolic process	-	-	5
GO:0070469	C	respiratory chain	-	-	3
GO:0015992	P	proton transport	-	-	4

Table S3 – Target genes studied in this report and their corresponding VectorBase codes. Codes: NTE – N-terminus missing in gap, CTE – C-terminus missing in gap, INT – internal exon missing in gap, FIX – assembly was repaired; JOI – gene model spans scaffolds; FX - gene model repaired based on de novo transcriptome assemblies. Multiple suffixes are abbreviated to single letters). Absent: Genes without VectorBase code.** Protein families included in the sensory scatter plot.

**Odorant receptors**	VectorBase code
Orco	RPRC000476
Or1	RPRC000579
Or2	RPRC001689
Or3	RPRC000059
Or4	RPRC000120
Or5	RPRC000083
Or6-Pseudogene	-
Or7	RPRC002152
Or8	RPRC001154
Or9	RPRC000895
Or10-Pseudogene	-
Or11-Pseudogene	-
Or12NTE	RPRC000376
Or13	RPRC000362
Or14	RPRC000166
Or15-Pseudogene	-
Or16	RPRC000420
Or17	RPRC001628
Or18	RPRC000616
Or19-CTE	RPRC000449
Or20	RPRC000573
Or21	RPRC006570
Or22	RPRC000375
Or23	RPRC000149
Or24	RPRC001980
Or25	RPRC000029
Or26	RPRC000619
Or27-FIX	RPRC000331
Or28-FIX	Not in VB
Or29-CTE	RPRC000125
Or30	RPRC000209
Or31	RPRC000423
Or32	RPRC000043
Or33	RPRC000235
Or34-JOI	Not in VB
Or35-Pseudogene	
Or36	RPRC000337
Or37	RPRC000456
Or38	RPRC000530

**Odorant receptors**	VectorBase code
Or39	RPRC000041
Or40	RPRC000229
Or41	RPRC000146
Or42	RPRC000108
Or43	RPRC000140
Or44	RPRC001291
Or45-FIX	Not in VB
Or46	RPRC001292
Or47	RPRC000413
Or48	RPRC001293
Or49	RPRC000433
Or50	RPRC001623
Or51	RPRC000507
Or52	RPRC000201
Or53	RPRC002122
Or54	RPRC000055
Or55	RPRC002161
Or56	RPRC002162
Or57	RPRC000318
Or58	RPRC000526
Or59-JC	RPRC004587
Or60	RPRC002105
Or61	RPRC000208
Or62	RPRC002135
Or63	RPRC002136
Or64-NTE	RPRC000095
Or65-NTE	RPRC000132
Or66-NTE	RPRC000452
Or67	RPRC000222
Or68	RPRC000541
Or69-NTE	Not in VB
Or70-FX	RPRC000459
Or71-NTE	RPRC000490
Or72	RPRC000053
Or73	RPRC000585
Or74-NTE	RPRC000200
Or75-NTE	RPRC000245
Or76-NTE	RPRC001928
Or77-NTE	RPRC000468
Or78-NTE	RPRC000733
Or79-FX	RPRC000202
Or80-FX	RPRC000441
Or81-NTE	RPRC000363
Or82-FX	RPRC002113
Or83-FX	RPRC006981

**Odorant receptors**	VectorBase code
Or84-FX	RPRC000431
Or85-NTE	RPRC000882
Or86-NTE	RPRC000079
Or87-FX	RPRC000317
Or88-NTE	RPRC000607
Or89-FX	RPRC000165
Or90-NTE	RPRC000249
Or91-NTE	RPRC000093
Or92-FX	RPRC002009
Or93-NTE	RPRC002158
Or94-NTE	RPRC002156
Or95-FX	RPRC002157
Or96-NTE	RPRC001767
Or97-FX	RPRC002120
Or98-FX	RPRC000136
Or99-FX	RPRC000065
Or100-NTE	RPRC000522
Or101	RPRC000188
Or102	Not in VB
Or103	RPRC000601
Or104-FIX	RPRC009806
Or105	RPRC000255
Or106	RPRC000371
Or107-FX	RPRC001726
Or108	RPRC000301
Or109-NTE	RPRC001799
Or110-FX	RPRC002141
Or111-FX	RPRC002142
Or112	RPRC000555
Or113	RPRC001309
Or114	RPRC004306
Or115-FIX	Not in VB

**Ionotropic receptors****		Comments
Ir25a	RPRC000589	
Ir8a	RPRC000349	
Ir76b	RPRC000469	
Ir93a-FX	RPRC008486	
Ir21a-FX	RPRC001826	
Ir68a-FX	RPRC00328	
Ir40a-FX	RPRC000383	
Ir41a-FX	RPRC000191	
Ir41b-FIX	RPRC000489	
Ir41c	RPRC013097	
Ir75a-FX	RPRC002112	

Ir75b	RPRC017348	
Ir75c	RPRC017347	
Ir75d-FIX	RPRC000105	Pseudogene in the genome
Ir75e-FX	RPRC000047	
Ir75f-FX	RPRC002124	
Ir75g-FIX	RPRC017347	Pseudogene in the genome
Ir75h	RPRC017355	
Ir75i	RPRC017354	
Ir75j	RPRC017353	
Ir75k	RPRC000314	
Ir75l	RPRC017352	
Ir75m	RPRC000247	
Ir75n	RPRC000007	
Ir75o	RPRC017345	
Ir75p	RPRC017346	
Ir101	RPRC005659	
Ir102	RPRC000847	
Ir103-FX	RPRC000379	
Ir104	RPRC002996	
Ir105-FX	RPRC000538	
Ir106-FX	RPRC000846	
Ir107-NTE	RPRC000385	
**Gustatory receptors****		
Gr1FX	RPRC001549	
Gr2FX	RPRC002023	
Gr3	RPRC001795	
Gr4	RPRC000056	
Gr5	RPRC001197	
Gr6	RPRC000553	
Gr7	RPRC001196	
Gr8	RPRC001195	
Gr9	RPRC000388	
Gr10	RPRC000106	
Gr11	RPRC001194	
Gr12FIX	RPRC002134	
Gr13	RPRC002133	
Gr14a	RPRC008717	
Gr14b	RPRC008717	
Gr15	RPRC001115	
Gr16	RPRC000730	
Gr17	RPRC001279	
Gr18	RPRC000336	
Gr19	RPRC000763	
Gr20	RPRC000451	
Gr21	RPRC000130	

Gr22	RPRC000073
Gr23a	RPRC017351
Gr23b	RPRC017351
Gr24	RPRC001956
Gr25	RPRC000216
Gr26	RPRC000368
Gr27	RPRC000068
Gr28	RPRC000290

Odorant binding proteins

OBP1	RPRC010284
OBP2	RPRC000378
OBP3	RPRC000527
OBP4	RPRC000274
OBP5	RPRC000221 – Coding erro
OBP6	RPRC004413
OBP7	RPRC000311
OBP8FX	RPRC000564
OBP9	RPRC000194
OBP10	RPRC000560
OBP11	RPRC004408
OBP12	RPRC000492
OBP13FX	RPRC000177
OBP14	RPRC004407
OBP15	RPRC008413
OBP16	RPRC011610
OBP17	RPRC000118
OBP18FX	RPRC001924
OBP19FX	RPRC000593
OBP20	RPRC007008
OBP21	RPRC001925
OBP22FX	RPRC000115
OBP23FX	RPRC000174
OBP24	RPRC009905
OBP25FX	RPRC000257
OBP26	RPRC000182
OBP27	RPRC009914

Chemosensory proteins

CSP1	RPRC013223
CSP2	RPRC013221
CSP3FX	RPRC013280
CSP4	RPRC013285
CSP5FX	RPRC013218
CSP6	RPRC000332
CSP7	RPRC010107

CSP8	RPRC000631
CSP9	RPRC000470
CSP10	RPRC007979
CSP11	RPRC011935
CSP12	RPRC011928
CSP13	RPRC011926
CSP14	RPRC006791
CSP15-NTE	RPRC000546
CSP16	RPRC000327
CSP17FX	RPRC007134
CSP18	RPRC000242
CSP19	RPRC000126

**CHEb****	
CHEB1-FX	RPRC004662
CHEB2	RPRC004663
CHEB3	RPRC004664
CHEB4	RPRC004665
CHEB5	RPRC004666

**SNMP y CD36**	
SNMP1A**	RPRC013907
SNMP1B-FX**	RPRC013910
SNMP1c-NT**	RPRC000399
SNMPd1**	RPRC002720
SNMP2-FX **	RPRC002754
SCRB3-FX	RPRC010585
SCR5-FX	RPRC013192
SCRB6-FX	RPRC006064
SCRB7	RPRC001716
SCRB8A-FX	RPRC015098
SCRB8b-FX	RPRC003042
SCRB8c-FX	RPRC012472
SCRB8d-FX	RPRC003036
SCRB8e-FX	RPRC003038
SCRB9-FX	RPRC013775
Group2[a]	RPRC006308
Group2b-a	RPRC015150
Grop2b-b	RPRC009306

**Transient receptor potential channel****	VectorBase code	Comments
NAN	Absent	
IAV-FX	RPRC002111	
NOMPC	RPRC000544	
PAIN-FX	RPRC000454	

TRPA1 ISOFORM A	RPRC000422	
TRPA1 ISOFORM B	RPRC000422	
WTRW	RPRC000127	
TRPA5A	RPRC001596	Former annotation *Pyrexia*
TRPA5B-FX	RPRC001597	
TRPA5C-FX	RPRC000570	
TRP-GAMMA	RPRC000350	
TRPL	RPRC000224	
PKD2	RPRC014320	
TRPM	RPRC007108	
TRPML-FX	RPRC009679	
**Pickpocket receptors ****		
PPK-LIKE1	RPRC013510	
PPK-LIKE2	RPRC000471	
PPK-LIKE3	Absent	
PKK-LIKE4	RPRC000341	
PPK-LIKE5	Absent	
PPK-LIKE6	Absent	
PPK-LIKE7-FX	Absent	
PPK-LIKE8	RPRC014276	
PPK-LIKE9	RPRC000099	
PPK-LIKE10-FX	RPRC000048	
**Ammonium transporters**		
Ammonium Transporter 1-FX	RPRC006389	
Ammonium Transporter 3-FX	RPRC014483	Former annotation RPRC004093
Ammonium Transporter Rh50 like-FX	RPRC007650	Former annotation RPRC007228
**Potential odorant degrading enzymes**		
Secreted Esterases belonging to detox/ pheromone hormone processing class		
	RPRC012523	
	RPRC003733	
	RPRC014623	
	RPRC010263	
	RPRC003566	
	RPRC010259	
	RPRC010265	
	RPRC014627	
	RPRC004681	
	RPRC003620	
CYP4 clade from CPY450s		
CYP4g	RPRC003227	
CYP4P	RPRC007300	
CYP4GG	RPRC013847	
CYP4u	RPRC013652	

CYP4E	RPRC004731
CYP4D	RPRC004769
CYP4a	RPRC000027
CYP4Y	RPRC007947
CYP4H	RPRC004733
CYP3093A2	RPRC005136
CYP4F	RPRC004766
CYP4EM3	RPRC000196
CYP4EM6	RPRC004758
CYP4T	RPRC007952
CYP4V	RPRC002974
CYP417a	RPRC000172
CYP4c	RPRC000277
CYP3093A7	RPRC002132
CYP4l	RPRC013674
CYP3097A9	RPRC003225
CYP4C75	RPRC015287
CYP3093A8	RPRC005184

Table S4- Reference and target genes used to validate RNA-Seq data by means of qRT-PCR, their primer sequences, amplicon and intron lengths (base pairs-bp), squared correlation coefficient (R), and qRT-PCR efficiency (E).

Gene	Primer sequence (5'to 3')	Amplicon length (bp)	Intron length (bp)	R^2	E (%)
G6PDH	For- AGCCTGGAGAAGCGGTTTACGTTA / Rev- GTGAGCCACAGAATACGTCGAGT	162	923	0.994	103.9
Tub	For- TGTGCCCAAGGATGTGAACG / Rev- CACAGTGGGTGGTTGGTAGTTGAT	118	202	0.999	100
Orco	For- GATCTGCACTGTTGCTGCAC / Rev- CCATGGATGCAGAACACAAA	157	Exon/Intron junction	0.988	97.1
Ir8a	For- TGCAGTCCAACAAGGTAGTCAC / Rev- GCGTAATGCCTTCATCTTCGTCA	155	295	0.993	105.8
Ir25a	For- AAGATGTGGCAGGCAATGAAGG / Rev- CTGTTGCATCACCAAGGAAAGC	118	732	0.997	89.7
Ir76b	For- GCGTTTGCGTACCAAATGGACA / Rev- GCGTCCGGTAGATCCAAAGTGATT	113	1,055	0.981	77
Or13	For- GCTGGTGACTGTTTGATGGG / Rev- CGAGCCGTAAAATTCCAGATCC	195	298	0.98	113,5
Or18	For- CAAGTTGGCCTGATCGTGTC / Rev- CTTCTCGGAGGTTTTCGCTG	115	Exon/Intron junction	0.989	97.7
Or20	For- GTGACGGGAAGATTACGACG / Rev- AGTAATTCGCTCGCATCACT	115	Exon/Intron junction	0.994	97.4
Or53	For- TTGTACGACTGCCCTTGGTA / Rev- ATGTAGGAATACGCAGCTTGT	169	377	0.979	87.1
Ir93a	For- TTGCCATCACTGGAGATCAATAGG / Rev- GGTTGGATCTACCAGTCCAACAG	105	192	0.991	91.5
Ir21a	For- TGGTAAGTCTTGCATTTCCTCC/ Rev- GCCAGTTGATCTTTCCTGCA	164	Exon/Intron junction	0.982	107.5
Ir75e	For- CACCGTCACGGAAGTTGAACTA / Rev- CTGTGCACCATCTATTCCAGTCA	193	270	0,995	95,1
Gr26	For- TTGAAGTTGACTGCATTCGG/ Rev- CTTTCCGTTCACTGGCATTG	124	350	0.986	107.9
TRPA1	For- ACTTTAGATTCTCAAGGACAGCT/ Rev- GCCAGGTACACTTTGCAGTT	117	1,748	0.993	84.8
Iav	For- TAAACCAAACAGAGTCGCCC / Rev- TAATGGGTCTGGTGGTGAGT	106	Intro-Exon junction	0.988	110
Piezo	For- TGCGGTGATCCAGTTTATGAA / Rev- AGTGTACAGTCCAATGATCCCT	144	Intro-Exon junction	0.943	130
foraging	For- AGTTCGAAGGTCTGCGACTG / Rev- GCCCATGATCTCCTTCTCGG	191	3,064	0.997	107.7
period	For- CTACAGCAGCCCAAAAGAAGA / Rev- GTAAGTAGCCGACGGACCAG	105	1,054	0.996	103.9
pp2a	For- GCATCCGATTCAGTTCCCAA / Rev- CCATCGCCTCAGAAGCAAAA	166	4,650	0.997	90.2
Ogt	For- TGCTGCTTTGAAGAACAATTCC / Rev- CATGGATTCCTATGAACAAATCG	119	601	0.975	120
Cryptochrome	For- AGAAATCCTGAGGCTCTTGC / Rev- TGCGTGTCTTGCCAAATG	117	956	0.999	86.2
timeless	For- ACATGCACATGGCTGGAG / Rev- ATTAACAACATCGCCCACAG	97	723	0.996	109.6
Saggy	For- GTATTGGGAACACCGACCAG / Rev- CTCGCTCGGAATACCTTCTG	110	4,903	0.994	104.9
CKII-beta	For- CAGTTCGTTCCAAGGTTGTATG / Rev- TCATCGCTCGTAAAGGTGTC	100	714	0.985	99.5
Nocte	For- TATCCACCACAAGGCAACC / Rev- TTTGATGCTGAAGTGTTGTTCTC	101	1,265	0.997	110.75
Takeout	For- GCTTTAGGCAACACCATGAAC / Rev- GCCAAAGCTGGAGACAATTC	77	1,339	0.982	121.5

Table S5 – Transcript abundances for 25 validation genes obtained using qRT-PCR and RNA-Seq techniques.

	Larvae *vs.* Female		Larvae *vs.* Male		Male *vs.* Female	
Gene	Transcript abundance by qPCR	Transcript abundance by RNAseq	Transcript abundance by qPCR	Transcript abundance by RNAseq	Transcript abundance by qPCR	Transcript abundance by RNAseq
CkIIb	0.85	0.57	0.72	0.34	0.85	0.60
Cryp	0.95	1.72	1.30	1.20	1.37	0.70
For	0.57	0.49	0.74	0.33	1.30	0.67
Gr26	0.49	0.81	0.52	0.46	1.06	0.57
Iav	1.21	0.71	1.09	0.76	0.90	1.07
Ir21a	2.20	5.58	2.70	3.71	1.23	0.67
Ir25a	1.92	2.27	1.79	2.87	0.93	1.27
Ir75c	3.94	8.65	3.21	8.65	0.81	2.51
Ir76b	8.24	1.55	13.04	8.28	1.58	5.33
Ir8a	4.52	2.58	6.31	13.56	1.40	5.25
Ir93a	3.08	2.31	3.83	2.04	1.24	0.88
Nocte	1.37	0.70	1.17	0.63	0.85	0.90
Ogt	0.80	1.12	1.00	0.78	1.26	0.70
Or13	6.30	26.06	5.42	14.48	0.86	0.56
Or18	11.97	25.33	12.06	11.95	1.01	0.47
Or20	3.00	9.55	2.82	6.78	0.94	0.71
Or53	3.25	18.27	3.09	13.39	0.95	0.73
Orco	5.43	11.70	5.36	18.03	0.99	1.54
Per	0.56	0.61	0.85	0.43	1.52	0.69
Piezo	2.40	1.57	3.85	2.30	1.61	1.47
Pp2a	0.94	0.54	1.07	0.43	1.14	0.79
Ssg	2.30	1.08	2.03	1.66	0.88	1.54
Takeout	0.21	0.12	0.18	0.06	0.85	0.50
Timeless	3.12	1.55	7.67	1.73	2.46	1.12
Trpa1	0.76	1.14	0.99	1.16	1.31	1.01

Table S6 – FPKM fold change of ORs and IRs in larval antennae compared to male and female adult antennae; and FPKM fold change of IRs in male compared to female antennae. Genes shown here with red values were not included in Supplementary Figs. S3 and S4. OR pseudogenes (Or6, Or10, Or11, Or15 and Or35) were not included in Supplementary Fig. S3.

Gene	FPKM larvae	FPKM female	FPKM male	FPKM fold change larvae *vs* female	FPKM fold change larvae *vs* male
**Or42**	-	1.14	2.19	-	-
**Or59**	-	0.14	0.00	-	-
**Or2**	-	0.00	0.14	-	-
**Or109**	-	0.25	1.03	-	-
**Or24**	-	0.12	0.43	-	-
**Or90**	0.02	0.23	0.16	9.20	6.71
**Or50**	0.05	2.72	11.65	55.16	236.44
**Or56**	0.05	9.06	8.34	181.57	167.15
**Or91**	0.10	0.00	0.00	0.00	0.00
**Or21**	0.11	0.13	0.16	1.19	1.42
**Or93**	0.12	2.35	2.29	20.23	19.70
**Or62**	0.16	0.24	5.08	1.49	30.85
**Or75**	0.17	0.58	0.55	3.52	3.29
**Or31**	0.17	0.91	1.75	5.32	10.25
**Or29**	0.18	0.39	0.17	2.23	0.98
**Or88**	0.19	1.58	1.70	8.15	8.74
**Or41**	0.21	2.53	3.03	11.93	14.29
**Or63**	0.22	1.77	1.20	8.21	5.55
**Or85**	0.22	0.70	1.08	3.22	4.94
**Or80**	0.22	3.06	3.09	13.72	13.89
**Or68**	0.24	2.34	2.00	9.87	8.42
**Or52**	0.24	0.71	0.62	2.96	2.60
**Or16**	0.26	0.78	0.82	2.98	3.13
**Or69**	0.28	0.91	0.50	3.27	1.81
**Or60**	0.30	0.88	0.28	2.94	0.92
**Or86**	0.33	2.42	2.86	7.40	8.71
**Or108**	0.33	1.86	1.25	5.58	3.77
**Or84**	0.34	4.30	8.36	12.60	24.47
**Or114**	0.34	0.73	1.40	2.12	4.08
**Or23**	0.38	0.38	0.69	0.99	1.82
**Or65**	0.40	1.62	0.91	4.09	2.30
**Or49**	0.40	2.46	1.96	6.15	4.90
**Or46**	0.41	6.27	10.10	15.37	24.75
**Or38**	0.41	0.90	1.15	2.19	2.80
**Or104**	0.43	2.05	1.00	4.76	2.33
**Or81**	0.43	1.20	0.86	2.78	1.98
**Or3**	0.47	5.40	3.77	11.61	8.10

Gene	FPKM larvae	FPKM female	FPKM male	FPKM fold change larvae *vs* female	FPKM fold change larvae *vs* male
**Or22**	0.50	0.70	0.79	1.39	1.57
**Or54**	0.51	9.99	6.27	19.54	12.26
**Or107**	0.52	1.97	1.78	3.81	3.45
**Or43**	0.56	1.48	1.29	2.62	2.28
**Or73**	0.57	0.80	0.69	1.40	1.21
**Or106**	0.58	4.01	4.25	6.91	7.31
**Or111**	0.65	3.56	3.38	5.47	5.19
**Or5**	0.68	4.61	4.65	6.81	6.86
**Or7**	0.69	2.16	2.55	3.12	3.68
**Or82**	0.70	1.79	1.15	2.58	1.65
**Or95**	0.70	0.78	1.55	1.11	2.20
**Or64**	0.72	2.56	1.58	3.55	2.18
**Or113**	0.73	2.87	4.00	3.95	5.51
**Or74**	0.74	9.38	7.86	12.76	10.70
**Or92**	0.75	3.08	3.67	4.11	4.89
**Or1**	0.75	4.76	4.16	6.31	5.51
**Or44**	0.76	8.86	7.33	11.73	9.71
**Or17**	0.76	0.00	0.05	0.00	0.06
**Or101**	0.78	9.76	9.39	12.55	12.06
**Or48**	0.78	3.45	4.29	4.41	5.50
**Or67**	0.84	7.64	4.26	9.14	5.10
**Or40**	0.84	12.23	8.04	14.56	9.57
**Or55**	0.90	6.78	5.12	7.57	5.72
**Or33**	0.94	12.51	6.59	13.37	7.04
**Or26**	0.94	5.13	6.83	5.45	7.26
**Or27**	0.95	7.10	3.60	7.49	3.80
**Or37**	0.97	10.37	8.14	10.69	8.40
**Or70**	0.98	2.57	3.15	2.61	3.20
**Or89**	0.99	8.50	8.12	8.61	8.23
**Or79**	1.01	5.23	8.48	5.17	8.38
**Or98**	1.04	8.91	8.26	8.57	7.95
**Or25**	1.04	2.73	2.02	2.63	1.94
**Or32**	1.04	6.27	4.00	6.02	3.84
**Or115**	1.07	3.07	1.71	2.88	1.61
**Or99**	1.08	5.64	5.70	5.23	5.29
**Or13**	1.14	29.68	16.50	26.06	14.48
**Or30**	1.17	2.51	1.91	2.15	1.63
**Or8**	1.23	5.47	2.69	4.45	2.19
**Or78**	1.24	25.01	20.76	20.12	16.70
**Or39**	1.28	16.00	22.73	12.46	17.70
**Or28**	1.29	3.48	2.76	2.70	2.14
**Or102**	1.30	11.72	13.19	9.03	10.17

Gene	FPKM larvae	FPKM female	FPKM male	FPKM fold change larvae *vs* female	FPKM fold change larvae *vs* male
**Or96**	1.35	11.06	9.81	8.17	7.24
**Or77**	1.41	15.87	15.28	11.23	10.81
**Or36**	1.42	12.77	11.77	8.99	8.28
**Or110**	1.52	19.37	17.70	12.74	11.64
**Or19**	1.52	7.19	5.35	4.72	3.51
**Or47**	1.53	4.76	11.38	3.12	7.46
**Or83**	1.56	18.24	13.25	11.69	8.50
**Or97**	1.60	3.20	4.42	2.00	2.77
**Or51**	1.65	2.36	3.71	1.43	2.25
**Or94**	1.82	5.21	4.89	2.87	2.70
**Or34**	2.07	11.54	7.99	5.57	3.86
**Or45**	2.15	9.59	10.65	4.46	4.96
**Or71**	2.41	6.94	3.82	2.88	1.59
**Or112**	2.43	11.99	7.42	4.93	3.05
**Or4**	2.50	4.52	3.75	1.81	1.50
**Or12**	2.55	3.58	3.06	1.40	1.20
**Or53**	2.86	52.25	38.29	18.27	13.39
**Or9**	2.86	3.03	2.78	1.06	0.97
**Or103**	2.94	21.25	13.60	7.24	4.63
**Or87**	3.20	5.96	8.23	1.86	2.57
**Or58**	3.70	51.18	28.96	13.84	7.83
**Or72**	3.91	6.88	7.46	1.76	1.91
**Or61**	4.32	49.35	24.02	11.42	5.56
**Or20**	4.44	42.37	30.07	9.55	6.78
**Or18**	5.47	138.48	65.37	25.33	11.95
**Or100**	6.10	32.55	31.62	5.33	5.18
**Or105**	7.91	24.41	23.17	3.09	2.93
**Or57**	9.69	8.44	9.81	0.87	1.01
**Or14**	12.48	12.86	6.33	1.03	0.51
**Orco**	12.95	151.56	233.53	11.70	18.03

Gene	FPKM larvae	FPKM female	FPKM male	FPKM fold change larvae *vs* female	FPKM fold change larvae *vs* male	FPKM fold change male *vs* female
**Ir101**	0.00	0.00	0.00	-	-	-
**Ir104**	0.06	0.68	0.27	11.67	4.73	0.40
**Ir102**	0.10	0.23	0.17	2.38	1.77	0.74
**Ir75k**	0.17	0.27	1.46	1.61	8.55	5.33
**Ir103**	0.19	0.42	0.34	2.18	1.73	0.80
**Ir75f**	0.22	0.39	1.61	1.76	7.35	4.16
**Ir41a**	0.26	0.09	1.43	0.36	5.50	15.24
**Ir75p**	0.29	0.36	1.55	1.23	5.38	4.37
**Ir75j**	0.33	0.51	1.82	1.53	5.47	3.58
**Ir75l**	0.33	2.40	3.18	7.19	9.52	1.32
**Ir75e**	0.52	1.86	4.57	3.55	8.71	2.45
**Ir105**	0.53	1.23	0.78	2.31	1.45	0.63
**Ir75d**	0.58	1.58	3.67	2.74	6.36	2.32
**Ir75m**	0.69	2.99	2.35	4.34	3.40	0.78
**Ir75n**	0.71	0.61	3.52	0.86	4.93	5.73
**Ir75i**	0.76	0.74	3.82	0.98	5.03	5.16
**Ir8a**	0.96	2.49	13.06	2.58	13.56	5.25
**Ir76b**	1.12	1.75	9.31	1.55	8.28	5.33
**Ir75g**	1.30	2.69	2.80	2.07	2.15	1.04
**Ir68a**	1.37	3.18	4.65	2.32	3.39	1.46
**Ir75b**	1.49	2.02	5.63	1.36	3.77	2.78
**Ir40**	1.56	3.02	3.50	1.93	2.24	1.16
**Ir75h**	1.62	9.50	7.94	5.86	4.90	0.84
**Ir107**	1.71	1.02	0.66	0.59	0.38	0.65
**Ir75o**	2.15	3.07	3.29	1.42	1.53	1.07
**Ir21a**	3.34	18.64	12.40	5.58	3.71	0.67
**Ir106**	3.85	6.26	4.83	1.63	1.26	0.77
**Ir25a**	4.53	10.29	13.03	2.27	2.87	1.27
**Ir41b**	5.55	4.07	9.01	0.73	1.62	2.21
**Ir41c**	5.58	8.66	13.25	1.55	2.38	1.53
**Ir93a**	6.90	15.94	14.06	2.31	2.04	0.88
**Ir75a**	7.52	43.32	38.71	5.76	5.14	0.89
**Ir75c**	8.89	6.32	16.01	0.71	1.80	2.53

Table S7 – Statistical results of differentially expressed sensory genes among studied stages. Comparison of normalized counts per million (CPM) among stages was conducted using edgeR package. LogFC: Log fold change; FDR adjusted p-value: false discovery rate. Asterisks indicate differentially expressed genes which had increased expression both in female and male libraries when compared to the larval on. Grey rows indicate those differentially expressed sensory genes with an FDR adjusted p-value <0.1 and ≥0.05.

VectorBase Code	Gene name	LogFC	P Value	FDR
Female *vs* Larvae				
RPRC011935	*Csp11*	4.6177639	2.03E-05	0.00543423
RPRC004413	*Obp6*	3.7337096	0.00033137	0.04369921
RPRC000492*	*Obp12*	4.1821106	8.20E-05	0.01432273
RPRC001924	*Obp18*	3.8870436	0.00020651	0.03026726
RPRC000257	*Obp25*	3.706511	0.00036841	0.0471979
RPRC000362*	*Or13*	4.7661175	1.60E-05	0.00471476
RPRC000616	*Or18*	4.5953717	2.27E-05	0.00585537
RPRC000235	*Or33*	3.9548902	0.00021066	0.03061596
RPRC000229	*Or40*	3.7800159	0.00037197	0.04730301
RPRC000108*	*Or42*	5.5382956	0.00030923	0.04113884
RPRC001623*	*Or50*	5.9073117	1.89E-05	0.0052805
RPRC002122*	*Or53*	4.1931722	8.71E-05	0.01488973
RPRC000055	*Or54*	4.0638092	0.00017001	0.0255673
RPRC002162*	*Or56*	7.7940823	3.71E-08	2.67E-05
RPRC000526	*Or58*	3.8404857	0.00025645	0.03618896
RPRC000733*	*Or78*	4.3605515	5.78E-05	0.01063686
RPRC013907*	*Snmp1a*	4.141709	9.35E-05	0.01555242
RPRC011926	*Csp13*	3,5668544	0,00055353	0,06094844
RPRC000311	*Obp7*	3,3984865	0,00091251	0,08134937
RPRC000177	*Obp13*	3,5636428	0,0005568	0,06094844
RPRC000118	*Obp17*	3,4025256	0,00090084	0,08078139
RPRC000476*	*Orco*	3,6257646	0,00047016	0,05494155
RPRC001628*	*Or17*	-5.11298295	0.00054051	0.0607387
RPRC000337	*Or36*	3.4458146	0.00103135	0.08918574
RPRC000041*	*Or39*	3.5437982	0.00070891	0.06926917
RPRC001291	*Or44*	3.5770097	0.00068945	0.06852904
RPRC001292*	*Or46*	3.5556707	0.00094406	0.08260624
RPRC000208	*Or61*	3.5621056	0.00058908	0.06259194
RPRC000200*	*Or74*	3.6689233	0.00057348	0.06160492
RPRC000468*	*Or77*	3.5871911	0.00061546	0.06373893
RPRC006981	*Or83*	3.6104178	0.00056403	0.06096787
RPRC000431*	*Or84*	3.8578002	0.00056242	0.06096787
RPRC002158*	*Or93*	3.9639634	0.00050038	0.0576933
RPRC000188*	*Or101*	3.6816444	0.00055654	0.06094844
RPRC002141*	*Or110*	3.6246434	0.00054084	0.0607387

VectorBase Code	Gene name	LogFC	P Value	FDR
		Male *vs* Larvae		
RPRC000492*	*Obp12*	3.8224689	0.00025253	0.02260681
RPRC000362*	*Or13*	3.9130394	0.00023271	0.02096184
RPRC000041	*Or39*	3.9666731	0.00019725	0.01916505
RPRC000108*	*Or42*	6.4892913	1.38E-05	0.00220318
RPRC001292	*Or46*	4.2496605	0.00011301	0.01229268
RPRC001623*	*Or50*	8.0103594	1.47E-08	6.53E-06
RPRC002122*	*Or53*	3.6938235	0.00041309	0.0336999
RPRC002162*	*Or56*	7.5779548	6.41E-08	2.17E-05
RPRC002135	*Or62*	5.3271478	1.40E-05	0.00221892
RPRC000733*	*Or78*	3.9627857	0.00019926	0.01919703
RPRC000431	*Or84*	4.7891779	2.62E-05	0.00370926
RPRC001799	*Or109*	8.8306133	2.82E-05	0.00393288
RPRC000476	*Orco*	4.1239946	0.00010028	0.01118905
RPRC013907*	*Snmp1a*	4.1743311	8.44E-05	0.00979137
RPRC011935*	*Csp11*	3.491218	0.00069117	0.0515252
RPRC000349	*Ir8a*	3.3822348	0.00104076	0.07171294
RPRC001628*	*Or17*	4.30274198	0.00132372	0.0857445
RPRC000616*	*Or18*	3.4190401	0.00088322	0.06338294
RPRC001980	*Or24*	7.7232531	0.00077626	0.05640957
RPRC000146	*Or41*	3.5572204	0.00115111	0.07807236
RPRC000200*	*Or74*	3.4363402	0.00109663	0.07496511
RPRC000468*	*Or77*	3.3209318	0.00138631	0.08880085
RPRC000441	*Or80*	3.5196305	0.0013427	0.08664938
RPRC002158*	*Or93*	3.7467987	0.00118704	0.07896118
RPRC000188*	*Or101*	3.5199762	0.00084971	0.06123249
RPRC002141*	*Or110*	3.4290217	0.00095651	0.06617165
		Male *vs* Female		
RPRC002135	*Or62*	4.50093249	9.30E-05	0.03496851

Table S8 – Number of reads mapped on sensory receptor genes in the three antennal libraries obtained by HTseq.

VectorBase Code	Gene annotation	Larval reads	Female reads	Male reads
RPRC004662	CHEB1	648	1,107	717
RPRC004663	CHEB2	359	700	332
RPRC004664	CHEB3	294	561	404
RPRC004665	CHEB4	205	613	323
RPRC004666	CHEB5	1,331	1,593	836
RPRC006538	chloride channel B1	717	359	876
RPRC013530	chloride channel B2	327	191	368
RPRC013223	CSP1	52,642	47,151	19,488
RPRC007979	CSP10	3,476	21,241	14,236
RPRC011935	CSP11	117,101	2,401,988	1,193,677
RPRC011928	CSP12	25,225	1,82,376	105,675
RPRC011926	CSP13	1,288	12,753	9,664
RPRC006791	CSP14	8	26	12
RPRC000546	CSP15	29	44	28
RPRC000327	CSP16	2,599	6,553	5,378
RPRC007134	CSP17	23,677	18,964	11,872
RPRC000242	CSP18	0	1	0
RPRC000126	CSP19	587	708	604
RPRC013221	CSP2	22,437	16,083	10,290
RPRC013280	CSP3	319,363	375,650	191,485
RPRC013285	CSP4	360	1,009	1,699
RPRC013218	CSP5	89,857	98,833	43,414
RPRC000332	CSP6	338	490	334
RPRC010107	CSP7	361	584	443
RPRC000631	CSP8	949	909	564
RPRC000470	CSP9	96	382	340
RPRC001549	GR1	116	686	465
RPRC001194	GR11	42	35	55
RPRC002133	GR13	0	3	9
RPRC008717	GR14	0	0	0
RPRC001115	GR15	10	21	26
RPRC000730	GR16	10	6	2
RPRC001279	GR17	12	5	7
RPRC000336	GR18	1	0	0
RPRC000763	GR19	0	3	1
RPRC002023	GR2	269	385	443
RPRC000451	GR20	62	122	323
RPRC000130	GR21	26	40	33
RPRC000073	GR22	25	19	120
RPRC001956	GR24	30	112	180
RPRC000216	GR25	95	123	89

VectorBase Code	Gene annotation	Larval reads	Female reads	Male reads
RPRC000368	GR26	964	695	390
RPRC000068	GR27	937	1,021	825
RPRC000290	GR28	3,281	1,836	1,467
RPRC001795	GR3	72	20	33
RPRC000056	GR4	2	6	6
RPRC001197	GR5	2	1	7
RPRC000553	GR6	4	4	16
RPRC001196	GR7	44	11	17
RPRC001195	GR8	18	9	32
RPRC000388	GR9	13	23	60
RPRC005659	IR101	0	0	0
RPRC000847	IR102	10	14	12
RPRC000379	IR103	52	107	100
RPRC002996	IR104	4	40	17
RPRC000538	IR105	48	82	57
RPRC000846	IR106	299	421	345
RPRC000385	IR107	132	71	49
RPRC001826	IR21a	324	1,599	1,186
RPRC000589	IR25a	605	1,128	1,501
RPRC000383	IR40a	129	221	308
RPRC000191	IR41a	21	6	112
RPRC000489	IR41b	584	368	764
RPRC013097	IR41c	330	454	703
RPRC003280	IR68a	1	0	0
RPRC002112	IR75a	2,440	3,050	2,827
RPRC000105	IR75d	48	103	255
RPRC000047	IR75e	40	125	333
RPRC002124	IR75f	101	134	642
RPRC000214	IR75k	1,451	1,795	2,647
RPRC000247	IR75m	61	209	198
RPRC000007	IR75n	66	45	270
RPRC000469	IR76b	71	94	507
RPRC000349	IR8a	148	259	1,400
RPRC008486	IR93a	1,420	1,882	1,689
RPRC014507	mrityu	683	280	783
RPRC007564	narrowabdomen	1,291	507	1,452
RPRC000296	NMDA1	804	371	466
RPRC006099	NMDA2a	3	0	12
RPRC001831	NMDA2b	19	8	23
RPRC000544	NOMPC	196	112	192
RPRC010284	OBP1	27,221	41,610	23,759
RPRC000560	OBP10	294	362	382
RPRC004408	OBP11	3,176	10,026	12,463
RPRC000492	OBP12	4,506	68,339	57,789

VectorBase Code	Gene annotation	Larval reads	Female reads	Male reads
RPRC000177	OBP13	4,057	40,078	30,127
RPRC004407	OBP14	91,134	84,408	44,892
RPRC008413	OBP15	41	58	79
RPRC011610	OBP16	1425	1702	1040
RPRC000118	OBP17	26,650	235,443	163,756
RPRC001924	OBP18	44,544	550,594	333,946
RPRC000593	OBP19	17	81	62
RPRC007008	OBP20	70,257	361,259	266,432
RPRC001925	OBP21	7,251	58,737	29,346
RPRC000115	OBP22	14,745	34,244	17,398
RPRC000174	OBP23	25,498	39,280	21,922
RPRC009905	OBP24	191,366	1,340,736	742,932
RPRC000257	OBP25	508	5,542	3,022
RPRC000182	OBP26	8	0	1
RPRC009914	OBP27	14	2	0
RPRC000527	OBP3	366	2,315	1,533
RPRC000274	OBP4	144	154	103
RPRC004413	OBP6	37,479	416,555	268,638
RPRC000311	OBP7	6,460	56,913	46,034
RPRC000564	OBP8	20,309	30,048	14,713
RPRC000194	OBP9	6	22	9
RPRC002158	Or 93	9	119	111
RPRC000579	Or1	36	191	191
RPRC000106	Or10	5	6	17
RPRC000522	Or100	344	1,505	1,632
RPRC000188	Or101	37	398	386
jose.estivalis020	Or102	78	495	588
RPRC000601	Or103	145	777	523
RPRC009806	Or104	18	89	45
RPRC000255	Or105	441	1,036	1,032
RPRC000371	Or106	35	206	212
RPRC001726	Or107	28	89	85
RPRC000301	Or108	18	76	59
RPRC001799	Or109	0	11	54
RPRC002141	Or110	81	836	792
RPRC002142	Or111	31	164	153
RPRC000555	Or112	148	603	374
RPRC001309	Or113	39	116	177
RPRC004306	Or114	0	0	0
jose.estivalis025	Or115	53	103	55
RPRC000376	Or12	130	144	135
RPRC002134	Or12	0	4	8
RPRC000362	Or13	60	1367	821
RPRC000166	Or14	534	379	348

VectorBase Code	Gene annotation	Larval reads	Female reads	Male reads
RPRC000420	Or16	19	34	42
RPRC001628	Or17	46	1	2
RPRC000616	Or18	323	6,526	3,133
RPRC000449	Or19	80	303	238
RPRC001689	Or2	1	0	8
RPRC000573	Or20	258	1,967	1,523
RPRC006570	Or21	4	4	5
RPRC000375	Or22	27	33	35
RPRC000149	Or23	23	16	37
RPRC001980	Or24	0	6	25
RPRC000029	Or25	63	151	99
RPRC000619	Or26	48	232	317
RPRC000331	Or27	42	279	157
RPRC000125	Or29	10	16	10
RPRC000059	Or3	38	257	197
RPRC000209	Or30	359	209	193
RPRC000423	Or31	9	45	83
RPRC000043	Or32	64	276	200
RPRC000235	Or33	60	779	383
jose.estivalis045	Or34	64	305	223
RPRC000337	Or36	47	429	361
RPRC000456	Or37	75	544	522
RPRC000530	Or38	23	44	56
RPRC000041	Or39	71	693	1,008
RPRC000120	Or4	157	236	203
RPRC000229	Or40	41	472	315
RPRC000146	Or41	13	112	140
RPRC000108	Or42	1	44	92
RPRC000140	Or43	33	58	59
RPRC001291	Or44	42	420	351
jose.estivalis006	Or45	130	312	410
RPRC001292	Or46	24	237	416
RPRC000413	Or47	82	204	512
RPRC001293	Or48	43	174	208
RPRC000433	Or49	18	99	84
RPRC000083	Or5	8	43	51
RPRC001623	Or50	2	107	498
RPRC000507	Or51	120	147	201
RPRC000201	Or52	10	24	23
RPRC002122	Or53	124	1,897	1,456
RPRC000055	Or54	28	393	248
RPRC002161	Or55	44	281	212
RPRC002162	Or56	2	396	369
RPRC000318	Or57	668	380	422

VectorBase Code	Gene annotation	Larval reads	Female reads	Male reads
RPRC000526	Or58	173	2,072	1,171
RPRC004587	Or59	0	0	0
RPRC002105	Or60	11	37	19
RPRC000208	Or61	191	1,886	931
RPRC002135	Or62	6	10	223
RPRC002136	Or63	9	75	51
RPRC000095	Or64	35	95	61
RPRC000132	Or65	23	63	35
RPRC000452	Or66	63	156	110
RPRC000222	Or67	48	296	179
RPRC000541	Or68	12	82	77
jose.estivalis012	Or69	11	36	23
RPRC002152	Or7	35	99	118
RPRC000459	Or70	46	91	127
RPRC000490	Or71	99	249	135
RPRC000053	Or72	160	249	297
RPRC000585	Or73	21	26	23
RPRC000200	Or74	33	352	325
RPRC000245	Or75	7	22	18
RPRC001928	Or76	26	133	147
RPRC000468	Or77	67	674	608
RPRC000733	Or78	54	929	765
RPRC000202	Or79	42	199	332
RPRC001154	Or8	89	326	174
RPRC000441	Or80	12	119	126
RPRC000363	Or81	20	50	37
RPRC002113	Or82	29	67	40
RPRC006981	Or83	82	838	639
RPRC000431	Or84	13	159	329
RPRC000882	Or85	8	24	36
RPRC000079	Or86	14	100	106
RPRC000317	Or87	138	220	313
RPRC000607	Or88	8	60	64
RPRC000165	Or89	51	340	332
RPRC000895	Or9	94	83	81
RPRC000249	Or90	1	8	7
RPRC000093	Or91	5	0	0
RPRC002009	Or92	41	144	168
RPRC002156	Or94	111	264	267
RPRC002157	Or95	43	38	95
RPRC001767	Or96	71	487	448
RPRC002120	Or97	89	146	223
RPRC000136	Or98	63	403	392
RPRC000065	Or99	64	260	269

VectorBase Code	Gene annotation	Larval reads	Female reads	Male reads
RPRC000476	Orco	689	7,107	10,892
RPRC014024	piezo	4,104	5,643	8,555
RPRC014320	PKD	0	0	0
RPRC000048	ppk-like10	2,063	2,468	1,681
RPRC014276	ppk-like8	38	38	44
RPRC013510	ppk-like1	191	214	151
RPRC000471	ppk-like2	78	106	98
RPRC000341	ppk-like4	251	157	208
RPRC000099	ppk-like9	344	346	283
RPRC013907	SNMP1a	1,205	17,772	19,724
RPRC013910	SNMP1b	906	5,231	4,895
RPRC002754	SNMP2	10,787	5,349	8,431
RPRC001596	TRPA5a	389	222	227
RPRC001597	TRPA5b	1,392	1,598	1,701
RPRC000350	TRPGamma	1,033	478	691
RPRC000224	TRPL	82	63	68
RPRC007108	TRPM	168	162	480
RPRC000127	WTRW	5,690	2,763	2,097
RPRC002111	IAV	1,545	973	1,052
RPRC000454	PAIN	8,150	6,240	5,928
RPRC000422	TRPA1	4	3	8
RPRC000570	TRPA5C	3	1	2
jose.estivalisGR14a	Gr14a	2	3	3
jose.estivalisGR14b	Gr14b	0	0	0
jose.estivalisGR23a	Gr23a	1	0	0
jose.estivalisGR23b	Gr23b	0	0	1
jose.estivalisIR75b	Ir75b	452	232	485
jose.estivalisIR75c	Ir75c	518	269	611
jose.estivalisIR75g	Ir75g	103	187	204
jose.estivalisIR75h	Ir75h	136	664	580
jose.estivalisIR75i	Ir75i	61	46	256
jose.estivalisIR75j	Ir75j	27	41	140
jose.estivalisIR75l	Ir75l	33	183	270
jose.estivalisIR75o	Ir75o	188	237	257
jose.estivalisIR75p	Ir75p	24	31	115
jose.estivalisNan	Nanchung	157	50	89
jose.estivalisOr114	Or114	13	17	13
jose.estivalisOr28	Or28	66	151	125
jose.estivalisOr59	Or59	0	0	0
jose.estivalisppk104	ppk-like6	316	211	207
jose.estivalisppk13-like	ppk-like5	409	411	274
jose.estivalisppk3	ppk-like7	242	319	211
jose.estivalisppk3-like	ppk-like3	276	214	222
RPRC009679	TRPML	891	451	897

VectorBase Code	Gene annotation	Larval reads	Female reads	Male reads
RPRC006389	Ammonium transporter 1	1,171	4,622	3,333
RPRC014483	Ammonium transporter 3	41	80	144
RPRC007650	Ammonium transporter Rh50	177	175	178
