# Supplementary material for: The molecular sensory machinery of a Chagas disease vector: expression changes through imaginal moult and sexually dimorphic features
Source: Sci Rep. 2017 Jan 6;7:40049. doi: 10.1038/srep40049 (PMC5216343; doi:10.1038/srep40049)
Supplement: Supplementary DataSet 1 [file srep40049-s2.doc]

>ORCO

ATGCAGAAAGTGAAGATGCATGGTCTCGTAGGAGACCTATGGCCGAATATCAGACTTATGCAGATGACTGGCCATTGGCTGCTGGAGTATCACGAAGAGACAGGAGGCATGGTCAGGCTGATGCGTATGGCCTTCTGTTGGTTTACAACTTTGATCGTTTTTACACAGTTCGCTTTCATCACGTGCTTTCTTATCCTGGAAACCTACGATGCGGATCAGATGGCCGCTGCAACGATCACTACGTTATTCTTTCTTCATTCAGTCACCAAGTTCATGTATTTTGCCCTGAGAAGTAAATACTTCTACAGGACTCTCTCCGCCTGGAATCAGATGAACTCCCATCCATTGTTCGCTGAGTCCAACGCCCGTCATCGAGCAACCGCTCTGGCTCGTATGAGGAAGTTATTGATGATTATCGGATCTGGTACTATCTTTTCTGTATTGGCTTGGACTACCGTAACATTCTTGGATGATCCACACAGACAGATCACTGATCCGGAGAACGCCAATGAAACAATTACTGTCGAAAAACCGATGCTAATGGTAGACGCCTGGTATCCGTGGGATGCCAAATATGGCATGACATACTTTATGTCATTTATATATCAGTTGTATTGGCTATTCATCTCACTGAGTCATGCCAACTTACTAGACATCTTGTTCTGTTCGTTTGTATTATTCGCTTGTGAACAACTGAAACACCTGAAGGAGATCCTACAACCATTAATGGAGTTGTCAGCCGCTTTAGACTCCGTGGTTCCCAACTCGGGTGATCTTTTCAGAGCATCTTCTACAGCGTCAAATATACCACTGATAGGAAACGATGGTAATGAGTTCGACGTACGCGGGATCTATTCCAACCAGAGAGACTTCTCGGGATTCCAGCCGGGTATCGCTGCGTTGCCCAGCAATGGAGGTGGTGTGGGTCCGAACGGGCTGACCAAGAAGCAGGAACTTATGGTCCGTTCGGCTATCAAGTATTGGGTAGAACGTCATAAACACGTGGTCAAATTCGTTACGAGCATTGGTGATTGTTATGGATCTGCACTGTTGCTGCACATGTTGACGAGCACTGTTACCCTCACACTGCTGGCTTATCAAGCGACTAAGATTGAAGGTGTGGATGTATACGCGTCGACGGTCATTGGCTACTTGGTGTACACTCTGGGACAGGTATTTGTGTTCTGCATCCATGGCAACGAACTGATCGAAGAGAGTTCCTCTGTGATGGAAGCCGCCTACAGTTGCCATTGGTACGACGGCTCTGAAGAGGCGAAAACGTTTGTACAAATCGTTTGCCAACAATGTCAAAAATCGTTGACCGTTTCAGGAGCTAAATTCTTCACTGTATCCTTAGATTTGTTTGCTTCAGTACTCGGAGCTGTGGTCACATACTTTATGGTGCTGGTACAATTGAAATAA

>OR1

ATGGAAGAATTTGCTGGGATTGATGTTAAGTATTTCAAGGTTATAGGCCTATGGCAGCTTTTAATGCCTTCACGAAATGGGAAATATGCTGTAGTTTTAAGTGCATTCATTGGTACAATTTTCACGGTACATATCAGCATACAAATGATTAACACTTTTCTCGGTGGTTATGATTTTTCCAAATTTACTGAAAAACTATCAGTAAATCTGACCTGTTTTGAATCAGCTATAAAAATTTTATATTATTGTTGGAATCGTAACAATTTGATGAATTTAATTTCATTGTTTCGTAAAAATTTACTAATTTGCTCAACACATGAGCCAGAAAAGTCTAGGAAAATTCTAACAAATTCAGCCCGATTTGTGAATTTGTGCACTAAAAGTTTTGTATATATGATATTCTGCACAGTTGGCGTATGGAATTCTTTGCCAATTTTGCGCTGCCTAATTAGCGATGATTGTGAATCATGGCATATAATGCCGTCCTGGTATCCGTTCAACACTAAACAAATACCAATGAACATCATTGTCTACAGTTTTGAATTTCTAATTATGAGCTATTGTGCGGCTTTGCTGTATACGGTGAACTGTTTGTTCTCGGCGCTGGCGCTCACAGTGGCCGCCCAATTTCAACTACTCGGCGTTAGTTTTGCCACCATCGAAAGACATGCTGACGATTATGTGGCCAAACTGGAAATGAGTGAAGAGGCTGAACTGGAAAAAAAGAAACATATGTACAGGCTTTTGAGGGAATGTCTGAAGGATCATCAAACTTTGTTAAGATTCACCAAAGCTTTAGAAGAAATGTATAATCCAATGTTTCTATTTCAAATGTTAACAAGCACTTTTACAATCTGTTTGGTTCTTTTACAATTAAACGTACATTTAGCCTCAAGTGAAGATCTACCAATAGCTATGGCTTGTAAATTTATTATGTATCTATTGTTCGGATCTATGGAGTTGCTGGTGTACAGTTGGGGAGGTCAGATAGTCTTTGATCAGTCAGGTGCAGTGCATTGGAATATGTATGAAAGTGGTTGGACCGAAGGATCTTTACAATTTAGAAAATCACTGGTGTTCGCTATGGCAAGAAGTTACACGCCTGCTACTCTAACTGCTGGAAAATTTTACAACGTTAATCTTAACTCTTTTACTCAGGTGATGAAAGCATCATATTCATATTTTACATTTCTACATGGATCTGGCTCGGTGAAATCATCAACTAAATAA

>OR2

ATGGCGATTGCTGGAGAAAATGTGTTCTTCAGACATGTGTACCTATTAAGGTACCTAGGTGTGACTATACCTTCAGAGGGCAATCCAAGAGGCAAGACCATTAGAATCTACTTCTTATTCATTCAGATAATGTTAACCATAAACACAATATTTCAACTTCTAACCATATTCTTACCAGATCATGAACTATCAGACAGTGCAATTTCTTTAGTATTGTTTCTGGTATCGGCCGTCACACAAATCAAACTATTGATAGTGCAAATGAATATATTATCATTAGCTCGTCTTTTTGAGGAACTGGATACGCCAGGAGCTAGCAATAACACGACGGACATGTTACGCATTGTGAATCAGATCAGTCGTATTTATGTGGCTATACTTGTCACCTTGCTAGGCGGCTGGCTGGCATATCCATGGCTGACTGGAAACTTTCAGTTACCCATGCCATATTGGTTACCATTCAAGATAAACAACATTTATAAATATTTAGTTGTCTATAGTTTTGTCAGTATTATTCTCGCCATTGAGTGTTATACGCAGGAAGCTGTGGACACGCTACTGTTGTTGATGGCCGGCCAAATTTGTAGAAGATTGTATCAGATTAGGCTAGCACTGGTAACATTGGGTACTGAGGAACCATTGAGAAAGAGACGATGGAAACGTGATAAACCACCAGTGGATCGTTTAGGATTGTCTGTGGTCAAAGTGGCAGCGCTCAGCAGGGATGAAACACTCTTGAAGGAGTTTATAAGGGAACATGTCGACATTATAAGATTAATAAGAACATTCGACGTCATATTAGATGATATATTTCTTGCACAAGTTATGCAATCGACCATAGTGTCTACCATGTACTTTTTTCTAGCATCTAAATCACGAGATCTGGTGAAAGATTTTCCGAGATTGTTTGTGGTTCTCGTAGCCACTTATATACAATTTTATATCTACTGTTGGCTCGGACAGGAAATTACACAACATAGTGACGAAATCCATCGTACATTATATCTAAGTGAATGGTATAAATGTCGTGAAAAGATCCAGAAATCCATGGTTATAATTGAAACTTTTACAAAAACCCATTTTAAACTGGAAGGTGGCCATCTATTTAGTATTAATTTGAGAACATTCGTATTGGTGTTGCGGGAATCGTTTTCCTACTTTATGGTCTTAAGAGCCATATTCAACTAA

>OR3

ATGGATATCTTGCAAAGATTTAAAGATTTCCTCCGTGAATATGATCGAGATAATGATGAAGTTGTGGATAAAACAATCTTCGATGAGTTTAACTATTTATATAGGATAACTTTTTTTTATCCAAATTTGAAAACAATGAAAATTATTGGTCTACAAATTGTTCTTTTCATATTTTATATAAGTTTCATTTTGTTACAAATTTTCTTTACATTTATGGGAGCCATATTTTCTGATGGAGATTTCTTTTTGATGGCACATTCTCTGCATATTAGCTTTATATATTCGTTAATGCCAGTGTTGACTTTCACGGCAATCACTCTACGCTCTCATCTATCTAATATATTCAAAATTATTGGAAACGGATTTTATAAATACGATGAAGTTACATCCAAAGAGGAAATTCAAATCAAATTGAAGTATTTTAAAATCAAAAGAAATTTGAAAATTGGTTTTCCTTTACTTGCTTTTGTCGCTATGTTATTGGCTGTTACAGCAATCCCATTATCAAATGAATACTTTGGATTGGATGATTCAGTCATGATCAATAAGAAAGGTGTTAATTTGTGGTTGCCATTAAATGGTTGGTATCCTTTTGACTCAAGCGAGGGTTTACCATTTTGGATAGCATTCTCTCTGCAAATAATATCCATGATTTTTGTTGTAATTATATATGTAGCAGGTTCAGCCACTTATTTTACCATCGTTTTACATGTTATTGCTCAATACCATATTCTTATATGGAGTATACGTAATATAACTAAAAGAGCAATGGTTAATTATTTGAAAATTAAACCATATCTGCAGGGTAAAAATAGAAGCAGGCCTAATTTCGAGGATATGGAATTCCAAAAACAGATGGTGTTCTGTTTGAAACAAAATATACAACATTATCAACAAATTATCAGGGTCGTTAATCTGTATGCAGAAGTGTTAAAAGTGACTGGATTTTTGGTATTTCTCTGCATTACTTTCATTATCGCCCTTTCTGCATTTATAATAGTAACGGGAACAGCAAGACCAGGAATTGTATGTCTCACATTTGGTATTGCAACAGTAGAAGTAGGCTATATGACAGCCCCTTGTATGCTTGGACAAATGATAATCGATTTGAATGAGCAACTCGGTTTTGAACTATACAATACACCCTGGTACCGTTGCTCTAAAGATTTCAAACAATGTTTGAACATTACCCAAGCTAGGATTGGGTATGGCGTCACAATAAGAACACTGTTTAATTACGCAATGGATATGGACACCTATTGTAAAATGGTGAACGCTTCTTATTCATACTTCAGTCTACTGTTGGCTTTCAAAAATACTTAA

>OR4

ATGGATATCTTGCAAAGATTTAAAGATTTCCTCCGTGAATATGATCGAGATAATGATGAAGTTGTGGATAAAACAATCTTCGATGAGTTTAACTATTTATATAGGATAACTTTTTTTTATTTAAATTTGAAAACAATGAAAATTATTGGTCTACAAATTTTTCTTTTCATATTTTATATAAGTTTCATTTTGTTACAAATTTTCTTTACTTTTATGGGAGCCATATTTTCTGATGGAGATTTCTTTTTGATGGCACATTCTCTGCATGTTAGCTTTATATATTTGATAATGGCAATGTTGACTTTCATGGCAATCACTCTACGCTCTCATCTATCTAATATATACAAAATTATTGGAAACGGATTTTATGAATACGATGAAGTTACATCCAAAGAGGAAATTCAAATCAAATTGAAGTATTTTAAAATCAAAAGAAATTTGAAAATTGGTTTTCCTTTACTTGCTTTTGTCGCTATGTTATTGGCTGTTACAGCAATCCCATTATCAAACGAATACTTTGGATTGGAAGATTCAGTCATGATCAATAAGAAAGGTGTTAATTTGTGGTTACCATTAAATGGTTGGTATCCTTTTGACTCAAGTGAGGGTTTACCATTTTGGATAGCATTCTCTCTGCAAATAATATCCATGAGTCTTGTTGTACTTATATATATAGCAGGTTCAGCCACTTATTTTACCATCGTTTTACATGTTATTCCTCAATATCATATACTAATATGGAAAGTACGTAACATAACTAAAAGAGCAATGGTTAATTATTTGAAAATTAAACCATATCTTCAGGGTAAAAACAGACGGAGGCCTAATTTCGAGGATATGGAATTCCAAAAACAGATGGTGTTCTGTTTGAAACAAAATATACAACATTATCAACAAATTATCAGGGTCGTTAATCTGTTTACAGAAGTCTTAAGAGTAACTGGATTTTTTGTATTTCTCGGTATTACTTTTGTTGTAGCCCTTTCTGCATTTATAATAGTAACGGGAACAGCAAGACCAGCGATTGTATGTCTCACATTAGGTATTGCCGCAGCAGAAATAGGCTATATGACAGTCCCTTGTATGCTGGGACAAATGATAATCGATTTGAATGAGCAACTCGGTTTTGAACTATACAATACACCGTGGTATCGATGCTCTAAAGATTTCAAACAATGTCTGAACATTACCCAAGCAAGGATTGGGTACGGCGTCGCAGTAACAACTCTGTTTAGTTACGCAATGGATATGGACACCTATTGCAAATTGGTGAACACTTCATATTCATACTTCAGTCTATTGTTGGCGTTCAAAAGTACTTAA

>OR5FJ

ATGGGTATCTTGCAAAGATTTAAAGATTTCCTCCGTGAATATGATCGAGATAATGATGAAGTTGTGGATAAAACAATCTTCGATGAGTTTAACTATTTATATAGGATAACTTTTTTTTATTTAAATTTGAAAACAATGAGAATTGTTGGACTACAAATTATTCTTTTGATATTTTATCTAAGTTTCATTTTGTTACAAATTTTCTTTACATTTATGGGAGCCATATTTTCTGATGGAGATTTCTTTTTGATGGCACATTCTCTGCATATTAGCTTTATATATTCGTTAATGCCAGTGTTGACTTTCACGGCAATCACTCTACGCTCTCATCTATCTAATATATACAAAATTATTGGAAACGGATTTTATGAATACGATGAAGTTACATCCAAAGAGGGAATTCAAATCAAATTGAAGTATTTTAAAATGAAAAGAAATTTGAAAATTGGTTTTCCTTTACTTGCTTTTGTCGCTATGTTATTGGCTGTTACAGCAATCCCATTATCAAATGAATACTTTGGATTGGATGATTCAGTCATGATCAATAAGAAAGGTGTTAATTTGTGGTTGCCATTAAATGGTTGGTATCCTTTTGACTCAAGCGAGGGTTTACCATTTTGGATAGCATTCTCTCTGCAAGGAATATCCATGATTCTTGTTGCAAATATATATGTAGCAGGTTCAACCACTTATTTTACCATCGTTTTACATGTTATTGTTCAATACCATATACTAATTTGGAAATTACGTAACATAACTAAAAGAGCAATGGTTAATTATTTGAAAATTAAACCATATCTGCAGGGTAAAAATAGAAGCAGGCCTAATTTCGAGGATATGGAATTCCAAAAACAGATGGTGTTCTGTTTGAAACAAAATATACAACATTATCAACAAATTATCAGGGTCGTTAATCTGTATGCGGAAGTGTTAAAAGTGTCTGGATTTATTGTATTTCTCTGCATTACTTTCATTATCGCCCTTTCTGCATTTATAATAGTAACGGGAACAGCAAGACCAGGGATTGTATGTCTCACATTTGGTATTGCAACAGCAGAATTAGGCTATATGACAGCCCTTTGTGTGCTTGGACAAATGATAATCGATTTGAATGAGCAACTCCATTTTGAACTATACAATATACCGTGGTACCGTTGCTCTAAAGATTTCAAACAATGTATGAACATTACCCAAGCTCGGATTGGGTATGGCGTCGCAATAACAACTCTGTTTGATTACGTAATGGATATGGACACCTATTGTAAATTGGTGAACACTTCATATTCATACTTCAGTCTACTGTTGGCGTTCAAAAGTACTTAA

>OR7

ATGCAGAGAATACGCTCTTTAATTCAGCACTTCGACAGATATTCAGATGAAGTTGCAGAGAAATGCATCATAGATGAATATAACCATTTACTAAAAGTTTCATTATTTTATCCAAGTCTGAAATCACCTCTTGCCGCAGGAATCCATTTATTGCAGTTTGTAATTTATGTAGTTACATTGATCTTTCACTTAGCAATTATAGCTATTACAGTTATTAAATCGCAACCCCAAGATTTCTTAATTGTCATTCATACCGTACATTTTGGCTTTATTATGTTTCTCTTCTTATCTTTTATCTTCATGACAAACTCAGTAAGATATGAATTGTGTATAAGTCACAAAATAATAGGCCAGGGTGTATATACGTACGAAGATGAGGAAGTAAGCGATGAAGAGTCGAACATGAAGCGGAAATACGAAGCTATAAAAAGAAAACTTAAACTGATACTGCCCTCTATGAACTTAGTTGCCGGACTTTTCGTCGTAGTGATTGGTCCTTGGGTTGATTCTATACTTGCTTCAGGTAACTTGAAAACCTTCACTGATTCTGGAATTAACTTGGCATTGCCGGTTCCAATATGGCATCCGCTTGACACACATGACAGTTTGAACTTTTACATAGCATTCTATGGTGAATGCATTTGTGCTCATATTGTTATTGTAATCCTGGCATCGGCCAGCTGTTGTTACTTCACTTGCACTTTGCATGTTATCATACAATTGGAACGGCTCATCATGTCTGTTAGGAATCTGGAGAAACGGGCTATTTCCATGTATAGAAGGAGAGTGCCTACTTCCGAAAATCAGTTAAAAAAAGATATTCTTTATGAAAATGCAGCCTACATGGAATGTCTAGAATTTTGTTTCCGCCAGAATATTCAACATCATCTGCAGATTTTAAGGTTTAACAGATTAGTAAATATTTTGACAAGAGGACCCGTTTTCGCTACCTTTTTTGCAGGAGGTGTAATTTTGGGAATGTCCGCTCTAGTGATTACACTTGGGAATGAAAAACCAGGCATTTTGTTGGGTACCCTTTCTGTGGCTATGACAGAACTTGCGAACATTACGGCTATATGCAAAGCTGGACAGACTATCACAGATTTGAACGAAGATTTACACTTGATGTTCTACAGTGTACCATGGCAACGATTTTCAAAGTCTTTGAAATCATCCTTGTACATTGTCCAAGAGATGACTTGCCGAGATATGATTGTGAACGGAATGTTTAACTTCACTGCCAACATGGAGACCTATGGATCTATTTTGAACACAGCATACTCTTGCTTCAGTCTTTTAATTGCATTTAAGAGCGCTGATTAG

>OR8

ATGTCTGCTTACTTCATGCAACGAATACGCTCTCTGTTGCAGCATTACAATAGATATTCAGATGAAGTTACGGAAAAGTTAATCATTAAGGAATATTACCATTTTCTAAAATTGTCACTATTTTATCCAAGTTTAAAATCACCAATTACAGCTGGCATCCATTTATTGCAATTTATTATTTACGTTGTTGTGTTAGGCATACAAAATGTGCTTGGTATTATATCAATTATCAAATCTAATCCGGAAGATTTTTTACTTGTTATGAATATAGGCCAATTCGGTCTTCTTTCAACGCTACTTATGTTGATTATCATCATGTTCAACGCTACAAGATATGAGTTCTGTATGTCGCACAAAATGATAGGCGATGGTGTCTATACTTACGAAGGAGAGGAGGTGCCAAGCGATGATTATTTGCTAATGACGATGAAGTACAAATCTATAAAAAGAAAACTTATGATCACACACCTATGCATCAATTTGAGTGTAGCACCAGTTATTCTTTTGATAAAACCTTGGATGGATGAGAAATATGGCTCAGAGAACTTAAGAACTTATACAAACTCTGGATTTAATACAATTCTCCCGCTTGCAGGATGGTATCCCTTTGACACGCACCAAGGACTGTACTTTTATTTAGCTCTTTTAGAGCAAGCTTTTTGTGGTTATATAATAATAATAATAGTGGCATCGTTTGTAAGTTATTACTTTACTTCAACCATACAAATCATAATTCAGTTAGAACGACTCATCTTGTCCATTAAAAATTTGAATGAAAGGGCTATTTCGTTGTACAAAAAAACAGTGCTTACCGCTTCCACGGACAACTTTAGAGGATGTAAAATTTTTGAAGATGATCCAGTATTCATGGAATGTCTGAAATTTTGTTTCCGACAGAATATCCAACATCATCAACAAATTTTAAGATTAATAAGATTGGTCAATACTGTGTGGAAAGTACCACTGTTCTTATCATTTCTAGCTGAAGGCATTGTTTTGGGAATATCAGCGATGGTAGTGATCTTTGGAGCTGAAAGGCCAGGAGTAGCGTTGGGCACCCTTTCGATTGCTGTTGCTGAACTTGTAAATATCACCACTGTCTGTAAGCTAGCGCAGACCATCACTGATTTAAATGAAAAGCTACACTTAATAACGTATGATATTCCTTGGTATCAATTTTCAAGAAATTTACAACGTTCCGTGTGCATTGTCCAAGAGATGACTTGCCAGGAATTATGCTTGGAAGGATTGTTCAACACGAAAGCCAACATGGAAACTTATTCAAAGACGTTGAACGCAGCCTATTCATTCTTCACTCTTTTGCATTCAAGATCGCTTAAGAAGCAGTCTTATCAGAAAGGATAA

>OR9JOI

ATGGAATTGAAAGAGTGGCTGAAGAAGGAGATAGATCATTATAATAGCGAACACGATGAGATTGTCGATAAGACGATCTACCATGAGTACTTATACCTACCGATACTTGCTTTATTCTATCTAAAAGTAACGCCAAAAGCTCTGATTTTGCATAGCTTACAGCTAATGTTTTATGTAGTCACATTGGCGGTGCATGTGTATTTTGCCTTTATCACAATATTTAAAGAAAAAGATTTTATATTAATTATAAATTTGGCACATTTAATGTACATACTGATGCTAACTATGGTGTTGCCGATCACGATAATAAAAAATCGACAAGTAATGAGTATTGTGCACAAGATAATCGGCACTGGTACATATGACTACAAAGAGGAAATACCTGATGAAGAACATAAGATTACAAAGAAATACTTACTGTACAAGAAAATATTGAAAACCATTTTGCCAGGCATGAATATTTTGGCCTGTATTATAATAGTGTTTGTTGGCCCATGGATCGACGAATCTCTTTCTGGAAAAGTGGAAATAAGGGACTACGATGATGAAGGTATTAATTTGAAAGTACCAGTACACTTATGGTATCCTACTGAAACACATCAAGGTATACCATATTTACTGGCATATTTTGGACAATTCATTACTGGCTTTGTATGTGCAGCAACTGTGGCAGCCTGTACCTGCATATTTTTTACTGTCTGTACACATCTGATTATTCAACTAGAAAGGTTAATTATATCTGTAAAAAATATACAAAAGAGAGCGTTGAAATTGTATAATCTAGGTCGTAATAAGCCATGTAAAATACAAAATATTGATAAACTCTACGAAGATTCAGAATTTATGAAAACTTTGGAATATTGCTTTAAACAAAATATTGAACATCATCAGCAAATTATCAGAATGTCTTCACTGTTCAACGAACTTTGTAGCGTTCCTCTGTTCGCCTCGCTGTTCTCTGGAGGTGTAGTGCTCGGACTATCTTCTTTGATTATTGTTATAGGAACCGACAAACCAGGCACAGCTTCTGGGACTTTCATTTTGGCTTTAGCTGAAATTGGCAACGTTTCAACCATTTGCAAGTTTGGACAAATAGTAACTGATTTGAATGAAAAGTTTCGCGAAGAAGTGCAGAACTTGGCGTGGTACCATTTCAGTGCAAAGTTCAAAGCCAGTATGTTGATAGTGCAGGAATATAATCGCCAAGAATTACAAGTCACTGGTGCTTTTCAGTTTAAAGCCAACATGGAATCTTTTAGCAGGATCTTGAATACAGCCTATTCATACTTCAGTCTGCTATTGGCTTTCGAGAATAATTAA

>OR12

ATGTTGAACTCATTGAATTTTGCTATCATTATAATATTCTTTGTAATGTTCAAGATCATAATATTTACTAGTCGCACTGATTTTAGAAATATACACAGAATGATTGGCATTGGTGTTTATATATATGAAAATGAAGAATTTGAAAATGATGAAATCAAAGAGATAAAAAGAAAGAATTTAAATTTTAAAAAATTACTGAAAACTTGTCTACCAATTGCGAATTTAATGTGCGGTTTCAGTGTGGTAATAATAGGACCGTACGTAGATAAATTTTTTGGTTATGCTGAGACTAGAATTTATTCAGATAGTGGCGTAAATTTTTTATTACCGATTCCAATAATGTATCCATTTGAAGCACACGAAGGTGCTAACTTTATTTTCGCCTTTTTTCTTCAAATCTCACTTGCCTTCATTGTTTTAATAATATTATTATCATCCAGTTCAATATATATTACCGTCTGTATGAATATAATTATGCAACTTGATCTATTGAAATATTCAATAAAAAATATAGAGATTAGAAGCAAAAAAATGTATGAAAAACTTTATTTTGAGAAAAAAAATAAAAACTTGGGCATATCACAACTTTATGAAGATAAAGACTTTATGAAATGTATGGAATCATGTTTGTTACAAAATATAAAACATCATCAGAACATTCACAGAATGCACAAACATCTTTTAAATATGATGAAGTATATATTGGTGATTACAGTGGGTTCAGGTGTTGCAATGGTTGCAGTTTCAGGTTTTCTAGCTCTCTCTCTTGCAGATCGTCCTGGAGGTCTGTTGGGCTTATTCATCATGTGTGGCGCGCAGATATCTAACATACTAAGTGTATGCTATTTGGGAGAACTTGTCTCTGATATGAACAATCAACTAAATTTTGAATTATATTCAGTAAATTGGTACTGTTATACAAAGAAAATTCATCAATATTTGAACATAATACAGATTTATACAACAAAAGACATGGCTGTAACTGGTCCCTTTAGTTTAATTGCAAATATGGAAACATTTGGCAAGATGATGAACTCCGCCTATTCTTACATGAGTCTTTTGTTCGCTTGGAATTCCTCATAA

>OR13

ATGTTTACTAAATTTATTGAATATCTGGATAATTTCATAGAACATTTTGATCGTGACGGAAATGAGATTTGTGATCAGACGATTTACAAAGAGTTTAAAAGATTATTAGAGATCATGGCAATTTATCCAAAATTGAAACCAAAAAGATTGGTAATAATTAATATTTTAAATTTTTCATTTATAAACGGTTTATTGCTGTTCATGATTTTCATTAGCACAGTCACGGCTGTGTGTGCAGGAAAAGAGGATTTTCCAACTATGGCGCACTCTATTGTCTATGTGTTGGTGACAATTCTTTTCTTGTTTTGCTACAATGGGATGGTTTTCAATAGATCCAGTATAGCTATCATGCACACAATATTCGGGCAAAACGTTTACGACTATATGGAGCAGAATAATGATATACCTGCGGCTATAAAAGCGAAATATGTCCGTATGAAGAAGGTGATCAACCAAATAGTCGGAGTAGCTTCAGTATTGTTGGCTTTGTTCATAGTTTGCGTCGGACCGCTGTTGAATAACACGGATGTAACGGAAATCACTGTCCAACATCCGGAAAATGGTATCAACTACGATCTACCGATACCATTATGGTTGCCGTTTGAAGCTAGCAATGGTTTTGCGTATTTCATAGCTGTAGCTATAATAACTGTAACATTTACCTATGTATTCCTGATAAACTCTGTTGTGTCGGCATTATTCTACAACGTGGCCATGACTATGGACTGTCAATTAGAAATTCTTATCAAATCAATTAACAATATAGAAGAAAGAGCTATAAATATGTACAAAAGAAATAATAATTTGTCTAAACTTCCCAAGTCATATGGATTATATGAAAGTTTGGAATTTCAAAAGATTATTGAAATCTGTATGAAACAAAATATTCAACATCATCAACAGATTTTAAGACTTCGTAAAGCGATAACAAAGATCTCCGAATACTGTGTACTCGTGTTATATGTGGTTTCTGGGATAATAATTGCGCTCACTGCTTACACGGCTACCACGGTCTCCAACGAACCGGGAACCGTGTTGACTTTTGGCTGCCTGACCACTTTGTTCATTCTGGCGATTGCTGGTGACTGTTTGATGGGGCAATCATTTGTTAATTTGAACGAGAAATTAAGAATCGAAATCTATTCGACGAAGTGGCGTCTTTATTCCAAAAGTACGAGGCAATTAATTGGAATAATGCAGCAATACACATGTCGTGATTTTAACATAAAAGTGGCATTCAATGTAACGTTGGATCTGGAATTTTACGGCTCGTTAATGAACACAACTTACTCATACTTGAGTATACTAATTGCAATGAACAAATAA

>OR14-JI

ATGTTAAATTTATGGGCAAGACTAGATCGTTATTTGGAAAGTTTTGATAGTGTACATGGAGAGCAATTGGAGCGTCTATTTATGGATTTATATTGGCATCTATATAAACTGACCGGTTTTGTGGCTGACTTAAGACCCATTCACAAATTGATTCTCACTTTGTTCAGACATGCAGTATTAGTTGGCACCTTTGTATGGCACATAATATTATTTATGTTCACCATACCTCTTATTGCAGAAGAATCTTTATTGATTAGAAATGTACACTTTCTTATGTTCATTGCATTTTCTTTGACTTTATTCGTTTGGTTTACCTCTAAAAGGAAAAGAATTATAAACACTCATAGATTGATTGCCGAAGGATTCCATTTGTATGGCGCTGAAGGCGAGATTATCACTGCTCGCTTGGTCAAGAACTTTCGTAGAACTTATATTGTCTCATTATCAATACCAACAGTTGCAATCTGTTCTTCTGCTTTCTTCATGTTCTTCGCTTCAACAATAGACGAATTAACTGGTTCACCGCCTCGAACTGATCCAGAAAATGGTGTTGATATTTCTCTACCAGTGAAATTTTGGTACCCATGGGATATAAAAAAATGGTATTATTTTCAATTGATACATGAACTTATGCTAGCTGCCTATGTTGGTATTATTATTTCTACGTCGGATTTAATATACGTCAACATCACATATAACATTTGCCTTCAATTACAACTACTTCTAGAAACTTTGGGAAATTTGGAAAAGAGAGCTTATAAAGAGTTTCATGAACTATATCCAGATCAGTGTCCTGTCAAAAAATACAAGGATCCAAAATTTCTGAATTGTTACGAGAGCTGTTTAAAACAAAATGTATTACACCATCAATTTATACACAGATTATTTATTGAAGCGATACCACTGGCTACTATACCAATATTTTTCGCTTTTGTGATGGAGGCTATAGCGTTGGGTATA

>OR16

ATGGAGAAATTTTTGGAAAACTACTTCAGCGAAGACAATGAAAATCTACATCAATTTGTACGAAAAGATTATGGCATTCTATTCCAGATTAGCGGCTTCTACAGAAGAATGGACACAGTAAAAGAGAAATGCTTCACCACATCCTACGTATTCTTAATAATTACAATGTTAACCTATCAGGCAATACTGTTCTTGATTACCATGTTCCACACTGATAATGATTTGATAATTATGTTTCAAACTTTTCATTATTTTGTGTTGACTTTTCTATCAATGCATTGGCTATTTTTTATGTTTGTCAACAGAGAAAAAGTTGCTCGTATTTTCAAAGCCATTCATCATGGGTTATATACTTACGATGAAGAAAGAGAAATTTATAGAGAAAAAGTGAAAAATGCTAACCAATTCTATCGTAAGTTGACCATTAAATATCCACCAATCAACTGCATTGCTGCGTCAATATTCGCTGAAATTGGTCCATTAATCGACCGCTGTATCAGAAATATTCAATATGAAACGTATTCACCTGGTGGTCTGTCACAGAATATCCCCATACCCACATGGATTCCCTATGGAACTGATACCAATTTTAAATTAATTTTCACAATCTTTATCAAATTAGTTTTTGATTTTTATGTTGGCTTAATATTCGGCACTTGTCTGACAATCATTTTCGGAAGCTCTAACATGGTGCGTACGGAAACGGAGCTGTTAATCCACTCGCTGAACCATCAACCATCTCGCACGGAAAATCTTTTCAATAAGACTGCATCGAACAGAAGAAAAATTAACATAACTAAACTTCGTGAAGATAAAGAGATGGCCAGCTGCTACAAGCGGAATCTTCGAGATAATGCTATACATTATCAAAGGATAATAAGTCTTTTCCTAGATGTACAAAATGTACTATCCGTGCCTATGTTTTTCACCTTCATATGCTCATCAGTAATTTTGGCATTATCAGCAAGTGCGGTAGTATTTGGTAAAGGACAAGTTGGCCTGATCACGTCCGTAGTAGGAATATTTATTACAGAATCCATGGCCTTGCTAATCATATGTTGGTTTGCAGAACGGGTAAGACAATCCAGTGAAAACCTCCGAGAAGCACTGTACGCTACAGACTGGATTTATGCGCATAAAGATGCTGCAATCTATATACAGATAATGTCTTCTAGAGCGCTCAAACCACTTACGCTAACGAGCTTTGGTCTTCTACCGATTACATTTGAAACATTTGCCAATATAATGAACACTGGCTATTCTTATTTTAACTTAATTTATGCCACGAACTCAGAATGA

>OR17

ATGGAGAGATTTTTGCAAAACCACTTCAGTGAAGACAATGAAAATCTACAACAATTTGTACGAAAAGATTATGGCATTCTATTCCAGATTAGCGGCTTCTACAGAAGAATGGACACAGTAAAAGAGAAATTCTTGGCCACATCCTACGTATTCTTAATAATTACAATGTTAACTTATCAGGCAATATTGTTCTTGATTACCATGTTCCATACTGATAATGATTTGATAATTATGTTTCAAAGTTTTCATTTTATTATGTTATATATATTTTCAATTATTTGGCAATTCTTTATCTTTGTCAACAGAGAAAAAGTTGCTCGTATTTTCAAAGCCATTCATCATGGTTTATATACTTACGATGAAGAAAGAGAAATCTACAGAGAACAAGTGAAAAATGCTAACCAATTCTATCGCAAGTTGTCCATTAAATTACCATCAATGTGCTTTTTAGTTGTGGCGGTAATTGTTGAACTTGGTCCATTGATCGATCGTTATGTCAGGAATATTCAATATGAAATGTATTCACCTGGTGGATTGTCACAGAATATCCCAATACCCATATGGATTCCCTATGGAACTGATACCAATTTTAAATTAATCATCACAATGTTGCTGAAATTGCCTGTTGATATATGGACCACCGTAACATGCGGCACATGTCTAATGATGGTATTAGAAAGCGCAAACGTGCTACATCTAGAAATGGAGCTGTTAATCCACTCGCTGAACCATCTACCATCTCGCACGGAAAATCTTCTCAATAAGACTACATCGAACAAAAGAAAAATTAACATAACCAAACTTCGTGAAGATAAAGAGATGGCCAGCTGCTACAAGAGGAATCTGCGAGATAATGCTATACATTATCAAAGGATAATAAGACTCTTCTTAGATGTACAAGATATACTATCCGTGCCTATGTTAGTGACCTTCGTAAGCGAATCAATATTTTTGGCCTTATCAGCGAGTGCGATTGTATTTGGTAAAGGACAAGTTGATCTGGTCATGTCCGTTTTAGGAATATTCGTTTCAGAATCCATGGCCTTGTTAGTCAGCTGTTGGTATGCAGAACGGATAAGACAGTCTAGTGAAAACCTTCGAGATACACTGTACGCTACAGACTGGATTTATGCGCCTAAAGATGCTGTAATCTATATACAGATAATGTCTACAAGAGCGCTCAAATCACTTACACTTATGAGCTTTGGTCTTTTTCCGATTACATTTGAAACGTTTGCCAATATAATGAACACTGGCTATTCTTATTTTAACTTAATTTATGCCACCAACTCAGAATGA

>OR18

ATGGAGAAATTTTTGCAAAATTACTTCAGCGAAGACAATGAAAATCTACATCAATTTGTACGAAAAGATTATGGCATTCTATTTCAGATTAGCGGCTTCTACAGAAGAATGGACACAGTAAAAGAGAAATGCTTCACCACATCCTACGTATTCGTGATGATTACGATGTTAACCTATCAGGCAACACTGTTCTTGATTACCATGTTCCACACTGATAATGATTTGAAAATTATGTTTCAAAGTTTTCACTTTTTAGTGTTATATTTTTTATCAATATTTTGGCTATTCTATTTCTATCTCCACAGAGAAAAGGTTGCTCGTATTTTCAAAGCCATTCATCATGGATTATATACTTACGATGAAGAAAGAGAAATCTATAGAGAAAAGGTGAAAAATGATTACCAATTCTATCGTAAGTTGACCATTAAATATCCACCAATCAATTCCATTGCTGTGGCATTATTCGTTGAAATTGGTCCATTAATCGACCGCTTTATAAGGAATATTCAATATGAAACGTATTCACCTGGTGGTCTGTCACAGAATATCCCAGTACACACGTGGATTCCCTATGGAACTGATACCAATTTTAAATTAATTTTCACAATCTTGGTCAAATTGGTTGTTGCTAGTTGGGCTGGCTTAATGTTCGCCATTGCCTTGACGATGATTTTAGAAAGCACCAACATGCTGCATCTGGAAATGGAGCTATTAATCCACTCGTTGAACCATCTACCATCTCGCACGGAAAATCTTTTCAATAAGACTGCATCGAAAAGAAGAAAACTTGACATAACCAAACTTCGTGAAGATAAAGAGATGGCCAGCTGCTACAAGCGGAATCTGCGTGATAATGCTATACATTATCAAAGGATAATAAGACATTTCTTAGATCTACAAGCTATCCTATCCGTGCCTTTGTTGGTGAACTTCATAGGCTCATCAATACTTGTGGCATTAGCAGGGATTGCGTTAGTATATGGCAGAGGACAAGTTGGCCTGATCGTGTCCGTAGTAGGAATATTTATTACAGAATCCTTGACCTTGCTACTCGCATGTTGGTTCGCAGAACGTGTAAGACAATCCAGCGAAAACCTCCGAGAAGCACTGTACGCTACAGACTGGATTTATGCGCCTAAAGATGCTGTGATCTATATACAGATAATGTCTTCAAGAGCGCTCAAATCACTTACGCTTACGAGCTATGGTCTAATTCCGGTTACATTTGAAACATTTGCTAATATAATGAGCACTGGCTATTCTTATTTTAACTTAATTTATGCCACTAGCTCAGAATGA

>OR19

ATGGATTTTTTTGAAAAGTTGTTTTCTGAAGATAATGACATTGTTGAAATTACCATGGTTCAGGAATATCATCATCTAGAAAAACTTAGCTGTCTCTATCCTGACGTGAAAACTAGAGGCAAGAGATCCGTCAGTATCGTACACTTTTTGATTTATATTCTAACACTCGTATATCATCTCTGTGCGCTGTCTATCACTGCTGGTTATGTCATGAATTCATTGTCATTACTGTTCCAAACAGTGCATCTGTTGTTATTGATACTGTTAACATTTTCGTTACTATGTTCACTCAATTTATGTAGACCTATTGTTGCTCGAATACATAAAGATTATTATGGAGGTTTACATGGATATGATGAGTTTACAGAAAATTACAGACCACATTTAAAGTTAAAAGAAAAAAAGAAAAAGAAATTTTTATATGGTTTGATTGTGGCATTGGTCTTAAGTGTTGGCGTAGTAGTTATTTTAGTGGCACCTGTACTTCATAGCGCATTGGAAACGACTCTAGGAAATAATACAAATGGAGTATCTTATAATTTACCATTTCCAGAATGGACACCATTTGGCACAGATACCTGGACTTTTTATATAATTTCATTTTCGTTGCAGACTTATTCTGCAAATATGATGTTATTGACCATCTACTGTGGTGTTTTTATGTTATTTACATTTACGCAATTTCTTCATTCGGAGATTGAATTATTGATACATTCTCTAGAACATTTGCAAGAAAGAACACTTTTATTAATTGGTAACAGAAACAAAACAGTGGATTTCTCTATAATGAAGATGGATAAAGAATTAAATAGTGCCTACAAATACTGCCTCAAAGAAAATGTGCTACATCATCTAAAAATTATAGAGATATGTAATGATGCAAATATTGCCCTTTCTGTACCAGTCTTTGTGAGTTATTTTTTTGGTACAATTACAATTGCTTTTGCAGGAGTAGTAATCTATGGAGATGATCCGTTAGCATTAAAATTAACGGCTTTAGCTCTAAGTGGTGCAGAAACATTTAATGTATTCTATTTGGCATGGTGTGGAGAACAAATAATAACTTCAAGTGAAAAATTAAGTTTCATTTTGTACAAGACTGATTGGAATTACTTGGATAAATCTGGATATCTGATGATACAAATAATGATGGCCAGAGCACAGAGACCTCTCTCTTTGGAGGGTGGATATTTCTTCCCAATGTCTTTTGAAACCTTTTCTTATGTAATGAATACTGCATACTCCTACTTCAATTTACTTTTTGCATTTAAAGAAGCTGCTGAATAA

>OR20

ATGGCATTTTATGACAATATAACAAAAGAGGATACTGATGTTGCTTATAAAACAATTAAAAAGGAATATGGCTTTGCAACAGTATTGTGTGGCACTTATGCGATATTTAAACCAGGCATTTATACAATTTTAAGTATAATACAAATTTCAATATTTTTTATTGTGCTCATATTAAATACGATCGCATTAGCAATTACAGTTTATCTTTCATTAAATGATCAAACCGTTGCTACACAAACTGTACATTTTATTTTTATGAATGCAATTACTTTTACAATTTTATTTTCAAGTAATTATACAAGGCCAAGTCTGAGTAGATTTCACGATGACATTGGAAGAGATATTCAACCATATGATCATGATACTATGAAAACTGTGGATAATTTGAAGATTTATATGAAAAAATTACAAAAAGTGATTTATATAACGGTACCTTTACTAATAGGTGTTTTTGGGATGTTAATTGTAAGCTTAGGACCTATATCTGATTATTTCACAGGGGATTTTGGTAAAAAGATATACAATGAAAATGGCGTTTCTGTAAATTTAGCATTACCATGTTGGACACCGTTCGGCAGTAACTCTACAATTAGTTTTTTAATTTCTGGTACATTGCAAATCCTCGATCATTACATAATTGTAACAGACATAGTAACAGCTAATCTGATCATGACAGTCATGGTAAGACATGTATTAATAGAAATACATTTATTAATATCAGCATTCAACAGATTACCTGAGAGAGCAGCAATTATGTATCAGAAAATATATGGTAAAAAATGCAACGGGAAAATTACAGAATATCATGAAAACCCCAGTCTACAACATTGCTACAATGAATGTATTAAACAGAACATTAAACATCATATTCGGATTATTAGATTGATTAACGATATGAACACATTCTGGAACTACACTTTAATGATATTCTTTTTTGTAAGTACAATCTCGATGGCCTTCTCTGGGATTCTTATAATGTTTGGTGACGGGAAGATTACGACGCTGGTTAATGCACTGGTAATGGGGTTTACAGAAATTTTTGATGTGTTTTACATTTGCTGGTGCGGGAAGTCAATAAGTGATGCGAGCGAATTACTATATATGGCACTGTATGATACAAAGTGGATAAACGCAAATAAGGAATCTGCAAAGTTTATATTAATAATGATGGCAATGACTAAAAATCCTTATGAAATTTATTTTAATTCAATCAATAAGCTGCCAGCAAATTTTGAGACATTTGCAAATCTGATCAATACAGCGTACTCATATTTTAATTTACTATACGCCTTTAGAGAAGATAAAGCAAACTAA

>OR21

ATGGCCAATATGATTAGAACTTTTATAAGAGATTTTGATGAATTTGGCGATGAACTATCTGAAAATACAATTTATGAAGAATATTTTATTATGTTAAATATTTCTTTATTATATGTCCCATTAAAGTCATTTTCAATCTCCATAGTACGGTTTCTTCAATTAGTTCTTACTGGAATAATCAATTTTTATATAATAACAATTTGCTTAATTACAGTAGTTGTTGGCAGGGATGATTTCATAATTTCAGGAGGTGCTCTAAATTATGCGATTATTACTTTTACTATTGACATTATACTTTTACATGTGGTTTCGAAAAGAAAGAATATAGCCAAAATTCATAGCATTATTGGAAGTGGTTTCTATAAATACAATGAGACACTTAGTGGCGAAATAATCCACGTAAAGTCAAAATATAAAAACGTTATTTGGTGGATAAAAAGAGTAATACCGGTTTGCACTTGTTTCTGCGGAACAGTTCTTTTATGTTTGGGGCCTCTGATTGACCGTTTGGCAGGAGTAAAGGTACAGGAACATCCAGATAACGGTATAAACTATAACCTACCAGTACCTATATGGGTGCCCTACGATACGAATGGTCCAATTTCATATTGGATAACTGTTATCTCCATTGCCATTTCGGTTACATTATCAATTAACGTTTTTATTTCATTCAATATATTATATTTCACCATCAGTAATTACATTGCCTGCCAACTGGAACTATTGAAAATTTCTATTCAAAATATTGAAATTAGAGCAAATCATATAAAAGAACTCCATGGAAGTTACAATTTTACAGAGTGCTTGAAAGTTTGTCTTAAGAAAAATATTGAACATCATCAAGAAATATTGAGCTTACATCATTTGGCAAATGAAGTGTTCAGTGTTCCAATTTTCATATTCTTTTTGTTTGGTGGAATGATGGTTGCCTTAGCCTCTTACGGAGCAATATCGGTCACTGGACAGCCAGGAACTCTTTGGATTTTTTTTAACATTAGTATAATTCAGATGTTTACTATAGGAAGTATGTGTAAAATGGGGCAAACTCTCATAGATTTGCATATGAACATACGACAAGAAGTATATTCAATTGATTGGTATCGTTATTCCACAAATATTAGTACTTCATTGATAATAATTCAACAAATTTGTTTACGTGATCTAATTTTTAAAGGACCTTTTGGACTCTCAGCAACTATGGAAACATTTGCAGATATTTTAAACAAAACATACTCTTACTTCAGTCTTATAGTAGCTTGGCAATCTTGA

>OR22

ATGGCCAACATGATTAGAACTTTTATAAGAGATTTTGATGAATTTGGCGATGAACTATCTGAAAATACAATTTATGAAGAATATTTTATTATGTTAAATATTTCTTTATTATATGTCCCATTAAAGTCATTTTCAATCTCCATAGTACGGTTTCTTCAATTAGTTCTTACTGCAATAATCCATTTTTATATGATAACAATTTGCTTAATTACGGCAGTTGTTGGCAGGGATGATTTCATAGTTTCAGGAGGTGCTCTAAATTATGCAATTATTACTTTCACTTTTGACATCATACTTTTACATGTGATTTCGAAAAGAAAGAATATAGCCCAAATTCATTATATTATTGGAAGTGGTTTCTATGAATACAATGAGACACTTAGTAGCGAGATGATCCACATAAAGTCAAAATATAAACAACTTATTTGGTGGATAAAAAGAGTAATACCGGTTTGCACTTGTTTCTGCGGAACAGTTCTTTTATGTTTGGGGCCTCTGATTGACCGTTTGGCAGGAGTAAAAGTACAGGAACATCCGGATAACGGTATAAACTATAATCTACCAGTACCAACATGGATACCCTACTATAGCAATGGATCAATTTCATATTGGATAACTGTTATCTCCATTGTCATTTCGGTTACATTATCAATTAACGTTTTTATTTCATTCAATATATTATATTTCACCATCAGTAATTACATTGCCTGCCAACTGGAACTATTAAAAATGTCTATTCGTAATATTGAAATTAGAGCAAATCATATAAAAGAACTCCATGGAAGTTATAATTTTACAGAGTGCTTGAAAGTTTGTCTTAAGAAAAATATTGAACATCATCAAGCAATATTGAGCTTACATCATTTGGCAAATGAAGTGTTCAGTGTTCCAATTTTTATATTCTTTTTGCTTGGTGGAATGATGGTTGCCTTAGCTTCTTACGGAGCAATATCTGTCACTGGACAGCCAGGAGCTGTTTTGAATTTTGTTGACATTAGTGTAGTTCAGATGTTTACTATAGGAAGTATGTGTAAAATGGGGCAAACTCTCATAGATTTGCATATGAACGTACGACAAGAAGTATATTCAATTGATTGGTATCGTTATTCTACAAATATTAGTACTTCATTGATAATAATTCAACAAATTTGTTTACGTGATCTAATTTTTAAAGGACCTTTTGGACTCTCAGCAACTATGGAAACATTTGCAGATATTTTAAACAAAACATATTCCTACTTCAGTCTGATAGTAGCTTGGCAATCTTGA

>OR23

ATGGCCAACATGATTAGAACTTTTATAAGAGATTTTGATGAATTTGGTGATGCATTATCTGAAAATACAATTTATGAGGAATATTATATTATGTTCAATATTTCTTTATTATATGCCCCATTAAAGTCATTTTCAATCTCCATAGTACGGTTTCTTCAATTAGTTCTTACTGGAATATTCCATCTTTATATAATAACAATTTGCTTGATTACGTTAGTCGTTGCCAGGGACGATTTCATAGCTTCAGGAGGTGCTCTAATTTATGCGATTATTATATTTACATTCCTCATTGTACTTTTACATGTGATTTCTGAAAGAAAGAATATAGCCAAAATTCATTATATTATTGGAAGTGGTTTCTATGAATATAATGAGACCCTTAGTAGCGAGATGATCCACATTAAGTCAAAGTATAAACAACTTATTTGGTGGATAAAAAGAGTGATACCGGTATGCACTTTTTTATGTGGGACGGTGGTTATGTGTTTGGGGCCCCTGATTGACCGATGGGCAGGAGTAAAGGTACAGGAACATCCAGATAACGGTATAAACTATAATCTTCCAGTACCTGTATGGGAGCCCTACGATACCAATGGACCGATTCCATATTGGATAACTGTTTTTTCCGGAATCCTTTCGGTTACATTATCAATTACCGTTTTTATTTCATTCAATATATTATATTTCACCATCAGTAATTACATTGCCTGCCAACTGGAACTGTTAAAAATTTCTATTCAAAATATTGAAATTAGAGCAAAACATTTATACATGGAATATTATAAAATTTCAACTTCTGAAGAGAGCTTAAATGATATAAACGAACTTTATTCAAGTTATAATTTTACAGAGTGCTTGAAAGTTTGTCTTAAGAAAAATATTGAACATCATCAAGTAATATTGAGTTTACATCATTTGGCGAATGAAGTGTTCAGTGTTCCAATTTTCATATTCTTTTTCTTTGGAGGAATGATGGTTGCCTTAGCTTGTTACGGAGCAATAACTGTCACTGGACAGCCAGGAACTTTTTGGAATTTTGTTAATATTAGTATAATTCAGATGTTTACTATAGGAAGTATGTGTAAAATGGGGCAAGCTCTCATAGATTTGCATATGAACATACGACAAGAAGTATATTCAATTAATTGGTATCGTTATTCTACAAATATTAGTACTTCATTGATAATAATCCAACAAATTTGTTTACGTGAACTAATTTTTGAAGGACCTTTTGGAGTCACAGCCACTTTGGAAACATTTGCAGATATTTTAAACACAACATATTCCTACTTCAGTCTGATAGTAGCTTGGCAATCTTGA

>OR24

ATGGAGGAGAAGCCCAACTTAGACTATCTGGTCGATAGCATCTTATACAACGAATATTGGTATCTGATGCAAATGTCCTGTATTTATCTTGACATGCGAACTCCAACAAAAAAATGTCTAAGTATAATAAATTTAATATTGATGGGATGCGTAGCTATTTACGAATTACTTATCTGTACGGAGACAGCGATACGAATACCCGAGGATAATCTAATATCAAGCCAGGCTATACATTTTGTTATTTTGCAAATAATTTCTTTATTCGTTATAACAACAACAAATATTTTTAGAAATAATTTTGAACAAATACACTATTTAATGGGATCAAACATTGGCGCCTATGATGAAGAAACAAAACAAGGAATGGAAAATGTTAAAAATAAACTGAAAAACAAGAAAAAATTTTTACTGCATTTGATAATCATATATTTAATATTTGCTGGGCTATTTACAGCAATATTGGCTCCAGCGATAGATAATTTCTTTGCTAGCAATATTAGAGAAGAAATTTATAGCCCTGGAGGAGTGTCTTTAAATCTGCCATTGCCGCTGTGGTCTCCGTTTGGCACTCACACTAGCTTCGGCTATTTAATGTCACTCTTCATGGAATTGTTATGCACATATTGTATAACTGTGGTTGTGATTGGCGGCGATATTCTTCTGTTAACCGTTTCACAATTTGTCACTACAGAATTGGAACTATTAATGATTGCATTGAAACGTTTACCACAGAGGACAATACGATTGTACAAAATACGCCATAATATCGATACTGAATGTCACATTAATTTTAAAGAATTAAAATATAACGCGAACTTTATGAAGTGTGTACAACATTGTCTAAAAGAAACAGTAATACATCATCAGAAAATATTACAAACTTTTCATGAATTAAATGGATTAATTTCTATTGCTGTATTTGTGGCATTTTTTGTCGCATCAATCGTGATTGCCATCAGTGGGTTTTTAATGATATTTGGAAACGGTGCTATTGGCTCGATGGCTACTGCTTTCATTTTGGGCATCGGGGAGGTTCTCAATGTATTATTGTTATGTTGGTGCGGAGAACAAATGAGGCAAGCAAACGAAAAGCTATCAATAGTTTTATATTCTGTGGAGTGGTATTACATTAAAAAAGAAGCAGTAACTTATTTGATGATAATGATGATGAGAGCTCAGAAACCGTTGGTGATGTTTGCTGGAAGACTAGTGCCGGTGAATTTTGAAACTTTTAGCAATGTTATGAATACAGCTTATTCCTACTTTAACTTGCTGTATGCATCAAAGATAGAACACTAA

>OR25

ATGAGCACTGGGACATTCGTAGACCGTCTGTGCAAGGAAGACGACGAAAGTGTCTCAAAAACAATTTACAGTGAATACTGGCATTCTATGGCTCTGTCTGGTTTGTATCTAAGATTGGACACACCGAAATGGAGGATGCTCAGCCTCTTGCAGTTATTATTAATGATGAGTTTCATAACATTTTTAATTGGTTGCGCCATCGTGACGTGCATTCAAACAGCACACAAACCGATGATAATGTTCCAATATATGCATTACATGATTCTGACCGTCTTGATTGTGATTTCAATCATCACCAGCATCTTCAACCGGCCAAAGATAGCAAGAATGCACCGTGCCATCGCCAACGGATTGTCGCATTATGACGAGAACACTGAACAACACAGAAAGAAGATGTTGAAACGAAAAAGCAAATACAAAAAAATTCTATTTGTTCTGCTAGCTTTTTACTTCATCAATGTGCCCATTTATCTGGCGTTTGTAGCCCCAGTGTTGGACGAATACTCTTCCAGCGCCAACAGAACTGAGACCTACAGAGACACTGACTTAGCAATAAACTTGCCCGTACCATTCTGGCTGCCATTCAGTACAGATTCTCTGTTGATGCCAACCATCGGCTATGGCATGGAGTTGATATTGGCCTGTATAATTGTTGGCCAAGTAGCTGCTGCCGATTTCGTACTCTTGACTGCAGGCATAACAATCACCATGGAACTGGAATTGTTGATCATATCGTTGAGCAGACTGCCAAAGCGGGCACTAGCTATGCATGAAGAACGCCATGGACCCGAAAGAAGTCGCCAGATGGACGTAAAACGGATTAGAGAGGATAAAGATCTAGCTAAATGTTACGCAGACTGTCTGAAACAGAACGTGCAGCACCATCAGCGCATTATCCAGATGTTTGATGACTATTACAACTTCTTTTTTATTCTATTATTTGTGGTCATCATGACTGGGACGATATTCATCGCCATATCTGGATCACTTATAATGTTTGCAGAAGGAGGGCTTGCTCTCAGAGTGATGTCCGTTGGATTCTGTTTCGCTGAAATATCCAATATATTCTTCAACTGTTGGTGTGCCGAGCAGATTTTCAATGCGAGTGAACAGTTATTGCTTGCCCTGTACTCAACCAACTGGAGGATGCTGACCAAAGATTCGGCTCGCACTGTTCTAATCATGATGACAAGGGCGACTCAGCCCTTAACTTTCAACTGCCTCGGGCTGGTAGTAATCAATTTTGAAACATTCTCAAACGTAATGAACTCTGCATACTCCTATTTCAATTTGTTAAACGCATTCGGAGGACAGGATTGA

>OR26

ATGGCCTTTTTGGACAAGCTGTGCGAGGAAGACGACCCGACGGTGGAGAGGTCGTTATACAATGAGTACTGGTACGTGATGTACATCCTGGGCCTGTATCTCAAACTGAACACACCAAAATGGCGACTCCTGAGCGGCGTGCAGTTCTTTGCATCGTGCAGTTTGATGATGTACTTTGCGATAATCATGATAATCACTGCGGTCAAGCTAAAGGAGAACATGATGGGAATGTGTCAGGGCTTGCACTATGCGATTTTACTATTTCTAGTTGCAGCGATGGCGTTCTCATTGAACATGTGTAGACCGAGCATGGCTCGTATACATCGTGGCATCGGCACAGGATTGTCTGAATACGACATGGAGACCGAAAAACATAGAGCACCACTCAGGCAGCGAATCAGAAACTATCAAATCCTCTTAATAAAGCTGTATTGTATTTACACAATCGTCGCGATAATATTTCTAGGCATCATTGCAATTCTTTCTGAAAATGCTGATGAGAGCGAAAACGAGCTGTACATTGAAAACGGTATCTCGCTGGATGTACCAATACCGCAATGGTCACCCTTTAGCACGGACACGACGCTCAAGTTCTTATTGGCTATCACTCTACAGGCCCTTGTATGCACGGACGTTGCTTTAAAGAATTGTGGATTTAATGTCGTGAATATGACTGTGTCCTTAAATATCGTCTTGGAGTTAGATCTGCTGATAATATCGCTAGACAGACTGCCTCAGAGAGCACTAATAATGTATCAAGAACGCCATGGCCAGGAAAGTAGTTCCAAGGTGGATATGAACCGGATTAGAGAGGATAAAGATCTAGCTAAATGTTACGCAGACTGTCTGAAACAGAACGTGCAGCACCATCAGCGCATTATCCAGATGTTCAACGATCTGAGCACACTAGTTACTGATCAAATGTTCCGCGCACTGTCCACTGGTGCTTTCTATACCGCCATCTCCGGAGCGCTAATTATGTTCGCTCAAGGATCAATCGCAGAAAGAGTTGCTTCCGCAGCATTCTGCTTCGCTGAAATAACAAACATATTCTTGCATTGTTGGTGTGCAGAACAAATAACGGATGGGAGTGTAAGATTGTCGCGAGCCCTGTACTCCACTAACTGGATCTTGATCGACAAAGAAAACGCTCGTATTGTACTGATAATGCTCACAAGGGCCAACCTGCCTCTAGTTATGACTGGCAAAAATTTAGTCAAGATTAATTTCGAAACTTTTGCGAACATAATGAATTCAGCATACTCGTTCTTCAACCTGCTGAATGCAGTCGAAGAACAAAAGTAG

>OR27FIX

ATGAAGTTCATCCAGAGAATTGACGCTGAAACTGATGATATAGTTTTGGCTCAGGCGATGAAGGAATATAGTTATATGTTACAGTTGAGTGCTTTGTTTGTAAACTTACGTCCAAAATGGCGCCTCTTCTCAGTAATACAATTTATTGTCTATTATTTACTAGCGATTTTCTTTCTCATCACTCTGCTAATCACGGCAACAGAATGGGAGAACGATATTAACAATACAACACAGGCGATTCATATTGCTGGCTTTACAATAGTTGTTTTGATTATTTTAACTGAATTTTCCGTGAAAAGAGCCGACTTTGTGGATTATCTAAGAATTGCTGCAAATAATTATTATATCTACAGCGATGGATTTCAATCGAAATACGCAACTAAATGGAAGAAAGATATAAGAAAAATGAAGATAATGGTACTAATCTTCTTACCTCTATATTATCTATTTTGCGCCTTTACAGCCATTTATTTAGCACCAATTATTGACGACCTTCAAGGATTCACTATCAAGGAAAGCTACATCAACAGCATTTACACAAGGCTTCCGGTTCCACTAGTACTTCCTTTCGAAGTGAATAATAACGCACTACATTATCTAGCATTTACCATGGAAGTAATAATGGCCGTACTTCTCGCGTCGGTGGTGGCTGGCGCTGATTTGGCCTTAATTTTCTACGGACAAGATATTGCTATACAACTTAAACTATTGAAAGAATCCGTACAGGAAATTGAGAACAGAGCTTTCTATCAATATAAAGAAATCAACGGAAATAATAAAGTAAAGAACTTAAAACAACTATATAAAGACGAAAAGTTTATGAAAATTATTAACAAGTGCATTAAACAGAATGTTGAACATCATAAAATAATTTGCCATGCATTTGACATTATGTACACATTGTCAAAATGGCCGACCGCAGCGGCGTTTATGGTCGGATCAGCTGTAATTGCGTTGTCTCTATTCTCAATTGTATCAACCGGTGTCGGCAGAATTGCCTCCCAGCTGTTCTCTATGGAGTTACTGTTGATTGAAATTGGAAACATGTTCATTATATGCTGGATAGGACAACAGCTAACTGATCTGAGCGCTGGATTATCTTATGAGCTGTACAATACAAATTGGATGCAATGGAATAAAAGTTGCAAAATGTCAGTCTTAATATTTAGAGAAAGACTGAAACGACCATTAAACATGAGCGCTGGCGGACTGACGCCGATCAACATGGAAACATTTGGAACGATAATGAATACATCATACTCTTATTTCAATTTGATATTCGCCTACACGGACTCAAGCAAGTAG

>OR28FIX

ATGGACCAAGATCAACATATTTATGATACGGCGAAGAGAGAGTTATGGTATTTCGGGCAGATTGGAGGAACATTTATTAATTTGCAACCAAAATGGAAAAGATATTCTATTCTACAATTGGTCATATATTTCATAATTCTGGTATACCATGTTGGATTATTAGGCAAAACCGTAATATTGTCTTTAGAGAACCACGATATAGCTACCATTGCAATGTCTGTCCATATTTTTACATTAGGAATTGTAATAATTATGATATTAATCAACATGAGTAAACACAGAGAAAATGTATCTATTTTTTTAAAAAATATTAACACGGAATGGTACAATTATGGTGACAGGACCACAACGAATCTACAACAGGAATTGAACTTGGAAAGTAAAAAATTTAAAATCAAATTGTTAACAGCTATACCATCATATTTGATAGTCATGGCCATAGCTTGGATAGTGGTACCATCACTGGATCAGTATCAAGGCGAAAAGGAAACATATGAAAATGGTATCTACAAGAAGACTCCAGTGCCTATGTGGTTCCCGTATCTGATCGACAACAATTTCAAATATTTATTGTCTGTGGCTTCTCAAGCTTCTTTTGCATTTGTCGTATGTGTAATCTCAACAGCCGCAATTGTTTCCATGATATTCTTTTCATTGGATTTGATAACACAACTGAAAATATTGAATCATTCTATAAGATCAATTGAAGAAAGAGCTAGAATATTGTACGAAGAGAATGGAGGAAAATTCAAGGAAAAAGCATTGAATTCAGATAAAGAGATTGCAGCTTATTCAAAAATTATAAATTATTGCATCCAGCAAAATGTAGAACACCATAAAGTTATCATAGAATCTTTCGATGCTATGGTTACAATCACAAAATGGCCGATGATGTTTGTCTTCATAATAGCGGCCTTGATTATTTCGTTGTCATTATTTATATCTGTATCAGGTAGAATCCGGTTCACTCATCAGTTACTGTCACTAATGATGCTGCTTGCAGAGATTTGCATTATATTCATATTGTGCTGGCTGGGACAACAAATATCTGATCAGAGTAATTATCTATTCCACCAATTTTTTAATATAAACTGGTTACATTGGGATAAAAGCTGCAAATCCTCCTTTTTGCTAGTAAGAGAGAGATATAAACAGCCAATGGTATTGTCCGGTGGTGGCATAATTGCTGCGAACTTCAGCACTTTCTCTGATATTATGTCGTCAGCTTATTCTTATTTTCAATTACTGTACGCATTGGAAAATGAAACTTAA

>OR29CTE

ATGGACCAAGATCAACATATTTATGATACGGCGAAGAGAGAGTTATGGTATTTCGGGCAGATTGGAGGAACATTTATTAATTTGCAACCAAAATGGAAAATATATTCTATTCTACAATTGGTCATATATCTCATAATTCTGGTATACCATGTTGGATTATTAGGCAAAACCGTAATTTTGTCATTGGAGAACCACGATATAGCTACCATTGCAATGTCTGTCCACATTTTTACATTAGGAATTGTAATAATTATGATATTAATCAACATGAGTAATCACAGAGAAAATGTATCTATTTTTTTAAAAAATATTAACACAGAATGGTACAATTATGGTGACAGCACCACAACGATTTTACAACAGGAATTGAATTTAAAAAATAAAAAATTTAAAATTAAATTGTTAGTAGCTCTACCATCATTCTTAACGGTGATTTGCTTAGCTTGGATAGTGGTGGTACCAATAGTGGATCAGTATCAAGGCGGTAAGAAAACATATGAAAATGGTATTTACATGGAATGTCCAGTGCCTTTGTGGTTCCCGTATCTGATCGACAACAATTTCAAATATTTATTGTCAGTGGCATCAGAAGCTTCCGATGAATGCAAATTCATCTTCTGTGTAACCATTGCAGCCGCAGATGTTGCCATGATATTCTTTTTATTGGATTTGATAACACAACTGAAAATATTAAATCATTCCATAAAATCAATGGAAAAAAGAGCCAGAATATTGTATGAAGAGAATGGAGGAAATTTCAAAGAAAATGCCTTGAATACAGATAAAGAAAATGCAGCTTATTCCAAGGCTATAAATTATTGTATCCAGCAAAATGTAGAACACCATAAAGTTATCATGGAATCTTTCGATGCTCTGCTTACAATCACCAAATGGCCGTTGATGTTGGTCTTCTTAGTAGCGGCCTTGGTGATTGCGTTGTCTTTATATATATCTATATCATATAATAATTTATTATTCCGGAAATCACTGTCAATAATGATCCTGTTCGGAGAGATTTGTAATGTATTCATATTGTGCTGGCTGGGACAACAAATATCTGATCAGAGTAATTCTCTATTTTACCAATTTTACAATAAAAATTGGTTTCATTGGGATAAAAGCTGCAAAACCTCCCTTTTAATCGCAAGGGAGCGATATAAAAAGCCAATGGTAATGTCCGGCGGAGGCCTAATTGCTTCGGACATCAACACTTTCTCTGAT

>OR30

ATGGGTTTCCTAGAAGAATTATCTAAAGAAGATTGTGCTATTGTTGATTCAACAGTTTTTAAAGAATATGGTTACCTAATGTGCCTTTCAGGTTTTTATCCTAGTTTAAAGCCAGAAAAGAAGGTATTAAGCGTCATACAATTTATACTATTTATGTTTACACTATTAATCAACATTAGTGCTTTAATAATTACTGTATATCTTGCTGATGATGATGAAACAATACTGACACAATCTTTACATTTTCTAATTATATTCGGTCTGTCTTTTTTTATATTTCTATCCTTCACAAAATCCAGACCACAATTCAGTCGTATTCACCGGGATATTGGTCAAGGTGGTTTCAAATATGATAAAGACACTCATATTTTTGCTACATCAATAAAAGATAGTTCAAAAAAGAAAAAAAAGATAATATTTTTTGCTTTACCAACTTTTGTAAGTGTGATTAGTTTGGTTATAATTGTAATTGGACCGTTAGCCGATTATTTTGCTGGTAAGTTAGGTGATGAAATTTACACTAAAGGAGGTATTTCACTAAACCTCCCAGTACCGTGTTGGACACCATTTGGCAGTAGTACCATTGCTACATTTTTGGCATCAAGTTTAATGGAAGCTGCAGGGCATTCTGTGAATGCATTAGCATTAATAACTGCTAATATTTCTGTATTTTATATGGTTGAACATGTAAGCACAGAGTTAAAAATATTACATCTGGCAATTAAGAGACTAAAAGAAAGAGCTCAAGTTCTGTATAGAAATCGTTTTGGAAACAATGAAATGAAAAATGAATATTTGACTGATTGTTACAAAGAATGTTTAAAAGAAAATATAGCCCACCACCAAAAAATTTTGAGCTTAAACTGTGATATGAATGATGTGTTATCACTTTGTTTTTCTTTTGGCTATTTTACTGGAACACTCATGATCGCAATTTCTGGAGTGTTAGTAATTTTTGGAAATGGCCACATAGGTCCGGTTGCGAACGCTGCGTTCATGGGATTTACAGAAATAATGCAGGTATTGCTTATTTGCTGGTGTGGTGAAGAAGTTACGCAAGCTAGCAAAGATATCAGCACAATATTGTACTCTTCAGAATGGTACTATCAGGATAAGGACAGCAACCAATTATTGGAGATGATGTTATTGAGATCACAAAAACCTCTGTTTCTATATTTTAATAATATATCCAACTTACCAGCTAATTTGGAAACTTTTTCTAATCTTATGAATACTGCATATTCCTACTTTAACTTGCTATATGCCATCAGAGAGGAAGCCTAA

>OR31

ATGGATTTCATTTATGATAGAGAAAATAATGATGCAGTTGAGAAAGCTATATACAGTGAGTTTTATTTATTACTGAGAATATTTGCCGTGTTTCCCAGCCTGAAAAGAGAACATATATTATTAACAGCATTTCAATTACTAATGTACAATGTGCTAATGTGGTACCACTTATTAATAAGTGCGTTCACAATTCAGGTGGTTTATTATGAAATAAGTTTATTCAATATTACTGTACACTATTTTCTGTTGTTCTTGCTTGTATCTATATTTTTGATTGCAATGCTTCCGAAGAGAAAACATCTTTGTCGAATGTACAAAATAAGTACTTCAGGTTTCTTCAGTTACGATGAAGATGTCGAAGATGCACGATTGGAACACATCTCCAAAGAGATACGAAAAGAAAGAATAACATTTGTCTTACTTCTCAGTTTAACAATAGCAATTGCTTTGTTAGCATTTATTTTAAGACCAGTTGTAGACAAGTCACTGGGTTATGTTGAAGAAGGTTCATATTCTGAACTTGGATTAATCCAATTGATGCCAATTAAAGCATGGTATCCATATGAAACCCACGAAGGATTTATATATTATGTTACCTTGTTTGGACAGCTCGCCGCTGCAGCTCTTTTAGGTGGAAATATTGGCTGCCTTACTTTAATTTACACAAATAGTGTACTGAAACTAATCCAACAGTGTAGAATATTGATGTATTCTTTAGAAAACATTGAAGCACGTGCGACCAAGAAATTCAAAAAGTTGTATTGTAAGGGACAGAATAATCAGAAAGTGGTTTACGATGATCGTTTCCACAGGTGTTACTACTTCTGTTTGCGACAAAATATTATTCATCAAAATATTATTTATGACTATTTTGAAGAAATGAAGTCGCTTCTTTCTCTGCCTTTGCAATTGGCAATAATTATTGGAACGTTAGTAATTGCCTTGTCAATGAATGAGATGGCGCAGGGAAGTAAAAAATTAGGTTCAATGATGGCATCGGGACTGTTAGTTTTGACAGAAATAATAATTATATTTATTATGTCCATGTACGGAGAAAAAATGACAAAACTGAGTGTTCAAATAAGAGAATGCATATATTCTATTAAATGGTACAAATATAAAAATCCTTATTTGAAGAATATGATACCAATTATGCTGGAAAGAACCAAAAGGGAAATGGTTATGAAGGGCCCATTCTCTGTAGCAAATTTTGATTCATTTTCAACTGTTATAAATACTGCTTACTCATATTTCAACGTTTTAAGTGCGTCAAAAAGCCAACAATAA

>OR32

ATGGATTTCATCAGTAGAATCGACGCTGAAAGTGACGATCAAGTGATGAAAACTATCAAGAATGAATACGGCTATATGTTGCAGATAGGCGCTTTGTTTCCAAATTTACGCCCATCATGGCGTTTTCTTTCAATTATACAAACATTATTTTATATTACAATTACAGTTTACCATTTTTTTATGCTGATGTATACAACAGCGATAAATTATAAAGATTTAAACAGTGCTTCGCAGACTTTACATTATGCAGCTTTGTTGTTGATAACCATAGCAATTCTTTTATCATTCCCTTTAAATAGACCGGTATTTGTTCATATGATCAGAATAGTAGGCAATAACTACTATACATACAGTGATGGCTTAAGATCTGATTTAGTAGACAAATGGAATAAAGATAATAAAAAGAATAAAATAGTATTTTTACTTTTGGTTCCTATATATTTATTTTTCTGTGCGATAAGTCTAATGTTTATAACGCCCATGATAGATTCCTATCAAGGATTTACTGTAAACGAAGATTATATCGATAATTTATACACAAAACTGCCAGTAAAAGTTTGGTATCCGTACACAATTGATAACAACATAAAGCATATGATTACACTAATGTTGCAAATAAGTAGCGCTGTAGTTGCTGCTGCGGTTATATCTTGTGCAGATCTGTTGATGTTATTCATTAGCCAGGGAATTGCAGTGCAGTTGCAAATACTACGCCATTCGATACAAAATTTGGAAACAAGAGCTGAAATGTTGTATGAGAAATTATATAAAAATGGGCCAACATTAAAGAAAAAACTACGCTATTCCAATGAGCTCTATATGAAAATGATTAATCTTTGTATTAAGGAAAACGTCCAACATCATAAAATTATTCTCAGTGCATTCAATGACTGGTACTTACTTCAAAAGTGGCCAATGGTATTCGGGATACTAGAAGGATCATTAATAATTGCTCTATCAATTCTGGGATATCTGCTGGGAGAAGCAAGATTTGGATCTCAATTGCTCTCATTGCTTTTATTATTCGCTGAAATAGCAAACATGGCCCTATTCTGTGGTGTGGGGCAGCAAATGACAAATCTTAATGAAGAAATTGTTGAAGAATGGTATAATTTAAACTGGATGAGATGGAATAAAAGTTGTAAATTGTCTTTACTCATAGTTAAAGAAAGATTTAAAGTTCCCTTAGAATTGAAAGCTGGCGGTTTAAAAGCATTGAATAGAGATACATTAGGCAGTATATTGAATGGAGCATATTCATATTTCAACTTAATATATGCATACATCGGTGAACATTAA

>OR33

ATGGACTATTTCACGGATGATAAAGATGTAGAAAAATTCTTAGTTGAGGAGTATAAATATTTGCCTTATCTAGCTCTCACTTATGCGTCTTGGAACAGAAAAAATGTTCCAATATCTTTAGCACAATACATTATTTACTTCACCATTTTTGGATTCATGATATTTGCTTTCACTTACACGGCTTATCTTTGTCTTGACGATATCGCTGTATGGTCGCAAAGTATTCATTTCGCTATAGTCGGCATTATATGCTGGACAATTTGTTTAACTGGATTTTTCTCAAGAAAGGAATTTATAATTTCACACCGTAACATGATGAGCAGTTTTTGCGAGTATCAATCAGGTGATTTCCTAAAAGGTTTGATTAGAAGAAGACAAAAAGAGAAAAAAATACTGCTTATATTTTGGCCTTGTTATTATGCAGTTTGCGCCCTGATTGCAACGATAATTGCACCAATGATAGACATTTATATCGGTGTAAATTCTGACGTAACAAACAAAAATGGCGTATTTCTTAAATTGCCATTAGCACTATGGCTGCCATTCGATGTAGATAATTCTATGGTGCTCTATTTGGTGGGCGTTTTTCTTATAGGAGCATATTGTGCTATAAGCGCCTTATCAGTATCGTCAGGAGTTTTAGTTGCTACTTTTATCAAACAACAACTATGCCTACAATTAAGATTGCTCAACTATTCACTTAAAGAATTAGAAAATAGATCTAAAGATGAGTACAAGCGAAGATCACAAAATCCTGATCTGTATAATGATGAGGAAAGGAAGTATTCCAGTTGTTACAAAGAATGCCTTAAACAAAATATCAAACATCATCAAAACATAATCAGCGCGCATGCGCAGTACACTTACTGCACAGTAACTGCTTCCTTTTTCAATGTATTATCAGTTCCCGCCAGTGGCTACATGTGCTACGTTTTTAGCAATTTTTGTTACTTTTTTTGTTATTCTAAATACCTAACTTTCAAAATATGGATCTTCGTTTTAATTATACTTTTTAGATTCTGCACGATTTTTTGCTATATAAAATATTTTCTGGGTGAAGAAGTTCGAGTAAGTGCTTACTCAATAAAATGGTACGATCATGATGAGAATGTTAGAAGATCAGTTTCCATTATGCATTTTGGATGTTTAATACCATTGGAATTTAAGGCAGCCCATATTTTACAACTAAACATGCAAACGTTTGCTACGGTCATGAATTCTGCATACTCTTATTTCAATGTAATGTATGCATTTAAGGAATAA

>OR34JOI

ATGGAAGGAAAAATGATGTATAACTTCCAAAAAACCAGAAAGGTTCTGATGATACTAACTGGCGTTAAAATAGTAAACAAACCAATGCTCAAACAGCTGAACATACTTTATCTAATACTGCTGCTTATGTCGCTGATAGGCCAAATTATATTGGCCATTTTTACCATTGTAATGTCAGTAAACAATGTTTCTATGATCCTGGAAGCAATTCAATTTATTGTCTCATTATTGATCATAACATTCGGCCTGGCGAACGCTCTTTACCACATAAAGGATCTTGACGAAGCATCCGAACTCATTGATAACGGGATATTTATCTACCCAGAAGAAATAACCAAAGAACAAGAAGAAATCAAATTGAAGGGTGTGCAAGAGATAACATTTATGACAAAAGTATTTACTATTTACATCATATTTTCAGGTCTGTCCTATTTGCTCTTCGTCCCACTGAAAGAGCTAATCAGTCCGAGTGTAGAAAATAATCGACCAATCAATAAACTATTACCAATACCACTTTACATGCCATTCAATACGGATGATATTATGGGTTTTACCATAGCTATTTTCATAAATTTAATCTGCATGTACTGTATAATTGCACTTATACTATCTATTGACGAAGTATACATAAGTACCATGGTACAGATCAGAGTTCAGTTCCAATTGTTAAACTACTCACTGCAAAATTTAGAAAGTAGAGCCATTAAAAAAATGAAATTACTCACCAATCAAGTAGAAAATAATGAAGAAGCAGAGTTAAATGAAAATTTACTGTTCAAAAAATGTACTTACCTCTGTCTGAAAGAAACAATCAAACACCATCAACAATTGAACAGGTATATAAAAAAAGTACAACCATATTTGGGGACCACATTTCTAATCATTGTCGCATTAGCTTCTTTGATCATTGCTGCTACTGGCTATCTAATCACCGCGTACAATAACACAGCTGAAGAACTCTTAAAGTTCTGTATTGTCCTACTGTCAGAATTGTTATTTGTTTTACATTTCTGCTGGTTTGGAGAAGATGTTGCAACTGAGAGTGAAAAAGTATTTAACTTTATATACGAAACTAATTGGTACACTTACGATAAAAAGATTACAAAATTAATTGATATTATGTTGGTAAACTGTAAGAAACCATTCAGACTTCATTCTAGCACATACAAATTGCCCGCATCTTTGGAGACATTTAGTACGATTGTCAGCAAATCGTACACTTATCTGAATCTTCTAAGGCAGGCAAATACATGA

>OR36

ATGGCACCTCCAGAGAACGATCGGTCTTATTTACGCAAAGGTTACGATAAAGTTGGCGGATGGATGCTGAGATCAGCAACTGTGTACTCCTTGGATATTATCTCTTCTACCAAAGAGAAATTTTTATTTTGTATTCAACAAGCTGTACATATAATAAGTCTGGTCATAATGTGGACATTGGCCATAAAAACGATTTATATTTACAATGATACTAGCGCCATTTTAGAATTTCTACATTTACTATTTGGTGCAACTGTACTTTGGGTGCAAACTTTAAATTACATTGTGAAACGGGATAGTATGGAGAATATGATGCTTTATATTGGTAAAGATTTTTACGATTATCCAAATGAAGATATGTCCGAAGAGAGGGAAATAAAAAAAAATACTTTGGATTGGTATGAAAAATTTTGGAAATTTTGCTTGATAATTACTGGATCCATATGCTTTTTATTTATAATACGTATTGCATTGATGTTTTTTTTTCAACATCAAGAATATGATTTAACAAAAGAAGCTCATCCATATCTTCTGTTAAAGGTCTACTATCCGATGGATTTAAACAATCCAATGTATCTAATTTTTGCAGTAGCACATCAACTCGTCATAATAGCTTCGGCAGTAGCAGTTTTAAATGCACAGGCAACTGTTTTTATTAGATTTATTGGTTGTATGGTGGCAGAATTTCGAGTGTTGGCCCATTCAATAGAAAGGATGGAATCTCGAATACATTTTTTATGTAAGCCGAACATTGAGACCGAGAAAATCGGTAATAAAGAATTGTATGTCAAGAAATTCCATGAGTGCAGTATAGTTTGTTTGAAAAATAACATCAAACATCATCAACAGCTTGTGGATTTCTTCATCAGTCTTCAGGATGTCCATGGATTTGATTTAAGTGCAGCATTCTTAGTTGCGGTCATCGTCTTGTGCTTTGGATCGTTCATGGTTATTGTGGGATTAAAGAGCAATTTAGCAGACTGCTTGGTAACTATTGCTCTGCTGACTATGGAAATGACGAACATCTTTTTTCTTTGCTACTTAAGTGAAGAATTGGAAACTGAGAGGCTAAAATTTTGTGAAAGTCTACAATTTATGAATTGGCGAGACATGTCGCTGAAATTTAAGAAAATGCTGATTATTATCAATGAATGTAACAAGAAAAAGTTTTTATTGAAAACTAATACAGGTGTGACCGCCTCAAGGGAGACATTCTCATCGATTTTGACCACAGCTTATTCGTATGTTAATGTTTTAAGAGAGTTTTTTGGGTAA

>OR37

ATGGCACCTCCAGAGAACAATCGGTCTTATTTACGCAAAGGTTATGTTAAAGTTGGCGGATGGGTACTGAGATTCGGAACTGTGTATTCGCTGGATATTATCTCTTCTACCAAAGAGAAATTTTTATTTTGTTTTCAACAAGCTGTTCATATAATAAGTCTGGTCATAATGTGGACTTTGGCCATAAAAACCATTTATATTTACAATGATACTAGCGCCGCTTTAGAATTTCTACATTTAATATTTGGTGCAACTGTACTTTGGGTGCAAACTTTAAATTATATTGTGAAACGGGATAGCATGGAGAGTATGATGCTTTATATTGGTAAAGACTTTTACGATTATCCAAATGAAGATATTTTGTCCGAAGAGAGGGAAATAAAAAAAAATACTTTGGATTGGTATGAAAAATTTTGGAAATTTTGCGTGATAATTACAGGATCCATATGCTTTTTATATATATTACGTGTTGCATTGATGTTTATTTTTCAACATCAAGAATATGATTTAACAAAAGAAGCTCATCCATATCTTGTGTACAAGGTCTATTATCCGATGAATTTAAACAATCCAAATTATCTAATTTTTGCAGTAGCACATCAACTCGTCACACTAGCTTCGGCAGTAGCAGTCTTAAATGCACAGGCAACTGTTTTTATTAGATTTATTGGTTGTATGGTGGCAGAATTTCGAGTGTTGGCCCATTCAATAGAAAGGATGGAATCTCGAATACATTTGTTATGTAAGCAGAAAATTGAGAACGGAAAAATCGGTAATAAAGAATTGTACGACAAGAAATTCCATGAGTACAGTATACTTTGTTTGAAAAATAACATCAAACATCATCAACAGCTTGTAGATTTCTTTATCAGTCTTCAAAATATCCATGGATTTGATTTGAGCGCAGCATTTTTAGTTTTTATCATCGTCTTGTGCTTTGGAGCGTTCTCGCTTCTTGTGGGATTCAAGAGTAATTTTGCAGACAACTTAGTAACTATTGCTATTCTGAGCACGGATTTGTTGAACATTTATTTTTTTTGCTACTTAAGTGAAGAATTGGAAACTGAGAGGCTAAAATTTTGTAACAGTATACAATTTATGAATTGGCGAGACATGTCGCTGAAATTTAAGAAAATGCTGATTATAATCAATGAATGTAACAAGAAAAAGTTTTTATTGAAAACTAATACAGGTGTGACCGCCTCAAGGGAGACATTCTCATCGATTTTGACCACAGCTTATTCGTATGTTAATGTTTTAAGAGAGTTTTTTGGGTAA

>OR38

ATGCCTTTTTTAGATAGTTTAATTACAGAAGATAAGGAAGAATTGCATACAATTCTTGATGAACAATATGGTATCTTACTCAGGATCTCCTGTATTTATCCACGTCTTAATCCAGAAAAACGGTTGAAGAGTATTGTTCACTTTTCCATTTTCTTTTTCATATTCATATTCGATATAATCAGCAAAGTGATATCGCTTATCTTAGTAAGTGATAATTTTGATTATATAGTCAAGGTAGTTAACGGTTTGTTGATAATGTGTATTATAATGTCTTGCTTTGTATTCTATAACGTTCATAGATCAAAATTGACAAACTTATTAACACTTATGTTCGATAATAGTGTACAATTTGACGCTGTAACTGATCAATTTAAAAAAAATCGATTAAATTACAATAAAAAGTACAATAAGACGGTATTTTATACGTGTCTTTTTTATTTCACAATAACTGATACAATAATAACAATAGTAATACCATTGGTTGATTACTTTAAAAACAATAGGCAAGAAGTTTACACAAAATATGGTGTATCATTAAATCTGCCCAAATTACAATGGACACCATTTGGTAGTGATAATTATTGGAATTTTACCATCAGTTGCTTAAATATAGCTGTTGCACAGATAGTGCATATTTTAATCATCTCGTTTAGTGTACCATTATTTTATTCACTTGTGGAACATGTTCGTTTAGAGCTAGATACACTAACTTATGCAATAAATAGATTACAAGATAGAGCTATAATTCTCTATGAGCAGCTATACGATGAGAGCGTATCTAGTTACGAGCTAGATAACAATCCAGCTCTACTGAATTGTCAAGTACAATGCGTAAAAGAATGCATCAGAAAACATCAACAGATCATCAGGGCAGTTTCTGACCTGAACGTATTGGGAGGAGTGGCTTTATCCTTCACTAGTTACGCTACTGCCACTTTGATTGCGATTGCTGGAATATCTATGATATTTGGAAGTGGAGATACATTCATGTCCGGACTGGCCTTTGGTATAGCGGAAATTGGCATATTTTTCGTAATTTGCTGGTGTGGAGAAATCATCTCTGGCGCTAAGAACAATTTTGAAAAAGCATTATACTCAATAAATTGGTACACTGTGAAGGATAAAGACAGTGCAAGAATGTTGCAGATCATGTTAGCAACAGCTCAACAGTCAAGGACATTATATTTTAACAAGAAACTTCATGTGCCAGCAAATTTTGAATTATTTGCGACCATGATGAATACAGCCTATTCTTATTTCAGTTTACTATTTCACATCAAGAAACAAATTTAG

>OR39

ATGAAGGACGATAAAGAAGTGGAAAGATTTCTAATTAAAGAATATTTTTACATACACAAAATTGGCCTGGCTTATGTGCCTTGGGATAAGGAATCAGCTATTATATCAATTTTTCAGTATGCTATATACTTAATTGTATTATCTATCTCTACAGCATTGCTTGGTTACACTGCATTCCTAACTATTGACGACATAGCCTTGTTCTCTCAGGATTTGCACATTACCCTGATTTGTGTGGTGGCCATTATTAATATAATTAATAACTCTATGATATATAGAAACGATATGGTTGAAATACACAAAAATCTAATGAATGGCCTGTGTAATTATGAGACCGGAGAGTTTTATGAAGGAGAAAAAAAAAATATATTCAAGCAAAAAAAATTTTTACTTTTATTCTGGCCACTTTATTATGGATTCTGCGCTTCTATGGCGATTGTAATTGGACCTCTTATGGATAGTTACTACGGTGTGGATGCTGTTCATACATCTTATCCTCAAGTTTATTTAAAATTACCTTTTGCTATGTGGACACCTTTTGATATGGGTAGCCAAGTACCTTTACACCTAATTTTGTCTGTATTCCAAGCTTTCATGGTTATTATTAATAACTTAACTATATCCGCTGGTGTAGCTATGTATGTTGTTGTATTAAACTATTTATGCCTGCATCTTAATCTACTTACAAATTCAATTGACAACTTGGATGTGAGGGCAACGGAGCTTTACAAGGTGCGCCAAGGAAATAGAGCTTCAAGTAAATTAGACAGAGTGCTTTACAACAAATGCTATAATGACTGTCTAATTGAGAACATCAAACATCATCAAAGTATTCTCAGATTACACAAGATGTATGTTAACCTGTCGAGCGTTCCTTTATTCGTACCATTCATTGCTGGAGCTGTTCTGATGTCAATGGCAGTAGTGCACTTTATGTCGGATGATGTGAGAATTGGGCCAAAGCTTACTTCAGGAACTGTAGTCTTTGGAGAAATGCTTAACATGTTAATGTTGTGCGTTTATGGAGAAAAACTAAAACTTTTGAGCGAAGGGGCCAGAGATACAATTTATTCTATAAGATGGTATGAAATGGACAAGGAATGCCATCAAACTGTGTCTATTTTTCAACAGGCCACTTTAAAACCAATGAATGTAAAATGTGTATACCTGCTTGATATCAATATGGAAACATTCGCTACGTTAATCAATTCTACCTACTCTTACATCAACTTGGTGTATGCTTTCAAATGA

>OR40

ATGGTGAACATTGAAGAGTATTTTGAATCAACTGAGAAATTATACATGTGGTCAGGTTTTCAATTATTCCAATCCTCTGGTCACATACTAAGAAATAAAATTAGAAGACAATTATTGATCTTCATAATAATATCATGCGATTCGCTATTACTAGCCGGAGCTGTATTTTCAGAAGATTTAATGGACATGACTGAATCGTTATTGATTTGTATAATAATATTCAACGCAGGCAATAAATTTATCAATATACTGATTAAAGAACTAGAAATGGTAAAAACTTTTGTTCAATTGCGTAAATTGGTAGAAGATCAGTTGAAGGTAAATTGGAAAAAAGAAATACTTGTCAACAGTAAAGCTAGAGTTGATTGGTTACAGAAAATGTTTTTATGGAATTTTGGTATTTGCGTAATTGCGGCTATTTTGAACACCATTGTAGACAATATAAGACATCCTGAGAATTTAAGGTTCCCATTAAAAATGTGGTTTCCTCTAGCACCTAACTCAGGGCTTAATCTTACCATTGGAACCATTTATCAAACTATTTGTAGCTGTGCTGGTATTATTATATTTACATTCGGATATATGACTTTTACTGGATTAATTATACATATATCTTCGCAAATTATTATCTTATCTAAGGATATGAGAAACATCTCTGCCAAAGCCAATGTAAAACAAAAGGAAATGCTAAAGAATTTTATAAGACATCATCAAATCATTCTAAGACTAGTTAAATCCACAAACAATGTTTTAGGAAATGTCCTATTCTTAGAGATAATATTATCTTCAATGCAGACATGTTTAGTAGCATTCAATTGTTTCCAGAGGCCATTAAATGATCCGAAACAAGCCTTCTTCGTGCCATTCCTCCTCTGCACAACTATACTGCCGGCCTCGATTTGCTGGTGCGGACAGCTTTTAATGACAAAGAGTGATGATTTAATAAATGCTATATACGATTGTGAATGGTATGCTATGCCTCCAAAGATTCGTCGTGATATTTTGATGATACAAATTCAAGCATCAAACTCGATGACATTAAATGCTCGCAATACTTTTGTAATGGCCATGCCTACTTTTCTCAAAACTTTGCAGACAGCTTACTCTTACTTGACTATGCTTACAGCTACTGATAAGTAA

>OR41

ATGGTGCTTAAGGAACTGGATCTTTATTATCATTTATTTCTAGTTTGTGGGCTTTGGTATCCAGTTCCAAAAGATTTCTTTAGTACAGTGGCGTTTATAGCGCAAATAATAAGAAATTTGTTATTTATTTTGCCGTGGTTTGTGTGCATAGTTAGCATATATTTTGAAGGTTTTAAGGAAAGTATGTCTGGTACAGCTTTCTTCTTTGTGATTGGTTTAATTAATAATGTAAATGAGATCATTTTTTACTTTAAAAGATATGATACCCATTTAATTATGAATATGATTAAGAGATCCGTGAATGGTCACAGCACTATGTGGGAACAGGAGTTCATAAAAAAAAGAGCAGCAATTTTATTTAAATTGGTGAAAATATATATTTTAATAATGGGTTCGTTCACCGTGATTGTGTATATGTTTTTACCAACGTTGAATGCTTTTTTGGAGTATTTTGATATTAAAACATTGAAAATACCTCTACCGTTTAGAGGCTTCTCACTAGGGAAATTGAAGAAGGACAACCTGGACAGATTTCTAGAATTTGGTGGTCTAATGTGGTGCTACTTGGGAATAATGTTTTACATTAATATGCAAGTAGCATTTTTCTTTTTCATTCATTACACGGCCACAGAAGCAGAACTGGTATGTGAGAAAATTAGACGTTTAAGATTTGTCCATGACGAACGTAATTTCTTGAGCGATAAAGTGTCTAAAACGCCAGATATTATAACACTCAGAGAAATTATTTTGCATCATCAATTTGTATTAAAGTTGGCCAGAGAAATAATTAATTATTTCGGATTGTCAATGGCTGTGCAGAATAACATTGGTGCTCTATGTATTTGTTTGAACTTAATATCAGCACTATCTGAGCTGCACAGAGACCATTCGGTTGTTATTGTCAGCGTAATGACCACTGCATGTATGGTGGCCCAGATGTTCTCTATCTACTATTTTGGACACTATCTAGTGGAACAGAGTAATATGATTCATATAAGATTGTACCAGTTACCTTGGTATAGATTGACTCCTTCCACGAGAAAAGGTCTTATAATGATATTCATGCAAGCTGTTAAGCCAATTGAAATTAACTATCAAGGTGTGGCACGAATTAATTTGCCTACATTTACGCAGGCGATGAATGCAGCGTATTCTTATTTCACTCTCCTGAAGTCTACGATTGATGAATAA

>OR42

ATGGCGTTTAAAGAAATGAACATGTTTTATCGTATATTTCATATGTTTGGTGTCTGGTATGCTCAACCAACAGACCTCTATGGGACTCTGAGTTTTATTGGACAAGTGTTCCGGCATTTTCTGTTTATATTATCCTGGTCTGCAGAAGTAGTGTCTGTATATTTAGAAGGATTTCGACGAAGTATGAACGGAAGTGCTTTCTTTCTGATTATTTCATTTGTAACCGTTTTTGAAATAGTAATGATTTTCTTAAAGAGAAAAGAAATTCACTGTATTATGAACATGCTGGAGATGTCGGCAAATAATCGCAGTGGTTGGGAAAAACAGTACTTTGACCAGCGAACAGTTTTTGTTAAAAAAGTGGTGAAAACTTATCAAACAACAATGATAGTTATTGGCCTGCTACTGTATTTGGTGTTTCCAATGGTGAACGAGTTGCTAGAATTGTATGGCGTCCAACCAATGAATGTGGCATTGCCATTCAGCGAACTATCTATTGAGGGACCGTACAAGAAAATACTAGAGAAACTAAGAACATTTTGGGGCATCATCTGGTGTTTACATGGGATTACATTTTTCTCTTGTGTACATAGTTTGTTCTTCACGTTCACACTTTATACGGCCGCCGAAATTGATCTCGTCTGTGAAAAGATTAGACGCTTAAGCTATATTGACGATGTGGAATATTACAAAGTTGATAAGGCCAACAAAAATGGAATCTCGCTGCTAGAAATTATCGTACATCATCAGTTTGTGCTTAAGCTGAGCAAAAAGTTGATCAATCTTTTTGGACTGATTTTTGCTTTGGAGAACACGATCGCTGCTGTGAGCCTGTGTTTAAATTTAATCACTGTACGTTTTGAGATTATGAATAGTAAGCCGGGAGTTGTTATCATTTCTGTGGTATCCATGTTCTCAAATGTGATTCAACAAACCACTGTCTACTACTTTGGAAATTATCTTACTGAACGGTGTAATGTGATTTATGAACGATTATATGAATTACCGTGGTATAGACTACCACCGTCTGTTAGGAAAACCATAAGTATCATGTCTAGACAAGCATCCATGCCCACAGTGATCAACTACCGTGGTCTAGCACGGATCACACTGGAAAACTACGCCCAGTTGATGAACGCTGCCTACTCTTACTTCACGGTGATGAGCTCATTGCTGGACGAGTAG

>OR43

ATGAACAAAATACCCTTGGAAATGATAGATTTACTAAAATTTGGTGGATTATGGTTTAATTATTCAGGCCATAAATATGAGAAATTAATATATTTTGTTCAGTTTTGCAGATTATTTGTTATTCTGTTTATATGTATGCTAACAATTATACCAATCTTTACTAAAGGAATGAACTATGTAATGTCTGGAAGTGCAGTATTCATACCATTGGGCTTAATTGTTGGCTGTCTAGCCATAATAACTGTGGCTGGATTTAAAACTGTGGAAAAAACTATATACTTAACGGATGAAATTATAAATTCCCATAAAGAAGTTTGGGAATTGAATGTAATAAATATTGAAGTAGATAAGGCTAGAAAAATTGTAGATATTCTAAAAAAATATCTATATCTATTCGCAGTATTCTACATTTTAGCACCAGTAATTATTGACCTATTAAGAGCTCAAATGGGCTATTTAAACCCATATAAAGTTTTACTTCCTTTGGACGGCTATCTAGATGAGATTATCGTAAGAGATTATAATTACTTTCTACTGACGTTTCTATCATTTATTTGGTTTGTTTTGATCGTCGCAAATAATGTAGCATTTGAAGGTTTGGTATTCTATCTTGTATCACTGGCAATTGGTGAAATGAAGATAATTAAAATGAAATGTGATTCATTGCGTGATGAGAAATACGAATGGAATCTGGAAGAGATAATCAACAATCATCGTATGCTATTGAGGTACATTAACTACCAGAAAGAGAGCATTGGATTGCAGCTCTGTATGCAGAACGTATTGTCAACTATGAATAATTGTTTGGTTATGTATTTAATTAATAATACCTACGATACCGATAAAATACTGTTCACATTTTGTTGCCTATTTTTAATATTTGTCGCAGTACTTTTGGGAGCAATTTGTGATCAAGGAGAAAGTGTACAATCTGAGAGTGAAAAAATGTTCCAATCTATTTACAATACACCTTGGTATAAACAAAGTCCAAAAAATAGAAAAAGTATAAATTTAATGTTGTTGCAAGCTTCAAGACCGTTGGTGTACGATTACAAGAGACAATCGCCAATCAATCGTTTGGCTTTTAGAATGATTATTAACGGATCTTATTCTTATTTTATGCTCTTAAAATCTTTTGAATAA

>OR44

ATGTTCACAAAGGAAATAAGAAATACAATTAAACTTCTAGAATGGACCGGCCATTGGTTTAACTATGATAACAAAACTAATGAAAAATATTTAAAAATTTTCCAGATTTTCAGATTAACATATCTTGGTATTGCATTATCTTTGAATGCTTATACTATCTATTTAAAAGGATTAAAGAAAAGTCTAGAAAGCTTTGGCGTATTCTTTCCAATGACTATTATGTGCCTGTTGTGTGCAATGTTGATTGCTATAAACAATGTCAGAATGAAAGATTGCCTAGTAAAACTAAATTCTATGATTGAAAATCATAAAGAAAACTGGGAACATGAAATTTTCCAAGAAGATTCCGAATTTATCTGGAATATAGTAAAGAAATATCTAAAAACGATTATCATGTACTACTTACTATATACGCCGATACCATTATTTATAGATTTTATTGCATATAATTGTAATCTATATGATGGGCCACCATTAAATTTGCCACAAACATTGTCTGGTTTTATGGATGATCAGCCTGTACGCGATGGGAAATATTATACGATAATTTTATTTTCACAAATGTTGACAGGAATTGGGATACCAAATTATTCTGGAAATGTATTTGGCTTTTGCATAATCACTGTTTATATAAGAGTGGAAATGAAGATTATACAAAAGAAGATACTTTTATTGAATGATATAGATAATAATTTGAAAAAAGTTTACTATAGTAAATTAGTGGAGATTATTGAACGATATTTACAAATCAAAAGCATCCTTAAAGACTTGGAGGCTGTGTATGGAGCACAATTTGCTTTACAAAATGCTTTAGCACCAATAAGTACGAGCTTCTGTTTGAATCTCATAGTTATGTTGGAAGACAAATCTGTTGCTCCAGCATATCTTATATGTTCTTTAGTGTTAACCACGGGTCCTTTTATCTGCTGCTTGGCTGGGGAATCTCTACAAGAAGAGAATTCAAATATCAACAAATTAATTTACAATCTGAAATGGTACGAATGGAAAAGTCGTGATGGCAGAATGGTATTGATGATGCTGGAGCAGACCTCACTTTCTATGACCATCACCTGTAAAGGGCTATCTCCTATGGGAATGCATACATTTCAGGAGATAATGAACAAGACCTATTCATATTGCATGCTATTAAGATCACTTAGTTAA

>OR45FIX

ATGGCCGATCTGCCACCTTTAAACAACTTAATGATGATGTTAAAACACAGTGGTTTTTGGTTTTATTTCGAAAAAGAAAATAAACTAAGATTTTCTTATCAGATTTTCCAAAATCTAAGCATACATTTGTTTCTCTATTACAGAATGTATTTAGTCTATGAAGATGGTATAATGGCATTACTTGGTGACAAACATATCTATATTGTCAACACTACTGTGGCAATGTTCATCAATAATCTCCTTTATATTTTTAAACAGAAAAGGATTTTGGAAGAATTGTTGAACACCTTGGGAAACATTGTCTACAAAATATCCAAAGAAAGGCACATGTTTACCATGAATTATGAATATGCTTGGAAATTTGTAAAATATTTTGGTTATTTTGTGATAGTCGGTACGTTGTTCATGTTTATGTCACCGTGCATTATCGACGGTGTCTACTATCTATTAGGCTATGATGAATTCACGCTACATCCACCGCCAACGGTGCTTCGTTTAAAGGGTATGCCAGAAGGACGTTTGCATAATTGGTTGTGCTTACTGATCAGCGGATATTATGCTTCAATTTGGGCTGAATGTTATTATTCATTAATTATCGCTTGTTTTATTATATTATCGTTTGCTTATATTGAAATGAAAATCTTTAATTATCAGCTAGCGGATTACTTAAAAAATCAACATTTACACGATTTAGAAAACAGTTTCCCTGAAATTATCACCAGACAATACGAGATTATGAGATTACACGACGAGTTGATAGAAGTACTAGCAGGACCAATGGGCGTAAAAAATACTGTAATTGCTGCTGCAATGTGTTATTTTCTTTTTACTGCTACTGCTGCCAGGAGCACGTACGAAGTGATCATGTGTGGCTGTGGTCTGATATATTTTGTGACAGACTCGTTTCTTTCCTGCTACGTGGGAGATCTTATTGAGTCCGAGGGTCATGAAATAAGTGAAACTTTATACGACTACCCATGGTATACTATGAAGCCAAAATATCGACGCCAATTTTCATTCATACTTCTGCAAGCAAGGAGACCAACTACAATCACTTTTAAAGGAATTTTACCAACAAATCTAGAAACATTTATGAAGATTGTAGAGTCAGCTTATTCATATTTTATGCTTTTAAAATCTTTTTAG

>OR46

ATGACCCCATATGAACAGCTATTGTTCTGTCTTAGCTTCGGCGGATATTGGTTTAGATTAAACGATAAAAATAAGCTGAAAACTTTTCTACACATTCTACAAAATATTATATTGTATGCAAACGTAATCTATGAAATAATAGGGCTATATATTGAAGGTGACCTCCAAGAATTGCTAGCTGGTAAATTTCTTTATTTACCAGTATCAATTTTTGAATTAACCAACCATCTAATATTGTTTTGCTTACGTGATGATTACGATCGAATGTTGTTAACTCTTCAGGAAATTTTTGCGAAGGATTTGGAAGAACGGAGAAAATTTCAACAATACTACAAGTATCTAAGATGGGGAATAATTGTTTATGGAACAGCACAGTTTATGTTATGGTTTGTTTCATTTTTTTTAGTTTTATTTAAAGAAATCATCGCAGTTATATTCCGTTTAGAAGAATTTTCTTTTGTGCCACCGATGCCTATTGAAAATATTATTGGTCTTGAAGAAGGTCGTCTGCATAATTGGCTGAAACTAATCTTTGGCTTTTTGTATGGTACAGCATGGATATTGAGTTACACCAGTATTATGATGGGAACATTTCATTTCATGGCGTTTACCATTATTGAGTTTAGAATTTTTAGATATCAACTGGAGGTTTACCACAAAAATATGTTGATTGAAAATTTTGAGCATACAATGAAAACCATCATAGAGAAACAGTGTCTTCTTGCAAGATTATTTGAGAACCTGAAAAATACATCTTCACTGTTTCTGGGTTTACAAAATTCAATGATGGCGCTAAATATTTGCTTTTTACTGCACACTTTTACCAAGATAACTAATGCTATTGCATGGTACATTGGTTTATACGGCTTCATAGGTTTTACAATGATTATCACTGCTACCTGTTATTTTGGAGATCTTTTGGAAGATGAGGGTGATGCCGTCAACAACATATTGTACGATCTGCCCTGGTACTCTTTACCACCAAAACAAAGAAGATATTTCGCTTTAGCGTTACTTCAAGGAAGAAGATCATTGAAAATTTCTTACAAAGGAATTTTACCAACAAATCTGGAGACATACTTACAGTTTATGCAAGCTGTCTATTCGTACATTATGCTGTTGAATTCTGTATAA

>OR47

ATGGATCATCCACTGAAGCAACTGGAATTTGTATTGAATGTGTCAGGATTTTCAATAGATTTATCTAGTAAAAATAAATTTAGAATTGTTTTCCATATTTGTCACAATTTCATTATGATGATCTTTGGCATTTATCTAGCCATATTGTTAAGCGTAGACGGCCTGAAACAATTAATCACAGAAGAATACACATATTGTGCACCTATCTATTTAGTAACTATTACACATTTTGTTATTTTTCTACAAAGAAAAGAAATTAGAAAATTTGTACAAATCTTCAAGGAAATAATGCCGATCAATGATAAAGTAAGAGATAATTTTAAAAATATTTACAAGAAAACTTGGAATGGTGTTAAACTTTGTATTCTTGGGCAAACATTAATGCTATTGTTGGTATTTATCTGGGATATAATTGATGAATTGGTAGCATTAGTCTATGGCTATGCAGATACCACTGAAATACCACCAATGCCCATGGTACGTGTGATTGGACTAGAAAAAGGTTTAACACAGAAATGGATAATACTGTTAGTTTCAACTATTTATGTAACATTTTGGAAATTTAGCCTTTTTGCGTATTTAGTCGGCACAAATATGATGTTAGTATTCTCATTGAACGAGTTGAAGATGTTCAACTATCAACTGAAAACTAATTATAATGAATTCTTGATCAAAAATCCAAAGATTGTAATGCGTAAAATGATAAAAAAACATAATGAATGTTTGAGATTATTTATTGCTTTGAAACATGCAACTGGCTTGATTTTTGCCGTACAAAATTCACTTATGGCTCTGGGCATTTGCTCATTGATGTATACAGCGACCACTATAACAAATATTAAAACAGCCATCCTTTATGTTTATGGATTATTGGTATTCAGCGGATTTACACTGATACTTTGTCATATAAGTGATCTTATTGAATCAGAGGGTACGGACATCTATAACTCACTATATGAACTTCCATGGTATCTTCTATCTCCTGACAACCGCAGGGATTTAACTATGATACTGATGCGAGCAGGTCGTCCATTGAAAATTGATTACAGAGGTATCTTGACCACCAATTTGGAAACATTTCTACAGATAATGCAAACCGCTTATACTTACTACATGCTGCTCAGAACCATGTAA

>OR48

ATGAATAGAACTGCTCACTATATAGATCAGTTAGTAGCATTATTAAAATTCTCAGGTTTATGGTTGTACACCAATGATTTGTGTAACATCAACTTCTACACGCCTATACTTCGACTAGGTTACATGTGGTCAATATTTGTGGCAAACGGTGTAAGTGTCTATCAAATCGGTTTAAAAGAAAGTCTAAAAGACATTGCCATCTATATTCCAGTCATGGTCTTAATTAATGTACAAACATTAACTGTTTTAATCAAGAATGAACAGATGAAAAATCTGTTAAACTTATTGAAGATCTGTTTTACTCCATACACGGAGCATTGGCAATTTGATTTACTGGAGAAAGCCAATAACTTTGGTTGGATCATAGTCCGGGTGTACAAAGGTGTAATGCTATTTTTCTACATATTTTACTTCTTACTACCCACATCCGCAGACATAATCATCTACTTCTTGGATTACGATACAGATACCACTTATTCCTTGCCATCACCTCTGACTGGCTATTTAGATAAACATCCGGCTAGAAGTTTGAAGAATTGCATAGTACTATCTTTAGCTTTTCTATGGGTCACAATAAATTTCCTAAGCAATGTTGGTGTGATGTGCTCATTTTTCATTATTATCCTATATAGTCATACAATATTGAAAATATTCAATATGTATGGTGATCGATTAGATTTCTCGTCCACTAAAACTATGAGATCATCAATAATCGAACTGATACACATGCATCAAAATATCCTAAAGCTAAGCCAAGGATTAAAAGATTTTTACGGATTCATATTTAGTTTTCAAAATCTACTCACATCTGTCTGCCTCTGTCTTTGCCTATTTACAGCAAGTACGAATAAAGATAAAGCAATTGTACTTTCTTACGGTTTTGGTGTTATCTACTACATTTCTTTGTCGTTTATGTGTAACTTACTGGGCCAGTTTATTCAAAGTGAGAGTGAAAATGTTATAGTTTCCATTTCTAACATACCCTGGATTCATATGAAAGCTGCCTTAAGAAGAGATGTTAACTTAATACTACTCCAAGGGAAAAGGGAAATAGTGATTTCTTACAAAGGAAGATCGCCATTAAATTTAAGGACTTTTATGAGTATACTGAACACATCTTATTCGTACTTCATGCTACTGCGTAGTGTCGCATAA

>OR49

ATGAAGATGGACCCATTGAACGAACTACGTTTCACCTTTAAAGTTGGTGGATTTTGGTATTATTTTTCAGAAAGAAACGTATTTCAAGTAATCCTACAGATTTTGCACCAAGCTGTACTTAATTTCGCCTTGTTTTTGAATATTTACTGTATTTGTAGAGATGGTTTAAAACAGAGCATGGGAAGTACAATGATCTATTTACCTCCTGCTATTGTTGTCAATGTATGTCATTACGCATTGTATTTCAGACAGACAGAAATAAAAGAATTATTAGATACTTTGGAATCTGTGATGTCTAAAAATAAAGAAGAAAGAGAAATTTTTAAAAATGATTATTTATTTGCAGTTAAAATAGCAAAAATTTCCAGATATACTTTATTTCTTATATGGATAATGTTCTTTACAACGCCACAAATTATGGAATTAATATTTTATCTTGTGGGTTTAAATGAATTTCCTGTCTATGTCCCATCTATTATAAATTATATCTTTATGGAAAAAGGAAGACTACAGACTTGTGTTTGTGGACTGTTTGGAACCTTGTGGACAGCTTGCGCTATGTTCAGTCATTATGGTTTTACAATATCAAATATTACTCTTTTAATATATTATCACATTGAACTAAAAATTTTTAAGCATAAAATAGATTATCAGTATGCAAAGTTTTTTGTGTCTCATCGAATGTATTATGTATTCAAGGATATAATTTCTGTTCAACAAGAATTATTGAGAACTCTTCAAGTTCTGAAGAATGTGGTAGCTCTGGCTCTTGGTTTCCAAAATGCAATCATAGCTCTTTGTATTTGCTACTTCTTATATACTATAACTGTGAGTGAAAGCAGTTTCGTGAAACTACTTTTTTCCTACGCTTTTATGTTGTATTTAGCAGATGCTTCTATAAGTTGCTACGCTGGTGAGCTAATTGAATCTGAGAGTCATGAAATATTTCAAACACTTTATGGACTACCATGGTATGAGATTGACGCAAATAATCGTAGAATGTTTAAACTGATTTTATTGCAAGCAAAGAAAACAATGAAAATTGAATATAAAGGAACTGCAGCAACAAATTTGGAAAGTTTATTACAGATCATGCAGGCATCCTATTCTTATTTCATGCTCCTGAAATCAATTTAA

>OR50

ATGGAACCATCAACATTAGGATCGGAACCGTTGAAATGGTTACATACTACGTTGAAAATTGGAGGTTTTTCGATGAATTTCCATGATAAAAATAAAAAAAGATTATTCGGTCATATTATCCAAAATATTGTATTGTATTTCTTTTTATCTTACGATTTGGTTCTAATTTATTCGGATGGAGTGATAAATATTGATATCGACAAATTTATTTATTTACCACCATTTATTATAAATAGCATCCTCCATGCGACACTGTTTTTTAAACAAAAATCAATTAGACATTTACTAGGCAAATTGAAAGAAGTAATGGAATGCAATAAAGAAGATAGAAATCATTTTGCAGAAACCTATAAATTTGTAAAGAAGCTTATTAAAATGTTCGTTACTGTTCAGATAACAGTTTGGAGTTGTGTTTACATTATCCCATTACTGCAGTCAATCGCTTCTTTATTTTATGCTAAAAACTTCAAAATATTTTTACTATCACCGCTTCAAAGAATTATGGGTTTCTCACCTGGATATTTCCAAGATTGGATTCTACTAATATGTACTATCCTCTGGACCTTCTGTCTTATGTGCACTTTTTTTAGTACTTTCGCTTCTGTCGATTTGATACTCATATTTAGTTATTTAGAAATTAAACTTTTTCATTATGAAATTGAAACTTACTACACATCATCATTAAAAAGTGATTATGATTCAACTTACCGGACCATCGTTAGAAGACAAGTTCAAATTTCAAAGTTATTTGAAGCACTCAAAAATGCTGTTGGTTTATATCTTGGTCTTCAAAATACTTTTATTGCCGTAACGATTTGCAGTTCATTGTACGCTGCAACCACAGGGCAAAGTTTGTATGTAATCTTGCTTTTTTGTTATGGCACATTGTTATTCATATTCAATTCGTTTCTCTGTTGCTACATTTCAGATCGTATAGAAACAGAGGGATCCAACATCTGTAATTCAATCTATGAACTACCCTGGTATGAAATGCGTCCGCAGGAACGAAAAGTTTTCAAGATGTTTATACTAAAAGCTTCGCAATCAATGAAAATGAATTACAAGGGTATTTTTCTTACAAACTTGGATACATTTTCACAGATCATGCAAGCATCTTATTCTTATTTTATGTTGTTGCAACAGTTTAGCTAA

>OR51

ATGAAGAAAGATCTGTCAGATGTGATTCACATTCTGAAAATTACAATGATTTGGTATGATTTTACTGGCAGTCGTTTCGCTAACATTAAAATATCATTTCAATATGTTCGTCTTATTGTCAACATTTTTACGTGGTTTTCAGCAGTATTTAGTATGTATCTAGAAGGTTTTCCAACAAATTTTGATGGTAGATACTATTATTGTGCAATGCTCTTTTTATGTTGGTGCCATTCGTTGGAGTTTTGTATTAATAAGAAAAAAGTTGAAAATTTAGTTAAAAAATTTCAAGAATTAATGAAATCTCAAAATGATGCATCATTGGATGAAAAAGATCTTAAGAATATGTTAATTGCTATAAAATTTTATGTTTCAAATTTATTCCTATTTGCTGTATCGTATTATATATCTCCTGTGATAATAGATATTTGTCTAGCTATATTTTCACCTGATTCAAAAATCTTCAAAGTACCATTCTGTTTCGACTCCTTGTTAGATGATGGAGTGAGGAATATAAAATATTATTTTATAGTTATATACCAGGGTATATGGGTGATGGAGAATATGTTAAATCATGCTGGATCCATAGGAATACTCAGCATTTTTGTTATGTACCTAGGAATACAGATAAAAGTTTTAGCACAAAAAATAGATAAGTTTTCTGCTGATGTTTTAAAAAGAGATGCCTTGTACAATCCTACCTTAACTAAGAAGGAAACTGAAGTTCTAAAGGATCTGGTCAAACATCATATTGTGATTTTAAGTTTGACAGAACAAATTAAGGAATACTTTGGATTTGCTTATTTAATCCAAAATTTAGTTGCTGCTTTTTGTTTGGGTTTGACAATGATCTCGACTGTAAATTTGCTAGATCAAGAAGAGTACTTCCTGGTTGCTATGAACTTACTCTCTTGTTTTATGGTGGTTGTACTTTCATTTAGTGCTTGTTATGTGGGCGAATATCTGCATTCACAGAATACATTCATTTACTATAAAATATGCAATATTCCTTGGTATGCCTTAAACTCAAAAACGCGGAAAGATGTTGGTTTAATAATGGCACTTGCTTTAAGAGAACGTGGCATAGACTATCATGATATGTCAGCATTGAAATTGCATACATTTATGCAGATAATGCATGCAGCTTATTCATACTTCATGGTGTTGCGATCAAGGTTGTAA

>OR52

ATGGAAGGTAGACGGGATCTAGCCGATGTGTTTAATTTGCTGATGATTTGTGGTATTTTAAGTGGATCACATGGAAATTCGCGACTGATTTTAAATGTTTTCCAATCGTGTAGAGCAGTTTTGTCAGTTACAGGTTTCATAATGAGTTTAGCCTCTGTTTATAATACTGGAATAAAAGATAGTCTTTCACGGTACGCTTTCTTCTGCGCTGCTGGGTTCTTCCTTAGCGTTCAAGCGGTATCTTTCTGGCTGAATCGACAGCTAATAGAACAAACCTTCGTCTCCATTAAACGCTTGAATGAAAAAAGAATAGAACCACGGCAAATAGATAATTTTAGAAATATTTCTATTAAAGTTTGGCTAGGCATCAATATTTTTATATATTCTTGCCTTTTTTTTAATTTGGTCTATTTCGTCTTTCCATTAGGATATGACATATATTTGAGTCTTTTCACTAATTCCACTGAACCTTACGGTTTGCCTTTGCCAAATAAGTTCATTGCGGGGACGGAACCTCCTACAAGATCTTTCTCTTACTATTCTGTAACGTTATTCATAATAATTTGGGTTGGTAATCTTTGCTTCACTGCCGTAGGATATTATGCTACAATTTTTTTTCTTATTTTGTATATTTGTAACGAGTTTACTATACTTTCTGAAAATATTCTGAACTGGGGTAATTTATGTAATAAATATAATCATGGCAATGAAGCTAAACTTACTCTAAAAATGATTGTTCAAGACCATCAGGAAGTCACTAGACTTGGTCCAGCATTAAGAGAGATTCTTGGTATTCCATTCATGGCGCATAGTGTGGTATGTCCGATGTCGTTAACACTTTTCTTATATACTGTCAAAATGCAATTGGGAAAGAATGCCATTGTTGTGTTTGTCAACAGTTTCTGTATAATTAGTACAAGTTCTATGCTATTTATTACCTGTTTAGCTGGAGAGATTCTACAGTCACAAAGTCTGAAGATCTTCGAAAGTCTTTGTTCGATTCCATGGTATGAAATGTCGCCTAATATGAGGAAGGAATTGAACATGATAGTGCGACAAGCAGTCAGGCCAATCACTATTCACTATAAAGGAAGATATTTCATTAATTTACGTACTTTCATGCAAATTTTAAATTCTTGTTATTCCTATTTTATGCTTTTGAATTCAATGAATTGA

>OR53

ATGGAGACGGAAGGAAATAGCCACTATTCCTTCTTGCACACTGTGTCATGGTTACTATATGTTGGTGGAATCAAGAAAAATGAATATGGCGACGTACCAATAACATTTCGCTCTTTGGTGCTCATGGTTTGGGTGTTTGCCTTGTTCTTTGCCATAATTTTAAAACTATTTTGCTGTGTAGCTTCATTGGAACTTTTTGTAGCTGGAATTATGGTGGCTGCCATAACTTTTCATTTAGTTGTAAAACAGTTCATCTTAATTATCAAAAGAAAGGAGATTATAAGTTTGATTGATGATCTTGAACACTGTTTTATATTAGCTCGAACCGATCCTAAATTCATTGAAGAAAAGTCACTTAGTGTAAAGAAAAGTGGAAATAAGTTGATCATTATAATGGGCGTTAGCATTGTAATTGCTTTTGGCTTTCAATACATCAATCACGAGCTGACTGGAGATACATCTTTACCTGTAGAAATGTGGACCCCAAATGGTGACGAAGGAACTTCATTTACAGTGCTAGTCTACCAACTGTTCATGGGTGGGGGCTGTGCTACAGAACTGGTCATCTACTACGCTCTGTTCTTAGGTCTTGTCTCAACTTGCAGAATGATAATTGATTCTCTTAAAGGGAATTTCGTCAAAGGACCAGACGAAAAAGAAACTTTAGAAGCATTTTTAAGCAGAAATGTGCTTGCTCATGCTCACCTGCTCAAAGTTGTTGACGACATCAACGCTAATATTAGTCCAATAGTATTCCTGGAAATACTGCTGTCATCTATACAATGCTGTTGCGCTGGATATTTGGCCAATTTTGATTCATTCCACAACAGTCTCTACTTTGTATACGTTCAAGCTACTTTAGTCATGCCAGGAATGATCTGTTATTTAGGGCAGGAAATTAAAAATTATTATCAGGATTTATTCGATCAGTTGTACGACTGCCCTTGGTACATTGCAACGCCTAAACAGAGGCGTTACTACCTGATGATGATCTCTATGACTTCAAAACCTTTGATTTTACATTTTAGATTCTTGATGACGTTTGATTTAGAGAGATATGCTAATGTGCTACAAGCTGCGTATTCCTACATAACAATATTACAAAACTTGTTAAATTAA

>OR54

ATGAAAGGACAAAAAACTTACTATTCCTTTTTGGAAACCGTGATCTGGTTGTTAGCCATTACTGGAATCAGAAGAGATGAATTGGGAAATGTTCCAATAACCTTTCGTGCAATCGCCTTCTGGTTTTGGATATTCCTAATGATAATCAGTATTATTTTGAAAATGTTTGGTGGACAGACGTTAGAAGAATTTTTGAATGGAGTATTGTTTGGAGTGATTATCTTGACTGTGGCCATAAAACAGGCCAATTTAATTATCAGAAGAAAACAGATTTTTAAATTGTTGGATGATATTGAAAACTGTCTGACTTTAGCCCGAGCAGATCTTCAACTTGTTGAAAAAAGATCCTTGAGACTGAAAAAAATTGGAAAACTTATAACGATTTATATTTTAATTGCCACTGGGACAATGTTTGCATTTCATTATGTGCATCACGAGCTAACTGGAGATAAAACTCTTCCAGTAGAATTGTGGACACCCTACAGCGAAGAAGGTACTACGTTTACTGTAATAATCTATCAGCTGATCATAGATACAGGAGCTACAATAGGGTTGATAGTATTTAGTGCATTATTGCTAACTGCTATCACATTTTCTACGATGATAATGGACGTTCTTAAAAAAAATTTAGCTGAAGGGCCCGACGATGAAGAAACGTTAGAGACATTTTTAAGCAGATACGTTCTAGCGCATGCTAAACTGCTTAATATTATTAAAGCAATCAATGTTAATATCAGTCCAATGCTGTTCATAGAAACAGTGCTGTCATCTATTCAGTGCTGCTGTGCTGGGTATCTGGTCAATTTTCGATCTTTTCACGGCGCTATGTATACCATCCATTTTGTGAGCACACTAACTCTCCCAGGTTTAATCTGTTATTTAGGACAACTAATTAAAAATAAGAATGAGGAATTATTTGATCAAATTTATGACAGCCCATGGTATACGGCGATCCCTAACCTGAGGCGTTACTACCTGATGATGATCTCAATGAGTTCAAAACCTTTGATTTTACATTTTAGATTCTTGATGACGTTTGATTTAGAGAGATATGGTAATGTTCTGCAAGCAGCATATACCTACGTAACAGTATTAAAAAATTTGTTGAGTTGA

>OR55

ATGAGTCCTCCAAGCAGACATCTGTTCCTACTAATGACAGGAGGTGGCATGGGATACCATCCTCAATTTAAATTTAAGTGGCTTATTAAAGTATATGCAATCTATTTGTTCATTATGTTAATGATGAATTATTATACATTAACCATGGCCTGGCTAAGTGCTGATCATACTGATTTTGACGTAAAAGCCGAATTAATACAGTATTACCTAGAATGCTTTCACATTTTAGCTAAGTTTCTAAATTTTCTATTCAGACAGAAAGAAGTGAGAGAATTGTTAAGGACCGCCGATGAAATAAGGGAAGCGTGTAGATCTGATCCAGACAACCAATCAGTGATTGAACAGAATGAAAAAACTGAATCAACTTTGGCTAGATTTACAGTTATTGGAGCGGAAACCGTCATTGTCACATTCTTCGCTTGTACATGGATAAATTATGTGACAACTGGGAAAAAGAAATTACCTCTCTTATATTGGATCCCGTTTGATTCGCTAAAATACTTCTGGCAGGCTGTTATATTTCAATTGCTACTTTTCTTAACACCTGTCCAAATTTACTCCTGCTGCATGGGATTTAATGTTTTATTAGCTATGGTTGCCACATCGCAAATGAGGATTTTACAGCGACTTTTAACTACTAGAGATTCAAAAGCAGCAGCGAGAATTTCTACATACGAGACACATCAAGGCATTAATAAGTTGATATTACAAATGAATGAATTATTGTCTGGCCAGTTTCTATTCGAAATTTCTTTATCTGCACTTATACTTTCTATACGAATTTTCGCCTTCATCAAAGCAATTTTATCAGGAAAGGAGGGCCTAGTTTTTACTGCAGGAATATTACTTTTAGCAATGTCCTCGCCTTGTGTTATAAGCTGGGTAGGTGAACTGATTAAATCAGAGAGTGAGAAAATATTTGAAGCAGCCTACGACAATGTATGGTACGAGGAGGAAGTCAAAACTAAAAAAGAATTAATATTGGTACTAAGGGCTTCACAACGGCCACATACACTCCACTATCGTCATATCATCTCTTTTAATAGGCAACAATTTGCATCGATGATGCAAGCAACCTATTCATATCTTACTTTGCTCTTAAGATTTGGCGACTTGTAA

>OR56

ATGATTCCTCCAAGCACACGATTATATTTATTGATAGAAATGGCTGGTTTTGGTTACCGTCCTGAGCATAAATATCCCACCTGTATGAAATTATACATGATTTTTATGATGATTACATATGCAATAAACTGGTGTATAATTATTGTTGCGTTGTACTATGAAGAGGATAATTTCAATACAAAAGCTGAATTAATCCATTATCTTTTAGAAAGTGTGATTGCAACTGTAAAATATTTAAACTTTCTCCTCAGATTTAAAGAAGTGAGAGGATTAATGAAATTTGTAGATGAAGTAAGGGAATCATGTAGATCTGATCCAAATAATCAATCATTTATCGAGGAGAGTGAAAAATCTGAATCTAAAGCATTAAAAAATTGTTTCATTGTTGCAATCACTGTGCCCATAATTTTCTGTACTTCTTCTTCGTTGGTTTATATGATAAGTGGATTAAAGAAATTACCATTTTTATATTGGGTACCATTTGATTCGCAACGATATTTTTGGTTTGCTCTCGCTTTTCAACTTATAATCTATTCGACAGCATTCAAAGTTTATTTAGGAAGTTTTGGCTTCAACATTTTGTTAGTTATGGTTATCACATCATACATGAAAGTTTTACAGCGATTGTTGACTACAAAATATGGTGAAACTGCCAAGAGAAGGTCTGCTTACATTCTGCACCAAAATATTAACAGATTGGTGTTTCTAACTAATGAATTTTTATCTGGCCAATTTCTGTTCGAAATATCCTTTTCAGCAATTAAACTGTCGTTGAGAATATTTGCATTCATCAAGGTCTTTTCAACAAGAAAGCAAGATGTTTTTGTGTCTTTTGGAATATTAATCTTTTCAATGAGCTCTCCAGTTGCAATAAGCATTATTGGTGAAATGATCAAATCGGAGAGTGAGAAAATATTCGCAGCAGCCTACGACAACGTTTGGTATGAAGAGAAGGAAGAAAAAACCAAAAAAGAATTATTATTGGTGTTGAAAGCATCTCTACGTCCGCATAGTTTGCATTACCGCCGACTGACTTCTTTTAACATGGAGACGTTTGGAGAGTTACTTCAGGCTTCGTATTCATACCTCACAATGTTGTTACAGTTTAGCGAAATCAATTAA

>OR57

ATGGCAATACCCAAGGATGATGATGATGAAGAAACAGATGCCTTCATACCAATGGGGTTTATGCTAAAATGCATGGAAATTTCTGGCGTATTATTTACTAAAGAGAAAAGATCTACCAGAAGGGCTCGCTTTTTATCAGCCTATTATGCCATATCCGTAATAGTAGGCTTTATACCTCTAACTTTAAATCTTGTCTTCAACATAGATGACTTTTTCAATCAAGCAATGGAATCACTACATTTTATTCTCGCTGGAGCACACGCTACCTCAAAACATTTCAATATGATGTTTAAACAGGATGAATTCATGGAGCTATTTGATGAAATTAGAGCACTGTGGAAAGAAGCGGAAAGTAAACCATATCTTAGAAAATATGCACGCAAAAAGAGTAAAAGGAGTTCAAAAGTGGTTAAACTTTTATACAGCACCTTGCCATTAACATTCGTCGTATGTTCAGTAACAATGACAATCTTCAAAAATACATTATTAGGTAAAAAAGATTTACCAATACAAGTATGGATACCACTTGATTTGGACCGTCATGGAATGATTCTATGTTCAATACTTCAATATTATGTAATTACAACCGGCTTTATGGCATATCTAACGAATATATTCCTGATGCTAACTATATTTGATCATATCACTCTTATCATGAGACTATTCATCAAAGAATTAAGTATGAATAGAATCTGGACAAGACAAGAGAGAATCAATGTTTACATTACACATTCCAGAATAATAAAGGTTATAATTAAATTAAGAGACCTACTAGCAGCACAGTGGCTCCTGGAAACTCTGCTATCAGCTGCACAGATTACCTTAAGGGGGTACATGTGTTTAAAGACAGCAAAAGAGCATGACGTAATGTTTCTTTATTCGTTTATTGTTCTAATTGTAATAATTTGGGCCATTTCACTTACATATGTGGAAGGAAGTGAGATAACTATATTGGGAGAAAGTATTCATGATGCAGTTTACGGTTTACCCTGGTACAATGAAGACATCCAAGACAGAAAAGAATTAGTATTCATGTTGACAGCAACCAGCAAACCGTTGGATTTAAATTACAGATCATTTGTATTATTTGACAGAGCTCAGTTTCTGAATATTGTAAGGGCATCATTTTCATATTTGACTATGCTTCAAGTACTAGATTCTTCTTAA

>OR58

ATGGTGTATTCCTTTCTCAAACTGACCGACGGTATGGAGGAAGAAGGTCAGCTTAGGTTTTTATTAAACTTGGGAACTCAGCTCTTTATCTGTGCATACGTAACCATGGTTACAGGCATAGAATTGATTTGGGGGCATGGTAATTTGTATGAGATAGTCAACGCATTGTCAACATTCACAGTCGCTATCAATTACTTGACCAAAGTTGTCATTATACTCATGTATCGGAGGGAAATCCGTCAACTTTTTGCCAGATTGGAGCATCTTCATATGGAATTGCTTAAAGATGAAGATCAGCGCCATATTATTTTGGATATGGAAAGATTTTCTAAAATGTTTCTAAGGTTTTACAAAATATCATTAAATTTTTATCCAATCTTGTCACTAATAACGAATTTCATAAATGATTATAAGACCGATTTCAGAAAGATTTATTTATCGCTAAAGCTCTTGACGCCCTGGACTATTAGTGACATATGGTCATATTCTGCAGGCGCTATGTTAATTTTATGGATATCCGTTATGATGCTCTCCTCCTTTTTCTCATTCATAGCCATGGAATTAGCTTTCACATTTGAAATAACAGCCTTCTTAAAAGTTTTACAAGGCAGATTGAATAACATGAATGAGAAAGATGAGAACATCTATGGCCTGCATAGAGACATTATCAAATTAGTGACAGATTTTAACGCACTATTTTCCGGCCAAATGTACTGGGATATATTGGTTTCATCCATCCAGCCTTGTGGTTCTGGATTTATTTTAATTAAGGCGCTTAAAAGGAAAGATCCTGAAGCCACCGAACTTTTCTACAAAATGATTTTGGTAGTCATAGGCCCGTTTATTTTGTGCGCATGCGGACAACAGATAAGTACAGAGTCGGAAAAACTACATGAAGCATCTTACATGATTCCCTGGTATGAGCAAACTCCACGTAAGAGGAAAAATCTAATTCAGATGTTGACAGTAACTACAAAACCTAGCACTATTAACTTCAAAGGAATTATTGTCTTTAACTACAGCTGTTTTGCTGCGGTGGCCCAAGGGATATACTCCTATCTGATGATGGTCAATAAATTTGCAACTGATGACTGA

>OR59JC

ATGGTGTATTCCTTTCTGAAACTGACTGACGGTAAAGTCGAAGAAGGTCGGCTTTGGTTTTTTCTAGAACATGCAATTCGGTTTTTATTAACTGCATATCTAACTATCGTTTCAGGTAAAGAATTGATTTTGCGGCAAGGTGATTTGTATCAAATAGTCAATTCATTGTCAACATTCGCAATCTCTCTTAATATCTTCACCAAAGTTGTTATAACACTCTTTTATCTGAAAGAAATCCGCCAACTTTTTGACAGAGTGGAGCACCTTCATATGGATGAAGACCATCGCCATATTATTTTGGATATGGAAAGATTTTCTAGGATGTTTTTAGTGGGTTACAGAATGTCAATAATGATTTTCCCTTTTTACATGGTAATAACGAATTTCGTAATCGATTACAAAAGCGACTACAAACACATTTTTTTTACGCTAAAGCTCATAACGCCCTGGTGTATTACAAATCTATGGACATACACGCCAGGCACCCTGTTAGTGCTGTGGATATCCCTTGTGTCGATCTCATCCTACGTTTCATTCATCGCATTGCTGTTAGTTTTTACATTTGAAATTCAAGTGTTTTCAAAAGTTTTACAAAGCAGACTGAAAAATATGAATGAGAAAGATGAGAATATTTTCGGCCTGCATAGAGATATTATAAAATTAGTGGCAGATTTTAACGCTCTATTTTCCGGCCCAATGAATTTGGATATAGTGGTTTCATCCATTCAGCCTTGTGGTTATGGATTTTCTTTGATTAAGGCATTTAAAAGAAAAAATCCAGCAATCACTGAAATGTTCTACAAAATGATTTTGGTAGTCTCAGCTCCGTTCATTTTGTGCGCATGTGGACAACAGATAAGTACAGAGTTGGAAAAACTGCATGAAGCCTGCTACATGATTCCTTGGTATGTTCAAACTCCGCGAATGAGGAAAAATCTAATTCAGATGATGACAGTAACTACAAAACCTAGTACTATTGGTTACAAAGGAATTATTGTCTTTAACTACAGCTGTTTTGCTGCG

>OR60

ATGGTGTATTCCTTTCTGAATCTTACCAACGGTTTGAAGGAGGAAGATCGGCTTTGGTTTTTACTAAAATATTCAATTAGGTTCTTAGTCACTGCATATTTAGTCTTCAGTTCAGGTACAGAATTGTTTTGGGGGCATGGCGATATATATGTGATGGTCGATGCATTTTCATCATTCTTAATAACTTTTAATATCCTCATCAAAGTTATTACAGTACTGTTATATCGGAAGGAAATCCGCCAACTTTATGACAGAGTGGAGCATTTGCATATGGATTTGCGTAAAGATGAAGAGCATCACCTTGTTATTCTGGATATGGAAAGATTTACTAAAACTTCTTTATTGGCTTACAAAATAGTAATAATTATTTATCCTTTATACTCATTTATAACGAATTTCGTAATTGATTACAAGACCGACTTCAAAAATCTTTACTCATCGCTAAAGCTCTATACATACTGGCCTGTTACAAATTTATGGTCGTACACACCAAGCACTTTGTTAGTGTTGTTTATATCGTGTGTGACGATGTCTTTCTATGGTTCATTCATCGCATTGGAGTTAGTTTTTGCATTTGAAATAACAGCCTTCTTAAAAGTTTTACAAGGCAGATTGAATAACATGAATGAGAAAGATGAGAACATCTATGGCCTGCATAGAGACATTATCAAATTAGTGACAGATTATAACGCTCTGCTTTCCGGACAAATGTATTGGGAGATAGTGATATCATCCGTCGAACCCTGTGGATATGGATTCACTTTTATTAAGGCAATTAAAAGTAATGATCCTGCAGCCTCCGAATTGTTCTTCAAATTAATGCTGGTAGTGACATCTCCGTTCATTTTGTGTGCGTGCGGACAACTGATAAGGACAGAGTTTGAGAAATTGCATGAAGCCACTTACATGAGTCCTTGGTATGAACAAAATCCACCAGTGGGGAAAAATCTGGCACAGATGATGGTAGTAACAGCAAAACCTAGTACTATTGGTTACAAAGGAATTATTGTCTTTGACTACAGCTGTTTTTCTACGGTGGCTCAAGGGATATATACCTACATAATGATGATCTTTAATTTTGATACTGATGAGTGA

>OR61

ATGGTGTATTCCTTTCTCAAACTGACCGACGGTATGGAGGAAGAAGGTCAGCTTAGGTTTTTATTAAACTTAGGAGTTCAGCTTTTTATCTGTGCATACGTAACCATGGTTACAGGTATTGAATTGATTTGGGGGCATGGTAATTTGTATCAGATGGTCAATGCATTGTCAACATTCACAGTCGCACTCAATTTCTTGACCAAAGTTGTCAGTATACTCTTTTATCGGAGGGAAATCCGCCAACTTTTTGCCAGAGTGGAGCACCTTCATATGAAATTGCGTAAAGATGAAGACCATCACCGTATTATTTTGGATTTGGAAAGATTTTCTAAGATATGTCTTTGGTCTTACAAAATATCACTAATGATGTATCCATTCTACTCGTTTATAACGAATTTCATAATTGATTTCAAGAACGATTTCAGAAAGATTTTTTTCACAGTACAGCTCTTGACGCCCTGGAATATTAGTGACATATGGTCGTATACGACGGGCGCTTTGTTAGTTTTATGGATATCCCTTGTGATGATCTACTCCTTTTATTCGTTCATGGCCATGGAATTAGCTTTCACATTTGAAATAACAGCCTTCTTAAAAGTTTTACAAGGCAGATTGAAAAATATGAATGAGAAAGATGAGAACATTTATGGCCTGCATAGAGACATTATCAAATTAGTGACAGATTTTAACGCTCTATTTTCGGGCCAAATGTATTGGGATATATTGATTTCATCCGTCCAGCCTTGTGGTTTTGGATTTAGTTTAATTAAGGCACTTAAAAGGAGTGATCCTGGAGCCACTGAAATTTTCTACAAAATAATTTTGGTAATCATGGGCCCGTTTATTTTGTGCGCATGCGGACAACAAATAAGTACTGAGTCAGAAAAACTGCATGAGGCATCTTTTATGATTCCATGGTATGAACAAACTTTGCGAATGAAGAAAAATCTAATTCAGATGTTGACAGTAACTACAAAACCTAGTACTGTTAACTTTAGAGGAATTATCGTCTTTAACTACAGCTGTTTTGCTGCGGTGGCCCAAGGGATATACTCCTACCTGATGATGATCAATCAATTTGCAACTGATGAGTGA

>OR62

ATGCTGCCTTGGATACCGGCGGATATGTCACCGGCGAGAGTGGCCTACTTCAGTCTGAGATTGTGCGGCGTAATGAATCAACAAGGTAGACCATGGTCTTTGGTACATACAGTCTTTCAAATGACTTGCACCATTTTGATGATGATATTCTCTGTTCTGGATCTGTTCGTGGGCGACGAAGAACTGCCAGAATTGGTCATGGCGATGACTTCTACGATGATTTCGGTGCAGGTCAACTCTAAGGCCGTCAATATTCTTCTTCATCAAACAGAGCTACGGCAGATACTAGATAGAATAGACAAAATTCGCAACGATATTCTGCAAGATAAGGATAAACGTCACTACATACTAAACGGAGAGCAATCGGCCAAACAGTACTTAAGTTCCTATGGGATATTCATAGCGGTAGTGCTGACATTCTCAATGTTTTCCAACGCTGCCCTGGACTTCAGCACCAACATGGAAAAGCCACATTTATTGTTGCAGATTTGGATTCCGTGGAAAATGTCTACAGGATGGTTATACGCTGCCGGCCAGATTTTTAACTGCTTCTGCGTACTACCGACAGCGTTCCTTTATGTGGTTTATGTTGGATTAAATTTCATGTTCACATATGAAATGTCTGCATTTTTGAAAGTATTACAGGCTCGTATGAAAGAGATGAAACGATTAGATGATCCTGATATCTATCGCCAGCACAGAGAGATCTTAAGGTTATTGACCGACTATAACGATTTGTTTTCCGGACTAATGTATATAGAAGTATTGGTATCACCGTTGGAACCTTGTGGATTTGGTTTTGCATTGATGAAGACAATCCACACCAACTTGTTCGTATCTTTAGACTTATTTTGCAAATTTCTGGTAGCTGTGTTAGCATCATTCATACTGTGCGCTTGTGGACAGGAAATCAGTACGCAGGTGGATAACCTACATGAAAGTGCATATATGAGTAACTGGTACAATGAAGAGCCTGCAATGAGAAAGTATTTGTTGCAATTGATGACTATAACTGTACAACCCAATGGACTTTGCTACAGAAAGATGCTAATGTTTAATTTTGTCTGCTTCACTTCAATTGCGCAAGCTATATATTCTTATCTGTCAATGGTGAGCCAATTTGATCAAAATTAA

>OR63NTE

TTAAAAGAAGATAAGTCACCCTTTAAACTTGCCTACAAAAGCCTTAAATATGCTGGCATTATGAACCAAGAAGGTCGGCCGTGGTCGCTGGTGTACACTACGGTAATGGTCATAGGCAGTAGCTGTCTGACGGTCTTATCAATCGCGGATCTAATATGGGGTCAGGAAGAGTTACCAGAGTTCGTCTCAGCACTTTCCACCTTAACAATAACCATCCACGTCAATAGTAAAATGATCAATATACTTAGGAATCAAACGGAAATCCGCCAACTGATTAATAGATTTGAAAATGTTCGCCAAAAAATCTTACAAGATAATAGGAGGAAACATTTAATTTTATGGGCTGATCAATTTTCACTGAAAGTTGCCTTTTATTATGGAATGTTTTTTGCAAGTTGTCCGATTTTCTCAACGATCTCAAATATCATCATAGACGTGGTCACTGGGAGTGAAAAACCACATTTAGTTTTGCAGTTGTGGGTCCCATGGCGCATCAGGGACCTTGAGACCTACCTGGGAGCTACACTAGTTTCCATTGTATGTATATTCCCTACAGCCTCGATCTACATGGCTTTTACCGTTTTCAATTTTATATTCACCATAGAATTGTCCGCATTTCTACAATTCCTTCAGTCTAGATTTGAGACATTGAAGGTGGATAATAAGGAGGTTTATCGACAACATAAAGAAATTATTGAATTACTAAGAGATTACAATGAAATATTCTCCGGCCAGCTGTACGTAGAAATCATTATATCATCCTTACAGCCATGTGGATTTGGATACACGTTCATTAAGGCTGTAAAAAAACTCAATATACGGGCAATAGATTACATTTATAAATTTATGGTTTCTGCCGCATCACCCTTCATCTTGTGCGCTTGCGGACAGGAAATCAGTACTCAGTTAGAGAAATTACATACAAGTTGCTACATGTGTAATTGGTACGAGGAAAAGCCAGCGAGCCGAAGATATCTAGGGCAGGTGATGACAGTGACTTTGAAGCCCATTGCTCTCAGTTTTAGAAGAATGGTACCCTTCAACTATGTCTGCTTTGCTTCGGTAGCCCAAGGAATATACTCATACCTGATGATGGTCTATCAGTTTGACGAAGGCGATTGA

>OR64NTE

TTAAAAGAAGACAAGTCACCCTTTAAACTTGCCTACAAAAGCCTTAAATATGCTGGCATTATGAACCAAGAAGGTCGGCCGTGGTCGCTGGTGCACACTACGGTAATGGTCCTAGGCTGTAGCTGCCTGACGTTCTTATCAATCGCGGATCTAATATGGGGTCAGGAAGAGTTGCCAGAGTTCGTCTCAGCACTTTCCACCTTAACAATAACCGTCCAAGTCAATAGTAAAATGATCAATATACTCAGGAATCAAACGGAAATTCGCCAGCTGATTAATAGATTTGAAAATGTTCGCCAAAAAATCTTACAAGATAATAGGAGAAAACATTTAATTTTATGGGCTGATCAATTTTCACTGAAAGCTGCCTTTTATTATGGAATGTTTTTTGCAAGTTGTCCAATTTTCTCGACGATCTCAAATATCATCATAGACGTGGTCACTGGGAGTGAAAAAGCACATTTAGTTTTGCAGTTTTGGGTCCCTTGGCAGATCAGAGACGTTGAGACTTACCTGGCAGCCACACTACTTTCCATAGTATGTACAGTCCCATCAGCCTCCATCTACATGGCTTTTTCCGTTTTCAATTTTATATTCACCATAGAATTGTCCGCATTTCTTCAAGTCCTACAGTCTAGATTTGAGACACTTAAAATGGATGATAAGGAGGTTTATCACCAACATAATGAAATTATTGAATTACTAAGAGATTACAATGAAATATTCTCCGGCCAGCTGTACGTAGAAATCATTATATCATCCTTACAACCGTGTGGATTTGGATACACTTGCATTAAGGCTGTAAAAAAACTCAATATACAATCATTAGATTACATTTATAGATTTCTAGTTACCTCCGCATCACCCTTCGTCTTGTGCGCTTGCGGACAGGAAATCAGTACTCAGTTAGAGAAGTTACATACAAGTTGCTACATGTGTACTTGGTACGAGGAAAAGCCAGCGAGCCGAAGATATCTAGGGCAGGTGATGACAGTGACTTTGAAGCCCATTGCTCTCAGTTTTAGAAGAATGGTAGCCTTCAACTACGTCTGCTTTGCTTCGGTAGCCCAAGGAATATACTCATACCTGATGATGGTCCATCAGTTTGACGAAGGCGATTGA

>OR65NTE

TTAAAAGAAGACAAGTCACCCTTTAAACTTGCCTACAAAAGCCTTAAATATGCTGGCATTATGAACCAAGAAGGTAGGCCGTGGTCGATGGTGTACACTACGGTAATGGTCATAGGCAGTAGCTGTCTGACGGTCTTATCAATCGCGGATCTTTTATGGGGTCAGGAAGAGTTGCCAGAGTTTGTCTCAGCTCTTTCCACCATTACAATAACCATCCAAGCCAATAGTAAAATGATCAATATACTCAGGAATCAAAGGGAAATTCGCCAACTGATTAATAGATTTGAAAATGTTCGCCAAAAGTTCTTACAAGATAATAGGAGAAAACATTTAATTTTATGGGCTGATCAATTTTCACTGAAATTTGCCTTTTATTATGGAATATTTTTTGGGAGTTGTGTGCTTTTCTCGACGATCTCAAATATTATCATAGACGTGGTCACTGGGAGTGAAAAACCACATTTAGTTTTGCAGTTGTGGGTCCCTTGGCGTATCAGAGACGTTAAGACGTACTTAGGAGCCGCACTACTTTCCATATTATGTACAGTCCCATCAGCCTCCATCTACACGGCTTTTACCTTTTTCAATTTTATATTCACCATAGAATTGTCCGCATTTCTACAAGTCCTACAGTCTAGATTTGAGACACTGAAAATGGATGATAAGGAGGTTTATCAACAACATAAAGAAATTATTAAATTACTAAGAGATTACAATGAAATATTCTCCGGCCAGCTGTACGTAGAAATCATTATAGCATCCCTACAACCATGTGGATTTGGATACACGTTCATTAAGGCTGTAGAAAAACTCAATATACAAGCAATAGATTACATCTTTAAATTTATAGCTGCCGCCGCAGCACCCTTCATCTTGTGCGCATGCGGACAGGAAATCAGTACTCAGTTAGAGAAATTACATACAAGTTGCTACATGTGTAATTGGTACGAGGAAAAGCCAGCGAGCCGAAGATATCTAGGGCAGGTGATGACAGTGACTTTGGAGCCCATAGGACTCAGTTTTAGAAGAATGGTAGCCTTCAACTATGTTTGCTTTACTTCGGTAGCCCAAGGAATATACTCATACCTGATGATGGTCTATCAGTTTGACGAAGGCGATTGA

>OR66NTE

TTAAAAGAAGATAAATCACCCTTTAAACTTGCCTACAAAAGCCTTAAATATGCTGGCATTATGAACCAAGAAGGTAGGCCGTGGTCGCTGGTTTACACTACGGTAATGGTGTTAAGCAGTAGCTGTCTGACGGTCTTATCAACCGCGGATCTAATATGGGGTCAGGAAGAATTACCAGAGTTTGTATCAGCACTTTCCACTTTTACAATATCCATTCAGGTCAATAGTAAAATGATCAATATACTCAGGAATCAAACGGAAATTCGCCAACTGATTAATAGATTTGAAAATGTTCGCCAAAAATTCTTACAAGATAATAGGAGAAAACATTTGGTTTTGTGGGCAGATCAATTTTCACTGAAAGTTGCCTTTTATTATGGAATGTTTTTTGCAAGTTGTCCGATTTTCTCGACGATCTCAAATATTATAATAGACGCGGTCACTGGGAGTGAAAAACCACATTTAGTTTTGCAGTTGTGGGTCCCTTGGCAGATCAGAGACGTTGAGACCTACCTGGGAGCCACACTAGTGGCCATAGTGAGTACATTCCCAGCAGCCTCCATCTACATGGCGTTTACCGTTTTCAATTTTATATTCACCGTTGAATTGTCCGCATTTCTACAAGTTCTTCAGTCTAGATTTGAGACGCTGAAGGTAGATAATAAGGAGGTTTATCGACAACATAAAGAAATTATTGAATTACTAAGAGATTACAATGAAATATTCTCCGGCCAGCTGTACGTAGAAATCATTATATCATCCTTACAACCATGTGGATTTGGATACACTTTCATTAAGGCTGTAAAAAAACTCAATATACGAGCAATAGATTACATCTATAAATTTATAGTTACCGCCGCAGCACCCTTCGTCTTGTGCGCTTGCGGACAGGAAATCAGTACTCAGTTAGAGAAATTACATACAAGTTGCTACATGTGTGATTGGTACGAGGAAAAGCCAGCGAGCCGAAGATATCTAGGGCAGGTGATGACAGTGACTTTGAAGCCCATTGCTCTAAGTTTTAGAAGAATGGTAGCCTTCAACTATGTCTGCTTTGCTTCGGTAGGCCAAGGAATTTACTCATACCTGATGATGGTCTATCAGTTTGACAAAGGCGATTGA

>OR67NTE

TTAAAAGAAGATAAGTCACCCTTTAAACTTGCCTACAAAAGCCTTAAATATGCTGGCATTATGAACCAAGAAGGTCGGCCGTGGTCGTTGGTGTACACTACGGTAATGGTCTTAGGCATTAGCTGTCTGACGGTCTTATCAATCGCGGATCTAATATGGGGTCAAGAAGAATTACCAGAGTTCGTCTCAGCACTTTCCACCTTAACAATAACCATCCACGGCAGTAGCAAAATGATCAATATACTTAGTAATCAAACGGAAATTCGCCAACTGATTAATAGATTTGAAAATGTTCGCCAAAAGTTCTTACAAGATAATAGGAGGAAACATTTGGTTTTGTGGGCTGATCAATTTTCACTGAAAGTTGCCTTTTATTATGGAATGTTTTTTGGAAGTTGTGTGCTTTTCTCGACGATCTTAAATATGATCATAGGCGTGTTCACTGGGAGTGAAAAACCACATTTAGTTTTGCAGTTGTGGGTCCCTTGGCGGATCAGAGACGTTGAGACCCACCTGGGAGCTACACTACTTTCCATAGTATGTACAGTCCCATCAGCCTCCGTCTACACGGCATTTTCCGTTTTCAATTTTATATTCACCATAGAATTGTCCGCATTTCTACAAGTTCTTCAGTCTAGATTTGAGACGCTGAAAATGGATGATAAGGAGGTTTATCAACAACATAAAGAAATTATTAAATTACTAAGAGATTACAATGAAATATTCTCCGGCCAGCTGTACGTAGAAATCATTATATCAACCTTACAACCATGTGGATTTGGATACACGTTCATTAAGGCTCTACAAAAACTCAGTATACAAGCAATTGATTACATCTTTAAATTTGTAGTTACCGCCGTATCCCCCTTCATGTTGTGCGCTTGTGGACAGGAAATCAGTACTCAGTTAGAGAAATTACATACAAGTTGCTACATGTGTAATTGGTACGAGGAAAAGCCAGCGAGCCGAAGATATCTTGGGCAGTTGATGACAGTAACTTTGAAGCCCATTGCTTTCAATTTTAGAAGAATGGTAGCCTTCAACTATGTTTGCTTTACTTCGGTAGCCCAAGGAATATACTCATACCTGATGATGGTCTATCAGTTTGAAGAAGGCGATTGA

>OR68NTE

TTAAAAGAAGATAAATCACCCTTTAAACTTGCCTACAAAAGCCTTAAATATGCTGGCATTATGAACCAAGAAGGTAGGCCCTGGTCGCTGGTTTATACTACGGTAATGGTTTTAAGCAGTAGCTGTCTGACGGTCTTATCAACCGCGGATCTAATATGGGGTCAGGAAGAGTTACCAGAGTTCGTCTCAGCACTTTCCACCTTAACAATAACCATCCACGTCAATAGTAAAATGATCAATATACTTAGGAATCAAACGGAAATCCGCCAACTGATTAATAGATTTGAAAATGTTCGCCAAAAAATCTTACAAGATAATAGGAGAAAACATTTAATTTTATGGGCTGATCAATTTTCACTGAAAGTTGCCTTTTATTATGGAATGTTTTTTGCAAGTTGTCCGATTTTCTCAACGATCTCAAATATCATCATAGACGTGGTCACTGGGAGTGAAAAACCACATTTAGTTTTGCAGTTGTGGGTCCCATGGCGCATCAGAGACGTTGAGACCTACCTGGGAGCCACACTAGTGGCCATAGTGAGTACATTCCCAGCAGCCTCCATCTACATGGCGTTTACCGTTTTCAATTTTATATTCACCATAGAATTGTCCGCATTTCTACAATTCCTTCAGTCTAGATTTGAGACACTGAAAGTGGATGATAAAGAGGTTTATCTACAACATAAAGAAATTATTGAATTACTAAGAGATTACAATGAAATATTCTCCGGCCAGCTGTACGTAGAAATCATTATATCATCCATACAACCATGTGGATTTGGATACACGTTCATTAAGGCTTTAAAAAAACTCAATATACAAGCAATAGATTACATTTATAAATTTATAGTTGCCGCCGCAGCACCCTTCGTCTTGTGCGCTTGCGGACAGGAAATCAGTACTCAGTTTGAGAAATTACATACAAGTTGCTACATGTGTAATTGGTACGAGGAAAAGCCAGCGAGCAGAAGATATCTAGGGCAGGTGATGACAGTGACTTTGAAGCCCATTGCTCTCAGTTTTAAAAGAATGGTAGCCTTCAACTATGTCTGCTTTGCTTCGGTAGCCCAAGGAATATACTCATACCTGATGATGGTCTATCAGTTTGAAGAAGGCGATTGA

>OR69NTE

ATAGAAGATAAGTCGTCTCCATTTAAAGTGGCCTTATTGGTTTTCAAGTACGCCGGCATTTTCAACAAAGAGAACAGACCATGGTCGCTGATATTTACGAAGGCTAACACTCTCGTTACTATCTGTGTAACGATCTTATCTTGTGTCGATATGATATGGGGCAATGAAGAAATGCCAGAATTTGTTTCAGCACTCTCAGCTTTCTTAATATCGATCCATATAAATGGAAAATTATTTAACTTTATTTATCGTGAAACTGAAATTGGCCATTTCATTGAAAGAATGGAAAAACTGCGCCAAAAAATTCTACAAGAAAATTGTGGAAAACAGATTGTTTTACGTGCCGACAGAGTTTCAATTAAAATGGCCAAGTATTATGCACTATTTGTCCTTTGTTGTCCAACTTTGTCTGTTGCTTCAAGTATTATTATAAATAATGTAATCGGCAATCGAAAAGCACAGCCCTTTTTTCAGATATGGTTACCTTGGGACATAACAGACATCAGAATAAATATTGCAACAAATATATTAGTGATTTTTTGCGTAACCCCATCAGTTTTGGTCCACTTGACATTTGCTACGTTCAGTTTCATTTTCACTATCGAAATGTCTTCATTTCTACAAGTTTTACAATCAAAATTGGAAACATTAAGACTAAACGATAAGATGGTTTATCGACTTCATGCAGAGATTATCCAATTGGTAGAAGATTATAATAACTTATTTTCTTTGCAAATTTATATGGAAATTTGCCTATCTAGTGTCCAACCTTGTGGATTTGGATATACATTGATCAAGGCTTTTAAAACTCACGATATACGAATGGTGGACTTTATTTATAAATTTTTACTCACCATTGTCGTTCCATTCATTATTTGCACCTGCGGACAGGAAGTTAATACTCAGTTAGAGAAATTACACTCAAGTTGCTACATGTGTGATTGGTTTGAGGAAAAGCCAGCGAGCCGAAGGTACTTGTTGCAGTTGATGATGTCAACCGTTAGGCCGCATGCTATTGGTTTTAGAAGAATGATAACGTTCGACTATGTCTGCTTTACTTCGGTACTGCAAGGAATATATTCTTTTTTGACGTTGGTCAATCAATTTGGAGAATAA

>OR70-FX

ATGGTCAATTTGGTACCGAAGAATATTTCACCAACAACTCTGTCATATAAGTCTTTAAGATTTGTTGGCCTACTGAACCAAGAAGGAAGACCATGGTCTTATCTGATCACTGCGTATCACTTTATTATCATGAAACTTCTAGTCGTCACTATCGTTTTACAACTATTTTGGACCACTCCTGATTTACCAGACTTTATCCAAGCTTTGTCATGGATAACTATATGCAGCCATGTTTATTCCAAATTAGTGAACATGTTTTGCCATCAGACGGAAGTCCGACAAATGTTAGACAGAATTGAAGAAATTCGCAACGAGATCTATCAAGACTATAAATATCAGCACATTCTCATAGATTCGGACGGTTTACTGATTAAATTAATTGAATATTATTGTACATTTTTTGTAAGCTATCCGGTCATTTCATTGGCTATGAATTTAGTTGTGGATTATGTTACTGGTTCGGAGGAACCACATTTAATACTTCCAGTATGGATGCCGTGGAATATGAAAGAATTTTGGCCATATCTAGGAGGGATGTTGTACGTAACAATCTTTCCAATCACAACTACTTTCATTTACGCAGCGATCTGCATTTTCCAGTTTACTTTCACATTTCAAATATCTGCTTTTGTGAAAGTTCTGCAAGCCAAACTGGAAGGCGATAGACCAAATAATCAAGTGTACAGACAGCACGCGGAAATAATACGGTTACTTCAACAATACAATGACCTATTTTCTGGACAGCTGTATTTTGAAATATTAGCATCATCCGTACAACCTTGCGGTTTTGGCTATACTTTGATTAAGATAATAAAAAGAAATGATCCAGGTGCGTTTGATTTGATTTACAAGATTCTTCTGGCACTTTCAGCACCGCTAACGGTCTGCGCCTGTGGACAAGTTATTCGGACTCAGTTTGAAAAACTACATACCAGCACTTACATGGGAAAATGGTACGAAGAAAAACCATCCATTAGGATGGATCTAGTGATGATGATGAAAATCACTGCAATACCAAAATCATTATCGTTTAGAAGATATGTGATATTTGATTTCGTCTGTTATGCTTCGGTTTTACAAGGAGTTTATTCATATTTAATGATGATCAGTCAATTTGATTTATAA

>OR71NTE

ATCTCTAAGGATACTACTCCGGTAAAATTCTGCTACAACTACCTTAAATACGCTGGCTTACTCAACAAAGAAGGAAGACCCTGGTCCATTCTACTCACAGCGTACAATCTGATCATGTTGAACGCATTAGGACTGGGAGCAATTAAGATAATTCTCTGGGGGGACGGCAATCTGCAAGAACTAATCCAGGCCATGTCAGCTACCATCATTTACGTACATATAAATGCAAAAGCTATAAAAATACTGTACTGTGAAACAGAGATTCGCAAGCTTTTTGAAAGTTTAGAACAATTACGCCAAGAACTGCTGGAAGATATTGATAATCAACATCATATAATTGACGCAGAATCATTTCTATTCAAGTTGAACACCGGATACGTCCTGTTGATGATGAGTTTCCCAGCTGTGTCCTTCGGCATCAACACATTCACTGATTATATCACACAGTCTGAACGACCGCATTTAATCTTCCAAGTGTGGATACCTTGGAGTACGAAAGAATTCTGGCCATATCTAGGAGCCATGTTCTACACTACTGTGCTGTCTATTTCAAGTGGCATCTTATACACTTCCTATACGATATTTCTATTCACTATAACCTTTGAATTGTCCGGATTTATCAAAGTTCTACAGGTGAAATTGGAAACCAATGGAATCATCGATAGAAACACATATCGACATCACCTTGCAATAATGCAATTGATACAAACCTGTAATGAAATCTATTCTGGACAATTGTATTTTGAGACTTTAGTATCATCTTTACAGCCGTGCGGATTTGGGTATACATTAATTAAGGCGGCTCGACGACAAGACCCAGGGGTATTTGATTTACTTTACAAATTCTTTCTGGCAGTCTCAGCTCCGTTCATAATTTGCTCTTGTGGACAGGAAATTAGTAACCAGATGGAAATGCTACACAATAGTTCATACATGGGACCATGGTATCAGATATCGCCGAAGTTAAGAAAGGAACTACTAATCATGAAGATATTGAACACTAAACCAATCACTTTAAGTTATAGGCGATTTGTTACATTCAATAATGCGTGTTTTGCAACGGTAGCTCAAGGAATATACAGCTATTTGGCCATGATCAATAATTTGGAAGATTCAGAATAA

>OR72NTE

ATCTCTAAGGATACTACTCCGGTAAAATTCTGCTACAACTGCCTTACATATGCTGGCTTACTCAACCAAGAAGGAAGACCCTGGTCCATTCTACTCACAGCGTACAATCTGATCATGTTGAACGCAATAGGACTGGGAGCAATTAAGATAATTCTCTGGGGGGACGGCAATCTGCAAGAACTAATCCAGGCCATGTCAGCTACCATCATTTACGTACATATAAACGCAAAAGCTATAAAAATACTGTACTGTGAAACAGAGATTCGCAAGCTTTTTGAAAGTTTAGAACAATTACGTACAGAACTGCTGGAAGATATTGACAATCAACATCATATAATTGACGCAGAATCATTTCTATTTAAGTTGAACACCGGATACGCCCTATTGATGATGAGTTTCCCAGCTGTGTCACTCGGCATCAACATATTCACTGATTATATCACACAGTCTGAACGACCGCATTTAATCTTCCAAGTGTGGATACCTTGGAGTATGAAAGAATTCTGGCCATATCTTGGAGGCATGCTCTTTACTACTGTTCTTTCTATTTCAAGTTGCATCTTCTACACTTCCTATATCATATTTTTATTTACTATAACCTTTGAATTGTCCGGATTTATCAAAGTTCTTCAGGCGAAATTGGAAAACAATGGAATCATCGATAGTAACACATATCGACATCACCTTGCAGTAATGCAATTGATACAAACCTGTAATGATATCTATTCTGGACAATTGTATTTTGAGACTTTAGTATCATCTTTACAGCCGTGCGGATTTGGGTATACATTAATTAAGGCGGCTCGACGACAAGATCCAGTGGTATTTGATTTACTTTACAAATCCTTTTTGGCAGTCTCAGCGCCGTTCATAATTTGCTCTTGTGGACAGGAAATTAGTAACCAGATGGAAATGCTACACAATAGTTCTTACATGGGACCATGGTATCAGATATCGCCAAAATTTAGAAAGGATCTACTAATCATGAAGATATTGAACACTAAACCTATAACTTTAAGTTACAGGCGATTGGTTACATTCAATAATGCTTGTTTTGCTACGGTAGCTCAAGGAATATACAGCTATTTGGCTATGATCAATAATTTGGAAGATTCAGAATAA

>OR73NTE

ATCTCTAAGGATATTACTCCAGTAAGGATCTGCTGCAACTGTCTTAAATATGCTGGCCTATTCAGCCAAGAAGGAAGACCATGGTCCATTCTACTTTCGGCTTATCATCTGATCATGTTGAACGCAGTAGGACTAGGAGCAATTAAGGTAATCCTCTGGGGGGACGGCAATCTGCAAGAACTTATCCAGGCCATGTCAGCTACCGTCATTTATGTGCATACCAACGCAAAAGCTATAAACTTCCTTTCCTATGAAGCAGAGATTCGCAAACTGTTTGAAAGTTTAGAACAATTACGCACAGAACTGCTGGAAGATATTGACAATCAACATCATATAATTGACGCAGAATCATTTCTATTCAAGTTGAACACCGGATACGCCCTGTTGATGATGAGTTTCCCAGCTGTGTCACTCGGCATCAACATATTCACTGATTATATCACACAGTCTGAACGACCGCATTTAATCTTCCAAGTGTGGATACCTTGGAATATGAAAGAATTCTGGCCATATCTTGGAGGCATGCTCTTCACTACAGTTCTATCAATTACTATTTGCGTATTCTACACTTCCTACATCATATTTCTATTCACTATAACCTTTGAATTGTCAGGATTTATCAAAGTACTACAGGCGAAATTGGAAACCAATGGAATCATCGATAGTAACACATATCGACATCACCTTGCAATAATGCAATTGTTACAGACTTGTAATGAAATCCATTCTGGACAATTGTATTTTGAGATTTTAGTGTCATCTTTACAGCCGTGCGGATTCGGGTATACATTAATTAAGGCGGCTCGACGACAAGATCCAGTGGTGTTTGATTTACTTTACAAATCCTTTCTGGCAGTCTCAGCTCCGTTCATAATTTGCTCTTGTGGACAGGAAATTAATAACCAGATGGAAATGCTACACAATAGTTCATACAGGGGACCATGGTATGAGAAATCGCCGAAATTGAGAAAAGATCTATTAACTGTGAAGATATTGAACATTAAACCAATAACTTTAAGTTATAGGCGATTGGTTACATTCAATAATGCTTGTTTTGCTACGGTAGCTCAAGGAATATACAGCTATTTGGCCATGATCAACAATTTGGAAAATTCAGAATAA

>OR74NTE

AATTTTAAAAGGCTTTCACCAATACAGTTAATCTACTACTTACTGACAAGATGTGGACTATTGAATGAAGATGGCCGCCCATGGACTGTGATCATCGCCACTTACCATTCCATCATGCTGATATTGGTGGGTCTTCTTACAGTCGCAAAAGTTTATAAGAAATTGGATGATATAAACGAGTTTATACTACCACTCATGCTGTGGGCAATGATCATCCATATATCTGGAAAATTCTTTTATTTACTAAAAAATCAGAGGAAATTACGTAGCTTACTAGATCGATTGGAACAACTACGCCAGGAAGAGCTCGGTGATTCTGTCTACGCTGAACATTTCATCAAGGCCGACAAGATTAATAAAACTATACTCTACTATCTTAATCTGAACATTCACTTGGTTCCGGTAATTTCTACCGGGTTCAACGTTTTGCACGATTGTCTGTCTACATCTGAAACTCCATCTTTAATGATGCCGATATGGATTCCTTGGGAGCACCAGAAATCTTGGCCTTACGCTGCAGCGGTTATCGCTACCACAATAATGGCATTTTCTGGCGTAATTGTGTTTGCTACGTTATATTCCCTGCTAGTAACAATCACCTTGCAACTGTCAGCGTTGGTACAAGTGTTGCAAGCTAAAATGGAGAAGAAACAAGCAAACGATAAGAGCATTTTCCGCCTGCACATTAATGTTTTACAAGTACTGCAACAGCTTAACGACCTATTATCTGGCCAATATTGTCTAGAAATATTGCTATCATCATTACAGCCCTGTGGATTCTGCTACATGCTGATAAAGTATTTGAAAAGTGGAGATTCAAGATGGGTGGACTGTTTGTATAAAGTTTTCGTCTCACTGTTCGCAACATCATTCCTGTGCGCCTGCGGTGAGGAGATTAATTCTCAGGTGGAGTACTTACATCAAAGTGCGTACAGAGGTTCCTGGTATGAAGAACAGCCTGCTGCAAGGAAAGACTTGATAATTTTAATAACTATAACATCTAGACCACTTCAATTCCAGTACAAAGGATTGGTTACGTTCAACTTACGACGTTTGGCTACGGTAATTCAAGGATTCTACTCGTACACAACAATGTTGAGCAACTTGGAAACAGCTGATTAG

>OR75NTE

AATTTTAAAAGGCTTTCACCAATACAGTTAACCAACTATTTACTGACAATATGTGGAATATTGAATCAAGATGGCCGCCCGTGGACTGTGATCATCGCCACTTGCCATGCCATCATGCTGATATTGGTGGGCATTCTTACTGCCGCAAACGTTTATAAGAAATTGGATGATATAAACGAGTTTATACTACCTCTGATGATGTGGGCAATGATCACCCATACATCTGGAAAATTCTTTTATTTACTACTAAATCAGAGGAAATTGCGTAACTTACTAGATCGATTGGAACAACTACGCCAGGAAGAGCTCGGTGATTCTGTCTACGCTGAACATTTCACCAAGGCCGACAAGATGAATAAAACTATACTCTACTATCTGAATCTGAACATTCACTTGGTTCCGGTTATTTCTACCGCGATCAACTTTTTTCACGATATTCTGTCTGCATCTGAAACTCCATCTTTAATTTTGCCGATATGGATTCCTTGGGAGCACCAGAAATCTTGGCCTTACGTTGCAGCATTTGTCACTTCCGTAGTAATTGGATTTTCTGGCGCAACTGTGTTTGCTACGTTATATTCCCTGCTAGTAACAATCACGCTGCAAATATCAGCGTTGCTACAAGTGTTGCAAGCTAAAATGGTGGAGAAAAAAGCAAACGATAAGAGCATTTTCCGCCAACACATCAATGTTTTACAAGTACTACAGCAGTTTAACGACCTATTGTCTGGCCAATATTGTCTAGAAATATTGCTATCATCATTACAGCCCTGTGGAAACTGCTACATAATGATAAAGTATTTGAAAAATGGAGATTCAAGATGGATGGACTGTTTGCATAAAGTTTTGCTCGCACTGAGTGCAACATCATTTCTGTGCGCTTGCGGTGAGGAGATTAACTCACAGGTGGAGAACTTACATGGAAGTGCGTACAGAGGTTCCTGGTATGAAGAACAGCCTGCAGCAAGGAAGGATTTGATAATTTTAATGACTATAACATCTAGACCACTTCCATTCCAGTATAAAAGATTGGTTACGTTCAACTTACGATGTTTCGCTGCGGTAATTCAAGGATTCTACTCGTACACAACAATGTTGAGCAACTTGGAAACAGCTGATTAG

>OR76NTE

AATTTTAAAAGGCTTTCACCAATACGGTTAACCAACAACATACTGACAAGATGTGGACTGTTGAGTCAAGAAGGCCGCCCTTGGACTGTGATCATCGCCACTTACCATGCCATCATGATGATATTGGTGGGCTTTCTTACTGTCGCAAACGTTTATAAGAAATTGGATGATTTCAACGAATTTATACTACCTCTCTTGCTGTTGCCAATCATCACCCATGTATCTGGAAAATTCTTTCATTTACTACTAAATCAGAGGAAATTACATAAATTACTAGATGGATTGGATCAACTACGCCAGGAAGTGCTCGGTGATTCTGTCTACGCTGAACATTTCAGCAAGGCCGACAAGGTGAATAAATCTATACTCTACTATCTCAATCTGAATCTTCACTTGGTTCCGGTTATTTCTACCGTGATCAACGTTTTGCACGATTATCTGTCTGCATCTGAAACTCCATCTTTAACGATGCCGATATGGATTCCTTGGGAGCACCAGAAATCTTGGCCTTACGTTGCAGCGGTTATCGCTTCTACGGTAATTACATTTTCTTGCGCAACTACGTTTGCTACGGTATATTCCCTGCTAGTAACAATCGCCTTGCAAATGTCAGCGTTGCTACAAGTGCTGCAAGCTAAAATGGAGGAGGAAAAAGCAAACGATAAGAGCATTTTCCGCCAACACATTAATATTTTACAAGTAATACAGCAACTTAACGACCTATTATCTGGCCAGTATTGTCTGGAAATATTGTTATCAACATTACAGCCCTGTGGATACTGCTACATGATGATGAAGTATTTGAAAAGTGGAGATTCAAGATGGATGGACTGTTTGTATAAAGTATTGATCGCACTGTGTGCATCAACAATTCTGTGCACTTGCGGAGAGGAGATTAACTCTCAGGTGGAGAACTTACATCAAAGTGCGTACAGAAGTTCCTGGTATGAAGAACAGCCGGCAGCAAGGAAGGATTTGATAATTTTAATGACCGTTACAGCTAGACCTTTTCAATTCCAGTATAAAGGATTGGTTACGTTCAACTTTCACCGATTCGCTATGGTAATTCAAGGATTCTACTCGTACACAACAATGTTGAGCAACTTGGAAACAGCTGAGTAG

>OR77NTE

AATTTTAAAAGGCTTTCATCAATACAGTTAATCTTCGACTTACTGACAAGATGTGGACTATTGAATCAAGAAGGCCGCCCTTGGACTGTAATCATCGCCACTCACCATTCCATCATGCTGATATTGATGGGTCTTCTTACTGTCGCAAAAGTTTATGAGAAATTGGATGATTTCAACGAGTTTATACTACCTCTGATGCTGTGGGCAATGATCATCCATATATCTGGAAAATTCTTTTATTTACTACTACATCAGAGGAAATTACATAAATTACTAGATCGATTGGAACAACTACGGCAGGAAGTGCTCGGTGACTCTGTCAACGCTGAACATTTCACCAAGGCCGACAAGATGAATAAAACTATCCTCTACTATCTGAATCTGAATATGCACTTGGCTCCGGTTATTTCTACTATGCTCAACGTTTTGCACGATTATCTGTCTGCATCTGAAACCGCATCTTTAACGTTACCGATATGGATTCCTTGGGAGCACCAGAAATCTTGGCCTTACGTTGCAGCGGTTATCGCTTGTATGGTAATAGCATTTACTGGCGCAATTGTGTTTTCTGCGTTATCTTCCCTGCTAGTAACAATCACTCTGCAAATATCATCGTTACTAAAAGTGTTGCGAGCTAAAATGGAGGAGAAAAGAGCAAACTATAAGAGCATTATCCGCCAACACATTAATATTTTACAAGTACTACAGCAATTTAACGACCTATTGTCTGGCCAATATTGTCTGGAAATATTGCTATCAGCATTACAGCCCTGTGGATTCTCCTACATGCTGATAAAGTATTTGAAAAGTGGAGATTCAAGATGGGTGGACTGTTTGTATAAAGCTTTCGTCGCACTGTTTGCAACATCATTTCTGTGCGCTTGCGGTGAGGAAATTAATTCTCAGGTGGAGAACTTACATCAAAGTGCGTACAGAAGTTCCTGGTATGAAGAACAGCCTGCAGCAAAGAAGGATTTGATAATTTTAATGACCGTTACAGCTAGACCACTTCAATTCCAGTATAAAGGATTGGTTACGTTCAACTTACGACGTTTGGCTATGGTAATTCAAGGATTCTACTCGTACACAACAATGTTGAGCAACTTGGAAACAGCTGAGTAG

>OR78NTE

AATTTTAAAAGGCTTTCACCAATACAGTTAACCAACTACATACTGACAAGATGTGGACTGTTGAATCAAGAAGGCCGCCCATGGACTGTGATCATCGCCACTTACCATGCCATCATGCTGATATTGGTGGGCTTTCTTACTGTCGCAAACGTTTATAAGAAATTGGATGATTTCAACGAGTTTATACTACCTCTCTTGCTGTTGCCAATCATCACCCATGTATCTGGAAAATTCTTTCATTTACTACTAAATCAGAGGAAATTACGTAAATTACTAGATGGATTGGAACATCTGCGCCAAGATGAGCTCGGTGATTCTGTCTACGCTGAACATTTCATCAAGGCCGACAAGATGAATAAATCTATAGTCTACTATCTGGATCTGAATATGCACTTGGCTCCGGTTATTTCTACCGGGCTCAACGTTTTGCACGATTATCTGTCTGCATCTGAAACTCCAACTTTAATGATTCCGATATGGATTCCTTGGGAGCACCAAAAATCTTGGCCTTACGTTGCAGCGGTTATCGCTTCTACGGTAATTTCATTTACTTTCGCAACTGTGTATGCTTCATTATATTCCCTGCTAGTAACCATCGCCTTGCAAATGTCAGCGTTGCTACAGGTGCTGCAAGCTAAAATGGAGGAGGAAAAAGCAAACGATAAGAGTATTTTCCGCCAACACTTTAATATTTTACAAGTAATACAGCAACTTAACAACCTATTATCTGGCCAATATTGTCTGGAAATATTGGTATCAACATTACAGCCCTGTGGAGCCTGCTACATGCTGATAAAGTATTTGAAAAGTGGAGATTCAGGATGGTTAGACTGTTTGTATAAAGTTATGGTCGCACTGGGTGCATCAACAATTCTGTGCGCTTGCGGAGAGGAGATTAACTCACAGGTGGAGAACTTACATCAAAGTGCGTACAGAAGTTCCTGGTATGAAGAACAGCCTGCAGCCAGGAAGGATTTGTTAATTTTAATGACGGTTACAGCTAGACCACTTCAATTCCAGTATAAAGGATTGGTTACGTTCAACTTTCACCGATTCGCTATGGTAATTCAAGGAGTCTACTCGTACACAACAATGTTGAGCAACTTGGAAACAGCTGAGTAG

>OR79-FX

ATGAACAAACAACCTGAGACACCAGAAGTGATGACAACTATCCAACGGGCCTATCACCAAATTAGATTTAGTGGGCTAGTGTATGACAAAAACAATCGAAGATCTGTATTTCTGGCAATATACCATACGATTATGGTGAACTATGTTGGTATGGCTTCAGCTGCAGCTTTGATAATAGGAGAACAAAGTTTAGTAGATTTTGTTCAAGTATTGGGAGTAACAATGATATACATAAACAATGTAGTTAAAGCTATTAATATATTGATTTATCAGAAGAAATTTAAGGAACTATTTGCCAGATTGGAAAAGTTCAGTATGGAAGTTACGCACGATGAAGCGGAAGAACATGTAGAACTACGTCCGAAAAATTTACATTCTAAATTTATAACCATTTTCTCAAAAGTATTAATATACGCACCACCAATTTCAGTGTCCACAAATATACTTAGTGATTATCTGAAGGACTTTGTGAAACCTCATCTACCTTTTCAGTTATGGATACCATGGAAACTGACCCCGTACTGGCCTTACTTCGCGTTTGACGCTATGCTTATGGTCACCATGAGTCAGTACTACATGTCATTCACGACCTTACTCTTTGCATTCACTACCGAACTGAACGCCTGTGTCCGGGTACTTCGGCATCGACTGGAAACTAATGGACCAGCTGACAAGAATGTCTACAGATATCACCATACAATTCTTGAACTGTTGAAAGATTATAACAAACTGTTTTCTGGTCCTGTATATTGGGAAATACTAGTATCCGCTTTACAACCTTGTGGCTTTATTTACGCTCTTATTAAGCTCTTGAAACGGAAGGATCCAGCTAGTACGGAATTAGTCATGAACGCACTACTGGCTTTAGGGGCACCCTTTGTTCCTTGCTCTTGTGGACAGGCCATCAGTACCCAGATGGAACTACTACACGACAGCGCTTACATGGGTAAGTGGTATGAAGAGAAACCAAAAGTGAGGAGGGACTTGCTTACTATGCAACAGGCTGATCTGAATTACAGGAAATTTATCTCCTACAACTTCGTCTGTTTTGCCACGGTTATCCAAGGAATTTACTCATATCTGATGATGATCATCCAATTTGAAGATGATTAG

>OR80-FX

ATGTCTCAGGTAACGGAAAATTTATCTCCAGTAAAGCAATGTTACCGCGCAATCAGATTCAGTGGTCTACTAGCTGATACTAATAATGCATGGTATATGATATTCTCAGTCTATCATATGGTTATGATAAATTACATCGGCGTGGCTGCAGTCTTAGAGATTTTACTGAAGGAAAGAAATTTACTCGATTTTGTGCAAGCCCTTTCAGTAATAGTGATCTACGTGCATGACATTGTTAAAGCTGTGAACATCTTCACTCACCAGAAGGGCGTCAAACAATTACTTAACAGGCTGGAAGAATTCATTCAGGAAACTGCAAAAAATGAACCGAATCAATCACGCAAACGATCTTTTCGCGAGACATTCATCTCAATTTACATAAAAATATTGATTTGGTTGCCATCTTTATCCCTTGTTTGTGGAGTGTTTGGCGATTATATGACTGGCTTTGTCAAACCGCATTTACCTTACCAAATCTGGATTCCATGGAGTTTAAAAGAATTCTGGCCTTACATGGCCGGAATGGTATTCGTGACAATGCTGGAGTTCACCACCTGCGTATACTACATGTCCTTCACAGTAATCAATTTTACTTTCTCAAATGAGTTATCCTCAAGTTTGAGAAGGCTACAAGAACGCCTGGAGACCAAGGGACCGGCCGATAAAACTGTCTATGAACATCATAATGCAATTATACAGTTATTACTGGATTACAATCAGTTATTTTCTGGACCTCTGTACGTTGAAACTTTAATGTCTTCTTTAATGCCTTGTGGATTTTTCTACCTGTTTATTAAGATTGTTAAGAGTTTCGATCCACTCGCTTTTGAGCTTATTTTAAAAGCGATTATGTGCGCAGGCGCACCATATGTTGTTTGCTCGTGTGTTGGACAAGAAATCAGTGATCAGATGGAGCAACTGCACAGGAGCGCCTACGCCAGTAATTGGTGTGAAGAGCCGCCGAGAATCCGAAAAAATCTGCTTACTTTGATGATCATCACTACCAAACGTATTGACTTAAATTACAGGAAATTCGTGTCATTCAATCATGTTTGCTTGGCAACGGTATTGCAAGGTATTTACACCTACCTGATGCTGATAATCAACCTGGAGACAGATTAA

>OR81NTE

GCAACAGAAAATTTGTCTTCAATAAAGCAGTGTTATAGAAGAATCAGACTCAGCGGTCTGCTCCCTGAAACAAACAGTATATGGTACAAGATAATTAAGGTCTATCACATGCTCATGATAAATTACATCGGGGTGGCTGCGGTCTTAGATATTTTACTGAACAAAAACAATTTAATTGATTTTGTGCAAGCAATCTCGGCAGTGATGATCTATATGCATAACAATACCAAAGCTGTGAACATCTATGCACGACAACAAGTGGTCAGGCAATTGTTTGACAGGCTTGACAATTTTAGCAAGGAAAGTGCCAGAGATAAGGCCAACCAAACTCGTAAACAATCTTTTCGAGAAAAATTCATTTCAGTTTACTGCCAATCATTTTTGTGGTTGCCAATATTTTCCTTTGTTGCCGGAGTGTTTGGCGACTATATGTCCGGCTTTGTAAAACCACACATACCCCTTCAGGTATGGCTACCTTGGAGTATGAAAACATTTTGGTCATATCTGGCAGGCATGGTGTTTATTACATTGTTGGAATTTTCCGTCTGTGTTTATTATATGTCAGGCATTGTGATCTTGTTTTCATATACAAACGAACTGGCTGAATGTCTCAAAGTTTTACAAAAACGTCTTGAGGTAAATGGTCCAGCTGATAAAACTGTTTATAAACATCATAAAGTTATAATCGAATTGTTACAAGAGTTCAATAAATTATTTTCTGGTCCCTATTATGTTGAAATACTCGCTACAACTTTACAACCATGTGGATTTGGATATGCATTTATCAAGATGTTAAATCGGTACGATCCCTCTGCTCCTGAAGTAATCCTTAAATTTCTATCCAGTGCTCTAGCGCCAGGTATCGTTTGCGCATGTGGACAACAAATTACTACTCAGATGGAACGACTACACGCCAGCGCTTACATGAGTAAATGGTATGAAGAGAAACCAAAGGTTCGTAGGGACTTATTGATACTGTTGACAATCACCTCTAAGAATATTGATTTAAACTACAGACACTTCATATCGTACAACTATGCTTGCTACGCAATGGTGCTGAAAGGCATATACTCTTATCTGATGATGATCTTCAGCTTCGATACAGAATGA

>OR82-FX

ATGGTTTTTTTGAATCCGAATTTGTCACCAGTCAGTGTAGCTTACAGCACTCTTCGATTTACTGGCCTAATGAATCAAGAGGGTAGACCATGGTCGCTGGTGATCTGTGCCTATCATATTGTTGCTATGAACTGTATGGGAATTACATCCGGTATTAGGCTGTTTTCCAGAACAGGATCATTACCAGAGATGATTGAGGCAATTTCAGCGTTTACAATTTACCTGCATGTCAATGCCAAAGCAGTTAACGTGCTCAGAAATCAAAAGGAAATACGACAGCTGTTAGAAAAGCTGGAAGAAATCCGTTATGGTATCCTACAAGATGTGGAAAATCGACATCATTTACTAAAGACAGATAATTTCCTGGCAAAATTAACTTTTAGTTATGGAACATTATTTCTGAGTTATCCATTTTTAGCGTTAATCATGAATCTAATTATTGATTTTACTTCACCATCGGTAAAGCCCCATTTAGTGCTTCAGGTTTGGGTTCCTTGGAGTATGAAAGAGTTCTGGCCGTACATCATTGGAATGGTAGCTGTGATGTTATTGTCTGTGACTGCTCTCATCTACTACGTGTCGTTCAGCATCCTTCTGTACACCTACAGCTTCCAAATGTCCGCATTTCTGAAAATATTACAAGCGAGATTAATCAAAAATGGACCAAGAGATCCTGAATTGTTTCGATTCCACGCTGAACTTCTAAGATTATTAAGAAACTTTAACAAACTCTTCTCTGGCCAGCTTTACTTGGAGACAATTCTATCTTCATTACAACCGTGTGGCTTTGGATTTGCATTGATCAAGGCATTCAAAAAAAAAGATCCAGGTGCTTTTGATTTACTTTATAAAAGTTTCATGGCAGTTCTTGCTCCGTTCATTGTCTGTGCATGTGGTCAGGTGATCAGCACGCAAGTTGGCAAATTACATGAAAGTTCTTACTTGAGCCGATGGTACGAAGAACATCCAAAAATTAGAAAAAACTTGCTGACCATGATGGGAATGACCGTCAGGCCAACCACGTTGAACTACCGCATATTCACTGCATTCGATTATATCTGTTTTGCCAGGGTTGTTCAAGGAATCTATTCCTATTTGATGATGATCATCAATTTGGAAACTGACGACTGA

>OR83-FX

ATGGAAAAAAAAACTAACAAGTTGTCGCCAATAAAACAGGCTTTCAGAGCAATCAGATTCAGTGGTCTTATCGTAGAGAAGGAAAATCAGTGGGTAATATCGCTTACCGTCTATCATATGCTGATGATAAGTTACGGCGCCATTGCAGTCGGTATAGAATTATGTTTAATGGAGGAAAATTTGTTACAATTTGTGGAAGTATTGTCAACCAGCATGGTTTACATCAACGACAGTTCCAAAGCTCTGAATATATTGTTGCATCAAAAGAAGATTAAAGATTTGTTCAACAGGCTGGATAAATTCAGTTTGAAGATTTTACAAGATCAAGAAGCTGGCGAACATGTAAAGAGCATTGATAAAGAAAACCTTTTTGCGAAATCTTTTCGAGTAAAGTTTATATCAGTTTTCATTCCGATAATGCTATGGTTACCGCCTATATCACTCATCACTGGCATCATCGGTGATTATTTAACAGATTTTGTTAAACCACATTTACCGTTACAGTTATGGTTACCATGGAGTATGGATGAATTTTTGCCATATTTAGCTGGAATGGTATTCGTTACAATGCTGGAATTAACTACCTGTATTTTCTATACGTCATTCACAATTATGTTTTTAACATTCACCAATGAACTGGCAGCATGTTTGAGAATATTACAAGCTCGACTGGAGACGAATGGACCGACCGATAAAAATATCTACAAGTATCATAAAGTGATCATAGACATTCTACAAACATACAATGGCATATTTTGCGGCCCACTCTACATTGAGATCTTAATCTCCACTCTGCAGCCGTGCGGTTTTCTTTACGCTTTCACTAAGGTTTTTAAGAACTACAATCCAGCCGCATTCGAGCAGATTCTAAAAGCATTGTTATGTGTGGCCGCACCCTACATAGTCTGCTCATGTGGACAGGAAATTAGTAATCAGATGGAACAGCTACACAACAGCGCCTATGCGAGTAAATGGTACGAAGAGTCGCCTCGAGTGAGAAAGGATTTGCTCACCATGATGATCATTACCACCAAACGTATTAATCTCAACTACAGAATGTTTATCACCTATAATTATGTCTGCCTCGGCTCGGTATTCCAAGGTATATACTCGTACCTAACCATGATCATCAACCTTGAGGCGAATTAA

>OR84-FX

ATGAAGAAACAGAAATTACCTGGGACACCTGAAATGATGACACCGGTCCAGCGGGCCTATCATCAAATCAGATTCAGTGGGTTAATTCTAGAAAAAGATAATCCAAAGACCATATTTTTTGCAGTGTACCATATGTTTATGGTGAACTATGTAGGTCTAGCTTCAGGTGTAGATTTAATAAGAGGAAATCAAAGTTTAATAGATTTTGTACAGTCACTGGGATTAGCAATGATCTACATAAATAACATAGTTAAAGCTACAAATATATTGTTACATCAGAAGGAAATTAAAGAATTGTTTGCCAGATTAGACAAAGCCAGCTTGGAAATTACACGTAACCAAGCGGAAAAACATATAGAACTTCGTCCGGAAAATCTACAAACAAAATTTATAGTGACTTACTCAAAATTGCTCATGTGGTTTCCACCATTTTCGTTTACTACCGGTTTACTTAGTGATTATCTGTCGGACTTCGTTAAACCTCACCTACCATTTCCGTTGTGGATACCTTGGAGAATGACAACATTCTGGTCATACTTGGCTACCATGGTGTTTGATACAATGCTTTCAGTAACCACGAGCTACTACTACGTATCGTTCACTGCCTTACTGTTTACATTCACAACTGAGCTCACCGCCTGTTTACGGGTACTGCAGCATCGTCTGGAAACTAATGGACCAGCGGACAAGAATGTCTACATATATCACCATACGATTCTTGAACTGTTACAAGATTACAATAAAATATTTTCTGGACCTGTGTATTCAGAAATTTTAATATCAGCTTTACAACCTTGTGGCTTTCTCTACGCTTTTATTAAGCTTCTGAAGCGACAGGATCCAGCAAGTGTAGATTTAGCCACTAAAGGTGTATTGACGGTTTTAACACCCTTTGTACCGTGCTCTTGTGGACAAACTATAAGTTCTCAGATTGAACGACTACACGCCAGCGCTTACATGGGCAAGTGGTATGAAGAGAAACCAAAAGTGAGGAGGGACTTGCTTACTATGCTGACAGTGACAACACGACAGGCTGATATGAATTACAGGAAATTTATATCCTACAACTTTGTCTGCTATGCTACGGTAATCCAAGGAATATACTCTTATCTGATGATGATCATACAGATCGGAGGTGATTAA

>OR85NTE

GCACCTAAAGAGATGACACCAGTTCAGCGGGCCTATCACAAAATTAGATTTAGTGGGCTAATTGTTGATAAGAACAATCCAAGATCCAGATTTTTTGCGATATACCATGCATTTATGCTGAATTATGTTGGCCTAGCTTCAGCTATAGCATTAATAAAAGGAGAACAAAGTTTAGTCGATTTCGTACAAGTATTGGGAGTCGCAATGATCTACATAAATAATGTGGTAAAAGCTGCAAATGTATTTCTACATCAGAACGAAATTAAGGAGCTATTTGCCAGAATGGACAAATTCAGCTTGGATGTTACACACGATGAAGCGGAAGAACATATTGAACTTAGACCAAAAAATTTACAAACTAAATTTATATCACTTATCTCTAACGTTCTGGTGTATTTACCACCATTTTCGTTGGCCATCGGTGTACTTAGTGATTACCTGACAGACTTTGTTAAACCCCATCTTCCTTTTCAATTGTGGATACCTTGGAGAATGACAGCATTCTGGCCCTACTTCGCCGCCATGGTGTTTGATACATTACTTACGGAAACCACGGGCTACTATTACATGTCCTTCACAACTCTATTGTTTACATTCACAACCGAACTGAACGCCTGTGTCCGGGTACTCCGACATCGACTGGAAACTAATGGGCCAGCTGATGAGAATGTCTACAGATATCACCAAATGATCCTAAAACTGTTGAAAGATTACAATAAAATGTTTTCTGGACCTGTTTACTGGGAAATCTTGATATCAACTCTACAACCTTTGGGTTTTATTTATGCATTTCTTAAGCTCTTAAAACGAAAAGATCCTGCTAGTACGGAAGTAATCATCAAAGCGCTACTATGTCTCGGGGCACCCTTTGTTCCATGTTCTTGTGGACAAGCCATCAGTACTCAGATGGAACGACTGCACGCCAGCGCTTACTTGGGCAAGTGGTACGAAGAGAAACCAAAGATCAGAAAGAATTTAGTCACTTTACTGACAGTGACCACCCAAAAAACAGATCTGAACTACAGGAAATTCATCTCCTATAACTTTGCCTGCTATGCCACAGTTATTCAAGGAATTTACTCCTATCTGATGATGATCATCCAATTTGATGATGATTAG

>OR86NTE

ACACCGGAAATAATGACAACTATCCAGCGGGCCTATCACCAAATTAGATTTAGTGGGCTAATCTATGACAAAAACAATCGAAGATCTGCATATCTGGCAATACACCACATGGTCATGGTGAACTATGTTGGTATGGCAACAGCTGTAGCTTTAATAAAAGGAGAACAAAGTTTAGTCGATTTTGTACAAGTATTGGGTGTCGTAATGATCTACATACATAATGTGATTAAAGCAATAAATATATTGCTTCATCAGAAGGAAATTAAGGAGCTATTTGCAAGATTGGAAAAATTCAGCTTGGAAATAACAAATGACGAAGCGGAAGGGCATATAGAATTACGCCCGAAAAATTTACATTATAAATTTATAACAATTTTCTCAAGAGTGTTAAAATACTTATCACCATTTTCATTTGTCATGGGTATACTCAGTGATTACTTGTCGGACTTTATTAAACCGCATCTACCACTACATTTATGGATACCATGGAAACTGACCCCGTACTGGCCTTACTTCGCCGGCATGGCGTTTGATACATTACTTATGGTCACCACGTGCCATTACTACGTGTCGTTCACAGCATTACTCTTTGCATTCACAACCGAACTGAACGCCTGTATTCGGGTACTTCGACATCGTCTGGAAACTAATGGACCAGCTGACAAGAATGTCTACAGATATCACCATACAATTCTTGAACTGTTGAAAGATTACAACAAACTGTTTTCTGGTCCTGTATACTGGGAAATACTAGTATCAACTTTACAACCTTGTGGCTTTGTTTATGCATTTATTAAGCTCTTGAAACGAAATGATCCGTCTAGTTCGGAGATGATCATCAAAGCATTACTGACCTTAGCGGCGCCCTTTGTCCCTTGCTCTTGTGGACAGTCCATCAGTTCCCAGATGGAACTACTACACGCCAGCGCTTACATAGGTAAGTGGTATGAAGAGAAACCGAACATCAGGAGGGACTTGGTAACTATGCTGACGGTGACAACACGACAGGCTGATCTGAATTACAGGAAATTTATATCCTACAACTTTGTCTGCTTTGCCTCGGTAATCCAAGGAATATACTCCTACCTGATGATGATCATCCAATTTGAAACTGATTAG

>OR87-FX

ATGAAGGGTCAAGCTGAGACCCCGGAAGTGATGACAACCATCCAGCGGGCCTATCACCAAATTAGATTTAGTGGGCTAATTGTGGAAAAAAACGATCAAATTTCCATATTTTTGGCAATATACCACATGTTCATGGTGAACTATGTTGGTATGGCTTCAGCTATAGCCTTAATAAAAGGAGAACAAAGTTTGGTTGACTTCGTACAAGAATTGGGACTCGTAATGATCTACATAAATAATGCGTCTAAAGCTGTAAATATATTGCTACATCAAAACGAAATTAAGGAGCTATTTGCCAGATTGGAAAAATTCAGCTTGGAAATTACACATGATGAAGCGGAAGAACATATAGAATTACGTCCGAAAAATCTCCATTCTAAATTTATATCCATTTACTCAAAAGTGTTAAAATACTTACCACCATTTTCATTTGTCATGGGTATCCTCGGTGATTACTTGACGGACTTTATTAAACCACATCTACCATTTCAGTTATGGATACCATGGAAACTGACCCCGTACTGGCCTTACTTCGCCGGCATGGTGTTTGATACATTACTTATAGTAACCACGACCCAGTACTACATGTCGTTCACAGAATTACTATTTGCATTCACAACCGAACTGAACGCCTGTATTCGGGTACTTCAACATCGTCTGGAAACTAATGGACCAGCTGACAAGAACGTCTACAGATATCACCATACAATTCTTGAACTGTTAAAAGATTACAACAAACTGTTTTCTGGTCCTGTATACTGGGAAATACTAGTATCAACTTTACAACCTTGTGGCTTTATTTACGCATTTATTAAGCTCTTGAAACGAAATGATCCGTCTAGTTCGGAGATGATCACCAAAGCATTACTGACCTTAGGGGCGCCCTTTGTCCCTTGCTCTTGTGGGCAGGCCATCAGTTCCCAGATGGAACAACTACACGCCAGCGCTTACATGGCTAAGTGGTATGAAGAGAAACCAAAAGTGAGGAGAGATTTGGTAACTATGCTGACGGTGACAACACGACAGGCTGATCTGAATTACAGGAAATTTATCTCCTACAACTTTGTCTGCTATTCTACGGTAATCCAAGGAATATACTCCTACCTGATGATGATCATCCAATTTGATACTGATTAG

>OR88NTE

GGGGTAAGAGGATTTAAAATAAGGAAGAATGTACAATACTCTGGATATCAATTAGCAAGATGGTGTGGATTTTTTTATGATAAGTCATGGACCACTTTTTGGAATATCACAAGAGCTCTCATCTTAGTAATAACTAGCGTCATTCACAAATTTCTTGCGATTCTTCTCAGTAAGGATCTTGATATATTGGAAAAGGTGCTCCTTGTAAACTATACAACTTTAGGATCTTCTTGTGCATTAATGGCATTGTCACTAATCTATAAATCTAAAAAGATGTCATTGTTTCTATCAGTAATCAAGTCTGAATTCTACCAACCTGAAATCAAATTATCAAGAAAACAACAGGCTATAAAGAAGAAAACAAATACATTTTTGCGGCTTTTCATCAATAACATTGCCATTTATTATCATTTTTCTATGGTAATCAACTTCTTCAGACGTCCTTTAATGGAAGGTCTTGATTTGAAGATACTTCCTCTACCTGGATGGTTGCCTTTTGAGATGAATTCATGGTTTAGATACATTGCTGTGTCAACATTTCAGTTCATGTCGGGTTTTAGCTTAGTGAATACCCATGTTGGAATGATTCTAACTTTTGTAATTCATTCTAAATTACTGTGCGATGAATTTGAAATATTATCAATTTACGTTGAGGAAACATTTGAACGTAATTTCGGAGAACTTATGGAACTATCAGAAACAAATGAAAAAAGACAAGAATTTATCAATCGTCGGTTGACCTATATAATCCGTCAGCATGGTAAACTGCTAAGTTATTTCGAGTTATTCCAAGAGATTTACAGCATGTTTTTATTTCTATTTTCTGCTTGCGCTGGAGCTTTGTTATGCACAGTGGTCTATATAATTACGGATCCCAATTCAAAAAACATGACAATAATAATTAGCGCTGGATGTGTACTTCTAACAGAAGCTGTTTTTGTTGGGATTTACTGTTGGTTTGGCCAAGAAATTACTAATAAGATTGCTAACGTTAGAGAAGCTGTGTATTTTATTCCTTGGTATGAGGAATCTGTGAGTACTCAGAAGTCTTTGCTTAATGTACTGACCGCTTGTAATCGAGATAGGATTGTTAAAGGAGGTGGACTTCAGGAATTTTCAATGAAAGGTCTTTGCGAGCTATATCAAGCATCGTTCTCCTACTTCAACATGTTGAACGCAATGCGCTAA

>OR89-FX

GGAGTAAGAGGTTTTAAAATAAGGAAGAATGTACAATACTCTGGATATCAATTAGCAAGATGGTGTGGATTTTTTTATGATAAGTCATGGACCACTTTTTGGAATATCATAAGAGTTCTCATCTTAGTAATAATTGGCGTCATTCACATTTTCCTTGAGATTCCTCTCAGTAAGGATCTTGATTTATTGGAAAAGGTGCTCCTTCTAAACTATACAACCTTAGGGTCTTCTTGTGCATTAATGGCATTGTCACTAATCCATAAATCTAAAAAGATGTCATTGTTTCTAACAATTATCAAGTCTGAATTCTACCAACCTGAAATCAAATTATCAAGAAAACAACAGGCTATAAAGAAGAAAACAAATACATTTTTGCGGCTTTTCATTAATAACATTGCCTTTTATTATCATTTTTCTCTTGTAATCAACTATTTCAGACGTCCTTTAATGGAAGGTCTTGATTTGAAGATACTTCCTGTACCTGGATGGTTGCCTTTTGAGATGAATTCATGGTTTAGATACATTGCTGTATCAACATTTCAGTTCATGTCGGCTGCTAGCTTAGTAAATACCCATGTTGGAATGATTCTAACTTTTGTAATTCATTCTAAATTACTGTGCGATGAATTTGAAATATTATCAATTTACGTTGAGGAAACATTTGAACATAATTCTGGAGAAGTTATGGAACTATCAGAAACAAATGAAAAAAGACAGGAATTTATCAATCGTCGGTTCACCTATATAATCCGTCAGCATGGTAAACTGCTATGTTATTTCGAGTTATTCCAAGAGATTTACAGCATGTTTTTATTTCTATTTTCTGCTTGCGCTGGAGCTATGTTATGCACAGTGGTTTATATAATTACGGATCCCAATTCAACACTCATGGCAATAATTATTGGCGTTGGATGTGTACTTCTAACAGAAGCTGTTTTTGTTGGGATTTACTGTTGGTTTGGCCAAGAAATTACTGATAAGATTGCTAACGTTAGAGAAGCTGTGTATTTTATTCCTTGGTATGAGGAATCTGTAAGTACTCAGAAGTCTTTGCTTAATGTACTGACCGCTTGTAATCGAGATAGGATTGTTAAAGGAGGTGGACTTCAGGAATTTTCAATGAAAGGTCTTTGCGAGCTATATCAAGCATCGTTCTCCTACTTCAACATGTTGAACGCAATGCGCTAA

>OR90NTE

GGGGTAAGAGGATTTAAAATAAGGAAGAATGTACAATACTCTGGATATCAATTAGCAAGATGGTGTGGATTTTTTTATGATAAGTCATGGACCACTTTTTGGAATATCACAAGAGTTCTCATCTTAGTAATATCTGGCATCATTCACATTTTTCTTGCGATTCCTCTCAGTAAGGATCTTGATATATTGGAAAAGGTGCTCCTTCTAAACTATACAACTTTAGGATCTTCTTGTGCATTAATGGCATTGCCACTAATCTATAAATCTAAAAAGATGTCATTGTTTCTATCAGTAATCAAGTCTGAATTCTACCAACCTGAAATCAAATTATCAAGAAAACAACAGGCTATAAAGAAGAAAACAAATACATTTTTGCGGCTTTTCATCAATAATATGGCCTTTTATTACCATTTTTCTCTTGTAATCAACTTTTTCAGACGTCCTTTAATGGAAGGTCTTGATTTGAAGATACTTCCTCTACCTGGATGGTTGCCTTTTGAGATAAATTCATGGTTTAGGTACATTGCTGTAACAACATTTCAGTTTATCTCGGCTTCTTGCACAGTGAATACCCATGTTGGAATGATACTAACTTTTGTAATTCATTCCAAATTACTGTGCGATGAATTTGAGATATTATCAATTTACATTCAAGAGACATTTGAACATAATTCCGGAGAAGTTATGGAACTATCAGAAACAAATGAAAAAAGACAGGAATTTACCAATCGTCGGTTGACCTATATAATCCGTCAGCATGGTAAACTGCTAAGTTATTTCGAGTTATTCCAAGAGATTTACAGCATGTTTTTATTTCTATTTTCTGCTTGCGCTGGAGCTATGTTATGCACAGTGGTCTATATAATTACGGATCCCAATTCAACACTCATGACAATAATTATTGGCGTTGGATGTGTACTTCTAACAGAAGCTGTTTTTGTTGGGATTTACTGTTGGTTTGGCCAAGAAATTACTGATAAGATTGCTAACGTTAGAGAAGCTGTGTATTTTATTCCTTGGTATGAGGAATCTGTGAGTACTCAGAAGTCTTTGCTTAATGTACTGACCGCTTGTAATCGAGATAGGATTGTTAAAGGAGGTGGACTTCAGGAATTTTCAATGAAAGGTCTTTGCGAGCTATATCAAGCATCGTTCTCCTACTTCAACATGTTGAACGCAATGCGCTAA

>OR91NTE

GTGGTAAGAGGATTTAAAATAAGGAAGAATGTACAATACTCTGGATATCAATTAGCAAGATGGTGTGGATTTTTTTATGATAAGTCATGGACCACTTTTTGGAATATCACAAGAGTTCTCATCTTTTTAATAATTGGCATCATTCACCTTTTGCTGTCGATTCCTCTCAGTAAGAACCTTAATATATTGGAAAAGGTGCTCCTTCTAAATTATATAACCTTATCATCTTCTTGTGTATTAATGGCATTGCCGCTGATCTATAAGTCTAGAAAGATGTCATTGTTTCTATCAGTAATCGAGTCTGAATTCTACCAACCTGAAATCAATTTATCAAGAAAACAACAGGCTATAAAGAAGAAAACTAATACATTTTTGCGGCTTTTCATCAGTAACCTTGCCATTTATTACCATTTTTCTCTGGTAGTCAACATGTTTAGACGTCCTTTAATGGAAGGTCTTGATTTGAAGATACTTCCTTTACCTGGATGGTTGCCTTTTGAGATAAATTCATGGTTTGGATACATTGCTGTGTCAACGTTTCAGTTTATCTCGGCTTCTTGCACAGTGAATACCCATGTTGGAATGATACTAACTTTTGTAATTCATTCCAAATTACTGTGCGATGAATTTGAGATATTATCAATTTACATTCAGGAAACATTTGAACATAATTCTGGAGAAGTTATGGAACTATCAGAAACAAATGAAAAAAGACAGGAATTTACCAATCGTCGGTTGACCTATATAATCCGTCAGCATGGTAAACTGCTAAGTTATTTCGAGTTATTTCAAGAGATTTACAGCATGTTTTTATTTCTATTTTCTGCTTGCGCTGGAGCTATGTTATGCACAGTGGTCTATATAATTACGGATCCCAATTCAACACTCATGACAATAATTATTGGCGTTGGATGTGTACTTCTAACAGAAGCTCTTTTTGTTGGGATTTACTGTTGGTTTGGCCAAGAAATTACTGATAAGATTGCTAACGTTAGAGAAGCTGTGTATTTTATCCCTTGGTATGAGGAATCTGTGAGTACTCAGAAGTCTTTGCTTAATGTACTGACCGCTTGTAATCGAGATAGGATTGTTAAAGGAGGTGGACTTCAGGAATTTTCAATGAAAGGTCTTTGCGAGCTATATCAAGCATCGTTCTCCTACTTCAACATGTTGAACGCAATGCGCTAA

>OR92-FX

ATGTATAATTTTTTAAAGAAAATGAAACAGCCGCAAGGCCGCATGCAAGGGAAAAAGAAGAGTGTAAAGTACACTGGATACTGGGTTTCCAAATGGAGTGGATTTCTGTATGACAAATCGTGGACATCTGTATTCCACATAACAAGAGCGTTGTCATTATTAATTTTTGCAATATTCCATACATTGTTAGCGGCAAAATTCAAAGAAAATATTGACATCTTAGAGAAGATGTTATTAGTAAACTTCGGTATTCTTTACATAAACAGTGCCCTAATGGTTTTTCCATTACTGCACAATTCCAAAAATATGATATCATTTTGTTCAGTTATCCGAATGGAATTTAACAAACCCATAATAAATCTATCACTAAAACAACTGGCAGTAACAGAATATACCAGAAGATATGTGCGACAATTTAGTAGGAAAGTTGCGTGCATTTTGTACTCCTCATTGCTATACAATTTTATGCGACGATTTATATTTGAAGGCTTCAGCATTAAGGTCTTACCTATTAATGGGTGGTTACCTTTCGAGATAAATACTTGGCTCAGGTATAGTGCCGTGTTTATTCTTCAGACTTTTGTATCAATAAATGTCATTACAGTTCATATTGGATTTTTAATAGTATTTGTTACTCATTCTAAATTCCTTTGTGAACAGTCTGAAATTTTATCATTACACCTACAGGAATCATTTGAAAATAATTTTCCTACTGTAAGTATTGGATTATATGAAAGAAATGTGGAACGAGCTAAATACATTAATTCCAGAATAAAAACTTCAGTTCAATTACACATTAATTTGTTGAAGTACTATAATTTATTTCAAGAGACTTACAGCATGTTTCTATTTATATTTGCCTTAAGTACGGGCGCCATGATATGCACAGTTGTTTATGTAATAACAGATCCTACTTCAACTTTAGTTCAATCAATTACTTGTTTTGGAACTCTGTTTTTACCTGAAGTATTTATTATAGGCATCTACTGCTGGTTTGGACAAGAAGTAACTAATAAGTACGAATGCATTCAGAATGCGGCTTATTCCACACCATGGTATACACAACCTGTGAGTACTCAGAGATTATTGCTTAATCTACTAACAATTTGCCAGCGAGAGCGGATTGTGATGGCAGGAGGACTTCAACAGTTTTCGCTTTTGGGATTTAGCGAACTCCTTCAGGCATCGTTTTCTTATTTCAATGTCCTCAACGCAATGCGTCAATAG

>OR93NTE

ATAATCAAAAGATTTAATAATCATAATCAAGGAGATGAAAAAGAGATAATTATAACTGGCTACTGGGTATCCAAATGGTGCGGATTTTTATACGACCAGTCGTGGACATCAGTAATTCACATCACCAGGCTGATCTTGTTTTTAATTCTAACAATTCTTCATATATTGATAGCGGTGTTATTGTACGAAGATATGAGTTTATTAGAGAAGGTGTTGATAACCAACTATGCTATCGTTTACATAAACTGCCTTTTTATGGTGCTCCCCTTGATTTACAATTCTAAAAAGAATCAATTATTGTGCAGTATAATCAAAACGGAATTTCAAGAAGCTAAAATACAGTTTTCACCCAAACAACTGGAAGTAAAGAAATATACAAAAAGATTTGTTCGCAATTTTGCATATAATGTTGCATTTTATCTTTATGGCTCTCTGATCATTAACTTTATCAGGCGATATTTATTTGAAGGTGTCAGCTTAAAGATCTTACCTGTTAACGGATGGGTGCCTTTTGAGTTGAATTCATTGATTAGGTACACTTTTGTATTTATCTTTCAAATAGTGGCATCAATTAGTGGCATGTCAGCTCATGTTGGATTTTTAATCATATTTGTGACGCATTCAGTGTCACTTTGTGAACAATTTGAAATTCTATCCATACACATCCACGAAACATTTAAGCATGTCTACTTCGGAGAAACAATGGAATGTTTTGAAGTAAATAATAACCGAGAAAAATATATACAGTTTAGATTAAAATATGCAGTCTATCAACATGGTAAGCTATTGCAATATTTCAATTTATATCAAGAGATCTACAGTATGTTCCTGTTTATATTCGCTCTCGGGACTGGCGCAATGATTTGTACAGCGGTGTATGTAATAACGGATCCTATGTCAAGTCTCTCGTTGGTAATTGTTTCTTTTGCTTGCTTATTAATACCTGAAATTGCTATCATAGGAATTTATTGTTGGTTTGGACAACAGGTAACTAATAAGTACTCATATATTCAAGAATCTGTTTACAATACACCATGGTACACGCAGTCTGTGACTACTCAGAAATTATTACAGAATATGCTGACAGCTTGCCAGCGAGAACGCATCGTGAAGGGTGGAGGACTTCAACAGTTTTCACTTTTAGGACTTAGCGAACTACTTCAGGTATCTTTCTCTTGGTTTAATGTACTAAATGCCATACGCCAGTAA

>OR94NTE

AATATAAAAAGATCTAATGGTCAAGAGCAAGACAATAAAAAAGAAGTATACACTGGGTACTGGTTGGCCAAATTGAGTGGATTTTTGTATGACAAGTCTTGGACTTCGATCTTTCATATCACTAGACTGATTTTGATTCTAATTCTGTCCATCATTCACACGTCAGTCCCGGTATTGCTAGGTGAATGTATAGATATCATAGAAAAGATGTTGATAATAAACTATATTATTCTTTCAATAAACTGCCTTTTAATGGCAATACCATTAATATTCAATTCCGATAAAAGTAGGCTACTTTGTTCAGTAATCGAAACGGAATTCCACCAGCCTAAAATAAAATTTTCCTCAAAACAACTAGCAATAAAACAATATACACAAAACTTTTTGCGAAAATTTGCGTTTAATGTTTCATTTTATTACTATTCTGCTATGGCCTTAAACTTAGGAAGGCGATATATATTTGAAGGCATTAGTATGAAAATTTCACCCCTTAAAGGATGGATACCTTTCGAGATGAATTCTTGGCTCAGATTTATTTTTGTGTTTATATTTCAAACTTTAATGACAGTTAATGCCTTGCAAGCGCATTTAGGGTTTTTAAACACTTTTGTTATACATTCCTATTCATTAAGCGCACAATTTGAAGTTCTATCCATACATATCCAGGAAACTTTTGAAAATTATTATCTTAAAGATGGAAAAGAAGTACCTGAAAGAGATTCAGTAGATCAAGAAAAATATATTCAATACAGAATTAAATATGCTGTTCAACATCACGGCAATCTCTTGAGATATTTCAGATTATATCAAGAGGCCTACAATATGTTTTTGCTAATATTTGCAATAAGTACTGGCGCCATGATATGTACGGTAGTGTATGTAATAACGGATCCTTCTTCCTCATTACTGCTAGTCATCATGTGTTTTGTTTGTTTATTTATTCCAGAAGTAATTATTATAGGAATTTATTGTTGGTTTGGACAGCATTTAACTAATAAGTATAAAAATATTCAAGAAGCAATATACTCTATACCTTGGTATACACAAACTGTGAAAACTCAGAAGTTATTACTTAATATACTAACAGCTTGTCAACGAGAGCGCATCGTTAAGGGTGGAGGTCTTCAAGAATTTTCGATGCTGGGACTTAGCGAACTTCTGCAGGCATCTTTCTCCTATTACAACATATTAAAAGCAATGCGTCAGTAA

>OR95-FX

AAAATGAAACATCCGCAAGGCCGCATGCAAGGGAAAAAGAGGAGTGTAAAGTATACTGGATACTGGGTTTCCAAATGGAGTGGATTTCTGTATGACAAATCGTGGACATCTATATTCCATATAACAAGAGCGTTGGCATTATTGATTTTTTCAATATTCCACGTATTGTTAGCGGCAAAATTCAAAGAAAATATTGGCATCTTAGAGAAGATGTTATTAGTAAACTTCGGTATTCTTTACATAAACAGTTCCCTAATGGTTTTTCCATTATTGTACAAATCCAAAAAAATTATATCATTTTGTTCAGTTATCAGAATGGAATTTCACCAACCCAAAATAAATCTATCACCAAAACAACTGGCAGTAAGAGAATATACCAGAAGATATGTGCAACAATTTAGTAGCAAAGTTGCGTATATTTTGTACTCCGCATTGCTATACAATTTTGTGCGACGATTTATATTTGAAGGCTTTAGCATTAAGGTCTTACCTGTTAATGGGTGGTTACCTTTTGAGATAAATACATGGCTCAGATACAGTGCCGTGTTTATACTTCAGATTTTTACATCAATAAATGTCATTACAGTTCATATTGGATTTTTAATAGTATTTGTTACTCATTCTAAATTCCTTTGTGAACAGTCTGAAATTTTATCATTACACATACAGGAAACATTTGAAAATAATTTTCCTACTGTAAGTATTGGATTATATGAAAGAAATGTGGAACGAGCTAAATACATTAATTCCAGAATAAAAACTTCAGTTCAATTACACATTAATTTGTTGAAGTACTATAATTTATTTCAAGAGACTTACAGCATGTTTCTATTTATATTTGCCACAAGTACGGGCGCCATGATATGCACAGTTGTTTACATAATAACAGATCCTACTTCAACTTTAGTTCAATCTATTACTTGTTTTGGAACTCTGTTTTTACCTGAAGTATTTATTATAGGCATCTACTGCTGGTTTGGACAAGAAGTAACTAATAAGTACAAATGCATTCAGAATGCGGCCTATTCCACACCATGGTATACACAACCTGTGAGTACTCAGAGATTATTGCTTAATCTACTAACAATTTGCCAGCGAGAGCGGATTGTGATGGCAGGAGGACTTCAACAGTTTTCGCTTTTGGGATTTAGCGAACTCCTTCAGGCATCGTTTTCTTATTTCAATGTCCTCAACGCAATGCGTCAATAG

>OR96

ATGAAAGGAGCGATGATTGTCAACAGGGAAAACATCTATACTGGTTATAAAATCGCCAAACATTTTGGTATAATATTCGATTATACTTGTATCTCAATATTGCAGTTAATTAGAAGTTATGGAATAAATATGATTGCTTTTTTCCATTTTTTAATTGAGATTATCACAAATTACCAGGAATATGATCTATTGGAGTTAATTGAAATGATACATTTCGCTACGTTGCACGCTAACTGTATGCTAATGTGTTCAACTTTAACATTTAATTCCAGAAACATCATAGAATTTACTGAATTAATCAAAACTGAATTTCATGAAGAGATACATACTTTGTCAGCAAAACAGAAGATAGTCGTCAGAAATACGCAAAAATTTATTTTAAAATTTATCCAAATAAGCGCCATGGTTCTATATTGTTCAAACGCTGCTACAATGCTCAAAAGGCCGATATTTTACAGCACAAAAGACAAAATATCATTGCCTTTCGACGGATGGTTTCCTTTTGAATTGAATTCAACAATTAGATTTGTTGGCGCTTTTACATTTCATCTTATCATTGGAATTAATATGGTGTCTGTACATTATGGAATGTTTTTAACTTATGTAGTTCATGTGCTACAGTTATGCGGGCAATTTGAATGCCTATCGGTTTATATTGATGAAACCTTTCAACATGCCCATTATTCTAATGGCAAACAATTGCAGAGTGGAAGCGAACAAAGAGAGGCGTTAATTAAATTTAGGACAAAGCTTATAATTAGCCAACATATTCATTTATTAAGTTATTTCGAGATGTTCTACATGTTGTATTCCAAGTTTCTGTTCGTTGCCGGCACCACTTCTGTTGCACTTATGTGCACAGTACTATACATAATTACGGATCCAGATGTGGAAGTAAAAGCAGTATTTACTGTGTTCGTCTTTCTCCTTTTACCAGAAGCAGTTTGCATTGGAGCTTATTGCTATTTTGGACAACAACTTTCAGATACGTGCTCAGCCATTCCTGAAACTATTTACTTTAACCCATGGTACTATGAATCAGTAAATATCCAGAAGTATCTTTTAAACTTACTAACAGCTAGTCAACGAATGAAGATAATTATGGCCGCAGGATTGCAGGAATTTTCAATGAAAGGCTTCAGTGAGATCCTGAAGGCTAGTTACTCATACTTCAACATGTTGCAGTCAGTTCGTTAA

>OR97-FX

ATGGATTTTAAAATTAGTAATGGTAAAAATGAAATGAAATTGGAGAAGAATTCAAAAGAGGAACGCATCTCAAATGGGTGGTTTGATGGTTTTAAACCTTGCAAATATGCTGGAACTATTTATGATAAAAGTGGGATATCATTATTTCACGTGATTCGATTCAATTCGATTCATATACTATCAATATTACATTTTGGTTTATTGATACTTCTGGAGTACGACACCATAGGTCTATTGACTATTGTGGAGAACATGCATTACATTATGATGCACATAAGTGGACTATGCATGGGTATTGCACTTTTATGTAATTCTAAAAAATTTATCACTTATTGCATATTAATAAATACGGAATTAATGCAACAAGAAATCAAATTACCTAAAATGCAAGAAGTTTATAGGAAAAACAATATATTTTTCAAATCTTTTATGAAAATGTCAACAAGTATGTTCATTTGGGGATTTATTGCCACATTTTTCCGGCGTCCATTATTTGATCGTAAAACGCATATGACTGTACCTCATGAAGGATGGGTTCCTTTTGAAATCAATACTTGGCCAAGATATTTTGCTGTTTGTACTTTCCAATTTGCAGTTGGATTGAATTTAGCAAGTGGACATTTAGCTACTGTTCTTTCCTATGTAATTCACGCCAAACATTTGTGTGGACAGTTTGAAATACTATCAATACTCATTCAAGATACTTTTAAAGATGATCTCAACGCCGAAAATAATAAAATAGAAGAAAATAAACCCCAAAGAGAGATGTTTATTAAATCAAGAATGTCCTACATAGTGCAAAGACATCATCTGCTTTTAAAATGCTTTGATTTATTTCAAAGTTTATACAGCGCATTTCTTTTTACTCTGAGTTTAACGTCTGGTATAATGTTTTGTACACTTATCTATATGATTACCGATCCTAAGAGTTCCTCATTTTTGATTACAGAATTTGTAACTTTACTATTTTCTGAAATGACAGTTATTGGATGTTATTGCTGGTTTGGTCAAGAAATGTCTGACTGGTGGGACCATATTAGAGAAGTTGTTTACAACATTCCATGGTATAAGGAATCAATTAAAACTCAGAAAACTCTTCTAAATATCATAACTGGAAGCACCAAGGACAAAACCATAATGGCTGGAGGCTTGCAGGAGTTCACCGTCAAAGGTTTCAGTGAGATGGTTCAAGCTTCGTTTTCTTACTTTAATATGCTGAAAGCGGTGAGGTAA

>OR98-FX

ATGAAAATCATCGAACAATTATCTCATCACCAAATCCTGAAAAAGAAGAAGGCTTCTAAGAAAAACAAAAAACTGGTATTCTATACTGGCTACAAGTTATTTCTGTATTATGGCTTTACTTACGACCATTCATGGATGTCTATTCTGAATATTTTAAGATATTTAACAGTGACTACCACCGGTTTAATGCATATTATATTGGCGATAGCGTTCAAGTATGAATCGTTTGATCTGCTGACTCTAGTGGAATTCGCTCATTATGTAGTAATGGCCATTTATTCTTTGACCATGTGTGCAGCACTGGTCTCCAAATCAAGAACCTTTAATGCATTATGTGCAACACTAAAAGAGGAATTTGAATTTCCAAAAATGAAACTATCACCTAAGCAGGAAGAAATTCACCGCAATACCAGTATTTTCATAGAAACTTTTATGAGAACTACTGCAACTGTAATTCTATGGGGCATTTCTGTCACTTGTATGAGGCGTCCTATTTTTGATAGACAAACAGAATTTACTATTGTATATGATGGATGGCTTCCATTTGAAGTGAATACTTGGTTTAGATACATAATTGTTTGTATTGGTCAAGTAATAATAACAATAAATGCATCATATGGGATGTTTAGTTGTCTGATTGCATTTGTAGCTCAGGCTAAACAATTGTGCGCACAGTGTGACATTTTGTGTGTTCATATAAAAGAAACATTCCAGTGTGTAAATTTTGACAGCAAAACTTCATCAGATAGGGAAACATTCATTAAATCAAGGTTGAAGTTCGCCGTAAAAAGGCATCATGTTCTAATGAGGTGTTTCAATATGTTTCAAGATATTTATAATTCATTTTTATTAATGCTAACTTTATGTTCCGGCATAATCTTTTGCACAGTTATTTACATGATAACTGACCCAAATAGTAATCTTTCGATAATTGCCGAATTTGTTAGTCTTCTAGTACCAGAAGTGACTATCATCGCATGTTACTGTTGGGCTGGGCAACAAATGACTGATAAGTGGTCGCAGATAAGAGGCGCAATATATGAGGTTAACTGGTACAATGAATCCTTAAGGACCCAGAAAACTCTTCTTAACATGTTAACTTATTGTACCAAAGATAAAATTATAACGGCAGCTGGCATACAAGAGTTCTCCATGAAAGGTTTTAGCGAGCTGTTACAGGCATCATTTTCCTACTTCAATATGCTTAAAGCGCTTCGTTAA

>OR99F-FX

ATGCAGTTTAATTTTGATAAAGAAGAAACAATACTTTATGCAATTCAGAAGTTGTGCTTTTATATGGGCTTTCTGTATGATAACAACAGTTGGGCATCTATATTTCTCGTGCTTAAGGCCGTGATTATGGTTCTCCTGTCATTTCTTCACTTCACCTTAGCGATTATACTAGATTCGGAGAAAGTGGGAAGGTTGAGCCTCATGGAAATTTTTCACTACTCTACAGTTGAAGGATATAGTCTGCTTGTGGTATTATCCTTTGTATACAAATCTAAACAAATCAAATTGTTCCGTCAAGCTTTAAGTGAAGAGATAAAACAGCGTAAAATGTTATTAACACCAAGACAAGAAAAAATCACAATAGATCTTAACAAATTTTTGGAAATATTCTGGAAATATGCTTATGGATTTATTATAGTTGTTGGTTTTCTTACACTAGTTAGGAGACCGTTATTTGATCGTGGATCAGCAATGGCAACTTGCTATGAAGGATGGCTTTGGTTCGAAGTGGATACATGGCCTAAATGGGCTGGAGTAGCTATATTCCATGTATTAATAGCAGTGCACACTGTCCACATACAGTATCTTTCAATGATTTCACTTACCACGCATGTTAAATATTTAGGATCATTGTGTGAAATTTTGGCAGTGTATATAAAAGATTCATTTGAACGTAATTATTATGATGAACTTAATTTATCATATAAAAAATACATTAGTCGAGAAAAGTTTATTAAGATGAGAGTTAGCAAAATAATCCAGCAACATCAACGCTTGTTAAGTTTGATTGATATGTTCAAAGATTTGTACAACGCATTTTTACTGTCAGTGGCCCTCGTTACTGGTTTAGTGATCTGTACACTAATTTATATCGTAACTGATCCAAATAGTTCAACAACTTTGATCATTCAATGCACAAGTTTACTTGCAGTTGAAGGTACTTATGTTAGTATTTATTGCTGGGTTGGCCAACAGTTAACTGACAAATGTGCAGTTATAAGAAACGCTATTTTTGATATCGCTTGGTACGATCTGCCAATAAGTGAACAAAAATCACTTCTTAATATTATGATTGCTTGTACCAAGGATCAGGTTCTAATGGCCAGCGGACTACAAGAATTTTCAATGAAAGGTTTAAGTGAGCTTCTGAAAGCAACTTTTAGCTGCTTCAATATGTTGAAAGCTATTCGCTGA

>OR100NTE

TTAATAAACCTGTTGGTCCAAAACCCTGAAGAAAGTACTTTCTACATAGGAGCAAAGATAGTAAAAAACGTTGGTGCTTATTATGACAACACTTGGGCCGCTGTGTTCCACATATCAAGAAATTTTCTAACCCTCTTAACTGGGATGATGCAATTTATTTTGCAGATTATATTCAACTTCAACAAAATTGAAAAATTAACAATTCTGGAGATAATACATTTCATTTTTTTGTACTTGTTTGCTATTTCATTAAGTTGTGGTCTGTTAGTAAGATCACGAAGATTTAAGTCCTTGTATACCTTAATTAAAAATGAATTTGGCAAAGAAACAAAATCTTTGACTGTTGAACAACAGAAAGTAGAAAAAGATGCTAGTGACTTTACACATCGATTTATAAAAATATCATTAGTATTATTTTATTGGGCTTGTATATTTACCATTAGTAGACGACCATTATTTGAAAGGACTTCTGAAATGACTTTAATGAATGAAGGATGGATGCCTTTTGAAGTAAACACCTGGTTTAGGTATACTGTCGTATGTATTTTTCAAACTGTAGTAGCTCTAAACTTAGCTACAATATTTTGGGCTCTCTTGGCTACATATGTGACATATTCTAAGCAAATTTGCTCACAGTTGGAAATATTATCACTTTATATAAAAGATACCTTTGATTACAACTCTCCAGCTCTCACTGAAACAAAATCATCCAAATTAACAACAGCTAAATACAAGAAGCTCAGATTAAAGCTGATAATCCAACGTCATCAAGTCTTGTTAAGATGCCTCAACTTATTCCAAGAAACTTACAGTTTATTATTGTTTGGTGTGAGTTTTAATTGTGGATTGGTTATGTGCACAGCTATTTATATGGTAACTGATCCACAAAGTAATTTTTTCTTAATTCTTGAATTTGTTAATCTTCTTGTACCAGAACTGGCACTCACTGGATGTTACTGTTGGTTTGGTCAACAAATGAAAGATTGGTGGACTAGAATCGGAGAAACAGTTTATGATTCCTCTTGGTATATGGAACCAATAACAATTCAGAAGGCTCTTTTAAATATGTTAACAATAAGTAATAAAAATAAGGCGCTTAAAGGAGCTGGTTTGCAAGAATTTTCTTTAAAAGGCTCTAGCGAGTTACTTCAAGCATCTTTCACTTACTTTAACATGTTGAAAGCAGCTCGGTAA

>OR101

ATGGCCAATTCAAACTCGATTCCCAGCAACATCAAACCAAAAGTACTACGAGTGCCGTTGCACACATTACAAATAGCGGCGATGTGGCCACCTTTTACCATAAATCGAGTTATTAAAACGTTAATGAGGATATATTTGGTGAGCTCAGCCATTCTTCTAGCATTATGTACTGTGGGGCTGTTGGTGAAAACTATAACAACCAAGGATTTGGTAGATAGAAGTGAAGCAATAGATATAATAACCTTGACATCTTCAGCTTTGTACAAACAACTATTTTTCGTGAAACATTATAATGAATTAAATGATTTGGTAATGACAGCTGATATTTTAGAGGTCCCAGTTGGTTGGCTCAAATATCCACCGTATTTATCCGCAGCTCATTGCACTTGTGGTTTCTTAGCTATTGGGTTCTGGTGGATGGTTCCGATACTCAAGGTATCTTTTGGTATCACAACTTTGCCTGAGATGCGACTACCAATGAATGTTTACGATTGGGGACCTACAGGTTGGCAATTTTGCGCTATTTATGTTTGTTGTTGTTTTGGTCTGGCGTTTTCCACTCATGTGTACATGGCAGTAGATGGATTCCTATTTACAGCAGTCTACACAGCTAATGGAGCTTTAAAACTTCTGGCAGATAGAATAATGAAAATACGTTCTATAAAAACTGAAGGAAAGGAAACACTAAATGAAATAATATTATCAGAACTCAAGAAATGCGCTAAGACTCATGCTCTTATACTACAATTTATTAGAAAATTGGAAGTTATATTTAGATCGCTGATTGTAGCCGATGTGTTGCATGCTATTATCTCTCTCAGTTTTGCCATGTTGCAAATGAGTGAATCAAAGGGAATACTAGAGTGGACCAAAATGGTATTCTTTATTTTTTACTGTTTTCTACATCAGTATCTCAACAGTTTTTTTGGCCAAGATCTAATTAATAAGCAAGAAAATCTTAGATATGTTTTGGAAGAAGTTCCTTGGGAAGAATGCTCCTTACCGGTGAGAAGTACCACTCTGATCATGATTACAGCTACAACAAATCCAATCAAGCTCAGTGCTTGGAGAATGTATTTCTTACAGTATGCGACATTTTCAGAGTTCGTCAAAAATATGATATCTACTTATATGGTTCTAAGGCAACTGCAGGATAATTCTGTTTAA

>OR102

ATGGGAAATTCTAGAGCAACATTTAAGAAAAAGTCATCAGTTTTAAGAGTTCCGTTGATTGCACTAAAAATCATAGCGATGTGGCCTTTAGATTCAAAGAATTTGGCCGTTAAATTTTTAATGAAGATATACGAAATTCTTTCACCAATTCTTCTGATATTTTGCACGTTTGGCCTATTTTTAAAGCTAGTTAGTACTGATGATTTGGTTGATCGAAGTGAAGCAATAGATATATTTACATTAACTTTTTCAGCTTGTTATAAAGAATTATTCTTCCTTAAACATTACAAAGAACTAAATAATTTGGTTATAATGGCAGACGCCATGGACTTACCTGATGGTTGGATGAAAAATGTACCGTTTTTAGCCACTCTTCATTGTATTTGTGGTGCTATATCCATTGCTTTTTGGTGGTTTTGCCCAATATTTAAGGTAATATTTGGAGAAACACCTTTATCCGAGATGCGTTTTCCATTGAACATTTATTATTGGGAAGTTGATGGCTGGCATTTTGGTATCGTTTTTTTCTTATGTTGTTTTGGCATTCAGTATGCCGCACAAACGTATATGGCAGTAGATGGATTTGTATTTGTTACTGTATACACAGCTAATGGTGCTTTAAAACAGTTGGAGCAAAAATTGATTAATTTACGAACAATTCAAACAAAAGGAAAGGAATCATTATATGAAGCAAGATTAAAAGAATTGAAAGAATGTGTGAAGACTCATGTCTTTATTTTGCAATTTCTAAAGAAAATGGATATGGTCTTCAGGTCCATGATAGTGGCTGATGTACTGCACGCTATAATTTCTTTAAGTTTTGCTATGTTACAGATGAGCGAATCGAAAGGAATATTTGAATGGTGTAAGATGATTGTTTTTGTGGCCTACTGTTTTTTACATCAGTATTTGAATAGCTTTTTTGGGCAAGAACTAATTGACAAGCAAGAATCTCTTAGGGATGTACTAGAAGAAATTCCTTGGGAGGAGAGCGCCCGTCCATTGGGAATGACAACCCTAATCATGATGACAGTTACCACAGATCCAATTAAGCTTAGCGCTTGGAGGATGTATTTTTTGCAACATGCCACTTTTGCACACTTTGTGAAGAACATGATATCAACATACATGGTTTTGCGTCAAGTCATTGACAATTCTAATTGA

>OR103

ATGAACCGATTCATAAATTACTTTGCTAACGATGACAAGAAAGTTGGCTTTACCTTGCCCAAAGCGGTTTTCAATGCAATAGGTTTAAGCTGGTGGGGAACACCAACGCTGCTTAAATCTTTGTATAAACCTGTAGCATTCATAATACTAGGCTTCATAACCTACGGTGTAACTAATTATTTGTCAGAAAAAGAAATTGTTGACCAAAAAACATTCATAGAAGTAACTGTGAACATTATACAAATACTGTCTATGCTATTTGTGGCCACTAAAATATTTCTATTTCATTGTTATTCGTCTGACATTTCTGATGCTATTAATACTAGTGAAATTCTACAATTAAAATACGGCGTTACATGCAGTACACCAACTGGTTCTAAGGTTATCAAAATATATGCAACAAACATAGCTATCGCTGTTACTGGATGGTTGGTGCTTGGTACTTACAAACATGGTCATCCCCTTCATGTTTCTTTTCCGTTTGATATTTCAACCAAGCATCCAATAAGATTTTTAATAGTTTGGTTTCTTGAAGTAATGTGTGGTTTCTACGTATCAGCTGGAAATGGCGCCGCAGATACCATTCCACTATATACTGTCAATGTCATAGTAAATTTTGGTGAAAAACTTAAAGATTTATTAAGGAAAATTGGTCAGAATCCCGATGAAGATGTAAAATTATTAGAAGAAGCTTTGGACTTGCACTTAAGAATATTAGAGCTGAAAGATAAACTAATCAAAGGTTTTGCCATGCTTGTACTGGTTCAGATAGTATTTACAATATCTCACACTTGTCTTATACTTTTCGCAGCTACTAAGGTGGACATCGCATTGTTCATGTCATTACTGATGCCCTTAACATTTGCTGACTATTTGGAATTGTTAGTTTACTGTTTCACTGGAGAAATGTTATCATCCAAGATGGAAGAATTAAGATTTGCTGCGTACGACAATCAGTGGTACAATTCAAGTATAGCAACCAGAAAGAATTTAATACGTATAATGGAAGCATTAAAGCGTCCAATAAGTTTGAACGGTTATGGCATTGTTAGTGCATGTTACGAGACATACTTAAATTCTATAAAAGAATCATTCTCATATTATATTGTTGTGAAAACGTTGGCTTCTGATTTTAAATAA

>OR104FIX

ATGGAGCATTACTTCAAAATCGTATTCTGGTTCAGTAGAAGAGTCGGCGGAGATAAAAAGTTGGAAGCTTTCACATTGCAGAGAGCTTTATTCAAGTTATTGGGTTTCTATTGGTGGGACCGTGGGTCGAGTGTGCCACTGCCTGATTTATTTAAAATATGGATCTATCCAGTCCTTTATCCAATATTGCTATTAGTTGTGAGCTATTTGACGATAAAAGCTGTCACTCAAATGGACATCATCGATGACTACGATTATGTTAGAAAGGTCAACGGATTCGTTGTTAGCATGACGTTTATTGCATCAGCTTTAAAATGTTTCTATTTGCGATTTATTGGCGAAGATATAAGATATTTAATTCAAATGACTGAAGATATAGGTGAAGTTAACACCACAAACGAACCATTAGCTGGCGCTTTAAGGACATGTGTAAATTATTTAAGTCTCTTATTTTGCAACGCTGGAACTTGGACATTATATTATCTAATTACATATCAAGATGTCCCATTTCCAACAGATTATCCATGGAATCAGCAATATACACTTGGCTGGTTTATAGCATTCTGTTTAACATTTCTGTGCGCACTGTTTTGTGCTACGATACATACATTGGTGGACACGATATATCCAATGAGCGTTGAGGTAATAACTATTCATATTGAAAGAATCAGGATCCATCTTTCTTTCATTGGTTCTGGTAAATGGTCAGACCAGACTAGATTATATCAAGCAGTAGAAACACACGTAGTTTTACTCAGAGTTCACAAACAAATGAAGAAGTGTTTTGGAATGCTATTTGTTGCGCAAGCAGTCTATACAGTTCTACACGCATGTGTGCTGATTTTTGCTGTGGTTAACACGAATGATGTAATTGCACCATTGATGAGTGCGTTACCCATGCTTTCAGCAGCTTATGTACAATTATTTATGTATTGCTACTATGGACAGAAATTAAACTCTCAGTTTAATTCATTGAGTTTTGCTATGTACGATAATGCTTGGTACAATTGTAATCCCTCAACAAGGAAAGTGTTATTACTTTTTGGAGAAGCATGCAAAAAGCAAGCTTACTTAGAAGGATTTGGTACTGTAAGAGCTGCGTTACCTACATATTTACGAAGTCTTCAGGAATCCATTTCTTACTTTCTCGTACTGAAAACCGTGACATCTGAAGCGGAGCGATAA

>OR105

ATGTTAAAACATTTCACTTGTTTTGGGATTGTTGAGGGAGTTTCACTCCCCTTTTTTAGAAACGTTCCCCTATACATGTATATATATTGTATAATTCAAGCAACTTACCATCAGTTTCAATACCATGCCGTCAACTTAAATATTATTGTGCTGGATATATTACAGCGACAGAATTATTTTCAGAATTATGTTAAAATAGCTGTGGGAATAAATTACCTAATTTTGGCGAGCTCTGGATTAAATAAATATTATGTATTAACAATAAAGTACGCTTTATTTCAAAAACTAGGAGAATGGACGATTATTACCAAGAACAAGCACAGGAATAAAAATATTTATAAGCACCTGGCAGAAAAAGTTATTACTATCCTAAATCCATCATTGTATTTTTTCACTAGTTTTGCCTTATTCATCTGGATTTCTTTACCATTTATCAGAGGCCCAGAAACGGCTGTGCTTTTAAAATTGCCTAATTCGTGGATCCCCTTTTTAACACTAGTAGAGTGTATGTGCATGGTGTTCCTCGCTACAATAATTTTATACGGACTTTTCTGTCAGGCCCATGCTCTTGGTTTATTGTGCACTGAGGTCGCCTACATATCGCAAAGTTTGAGACAATTAAAATATGAAAGCAAACATCCTTCAAAGTTTTATGAAAATTTGGTAAATTGTCTCCAAGAGCATACAAAAGCTTTAGGAATTTTTCGTGACATTTCAATAATCTATTCAGAAATGTTTGGATTTCAAGTTTTCATATCAATAATCTCAACGTGCATACTTATTTTTGGAATTCTTAAGAAAGAAGTTATGGCTAATGCTATTATCTATATTTTACCCAATACTTTAGCAACAACGTTTGAGTTTTATTTCTTATGCTGGCTTAGTGAACAGGCTACTCAAGAGCTTCAAAATATTCATTGGGCTGCATATGATAACAACTGGTATGATGCACCAGTGGCTATACGTCGTCCTCTAGGTATAATACTGGAAATCTCTAAAACTCCCTTATATTTAAAAGGAGCTAATGTTATTAATGCTTCACTCAATACATTCGTCGAGGCAATGAAACAAGGTTTTTCACTTTATACTGCACTGAAAGTAGTCACCAGTGATGAATAA

>OR106

ATGTCTAATAATATCGTCAAAAAAGTTATTAGTTTAAAAAAGAAGGAAGCAGAAAGTAAGAAAAGAAATGAGAATGAGCAAGCGGTCATATCTGTAAATAATGCGTTTAAGGCAAGTTACGGTTATCTGTTGACTTGGGGTGGCTTGTTCAGAGGCAGCATGTTATATTCAACATTTTATGTACTCTGGACGGGAATGCACATTGGATTTCTTGTTTATACTGTCTGCATAAGCAAGAATGATTTGGGTATCTTGTTTGAGGCAGCTCATTTTGTGGTGTTGCTCACTAACGCATGTCTTGGCGTTGTGAACCATCGGTACCACAAGGATAAGATCGAGTTTATATTAAAATGCATTGGTGATAATTTTTTTGATTACGGGCACACAATAGATGATGAATTGTCAGAAGAAATAGAACAAATAAGAAAATATAGAAAAGAAATTAAAAACTTTCTCGCTACAGGTTTTATAATTGCTATACTTTTTGTTGGTTCATTTTTAACTGTAGCACGCCCATTATATGATATAATGGTTGGTACCTACTTTGAAAGTGACAGAAGTGATGGAATAGTAAGAACTTTACCTGTGGCATTTTGGTTACCAATTCAATCTGAAGGAATCTGGGTTAATATAGTGGCAGCTTTTGATGAATATTACATTATTATAGGTACAGCGGGCATTGTGCTTGGCGTGGACATCAGTTATTTGTGTTTTTCACACGAATTATGCACCGAATTGGAGATTTTAGGTTTGACATTAAAAAAATTTGATCAACGAGCTTCATTCTTATACAAATTTCGGTATGGCACCAAATTGCCTAAAAACATGGAAAACGACTACCATTTCAATAAATGTGTTGAGGAATGTTTGAAAGAATCCGTGAAACATCATCATTTGCTTCTTAATTATTTTAACTGTCATTACACCTTGAACTATATAATGTTACTTGCTCTACTATTGGCTGCCACATTTATGCTCTGTTTGTCTGGCTTTATTTTTACCAGTGATGGTGTTTTACTGAGCTCCAAGCTAACCTTCCTGCTGTTTCTCGCATCAGAACTGTGTCACACGTTTATTTTCTGTTGGTATGGAGAACAAATTTACCAGATGAACAATAATTTAGGAGATGAACTGTATCGCAGTAAGTGGACACATTATTCCAAGACAGTAAAAAATTATATCTTGATAATGCAAGCAAGGTGCAATAAACCATTGAGGATGAGTGCTGGTGGATTTATGGAAATTTCTTTGGACACATACAGCAACGTTGTCAGCTCAGCTTACTCATACTTCAGCTTGCTCAGAGCTATTAGAGAATAA

>OR107-FX

ATGGAACAATTATATGCAAAGAATGCAGAGAAGGCTCGCAAGAAAGGTTTTAAAGAAAATTACGGATTCTTACTAATCTTAGCGTGTAATTACAAAGGTCCATTATGGTACACTGGATTCAACATCATCTATCTCTTCACCATGTTGCTTTGTTTAGTGTACACTGTATATATATCATCTGGAGATGTTGGCTTATTTGTTGAGTGTGCACATTTTATTGTAATGGTATCAAACCTCTACTCGACTGCCCTATATTCCTTGTGGAATAAGAAGCATTTCGATAGAATGTTCGAAATCGTGGGAAGTGATTTTTTTGACTACGGAGATACCTACGACAGTATCTTCTTCAACAATCTGGTCAATAAATGGCGTTACACGTCTAAGAGCAGAAAAAGAATAGTATCTAACTTGTATGTAAGAACCATATTTGTGGTTTCTGTTGTGCACTGCATTATCAGACCAATGTTTGATTGGCTTACCGGCAAGTATGAAGGAGTCAAAACTAGCGATGGAATAGTCAGAACTTTACCGATTGCATTTTGGTTTCCTTTTGATCCGGACTCTAATGCACATTTCATTGGTGCCCATCTGTATGAATATTATGGAACTTTCTTAGTTATTATTTGTGTATTGTGTTTGGAACTGCCTCTGATTTCATTTACAGAAGAACTGTGTAGTGAATTTGAGTTATTAGCGAAAGGATTACGCTCATTTTGTCACAGAGCGCAAGCATTGTATAAAATAAGACACGGAAAGGAGCCAAAAGAAAAGGACTCTGAATTTGAAGAATGCATAAGGATCTGTCTGCGGGATTCTGTCAGGCATCATGTGATCTTAATAAGATTTGCGAGGAGTTTGCGTCGAATACAGTATGCTCCATTAGCTACAGGAACGCTAGCCGCCACATTGTTACTATGCTTTTCTGCAGTTCTTTTCACAGATGAGAGTTTTTCAATAAGCGTAAAAGTGAATTTTTTGGCAATGCTCGTTGGAGAATTGGTCTACGCTTTTTTATTCTGTTGGTATGCCCAGAAAGTTCAAGACGCGAGCTGTTTACCGTGTCAGGCACTGTATGACAGTGAGTGGATAGGTCATTACAAAACTGTGAAGCCCTTTATGATCATGATAACCTCTTGCACTTCGAAGCCACTTACACTATCTGCTGCAGGATTTCTCTCTGTGTCGCTGATTACTTTTTCTGATGTTTGCAGAACAGCTTATTCGTACTTTAGCTTGTTGAAAGCTGTAAACGATTAG

>OR108

ATGGCCAAAACTGAACACGGAAGAGAATTTTACATTAGAAAAGCGTTTAAAGAGAACTACGGTGTTCTAATGAGATGGGGCGGAATGTATCTGGGAAGTTGGATAACCCCGACTGTATATCTGCTAAATACAGGCTCTCACGCGTTGTTCTTGATGTACACAGCATATTTAAGTCTAGACGATATTGGTCCAATGGCCGAAGCGTTACACTTTGTGGTGCTGTTGACTACAGCTTGTACATCTGTGTTGAATTCATTATCGCAAAGAAATCGCCTGGATAATATTCTTGAAGCAATGGGCCGAGGATTTTATGATTATGGAAAATCAATGGATGAAGTATCCAAAAGAGAAATAAAGATCCATGAGAATGTGGCAACAAGAAGGAAGCTTTTGTTACCAAGACTGTTCATAACAAGTGTTTTAATATCAGGTTTTGCTATATCTATTAGACCATTAATTGAATATTTAATTGTTGGTAAATTCAAAGAATATCGAGGTGATGGAATAAATAGAACAACTACTGTGGCCGTGTGGGTTCCATTTATTGATCCATCAATTTTATGGCAAAACGTATTAGTTTTTTTTTTACAATATGTCATTATATGGACTACTCCAGGTGTAGTATTTGGAACAGATATCATGTTTTTATGTATGGCAGAAGATTTCAGTCTACAATTACGTATCATTGGGATTGCGTTTAAACGTTCTGGACAGAGAGCAATGGATTTGTACAAGAATTTAATTGAGGAAAAACGTTATCTTGGCCAAATATACTCTAAACAAGAACTCTTTAAGAAATGTTTAGAACACTGTATCGCCCAAACTGTTGAACATCATTCATTGATTATCGATTTATTCTACCACTTTAGACTTTATAATCGAGTTATGCTACTGTGGTTGTTGGTCGGTGCTACATTTTTGTTGTGCTTAAGTGGCATTTTATTTGTGTCAGATAAAGTGTCTTTACTCTCGAAAGTGACATTTTTCTGTTTTTTGGCTACTGAACTGGTACACACTTTCATTTTCTGTTGGTATGGAGAACAACTACAAAGACTTAGTATGGATATTGGAAAAATGTTCTACAACAGTGATTGGTTTCAGTACAGTTCCTTAGTGAAACAAGATTTACTGCTTGTACAGAAAAGGACCAATGCCCCTTTAAAACTTACAGCAGCAGGCTTTATGGATGTAAATCTCAATTCCTATTCAAATGTGCTGAGCAGTGCTTATTCATATTTCAACCTTTTAAGTGCAGTTAAGCACTGA

>OR109NTE

AAGGCCAGAGGGGTGAACGATGACCTAAGAAACTGTAAATTCTTTTGGGATAACTACGGACAGTTTCAGGGAGAAGCAGACTGGTCTATCGGAGCAAGATCCGGTTTGCAGAAAGGGAATCCTGTGGCTGTTAGAGGAAGGATTTTAGACCGTGGACTCCATGACTATGAAGATACAATTGATGAACACTCTTTTGCTGTGATTGAAGACTTAAAACAAAAAAAAGACCGCAGGAAAAACGTGTTAACCCGATCTGTCTGCATTCTAATAGCTATTGCTAACATAGCTATGGCAGTGGTTAGGCCAGTGATTAACTATTTAAGCGGTAAATACGAAAATCCAAAAAATGATGATGGTATAAATAGGTGGTTACCGATTCCTCATTGGTATCCATTCGACTGTAAACCTTGGCCTGCTCGACTGTTCGCCTCGACAATGAGCTACTATGTGATGTTCTCCACCTCTCTAATCGTCCTTACAAACACATTGATATTTCTAATTGTTGCTGAAGAAATAATTTGCGAGCTCAAAGTTGTAGCAGTATTGCTGACAAATATTTCTGCGCGAGCTGACATGCTCATGAAACGTACTAAATTTCACAGGGATGAAAAGAAAGGCAAAATTCAGTTTCAATATCTAAGACTGTGTCTTACACATTGTATTAAACATCATGAAGCAATATCAGAGGTATTCAACCTGTTCGACTACATGTACACTTTTATCCTCATGTTCACATTCTTGACCAGTACCTTCGTGATCTGTCTATGTGCAATACTACTAGCAGAGGGTGATTTGGAACCGAGTTCTAAATTTACATTCATATTCTTATTGTTGAACGAAGTACTTCATATTTACATATTCAGCTGGTATGGACAGAAGCTCCAGGATTTGAGCGAAGGTCTCAGGGACACTTTGTACTTCACATTATGTTATGATGACATGATGGCTATAAAACACCATTTGTTGATGCTGCAAGCACGATGCCTGAAGCCGTTAAAGTTGTCTCTTGGTGGATTTGCACATGCCTCTATGGATACATTCGGAACTGTATTAAATTCCTCATATTCATACTTCAACGTAATACAAGCTGCTAAATAA

>OR110-FX

ATGAGAAAAGGATTTCTGGATAATTATGGTTTTCTGATGTCCATGGCAGGAATTTATCTTACCTTTCCGGGCAATTGGATCATCTCAATGTTGCATTTAGGGGTGGGCAGTTTACAACTTATCTGTTACTTTTACACTATTTATTTGACTAATGATATGGGCAACGTTATCGAGTTATTGCATTCTGCTGTAATAGTAATGACAATCACTGGACCGGCTATAATCAATGGTCTTTTGCAAAGAAAACGTCTTGAAAGAATCATGAGAGCCCTAGGTACAGGGGTATACGATTATGGAGTAACATTGGATGATGACTCGCTGAAAGAGATTCGAGAATTCTCGATTCAAACAAGGGCTCGAAAGAATCTTTTCTGCAGGATATTTTTAGCCATAGTGGTGGCCAATCTGTTTGCCATAGGTCTTATCAAACCAATTGCAATGAATCTTTTAACAACGATCGAATATGACGATGATTTTGATGGAATTAATAAACGAGTCCCGGTGCACTGTTGGTATCCTTTGGACAGCCGGGTGCCATTGAACAACTTACTATGTTTCATTTTTCAATACGTAATCGTTGTCACTATACCATTAGGGGTTTGCGGCCTTGATTTAATATATTTGTGTATGGCTGAAGAAATAGTTATACAGTTGAAAATTGTTGGCACTTCAGTGAAAAATATTAAGAAAAGAGCGATGAAAGTTATGAAACTAAATGGAACAAATGATATAAATAAAGCTATGTCACGATGTCTAGGACACTCTATTGATCATCATAATGCACTATCTAGTTTATTTGAAGACTTCAGGAATTACCATTTCTATATCCTTCTATCCGTGATGACTGGCGCCACGTTAATGCTCTGTCTGGCTGGAGTTATATTTACTACAGATAGTGTTGCTCCTGTGTTTAGATTGACTTACGTCGTGTTTATAAACACTGAACTGGTGCATGTTCTGGTGTTCTGTTGGTATGGCGAACAAATCAAAGAAATGAGTATAAAAATTGGCGATGATATGTACGATGCTGATTGGTTCAGCGAGAGTGGCCACATTAAAATACACATGCTGATGATCATCCAAAAAATTGTGAGGCCGTTTAAAGTTACAGCCGGCGGCTTTATGGATGCATCCATGGACACATATGCTAATGTGCTTAGCAGTGCTTATTCTTATTTCAATTTGGTGAAAGCTGCACGTGAAAATTAG

>OR111-FX

ATGAAGAAAGGATTCTTGGATAATAATGGATATTTCATGGCTTTAGGTGGACAGTATCTTTGTTTCCCTGGCAACTGGATCCTGCCATTTTTGCATATAACAACCGGTTCAATACATATAGTACTTCTAGCTTGCACAGCTTACATTGTAAACATACAAGATTTGGCAATAATGTGCGAAAGTTTACACTTTGTTGTAGTTCTCGCTACCACGTGTACAATGGAAATAAGCTCTTTGATGTTAAGATCCAAAATAGAGTACGTTATGAGAACATTGGGTGAAGGTGTATACAGTTATGAAGATTCTTTGGATGAAGCTTCCCTAAAAGAAATGCGACAGTTATCAAGTAAAGCTAAATATGGAAAAAAATTAGGCACAAGAATATTTGTCATTTTGGTAATGTTGACTTCGGTGGTCATAAGTTTTGTTCGACCAATAATAATGCATCTAATACAGAAATTGGATTATAGTGATGATTTTAAAGGATTAAACAGGTACCTTCCTGTACATACCTGGTTTCCCTTCGATACACACGATATTTGGGTCAGAATACTTTGCGGTGTGAATCAGTTCATAACTATATACACAGTACCTTGTACAGTATTCGGATTTGATTTAACATTTCTTTGTATATCCGAAGAGATTATATATCAATTGAAAACTGTTGGCTTAACTATGAGAAATGTAAAAGTGCGAGCCAAAACGCTGATGAAGTCACGTGCAGATATGGATTTCGGTAGTGCACTTACAAAATGTTTAAACCATTCAGTTGAACACCACAATGTCGTAGCGAGGTTATTCAAAGTATTCAATGACGTATACTATTACTCTCTATTAATGATGATGACTGGTGCAACATTTATGCTCTGCTTATCATGCATACTTTTCACTGCGCCTGACGTTGGATTTTCATCAAAAGGGACGTTTCTATGTTTCTTGTTAGCAGAACTTTTTCATACATTCTTGTTCTGCTGGTATGGAGACCAAATGAAAGAAGCGAGTTCAAACATTCTTAATGAAATCTACGGCAGTGATTGGATTGAATACAGCGTAGAAATGAAGTTATATATCATAATGGTGATGCACAGGTCTCTGAAACCCCTTACTGTAGATGCTGGAGGATTTATGGACGCTTCCATGCATACATTTGCTAATGTACTTAGCAGTGCTTACTCTTATTTCAATCTGCTGAATGCTGCCAGGTAG

>OR112

ATGCAGGGCTGTCGAAGAGTTGAGCGAGAATATATGCGAGAAGAGGAGAGGAAAAGACAATTTGAAAGAAGCGATCTGATAGGACTTAAGAATGGAATGACAGTTAAATGCTCAGAGAAATCTCAATGGAAAGCGATGAACGAGAATTACGGTTACAGCTTCTACTATGCAAACAGTTATTTAGCGCCCTGGTTAAGAAGATCACCATTGCATTATGGTGCATGGTTGATCATGTCAATGTACAACACTTTCAGCGTCTCTTCATGGATGTTCATACTTTGCAAATCGGTTATATTGAGGCCTAGAAATTTTCAAATACTTACTGAATGTACGCATATGATCATACTACTAATTTTATTATTATCTGGCGTATTTAACACAAAGTACCGGAAAGCGGAATTGGAAAGAATCTTGAGAGCTGTAGGAGAGGACATTTACAAATATGGTGACACACTGGACGAGGAAACCGCAGAAGTATTGAAGCAAATCAAAGAGAAAACTTTCCAACAGAAGCAAAGTTTATCTAAGGTTGTGAATATTATTATAATATCGGTCACCGTAGCGCACACTACGCTCAGACCGCTGGCCGATCTGTTTACAAGCGACAGAATCTATCAGCCAGACGAACCGGACGGAATCACCCACGTTCTGTCCATGGATGTCTACTATCCCATGAAATTGTATAACAGTGTTCCCGCCATAATCCTCGCTTATACACATCTTTTACTGATATTCTTTTGGGGCGGAAATGTGGTCGTTTCCGCGGCAGTCACCTTCATCTGTTATTCTCATACACTCTGCGGAGAGTACCGGATATTATGCGTAACGACTAGAAGATTAGTGGAGCGAGCAAAGGCAATGTTCCATAGGGAAAACCCTGACAAACCATTTATATCCTTTAAAAATGATATCATCTTTGAAAGATGTCTTAGACGTGCTCTCAAAGATTCTGTGAAACATCAACAGAAATTAACGCAATTTGCGGACGATATTAAAGTACTCTATTATTACGTCATTCTAACAGGGCTCTTGTGTATAACAGCTCTCTTATGTTTTGGTGGAATTGTGTTCACAGTGAAAGATCCACCAATTGCTACACTGGCGTTATACATCATGTTCTTGTCAGGAGAACTGTACTTGCTACTCTTGTTCTGTGTATACGGAGGAATGATCAGAGATTTGAGTATGCAGGTTGGGCAGAAAGTGTATTTCAGTGATTGGACAGAATATGTTTCAATGATCAAAACGGACTTGTTGATAATGCAGACTTCAAGTCAAATGCCACGAGGCATTTCAGCTGGTGGATTTCATTACATCGAATTCTCCACGTTTTCAGATGTGACAAGTTCAGCTTTTTCTTACTTCAATATCGTGAATGCAGTACGGAAAGATTGA

>OR113

ATGAATCGACTCAACAATGATCTTCTAAAAGGATATTTGAACTATTTCGCCACTTTTGCTTTCTATATTGAAGACAAGCGTTCAGCTAAATTATATAGCATTTATTTTACCATATACAATGTTCTCACTTTGGCATTAATTACAAATTCTTTCCTCAGCTTAATGCCGGATATAGATAAAATGGCCGTATTATTCCACCATTTCACCGTAGAGTTCGATATGACCGCACATGTAATTATCTGCTACTACTGTAGACCCAACATTCACAGATTAGTAGAAGGACTAAACGTTACCCATGATTATCAAAGTCAATTAATTCAAAAATTTGTTACCCAAGTTGAAGTAGAGCGTAATGAAAAAGCTAACCAAATTTTAAAAGGAAGTTATATCGCATGCTTTTTCACATGCGTAACTTTTGTTCTTGCTGGCGTTTTTGACAGATACATACTACATAAAACTAAATATTTCTTATTGGTCCCAATGGTTTTCCCTCTATCGTTGGATCAATGGACCGGTTACATCATATTACTGATTTGGCAATTTGTTGCCCTTGCTTTGGCAGTCGGCATGCTATTTTGCCTATTTGACTTTTTATATTTATTTTATATAAAAATTATAACTGAATTAAAAATTCTGGATTATGCACTGAGCACGATCAAAGAAAGGGCTGCAGAAATTAGGGAATCAAATGAAAAGAAATATAATAAAACATTAGATCACAAGATGGGCGACTATTATGAGGAATCTATAAAACACTGCGTCATCCACCATATTCAGATTATAAGTACCTTTAACGATATGAGGACCACCGAAAGTTGGATGTACTTCATTGGAAACGCCGTAATTCTAGGACTGTTGGTCTGTACAGGTGTTACTCTACTCACCGATGATTTATTGCTGAAAATTAAATTTGTTCTAATAATGGGCATTACAATGGTTTACGTATTCTTTTTCTTCTGGATGGGACAGGATATGTCCGATGCGAGTGAGGATATTCTATTGACATTACACAACGCCCACTGGATAGATATGCCAAAGAAATGTCGTACAGCTTTACTAATAATAATGGCCAGAACAATGAAGCCATTGATTATTTCTTCTGTTGATGGTGTCGATATAACTATGGAGAATTTCATGGCGCTGCTAAAGGCTGCATATTCATGCTTCAACTTGTTGATGCAAATGAAGGAATAA

>OR114

ATGCAGGAAACCAGCAGACAGAAAAGTGTTATGAAAGATTATTTAAGAATTTTCGATATTTGCGCTTTCTACGTTCAAAATGATCGATGGGCCAAAATGTTCTGCATTTATTTAACCATCTACCACGTGTTTACTTTTATATTAATGATAAATTCCTTTATCATTTTTATACCAGACACAGATAAAATGGCAGTTCTATTTCACCATTTTACCGTACTAGTAGATGTGGCCGCACATATCTTAATCACTTATAGTTACAGACCGACAATTAAAAGATTGATAGAAGGAATTTACGAGACACATGATTATGAAAGCGAATTAATAGATGAATTTGCGGCTAAAGTTCAAGATGAACGTCTGGAAAAATCTAAAAAAGTTTTAAAAGGGAATAACTTCGCCCGTTTTTTGACATGCATACTGCTCTTTCTGACAGGTGTCTCTGACAGATATGTATTAAAGAAGACAGAACGTTTTTTACTAATCCCAATGGTGTTCTTCGTATCAATAGACACTTGGACAGGTTACAGCCTGTTACTGATTTGGCAATTTTTCGGCATGATTTATGCTGTATGCATTCTATTTTGTATATATGACTTTTTATATTTATTTTATATAAAAATTATAACAGAATTTAAAATTCTGGATTATGCACTGAGTACAATCAAAGAAAGGGCTGCAGAAATTAAGGAATTAAATGAAAAGAAATATAATAAAACATTAGATCACAAGATGGGCGACTATTATGAGGAATCTATAAAACACTGCGTCATCCACCATATTCAGATTATAAGTACCTTTCAGAGCATAAGAGACGCAGAAAAATGGATATACTTCTTTGGTTATGCCGTCAATTTAGGGCTATTAGTTTGCACTGGGTTTACTTTGGTCACCGATGACGTCATGTTGAAAGTTAAATTTCTCGTAATAATGGGCATTAACATGGTTTATGCATACTTCTTCTTTTGGATGGGACAGAAAATGTCTGATGCGAGTGAGAATATCTTATTGACATTACACACTGCGGATTGGCTTGATATGCCCAAGAAATGTCGCTCAACGTTACATCTAATTATGACTAGAACTATGAAGCCATTAATTGTGACGTCAGTCGAAGGGACCAAAATAACTACGGAAAATTTCATGGCGCTCGTATCTGCTGCCTACTCGTATTTCAATACAGTAATACAAATGAAGGGATAA

>OR115FIX

ATGCAGGAAACCAGCAGACAGAAAAGTGTTATGAAAGATTATTTAAGAATTTTCGATATTTGCGCTTTCTACGTTCAAAATGATCGATGGGCCAAAATGTTCTGCATTTATTTAACCATCTACCACGTGTTTACATTAATATTAATGATAAATTCCTTTATCATTTTTATACCAGACATAGATAAAATGGCAGTTCTATTTCACCATTTTACCGTCGTAGGAGATATGGCCGCCCATGTCTTAATCTCCTATTATTACAGACCGACAATTAAAAGATTGATAGAAGGAATTTACGAGACACATGATTATGAAAGCGAATTAATAGATGAATTTGCGGCTAAAGTTCAAGATGAACGTCTGGAAAAATCTAAAGAAGTTTTAAAGGGGAATTATTTCGCTTGTTTTTTGACATGCATAATGATCTTTCTGGCAGGTGTCTTTGACAGATATGTATTAAAGAAGACAGAACGTTTCTTACTAATCCCAATGGTGTTCTTCGTATCAATAGACACTTGGACAGGTTACAGCCTGTTACTGATTTGGCAATTTTTCGGCATGACTTATGCTGTATGCATGCTATTTTGTCTATTTGACTTTTTACATTTATTTTATATAAAAATTGTAACAGAATTTAAAATTCTGGATTATGCACTGAGTACGGTCAAAGAAAGGGCTGCAGAAATTAGGGAATCAAATGAAAAAAAATATAATAAAACATTAGATCACAAGATGGGCGACTATTATGAGGAATCTATAAAACACTGCGTCATCCACCATATTCAGATTATAAGTATCTTTCATAGCGTAAGAGACACAGAAAAATGGATGTACTTCATTGGAATAGCCGTCAATTTAGGGCTATTAGTTTGCACAGGGCTTACTTTGGTCACCGATGACGTCATGTTGAAAGTTAAATTTCTCGTAATAATGGGCATTACGATGGTTTACGTATTCTTTTTCTTCTGGATGGGACAGGATATATCTGATGCGAGTGAGAATATCTTATTGACTTTACACACTGCGGATTGGCTTGATATGCCCAAGAGATGTCGCTCAACATTACATCTAATTATGACCAGAACTATGAAGCCATTAATTGTAACGTCAATCGATGGGACCAAAATAACTACGGAAAATTTCATGGCGCTCGTATCTGCTGCCTATTCCTACTTCAATACGATAATACAGATGAAGGGATAA

>GR1-FX

ATGAAGATTCTACTAAAAATACTAGGTCTAGAACCGTTTTATTTCATCAATGACAAAAGCAGGTTTTCACCATTCCTTTCCATATGGAGTATTACTCTGGTCAGCACTTGTATGGCATTATGCGCTTGGATCTTAGGAACGGACGTAGCATTTACAGAAAGGAAATCTACAAAAATGAAAACAACTATCAGCACGGTTGTAAACATTTGCGATGTTGGAACAAGTGCTGTAGCAAGTATTGCTTGTGTGGTAATGGCAGCCGTTCATGCACACTTAATTGCCAGAGTACACTACAAATTGACATTAACAAATACAAAATTGAACATTATACCACCTTTAGTACTACATGGAAATAATCTCCCTCATTTAATTGCACCTTGGTTGTCTTTAATAATCATCATTTTGTATACTTCTTTTCTAATTACTGACTTTATTTTAACAATTGTGATGGAAAACAACAGTTATGTTTATACTTTGATGTATGTATTCCATTATGTAAATTTTACAGTTGAACTTCAAATAGCTTTAATTGCCAGGAAGATAAAATATTTGTTCAATGCTTTGAACGTTACAATATCCAAATGTTTAGCTAGAGCTATACTACGACCATTTGTATTACCGCCGGTGGAAACTTATCAAGGTGGAACGGGGACTCGAGAGATATGTGAGTTGACAGACCTCAGTGAACTTCATTGGAATTTGTGCACTGCTGTACAAGATATAAATAACGCATTTGGAGTTCAGTTGCTGACTTTATTGCTGTCTATTTTTGCTCACGTCATAATAACACCCTATTTCTTTTTTCTGGCTTTAGTCGATCCAGACACTTACAGTGGAAATGGTCCGTACTTATATTTAAAATTAGGGTGGATGCTTCTTTATTTTGGGCAGCTATGTATGGTAGTCGCACCGTGTTCAGCCGCAACAGACAAGGCAGGAGAAACCGCAACAGTATTGTGCAAATATTTGAATACAGACTTAACTCATGATGTCGAAAAACAGGCTGAAACGTTTCTATTGCAGTTACTCCATCATGATGCAACATTTTCAGCTTGTGGGTTGTTCAACATTAATTTGCCAATAATCACATCGGTAGCCGGTGCAGTGTCAACTTATTTAGTTATTTTGATTCAATTCCACAAATCTGATTCAGCTGCTCCTGATTAA

>GR2-FX

ATGAAATTTAAAGGTGATTATATGAGAAGTGGAAAAAAGAATGTAGATAAAGATCCTTGGGTTGAATTAGGTCCCATCGTTTGGATATCACGGCTTGTTGGACAGGGTCCAGGAGGAACTATTATTAACATCTACATTACGATAATGGCTATAACTGAAGCAACTATATCATTTACTTGGATTTTGTTTGTTGGTCACCATTCGAAGAACACATTGAGTCGAGTAACTGAAGATGGTCAAGTGTTGATATTGTATCTGTCAGCCAGCTGGTCAGCTTTGAATGCTGTTTTATGGCGTGAAGATGATAAAATTATGACCCTACTGAAAGGTTTTCCTTTGGAAGGAAAATATAATCCATGGAGTTCTTATAAAAAAATTGCATTAAAATTAATTTTATTAGCTCCTGTTATGGTATCCATTTGGCTTCTTTCCAGAGAGCTCGCTGCTGAATCGAAATGGACATTTACCGCCTACTACCTACCGCTAGTACAAATGATTTTCGCCCAACTTCAGAAGACAACAGTAGTTGATATATTGACCAAAAGAATGGTTCAATTAACCCAACACCTTTCCAAATGTGTTGGAATTGTAGACAGTAAACTAATTAAAACTAACTCCCGGCATTGTTATGCAATGGAGCAAACATTTTCAAACGACATTTTAATGGAAGTACGCTGTAAAACTCCTCCAAATTTATACAAAACCAATGGGACAAATTTTGATCGAGAGATGTTAGTAAATCTAATTTCAGGATATGCTCTGCTATTGAAAGCTCGAACACTGGTTAACTCTTCATTTGGTCTCCAAACTTTAGTTTCATTGGGAAGAACATTTCTTATTGTAACTTTTAATTTTTTTCTGTTCATTAAAAGAGAATTAATTAAATACATCCAGACTGGATTGAATTTGGATTTCATTACACATTCATTTTATTTACTAGCAACGATTTCAATACATCTAATTGAACTATGGAGTCTAGTTTCTTCAGCAGATAACTTCTCTAAAAGTGCAGAAACTTTTACCAGAAACCTTTTTACCGCTGCAATTGAAGATGATACCGAAATTTTAGCCGAAATGGATGTAGTGAAAAGTCACATTGCATCGTCAAAAGAAAGCACAATAACTGCATTTGGCTTTTTATCAGTGGATTATTCGCTCATTTGTGGCGTAAGTATAAAGTTATAG

>GR3

ATGCTTTTACATAGTCTGTATGATTCATTTAAGCCATTATTTTATCTTTCAGTTATATTTGGAACTATGCCTTTATATTTTTATTATAAACCTGTAACACATTTGAAACTTTCAAAGTATAAACTGATGTATTCTTGGTTTCTTTTTGCGTGTGTAATTGTTCATGGTGTAGCTACTCCTTGGGTTGTTGTAGAAACAAAAATCTCCGCTGTTAATCCGGTAGTCGTACCAAAGGCTGTTTCAAATGTGAGCACTTATGTTAATTTTATTAGAAATCAAACTTCTGCACAGATAATTGCAATGAGAGTTATAAATCCTTTGATAAATATATTTATGTGCTTAGGTTCTTTGTGGGTCGGCTTGATATATGTAACTGATAAACTTCCAGTATTTATTTCACATTTAACTGACATTGATAGAGGATTAGTTCCATTTATGAAATTGAGACCAAAATTGAATCTATTTATATCATTAACGCTTACAATGTATTTTAGTATATTGAGCGTTCCAGTGTTTACTATTTATCTTTGTACAGTTTATTCGCAAGGAAAATTTTGGCCACAAGTTTGGAGTACATTATTACTTTTTTGTGATGCTTCAAATTATTCGGTTGAAATTCAGTTTTTGAATTGGGCCCTCTGTCTTCATATTAGATTCAAAGCTATCAATGATTTTCTTCAGAGGCATTCCACACAGAATGAAATGTTTAATGCATTTAATCATATAAGCCACAAAAAATGGAGTGTAAAAGAACGTTTAACCATTCGAAAAATAGAACTAGCTCATGAATGTAATAAAAAGTTATGTGATCTTATAGAAGATTTAAATGACATGTATCAATTACAACTATTGTGCTCTGTTGCCGGTTGTTTTATTAAAATACTTTTGAACGTTTATTTTGCCTTATTTGCTTCTTTTATTAATGTAAAGAAAACTTCTGGAGTTCAAACTGCTAATACAATCATGTGGACAACATTTTATTTGGTCAGATTTCTTGTTACTACGGTTGTCTCTTGTAAAACAAGCCAGGAAGCAACTAAAACGAGATCACTTGTGACTTCAGTAAACAGAAGATCTTTTGATAATGTCATCAAAGAAGAGTTAAATTTGTTTCTGCAAAACACGTCCAATCGTAGTATGAGATTTTCAGCTTGCGGCCTATTTACTCTAAGCACACAACTCATAACATCTGCATTAGCAGCAGGAACTACGTACCTTGTAATACTTATTCAATTTCGACCACAAATCAGCTAA

>GR4

ATGGAAGAAGAAGAAATAGTGCTTCCGATGCCCAAGTCCTACCGTTGGGTTAGGATGACCATAACGGTATCTCAAGTGGCCGGCGTTAATCAGAGCAAGGGAAACAGATTATACTTACCTTTAGTGGCTTATTCGGCTTTATTGTCCGCGATGAGTGGATTTACGATGCTGTACGACTATGTGCGTCAAGCGGCCGGGGGTCTGTTCCCGGACACCGCTGCTATGGTTTTGTCTTCATTGATAGTTGTGGCCACGCTGTGTAATGTTGGAGTCAGCTGGAGAAATACCGTCTACCACGCACACCTATCGTCAGTGTTTCGTCAGCTGGATGAACTTGCTGAAGATATTTGTCGTTTAGGACTGCAATTGGATTTAGATAGGTACTTTGATCTATCCAGAACGGGACTCTTGTTACTATTCATGATTACAATGTATGTATTGGAAAACATTTTGTACCCACCTGAAGAAACGTTAGTGTTACTCATAAAACTGTTGGCATTCTTGCCACTTGTACAAAAATCGCTCACTGAAAGGCAATTCTTGGGCTTACTTCATGTGATTTCAAGATACTATAGTGCGATAGATACTGGATTGAAAAGTCCAGAAATAGAGAAGACATCACTGGTGAAGATCCACAGATCACTGGCTTCATTATGTCACCAAGTGAACCATATATTCAGCTTACAGATTTTCTTGTCAATAGTATTCATCTTTGTTATTGTAATGGTAAATCTGGAAACCATGTTACCAGTGTTCGTTTATTTATACAGTCTGGATGTCGACAATCAGAATATGTTAATGCTTAAAGTATTTCAGTCACTTTTCTTTATGTTTCAAATATTTGATTTGATGTCGACTTCAGCTAGAGTTGCAGAACAGGCTCGAATGTTTGATTGGACGTTGTATCAAGCAATGTTGGATGATCAACTGACCAATATTAATAAAGACCTATTACTTTCTCACTTATCAATGAAAACAGAAGTCAAGTTTACAGCGTATGGTGTGATTGGTCTGAATTATCAAATGATAACTTCAATACTAGGAACACTAATGACATTACAGTTGATGTTATTGTTAATTAATTTACGTGACTTGGATGGCGCTGATAGTGGTAAAATTAGAGCAAAATTAAATTATATCAAATAA

>GR5

ATGACTGAATACATCACGTACAAACCATATTTAGCCCACTTGTTAAACAAAATTACATTAACTTGGATTTTAAAAGCTAATTTAGACGTATCTCTCAAGTTGTTAGTTTTATTTATATTAAGCACCCTGTGTAAAATATTTACAGCTTGTCATTACTCAACATATTTATTGCGCAATCAAATTCTTGTAAGTTCAGCTTTTTTTACTTTGTATTTTCTGTTGAATTTTATGAAAATTTTATTGAATATTGTCCATATTTATTCACAAATTATGTATAAAGATTTATGGAAGAAATTGCAATTTAAACGTGAATTTGGTGACAAAAGAATTTCCGCATCAACTTGTGCATTATGTATGCATTTATTCATTTATTTATTATTAATCTTAGTGTATATACATTATTTAACTAACATTAAAGATACAATTTACACTAAAGTAATGGCAGCCGTATTTTTATTTACATCTGTAGCAAATATAATGGTATTGTATCAGTTCACTTATGTTATGGACATTATCATTCAACTAATGAAGAAAATAAATAAAGAAATGGAATCAACTGAACAATTTAAATCAGCTGTAAGTGGAAATCTAAAACTTTTAACCGAACAGCATAACCATTTGATGGATTCAGCACAAATTGTAAACAAAATATTTAGTTGGCAAATACTAACAATATGTTTGCGATCATTTATGGAATTAACATGTCATAATTATGGACTATTGGTAAATTTAACTAAAGATTATGACAAAAATATTCAGGATAGAATATGGCATATATTTAGAATATTGCTTAATATCACTGAAATAAGTACTATTGTACATTTTTGTGAATACTTTTCTAAAGAAGCAAAAAAGTTCAATACATATTTGTTCCAATATTTGCGAAATAAGCAAATGGAATTTACCAAAGCCGAGTTTGATCTCCATTACTACTCTCTAATGAAAAGAGAAATAATCTTTACTGCTGCCGGCTTCTTTGATTTGGGTTATCCTCTCATAACTACGATGATTGGCACTACGGTTACATTTTTAGTAATTGTAGTGCAATTTACATTTTAA

>GR6

ATGTACGATTTAAAACTAACATATTTCATATCAAAACTATCAGGCTTATGGTTGTTTAATAAATTTAATGACATCGATTATTGGTATTTAATTGAATCAATTATCGTTAGATTATTAGAAATGTTACATATAATTATGGAAATTTATCGATTAGCAAGTTTATCGAACTTAAATAAAAAAAATTTAATATTGAACATTATATTCATATTGAAATCTGTAGTAAATAATTTAATGAATCTGCTGCATATTTATTCACAATTTCGCCATGAACAATTATGGAAATTATTAATATTTTTTAAAACTAGAAAGACTAAATATTCAGATTTGGTGCTTGTTTATAATCTAGTTTTATTAATTATTATATCTGTAGGTATAATAATTTCTTTAATAAATAATGTTGATCGCTATTTAAATTTAATCATTTTTCAATTCATAATTATCCAAATAGTGAATTTTTTCAACAATTTAAAAATTTATCAATTCAGTATGTTAATGGAAAATCTAGTGATTAATTTAAAAGATTTGAATAAAGAAATAAAAAATTTTAAAAAACAATTATATCTAAAATTGTATTATAAATTTTTAATTAAAAAACATGATGACATCATTAATCAAGCTAGATTAATAAACTTTGTATTCTCGTGGCAGAATCTATTGCTTTGTTTAACTAGTATAACCGGTTTTATAGCTAACCTATACAATTTGTGTATTTCTATTGTGACTGTTAACATTAACATTTCATTATCTGCTGTAACGGTTATACTTTTTATTATTTATTATATTGGAAATATACTAACTGTGGTGTATTGGTGTGAAAATTTTAGAAATCAGGTTGAAATATTCAACGACCAATTGTTCCAATTTGCATCAGAAAGAGCCTTTCGTGGAAACCAAGATAATGATCTACAGTTCTACGCCGCACAGATGAGGAACGTCACTTTTACAGCAGCTGGATACCTTCATCTGGGATATCCACTTATTACTTCTATAACGGGAAGTTCGCTAACATTAGTGGCGATAGTTGTACAGTTCACTTTTTAA

>GR7

ATGTTCGATTTCTCTTTATCATCTTCTCTGCCTAAAATCACAGGTTTATGGTTGTTTAATGATTTCTATGGTATTAATAGAAAATTCTTAATTTACTCCATAATAGTTCGTTTTTTTGAATTTGTTTGCATCATTTTCTCCATTTATCTAATATTTCTATCGATTAAAGACAACGCAAACATTATTTATGCTTTAATATTTCAAATACCCATATTAATTAACTCAGTACTTAATGTAACACATATTTGTTCACAATATTATTACAAAGAATTATGGAAACAATCATTGGTATTCAAAACTGAAAAGACAAAATACTCAAATTATATATTTTTAACAAATTGTGCAGAATTTGGTATTATTTTTATATTAGCTTTATTGTTTAGATTAACTCAAAGGTTAGACCATACCTGGAGAGATAATGCAGTGTACATTGGTTTGTTAATTGTACAAATCACTTTTTCTTTCAAAAGTATTATAGTAAATCATTTCAGTATACTCATGGAGAATTTAATAATTAAACAACAAAAAATTAATAAAGAAATGGAACTATTAAACAATGAATTATATTTAAAATTATTTGTTAATATATTAATCAAAAAACATGATGAAATCATAATCAACGCTAGACTGATTAATACAATTTTCTCTTGGCAGAACCTATGTTTGTGCACAATTTGTACAATTGGTTTTATTTCAAATGTTTATGGTGTTTGTGTTAAGTTATTCCTAAAATTACAAATTAGGGCTCTTCTATTGAATATAACATATTTTGTGCATTACTTTGGTATTATCCAAGCGGTTGCTTATTACTGTGAAAATCTAAGAGGGCAGATTGATAATTTCAATTATTTATTGGTTAGATTTACTAGTGAAAGATCTTTAAGCGGATTTATAGACGTACGTATAATTGACGATCTTCAGTTTTATGCAAGCCGTAATAGAAACGTCACCTTCTCTGCAGCCGGATACTTCACTCTTGGATATCCACTTGTCACCACATTGGTGGGCACTACAATAACTTTGGTGGCAATAATAATACAGTTCACATTTTAA

>GR8

ATGATATTCGATTCATCGATAAAATTCTTCATAACAAAACTACTAGGTTTATGGTTGTTTAATAAAGTTCATGACATTAACTCTGGATATTTAATTAAATCCATAATTGTTAGAATTTTAGAAATTTCGGTAATAATACTTAAAATTTATTATTTATGTGTAACCAATGAGAATAAACGATCAATAATATTGCAAATTATATTCACAATTAATACAGTAACAAATCATTTTATAAACTTGATTCACGTTTATTCACAATATCGTTATAGGAACTTATGGAAATTATTGATATTATATAAAACTAGAAAGACTAAGTTTTCTGATTTGGTACTTATTCTTAATTTTCTATTATTACTTACTGCAACTGTAGGTAGTTCAATCAATTTGAAAATCAACTTCAATAGTAATCATTTGGTTATGGTCATTGTAGATCTTCTGGCTAGTCAAATAATCCATTTTTCCAGCAATTTAACAATGTATCAATTCTGTGTGTTTATGGAAAATTTAATAATCAATCTACAAGAAATGAATAAAGAAATGAAATTGATTAAAAAACAGTTACATTTAAAATTATATGTACAATTATTGATTAGAAAGCATAATGAAATCATTAATCAAGCTAGAATTGTAAATAATGTATTCTCCTTGCATAATCTATTGTTAGGCATATCTAGTACTACTGGTTTCATTGCAAATCTTTACCATGTTTGTATTACTATTGATAGATTTGATGCTGTTGTGTTATTCACACTGATCGTACGTATTCTTTTTATAGTTTACTATCTTGGAAATATACAGGTTGTGGTGTTTTGCTGTGAAAAATTTGGAAATCAGATTGACCTATTCAATGACCGACTGTGCCGACTTACAACTGAAAGAGCTTTTCAAGGAAATCAAGAAGATAATGATCTACAATTTTATGCCTCGCAGAAGAGAAACGTCAAATTCTCAGCAGCTGGATACTTCAGTCTGGGATATCCACTTATTACTACAATAATTGGCAGCACAATAACATTAGTTGTAATTGTTGTGCAATTTACTTTTTAA

>GR9

ATGTTCGATTCAGTAATAACATTTTTCATAACAAAACTAGCAGGCTTATGGTTGTTTAACAAACTTTATGAAATTAATTATTGGTATTTAATGGAATTTATGATTGTTAGATTATTAGAATTATCATTTATTATTTTTGAAATTTATCTTTTATGCGTAACAATTGAAAATGAACGCATCACAATGTTGAAAATTATATTCATATTAGTAGACATATTAGATCATAGTGTAAACCTTCTTCATGTTTATTCACAATTTTCTAAAAGAAATTTATGGAAACTATTAATGCTGTTTAAAGCAAGAAAGACTAAAGTTTCAGACTTTGTGCTTATTTATAATTTCCTATTAGTTATTATTGGAAGTGCAGATGTTTTAGTCGATTTGAGCGATTATAGTAGTAACGATTTAATTTTGTACATTGTAGACAATCACGTGAGTCAAATGATATATTTTTTCAACAATCTAACAATCTATCAATTCTGTAAGTTAATGGAAAATCTAATAATCAATCTAAAAGAGGTTAATAAAGAAATGGGAATGATTAAAAATCAATTATATCTAAAATTGTATGTTCAATTATTAATTAGAAAACGTAATAAAATAATTAATCAAGCTAGAATTGTAAATAATGTATTCTCATGGCAAAATCTATTCATAGGCCTCTCTAGTACAATTGGTTTTATTGCTTGTGTATACGAAGTTTTTGTTGATGCAAACAGTTCTGAAGATAACATGTTATTTAGTATAGTAATGACTATTGTTTTTATTGTTTACTACATTGGAAATATATTTACTGTCGTGATTTATTGTGAAAAATTTGGAAATCAGGATGAAATTTTCAACGAACAGTTGTTCCAGCTTACAGTAGAAAGAGTTCTCCTTGGAAATCAAGATGATGATCTGTGGTTTTATGTCTCTCGGAAGAGAAACGTCAAGTTCACAGCTGCTGGGTTCTTTAATCTGGGATATCCACTTATTACTTCTATAATTAGCAGCACAATAACGTTAGTGGCAGTTCTTGTACAATTTACTTTCTAA

>GR10

ATGTTTGATTCAAAACTAACTTCATTCGTAACAAACTTAGCAGGTTTATGGCTATTTAATAACGATTATAATATTAGCTGTTGGAGTTTAATTGAATCTATATTACTTAGAATTTTGGAACTTACAGGTATAATCCACGAAGCATATCGTATCAGCAAGCATAAAAAAGGTCTTGATTTATTGTTGCGTACCATTTTCATGATATTATCATTAATCAATCATACCTTGAATTTAGTGCATATGTTTTCCCAGTATCATTATCGAAATTTATGGAGAAAATTGATTCGTTATAAAACTAGAAAGGCAAAATTATCTACTTTGATACTTGGCTGTAATTTTGTCTTGTTATTTACTGGAACATTTGGATTTTTGTTTACTTCCAGGAACGCTGAAAGAAATGATGTACTTCTATGTGTTTATATAGTGCTCAAGCAAGTTATACATTTTTTCAACAATATTTCGTTCTATCAATTCACTGCATTCATGGAAAATCTAATCATCAATTTAAAAGAAATCAATAATGAAATGGAAAAAATTGAAAAACAATACTATTTTAAAGTGTTTGCAAAATTATTGATTAGAAAACATGATGAACTTATTAAACATGGTAGATTAATAAACATTGTATTTTCATGGCATAACCTATTATTGTGCATAACCTGCACTTTAGGTTTTATTGCAAATCTATATCGAGTTTGCCTAGCTGTGTACAGTGTGAACGATCAAATCTTATCGATTATAATTGTGGGTCTAATTTTTGTATCGTATTACGTTGGTTGCGTACAAACGGTTGCTTATTTTTGTGAACAGTTTGGGAAACAGGTTGAGAACTTCAATGAACATCTGTTTCGATTCACGACAGAGAGATGTTTTCAAGGAATCAAAGATGATGATCTGCGATTTTATACCGCACAGAAGAAAAAAGTTAACTTTACAGCAGCTGGTTACCTGAATCTGGGCTATCCACTCGTCACCAGTATAATTGGCAGCACAATTACATTTATTGTAATTGTTGTACAATTTAGTTTGTAA

>GR11

ATGGAAACTAGAAAATCAGACAATATTTTCGGCAGTTATGTATTTTTTGTAACCAAAGTTATTGGTCTGTGGATTTATGATAAGAATATGGACATATCAAAGCGATTGTTAACTCTAGCTATTTTAATTCGTTTCATTGAAATTATATTTTCATTAATAAATATTATTAACATATTAAAAAATTTTGAAAATAATGGAAATCTCCGAACAATTGCTATATTGTTTTGTTTAGCAAAGTTATTATTTGTTTTATTGAATTTCTGTCATATAAATTCTGAATTCAGAGAGAAATCACTTTGGAAATATTTAGCTGTACAAAAGTTTAACAGAAATCATTTTAAACTAACCAAAATCACATTATACTTTGTAAATATCCTCTTTATACTACTATTAGGTGCTCTAGTTTTTGATGTAATAAAGAAATCTATTCCATGGTATTTGTTACCAGATAGGTTACTAACAATTCATTTTTATTGGACATTCGTAATAGTGATAATCCATTTTTGTACATGTCTACAAATATTGATTACATTAATGAAAGAGATAAATATAGAAATGGCGTCAATGGGCGATCAAATTCGAACAGCTGATTTGAGAAAAATTAAATTATTAACCAATCAACACAGCTGTTTGATTGACATTGCTGAAATTGTTAATAGAGCTTTTGGTTGGCAAAATATTATCATTTATTCAATATTATTCATTATAACCATTTCAAGCCTGTTTGGCTTTGTAAGGAGATTCACTAGTGATCTATTGATTGTGACCATTGCACGTCAAACTACAATTTTCTTCTTATCCTTTGGTTTAATAGTTGTCCCTGTATATTTATGTGAAGATTTTAAACAAGAGGTAAATCAATTTAATATGAAATTATTCTATTTTGTGAAAAGGCGATTTTCCGATGGTTTTCAAGATGATGAATTGCAGTTTTATACAATAATGAAGAGAAATATGAGTTTCACAGCTGGAGGTTGTATCAATCTAGGTTATCCATTAATATTATCGATAATTAACAGTGCTGCAACGTTTTTAGTCATTTTAATCCAATTTACCAACTAA

>GR12-FIX

ATGGAACAACCAGTGAAAGCTTGGATATCACAGTCTAGCCAGGAAATGAGAAAAATAGAACCTAGACAAAAATTAGCAATTTCTTTGCAGATTTCCAAAATATTCGGTTTATTACCACACAATGATAACGCAGAATTATCTAGATTCTGGTTGGTAGAAGCGATAACAATTAGAATTATAGAATTAATTATTGGGATAAGTGGTGTTTATTTTAGTTTAACGGTGAAATATTCCCCAACAGACTTATATTTAATGTACTGCACATTTACTGGCACTTTGTTAACTACCATTTCTCATTTAGTATCATTAATCATAAACCATGATAAATGGCAAAAATTAGTAAAAAATAATATTCAAAATTATTACCCAAAATATTCTAATACTTATTTTTATATATGCATAATCAGAATTCTAATACAAATTTTATTGAATGTCATGACTTTTTACTTGCAAGATACAGATCTTCTTGAATTGGCCGATAGTTTAGTCTTGAACTACACCTCCATGATTGTGAGCATAATTACAAATCATTTTTGCGCTAATATTGAATTATTAACAAAACAATTGCATTTAATTACAAATGAGTTGATTAATGTCAAACGTTTCAAAGAATATAAGCCATTAAAATTGAAACAATTAGCAATGAGACAACATCAATTGATAGATTTAGGACAAATTATTAATAAAATGTTTGCGATACAAAATTTAATCATTTGCACAATATCGTTAATTTGTTTACTGGACACTACGAGTGAATTAATTGATACATTTGCTATAGTAGAACCATTATCTACTCGATTAGCTAGAATATCGGACAATATTTTTTATTTAAGTAATGCCCTGTTTGTGGCCTACTTTTGTGAAGTGTTCGCTGGAAAAGGCGGAATATATCTACATTGCTTAATGAACAGAAATGTTGAATTCACAGCTGGAAGATGTTTTGTTTTGGGTTTCCCTCTAGTAGCATCGATGATTGGTACGGCTACAACATATTTAGTGATTTTAAAGCAAATGTAG

>GR13

ATGGTAAAATCAACAAAAGTTTGGACTGTCCCATTAGGTGAACAAGTGAACAAAACTATTCCTCCAGCGAAACTAACCGTTGCTATGTTGATTTCTAAAATATTTGGTATATTTCCTTTCAATGAACTTTCAGAATTATCTACATTCTGGTTTGTCGAAGCGATAGTTATTAGAATAATAGAACTAATTATGGTATCGATTAGTTTTTATTTTTGTATTCAATTAATAATACAAGATCGTGTAAAGACACTTTATCTATTGCTTTTTGTAAATACATGTACATTATTTACGACGAATTTGCATCTAGTCTCATTTTATAGAAATAGAAAAAAATGGCAAATAATTGTTTATAGTAACAAAAGTCATTATCACAGAAAATCGGATATATATTTCTACATTGATTTGATCAGAATAATTATTCAATTTCTAATTAACTTCAGCCTTTTTCGCCTCAACAAACATTATGATTTTCTAACTATTAGAATGTGTTTGCACTACGCAGGTTTCATTGACTCAATAGTTATAAATAATTTTTGCGGGAAACTAGAAATATTAACAAAACAGATGCGTACAATTGATAGAGAAATAATAAAAGCAAAATATTTTAAAGTAATTATTCCCACCAAACTAAAGCAATTAGCTAAGCGCCAAAATTATTTAATCGATTTAGCGCAAAATATTAACAATATTTTTGCTATTCAAAATTTAATAATATACTCCAGATCAATAATTGGTTTTATCTGCGTTACCTACGAAGTAATTCGAATTTCTGCTACACTAAATATTCAATCTAAACAGTCGTTCACATCAGGAGTTTTGGAAGTTTTGTCTTATTTTGGAAATATTTTATCGACTGTTTATTTTTGTGAACAATTTTCTGAAGAAGTGGAAAATGTCCATCAACAGCTTTTCTCAACGATAAACAAGAATTTAGGAACTGATATTAATGAAGAAATACATCTACATTGCGTCATGAATAGAAATGTTAAATTTACTGCGGGAGGTTGCTTTAACTTAGACTATGCTCTCGTAGTGTCGGTGATTGGAACCACCATTACATACATAGTGATCTTGCTACAATTGTAA

>GR14A

ATGTTACCCTGGAAGAAATCTAATAAAATCTTTAATTCTTCAACTATTTATGGCAAAAAGAATAATATCTTAATTACAGAGATGAAATTTATGATCAGATTTAGTAAATTGTTCGGCCTATATCCGTACAGTGATGAATTGCAGTTAACACTGCCATCAATACTGTTCACTGGTTTGATGCGCATATTAGAAATTATCAGCTGTTCGCTGTATTTGTTCGTAAGAGATGCAAACCAGACAAGTGGAGACACCAAACATCTGATCTTAGAATTACTAATTCACTTGCAAATCTTAACATTAATACCAATAAATCTCTGCCATATTTACGTCCATTTTAAGCATAATCATTTATGGAGGATATTAATTAACTATATCGACGTAAATATAAAAATTATTTCAACGAAAATGTTTATTTTATTTATATTTTTGTTTATTTTTTTGAAAATGGTGTGTTTCATATGTGCTGGTCGTTTCGATTCATTATCGATAGGAAAACATTTTCTTTATGTCTATCGAGATTTAATTCTAATAATTATTTCATCGCAAATTTTCCTATTATTAGAATGCTACACTAAACTTTCACAACAATTAGTGGAAAAATTAACTGTTGACAAATATCGCAAAATGAGTTACATAAAATTAAATGATTGTGTACAATTACAAGATCATATTCTGTGCAACGCTAAGATAATTAACAATACTTTTGGTAAACTGATACTTTCGCTGTGCACATTCGCTGTGTTCGCATTCATCAATCTCACTTATGATATTATATGCGATTACAAAAATCAAGCATTGTCCTCTACATTGATGAAATTGATTTCTAGTTTTCAAATGTTATCGATTATCATCTCTTTTAGTTATTTTGGTGAACAGTTTATGGAAAAGGCTGAGAATTTCAATTTAAATTTGTATAAATTGCTGGAAGCAGATGGAAATTTAGATATATATGTTAGTGGAAATCTACGGTTTTATTGTTTGATGAAGCAAAACGTCCAATTCTCGGCCTGTGGTTTTTTTAACATCGGATATCCTTTAATAACCTCGACGATTGGTGTGGCTACAACATATTTAGTAATTGCTGTTCAACTAACTACATGA

>GR14B

ATGGTGCGGTTTCTTAGTAGTTTTAATAGTTTACACAATATCTGCAGATTTTATGGTCTATTACCATTGAATAATAATTTCCAACTATCCAAACTATTTCTAACGGTGACCCTGTTGGATAGACTGTTAGAAATGATAGTTTCCATTTTGTTTATATATTTAAGAACTTCAACGGCACAACCCCATGGCTTGGAATTCATTGCTAAAGAATTCACTTTATATGCCCGTGTGATTTTCATTCTTCCAGCAAACTTTCTGCACATTTATTCTTTGTTAAAGAATAGAAAATATTTGCAAAAAATACAAGAACATATTGGCCACAATGTGGATGTATCCAAAGTGAAATTATTCATATATTTATGGTTATTATTGGTTGGCATAAAAGTTAGTGCAGTATTCTACACAAAATTTGGATATTTATCCAAATTTGTACGTAGAGTAATATATACTTACTATGACATCAGTTTAATGCTATTAATAGCACAATTCTTCATCCAGGTAGAACGATTAACCATACTATTGACTCGATTAGACCAACAATTGAAATCGATAATTCTATTCGATTCTAGAATAAATATAAAAAAATTGTTGTCTTTGACAATAAAACATCAGAAATTAATCTATTTTGCATATTTAATTGATTCTATATATTCTAAAGTGTTACTATTTATTTGTACCTACTGCTTTATCACCTTTGTTATCGTTACTTATAATTTGATTTGTACCTTCCACGGAGTAGATATTACTTTTCTAGTACAGAAATCTGTTTCTATAATTATGGTATTGTTGGCTATAGTTTCTGCTGCACATTTCTGTGAAATGTTTGTTAAAAAGGCTGAGAATTTCAATTTAAATTTGTATAAATTGCTGGAAGCAGATGGAAATTTAGATATATATGTTAGTGGAAATCTACGGTTTTATTGTTTGATGAAGCAAAACGTCCAATTCTCGGCCTGTGGTTTTTTTAACATCGGATATCCTTTAATAACCTCGACGATTGGTGTGGCTACAACATATTTAGTAATTGCTGTTCAACTAACTACATGA

>GR15

ATGTCACTTCCGTTAGTGTTTACATACGGTGAACGTAAAAATCTACGCGTAACCAACATATTTACGAATACCATTGATTATAGTAAACAATTAGGCTTACCATTTACTATTTCCCGTTTACTAGGCTTATTTCCATATGATAATTACTTTAATTTCTGTAGATTTTGGTATATTATCGGTATAATTGATGTTATATTTCAATGCGTGGCCATCTTGATGATTATTTTCTTTCAAACTTATAAACATATGTTAGAGAATAAAACGGAAATATCAATACTATTTACGCTAACTTTTAGTTTATTTTTACAGAATTTCTTACATTTATATTCACAGTATTGTGGCGAAGAGATCTGGCTGAAATTCTATTCATATCTGAAGTATTACACAAATAGTAACAGAAATGTTGGTATTACTTGGCCCAATATCATTTTTTGGGTTTCATTTAGTATGTCCTCGGTTGGATTACTGATCAGAATTATCATATTATTTGTGGATAACATAAAATCATTTATAATGGCTTTTTCATTTCTATATCTTTGTGAAGCTTCAATATTGTATCAATTTTGTGTATACTTGGATTTATTAAATATAATCATGAAAGAAATCAATCATGAATTGATTGCAATCAACGATGTTAAATATGGACAGGAGAAACTGATTGCTTTAACAGATAGTCACTCCAGTTTGATTGATTTAGCTGAAAAAGTGAATCTTGCTTTTACTAAACAAATGCTGGTGATTTGTTTATCAACTTTGGCGGCATGTACTGGCGGTATTTATAGTGTCATCAATGCGGCCGTTTGTCGGCCGACTGTTCATGTTAAGATTATCTATAGGCTGGAGGATGTTATACGTAGTTTGTTCAAGATTTTTGCTATTGCTTATTTCTGTGAACAGTTAGGAAATAATTTTAACGTTAAATTGTTCTATCTGCTGAAAAAATCTATTGGAATGAAGAAAAATGAAGAGCTGGTATTTTACTGTCTGAAAGAGAGGAATATCAAGTTCACCGCTGGAGGTATCATTAACTTAGGATATCCTTTTATCGCTTCCATGTTGGGTACAGCGATCACATACGTAGTGGTTGTAATACAGTTCAGCTCATGTTAA

>GR16

ATGATGACGACACCACAACAACATATTCACTACCATCCTAGATTCACCTATGAGAAACAGATTAGTGTATTGCTAAGTGTTTGCCGATTGTTTGGCCTATTTCCGGTTGATAATTATTATAATTTTTCCAAATTTTGGTATACAATTAGTATTTTGCTGAATTTATTAATTTTCTTTCTATTATTGTTCATACTTTATTGGAAATTGGTTTTTCTAGTAATTGTACGTAGTAAACACGATCACACTAGAAATATAGTATTGTTAGGCAAACTATTCGATGAATTATCTATAATAGTGATACATTTGTATAATTGTTATAATTATATAAGCATTTGGAAACAACTTATTTTATATAAAACAAAATTAAATTTACAAAATGTGCTAATTAAATCAAATATTATAACAATTTTTACAATTGTCTATTTTCTATTTGAAATTCTAATCTATCAAATCGTCTATAAATTTTTAATTAATAACACAAATATATTCACTGTTTTATTCTCAATAATTAACGTATTTTATAAATTTAGCAATACAATGATTTTATATCAGTTTTCTATGTACATAGAATTATTAACTCAACATATAATTAATTTAAAAAATCAATTAAATATAGAACGTATACAAAGTACACAAAACTTGATTGCATTAACTATTGAACACAATCAATTAATTGAACAAGGTTATCATATAAATTGTATTTATTCTAAATCAATTTTTATCTCTTGTACGTCAACTTTTGTTCAAATATTGTTCGGTGGTTATTTGATCATGCTGAAAGTTGTATGCAATGATCATGATCTAAGTTTTTATGGGATCAATGAAGCTGCTCATGGTTTAGTGAAAATTTTACTTTTGGCTTATGTTTGTGAAAATTTTCATGGAAATGCCGAGCAGTTCAATGTGAAATTGCATAATTTGCTGAAGAAGTATGAACACTACAGAAATGAACAATTAATATTTTACTGCTTAAAAGATAGAAATGAAAATTTTATGGCAGGAGGTTTCATTAAATTAGGTTATCCGTTCATTGCGTCCGTTCTGGCAACTGTGGTTACTTATGAAATAGTTATATTTCAGTTTACACCATGTAAATAG

>GR17

ATGGTATTGAATAAGTTGACCAGTGCATTGTTTAGTAAATATAAACCATTTAATCAAACATTTGTAATTAGTCGATATTTAGGTAAATTTCCATTTAAAAATTCTAAATTAAGTAGAAAATGGTATTATATCAGCGTCATCATATTACTGATTGAATTAGGCATGTTTCCATTGTTTTGCTATTTCTATAGTGATTATGAGAAGATAAATTTACTAAAATATCTGTACGGGATGTTACATTTTATGGAAATAATAAATAATATTTTGAATTTATATTCGTTAAACAATAATCAGAATATTTGGAATAATTATGATCATTTGCATTATCAATATATTTTGCAGAATAATATTGCTTTTAATAAAATAAATAAATCGGATTTATTATTCTTTATTGGTATAATTGTAACATTATTAGGGTTTATTGTAATGATGTGTACATTTCCGCACAATTATTTCCTAATAATATTATCAATAATATTGTTGTATTTCCTGTTATGTTCTCTTGCTATCGCTAATCAATTTTGTAAATATATAGAAATGTTAAATACAATATTAGTAATTACAAACAAGCAATTGATTGAATTAAACATCAATATAAATGGTAGGTCTGAGATTTTACAATTAGTAAATATTCATTCTAACATCATTGAACTGGCTGGTAGAATTAATGATGTCTATTCAAAACAAAATCTATTGATTTGCATGATATCTTTTACTAGATTTACTACATCATTATATAATTTGATTCTTAAAGCAGTGGTATGTCCAATAATCTCTAAAGAGATCTTTATAGTTGCTGAATTCACCGCTGTTAGTGTTATTCATTCTGTTTTGAAAATAATTATGCTAACAACTGTTTGCGAAGCGTTCAGGCACAATAGTAATCACTTCAACAAACAATTGTTTTTAATAATCAATAAAAACATACACTTATTTACACAGGATGAAGTTTTACTCTACTGTTTCAAAGAGGAAATTTGCCATTTTATTGCCGGAAGTATGTTCAACATTGGCTATCCATTTTTAATATCTTATTTTGGCACGGCTGTCACGTTTTTAGTGATATTAATCCAGTATATACAATGTTGA

>GR18

ATGAGCCAACACACGAATGCAGGACTGTTTCTTCTAGAAAAAGTCAGTCCTCTATTTCCTATAGCCAGACTTTGCGGTGTCCTTCCCTATAGTGACGAATTTTCCTTGTCAGAAAGTTGGTTTAAATATTCAATTTTACTCGTGTCATCTGTAGTTGTTGGTGTAAATGGATTCAGAGCAGTTTATTTAGTCTATTCTGCCAGCGATTTTCTCAATGAAGGGTTCAATACAGCCTTATACATTATTCAAAGTTTACTTAATGACGTATGCTTAATTTCGCATGTAATCCAATTGTTGTTCCAAAGAGACAAACTAAGAACAATTTTAGAGCGATTGAAGACGTTGGATACCATTTGGTTAAAATATACAACAATATTTTCGAAAAGGTTAACAGGTTTTGGGCTTCTATGGGTAGCAATATCGCAGATAGCAATCTTAGTTACTGATTCCACCACTTATGAAAAAATGATCCGTTTTACTTACTCAACAACTTACGTTTTCTTCTATATGATTGTAATGGAATTCTGCTGTTGCCTAGAGTTAGTTCGCAATCATTTGACCGCACTAACGATAGATCTCAGCCCTGTAGAATCTAAATTGCAAAATAAAATTTTTTCACATTACGACTTGTTAAAACTTGGCAAAAAAATCAATGACTTTTTCTCATTACAACTGTTGATCATTTGCATGAGGTGTACAATAAATATTGTATGTCAAATCTTTTTTTTATTACAATTTTTGAAAAATAGTAAATGGGGAGTTGAAATGATTTTAGTACTGGCATTCCTGGTCGGCGATTGTTTTATGGAACTTTTGATTTTGCTTTGTATTGCAGCTTCTTGTTCTGAGACTTCGGCGAAGGCTGCTCGCTTTAGTGAACAATTATATCAAACAATGAATAAAAACAAAAACCTTTTTTCATATGTAGAACTGCAGTTATATGTCACACTAAATAAAGGAGTACAATTTAATGCATGTGGATTTTTCAATATTGGATATCCATTGGTTACATCGGTGCTGGCTGCAGCTACTACTTACCTTGTAATTCTAGTTCAATTTAGCACATCTGGAGCTTTGAAAAAACAATTAGGAAAGTCAAATACGACTTTTATTCAATAA

>GR19

ATGGCTACCGTACATATCGGAGAGTCCCCCAGAAAATTAATATGGAAAGAATTGTTTCCATTATTTACTATATCAAGATGTTTAGGAATTTTCCCATACGATAATCGATTTAAGTCATCAAAAATCCTTGTTCTATATTCTTATATAGTTACCACATTTTTTATGTCCTTATCATTAATTCTAAAACTTTGGTATATGTTTAGTGTAAATGATCCATGGTTTGTCACATCTGATAGGATATTTTTAGGCATTATCCAGATTTTTGGTGAAGTTGGTACAGTTTGTGTACATCTATTGTGTGATAGAAAATGGAAACTTGTATCGAAAGATTTCCATGAAATTGGATTGGTTAAAACGAAATTTTCACAAATGTTTGCAACATGGTTGACAATATTTACATTAATATATGTTTTTGGATGTTGCATGGTATATTCATTTTTCACAGAATGGGCAAATATCTATGGCTTTTTTTTAATTTTCTCCAATCTATTTCATTCTCTTTTTTTCTATATTATTATTTTACAATTTTGTAGTTCTATAGAGATTGTTAATTGTATGCTAAAAGATGTTAACAAACAATTAAATCTATTCCGAACACCCGACGAGGTGTTAAATTTTATAAATTTATATTATCTTTTGCAAAACAAATTCATAAAAATTAATAACAGATATTCACTTTCAACGTTGACAGCTATATCAAGAATAGTAGTGCAACTTGTACTCTGTATATATTTGTCTATTAATTTATTGAACGTTGCATCGCAGGAAAATCTAACCCAAGCAATAATGATATGGATCAACAGTGTGTTCGAAGTGTTCTTACTTTATTGCCTGACTGGAATTTGTATGAAAACTTATAGACAGGACGAGCATTTCAAAAACAAAATATTGTTTCTCATGCTTAGAGATGAAAACTTATTAAAAAATGAGAATTTACAAATATTTTTAAGAAGGAAAACTCAATTGATTTTAACTGCTCATGGTTTCTTTACATTAGGATATCCATTTATTACATCGATTACAGGAGCTGTAGTGATGTATGTAATAATATTGATGCAGACAGATCAATTAAAGTTGGAAGAAGTGGATGTTTTAAATTAA

>GR20

ATGGAGAAATTATTGAAAAACCACATCAAAATCTTTAGATATTTAGGCATATGTCCGTTTGAACTAACCAATCACGGTAAATTAAAACTATTAAAAACATCGCTAATTTATACAATTGTAGTACTTTTAACTATAGACACATTATACATAATTAATTTAATATCATTTTTAAAAAAAATATCAAAATGGAATAAACATCAATCATTTGTAGCAGGTATGTTTCTAATATTACTACCATTAACCATTAATATCGCTGTAGTTTATATAATCACCAAGAAAACACAATTGGAAAGTGTAATCAAAATATTTGTTAATTTCAAAAATAAATTTCCCGAATTCATACCGGAAACAAAACTGAAATATTATCCAATCTGTCCAACATTTATAGATTTAGTAATTATTTTTGTTGATGTTCTAGACGAGCTTGTATTTGTTGAATATATTCCATATTTGTATCTATTTGGTATTCTAGTATTCTTCACTAGAACTGTAATAATTAGTAAATCTTGTTTAGTACATATGCAACAGTTTTTGAAGAATAAAGGTGACGTAACTAGTTTGCCTTTAATATTGGAAGCTCATGATTTAATATTGGATTCTTGCGCTAATATTAACTCTGTATTCGGTCCATTCTTATTGGGCACAATCTCAGTTTCCTTTTTATATCTTACTATCGGAATTTATTGCTTCACACTGTCACATGCCACCCCATTGGGCTATATTCACTACACACTGATTGTCTTTGCTAACTTATCATTATTGTATGGATTAGCAGATCTATGCGTATCAACAATTGATGATTCCTTACAAATAAAGCAACAATTAACAAAATTAGTTTATCAAGATTATTCTAGAAAATTTGATAATTATAGAGTGATTGTAATGTTTCAAATTATACAGAACAGAAATCCAGAATTCACTGCGTACAAATTCTTTAATATTTCCTTTAATATGATTGGCTCTATTGTGGCTGTAATCTGTACATATGTGATTATTTTAATACAATTCGATATTTCTGTTGAACCGCAATAA

>GR21

ATGTGCAAAGTGAACACCGTTGATAATATATCTGCCTGGACGGCTCAGATTACCGAATCTATCCAATCTTATCAAAGACCGTTCAGATATCTAGGTGTATTTCCGTTTATCGAATCAACTGGGACTACTAAAGTGAATCGATTAGCATTAGCCTGGTCATACTGTATAGCCACTATAGTATTAGCCTTATCATTACAATATCTAGTGATTTCACCAACAACATTTGACTTGCCGAAAATCTTAGTGATTATAAGTAAAATCTTATCAATAACCGTGGCCACTTCGCAAATTGTTTCTGTAACATTTTTGGTCATCTATAGGCACACCTTAAATTCAGTTTTTCAAATATTGCGACAAATAGATAGGAAATTAACAGGTTTAAATCCAAAGTTCTCATTTAAATATCCAATTTCCAGTATAACATTATTGATGATCACTTTAGTTTTATTGATTATTTGTAGTCCATCCATAGAATCCCGAAAAGAAATTTCAGTTTTCTGGTTAACCACATTTTTCATCCATATTATCGCTTATTTATACACAAGAACCATATTGTTAATCAAATATTGTTTCAATTTTATCAATAAATCGTTAAAACAAGAGGCAAACAATTTAGCGATGTTACTGGATTGTTATGATCTATTATTGAACTGTTGCGATGATTTCAATAAATGTATTGGACCATCATTATTAACAATTATGACGTCAAGTTTTTGTATACTGACTTTTGATTTTTATCTATTGTTCTCCACAAAATACAATGATATCTATGTTAATCTGCGTAGCATTATTGGTATATTATTTTATATAAATCTAGTGATGCATGTGATTTGGACCAGTCATTCTACTGTCAAAGAGGCTGAATCTTTTAAGGATACAATCACCAAATTTGCTTACGAAGGAAATAGTGATCAAATTAGAAAAGCCATGATTCTATTAACGAGTGACAAAACCGTGGTATTCTCAGCTTGTGATCTCTTTGACATGGATTTCAAAATGGTTTGTTCAATGGCTGCTGGAATCACAACTTACATTGTGGTTCTCTTACAGATGGGCAATGATAACGTCGCTACATCTCCTGCACCATCAGCTGATACAGTTTAA

>GR22

ATGAAACGATTTTGTTGCGTTCAATTGTCTGGCGGTGTTGATTCAGCCTTTCATAGCGCATCCAGATTATCTAAATATTTAGGTGTATTCCCATACGAATTAAAATCGAGAAAGTTAAAATTGTCTAAGTGTGGACTATTATGGTCACTATTATTAAAGTCTATTATAGTGACTATAGAAATATTATTAATTTTAGATCCACCAAGTGAATTAAATAAAAGTTTTATCGGTACTTTCTTAATTTGGGTACAACTTGGCTCGCTGACTGCTGGTTTGATGATAAATCTATTATGGCTACACAACACTAAAGAAAAATTAGAAGTAGCATTGTCATTATTGAATGATGTACACGATAAATTAGTTGATTCTGAAATGAATGTCTCATTGGTTTCACCATTTCTAAGTTTGTTAATGATAACTATCATAGCTACAGTGGGCAGCGCATTGAACTTTGATTACAAATTAAATACTACCATTTTATACATATCTTACTATCTTACATCTGTAATGATTTGTGGAGTTGCATTACAATTGAGTGGCACTTTACAATTTACTAAACATTTTTTTAAAGAACTTCGATTACGTTTACCAAAACCATCTAGTCAAACCTGTGCTTCTGTTGACCTACTTATAGAATGTCATGAAATACTAATTACTGCTTGCGAAGCAGTTAATCAAGCTTACGCTCCACAACTTTTATTAATAATACTATCGTCATTTGTATTGGTTACTGGCAATAGTTATTCCGTACTAGTTTTTAAAAATCCATCAGATATTGTTCTTTTAATATCATGGGCATTATTGTTCGCACATCTAACATGGCATATTATATCAGCATGTGATGCAACTTATACTGAGGCTGAAGAATTTGACAAAACTTTGTATCGACTTATTTTAGACGACAATGTAGGAATAATTTCGCAAAATATGAAAGTGTGCATGCACTTCACCAATAAAAGAACAGTAGAATTTACTGCTTGCGGATTTTTTAGTATTAATTATACTACTACCAGTTCGATGGTTGCCACAGCCTGTACCTACCTGGTTATTTTAATACAAATGGCTGGTATAAAGGAAAATTCAACTCAGATAAATATTAGATAA

>GR23A

ATGGAGAAATCTCTAAAGAAATCTCTTGGTATATTTAAATATTTGGGTATTTTCCCTTTTAATTATTTTAGTGGTGAAATTCTGTTTTCTGAAAAATGGTTTTTATACAGTTCTATGCTATTTTCCATACTTTTATTCAATAGTGTATGCATCACATATAATTTGCACACTATTGATTTACCAGTAACTCAGCTAATGAAATTGATTATCAATTATTCACTGTTGATTTCAATTTCACAATATATTCTTGGATTTTTTGCCAGTGTATATTACGTGGACGAATTGAACGTGGCGATTAATCGTTTCTACGATATAGAATTATTGATCGGAACAATGAATGTTGGCAATAATAAATTTCTAACGTTATACTTGTGCTATATATACAACACCTTTATCATGATAAACTCACCACAGATCGATTTATTCCCAAATTCCAATCGACTACAAGAATTTTTCGCATCAATACTAGTTTTTCAGATTATAAGTTTGAATTATTTATTATTTTATATGATATCATTTGTATACAGTTTATTAGATTTAATTGTCAAAAAATTGAACGAAATGAATCATATTAAGGACTTGGAGTTACTATTAGAATCATATTTTTTATTAAATGATTCTGCCAGTCATTTACAACATTATTTCAACATTCCTCTGATAAATATAAACGCTGGTTCATTTTTCTCTATACTGACTTATATATTTATGCTAATCAAATTGAAACCTTCCCTTAATTTGAATTTAGTTATAATCATCTGGCTAGTTTTAACGATACTAATATTGTTTGATATTGCATTTATTTGCCAAAATTTACAAAAAAAGTGTCATGAGTTTGATAGAATTTTAAGGAGAAAAGTATTCGAGGATAATGTCGGCCATATTGCGAACAATTCTAAAGTATATCTACACTTTACAAACTGTAGGATTATCAAATTTTCCACTTTCAATTTCATTGATATAAATTACAAAATGTTAAGCTCGATGTTGGCTGCAATTACTACTTACCTCGTCATACTTCTGCAAATGGACGATGAGCATGCGCAACAACTCCACGAGATAGCCAATAATTATACTAATAATACCCAATGA

>GR23B

ATGAACGAAAATATTTTAATATTTCAAAAATATTTACAAATTTTCCAATATTTAGGTGTTTTCCCATTTAAATTTCGTGAAAATCAAATAATTTTAGATTATTTGAAACTTTTCTTTAGTTTTCTATTAATTATTGGTTTTTCTTTGTTATTTTCTTTATATTATTTAAACATTGATGAATTTATTTTTACTGAAACATTATTAAAACAAATATTATTTTCAAACGCAACTATTTTATTAATTGTTACATATTTTTTACATTTAATATTTTTAATATTTAATCGTGTAACAATTAATTTTGCCATTCAATTGATTTTCAATATTAGTAATTCAATTGAATCTTCAATCAATTATCGCTTGAATATTGGAAAAAGTTGGATTCTATACACAATATTGTTTCTGCTCTTTATTACCTGTGCACCATTCAATTATATGTCCGGTTTTTGGAACAGAACTTATCCATTTTATTCAATCACGCTTGCATTCAACAATTGTATTATTGTATTATTAATTGAATTATTTTTCACGTATATATTTATATTGATTAAAAATATTAATACGCTTTTAACTCAACAAATAAATGATATTAATCGATTGGAAAATCTATTAAATTATTTTGGAAATATTATAACAATTTTGAAATCGATTGAACAGAGTTTTAGCTTGATTTTGTTAGTTTTATTGGGTGAATCTTTTAGTTTGGTTACACTGAATTTATTTTTTACTCTTCGAATAAGTTTTCGAGGAATTGAGTATTTTGCTGCCACATTGTGGACTGCAATATTTGCGTTTATCGCTTGGCACATCGCTTATACATGTCAGTCTGTGCTTTATCAGTGTCATGAGTTTGATAGAATTTTAAGGAGAAAAGTATTCGAGGATAATGTCGGCCATATTGCGAACAATTCTAAAGTATATCTACACTTTACAAACTGTAGGATTATCAAATTTTCCACTTTCAATTTCATTGATATAAATTACAAAATGTTAAGCTCGATGTTGGCTGCAATTACTACTTACCTCGTCATACTTCTGCAAATGGACGATGAGCATGCGCAACAACTCCACGAGATAGCCAATAATTATACTAATAATACCCAATGA

>GR24

ATGTCTCTGCCATTTAAAGCTATGTTAGACTGGATAAATGATGAAAAAATTAGTTCATTCTTTTTATTGCATCAATTGTTTGGGACTTTTCCGTACCGGGTAAATAGAGGAAAATTTACTTTCCATAAAAGACTTCATTTTTGGAATTACATTGTTATATTTTCAATTGTTGGCACCTGTTTTTCATGTATTTTATATGTCCCACCTTTCTTACAAGGTACTCTAATACTGGTATTTGTAAATGCTGGACAAATGGTGATATTAATGGCACCATTGCTATTATTTTCTATTATTGTAATTTTAAAGAAAAGTAAATTAGACAAATATTTGTATTATATACGTTCCACTGGAAATTCATCTTTTGGGCATTCAAATACAGTTACAATATTAATTTTATCATTGTGTCAGATAACGCTTGTATTTTTTATAATGACCGATTACAATATATTTATTGCCAATATGAATATATACGTTGTAGATTACTCGTGTTTTCTAATGTTCATATCAATTGGTGGTCAATTTTCTGGTATTTTAATTTCTATTAATAACAGATTTATTGTTTTAAGAAAAGCTTTAAGCAGCGGAAATCCGATAAAATTGGTCGATTCACTGAAAACATATGAAAAATTAATCACCATTTGTGAAGAGGTTAACGATGTCTATGGATTTCCAATTATATTTGTTTTTGTATACTCGTTTGCCTTTTCAATTACTGAATGTTATTCAGCACTGAGTAAAACAGCTCCAAATGCTTTAATGTACGTAACATGGTCTACTCTTACTTGGTCCACTGCTTTTCGACTCGTCTTCACCTGCTCTCAAGTGATGTATCAGGCCAAAGAGTTCAATAAGAAACTAAAAGTCTCAACTGCAGACTTCAGGTATCTGGAAAGAAGTGTACAGCTTAAAGTTCTCATACACTTCGCTCGTAGACAGAAATTAGTATTTACTGCCTCTAAGTTTTTCAACTTAGATTTCCCATTTTTGGGCAAGATACTGATGCAAGCAGCTATTTACTTTCTATTACTTATACAATTAAATGGATTAATACCACTTTTTTAA

>GR25

ATGTTCGAACAATTGAAATGGCGAAAAAGACTAGTCGCTTTGGATGTATACCAAGCTTTAAGACCAGTATTTCTTATGTGTAAATGCTTTGGTACTTACAATTATTCCCATTCTAAAATAAATAAACTTTTGCTACGTTGTTTCACAATCTTGCTGACCCTTTTTATCACCTTAGCCACTACTTACGCTTTGGAGTTTATTGATTTAGTTGAAGCTGAATGGTACGAGAATTTAGTATACTTTTATATAGAAAAAACCCAGATTACACTGACATATATTTGCTTCATTGTAACTATAAGTATGCATGCTATAAGGCAGGAAAAAATAGGAAAAGAATTACTATCCATCCGAAACATTGACCTTCTCTTGAAAAAGCAAAATTTTAAATTTATTTATTCTACCTTAAACCATCTATGGGTATCAAGCATGCTAGTTACTACTGCTTTACTCACTTGGTTCATAGATTTCTGGTTCTATACAAACAGTGGCTCTTTTTACGCTAAAGTAGAATTACTATCATGCTATTTCCCTTTATTAGTTATTTACAGTTCAATGGCGCAATGGTATAGCATAATTAAAATCTTGGGCGATCATTTTGTACATATTACCATTAGCGCTCTATCAACTCCATGTCCTGATCGGCTGTCGATTCTCGTCAAAGTACATGGAAGATTGTGCACTGTTTCCCGAGCAATCAATGAATTATTCGGACCTTGTTTACTAATCTGGACTTTATTTGCTTTTGTAATGATGACTGAATATTTATTTATTGCTATGAAAGTAGCGTTCCAGCAAACATATAACTACACCGGTGTCTCAGCCTTAATCATCTTTGTATTTGTTAGATTTATGAATATGTGGGTAGTAGTTAGTACTTGTGAACGGACTTCTACAGAGGCTGATGTTTTTACTGCAACAATATGCAACTTGTTAGTTGAAGATAAGACTGGAAGAATCGCTCAGAAAGAAATGTTCCTTCTACATATCTCGATGAAGAACACTTTGACATTTACAGCCTGCAGATGTTTTAATGTTAATTTCAAGTTAATCCAATCAATGATTGGTGCATCGGCCACACTTTTGGTAATATTAATACAGTTTGATATGACACAAAGTGATTTAGCGACAGAAGATCCATTTAATGTTACACTTGCTTCATATTAA

>GR26

ATGGCAGAGAGTGTGGTGCTCTATGTGAACTCCACGACCAAGCTGTGCAGATACTTGGGACTGTTTCCCTTCTATCTGTCCAATGAAGGCCAGCTGATTGTCTCCAAATTGTGGTCCACCTGGGCTGTCCTGGTGCTAACAGTGTTCACATGTTCGTCTACTTATTCACTGTTTGCCTTCGAAGTAAAGAGCACCAGTGCACTGTCCGTGATATTAAATTATTTTGAATTGTTTATATTTCTTATCCTAATTTGTGCTCATTTAATCTGGATATTTGTGAATAAAAAACTGTTGGAAGTTTCATTTCGTCCGCTGCTTACCCTAGAACTGGAGTTAATAAAACCTTCCCTCTGGCCCCCGTGCTTAGCTTACCTAGGAATATTTGGTTTAACTGTGATGCAATGTTTAATGTTCCCCTCTATCAAGCACATCTATGTCTCTATTGTTGACTACACACAAACTGCCTTTGCTTTTGCCGTTATCTTTCAGATTTATCGCCAAGCATTTATAAAACTGGCTAATTCGTGTAGAAAATGCGAAGAAGTAGGGAAACCTTTCCTTTTACGTCAATTTGAATTAAATGAAGAGGTGGTGGACTGTTGTGAGACATTAAGTTACTGTTTTGGTCCGGCATTACTGATCATGATACTCAGCTCCTTCATTATCATCAGTTCCAGCTTGTTCGCCGTCTACGATTTTTGGTCCGACAAGACTGAGGTCTCTGTGAGCTGTGTTTGGATAGTGCTTTACCTCTGTCAGTTCCGCCATCTCGTCAATGCGTGCGAGAACTCGAGCGCCGCGGCAAACCAGTTTTATTCCAGCCTATTGCAAGCTGTAACTAAAGATAACCAACTTTCAAACGATCATACCTTTGGTTTATTGTTAATGATGAGAAAAATATTGAAGTTGACTGCATTCGGATATTTCTTCATCGATTATGGTATATTCTGTTCTATGCTGGCAGCTTCAGTCACCTACTTCGTCGTGTTTATAGAATTTAATCACAATGCCAGTGAACGGAAAGCCGGACCCACGGATCTTATCTAG

>GR27

ATGACATTTAGTATTCAACGTTCTTTACAATCTGTGGTGAGGTGTTCCCAATTTACCGGCCTATTTCCTTTTAATATTGACGAAGATGGAAGTGTCAGAATATCCATCCCATTTTTTATTTTATCCATTTTAACACAGTCTACTTTAACATTAGTTAGTGTAAGTTTTTTTATTTTTAGATGGGCGTTCGCGTTTAAATTTAATTTTATCCATGAAATTTTAATATCGATGGGGCAATTCTCTATACAATATGCACAAATTTCATTAATAATTTCCTTGTTAACTAATAGAAAATTGTTGAATGAAACTTTTAAAAGTTTAGTACTCTTGGAAAATGTTGACGAACCAATCAACAACACCGGCTGTCTCTGGTATCCAACCTTCTGTGGAATTATACTAGTTGGTGCTCCTATTATGGAACTAGCTGCAGATATGTATCATTTCATCAGTATAGAATTATTTCTCATAATCTTGAAATGGGAACTTGCTTACTTTGCAATTTTTCAGTTCACAGGTTTGTTGGCAGTCTGTAGAACTTGTTTCATGGCTCTAGAAAATAGGCTGAAGAACCGATTAAGAGGATATAGTAAATGGGAAGCTGTGGAAAAATCAGATCAGTTGATAAAATGTTGTGAAACAATTAACTCATGTTACGGATCAATGTTGTTGATAATGTTAATAAGTTTTTTCGGCTTAATTACCACAAACTTGTATGCAGCCTATTTGAAATCTAACTACAAGTGGACTGTATTTATTTGTGTACTGAGTGCATTTATATTGCTGACTACATTTCATTACTTAGTTGAAGCTTGTACTAAAACAGCAGAAACGGTGAATAAGTTTAACCGGTACATCCGTAACTCAATAATTCAAGATATATTAACCGAATGTGCTGAAAATAGCACACTAACAATGGAGGTAGTTAATCGAAAAGATATGGAATTCACGGCTAATGGTTATTTTTCAATAAACTACTCACTGTTCTATTCGATGATTGAAACTTCAGCAACATACTTAATTATTTGTATACAATCATACACAATTGAATTCATCAGTGAAACAAAACCGTCATTTGAGAATTCAACAGGTTTTTTCAATGATGGTACTGATTTCCAGAATGATTCTTTCCTGGCGTAA

>GR28

ATGACTTCAAATGTTCTACGTTCGTTGCGATCATTATTCAGACTGTCCAGATATTTAGGAATATTTCCTTTTAGTATTGATGAAAATGGAAGTGTAAAATTTTCTATGTTGTCATTTGTTTTTGCTATATTTTTGCAATTGTTTCTGTTCTTGTTCTCAATGGGATACGCTCTGTTCAGATTGGTAATTTATCAAAAAATAAATTTTATTCATGAGGTTTTGATACAATTAGGACAGTTTGCTACACAAGCTGCACAGTTTGTACACATTATAACGTTGTCAATCAACAGTAAACGTTTGGATGAGACATTTAAAATGTTAATTCTTTTTGAAAATGTGGACCAACCAATAAATACGCATGAATGTTTTTGGTATCCAACTTTATGTTGCTTTTTACTTATTGGTGCTCCCATCATGGAATCAGCAGCAGAAATTTTCATCGTAGCTGATGCTGTGGAGTTTATTCTTATTATGTGGAAATGGGAATTAGCTTTCTGTGCCATTTTTCAGTTTACAGGGTTGCTGAGTATATGTCGAAATTGTTTTAAAGCCCTCAATAACAATCTAAACAACCGAGTAGAAGGATACAGTAAATGGGAAGCATTAGAAAAATCTGAACAATTAATAAAATGTTGTGAAACAATAAACAAATGTTATGGACCAATGTTGTTAATAATGTTGATCAGTTTCTTTGGCTTAATTACCACCAACCTTTATGTAGCCTATTTGCAATGGCTAAACAAATGGATTGTATTTATCGGCGTATTAAGCGCGACCATATTGTTCTGCACATTTTATTATCTGGTGAACGCATGTGCTACAACTGCTAAAATGGCAAACCAATTTAATAGAGATGTTCATAAATCACTACTCCATGACATTTTAAATGAATGTTTAACTAACGGCAGTTTAGCTGTAGAAGCAATCAATCGAAAAGAATTAGTATTCACCGCCTGCGATTATTTTGTCATCAACTACTCATTCATGTGTTCGATGATTGAAACCTCAGCCACATATTTATTAATTTGTGTCGAATTTGATGATGACGAAGAAGAAACGCAGGTGTACGATCAATCCATCAATAACTCTACTGAATATTACGATAATGTAACAACTGAATTTCTTAATAAGACTTTTTTTACATAA

>IR8A

ATGCAAATAAATATAGTGCTTTCGATAGCTGTGATTTTTTCAGAAAAATCCTTAGCTGGGAAAATATTAACTGTGAAAGAACAAGAAGATGTGACGTGGACAGGTTTAGGAGATGAGAGTATGGAAGAAGTAATCATAACAATTGACCGAAATGATGAATCAGATTCAACCAATAAAGTTTGTACAGCTCTGAAAGATGGAATATCAATTCTATTAGACCTTACTTGGACTGGATTAGATGAAGTTTTCCAAATTATTAATGGAACTGGATTACCATATATAAGAGCAGAGCCTGAAATTGTGCCTTTTTTAAGAACATTAGATAGAACTATAACAGATACTAGAGACGTCGTGGATGCAGCACTAATATTCCAAGATGCTCAGGGTTATGAACAAAGTTTACAATATCTTATACTCGAGTCATCACTACGAGTGGCATTATTTAATAATCTGGATAATGAAACTTCAACTAAATTAATAAATATGCGACCAACTCCATCATTTAATGCCATTTTTGGTGAAACAGAAGCAGCTGCTAGTATTTATAAAAAGGCTTTTGAAAACAAATTAATAAAACTGCACGATAGGTGGGCTCTAGTTTTAACGGACATGCATTATGAAAATTTAGATAACTCTTTATTTGAACACCGAACTAATATATATAATTTGAACAATAATCTGTGCTGTGAAGAGGAAAGCAATTGTACTTGTAGTAACGATTTCAATTTAAACAAGGAATATCTAGCACAACTTAAAATTGCCTTAAGAGAGGCAATTGCTAATGCTAAACTATCCGAATTGTCAATTACAGTAGACTGTGAAAATACAAATAACATTAAATCAGAGAATACATTATTTTATGACCAGTTAAATGAGCTGTCTGGAGGATATCATTTACTACAAATGGATCTTGCTGAAAATTTAGTTCGTCAAAGGATTATAATGGATTGGAAATTAATAACCAAACAACAACAACATCCAAAATTATTGGGAAAATGGGACTCAAAAACTGGATTAATAAAACATCGCAAAATTGAGCCCATCAAAAGATTTTTTCGTATTGCAACTGGTTATAGCATTCCTTTTGCCTATCCAGTGATAGATGGAAATACAAAAAAACTAATGTATAATGAAGATGGCAGTGAAAAATGGGAAGGATACTGTATTGACCTTATTGAAAGATTATCACAAGAAATGAATTTCGAGTACGAGCTATCAACATCTTATGATTATGGGAAAAAGTTAGAAAATGGAACCTGGAGTGGTCTTGTGGGACAATTAGCCACTGGGAAAATTGATATGATAGTTGGAGCACTAACTATGACTTCTGAAAGAGAAGAAGTTATTGACTTTGTTGCACCATATTATGAACAAACGGGATTTTCAATAGTCATTAGAAAACCTTTCCGTAAGACCTCACTTTTTAAATTTATGACTGTGCTAAGAGTTGAAGTATGGATCAGTATATTAGGAGCTCTTTGTTTAACTGCAATTATGATATGGCTACTGGAAAAATATTCTCCTTATAGTGCTAAAAATAACAAAGACAAATATTCTTATCCTTGCAGGGATTTCACTTTAAGAGAAAGTTTCTGGTTTGCAGTTACTTCTTTCACTCCTCAAGGAGGAGGAGAAGCACCCAAAGCTCTCTCTTCCAGGACTTTGGTAGCAGCTTATTGGCTGTTTGTAGTTTTGATGTTGGCAACATTTACGGCAAATCTGGCCGCATTTCTTACAGTGGAACGCATGCAGAATCCTGTACAATCTCTAAAACAACTTGCAAGGCAATCACGTATAAATTATACGGTTGTAAGAAATTCAGAAGCTCATGCTTATTTTAGAAATATGAAATTCGCTGAGGAAACTTTATACAGAGTATGGAAGGAAATAACACTCAATAGCAGTGCAGACCAATCACAATACAGAGTCTGGGATTATCCAATTAAAGAACAATATGCGCATATACTTATTGCAATGGAGAAAACGGGTACTTTAGAATCTGCGGAGGAAGGATTCCGAAAGGTGAGCGAAAATGAAGATGCAGAATTTGCAATGATCCATGATGCACTTGAAATTAAGTATCAAGTGTACAAAGATTGCAATCTAACTGAAATAGGAGAACCTTTTGCTGAACAACCATACGCTATTGCAGTCCAACAAGGTAGTCACTTAAATGAAGAAATAAGCAGGAGAATTCTGGATTTACAAAGTGACAGGTACTTCGAAGCATTATCTGCCAAATTCTGGAACGCTACTATGAAAAATAAATGTGACAGTTATGACGAAGATGAAGGCATTACGCTAGAAAGTTTAGGTGGTGTATTTATTGCAACTCTTGTAGGACTGCTGTTAGCTATGCTTACATTGGGTGCAGAAGTTTTATACGAGAAGAAGAAAAAATCTAAAAAAGTTAAAGCTAAGGAAGACATCAAGATTCCAAATATGTTAAGAGATGAATTTTTAACGGCTAAAGAATTTGGCCATGTTAGAACGAACAAAATTAAAGCAGTGTTCAATCCAGAACAAAAGCCCAAAGGCAATTATATTACAGTCTATCCTAGAGGGCAATTGTATTAA

>IR21A-FX

ATGATGAATATTAAGATTTTTATTACCATATTTTTCTTATCAACTAGTTTGGTAATGAGTGAGGCGCGCGGCGGCAGGGAAAATGTCACGATTGCCTCTGCAGCTCAATATCTCTTCAACAGAAATGTGATTGTAAGAAGGACATGCACGTCTGAATATGGAGTACCCTACTTGGTGCCATTGTTATCTGAAATAGTACAGACTTACATGTCAACCAATGTAGTAGTCTTGCTGTACGATGAGTACTTCTATTTCAGTTCTTCATTCCAGTTAATATTGGACAATCTGCTGAAAATGAGTATTTCCTTACGTCATGGTAAATTGGATACGAGTTTAGCAACACCCCGAGCCCCGCCTGGCTTCATGCAGGTCAACAATAATGAACGTTTGGCTTTTATCGTATTTTCAAAGGATATAGATGTGAGCGGTGAAGCAGTCGTTGGAAATGTTTTAAGCGATACAAAAGTTGTACTGATTTCACAGTCATCAAGCTATCAAGTCAACCAGTATCTATCTAGTAAATCGGCCCGAAGAATTGTCAATCTTCTAGTCATAGTTGATATGACATTGAAAGTGGATCTTTACGCACAGCGCAAGCGCAATAAAATTGGAGAGTGCGATATTATGCTATACACTCATAAAATGTACGCAGATGCCTTGGGTAACAGCCACCCAATGATCATTACAGCTTGGCGTCGTGACAGTTTTACTCGGACAAATATCACACTGTTTCCAGACAAATTCAAGGGCGGTCTCAACGGGCTGCATTTGATTGTTTCTGCTTCAAATAAACCACCTTTTGTATTCAGAAAAAGGTGCCCGGATGTCTCAGAGCCTTCACAACTAACGACAGAGACTGGACTAGAAGTTAGGCTTATAAAACTTCTAGGTAAAATAATGAATTTTACTGTTGAATTTAAAGAGCCATCTTTTGAAGCCAATGATGGGGAAGACACAGCTGATGTAGTATTAAATGAACTTTTAGAAGGTAGGGTACAAGTAGCACTAGGTGGTATGTATATGACATCATCAAGGTATCAACTATTTTCTTTTATCTTCCCTCACATCCAAGATTGTGCTTCATTTATCTCTCTGACGTCAACAGCTCTACCAAAATATCGAGCCATAATGGGACCATTCCTTTGGGATGTTTGGCTGGCGCTCACCATTGTCTACCTTTTGGCAATAGTACCCATTTCATTCTCGGTGTGGCATACTTTTACACCAATTACAAATGATTTCAAGGAATTAGAGAATATGTTCTGGTATGTGTTTGGCACATTCACCAATTGTTTCTCTTTTAGTGGAACCAATTCTTGGTCAAGGGGTGATAAAACAGCGACTCGATTTTTTATTGGAACATATTGGGTATTTACAATTATCATCACCGCTTGCTACACTGGCTCTATAATCGCGTTCATAACGCTACCAGTATTTCCTGAAGTTATTGATACGACAAGGCAATTTGTTGAGGAAAAGTATAAACTTAGCACAATCAGAAATGGAATTTGGCATTCTTTGGTGAATGAAAGTTCTGATCCCGTAGCTTTGCAACTTTTACCAGACATAGATTTAGTATCAGATTACATTGAGGGTTTGAGGAATGTCTCAGAAAGTGTTAAACGTCACCGTAAGTCAGTATTTCTCGGCTCTAAACACCTCCTGGAGTACGTCGTCAGAACTAACTATACACCTGAAGATACAAGCAAGCGTCTGTTATTCCATATCAGCAAAGAATGTTTTGTTCCTTTGATGGTAAGTCTTGCATTTCCTCCTGGATCGTATTTCATAGAACACATGAATGTCGCTTTAGAGAATTTAATTCAAGCTGGCTTTTTGGCGAAAATTATCAGAGAAGTAGACTGGATCATTTATAGATCATCTACTGGGAACTTGTTGCAGGAAAGATCAACTGGCTTGAGAACTCCACCGGAAGACAGAGAATTAACATTGGATGATACCCAGGGAATGTTTCTTTTACTTGGTGCCGGATTTGCCATAGCTGTGGCCGCTTTAACTGGAGAGATAGTCGCCTGCGTCTGGAAAGAAACCAGAGCTCCACCCGAGTTTGGCAAACCAACGCTAGGAGAAAGAATTTTGGAGAGGATATACAAGTGGGCATATAAATGCAAAGGATGTCTACTATCACCAATAGAAGTTCAAGAAAGGCTCAGTCGACTTTTTCTTGACAGAAGAGAATCGACAGAGAACAGAAACTTATCAGAACTATTTGGCAGTGAAGTGGATAGAGAGATGAATAATGATCGTATTTATAATAATCGTATCGAAATTATGTCTACAATTCATTTACAAGCACCTAGAACTGAACAACCAATTAGGCTTCGAAGTTTTTAA

>IR25A

ATGTATGTTTTTCTTCTGCTAATTATTTGGATGAAACTAAGATTCCTGTCCAGTCAGTCAACACAATCCATAAATGTCATGTTCATCAATGAGAAACACAACACTATAGCAGAGAAAAGTTATACCGCTGTGATGGATTACATCAGAAGAAACAGAAAACTGGGAGTACATATAGATGGAAATGTAAATGTAGTAGTAGCAGGAGATGATGCTAAAGCGATATTAGAACAATTATGCGAAGTATTAAATTCATCAGTAACAGCTGGAAAGGCTCCATATTTAGTCCTAGATACCTCACTGACTGGAGTACCGTCAGAAAGTGTAAAAACTTTTTCTGCAGCCTTGGCATTACCAACAGTAAGCGCGTCGTTTGGGCAAGAAGGTGATATTAAACAATGGCGTAATATAGATGGCGAAAAATTAAAATTTCTAATACAGATAAGTCCACCGGCAGACATAATTCCTGAAATAATTAGAAATATAGTTACTATGCAGAATATTACAGGGGCAGGAATACTTTTTGATGATTCCTTTGTAATGGATCACAAATATAAATCATTACTACTAAATGTAGCTGCTAGACATATTATAGCACATGTAGGAAACGATAGAGAAATCAGAACGCAATTGCTTAGATTTCGAGAGTTAGACATTGTGAATTTTTTTGTACTGGGACGATTAACAATGATAAAAGCAGTATTAGATTTAGCAAACAACAAATACTTTGGAAGAAAGTATGCTTGGTATGCAATCACTCAGGACAAAGGCCAACTAAAATGTTCATGTACAAACGCAACAATACTTCATGTAAAACCTGAACCAGAAGCAGCAAGTCGTGAGAGATTGGACAATCTAAGAACAGCTTTCAACCTTGTAGAAGAGCCAGAAATCACATCAGCCTTTTATTTTGATTTCTTTCTACATGCAATACTTGCTATAAAGGGTCTCACTGAAAATGAATTACTACCAAAAGATTTTAACCGAACAACTTGTGACGATTACAAAGAATATCAGGAAATTATTAGAAAAGATTTAGATCTCAGGAAATATCTAAAAGCGGTAACAGAGCCATTTTCGTACGCTCCTTTTATTATTGGAGCAAATGGGCACAGCTATCCTGAATTTATAATGAAACTTGAAAAGGTAAGCATAGTGAACTCCCAATCAGAAAGTGTTGAATCTTTAGGAACATGGAAAGCAGGACTATCATCTCATATTTCTTGCAAAGATCCAGTTGCAATGTCAAATATGTCTGCAGTGACTATATACAGAGTTGTTACAGTTAAGCAACATCCATTTGTTATTGAATATCAAGATGGAGGAGAAACCAAGTTTAAAGGATATTGTATTGATCTTATTGAAGAAATCAGATCTTTTGTTAATTTTGAATATGAAATATATGTAGCACCAGACAATAGTTTTGGAGATATGGATGAAAATGGTAACTGGAACGGAATGATAAAAGAACTTATTGAAAAGAGAGCCGAAATAGCCTTAGGCTCACTTTCAGTGATGGCTGAAAGAGAGAATGTTGTAGATTTCACTGTGCCCTACTATGATTTAGTAGGAATAACTATTCTCATGAAAAAGCAAATAACGCCAACATCTTTATTCAAATTTTTAACCGTTCTGGAAAATGATGTTTGGCTTTGTATTCTAGCAGCATATTTTTTTACTAGTTTTCTAATGTGGATATTTGATCGTTGGTCACCTTACAGTTATCAAAATAATAGAGAAAAATATAAAGATGATGAAGAAAAACGAGAATTCAATTTAAAAGAATGTTTATGGTTTTGTATGACTTCACTTACACCTCAAGGTGGTGGTGAAGCTCCAAAAAATTTGTCTGGGCGATTAGTGGCAGCGACATGGTGGCTTTTTGGTTTTATAATTATTGCATCGTATACAGCCAATTTGGCTGCGTTTTTAACAGTTTCTCGACTGGACACGCCTATAGAATCATTAGACGATTTGGCCAAACAATACAAGATACAATATGCGCCAATTAATGGATCTTCATCAATGACTTATTTTAAAAGAATGGCTGATATTGAGGAAAGATTCTATGAAATATGGAAAGATATGAGTTTAAATGATAGTCTGAATCCAGTGGAGAGAGCTAAGCTGGCTGTGTGGGATTATCCAGTTAGCGATAAGTACACTAAGATGTGGCAGGCAATGAAGGAAGCCCAACCACCTGCTACTCTTGAGCAAGCTCTTAAGAGAGTAATGGACTCAAAAAGTTCTACAGAGGGTTTTGCTTTCCTTGGTGATGCAACAGATATCAAGTACCAAGTATTAACAAACTGTGAGTTTCAAATGGTAGGAGACGAATTTTCAAGAAAACCATATGCTATAGCAGTACAACAGGGTTCACCGTTAAAAGATCAGTTTAATAATGCAATATTACAATTACTAAATAAACGAAAATTGGAAAAATTAAAAGAAAAGTGGTGGACTGAAAATCCTGAACGTAAAAAGTGTAATAAACAAGAAGAACAATCAGATGGAATTAGTATCCACAATATAGGAGGTGTATTTATTGTAATATTTGTGGGAATCGGACTGGCATGTATAACACTTGGTTTAGAATATTGGTGGTATAAATTCAAGAAACCTACGACAAATCAGCCCCCACCAGCTGCTGGAATAGGGCTGAAGGCTTTGACAAATGAACAACATAGACAAGCTGCGCTCGGAAAATTAAGTGTTACTGGATTGTCTGATTATACCAATCAACAATTCCGAGCTAGAAATCATATCAGGTAA

>IR40A-FX

ATGGAATGCAGTGCACTGTCTAAAATCTTATCTACACTACCCACATCAAATGCAGCGCTTATTGTAGACGACGAGCAAGACGAACAGTGGTTGAACTTTGTAAGCGACAATCTGTTCAAATCTGGCATATTAACGGTTCTCTACAGACTTCATTCGGATTCCACAAATAACTCTTACTTACAGGCGTTCACAGATGATATCAAACTGAGGACATCCGGATTTTTGGATACAGTCTATGTATTCAACGTGGACACAAACGCAGCTGAAACTATTTTACAACTGGTTATGACTGAGAATGTAGCCCGAAGGAACATCTTGCTGGTGTTCCTATGGCAGGACCTGTTTGTGTCGCGGCGCTTTCGCGATTTCCTCCAAGAAGGCATGAGAGTGTGCGTCGTTATCAACAATCGTTATGGAGTATTCCAGGTACTATACAACCAGGCAAGACCAGACGGTGGAAAAGGACTTGACCTAGTCAACTGGTGGTCTCATACCACGGAGAGTATGTTCCACTCACCTCTGCTGCCTCCAGCAATCAAGACTTACCAGAACTTCAATGGTCGAATATTTAAGGTTCCAGTATTACATAAACCACCCTGGAACTTCGTGATCTACAGTAACATCACTAACAAGATTTTAGTGCAAGGAGGACGTGATGACAAACTGCTTCGCTTGTTGTCCAACAAGCTGAACTTCAAGTACGAGTATTTCGACCCTCCAGATCGAAGTCAAGGCTCGGCCATAGTCAATGGAACCATGCAGGGTGTACTAGGTCTCATCTGGCAAAGAGATGTAGAAATGTTCATAGGAGATTTAACTGTAACATACGAACGCAGTTTGGCTGTGGAATTTTCGTTTCTGACACTCACTGACAGCGAAGTATTTCTCACACACTCACCAGGCCGGCTGAACGAAGCACTGGCTCTGATAAGACCATTTCAGTGGCAGGTGTGGCCAGCCGTGGTGACCACAGTTCTGCTTGCCGGCCCGCTGTTATACGCTCTGGTATGGTCTGCTAATGGATGGACTTCAACTAGGTCAAATCTATGCGACTGCATTTGGATCACCACCACGATATTTCTAAGACAGATACCACAGGCATACACACCGAGCAGGGTGCGCTTGTTGATGGTCACCTTATACCTGGTGTCCACCTACGTTATTGGAGATATGTACTCTGCCAATCTAACTTCAATGCTAGCCCGTCCGGCAAGAGAGAAACCTATCAGTAACCTGGAGCAGCTCTACCAAGTGATGAAGTATAGCGATTTCCAACTGCTAGTGGAGAAACACAGTGCCAGTCACGCAGTGCTCGAGAATGGAACCGGATTGTATAAGAGTATTTGGGAGAAGATGAAGTCGCAGCCAATCTACCTGATTGAATCCACAGAGGAGGGCATGCTCTTAGTTCGAGAACAGAAGAATGTTGCACTAATTGGAGGCCGAGAAACTTTTCTCTATGAAACCAGAAGATTTGGGGCACATCACTTCCATTTGAGTGAGAAATTGTACACAAGATATTCTGCGATTGCACTGCAGATTGGATGCCCTTTTGTGCATAATTTTAACGACATATTGATGCGGCTATTTGAAGCTGGTGTGCTGACCAAAATGACACAGGAGGAATACCGCAAGCTGGAGGATACTCGTCCTAGTAATGAGGAGAAGAAGGACGTTGGGGAAGGACGCGGTCAGGCTGAGTCCGAGGATACTCATCGCATGGCTGCCTCCATGGAGATGCTACAAGGAGCGTTCTTCTTATTGCTAGCTGGATACTTGGTTGCAGGTCTGATCCTGTTTGCTGAATGGTGTTCCATTGGGAAGTTCAAGCCTGTCAAACTGAACAGCATAGAAAAGAAACCAGATCTATATAAAAAATCAGTATGTAATGGATTGTAA

>IR41A-FX

ATGAATAACATGCCTAAATTAATCTTTTCCTTCACTATTTTAAGTTATGTTATTGGTTCGATTTCTGTAAAAGTTGCCACCTGCTTTATTTTACCAAATGAAGAAGATAATAATTGCAAAAATATCGAGATACTTACAAAACAAGTACTTTCTACCTACTACCTGTCTCATAAATGTACTGTTCTTATTACAATGATGGAAATGTTTCATGAATTCAACACAGATTTAGGAACCAGCTTTATTCAGATAGCTCCTCAAGAGAAGTGTGATTACGGATTAGCCAATAAAATTGCAGAGGGCTTTAAACAAAACTGTGACAGCTATATTGTACAAGTAGACGATCCTTTGTGTTTTCTTAAAATATGGCACATATCACTTCAAATGACTTTTCACAGGCACAACCCTATTTTTTTCTTTTTACCTGATTGCAAAAACAGACGAAACCATGCTATGGAAATACTTTCTGCTCCAGAATCAGATATTACAGTCAATATCATTGTAGTGGAGTTAGAATTCAACGATAATAATAAGTTTAAGCTAAATCATTCTGAAGACTGGCCAGTAACATTGTGGACGAATGATTTTAGCAAACTTGCTGGTTCACCTGAGCGAACAAAAATATTTGTAGATAGCTGGCATCCAATAAAGGGTTTCCGTTTCAACAATAATCTATTTTATGATAAAATTTTAAACCTTAAGGGAAGAGTGTTGAGAGTGGCTGCATTTACATATCCTCCTTACACTATAGTAGAAAAGAATACAATTGACGGAATAGAAATGCGCATTGCTTTTTTATTTTGCCGTTTGTACAATTGTACTATTGAAACTGTATATGATGATGGATTATGGGGAACTATACTAGGAAACGGTTCTGGTGATGGAATATTGGGGATGGTGTACAAGAACAAAGCTGATATAGGATTTGGCGGAATCTACCTCTGGCTAGAACCAGCTTACTACTTGGATTACAGTGACACTTGGCTTTATGCTGCAGCAACTCTGCTTGTTCCGAAACCGGTCCCTTTAGGGGGATGGAGAGTTCCGTTCCTACCGTTCGATCTGGCAATGTGGACAGCAGTTTTCTTATCGCTCGCAATATCTTCTTTTTGTATTTCTTTTGCTTCCTGGTTAATCAACAGAAAACGTTACAATGTGGACGTCTATAAATTACTAGGTATCAATGATACTATTTTAATGATTTTGGGAATGTCTGTACTACAACCTCCATCAAGACAATTAAGTGACAACCATGTATCTATAAATCAGATGGTTACCTCTGTAGCAATTATGTGTTTATTGCTAACCTCCTGCTACTCGGCTGGGCTTTCTTCTTACTTGACCGTTCCTCAATTCACTAATCCAATAGATACATTCGATGAACTGTCTGCAAGCGGACTATATTGGTTAGCCATTCATGAGGCCTGGGTATATTCTTTGCAACAAGTTGAAGATGAAAAAGTTCAAGCTGTAGTAAAACATTTCAGGAAATTGTCAGAAGAACGTCTGCTGTCAGAAGCTCCAAAAGGAAAATATGGATTGGCTGTAGAACGACTGCCAGCTGGACACTTTGCTGAACAGAGTCATTTAACTGATGATGTTATCAGTAACTCTCACATAATGAAAGAAAATCTATTTGGATCTCTGCTCACTACAGCTCTGCGTAAAGGATCCCCATTTACCTACCATTTAAACAAGTTAATGAATGTTCTTCTTAATTCTGGGATAATGTATTATTGGGAAGGGGCAGTGTCCAGAGATTTTTTAAATACCAGAAAACAAATGGCTCTAATGGAGGCCAGAAATGCTCGCAGTAAAACTGAGCCTACCAATCTTCAGCTGTCTCATGTACAAGGAGCGTTTATTATTTATGGAATAGGTGTTCTACTAGCAAGTTGTACTATTGTGATAGAATTATATAGATCTAAAAAATACAAAACATCAACCAAAATTAGAATACATCCTTATACGTTCTAG

>IR41B

ATGGGTCAAGTTATTTATGTATTTATTATGCTGACTAATGCATTGATTTCCATAAAAGTTGCAGCCTATTTTATTTTACCTAATAAAGAAGAGGATAATTGCAAAAATATGGAGATACTTACGAAACAAGTAATTACTACCTACTACTTGTCTCATAAATGTATCGCTCTAATTACAACTAAGGAAATGTTTCAAGATTTTAATACAGATCTAGGAACCAGTTTTATGCAGATAACTCCTCAAGAGAAGTGTGATCAAGGATTGGTGGATATTATTGCTGAGGGCTTTAAACAAAACTGCGACGGTTACATTGTACAAGTAGAAGACCCTTTGTGTTTTCTTAAAATATGGCATACATCACTTCAAATGACCTTCCACAGGCTTAATCCCAAATTTTTCTTTTTACCCGATTGTAAAAACAAACGAAATCATGCTATGGAAATACTTTCTGCTCAAGAATCAGATATTACAGTAAATATCGTTGTAGCTGAAATAGACTTCAACGACACTAATAAGTCTAAGTTTGGTCTAAATCATCATGAAGACTGGCCAATAACATTATGGACGAATGACTTTAGCAAGCTCGCTAGTTCACCAAGGCGAACAAAAATATTTGTAGACAGCTGGCATCCAATAAAGGGTTTTCGTTTCAACAATAATATATTTTATGATAAAATTTTGAACCTTGAGGGAAGAGCGATGAGAGTGGCTACATTTACATATTCTCCTTACACCATAGTAGAAAAGGGATTCATCGAAGGTATAGAAATGCGAGTAGTCTTGTTATTTTGCCGTTTGTACAATTGCACAATACAAATTGTATATGATGACGCATTATGGGGAACTATACAAGGAAACGGTTCTGGAGATGGTATATTAGGAATGGTATACAAGGATGAAGCTGACGTGGGATTTGGCGCAATTTACCTTTGGATAGATCCAGCTAGCTACTTGGACTACAGTGCTACATGGCTTTATGCTGCGGTAACTCTGCTTGTTCCAAAGCCGGTCCCTTTAAGCGGATGGAGGGTTCCATTCCTACCGTTCGATCTATTCATGTGGAGTGCTATTTTCTTATCGCTCGCAATTTCATCCATTTTTATTTATTCTGCTTCCTGGATAAGCAGCGGAAGAAATCATAATGCGAATGGGCGTGAATTAGAAGGTATCAAAGATATTACTTTAATGGTCTTGGGAATGTATGTACAGCAACCTCCATCAAAAAATTTAGAAGACAATCACGTCTCTATAAATCAGATAGTTACTTCTGTGGCAATATCGTGTGTACTGCTAACCTCCTGCTACTCAGCAGGACTTTCTTCTCATTTGACTGTACCACAATTCACTAAACCAATAGATACATTTGATGAACTGTCCACAAGTGGACTATATTGGTTAGCCATTCACGAGGCCTTTGTATATTCTTTGCTACCGGTTGAAGATGAAAAAGTTCAAACTGTGGCAAAACATTTTAAAAAATTGCCAGAAAAACGTCTGCTAGCTGAAGCACCAAAACAAAAATATGGTTTAGCTTTAGAGCGACTGCCAGCTGGTCACTTTGCTGAACAGAGTCATTTTACTGATGAAGTAATTAGTAATTCTCATGTCATGAAAGAAAATCTTTTTGGATCTCTGCTCATTTTCGCTCTGCGTAAAGGTTCCCCATTTACCTACCGGTTAAACAAATTAGTGAACATACTTCTTAGTTCAGGAATAATGTACTATTGGGAAGGTGCGGTGTCCAGAAAGTTCTTAAACACCAGGAAGCAAATGGCTCTAATGGAGGCAAGAAATGCTCATAGTGAAACTGGACCAATTATCCTCAAGCTGTCTCACGTACAAGGAGCGTTTATTATTTACGGAATAGGATTGTTATTGGCAATTTGCACTATTGCAGTGGAAATATTTCTATTTAAGAAATACAACACACCAATCACAATTAGAATATATCCGTATACGTACTAG

>IR41C

ATGAATTTAATTTTAACAGGAATATTTTTTCTCATTTTCATGAAATATGTCTTATCTTTTAATAATGAGGAAGAGTTAGAAATTTTTCATAAAAATTTGGAAGAATTAACAAATCAAATTCTAAATAAATATTTCTTAGATAAACACTGCATACTATTGGCTACAGAACGTGAACTCTTTAATACATTTTTTACAGATTTGAACGTAACTTTGATACATGTCTCACCAAAAAAGAAAAATTTCTGCGACCGTATTATGGTGGCCTCAATCGCACAAGGTTTGAAACAGAAATGCGACGGTTATATAATACAATTATCAGACCCCAAGTGCTTTCTAAGATTGTGGCTCAAAGCACTGCATACAGTGGTTAATAAGCATATTCCGAAGTTCATATTTCTTCCTAGTAGTCCGAAACACAATGACTATGTGGACGAGCTTTTCTCCATGGAAGAATCCGATGTGAGTGCCTACATCATCGCGGCAGAAGTGGACATCAATAGTAATGACTTGCCAGTGATACTATGGACAAACGACTATTCACTACCAGCTGACACTCCAGGAAGGGAGAAAATATTCCTAGATAAGTGGACCTTAAAAGAAGGCTTTCATTATAGAAATGACCTTTTCTATGATAAGATACTTGATCTGAAAGGAAGAGTAATAAAAATGATGAGTTTCACGTATCCAACGTATAGCGTAATCGAGTATGATGGCGATACGCTGACACTGGATGGTACTGAAGGTCGTATGCTGACGACCTTCTGTGAGATTTATAATTGTTCATTCGAAGTGTATGATGATGGAAATTTTTGGGGCACTCTAAATTCTACAAATGGAACTGCAGAAGGTGTAATAGGTTCAGTTTTCACGAGGAAAGTAGAAGTGGGATACGTTGGGATCTACCAATGGCCAGAAGTTAATTTTCACATAGATTATTCAGCATCCTGGCTGTACAGCACGCTCAACCTACTTGTGCCGAAGCCTGTACCAATAAGTGCTTGGAAAACTCCTATTTTACCATTTGATTGGCAAATCTGGCTAGCCACATTTGTCTCTTGGATATTTGGTACTATTGCATTCTTTACTGTTTCAACATGGGCAATACAAATGCATGGTCAAATTTTGAGCATTGCCAAGATTGGGAAACTGAAAAGATTCTCTATGACAACGATGGCTGTTCTGGGAGTTCTTGTACTGCAGTCTCCGCCACGTTACTTACTGCCCAAAATATCATCGCTTAGAATTGTCTTCATCTGTACCGAACTGTTAGCACTGATATTAACCTCTTGTTATGCAGCTGAATTGGCTTCATATTTAACAGTGCCGCAATTTTCTAAACCAGTCGATACGAGGAGAGAATTTGTTGACAGTAATCTGATATGGATTGCCCCACATGAAGTCTATACTCTTTCACTGAAAGATACTGGAGACCCAATAGCTGATAAATTAGTCGCTAGCTGTAAGGTATACAAAGATATTGACGAGTTAACTGAAAAAGCGGAAACTGGCAAATTTGGAGTCGCAATTGAAGTACTACCTGGAGGTGCAGTTGCAGATGTTCCACCTTTGACGGAGAAAGTTATTACCAAATCACATTTTATGAAAGAAAGTCTGTATGGATCGCCTTGTGCATTAATGTTTAATAAAGCTTCTCCTTATGTCCACCACTTTAATAACATGCTTCACAGACTGTTGGACGCTGGAATAATAACTTTCTGGGAAGAAGACGTTGCACGAAAGTACATGTCAGTCAGGAAGCAAATGGCCATAAAGGAAAGTAAAGTAAAACGTTCAGAAAATTTAGTGAAGAAACTAACTATCGACCATGTACAGGGAACATTTTTAATTTACACAAGTGGAATATTGTGTGCCCTGATTACATTTCTTTTTGAAATCTATTATTTTAAGAAAAAGCAAAATCAAATGCATGAATGGGTTTTCTAA

>IR68A-FX

ATGTTAACTCATTTAGCAAGAAAATTAAGTAAAAAAGTGAACTGTCTAACGTTAATAACTGATTCTTATTATAATTTCGTATTTAGTAATGAAATGTTTCACACGTTTCATGGGATACCCTATTTTAAAGTCCTAGTTAAAGATTACGAAGACTTATTAGCACCAAATTTTCAAACATTAACCGTAATACAACAATCACAATTGAAAGGATGTAATGCTTATATTATTTTAATAGCAAATGCTGATCAAACTGCAAGATTTCTTAGATTTGGAGACAGACATAGAGTGCTGGATACTAGAGCCATGTATATTGTTATCTATGATCCTAGACTGTTCCATAGGGAACTCTACTATTTATGGAAAAAAGTTGTAAATGTAATATTCATTAGAAAATTTCAAGAAGTTCCTAGATATGAATTTTCAACCGTTCCTTTTCCAGCTTCAATTAATTCTAAATTTGTTCCACAAAGAATTGACACATGGAGAGGTGGTAAATTTCAGAAAGATAAAGAATTATTTACGGACAAGACTTATAATCTGAAATATAACACGCTTAGAATAGCAGTATTTGATCATATGCCGTCTGTGGTGAGAATCGCAACACCTCACAGTGATGAAAATTCGCAATTAGCAAATTCACCAGCTAAATTTGGAGGACTTGAAGTCCAAATTTTGCAAAGTTTATCAAAAGCAATGAACTTCCAAACAGTACTTTATGAACCAATGAATGCAGATGTTGAAGCCTGGGGGAGAAAACAATTGAATGGTACATTTTCTGGAATTCTGGGAGAAATTACCACAGGAAAATGTGATATGGCTTTAGGGAATTTCCTGTACACACCTTACAATCTAAAGTTGATAGATCTAAGTAGACCATATATAACGCAGTGTTTTACATTTTTAACGCCAGAATCTACCACAGATAATTCGTGGAAAACTTTGATATTACCATTTAAGCTCTACATGTGGATTGCAATCATTCTCACGCTTATCACTTGTTCCTTCATCTTTTATGGTCTTGCTAAATTCCATACATCATTAAATCAATATCCAACACAAAAACAAAAAGCTGATCGTGCATTCTGGAAAGATTTACTAAGACTATGGAAAGATGGAAAGTCTAAAAATACTATGCAAATAATGAGCGTAAACAATTTGACAAATCATGGTGAAGAAAATGGTCCAAAAGGATTGTATTTATTTCAGAAAATGGAGGACAGTCTGATGAATGCTTACAGTATGATGTTGTTAGTGTCTTTACCAAAAATGCCAACTGGATGGTCACTAAGAACGTTGACTGGATGGTGGTGGATCTACTGCCTGTTGGTTACTGTTGCTTATCGGGCTAGTATGACGGCAATTCTGGCGCATCCAGCGCCGAAAATAACCATAGACACACTCGAAGAACTGGCTACCAGTTCGATTGGTTGTGGAGGATGGGGCGAAGAAAATAAGAAATTCTTCACTACGTCGTTAGATGTCGCTGGACAACGAGTTGGTAAAAAATTTGATGTAGTATACGATGCAGACGAAGCGATAATGAAAGTAGCGGAAGGAGGCTACGCATATTATGAAAATATTTTCTATCTGCTTCACGCGAAGGTTAAACAAAAATTGAAATTAATTGAAACAATTGTAAAAGATCTTCATATAATGGGCGACTGTGTTATAAACATGCCAATATCATTGGGTTTGCAAAAGAATTCTCCATTAAAGCCAAGAGTTGATGTTTTCCTACAAAGAGTCATTGAAGCAGGCTTGGTAAAAAAATGGTTGGGCGATGTGATGGTGAAAACTACAGCTGCTGAAGCACCATTAAATAAAGCTGAAATTAAAGCTTTGATGAGTTTGAAGAAATTTTCAGGTGCTCTGGTGGCTTTACTTTCTGGTTATGTTATTGGAATTTTGGTATTGATTGGTGAAAAGATATATTGGTATTATTTTATTATGAGAGATCCAATGTATGATAAATATTCTAAAACATTGAATTTTGGTAAAATTACTTAA

>IR75A-FX

ATGATGTTATATAAGTCTTATCTGCTTTCAACGCTGTTTTTCTATTCAGTAGCAGCTGAATTCCCGTTGAATCTTATTTCAAATTATTTTAACCAGCTAGAAATCAATGTGGTCACTATACTTTCTTGCACTTCACTAGAAGACAAAGTAACCATATTAAAATATTTAAACAGTAGAGGTTTTCAAGTATCTTATTCCTGGTATTCAATAGAAAGAATTCCGTTACATAGAAGAGGTGCTGTACTTGATCTGGATTGTCCAGGCAGAAACAAGATATTATGGAAGATAAACAAGGAAAAAGCATTTGGAATGCATACTGAATGGCTACTGTTGAATAGAGTACAAAAAGAATCGGATCAACACTTAAACGGTCAAATATTTATGAGCGAAGCAGTGATTTCAGTAGCTGAAAGTTATGCTTTACCAGGAAGTTGTGTTAAACTGTTGCAGCTAATAGATGATGTGGAAAAATTGTATTATTTTGACATGTATAGAGTAACAATTAGAGAACCTATGCAATTCCAACTAGATTGGTCTGGTTCGAAAAAATTACCTCTTCCAGAAATAGTACCTAGATGTGAATACAGAAAAAGATCTAACTTTGGAGGAATAACAATGAAAAGTGCTTCTATAATCAAGTTTCCAGACTTTTTTAAGGGATTTGAAAGTCTGGACTACCGCGAGGTAGATACATGGGCTAAAATGCATTTTCCTATGATGGTATTACTTACGGAACAACTCAATTTTATTCAGAACATAACTATTACTGATTTGTACGGTTGGGAAACAAATGGTTCTTTTGATGGTCTAATGGGTCTCCTTCAACGAGAAGAAATCGACTTTGGTGCTACAGGTTTTTTTATGCGCAAAGATCGTATGAAAGTATCAGATTTTTCAGCCGAAACTTTTTATGTAAGGACAGCCATAACTTTCAAACAACCAACATTATCTAGTGTCAGTAACATTTTTGTGCTACCATTTAGCAAGTTAGTCTGGATTGCTGGTGCAAGCCTTACACTCCTTGTAGCGGTAATTCTCGCTAGCGAATATTTCTTGACTAAGAAAATATCTATTTATCCTTATAAGGAGAAGCATGCCGCTACAGCCGATCTCATAACGATGGTTATGTCAACAGTTTGTCAACAAGGTACGGAATTAAATCCTATATCGCTACCATCTCGAATTACCGTATTCTTATTTTCATTATTTGCATTCTTCTTGTACACTTCATATTCGGCCAACATTGTTGCATTGCTTCAGTCTTCGGCTCCTGTTATTAAAACTATCAATGATCTTACAAAGAGTCACTTAAAATTTAAAGTTCAAAACGATATCTACAATGAAATTTATTTCAAGGAAGCAGTTGATAAAGCTATAATAGATCTCTATAAAGTTAAAGTAAAACCTCAAGGTAAAGAGGCTTACTGTAGTCCAGATATTGGTGTTAAATACATGAGAACTGGAACATATGCTTATGATGGGGAAACGAATTTAATCTATAAGTTTATTAGTGACACTTTTGAGGAAGACGAGAAATGTAGTCTAAGTGAAATGGACTTATTTTATTTACCTGCCCTGGCAGTGCCTGTAGTCAAAAGATCTGGGCATAGAGAACACCTGACTAGAATGATGTCCTGGCAACGAGAAGTTGGCATGTTTTCTAGAATAAAAAGTGTTTGGCTAGCACCTAAACCACAGTGTGGTTCAAACGGAAGTGGTTTCGTTAATGTAGGACTTGCAGATTTTTTACCAGCTTTATTGGTGTTTGTTTATGGAGCAGCCGTAACGACTGGTTTGTTCATTTTGGAAATTCTTTTTCATTACAGGAACAAGTTACGCTGGCGGAACAGTATATGTGGCGGAAAGATTGTAGTAACTGACCTTAGGTCCATATAA

>IR75B

ATGTCAATCATTGCTACATCAACTAAACATGGCTGGATAATTTGGTTTTATTTTGTCGGTATTGTAAAATGTGCAAATGTGGATTTAGTGGATCAACAACGACAAATAATATTTAATTATTTTGAATTGACCAATATTGAAACTATAAATTTGATTACTTGCTGGAATATTCGAGAAAAATATGCAATGCTTCAGGAATCATCTGAAAAGGGTTTAAGAGTGTCATTTTCTTTTGAATCCATGAAACCATGGCCTGAATTTCGACGTGGCGGTGTTCTAGATCTGGGCTGTCCATTTACTGAATATTATTTAACTAGGATTTCAGCAGAAAAGATGTTTGGAATTCATACAGAATGGCTGTTGATAGATGTTACAGATAATACTGACGATAACCATGTAGAAGGCCAGATATACGAAAGTAATGGAGTGTTACGAGCAAGTAACAGTTATTCACTGCCTGGAAGCTGTGTAAGATTGGTGCAAATAATGACTAATTCTAAAATGGCCTTTTACTTTAACATGTACAGAGTAACTATGAGAGAACCAATGCAATTCCAATTAGACTGGTCAGGTTCTATTGACGATCTGCCGTCTTTATTGACACCATGTTCTAACTACAATAGATCGAATTTTGGAGGAGCTACTCTAAATGCGGCTGTGGTAGTTGAATTTCCACATTTATTTGAAGGATTTGATAGTATGTTACACATGGACGTGGACATCTGGCCAAAATCACATTATCCAATTACAATACTATTTGCTGAACAGCTTAATTTTCGAATTAACTTTACAGTAGTACCAAGTTTTGGATGGTTGGTAAATGGTACTGACTTTGATGGAATGATTGGAATGTTGCAAAGGGAAGAAGCGGAAATCGGTGCCACTGGTTCATTCATGCGTGGAGATCGTATGAATGCTGCACAATATACTACGGATACTTTTGAACCTAGATCGGCGGTACTTTTCAAACAGCCACCTTTATCTAGTGCGTATAACATATTTCTGTTACCATTCAGCCGATTAGTATGGATTGCATGCGCAATACTATCTCTTTTAGTTTCTCTTATTTTGGCAATAGAATTTGTTTTAACCAAATACAAATCTGTAAGAGCATATGAACAGCAAAATGTTGAGCTAGCCGATCTGATTACAATGGTACTTTCTGCAGCCTGCCAGCAAGGAACTGAACTCATTCCAATATCTATGCCGGCTAGAATAACAGTATTTCTATTTTCATTAGCAGCTTTCTTTTTATATACCTCCTATTCAGCAAATATTGTTGCACTATTACAAAGTACTGCGCCAGTGCTTAAATCATTGGAAGACATCACAAACAGTCATCTTGGTATTAAGATACAAGATCTTCGGTATAACAAAAAATTCTTTAAGGAGACCGCTGATACAAATATTATTAATTTATTCCAAAGGAAGGTAGAACCATTTGGTGATGAAGTGTATTGTTTGCCAGAGGAAGGTATGCGACATATGAGATCTGGAAAATTTGCATTTTTGGTAGAAGCAAATGTTGGTTATAAAATAATCAGCAATACTTTTGAAGAAATGGAGAAATGCGGCCTGGGAGAAATGAAAATGTTCTTCAACCCTAAACTGAGCATACCATTAGTCAAACGATCAGGACATAGAGAACATATAACCAGAATGATTACCAAACAAAGGGAAACAGGACTTTTAGAAAGAATACGGCTTTATTGGTTACCACACAAACCGCTATGTGAATCCAAATCAAGCGTTTTCATAAGTGTTGGTATTGCTGACTTTCTACCAGCTCTGCATGTTTTCTTATATGGGGCAGCAATTACAGTTGGCGTGTTTATTTTTGAATCTGCCTTCTGTTATGGCAAAAAGATCTATGTGCTAAAGAAGAGTCAACTAAAGAATGTCCGAAAAGTAATTCCATCTTACAAAAACGTGCATTAA

>IR75C

ATGAATTACCTCTTTCTTTTCCTATTTACAGCTTTACTCAGTTTACATTTAGTCCATTCACTACCAGGGAGTCTCATCATTAACTTTTTTGATCAAACTCACATAGATTCGATCACTATAATTAGCTGCGTTTCTTTAGGAAGAAAGTTATCTGCTTTGAGAGAAATCACTGCTGGAGGCCTGCAAGTGTCATTTTCATGGAAAACGATAGATCCGACACCTAAACAAAGAAGAGGAGCCGTCTTAGATTTGGACTGCCCAAAGATCAACACAATTATTGCAGAGATCAACAAAGAAAAAATATTCAATACACACACGGAATGGTTAGTAATCAACAGGATCAACAGCAAATCTTCCTACTCAAAAAAAGGAAACCTATACCTAAGTGAAGGTGTGAAGGCTATTTCAGCAAGCTACGCTCTACCAGGAAGTTGTGTCAGATTCGTACAATTAATACTTGACACAGGAGAAGCGTATTATTTTGATATGTACAGAGTTTCTATGTTAAGACCAATGAAATTTCAATTGGATTGGGCTGGCCCATATGAAAGTTCACTTCCAAATGAAATGACGCAGTGCGCAGAACGAAACAGGTCTAATTTGGAAGGTATTTTCCTGAAAGGTGGATCTTCCGTGGTGCACAAGGAGCTAATCGAGAATTTTGATAAAATTCATAGGGATGATGTGCCAGAGGTGGACTTCTGGGCTATGGTGCACTATCCAATTGCTAAATTGATTGCTGAACAACTGAACTCTGTATTAGAAATGGTTTTAATGGATGACTATGGATACCTGACTAATGGTAGTTTTGATGGATTGATGGGACTGTTGCAAAGAGACGAAGTTGACTTTGCAGCCACAGGATTAATAATTAGGCACAGCCGATTTATTGCCGTTCAATTTACTGCAGAAACTTTTGAACTTAAAACAGCCGTTATATCTAAACAACCGCCTCTATCCAGTGCCAGTAATATCTTCCTGCTACCATTTTCACAATTAGTATGGATCGCTTGCTGTTTGTTGTCATTGATGGTTACGGCTATTCTTATTTTGGAATACACTTTATCTAAACATATGCCAGAACAGCCTTACAGTCAAAAATACACGACAATGGCCGATTTAATTACCATGGTGCTATCTACAATTTGTCAACAAGGTACAGAATTAGCACCAGTCTCTTTGTCGGCCAGAATCACTGTTTTTCTGTTTTCATTGTTTGCCTTCTTCTGCTTCACATCCTATTCAGCTAATATTGTTGCACTTCTGCAGTCGTCAGCTCCTATATTAAAGAATTTGAATGATATTACGAACAGCCCTTTAGGTGTAAAAGTACAGAATTTAACTTACAATCGTTTCAACTTTAGGAGTGCAGTAGCTGAAGATGTGATCAACTTATATAAGAAGAAGGTAAAACCACAAGGAGAGAAAGCTTATTGCGAACCCATTGATGGCATGAAACTGATGAGAACTGGAAAATTTGCTTTTAGTGTAGAAACAAATCACGCCTACAAAATAATAAGTGACACTTTTGAAGAACACGAGAAATGTGGGCTAAGTGAAATAAGGATATTTTTCATACCTAAATTGGCAGTAGGAGTAGTTAGAAGATCTGGCTACAGAGAGCAAATCACACGAAAAGTTACCTGGCAGAGAGAAATCGGACTTCTGTCAAGAATAAAATCTGTGTGGTTGCCTCAAAAACCTCATTGTGAATCCGGTACATCCACATTTATTAGTGTAACCATCGAGGATTTTATACCGGCTCTTCTTGTATTTGCTTACGGTGCAGTGGCTGCTTTCTCTATACTTGCAATGGAAAACGTTCATTATTTTAGGATCAAGTATTTTTCTAACGCAATTGCAACTGACATTAAAAAAATACAATTAAATCCAGAAGTAATGATAATAAGAGGAAAGCACAAAGGCAAATGA

>IR75D-Ps

ATGATGAAGTCAATCTTGTCAACAATTTATTTTGTACATTTTCTAAATATTTATGCAACTGAAATGTTCACAAGTGCACAACTGTCACAGATAATTAGCTATCACTTTAACGATTTTGGTGGAGCTCACTTATATTTCTGCACAGCCAGAGCTGCTAAGTCTATTTTTAAAAGAATTACTGAAGGAAATAATGTTTATAACGTAAAATCATTGGATCGTTCCTTGAAAAACAAGAATATAGAAGTAAATTTCCAACAACCACAAGTGCAAGGAATATATCTAGATCTTACATGTAACCATGGAGTAGCCTTTCTACATCAGAGTTCAAAGATGTTCAACAGTTCTTACCGTTGGTTATTATGGAGTGATAATTACAAGAATACAGTGGATTTATTGACTCCTATGAGGGTATTAGCTGATAGTGATGTCACCATTGCCATATATGAGGAACATGGTGTGATTTTGGCCGATATATACAGGATACATGAGTCGCAACCCTTTAGAATAACGAACTTCACACAGTGGTCTGATAGTAAATTGATTCATCTTGATGTACCACTACCTAGAGACAATTTTGAAAGCATAGAGTTAGCCGCCAGTCTGATGATGTCAGAAATAAAGTTCAATAAGAGTAATTTACTAGAGCCTTTATTGGACAAGTCTTACAAATACGGTATTGATGGGATCTGCAGATACGGTCTGGCTGTTTTCTTACATCTTTCCGACATGTGTAACTTCACGTATTCATTCACCTTAAATAATAAGTGGGGAGTACAACTAAACAATGGAACCTGGAATGGCATGATAGGACAGTTACAACTAAGGCAGACAGAAGTGGGTTTAGCCCCTTATAAGTATGATGTTCAAGCAACTAAAGCAATTGATTATGTCACTACTATAAATACATTCAGGTCATGCTTCACATTTCTACAGACTAAAATGTTTGGCACGAACAAAGCTTTAATTCGACCATTAGATTATTCAGTCTGGGCTTGCCTACTTATTATAACCATTGCAGCAGTTCTCATGTTCAGATTAGTTGCAAGTTATGAAGGAGATACTATGTACAACGACAATCTCGGCGGAAGTGTGCTGATAGTTATAGCTGCATTCACTCAGCAAGGTTTACCAGATAGTGACGAAAAAGCCTCCACCAGACTGATTTATCTCAGCTTATTGATCATCTCATTCTTTACAGTTACATATTACAATACGGCAATACTAAATGGACTATTGTTAAAGGCGCCTAATGCCATTCACAATATTGAACAGCTTTTAGAAAGCGAGCTAGAGATGGGATTGTTGGACAGACCTCTACTGCGAGAGGCGCTAAGAAAAAATAATACAATAACAAAGGAAGTGCGTAAGAAATTGATGCTCGGAGAGCCTTCTGAACATGTCTACTCTTCCATCGTGGAGAGTGTGCACAGAATCAAGAACGGACAGTTTGCATTGTTCACTAAAGATGAAGAAGTGTACGCCGAGTTGCTGAACCAACTGAGCGATGCAGAGGTGTGCACACTGTCGGAAGTGCAGGTATCAAATCCCTTCCATGTTGGTGCTCTAGCCGTGGACGACAGTCCCTATAAGGAGATTTTCAGTAGAAAGTTCACGCTGATGAAGGAGCGAGGAGTGCTGGATCGCCAACAGCAGTATTGGCGAGAACAAAAGCCAGAATGCCATTGGCGTCAGGATCCGCTGATAGTCAGCACAGGTCCACTAGCATTGGCGTTTTTAATTCTGATTGTCGGTATGGCAGCGACACCCTTCGTCCTGATGTTGGAGATGAGCATTCACAAGATCAACCGCATCAAATCTATTGCTCCTGTGAAACCTTTTGGAAAGACAGATTAA

>IR75E-FX

ATGAAGGTTAAAGATTTTCTTTTTGCCATAATTAGCGTTGTTTACTGGCTGGATTCATTTGGGATTGGCCTGCCATCAAGTATATCGCATATCATCAGCAAACATTTTTTAGAAATTCAAGGTTTCCAGCTGTACGTTTGCGACAAGAGAGATGCTATAAAAATAATGAAAGCATTTTACAATGAACCAAGGCTTTTGTCTTCAAAGTTGTTAACTACAGCAAACTCTACAAGAGATACTCCGCTACATTTTATACAACCATCTGTACTTGGGATTTTTGTAGATTTGAGTTGTGAAAAAGGAATACGTTTCTTAAATGAGAGTTCTACAAAGTTCAACGCTTCATACAATTGGTTATTTTGGACCACAAATTACAATTATTTACTGACTCTTCTGCGTCCGACCAGGGTGCGGACAGACAGTAATGTAACCATAGCATTGTCGGGTGCCGGCGACACAATCAGAATGCTAGATTTGTTTAGGATGCACATATCGCTGCCATTCACCGTCACGGAAGTTGAACTATGGTACAGCAACGGAACATACATTCATCTGAAGGACCCTCCAGAAAGGAAGAATTTCCAACGAGCAGTGATTCCAGCTACTTTGCTTCTAACTTCCATGGACTACGATTTGAATAACTCCATCGATCCTTTACTCGATCAAGCTTACATGACTGGAATAGATGGTGCACAGAGATTTGGATTGTCAACATTTTTAACTATTGCAGACATCTTTAACATTACGTACAGCTACACTTTGGCGAAATCATGGGGATATCCAAAGAAGGATGGTTCCTGGGACGGAATGATGAAACATTTTCAAAGCGGAATATCGGAAGTTGGCTTTGGTCCAGCCCAACTTAGCTTTGGAAGAGAACCTTTAATTGACTTTCTAATGTCTCTCTATTTGTTCAAGTGCACCTTTACATTTAAACAGCCTAAATTATTTGGTACATCCAAGGCGTTAATAATACCGATAAACTATAAGACGTGGATTTGCATTTTTATTGTTGTAATTCTCGGAGCAATTATACTGCGGGCAATGACTGTCCACGATAAATCAGAATATTCTAATGATAACTGGAGTGGCAGTACTTTCTTAGTCTTAGCTGCACTTTCTCAGCAAGGCATGCCAGACAATCCAGATAAAAATCCTACTAGATTATTTTATTTCTTTTTCTTGTTTTTCACATATTTCATTTTTCTCTACTACGGCATTTCAATATTAAATGGACTTCTTTTACCAGCGCCGAATGCAATTCAGAATATGGACCAATTACTTGAAAGTGATATAAGAATTGGTATTCAAGATTTGCCTTACTTCCATTATGAATTGGAAAATTGGAATGATAGTTGGACAACTGGAGTGAATAAGAAGCTGCAAGCTCTCAAGCCTCCACATAAAAAGTATTATGGCACAGTGGAGGGATTACAAGAAATTAAAAAAGGACACTTTGCGCTGTACAATTTTGTTGGGCAATTTTATGATAGCGTACCTGAAGTGTTGACTAACGCAGAAATTGCATCTTTGACTGAAATTGAAAAGTTTCGACCGCGCAAAAATGGTGCATTAGTTGTAGAGAACAGTCCGTATAAAGAGATTTTTAAAAGAAAGTTGTTCTTCATGAGGGAAGTTGGCATAATTGATCGGGAACGAATATACTGGATGCCTCGCAAGCCTTATGCAAGCTGGGAGTTGGATGCTTTGTCAGTTGGGATGAAACCATTGAGCATTGCTTTTATAATCATAATACTAGGAGCTTTCATAGCCATAATTTTTTGTATGACAGAAGTTTTGATCCGCGAACGACTTAAAAAGAGGAATAAAATGTTAAAGAGTAAAAAAATTGCTTTTACTAGAAAGTTTAGAAATTGA

>IR75F-FX

ATGTGGAAATTGGTATTTTTAACACTTCTGTTAAACCAGTTAATGCCCCTTGCAAAAAGTGAAAATATATTACCATCTATGGTAAAAATTATAAAAATGCATTTTAGAGAACGAGAAGGGACCAATCTATTTTTTTGCCAAAAAATAGATGCTTTGATGATGTTGAAAAATATTGTAAATGCCGCAAGAAGTTACAACGTCAATCATTTACGTAAAGATAACATGACTAAAAGACTTCAAATTACATTTATGACACCTCAAATAATGGGAATATTTCTTGATTTGACCTGCAGCGAAGGACAGTATTTCTTAAAAGAGAATTCTGATTTTTTTAACGCTTCATTTCGTTGGTTACTCTACACAATAAAGCCCGCAGAAACCGCAGAACTTCTTCAACCTACAAGGTTTATGACAGACAGTGATGTCACCCTAGCATCTCCAAATGGTTCTGACATCATTCTTCAAGATGTTTATCGTATGTTCAAAACATTACCGATTATATCTACAGATATTCAAAGATGGTCAGAAGAAGGAACGGTAACCCAGCTTGCCGACCCTCCACCAAGAGTGGATTTTCAGGGAGTTACACTCACAGCGAGTTTGTTGATGGTTGACACGGTAATAAATAAATCTGAAGAAATCCCTCTTTTATATGATCCATCTTATCTGACGACGGTTGATGCAAATCGTTACGGCCTCACTCTCTTTACGCACATGGCGGAATATTACAACTATACGTACAGATTTATATTTTCCGATGTGTGGGGATATCCACTAAAAAACGGCTCTTGGTCTGGCATGGTAGGTCAGGTGAAAAGACACGAAGCTGATATGGGTTTAAGCGCTGCACACTTCAGTGTTGCCAGAACCAAAGTAATAGATTTTGTTGGCATCATAGATATTACGAGAGTGTGCTTCGCTTTTCTACAGCCCAAATTATTTGGCTCATATAAAGCTTTAATTCTGCCATTAGACGTGACAGTCTGGGCCTGTCTCCTAGTGACTGCAGTGGCCGGCGTTGTGGTATTTAGAATCGTTGCCAATTATGACGATACCTCTCAACAAAATGATTCCTGGAGCGGAAGTTTCCTCTTAGTGTTGGCCGCAATTTCGCAACAAGGTATTCCAGACAGCACGGATAAACTACCGACAAGGATCATCTACCTCAGTTTGCTGGGAGTATCATTTTTGGCGGCTGTCTACTACAACACGGCAATTCTAAATGGACTTCTACTGCCAGCTCCCAACGCGATACAAAACATGGATCAGCTATTGGCCAGCGATATGAAAATTGCCACTTCTGATACGAAATATTTACTCTATGACATGACTCACAATGACACTTTAACAGTAAAATTGCGTAAGAAAATGGAGAAATCAAAGTTACCAAATGGGGGCATTTTAACAGTTAGTGGAGGAGTCGATTTGGTGAAAAAAGGTGGTCTGGCATTGTACATACTGACTAGGGAATCTTATCAAGAAATAAACTATAAGTTTACGGCTGCTGAGATATGTTCTTTGACTGAAATAGAGAAGTATCGCCCACTTCCAACTGGGGGAATCGTGCAGAATAACAGCCCTTTCAAGGAGATATTTAACAGGAAGTTCACTTTAATGAGAGAGACCGGCATCATGAGCAGAGGTAAATTACTTTGGAATCCTCGTAAGCCGGAATGTGACTGGAAGCAAGATGCTCTTGTAATCGGTGCAGAACCATTGTCCATGGCATACGCTGTTTTATCATTAGGAACGATTGTAGCCATAGTGCTTGTCTTCATAGAAGTACATACATTTAGAAAACAAAAGAAATTATTGCGAAGAAATCATTCTTCAAAGTTTTTTATGCATCGGCAACCTCATTAA

>IR75G-Ps

ATGTCGAAGTCGTTCTTGTCAATAATCTATTTTGTATATTATCTAAATATTTATGCTAATGGAATGTTCACAAGTGCACAACTATCGCAGATAATTAGCCATCATTTCAATGATTTTGGTGGAGTTCACTTATATTTCTGCACACCCAAAGCTGCTAAGTCTATTTTTAAAAGAATTTTTGAAGGAAATAATGTTTATAACATAAAATCATTGGATCGTTCCTTGAAAAACAAGAATATAGAAGTAAATTTCCAACAACCACAAGTGCAAGGAATATATCTAGATCTTACATGTAACCATGGAGTAGCCTTTCTACATCAGAGTTCCAAGATGTTCAACAGTTCTTACCGTTGGTTATTATGGAGTGATAATTACAAGAATACAGTGGATTTATTGACTCCTATGAGGGTATTAGCTGATAGTGATGTCACCATGGCCATATATGAGGAACATGGTGTGATTTTGGCCGATATATATAGGATACATGAGTCGCAACCCTTTAGAATAACGAACTTCACACAGTGGTCTGATAGTAAATGGATTCATCTTGATGTACCACTAGCTAGAGACAATTTTGAAAGCATAGAGTTAGCCGCCAGTCTGATGATGTCAGAAATAAAGTTCAATAAGAGTAATTTACTAGAGCCTTTATTGGACAAGTCTTATAAATATGGTATTGATGGGATCTGCAGATACGGTCTGGCTGTTTTCTTACATCTTTCCGACATGTGTAACTTTACATATTCATTCGCCTTAAATAACAAGTGGGGAGTTCAACTAAACAATGGAACCTGGAACGGCATGATAGGACAGTTACAACACAGGCAGACAGAAATGGGTTTAGCCCCTTATAAGTATGACGTTCAAGCAACTAAAGCAATTGATTATGTCACTTCTATAAATACATTCAGGTCATGCTTCACATTTCTACAGACTAAAATGTTTGGCACGAACAAAGCTTTAATTCGACCATTAGACTACTCAGTCTGGGCTTGCCTACTTATTATAACCATTGCAGCAGTTCTCATGTTCAGATTAGTTGCAAGTTATGAAGGAGATACTATGTACAACGACAATCTCGGCGGAAGTGTGCTGATAGTTATAGCTGCATTTACTCAGCAAGGCTTACCAGATAGTGACGAAAAAGCCTCCACCAGACTGATTTATCTCAGCTTATTGATAATCTCATTCTTTACAGTTACATATTACAATACGGCAATACTTAATGGACTATTGTTACAGACGCCTAATGCTATTAACAATATTGAACAGCTTTTAGAAAGCGAGCTAGAGTTTGGATTGTTGGACAGACCTCTACTGCGAGAGGCGCTTAGAAAAAATGATACAGTAACAAAGGAAGTGCGTAAGAAATTAATGCTCGGAGAGCCTTCTGAACATGTCTACTCTTCCATCGTGGAGAGTGTGCACAGAATCAAGAACGGACAGTTTGCATTGTTCACTAAAGATGAAGAAGTGTACGCCGAGTTGCTGAACCAACTGAGCGATGCAGAGGTGTGCACACTGTCGGAAGTGCAGGTATCAAATCCCTACCATGTTGGTGCTCTAGCCGTGGACGACAGTCCGTACAAGGAGATTTTCAGTAGGAAATTTACACTGATGAAGGAGCGAGGAGTGCTGGATCGCCAACATCAGTATTGGCGAGAACAAAAGCCAGAATGCCATTGGCGTCAGGATCCGCTGATAGTCAGCACAGGTCCGCTAGCATTAGCGTTTCTAATTCTGATTGTCGGTATGGCAGCGGCTCCCTTCGTCCTGATGTTGGAGATGAGCATTCACAAGATCAACCGCAACAAATCTATTGTTCCTGTGAAACTTTTTGAGAAGATTGATTAG

>IR75H

ATGAAGGTTAAAGATTTTCTTTTTGCCATAATTAGCGTAGTTTATTGGCTGGATTCATTTGGGACTGGCCTGCCCTCAAGTATACCGCATATCATCAGCAAACATTTTTTAGAAATTCAAGGCTTCCAGCTGTACGTTTGCGAAAAGAGAGATGCAACAAAAATAATGAGAGCATTTTACAATAAACCAAGACTATTGTCTTCAAAGTTGTTAACTACAGCAAACTCTACAAGAGATACTCAGCTGCATTTTATGCAACCTCCTGTACTTGGGATTTTTGTAGATTTGAGTTGTGAAAAAGGAATACGTTTCTTAAATGAGAGTTCTACAAAGTTCAACGCTTCATACAATTGGTTATTTTGGACCACAAATTATAATTATTTACTGACTCTTCTGCGGCCGACCAGGGTACGGACAGACAGTAATGTAACCATAGCATTGTCAGGTGCTGGAGACACAATCAGATTGCTAGATTTGTTTAGGATGCACATATCGCTGCCATTCACCGTCACGGAAGTTGAACTATGGTACAGCAACGGAACATACATTCATCTGAAGGACCCTCCAGAAAGGAAGAATTTCCAACGAGCAGTGATTCCAGCTACTTTGCTTCTAACTTCCATGGACTATGATTTGAATAATTCTATCGATCCTTTACTCGATCAAGCTTACATGACTGGAGTTGATGGCGCCCAGAGATATGGAATGTCAACTTTCTTGACTATTGCTGATGTCTTTAACATTACTTATAGCTATACAATGGCCAAATCATGGGGATATCCACAGAAAGATGGTTCTTGGAACGGAATGATGAAACATTTGGACAGCGGAATTTCAGAAGTTGGCTTTGGTCCGGCACAACTTAATTTTGGAAGAGCTCCCATTATTGACTATTTAATGACTTTCTATTTGTTCAAGTGCACCTTTACATTTAAACAGCCTAAATTATTTGGATCATACAAGGCGTTAATCGTACCTATCAACAACAACGCATGGATTTGCATTTTTATCGTTGTAATTCTCGGAGCAATTATACTGCGGACAATGACTGTCCACGACAAATCAGAATATACCAATGATAACTGGAGTGGCAGTACTTTCTTAGTCTTAGCTGCACTTTCAATACAAGGCATGCCAGACAATCCAGATAAAAATCCTACTAGATTATTTTATTTCTTTTTCTTGTTTTTCACATATTTCATTTTTCTCTACTATGGCATTTCAATTTTAAATGGACTTCTGTTACCGGCGCCGAATGCAATTCAGAATATGGACCAATTACTTGAAAGTGATATAAGAATTGGTATTCAAGATTTGCCTTACTTCCATTTTGAATTGGAAAATTGGAATGATACTTGGACAGTAGGAGTGAGGAAGAAGCTGCAAGCTCTCAAGCCTCCACACAAAACATTCTATGGCACAGTGGAGGGATTACAGGAAATTAAAAAAGGACACTTTGCGCTGTACAATTTTGCTGGACAATTTTATGACCGCGTACCTGAAGTGTTGACTAACGCAGAAATTGCTTCTTTAACAGAAATAGAAAAGTTCAGACCTCGTCATACGGGTGCTTTGGTTATGGACAACTGTCCATACAAAGAGATATTTAAAAGAAAGTTGTTATTCATGAGAGAAGTTGGCATTATTGATCGTGAACGAACATACTGGATGCCCCGCAAACCATATGCAAACTGGGAGTTAGATGCACTATCAATTGGCATTAAACCATTGAGTCTAGCTTATGGAATCATCATAATGGGAGCTTTCATTGCTATAGTGTTCTGGTTGGCGGAAATGTTGATTTGTAAACGGCGTAAAAGCAGAAATAACCTTTTGACGAGACCAAGAATAGCCTTTACT

>IR75I

ATGTGGAAAATCGTTGTTTTGACATTCTTGTTAAATCTATTACTTCCGGCTATAAAAACTGAAAGTTTTTTGCCATCTATCACAGAGATTATTAAAATGCATTTTAGATCAGCTGAAGGCACCAATTTGTTTTTTTGCCAAAAAGAAGACGCACTACTTACGTTAAAGAAAAGCCTAAATGAACGTCAAAGTTACTTCATTAACCATTTAGGTGAAGTTAAAAAGCTGCAACTTACGTTTCTAATGCCTCAAGTTTTGGGAGTATTTCTTGATTTAACTTGTGAAGGCGGACACGTTTTCTTGAAGCAGAATTCTGGCGCTTTCAATGCTACATTCCGTTGGTTATTCTGGTGTAACGATCCCACGGCTACCACTAAACTCCTCCATCCTACAAGATTGATGACAGACAGTGATGTCACCCTAGCATACCCAAATGGCACGGATATTATCCTTCAAGATGTTTATCGGATGCATGACACCCTGCCAATTGTAGCCACGAATATTCAACGTTGGTCAAAAGAAGGTACAGTAACTTATCTTGCAGAACCGCCGCCAAGAGTGGATTTCAAAGGAATAGCGCTCACCGGAAGTCTGTTGCTAGTTGATACGGTTATCAACAAAACAGAAGAAATACCACTTTTATTTGATCCGGCTTATCTGCCTAAGGTTGATGCGAATCGTAGATATGGTTTCACTCTTTTTAAACACTTGTCGGAATATTATAACTTCAAGTACAAATATATCTATTCTGACGTCTGGGGATATCCACAAAAGAACGGCTCTTGGTCTGGCATGGTAGGTCAGGTGAAAAGACGTGAAGCTGATATGGGCTTAAGCGGAGTTCATTTTAGTGTGCCCAGAACCAGAGTAATAGACTTTGTTGGAATCTTGGATACCACTGTAGTATGCTTCGCATTTCTACAACCGCAGTTGTTCGGCTCGTATAAAGCTTTAATTCTTCCAATGAATGCAACAGTCTGGGCCTGTCTGCTAATGATAGCAGTAGTCGGCGTTGTGGTATTTAGAATCGTTGCTATTTACGACGATACCTCGCAGAGCAACGACTCCTGGGGTGGAAGCTTTCTCTTAGTGTTGGCCGCAATTTCGCAACAAGGTATTCCAGACAGTACGGATAAACTACCGACAAGGATTATCTATCTCAGTTTGCTGGGAGTATCATTTTTGGCGGCTGTCTACTACAACACGGCAATTCTAAATGGACTTCTACTGCCAGCTCCCAACGCGATACAAAACATGGATCAGTTATTGGCCAGCGATATGAAAATTGCCACTTCTGATACGAAATATTTACTCTATGACATGACACATAATGACTCGCTAACAATGAAACTACGTAAAAAGATGGAAAAATCAAAACTGCCAAATGGAGGATATTTTACGATCAGTGGAGGGGTAGACATGCTGAAACATGGTGGCCTGGCATTGTACATCATGACTAGGGAAGCGTATCCTGATATAAATAGTAAACTGTCAGCCGCTGAAATTTGTTCCTTGACTGAAATAGAGAAATACCGGCCATTTAAAACATCAGGGGTCCTGCAAAAAAACAGTCCTTTCAAAGAGATCTTTAACAGAAAATTCACGCTGATGAGGGAAACCGGCATCATGAGCAGAGGGAAACTGCTTTGGAACCCTCGTAAACAGGAATGCAATTGGAAACAAGATGCATTGGTAGTTGGTGTAGAACCATTAACTGTTGCCTACGCTGTTTTATCATTAGGAACCATTTTATCTTTTGTTATTTTCTTGATAGAAATAAGCGCTGAAATGAAACAAAAGAAATTTACAAGAAAAAATTCTTCATCAAGATTATTTAAGAAAAATCAATTTTAA

>IR75J

ATGTACATCCCTCATTTTATCAAAATGTTAATTGTAATTGTGCTATTATTTTTCAACAATCAAGCAGATTGTAGGATGATAGTTGAATCTGGTAGTAAAAATGCTATCATGTACAAAGTTATTGGTGAATTCATCGGAGTATCATCGGCAGGGACTCATCTTTATTTTTGCAGTAGAAAAGATGCGACGAACATTTTAAAACATTTTACTGACAAGCATACTATAAACAGTAGAGTGTTAAATTTTAAATTATCTTCAAACGAAATTATTGCATCCTTTCTCCAACCTCAGATTACAGAGATTTTTTTAGATTTAACTTGTCGAAGTGGAATACAATTTTTAGTACAGAGTTTTCAACACTTTAACAGTTCGTATCGTTGGCTACTTTGGACCAATAATTATAACCAAACAATAGACCATCTTAATCCGGCTAGGATATTGGTGGACAGTGCAGTAACTGTGGCGGTAGCTAGCGAAGATGAAACAATAGTTTATGATCTGTATAAAATGCACTATTCCACACCTTTCACCGTTACCAAGCTAAAATTGTGGCCAGCAACTGGGACTTCGATAAATTTTGGCATGCCTAAACCTAGGAATAATTTTATGGGCACTGTAATAAAAGCGTCCGTTGTGGCACGATTTTTGAACTATAGTCAAGATAACATCATAGAGTTGATGTTAGATCCTTCCTATATGCCAGGTATGGACGCAAAACGTAGATTTCATTTGCGTATGTTTATATATTTAGCAGAACATTATAACTTTACTCCCAGCTACAAATTTGCTAGAACTTGGGGAATTCCAACAAAGAATGGCAGCTTCATTGGTATGATAGGACAGTTGCAAAGGAATGAAACGGAAATCAGTGCCGTCTATACAATATTTAGTGTGCCCAGATCTGCAGTTATTGATTATGTTCTGGCAGTCACTTATGCTAAAGGCTCGATCTTGTTCTTACAACCTAAGCTGTTGGGAACTTACAAGGCTTTGGTTGTTCCATTGCAACCAATAGTATGGGTAGCTATACTTGTAATATCCATCGTAACAATAATCTCATTTAAGGTAGTCGGCCGTTGTAAAGAACTCTGGAGCGACAGTACTTTGATGGTGGTTGCTACAATTGCACAGCAGGGTGGTCCAGACATGGAAAATCTGTCGAAAAGACTATGGCATCTTGCTGCATTGAATTTCGCTTTCTTCATCACATTATACTACAACATTGCCATATTGAGTGGCTTGCTGCTGCCTGCACCGAATGCCATACAGAATATGGATCAACTACTTGCCAGCAATCTGAAGTTCGGTGTTGAAACTGAACCTTACATTCGCAATGATGCTGAAAGCAATGATGAACTATCAGTTCGCCTAAGAGCAAAAATGGCCAAATCTAAACTTTCAAATAAAGTGTACTTTGATTCAGATGAGGGACTAGAATTGGTCAGAAGAGGAGGCTTTGCATTTTATAATCTTGTTACTGATAGTTATGCTAAAATTAGGGACAAATATACTGAAGCTGAGGTATGTTCATTAACGGAAATAGATAAATTTAGTAAAACTCACATTGGACCAATAGTCCAAAACAATGGTCCCTATAAGGAATTATTTAGACGAAGCATACTATTAATGAAGGAAAATGGCATGATAAAGAATCAATTTAAATTCTGGGTTGCTAGCAAACCAGAATGTATTTGGAAGCAAGAAAAATTCACGATTGGTACGAGAGCCCTTACAATAGCTTTTAGTGTACTGCTAATTGGATTATTTGTTTCGGGAATCGTGTTCATACTTGAAGTGCGCAACTTCAGGAAGAAGCGGAAACAAGTTGTGTTCTATTCTAAAGAACTCTCGCTTCTTTCACAGAAAATTTTACCATAA

>IR75K-FX

ATGAATATTGTTTGGTCAATATTAATTTTATGCACAATTTTCATGGTTTCATTTACAATTAGCTCTCCTATTTCGGCATTAATCACGGATTTCTTTAAACTAAAACATGTGGGAATACTTTATTTACAAACTTGTGACACATATAATGCGACCCAATTCATGAAATTCTTGTCAGATGAATTTATTTTCTCAATGAACATTAATTCACCAAGATCTTTAGCGAATATTGTACAGAAGAGTGACCAGAGAATCGGAATCTTCCTTGATTTATTCTGTGAGGAAAGCATTCAGTTCTTGAAACTGGCAACATCTCTACATTTGTTCACCATAACCTATGAGTGGTTGATATTGTTAAAAAGTGAAAGTGACTTTTCTCTACTGGAGAATACTGGTATTGGAATGAGCAGTGAAGTAACGACAGTGATTTCATCACAATCAATTTTATACGACCTACATCGACCAGATGTAAGCAGTCTATTAATTAAGACACCCGTAGGGCGTTGGTCAGATCTATATGGGCTGGATTATAATCGAACACCAAGGGTACTTGACTTAGAAGGTGAAACCATCATTGGTTCAATGTTTTTTATTCAAGACGTGTACGATCACAAAAATATTTCACAGCAGATGCAAGACTGGTCTTTTAACAAAAATGATGATGTTGTTAATAGATTCTCTTACGTTTTACACATACATATTGCAGATATTTACAATTTTAAGTTAGAGCTCTTAGTAACGGATGGATGGGGAGCTCAAAAACCTGATGGACACTATTCTGGAATTATTGGTCATTTAGAGACAAGAGCGAGTAACGTTAGTGTGGCCCCATTGCTAATACTGCCCCATAGAGCCGCAATTGCAGATGTTTTAACCCCAATGTCCGACTACTGGAAGCCATTTGTATTCAAACAGCCACGTGCTTTGGGCACATATAGAGCTCTATTTTTGCCATTTGAGCCTGCAACATGGGTAACCGTTGCTTTCATACTTTTTTTTGCTGCCTTAATTTGCTATAAATTTAGTATAAATGAAAACCATCGACCAACATTTAGTGAGAACATTATTCTCTTCATCGGCATCATTGCACAACAAGGTTTAACAAGAGATCCCAAGACAATAGGTGAACGTCTAATGTGCTTCACTATGCTAATCATGGGAGCGATGGTGGTCTTGTACTATAATGCTGCCACAATGAGTGCGCTATTGTCTTCAACTCCACCCAAAATTAATAAACTTGACCAATTGGTTGATAGTAACGTCCCTCTAACACTTATGGACGCGCCGTATTATAATAATAAGGAGGTCCGGAATCGATTGTTGGATAAAAACGTTAGAGACAAAGTTGATAAATATTCTAAAAAGACTTATGGTCTTGAAGAAGGTTTACAAATTATTTTGAAAGGAGGAGCGTTTAGTGCAGAATATTTTGCAATTTTTGAAGGCATTGATAAAAAATTTAAAGATTCACAAAAGTGCGATCTTACCTACATTCCAGCCTATAAACCTCTGTACGTGTCCTCCTACTTTCCAAAAAAATCTCACATAAAAGAACTCTATAATCGTGGGTTTCTTCGATTGATGGAACATGGACTGCTTCGAAGAGAAAAAGAAAAATGGATTGTGAATAAGCCCAAGTGCGCTGGCAAAGAACAATTTCTTTTCGTCAGTTTGGAAGCAATTACTATAGCATTAGCCACACTGGGACTTGGTGTGACCATTTCCATTGTGGTTTTAATTTTTGAAATATATGCATACAGCAAATCAAAGAAACTTCTAAACGTTAAACGTAGAAAAAACTTACATACTGAATCATCTCTATCTCTTATATTACCAGTGATTAATTAA

>IR75L

ATGGATTCTTTATTGCAAATGATGATGAATGTGATGAACATTTCATTATTCTTAACAATTTATCTGGTGCATCTTCCATTTTGCAGTGCTTCTTCAATGTTTGTACGTGATTTCTTCATTTCAAGAAGTATCAATACTATTGAAATACATGCTTGCACCACTAATGATGCTATAAAACTTGTCAAGGATCTGACCAATGACGGTTTTCAAGTCATTTTAGCCAGACATAACCGCCATGAAGAAAATTATTATAGACGAAATCATTACTATAAACATGGAATTTACATTGATACTAACTGTAAAGCAGGAGTTGATTTGATTTACATGGAATCAAGTTTAAATTCATTTAATTCTAGCTTCTGTTGGCTTTTATATCATCAAAATTATAAGAGCATCATTTCTACTTTGAATCAGTTACAACTTGGCATGACCAGTGATGTAGCATTGGCTGTACCTTTTGATTCAATATATATATTACTGTATGATTTGTACAAAGTATTGGCTTGGCAACAGTTAATAAGCCAACTTATAGGGCGTTGGTCGCAAAGCGAAGGGCTTATTAACTTTGCATTACCTAGAAGGACTTATAACATGGGTAGAACTGAAATAAAAGCTGGTATATATTTTCTGGAAAAAGTTTACCATTTAGAGAATGTTGTGAATGAGCTTCAAGATGATAGATATAACGCAGAACATGACATTGCAAACCGGTTTACCTACGCTATTTCCCTTCACATAGCTGATTTGTACAATTTTTCGATAAATCTAGTAATAGAAGATAAAGCCTGGGGTACAAAACAAGAAGATGGCCATTTTGATGGTTTAATGGGTTTGCTGGAAAGAGAAGAAGTAAACCTGACGATCACTGCAATGCAGTTCATGAGCGCTCGACTAGATGTATCTGATTTTATTTTCCCTTCACATTATTTCAACTTTCCATTTATCTTCAAACAGCCGCGCGCTCTTGGTACCTACCGTGCGTTGTCACTTCAATTGCAGACACCTGCGTGGATCTGTGTGGGATGCATGCTACTTATCTCAATTTTTATTTATTGGATTGTCCAGAATGTTTACTTGAGAGCCAATGATCCTGATGGAGAGGCTACTTGGAGTGGCGCAGTTATCCAAGTGATTGGTGTGGTCACCCTGCAAGGATTATCTCAAGAACCTTATAGAATTGCAGGAAGGATAGCTTGTTTGTTTATCTTGCTGCTAGCCCTGATGATCTCAGTCTATTACAATGCTGCAACGATGAATGCTCTCCTCTCTCCAGCACCTGTCTCCATTCGTAATCTACAACAATTGATTTCAAGTAACATGCCATTATTTTTAATGGATATACCATATTTCAACACTAGAACGACCCGCGAAAGACTCTTATCTAAAGAAGTGATGCATAGAATTTTATCTCAACGTCAACCAGATTTTATAGCCTCATTAGATGAAGGCCTGGAGATAATACGAACTCGTGGGGCTTTTTTAGGAGAAGATTTTACACTTTATACTGCTATAGAAAACAGATTTCAAGATAGTGATAAATGCGATTTAAGCGCAATATCTGCTTTACCAAATGTCCCTGCAGGAAGTTACATTAAAAAATACTCCCATCTTAAGGAAATCCTTTTTAGAGGAAATCTGCGCATGAAAGAGCATGGGTTAATAAACAGACAAGGGTTGCACTGGTTTCCAAAAAAACCCACATGCGGTAGTCAGCAAATGCTTTTACTTGTTGATTTGAAGGTTGTTACAATAGCTGTTTCAATCATGATTTGCGGAATTGTTGTTTCTATAACCTTAATTTTTCTTGAATGTTTCTTCGCCCGAATCAATAATTTCTACAAAAATGATGGTAAAATTGAAGTAAAACAA

>IR75M

ATGTTGGTCGTACAGTATTTCCTTATACTACTGCTGCTCATTTGTAGCACACCACTTACATTTTGCGTAGAAGTTAAAATAATTAGAAATTATTTTAAATTTCAAAGTATATATGTAGTAACTGCGTATGTCTGCAATAGAAAAGATGCCTTATTTCTGTTCCAAAACCTAAGTATTGAAAAATTCAGGCTAAAAGTTCACGCAACTAACTGGAATAATAATTTAGATTTTAAAGCTAGACCGTTTCCACAAGGTATTTTCATTGATTTGACTTGTGATGTCAACCAAAAATTTATAATAAATGAATATAAACCAATGAGCACCGGTTTCAAGTGGTTCGCTTGGCTGGGCAACGAGAGTTTCAACTGTCTGGGTGGCTCATTCATCGGTGCAGACACTGATATGGTAGTGGCAACTGAAGCTGGAATGCTGTATGAAGCGTATAGAATATCTCTAGCGCATCCAGCTACTGTCAATTTAATTGGCAACTGGACATCTTCAGGAGAATTTACTATAGGTCCTAGACCGTTCAGAGATCTGTCAGGGGCCACTCTAAGAGGTGCTGTGTTTTTTTACCACCTGAAAGTAGATAAAACCAACTATTCAGATGTAATATTGAATAAAATGATTGAACCAGATTTGGACGTTTTCCAGCGATTTCATTACGCCCTGGAAGAAGATCTTCGTTTCATTTATAAATATAATTTGCAGCCTGTGTTCACCAATGCTACCGGTATTCACTTACCAAATGGAAAATTTACTGGAGAAATAGGTGATATTGCCTACAACAAATCGGATATTGGATTGAGTCCAGTCCGATATTTAGCCATGAGGGCTAAATACCTCAGCTATTTACCAGATTTACAAGTTTTTAGAATAATATTTGGATTTATGAATCCTAAAACAGTTGGGACTTATAAGGCCTTGCTATTACCACTCAGCGAAGGAGTTTGGGCTACATTGGCTGGCATAACTGTAGCCACTGTTCTTTGCTACCATTTTATCCAGCGATATTCTATGAATAGGCGAGATGATTATGATATTAGCGCTGGCGTCGTGTATGCAATCGGTTTGGTGGCACAGCAAGGATTAGTCGGCCATTCAGTCACAATTTCCTTCAGAATAATGAGCGTTCTGTCATTGCTTCTTGCCCTGCTTCTTTCCAATTACTATAACGCATCAATTATGTATGGTTTATTGTCTGAAGCGCCAACTAAAATCAAAACTCTTGACCAACTAATAGATAGCCATCTTGACCTAGGACTTATAAATCTGCCATATCTAACTTTAAAGGAGAATAAGGATATATACCTTTCGAAGAAATTACAAAAGAAAATTTATAATACCAAAAATTCAGTTCTTGACCTACAACCTGGTCTTCAAAGAGTTCGAACGTCAAGTTTTGCTTTTGCCGTTGAAGATGGACTTTTCAGAATAGAAGCAAATAATATGTTCACAAATCGTGAAAAATGTGATCTTAAAGAATTGGAATTGACCAGATTTCCAATATCACTAGCATTACAAAAAAATAGTTCATATGTTGAGTTGTTTACCAGAGGACTACTTTGGATAAGAGAAACTGGTATCAGCAAGAGGCTGACTGATCACTGGTATCCAAAACCTGCATTCTGTCTGGCAACTACAGAATTCGTTCATGTCCCTCTAGAAGCAGTGGCAGTAGCGTTGCTAATTTTTATTGCAGGAGTATCATTATCAATATTTATTTTACTTGGCGAACTTATATTAATAAAACCTCAACAGAACAATAAAGTTAGTGATGTGGTAATAGTAAACTGA

>IR75N

ATGTCCTTGATGCAGTACTGTACTAAACTATTGTTGCTTGTCTCTTATATATTTGCTTCATTTGGTTTAGAATTTAAATTAATATCAAATTATTTTAAAACCCAACATTTATCAGTGATTACAGCGTGTGTCTGCAACAGGAAAGATGCAATATTTTTAGCGAGGAACCTAAATGCTGAATCGTTTAGATCTAAAGTACACACTCCTCAATCCAATGATAATTTAGAGTTTAAAGCAAGACAGTATGAGCAAGGTATTTACATTGATCTAACTTGTGAATATAACCAGAAATTGATGGAAACTGAATATAATTCAATGAACACCAGTTTCAAATGGTTAGCATGGTTAGGAAAAGCAAGTTTCAACTGTTTGGACGGCTCATTCATCGGTGCAGACACTGATATGGTAGTGGCAACTGAAGCTGGAATGCTGTATGAAGCGTATAGAATATCTCTAGCGCATCCAGCTACTGTCAATTTAATTGGCAACTGGACATCTTCAGGAGAATTTACTATAGGTCCTAGACCGTTCAGAGATCTGTCAGGGGCCACTCTAAGAGGATCTGTTTATGTTTACGAACTCAAAGTGGGTAAAAACAATTTTTCAAATATAATTTTAAATAAAATGATAGAGCCAGATTTGGATATATTCCATCGTTATAATTTTGCTCTAGAAGAAGAGCTTCGTTTCATCTACAAATATAGTTTGCAGCCCGTTTTTGCTAACGCAACCGGCCATCTATTACCAAATGGAAGATTCATCGGCATGGTTGGTGATATTGCTGAAAATAGGTCAGACATAGGGTTGAGTTCAGCACAACCCATGGCAACTAGGCAAAATGTGGTCAGATTTTTAACTACGTTACAACAAATGAAAATGATATTTGTAATGATGAATCCAAAAACAGTAGGGACTTACAAAGCCTTGCTATTACCACTCAGCGAAGGAGTTTGGGCCACATTGGCTGGAATAACTGTAGCCACTGTTCTTTGCTACCATTTTATCCAGCGATATTCTATGAATAGGCAAGATGATTATGAAATTAGCGCTGGCGTCGTGTATGCAATCGGTTTGGTGGCACAGCAAGGAATTGAGGGCAAAACAGTCACATTTTCTTCCCGATTGATGAGCGTGATGCTTCTGTTTCTTGCTATAATTCTCTCAGCTTGCTATAACGCATCAATCATGAATGGACTACTGACACCAGCTCCCATTAAGATTAAAAATGTTGTACAATTGGTTGACAGCCATTTTGATTTGGGAATGGTAGATCTGCCATATTTAACGATGAAAGATAATTATGAGCTGTACTTTTCTAAGAAGGCGTTGAAAAAGATATTTAGTACTAAGGATTCGATAGTTGACATTCAATCAGGACTTGAAAGAGTTAAAAATTCTAAATTTGCTTTTGCTGCTGAAGATGTACTCATTAGAACGGAAACAAATAGATTATTCAATAATATTGAAAAATGTGATATCAAAGAATTGGAGATTACAAGATTTCCGTTAGCGATGCCAATACAAAAAAATTGCACTTACAGAGAATTGTTTACTAGAGGGTTGTTATGGATACGAGAAGTTGGACTTAGTAAAAGGTTGAAGGACCACTGGTATCCAGAACCTGCATTTTGTTTGGGCAACCCACAGTTCATAAATGTCACGCTGGAGGCAGTTTCAGTTGCGTTGCTCATTTTTCTATTCGGACTATTATTGTCTATGTTTATTTTTCTTGGTGAAAATGTAGTTATGAAACGTAAAATGAAGCGCAAGGGAATTATAAAAGTAACCAGAATAAAGCAGAAATTTAATTTATAA

>IR75O

ATGAAATTTATATTTAGTTTTGATTTTATTTTAATATTTACTTACTTATTTCTCAAATTTAATCATTTTGGCAACTGCCATGGAATTCCAGTTGAAACTATAAAATCATTTTTCTCATTTAAAAATGTAGGAGCTGCCAATATATTTCCTTGCACGAATCGAGATGGTGTTGATTTTTTGCTTCAAATGTCGATCAGAAGTGATAAGAATTCTTTACTGACAGGAGTAAAATTAGCACAGTATCAGAAATTGGATTTCTATAACATTTTTAAATCTGAAAAACATCCTTTGGGGACATTCATAGACTTGCGATGTGAATCTACAAAATATGTCTTTAATAAGGCATCTATGGAGTTGATGATAAATATGAGCCATGTCTTTTTATTGTATTCGCAAAATTATGATGAAATAATGGATTACCTTCAAAACACGAGCATAGCATTTGATAGTGATGTATCTGTAATGACCAGAGGAAGAATTTATGATTTATACAGAATTCAAAATGATATGGCTCTAATTGTCAATGAGATTGGATCATGGGCTAGATCAAGTACTACTCTCAGAACACCACCAATCAGGGATAATTTGCAAGGAAGTTTTATTAAAGCTTCAATGGTTTTAACGCGCCTAGAAGTTAATGGAACTTGTGCAGAACAACAATTGCTGGATAATACTTTTAAGCCACAGTTGGATGTTCTATCTAGGAACATTTATGCCAATAATAAACATATTGAAAGCATTTACAATTTCAGCTATTTAATAAACATTACTAATGATTGGGGTTACCTTCAAAAAGATGGACATTTTGGCAAAGGCAGTATGTTTGATGCTCTTGAAGAAAAAACTGTCGAAGTAGGACTAACGCTTTCAAGATTTTTTAAAGAAAGATATATTGTTTCATTTTTTCTTCCTGCTTCTTCAAAATACAGGACTTGCTTCATTTTTAAACATCCTTCAACTCTGGCATCTTATAGTGCCTTTGCTATGCCATTCACATTGCCATCATGGATGTGCACTTTTGCTACAATAATGCTCTCGGGATTGTGTTTAAGAATGATCAGAAATTTCAATATTAAAGACAAGCCGCCTAATGAGAAGAGTTTCTCTGCGTCAGTTTTGGTCGTGCTGGGAATCTTCTGCCAGCAAGGTTTCTATCCCGATTCATTTCGAGCTCCAGTGCGGATTCTTTGTGTTATTATGGAGGTAACTAGTGTAATGGTATACTCATACTATGGAGCTGAAGTGGTTGGCTTTCTCTTATCTCCTTCGCCAAAATTTCTCAATACAGTTGATAAACTTATAGAAAGCCCATTAAAAATGTATTCAGAAAATATCTCCTATCATCGCTCATATTTTACTGGCAATGTTAGTGAGAAAATAACTCGCGCATATGAGAAAGCAATCAAAGGCCCTACAATAGAACAAGACAAATTCATAAATCTAGAAAAAGGGTTGGAGGAAGTTCGGAAAGGAACTATGGCTTTGTACGGACAGGACACCAATATGTATACCGTTATCGGAAAAACGTTTAGCAATCCAGAAAAATGTTCTTTGTTCGAGATTGAAATGATTCGTCTTTTGGGATCACCAGTAATAAGAAAAAAATCACCTTTTAAGGAGTTAATGTTTAGAGGGACGATGCGCGTGCTGGAAAGCGGACTGCAATATCGTGAAACCAGGAAATGGTATGCCCAGCGACCTGAATGTATAGGCCAGCAGGAGAGTAATGCTGTTACTTTGGAAGCAACCCTTCTTGCCCAAGTTCTCTACGCATCCGGTCTTATAATTGCTATTGTTTTGTTTTTTATGGAAAAGCAACACTATTTTCTGAACAAAAATAAAGCAAAATTGAAAAGGCCCCGGATTATGGTAACACCGAGTGTGGTTGGTATAGGAAACATTCAT

>IR75P

ATGATATTTCCAAAAGAATATTTCCTTATAATTTTTTTAAACTATGCTAATTTGGGTATGGCTAGTAGTAATGCACCAGTGAAATTCGTTAGAAATTATTTTTTATTTAAAAATATAAATTCAGTTAACCTTATCTGTTGCTCTAAAGAAGAAAGTTTTAGTCTGATAGACTATTTGAGTAAAAATTCCAGCTTTCTGACTATGCACAAGTACACTTCAGTAGTTAATTCGACGAACTACTGGTTTTACAATTCTATGAAAAATACAGCAATACAAAGAATTGGTATTTATGTGAACTTAAACTGTGAATTTTCTGCAGAAATGCTTGAAAAGCATTTTTTTACTTATTACAGTGCCGGAAATTACAGTTGGATATTTTGGAGTGAAAATTATGAAGAAGGCTTCAAGAAAATAGAAAATATAAAAATTCAGTGGAATAGTGATATTTTCTTGGCAATCCCAGGCATTCGTATGTACAAAATCCACAGAATTCTGCACAGTACTGCGATAAGCTTACCTATTTATGCTTCTTGGAATGAATCATCTGAATTTAATTTTTATCAAGTGAATAATATACACAATTTAAAAGGACTTTTAGTCAGAGCATTTATTACGATAAAAAACTGGCAAAATGAGACTAATGTTTCAGAAAGCAAACTCCTTAACAGATATGTGGACGATCCGTCTGAAATTCTGCCAAAATTATCTTATACTGTGCATAATCTACTGGCAGAAATCTATAATTTCAGTTGGAATCTAAGTTTGTCTTTTAACAAACTTAACAGTAACTACTTAAATCAAAATGATGCTGAAGGCCAATTTTTAGCCCTCCAATCTGGTTTAGTCGATGTAGGCCTCAATCTTAATCAGTTAACATCAAAAATAAATGCGGTCGACGTTATTCCAAGTATTTGTAAGTTTAGGCAAGGATTCGTCTTTAGACATCCAATAATTTTTACTGAAGCGACCGATCTGCTGAGGCCGTTTTCAACCAAAGTTTGGTTGTGTTTAACAATAACTATATTTTTATGCGCTATTGGATTAAGAATTATTTTTTTACACAGAAGTAATGAAGAAATTTGCGAGTCTTCACTAAGCATAAGTATACTAGATTCTGTTGGTGTTCTGTGTCAACAAGGAATAAGCTCGCCTTCGATGAAAACTTCAGCAAGAATTGCACATGTTTCACTACTATTACTTGGCAGCTGGGTGTTCGCATTTTATAACGCCGCTATCGTTATACTACTGCTGTCACCGTACCAACGTTATATTAACTCTGTCGATTTGTTGATGAAGAAGCAGTTTGTTTTAGGTGCTGAGGATTTACCTTATAATAGATATTATTTGGGGATGAATTATGATACCAGAGCACAAATGGTGTACGATTTAAAATTAAAAGGTGAAACTCCAGAAAAGGACAAGTTTTACAAACTTAGTGATGGTATATCAATGTTGGAAAATGGAAGATTTGCTTTTTTTTCACATATTACTAATGTGTACAGCAAAGTAGGCCATTTTAAGACAGCCGATTTCTGCTCTTTAACTGAAATCAAGGTTTATGAACCTTTTCAGGTATCTGGATTCGTGAGAAAGTCTCTACCTTACAAAGAACTCTTCATTCAAGGATTGATCCTACTGAAAGAGACAGGAATTATAACAAGAGAAAATAATTATTGGCTAAACAATAAGCCTTTCTGCACTGGCGCTGTTAAAAGTATCGCGTTTAACATGCATTCATTATTTTTAGCTTATGCAGTTTATGCTTTTGGATTTTTAATTTCGTTATTAATATTGCTTTTGGAATTGCTATGCAAGTATAAACAAATGTAT

>IR76B

ATGGCACGAAGTTCTTTAATAAATTTCCTGCTAATTGGTCTTTGCTCCAATTATAAGTCTGATTATTTAAAGAACACCACTGAAGTGGATTTTAGCTGTAAACTCAAAGAATCTCATGAAATTGACAAGGATGTTCTACGTGGTAAAACATTAAATGTCCTGACTTTGGATGCAATGCCATTAAACGGTGCCAAAATTCAAGCCAAAAATGTCTATGAAGGATTTGGAATAACTTTTGAAATCTTAAGGACGCTTCAACAAAAATATGGATTTACTTATAATCTTACAAAACTGATACCACCTGTTATTGGCGACTCCAAAAATGGTGCCTTAGGTAAATTGGTGTCAAGGGAGGTGGATATGGTAGCTGCGTTCTTGCCGATAACTAGAAATTCACACGAGTTTGTGAAATGGGGAATCGACCTATGGCACACATCCTATTTCGTCCTCATGCAAAGACCACTCGACTCGTCCAGCAACAGTGGTCTTCTAGCACCATTCGATATGCATGTATGGATTTTGATTCTGGTATCATTAATCAGTGTTGGACCAATAATGTATTGGATCATGTGGCTCAGGGTCAAATTATGCCAGGACGATGATGCCCAATTATACTCACTATCTGCTTGTGTTTGGTTTGTCTACGGAGCACTCATGAAACAAGGATCTACACTGAATCCAATGTCGGATTCCACAAGAATGTTATTTGCAACATGGTGGCTGTTTATACTAATCTTGACGGCTTTCTATACAGCAAATTTAACAGCATTTTTGACTTTATCTTTGGCGAAATTACCAATCAATGACATTAGTGATGTGGCTAAATCATCGAATAAATGGTTTTCATCCGATGGTGGAGCCATCTATTATGCGGTTAAGGATAACGACGGTGATTTAAATATTCTGACAAAATCCATAGACAATGGACGTGGTCTATTTGTGGACGTATCACGCGAAGCAACTGTCACTTCCAGGCTATCTGATGGATGGCTCTATCTGGACGATTGGTACACATTGACGCGATTGATGTTTAACGATTATAAAAAGAAGCGATTGTACGATAATCCGAACATGGAGTCTGAAAGCTGCACATATGTGTTGACGGAAAAACATGTTCTGGAACGGCCCTTAGCGTTTGCGTACCAAATGGACAGCGTATTGCCAGAATTGTTTGATAAGGTAGTGCGACGTTTTATTGAGTCCGGCATCATCAAGCATCTAATCACTTTGGATCTACCGGACGCTGTAATCTGCCCATTGAATTTAGGTAGTAAAGATAGGCGTCTGCGTAATTCAGACCTTTTCACCACTTATATGGTAGTGATGACTGGATTTGTAGCCGGACTGTCCGTTTTCTTGGGCGAGATCTTGTGGCGTTTGACCCGTGGCCCTCTAATAGCGGATGCTTTACAACCAGAATTGTTGACCGCTATGCAACAGAATGGCCAGAACTATTTCACTATTGATAAGAAGTTCATGCCATTGCGCACACCATCGTCAATGATCTTGCACTATGGATAA

>IR93A-FX

ATGGCAGTACCATATTCAATAGCACTCTATGGGATACGTAATGAATTTGTGTCGGAAAGAAAAGCTAATGATACATTGTTGATTTTAGTTGATCCATTCTTCATGTATCCAAAATTCAGATATTTTGAAAATAAATTGAAAGAAATAGTGAACTCCAGGATTCACAAGTTTCTGGATGATGGAAGTCTCAGTGTGATATATAATGGACGAGATCTCAAATCTAAAGAAGATCTTACAGCTATATTCAGTATAACATCTTGTGAAGAGATGTGGAATCTTTATAGCAATTTCACTGGCAATGGTATTATATTTATAACTATAACTGAACCTGACTGTCCCAGACTTCCTCAACATGTCGGCACAACTTTACCTGTTTATGAAAGAGGTTCAGAAATATCACAATTGATACTTGATTTACGATCAAAGGAGAAATTAGATTGGCAGTCTGTAACTATTGTCCACGATAACTCGATTAGTGACAAACTTGTTGAGAAAATCACTCTAGCAGTGACAAAATCATTACCTATCACTAATAGCACCTGCGCTATATCACTTTATAAAATAGAATCTTCTAAAAATGATGTTGATGTAAAAAGGAATAAGGAGATATTTAACACAATATCCTCATTGCCATCACTGGAGATCAATAGGAATTTTCTTATCCTAGCAGAAGTGGACATCATTCCCGTGGTGTACGAATCTGCAAAATCTGTTGGACTGGTAGATCCAACCAGTAAATGGCTATTTATTGGAATGAAAACGGATTTTTCCAATCATAATAATATCAATAAGTTTATCCATATCGTCGGCGATGGAGAGAATGTAGCGTTTATTTATAATTCTACGGATGATACCGGTCTCTGTCTGAACAATTTACTCTGCCACGCTGAAGAATTAGTGGGAAATCTTGCTGTAGCATTGGATTACTCCATAGAAGAAGAGATACGATTATCTGAACAAGTTTCTGACGAAGAATGGGAAGTCATAAAACCGACGAAACAGGAACGAAGGGAAGCTATTCTAAATTTTATGAAGAATAAACAAGACGATATTGGTACATGTGATAATTGTACCTTATGGTATTTCAAATCAAGTGAATCATGGGGTATGGATTATTTTCATAAGGGCAATGCTAGTCTACTTGAAGTAGGATACTGGGCACCTAAACCTGGTCCAGTACTAGTCGATGAACTATTTCCAAATATTGTACACGGTTTTCGAGGGCGCAGTATTCCAATTGCTACGTTTCATTATCCTCCATGGCAGGTGATTAAATACGATGATGTTGGAAAACCTACTGAATATAAAGGATTGGTGTTTGAAATCATCAATGAACTGTCCAATAGTTTAAATTTCACATACGATGTCATCATAATATCAAACAGGACTGTGCTGAAATCTATAACTAATTCCTTAAAAATTGATGAGAAATTGGGAGAAGTTAGCCTAGATGGAAGAATTGAAACTTCTGCTTGGAAGCAGGCATTAAAATTATTAGAAAATAAAAGGGTACTCATCGCTGCAGCAGCATTTACTGTGACGGAAGATCGTAAAAAAGAAGTGAACTTCACGTATTCAATAAGTATAGAAGCTTACGCATTTTTAGTGTCTCGCCCGAAGGAATTAAGCCGAGCTTTACTATTTATACTTCCTTTTAGTTCAGACACATGGTTGTGCATCATTGGAGCTATACTGCTAATGACACCACTTCTATGTTTTGTCCACCGTATTAGCCCATTTTATGATCATTACAGCCATAGAGGTAAAGGTGGTTTTACGAAAATGATGAACTGTTTTTGGTATCTCTATGGTGCATTATTGCAACAAGGTGGTGGAATAATGCCGGAAGCTGACAGTGGTCGGCTGGTAATCGGAACTTGGTGGCTAGTAGTTCTGGTTGTAGTAACTACATATTCAGGAAATCTAGTAGCATTTTTGACATTTCCTAAAATGGATAAAATTATTTCTAACGTTGACCAGTTAATGGAACGAAGAGAGTCTTTATCTTGGGGTATGCCAGAAATCTCGACTCTGCATAGTATTCTTAAGAGTACAGATAACAGTAAACTTAACGCACTATCAGATGGTGCAAAACTTCATTCAAAGTTAACACCAGAAATTATATCAGACATACAAAATGGGAAACATATTTATATCGATAGAAAGACTATCTTAGCTTTTGTAATGAAGCAGGAATTCATTAGAACAAATCGATGTGATTTCTCTTTGGAAGAAGAATTCTTGGAGGAACATTTAGCGATGGCCTTGCCAGTACATACACCGTATTTGAAAATATTCAATTCCAGGATTTATGAAATGCATAAAGTTGGTTTGATCCAAAAATGGTTGGTCGATTATCTACCAAAAAGAGATAAATGTTGGGACGCCAAATTGTCTGGCGAGAGTAATACGCATACAGTCAATATGGACGATATGCAAGGATCATTTTTCTTATTGTTTTTAGTTTCTTTGGGAATAATGTTGATAATTGCCGAATATTTATACCACAAATGGAAAACTACTCAAGAAAAACATGTCATCCAGCCGTATGTTAGCTAA

>IR101

ATGGTAGATGGTGTAGTATTTTTATTGGAGAACTATTTCGATGGGGATTGTTTAACGCTGTTCACCTTGCGATTACAAGCCACCGAGTTGGAGTTACTATTGCTGAAACAACTGCACAAAATGAAGGCATTCTATTTACTACGCCCTGTTGTCAATATAGTGACTAAAGTGCCCTTCCCATGTTTCTATATAATACTCCTAGGAGAAAAAGATTACCTTTTAGACAATGATCCCGCGAAGATTAATTCCAAATTTTTCTTAAATAAAGAAACAGAACTTTCCAAATATATCGTGATTGCCCAAAACAAACTGAACTTCCAGCAAAAATCTAAAGCATTGATAACTTTATTTCGTCGAGGACATCCAGATGCTCTACTATTGAATCCGGAAGACAATCTTTTTGCGTATACACTATTTCCATTTGGTGAAAAGATGAAAAATTGTCCAGAAAATGGTATAGAAATAAAACTTCTGGATAAATGGGTAAATAATAGATTTCATAAGAATTTATATCCACATAAAGTACCACAATTATTCAATGGTTGCAATCTGGATGTGTCCTTCATTGAATATGTTCCATATACAATTTACCATGGAAGGAATGAAGTTAAAGGAATTGATGGTGTTATGGTAAAGCTGATTGGAGAATCTTTAAACATGACAGTTCGTTGGTTACTAAGAGAGTCATGGTTCAGTTACCTCAAGAACGGTAGTCATTATGGTATACTGCCGGACATATTGTATGGTAGAACTCATTTGGCTTGCGGAGGTCTTATCCGTTCTCCCGAGTTGGCCGGGTACGTGCATTTTACAACCACTTACGCCATAAGTTACCTAACATGGTATCTACCTCAACCACCCTACGAACAAACCTGGAAAACTATGTACAAAGCTTTTCAAAAGAAGTTACTATTGCTTTCCATAGCAGTTATATTTACTATCCCAGTTTTTATAGCAATTATAGCTAAACTCGCCGACAATGAGCTATCATTATTCAAATGTATAACAAGATGTTATGTAGCTGCCTGGGCGTTAGCGGTTGGCAATTCAACGGGCAAAAAACCTCGAACAACTTCCGTAAGAATTATCTATGCAGTTTGGATGATCTATGCGATGCACTTAGCTCTAGCGTATACAGCATCCTTAGCAGGTCTCTTGACGAAAAGTGCGCACCGACCAAGAGTGAAAAACTTTAATGACCTGATCAATACTGGCTTGCCTACAGCCATGTTAGACGTTCACCAATCGGCCACGACAAACATTTCGGATCCGGAAGTTAGACAAGCGTTGGACAAATTTCTCCTAATAGAAGACACAGACGAGGCGATCAGCTTAATGGCTCTTCACGGTAATATGTCCATTCTAGACAACAGTGCTTACGTTGATTACACAATCTGTTCAAAATTTAGCAAGAAACCGATCTATAACAGCGGCTTATCCACCTTTCCAATGAAAACTGGATTAATGATGAGAAAAAGTCATTTTCTTTACAAAATAATTAATAAAATGATTATTAGACTCGTGGAAAGTGGTATAAATGATAAAATAATCAAAAGCACCACAAAGTGTCGTAAAACGAGCAGAAGATTAAAATCTGGACAATTTAAGGCCAGCACTTTGAGAGGGCTTAGCGGTGCATTTCTGGTTTTAGGCGTCGGTCTCCTTGTTGCCATCGCTGTCTTCTTTTTGGAGCAGATTATTTATCAAAGAAACCTTTACAGAAATGATAAAGAAGCTATTATTCCTTATTTAAATTAA

>IR102

ATGAAGCTATTGATTCTTTGTATTATAACTAATGTTTATCCACTGCCTGCTTCTTCTGTTGAAACTAATAACAATTTATATGATTGTATTCATTTAATTGCTAATAAAAGTGTTTTAGTGAAAACAATTATCATTTTAAGAGAGGAAATTGAAAACTATAAAGTTAGTGTAGATTTATTGTTAAAATTAAATTGGCCAAAAATGATAATGAATTCTAATCAAAGTTCATTGAAACATCTTCAAGGTGTATATTTATTATTTCCAGATAGTAACTGTACAGATTATTTAAATTTATTTGAGAAAAGTAGCAAAATGATCAATTCAAAATATATAATTTATACGGAATCGATGTTTGCTATGTTTACTTTATTCGATATTAGTTGGAAATTTAGGGTGATCGATATTATTGCCTTATATGGATCGAAAGAGGACACTATCCTTGTTTTTACATATTTTCCATACGGAACTCATGGTTGTATGTCCACACAGCCGGTTTTGATCGATAGTTGTTTTAGAAAATATTTTCAATATGATTATTCATTGTTTTCCAGAAAAAACAAATTGTACAATTACCATGGTTGTCGTATTAAGGTTACCCTTAAAGATAAAACTGTTGAAAGTGTTGACACGAACGCAGACGATCTTGCTTTTAAACTACTGAAATTTCTAAGTATAAAAATGAACTTCACCATGCTGATAAATGTGGCAGATGCTGGAGAAAATGCTTATGCAGCTGCTTATCAGAATTTCACTTCAGCTTCAGTGATTAGCGTTGCTAGAGGTGATTCTGAATTAGGAGTTGGAAGGTTTTCACAAATTTTGGACTATCATCCGATGGTTATTACATCAAAGGAAATTTCGATGGATTGTTTTACATGGGCTGTACCAATTAAAGCAGGTAAAGTGCCAAGCATTTGGTACGTTTATGTAAATGAATTTGATTATTTAACTTGGATATTGATTGTATTGGTATATTTTCTAATTGCTTTACTTTTTAATTATATTTTTTCATACTTCATCAGACCTAAAAATGATCGTAACTTAAATGTGTTACATATTTTCGGCACTTTCATCAACGTTTCTGTGAAAAGTCACATCTTTCACTGCACTGAAAGAGTTATGTACGCTTCCTGGCTTTTATATTCTTTTGTCACAATATCGGCTTACCAAGCATCCTTAGGAAGTTTGGTCACAGTACCGCCGGAAATACCTGAAATCGATACTACTCAATATCTTCTACAAACAGGTCTAGCACTAAAAAGCGAGCCGAAAATCTACCACGTACTGAGTGCTTCTCGAGAAACCAGTGAACAAATCAAACTAGTACTCGACAGTTTCGAGTTAATTCTGCCAGATGAAAACTTCTCATCAGCTGTACATAATATTTATAAGAAACGTAATTTAGCTTTATTCCATACTAGTGATAAACTAGCCAAAACTGAATATGAAATCAAATTAAAATTAAATACAAACAAAATAGTGCATATTATAAACGACTGTCTAATTAGTTCGCCAACTTCACCGTTTATATTGAGAAAAGATAATTTTCTGATAGAACCAATCAATGATTTTGTTACTAGACTTTTTGAAAGTGGTATACTGAAATATTGGGATAGAAATACATTGGAGTTTAGCACGCACAATTCAACAAATCTGCATTCAGCATCTATGAATATTATAAATCTGTTTGGTGGTTTAGCTGTATTGATTATAGGTTATGTTATGGCAATTTTTATATTTTTAGCAGAAATTTTATCAAAAACATGGAATTTATAA

>IR103-FX

ATGATAGCTTGCTTGGCGGAGCCTCTGACACTGACATCGCTTAATGCCAACTATAGCCTGATCAACCAAGACAATCTAGCCAACATATTTGCATATGCGTTTGAAAGACATTATTGCGCGCAGATATCTTTTGAACAATCTGAATATGCGTCTTTTTTAATGAAACTAATACATGAAACAAGAACTTCGCCGACCATCGCAATCGGCGACCAAAATGAAAAACTGTCATGCCCTTATCATATAATATTTACTTTAACACTAAAAGCCTTGCCATATTTACTAAGTAAAACGAAGATAGCCGATCGAGCTAAATTACTCTGCTTAGTTGCAACTGCTTCATTTAAAGATTTTCAATTGTTTATTAAAGAGGAAGCAGTAAAATATTTTGGACAAAGTTCAGGATCATTAGTTGTACTGACGAATCCGGACATAGTTTACAGATACAGTTTAATAAGCAACAGATTTATTAAAGAAAACATTGGTAATCCAAAATGGATGTCTGATGTCGTGCGATTCGAAGATTTGAACGGCTTAGAATTAACGGTCAGCACTTTTAACTGCTCAATATTTTCTCAAATTGGTCCACTGGATTCAAATGGACATCCAAAATGGATTTATGGTGTAGAAATAGATTTCCTACATTTATTATCAAATAGACTGAATTTCACATATCGTATAGTAATACCAACTGATGAAGACAAACGTGGCTTTATTAACGAACAAGGAATGTGGTCTGGTGGCTTACTGAAGCTAGTTGGAGAGAAGCAAGTTGATTTAGCATTTTGCGGGATCTTATTAACAGATTATTTAAAAATGTCAAGCATTGAGTTCGCACCACCGATTACAAAGATATGCCACAAGTTTCTCGTTCCCAGGCCAGTTCTATTAACAAATCAGGTTTTTGGCATATTTAAGCCGTTCAGAACCCATCTATGGGCTTTTATCACCGCCACCACTCTGTTCATGGCCCTTTGTTTCCGATGGATTGCGGATATCAGTAGGGAAATACCAACCATCGTCAGTCGTCAAAATAGCGTTTACAGAAAATATCCGAAAAGTTTGTTTATTGTTTGGGCAATCCTTTTTGGCAATTTCCCGAAATCATTAGTAACTAAAGGACCTCTACGCCATATCGTCACTTGGTGGGCTCTATTCTGCATGCTGATGAGTGCTTGCTTCGCCAGTTCACTCATCTCTCATCTTACTACACCTACTTATTCTGAAAAAATGGATACTTTAAAAGAATTAGTAGAGCAAGAATATTTTTGGACCACCAATACAATGTTAGATTTTAGAGGCATATTCAATCTGGAGGATCAGTGGCAAAGAGAATGGATAAAAAAATTCTTTTTATCATCGGCGGACGAAGAAAATCAACTTATAAAACAGAAAGCAAAGATGGCCGTATTTGGTGGGCAATTGGACGACGCTTTTTATGTTTATGATGATATTTGCCCTTCATGTCTTCGAGCCTATGAAGTGTCTTCACAGTGTATTTCTAGCCACACTACAGGATTTATATTACCCAATGGTTCGCCATACAAGAATATATTCACCCATTTTGTACGAATATTTCAAGAGCGAGGATTGTTTATAAAGAAATTTCGCGATATATCAGATAAAGAAGCGTTGAAGAAACAATACATCAGAGAAGTGGTTGTTCCTAAGAAGAGGACGATCGTTTGGAGGCAGCAATTGGAGTTAGACAATATTATTGGGATATTCTACGCTTTTGCACTGGGCATTCTAGTGTCTGTACTGGTGTTCCTCCTAGAATGGACTATTTAA

>IR104

ATGAAGTTAAAGTGGTTTGTGTTAAACTTGTTGCAAGTGATAAGTGCAGATTTAATCAATTCATCTAAAGAAAATTTCTTTCTGAACATTGTCGCGAACTTACCTAGGGAAAAAGGTGGAAATTTAATTTTTCTACAAGAAAATTTTACTAATTTACTACCAAAATTAGCACAGATTATCGCTTATTCATTGCTAGCTGTGAAATCTCAATATTTACTAGCAACGCCTAGCAACTGTATAATTTTATCCACAAACACAGCTGATCTAAATAAACATATGTTTTCTTATGGTACCTGTATAGTTACTGTATTCTATGGTATCACATACAATTCTGCTAGCAACACGAAATTTCTAAAAAATCAAACTAATTCAGCCATTATTATACCCATAGACAGTTCATCAGTAATTCATTTGAAAACACCATACACACAGTACGAGATAAATGAATATAATCGCTTATTATTGAAGAAAATTCTAGAAAAACCACCAACTAATCTATACAAAAAATCGGTACGTGTAACGATGTTTCCAAAACGTTTTACTTCGGTGAAAATCGATGGTGAAAAATTTGTCGGTTTTGATGGCGATATATTGAGCATTTTAGAAGAACGATTGAATTTCTCAACTGTGATGATAAAAAATAATGGACGTTATGGTTATCAGCATCCGAATGGAAATTTTACCGGATCATTAGGCGATGTTATACATGGTAAAGCGGATTTAGCCACAAATTCCATCTTTATAAAATATTATAACAGTAACGATATACAATTTACGACAGTGGTGATGCTGGAAGAGTTGTGCATAGCCGTACCAGCATCCGAGATGATACCACAATGGATGGTCGTGTTTCTATGGTTTGAGCCTGAGGTATGGTTTGGCATACTATTGACCTATACAATTGGTGGCATCCTATGGCAGAAACTTGTTAATAATTTTGGAAAACCAAGAAAACTTCACATCAACAGCAGTACAACATGGCTGACAATGGCTAAAATTATCTTACAAATGTCAGTAAATGGATTTTCTACGAGATTCTGGCAAAGAATATATCTATTGAGTCTGATATTGGGCAGTATGGTGCTGACGAAATGTCTAGAAGGATCTTTGCTGGATAAACTGACCAGAAATTCCTACTGGAAGGAGATAAGCTCGCTAGAAGAGTTGGGAAAATCACCATTAAAACTATACACCTTGTCAGAAAATCTACGAAGAGATACATTTCTAAAATCTAATAAATTACAATCGAAATTTTACGTCTATAAGAATGTTTCAGAAGATTTGGTCCAGCTGGTGTACAGGCGTAAAGATGTTGCATATCTTGGCAGGAAAATTGATTTGATCAGGCGAATTGGTGAAATCCGAACCAATCCGAGAGCAGTTCATATCATTGAGGAATGTCCCAGGTCTTATCATCTAGGCTATCTATTGAGAAAAGATTCTCATCTATACCAGCCAATAAATACGCTATTAGGTCGCCTATTGGCTGTTGGCATAATAGATCATCTACAAAATAACTATATAAACGCAACAATGGCCAAACAATTGGCTAAAAAGTTTAGGAAAAATCAAAGAAAATTCCAACCAGTAAAATTACGTTTACAAAATTTAACAATTGGTTTGCTAGTATTGGTGTTTGGCTACACGATTAGTAGTTTAGTATTTATTGGTGAACTACTAGTTAAACGTTTTTTAGTGAAACGTAACGAAGGATTTGTTTACGTAAATTAG

>IR105-FX

ATGTTAAGGTTCATCAGCTGTTTCTATTTTTTGTTAATTTTTGTTTGTGTAAACAGTAGTGAGCTGTTTTTGAATTCATTGGCCGATATAACGCATAATACAAATATTAAATTAATTTTATTAAATAAAGAATTATGGCGACCGTTTGTTTATAGCTTTCATGGAAGTAATTGTATTCAGGTGACAGATGATAATTCAATATTTGCTAATATCATACTGAAAACACTGCATAATGTGCACCAGTCTCCAGCATTGTTCACAGCAAACATCCAAGAAGTGCAGAACAGATGTCATAGTCATCTGGTGCTGGCAAAGAAGTTAGACAAACTACCATCATTGTTGGTACAGATACCTCGTTGGGCTGCCGATCGTATATTGTGTTTAATTTACAAGACTACTGATAATGAATTTCACAGATTTCTTAAGGAATTGTTGGTGGCCCACCTAACAGTCCGCGGATGTGTTTATTGTTTATTACCATTAGTTTTTGCAAAGTCGGTTGGAACCTACCTCAGTAAAGGTCATATTCTATTATCTTCTTACTTGCCGAAGTACGACGGAATCTATAAATTCTCCGGACGTGAAATCGTGGTTAGTACAACCAATTTCTCAATATATACAAGAATTTGGCCTTCCAATGATTCTTCTCGACCAGCTGAACTTGGTGGCATTGAGTTGAACTTATTTCAAGATATGACCAAAAGAATGAATTTAACCTACAAGTTGCGTCTATCAATGGAAGGTATTGGTGTTAAAGAGAATGGCCGTTGGAAAACTGGGATATTGGGCGATATAGCTGAACAAAGAGCCGACGTTGCAATCCGTGGATTATGGGTATCCATGGATAAAGTAAACAGCGGTATTGTAATGTCCTCATCTTTTAGAGCCATTAAATTGATGTTTTTGGTGCCTAGACCAAAAAGGTTGATGGGTCACTGGTATGGAGTGTTTGAACTGTTTCAAAAATATTTATGGAGCGTAATTGCTATTACGCTGATAGTTTATACGCTTATATTGTACGCTACATGGAAATTAACCAAAAGCAACATCAGGGGAAAAGACATGAAATCTTTAGAAAGAGCATTCTTTCAACTTTACGGTATGCTGGTGCTTGGATCTTGGCCCAAAGTTAATCCACTTCAAACTTCACAGCGACATTTGTTTATGTGGTGGGCCATATTTGCGCTGCTTATATCCACAAGCTTTTCTAGTAGTCTGGTCTCCAAGCTAACTGCTCCAACCTATAGTAGATCTGTGAACACAATTGAAGATTTCCTACAAGAAGGATTAACTTGGACCTTGAGAAAACCGTTCTCTTTCTACATTGTTTTCAATAATAATGATGACACACATAAAAGATGGCTGTCTCAGATTGAATATATAAACTGTACAGAAGAATTCAACCAAATTTTACAAAAAGATGACAAACAAGTGATCCTTGGTTATGAAATCAATGACAGCTTCCATTTGTTTGACGATGCTATCCAAGAAGATTTACTAGAAAAATATGTAACTACAAACGAACGTCTAATAGAATTCTTTATAGCATTTGCACTGCGTGAAGGTTCCATCTTCAATCAAGCTATCAATAGATATATAAGATATTTTGTGGAATCTGGTCTTGTGCAGCATATCACAGACTTAGAAATCAGACATAAGGCTATATTCAAACAATCTGTTTATAAACTTCGTAAACCTGAACGCATTATTGAACAACAAGATTTTAAAAGTTTAACTTTGGAAGAACTGAAAGGTATATTTACCATTTTAATTATAGGATACAGTGTTGCCTTTTTAATTGTTCTATTTGAAAGATTACACAAAAGTAAAGTTATACATACGAGTATATAA

>IR106-FX

ATGTCTAATTTATGTACACTGGTAATGATTTATCTAAGTCTAAGCGTATCTAAAGGTAGTGATGATCTGTACAAAGCTATGGACGACGTCGCCGATGTACACGAAGATAGAATTGCCTCCTTAGAGAAAATTTTCTCATATACACTTAGTGGTTTTCTCTGTATGCAGGTTATCATTGATGGAGCACCATTTTCTGCTGCACTCCTGAAAGATATCCATCAAACAAATACTTTACGACCAATAATAGTCAGCGATACCGGCACAATGGAAATGAGATGTAAAGCGTTCGTAGTTATATCACAGAATATTTATAAAGTACCAGAGTTGATTGATGGACTTCCTTTATATGCTGAAAACAGATTGGTGATCTTTGTGAAGAGTCACAGATATTTAGTTACCCAATTTGCTATGAAAAAAGCCCATATTTTTGGAAATATAGATGTATTTATAGTGACACCTGATAATTATACCGTCGGATTCAGGATGGGTCAAACATTGAGAAAATTTAATCTAATTGAAGATCTATCGGGTGAATGGACTGACCAAGAAAACCAGATGGATTTTCACGGTAGAACTATTAAAGTTTTAACTTACAACTGTTCGATGTTTTCAAAAATAGGACCTCCAGACAGCGAAGGGAAACCAAGTTTCGAAGCAGGAGCGGAAATGATAATTTTCAAAGAAATAGCAGCACGACTAAATCTTACTTACAAAATCTACACCCCAAAATCATATGTAAATAAGCTAGTTAACCCGCTATTTCCAGTGGATAAATGGTATGAAAAAACATTGGAAGACCCTGGAGATAATGGCACAGACTTGGCGTTCTGTTGCCTGTTGAATTCCTTTAAAAGGCGTAAAAGTGAACTGACTATGTCAGAATCGCTGATGCTATTATGTTTTCGATTTCTGGTGCCCAGGCCGCGCCCAATACAGACTGAATGGTATGGTATATTCAAACCGTTTGCAAATGATCTGTGGTTTACCATTGCCATATCCATCGGATTGACTACGTTTGTATTACAAAGAGTGACAATCTTATCGTCACGTATTTTAGAGACACAAGGAATTTCTGGATACTATGGCTTAGGTGGATCTTTCTTACAGCTGGTATCTATACTAGTGCTATCTAGCATCCCTTCATCCACTGAACACCAAGGACCTTCTAGATATCTTCTTACTTGTTGGGCTCTTTTCACCGTGATTATTACCACATCATTTAGTAGTAGTCTATCATCAAATCTAGCCACTCAGTTATATTCGCCAAAACTTGATAATATTGATGACTTTATAAGGGAACAATATGCTTGGATTTATGTTAAAAAGCCGGATACTGAGGAAATGTTTAATGTTGCGAATAAGGATCATGCCAGATGGTCAGTCAGAGCAAAATATGCCAAGAATTTCGACTCATATAACGCCCTTTTGCGCAAAGGTAAATATATTCTTTGGGGAGCCCAAGTAGAAAATGGATTTGTGCTGCCACTTGAACAATTAATCGATAAAAAGATCCTAAGGAAATTTGAAGCTTCTTCAGAATGTTTTATTGAATATTTTAGTGGTTTTGCTTTCAAAGTGGGCTCTTCATTTCTGGGACCATTTAATAATCTAATACAAAAATACAAAGAATCAGGCCTAATTTCAAAAGTTTTAATCGATTTGAATAAAAAACGTTACAAGAAATATCGATATGTAAGGCGAGTTTTTCATTTTACAAAAGCCAAATTGTCAGGGCCACAAGGTATACCATTGAATCAGCTTATTGGAATTTTTGAAATTTGGGCTGCTGGTACTTTTCTGGCCATTGTTGTGTTTATTTTGGAATATTTTCAAAAATTTAAACTATAA

>IR107

ATGAAATTTACAGCAATATCCGTTGGCGCTGGGATCGATGGCTCGTTCATGTTGGAATTATCACGCCACTTGAATTTCACACCAGTGATCATTAAAGCCAAGAACTATGATGAAGCCATGTCCAAAATGTTAGCTAAAGCTGTAGGAATGTCCGTGAATGCTTGGTCAATGCGATTTCTTAACGAACATGTAAGTATGACGCACGCCATGTATTCAGACCAGAAATGTGTAGCTTTACGGAAAGGAAGTGTCTTAAGAGGATGGCATGTCTTCCTGCAGACATTTCGTTGGGATGTATGGTTAGCTATCATCTGTACGGCTATAATCACGAACTGGATAGTAGCTTTGGTCACCAGACGTAGAAGCTGGTATGAAGCTATGCCGACAGTCTTAAGGGCTATGTTCACGGTACCGCTGAGAAGACCGCCGAAATCCACCAAAGAAAGGATAATAATAGCCTCCTGCCTTCTATTCGGCATCGTAATCATGACTACATATCAGGGCAATCTGTACTACTTCATCAAGACCAAGGTGAAACACAAACCGCCTACAACTCTGAGCGAAATCAGGCAAGAGATCAGAGGACGACATCTTCCAAATGATACCACGTTAACCAATAGAGTGTTTGAAAAGTATGCCATCCGGATTAGACGCAAAATCTTTGATCTATTGATGCAAACCGGACAACTGAGTAATCTCTACCTGGTGCCGGAGTGTTTATATACGGCTAATTTTGCGTACGCACTCAGAAAGGGAAGTGTTTGGTTGGCACCCTTGAACAGATTCCTGTTGAGCATGTTTGAAGCCGGATTTCCAGAAGCGTGGTACCGTAGAACCGTGTTCACTAAGAAAAGGATCAGCTATAAAGGGAAACGGAAAACTGTGAAAAGTGCCAGAGTGTTCACTCTTCGAGACCTGGAAGTGGCCATCTTTGTATTGTTATTAGGCTTAACGCTAAGTTTCGTTGTGTTTCTGTTGGAGATCCTCTCGGCGACTGTAGCGTCGAGAAATTTGTTTCTTAGGTGGAAATTGAAAATACGTCATGATTACGTCAATTAG

>NAN

ATGGGTAATGCAGAGAGTAATGTAACCAGTGGTGTTAAAAAACAGGCGGACACTTCTTCAATTAAGATCTACAAACTGGTAGATCTTAAAGGTGGAGGACTGCTAGTGGAAATGATGAAACGAGCCGCTCAAAGTAAACAATACGCGGAACTGGATCATGCAATCAAAACAAAAGTTGAACCCTTTTTATACAATAAAGGACAAGGAAAATTGATGCCAATCAGTCAGTTAGTACTTATGAGAAATAAAGAAAGGCCACGACATAAAATGCTTCCGCCATTGAAAAATTTGGAAAATCCTGATGATTACGACATTGAATCCTATCCAGCTCCAGAAAGCAAAATAGGCGATAAACTAGATAAGAGTAGATGCCGCCATGTTTGTTGGGATCTTAAAGAAAGAGGTGCCGTTGGTGAAACCATATTACATCTTTGTCTATTGAATGCAACCTCCATCCATGCCGATTTGGCCAAACGTTTGCTCAGGTTCTACCCTATGCTTATCAACGACATATACATGTCTGATGAATATTATGGTGAAAACGTTTTACACATTGCTATTGTTAATGAAGATCCTGCTATGGTTAAGTTCCTTTTGGACAGTGGTGCAAACTTCCACGAAAGGTGCGTAGGAAACTTTATGTGCCCTGAGGATCAGAAAGCTTCTAGAACGGATTCATTAGAACATGAATGGGTCAATTTGTGCCCATTTACAACATATGAAGGTTACGTCTACTGGGGAGAATATCCATTATCTTTTGCTGCATGTCTTGGACAAGAGGAATGCTATCGGTTAATGTTAGCACGGGGTGCGAATCCAAATAATCAAGATACAAATGGAAATACCGTTCTGCATATGTTGGTCATTTATTCAAAATTGACGACTTTTGATATGGCGTACGAAGTAGGTGGTGATTTATCGATAAGAAATGTACAAAATTTAACGCCTCTTACACTGGCTGCTAAATTGGCTAGAATAGACTTGTTTTTTCATATACTGAATATAGAACGAGAAATCTACTGGCAAATTGGAAGCATAACTTGTGCAGCTTACCCTTTATCTCAGATCGATACAATAGACATCGATACTGGAAATATCAGCAAAAATTCAGCCTTAAATTTAGTGGTTTTTGGAGAAAAAGATGAACATCTGGAACTAATGGATGGTGTATTGATAGATTTGTTGAACGCAAAATGGAATGCATTTGTTAAAAACAGATTCTATCGCCAGTTCTTCCTCTTTTCATTCTACTTTTTGATTTCGCTGGTTTGTTTCACCATAAGACCTGGACCACCTCCTCCCAAACCGCCGACCACCACTGCTCCTCGTGCTACTAATGTCACAGATCAAGCTGGACTAAATACAACAGATACTACATCATTCTATCCGTACAACGGTAGGGTAGAACAGTGGTTTGAAGATTGGCAAAAATGTAGATTACTTCAGGTATCAGATATTGAGGATGTTATTAGACTAACTACGGAAGTAATGTTGGAAATCGGCGCTATACTCTATATTGTAGCAGCCCTCCGAGAAGCGAGATTTCTTGGATGGAGTATGTTTGTCGAGAATCTCATGACTGCTCCATCGAGGGTTATGTTTCTCTTCTCCTGTTTGCTGATGATGTTCATGCCGTTCTTAAGATTTTCTTGCAATGAACGAATAGAAGACATAATGGCTGTTGTTATAATGCTCACTACTGCTCCATATTTTCTTTTCTTTTGCCGAGGTTTTAAGACTGTGGGACCATTTGTTGTGATGATTTACAGAATGATAATGGGAGATTTACTTCGTTTCGCTACAATTTATTTGGTTTTTGTAATGGGCTTTGCTCAAGCATACTATATAATATTCTTATCATTTGACAATCCGAATACGCCTGAAGGTGTAGATGATTCTGTGAGTAATCCAATACCTAATCCAGTTGAAGCAGTTATGGCAATGTTTTTCATGTCTATGACCAGTTTTGGAGATTATTATCCAGCAATGGAGAAAACAGATCATGAATTTGGAGCCAAGTTGTGCTTTGTAATCTATATGGCTATAGTTGCTATTCTACTAGTTAACATGTTGATCGCCATGATGGGCAACACTTACCAAAAGATTGCTGAAACAAGAAACGAATGGCAAAGGCAGTGGGCCAGAATAGTGTTGGTGGTTGAACGAGGAGTAAGTCCTTCGGAACGTTTAACCAAGCTGATGTGGTATTCTCAGCCCATGTCAGATGGACGTCGGGCGCTTGTCCTTAGACTCAATCAGTCGGAAGAGGATAAAGAAGAAATGAAAGAGATCCTGGAGATGAAGAGGATCCATAACAGAATGGTGGCCAAAAGGAAGCAGAGAGAAGAAGAACTACGAGCTAGGGAAGCAAACGGACCTGAACATCCACAGTTTAGTACTAACTCAAATATGGACAAACTAATTGATATGAAAAGTCCTCTTGGACGAAAAAGTAATCTTTCAACCACCGCCCAATTCAATGATATGGAAAAGAGAAAAAATCTTATCAACTTATCTTGA

>IAV-FX

ATGGGCAATACTTGGTGCAGCGGAGCATCGGTGAATGCTGGCACAGTCTTGGACAGAGTCATATCCCAGGCATCCAATAAAGATGAATGTCTTCTGTACAGACTGGCGAACTATAAGAAAGGAGGTGAACTGGTAGATGCATACAACACTGGCGGCCAAGCTGAAGTGGAAAAACTGATTAAAGAACAGTTTGGAGTACTGATGTACAATGATGGCAAAGGAACGATCATCAATCGAGCGGAATATCTTCGTTGGAAATTCAGAGATATGCAACAGGTTCAAATTCCTATTGAAGCCTCCTTAAGTACTCAAGATCCTTTATCTAAATGGGAAGATCATCAGGCTTGTTGGCAAATGCAGTACAGAGGTTCCCTTGGAGAAACTCTACTCCATGTGCTTATCATCTGTGACACCAAAATACACACTCGTCTGGCACGCACTCTACTGAAATGTTTTCCAAATTTAGCCATTGATGTTGTAGAAGGAGAAGAATATTTGGGAGCTAGTGCTTTACATTTGGCTATTGCGTATTTTAATAACGAACTGGTCCAGGATTTAGTGGAAGCCGGAGCTAATGTTGAACAACGAGCTATTGGAAGTTTTTTTTTACCTCGAGATCAACAAGGATCCAGGCCAAAAAAGTATACAGATTATGAAGGTCTTGCGTATTTGGGTGAATTTCCGCTTGCTTGGGCAGCCTGTTGTGCTAATGAAAGTGTTTATAATCTATTACTAGAAAACAATGCAAATCCAGATCGTCAAGATAGTTTTGGAAATATGATTTTGCATATGGTGGTTGTATGCGACAAATTGGATATGTTTGGTTATGCATTGCGACATCCAAGAATGCCTGCAAGCAATGGCATATCGAATGAATGTGGCTTAACTCCATTAACATTAGCCTGTAAACTAGGTCGCGCTAAAGTATTCAGAGAAATGTTAGAGTTAAGTGCCAGAGAATTTTGGCGATATAGTAATATAACATGTTCTGCCTATCCACTGAATGCGCTTGACACAATTCTACCGGACGGACGAACAAATTGGAATTCAGCTTTATTTATAATACTAAACGGCACAAAAGAAGAGCATCTTGACATGTTAGATGGTGGGATAATCCAGAGATTATTAGAAGAGAAGTGGAAAACTTTTGCAAGGAGACAATTTTTGAAGAGGCTTGTCATTTTGATGTTACATTTGATATTTTTGAGTGGAGCAGTTTATCTTCGGCCTACTGACAGAAATAAACCATTACTTGGTGGAGATGACTGGCAGGATTATATACGCCAAGGATTCGAAATCTGTACTGTAATTGGTGTTCTATCATATGTCATTGTACAACAAGGAGGTGAAATAAAAAATCAAGGATTAATTTCATTTCTTACTCAATTGGACCCTGCCAAAGCTATTTTTTTGGTATCAAATCTCTTAATTTTGGCATGTATTCCATTTCGATTGGCTGATGATAAAAGAACAGAAGAGGCAATCCTAGTCTTTGCAGTACCAGGTTCTTGGTTTTTATTAATGTTCTTTGCTGGAGCTGTACGTTTAACCGGACCTTTCGTTACTATGGTCTACAGCATGATCATGGGTGATATGTTCACTTTTGGAATTATTTATTCCATTTTTCTATTTGGATTTTCTCAATCCTTTTATTTTCTTTATAAAGGATTTCCAGGGGTGAAAAATACACTATACAGTTCATATCCAAGTACATGGATGGCATTATTTCAAATTACTTTGGGCGATTATAATTACGCAGAATTGTCTCACACGACTTATCCTACTTTAAGTAAAACAGTGTTTACAATTTTTATGATTCTTGTACCAATCCTCTTATTAAACATGTTGATTGCCATGATGGGAAACACTTATGCGCACGTCATAGAACAAAGTGAAAAGGAATGGATGAAACAGTGGGCCAAAATTGTTGTCTCTTTGGAACGTGCCATAAATCAAGAGGATGCTAAACATTATTTACAAGAATACAGTATAAAACTTGGTCCTGGAGATGATCCGTCTACTGAACAACGAGGTGTTATGGTTATCAAGAGCAAAAGTAAGACCAGGGCAAAACAGAGAAAAGGTGCCGTGGCCAATTGGAAGAGAGTTGGTAAGGTAACAATTAATGAACTACGAAAGAGGAACGTGACTGGTGAACAACTAAGAAGAATCATGTGGGATCGTGCTTCAATATCAACTCCTGTCAAATTGCCAGTGAATCCGGTCATTGAACAAGCACTGGATGGAGCAATAAGTGATCAACAGCAGAATGCCCCAGCAAATGGAGGAGTATTTGGGGGTGCACTGACGGCTGCATTGGATGTCATGGCGTTCACTCACGATCTAGATCTTACTGCAGCCGCTATTTCGCCATCTCCACAATCAAAAGAAATTTATGTAAGTGACCCCTTCAGGGATTTGGTGTTAAGCGCAGAAATTGATGCTGGTAAAGATGAACTGTCTTCCCTGGCAGAAGCAGCTGTTCTATCTGTTACCACAAAGAGCAATGAAATTGAAAGCAGTGAAAAAATAATTGACAAACAGCTTAGCATTGAACAATCCGCCGTACTTGGAATGAGCGATAGTGACATATGTGCGGAGGAACCACCTTTGGGACAAGGATCTCGAGCAAGAAAAGTAAAATCAGCCCAGCAGAGGCAAAGAGCTGAAAATGGCAGCACTATTGATACTAAAATTAATTTACTAAGCTGGATGTACAAGAGTGGAAATTCAAGTGCATCTTCTGCTGATAATGACCCACCACCTCCTTATACTCCGTTGCCAACTGCTCCTCCAATCATTAAGAGGAAGTCAATGAAACGACCGAAAACTGCTAAACCAAACAGAGTCGCCCCAGAACCTGAAGTACCACCAAGACCAGGAAGTTCAGCTGTACCGGCCAGTGAAATTAAAAGACAAAACTCACCACCAGACCCATTAGAACCTTGGAGTACAAGAGAAATTACCAATATGAACGCAATATTAGCTTGGCAACCGTCTGATCAAGATAGTATGTAA

>NOMPC

ATGAGCAGCTCGACAAACGGTCGGAAGAAAAGTGCCGGAAAAGTTCCTGCAGGAGGTACTGACGACAAAGCGTCCACTGCAAGCACCGCCTCCAAGGATGAAGCTGTGGACAAGAAGGATGAAAATGGTAAGCCTGGTTCAGCGGGTGCCAGAGATGGAGCGCAGAGGATTTTGCAATTGTGCCAGAAAGGAGAATGGCCGCCGTTCGACCAAGTGCTCAAGACTTTGGAGAAGGCGGTGGCTGCAGGAGGCGAGGATGCCAATCCGACACCACTTGCTGGAATCGCTGATCCGGCGACCGGTATGACCCCGTTGATGTTTGCAGTGAAGGAAAACAGAACCACGTACATCGATAGACTGGTCGATCTGGGATGTGACGTCACCGCTAGGAATAACGACAACTACAATGCTCTGCACATTGCGGCCATGTACTCCCGAGAGGATGTGGTCAAGTTATTACTCGCCAAGAAAGGTGTGGACGTCTACGCACCTGGAGGGGCGAAGCAGCAGACAGCAGTGCATCTGGTGGCGAGTCGGCAAACAGGAACTGCAACCAGCATACTACGAGCCTTGCTGGGTACAGCCGGAAGGGACATCAGAACCAGCAAAGATGCGAGAGGCAAGATACCGTTGCTGTTGGCCGTGGAAGCTGGTAACCAATCTATGTGTCGAGAGCTACTGTCGCAGAGCACGGTGGACCAACTGAAAGCAACCACTGATAACGGAGACACGGCACTTCACTTGGCGGTCAGGCGCCGCGATATTGACATGGTTCGCATTCTTGTGGACTATGGAGCCGCCATTGATGGACAGAATGGCGAAGGTCAGACAGCACTTCATATTGCTTCAGCTGAAGGTGATGAAACGTTGGTAAAATATTTCTACGGTGTGAGAGCGAACGCTTCTATAACTGACAATCAAGATCGCACGCCGATGCATTTGGCAGCTGAATATGGGCACGCTACTGTCATAGAACTGCTGGCTGATAAATTTAAAGCGTCCATTTTTGAAAGAACAAAAGATGGCAGCACGTTGATGCACATAGCCTCGTTGAATGGTCACGCTGAGTGTGCCATGATGCTGTTCAAGAAAGGAGTCTACCTGCACATGCCGAACAAGAGAGGAGCCAGAAGTATTCATACAGCTGCCAGATATGGACATGTTGGCATTATCAACACGCTACTCCAGAAGGGAGAACAAGTGGATGTCACCACAAGTGACAACTATACCCCTCTGCATATAGCTGTGGAATCGGCCAAACCTGCCGTGGTGGAAACCCTTTTGGGTTATGGTGCAGATGTTCATGTCAAGGGCGGAATCCAACTGGAGACGCCTTTGCACATTGCTGCCCGGGTCAAAGATGGAGACAGATGTGCCCTGATGCTGTTAAAATCTGGAGCTGGACCCAATCTGTGCACATCGGACGGCCAGACACCAGTACATGTTGCTGCTAAACACGGGAACTTAGCGACTCTGTTACTACTTTTAGAAGATAATGGAGATCCACTCTACAAATCAAAAAATGGAGAGACTCCTCTGCACTTAGCAAGTCGCGGTTGCAACGCTAACGTGGTGCGACATCTAATAACATTTGTAAGAGAGACCAGAGGAAAAGATGTTTCTACTGCGTATGTCAATTCATTGACCGAAGAAGGGGCCACAGCGTTACATTACGCCGGACAAATTGCCAAAACTGAAGTTCCTTCTGATCGTCCTTTAGAAGATAAAGAAGTTATTAAACTCTTAATAGACGGTGGTGCCAATATGGATCTCGTTACGAAACAGAACCTAGAATCAGTCTTCCATTACTGCGCAATGGTAGGAAACAATGATATATTAACGGAAATGATCTCAGCTATGTCCACAACAGATGTACAGAAGGCGTTAAATCGACAGACAGCAATTGGATGGACACCGTTGCTGATCGCTTGTCATTACGGTCATATGATTATGGTCTCCAATTTACTATCCAATCATGCTAGGGTAGATGTGTTCGACAATGAAGGTAGATCGGCTCTTCATCTGGCTGCTGAAAGAGGATATCTCGAGGTGTGCGATGCCCTGCTGACTCATAAGGCCTTCATAAACAGCAAATCAAGAGTAGGCAGGACGGCGCTTCATTTGGCTGCAATGAAAGGCTTTGCAAACCTTTGCAAGTTCCTTGTGACTGACCACAATGCTGTGATAGAAGTGTTGACGCTAAGAAAGCAGACCCCGTTGCATTTAGCCGCGGCTGAAGGACAATTAGAAGTGTGTAAACTGCTGTCAGAGTTGGGTGCATCGATAGACGCTACAGACGATCAAGGCCAGAAGCCAATACACGTGGCCGCACAAAACAACTATCCTGAAGTTGCCCAGCTGTTTCTGCAACAGCAGCATTCGCTAGTATTGGCTTGTACAAAAGATGGCAACACTTGTGCGCACATCGCTGCAATGCAAGGTTCGGTCAGGGTGATTGAAGAACTGATGAAATTTGACCGAGCTGGTGTTATTTCAGCCAGGAACAAAATTACAGAAGCAACCCCACTGCAACTGGCTGCCGAAGGCGGGCATGCGGATGTGGTAAAAGTTTTAGTGAGAGCTGGCGCATCTTGTACGGATGAAAACAAGGCTGGATTCACTGCCGTCCATCTGGCAGCGCAAAATGGTCACGGAGCCGTACTGGAGGTGATGAAATCTTCTCAATCACTCAGGATATCCAGTAAAAAGTTAGGAGTCACGGCTTTGCATGTGGCGGCTTATTTCGGCCAAGCAGACACAGTTCGAGAATTGTTGGCCAATGTTCCCGCGGTAGTGAAATCAGATCCACCACAAGGTGCCAGTCTAGTTGGAGAACTAGGGAATGAATCAGGAATGACACCACTACATCTGGCAGCTTACTCTGGCAATGAGAATGTGGTCAGATTGTTGTTGAACTCTGCCGGAGTCCAAGTAGATACAGCTACTACGGAGAATGGCTACAATCCTCTACACCTGGCCTGTTTCGGCGGACACATAGCCGTGGTAGGTCTTCTGCTAAGCAGAGCAGCAGACTTACTGCAGAGTCAAGATAGATATGGCAAAACTGGACTGCATATAGCTGCAACTCATGGGCACTACCAGATGGTTGAAGTTCTGCTGGGCCAAGGGGCGGAAATAAATGCTACAGATAAGAATGGTTGGACTCCCCTACATTACGCAGCCAGAGCCGGTTGTTTTGATGTGGTCAAACTGCTGGTTGAATCAGGCGCATCGCCCAAATCTGAAACCAACAAAGGCTCAGCGCCGATATGGTTTGCTGCTTCAGAGGGCCATAACGATGTGCTAGAATATCTGATGACGAAAGAACATGACAGCTATACGCTAATGGAAGATCGAAGGTTTGTCTACAATTTGATGGTCTGCAGCAAGAAACATAATGACCGGCCGATGCAAGAGTTCATATTAGTTTCTCCAGCGCCGGTTGATTTGGCAGCTAAACTTTCCAGTATTTACGTCAACCTGTCCAATAAGGAGAAAGAACGAGCTAAAGATTTAATAGCAGCCGGAAAGATGTGCGAAGGAATGGCCACCGATCTGTTGGCACTGGCCGCAGGTGCAGACTCTGCCGGTCGCATTCTTACTGCCACTGACAGGAGAAACATTGAGTTCCTGGATGTACTCATAGAGAACGAACAAAAGGAAGTTATTGCGCATACAGTAGTTCAAAGATATCTTCAGGAACTATGGCAAGGTAGCTTACAGTGGGCCGGCTGGAAGACCATGCTTCTGTTCTTCACCTTCATCGTCTGCCCACCGGTATGGGTATACTTCACACTGCCACTTGGACATAAGTACTTTAAAATCCCCATAATAAAGTTCATGTCCTATCTGACGTCACACATTCAACTAATGATGTTCTGTTGTTTGACGGGCATCACGCCCATACACCCGGTAGTAAGATCTACAATTTTGCCGTATTGGTATGAATGGGCGTTACTAGTGTGGCTGTCGGGTCTTCTACTCACAGAACTAACAAACCCATCCGATAAGTCTGGCCTAGGATGGATCAAGCTTGCTGTTCTGTTATTTGGTGCCGCGGGAGTAGCTCTGCACGTTCTTGGCCTGGTCATAGAGCGACCATATTGGCCAACATTGTTCTACCTGAGGAATCAACTGTTTGGACTGTCATTTCTCCTGGCTTGTGTACAAATCTTAGATTTCCTATCATTTCATTATCTGTTTGGACCGTGGGCCATTATTATTGGCAATTTAATGAAAGATCTAGCCAGATTCTTGGCCGTTTTGGGTATATTTGTTTTTGGATTTTCCATGCAAATTGTGGCGATGAATCAACCATTTAAGAATCAAACGCCCGATGAAGTACGGAAAGGCGTGAAGCCTTTACCATATTCTGGAATCTTCACCGAAGTGAGGATGAATCCTTTGGAAGCGCTGGAGCTGTTGTTCTTCGCCGTCTTCGGCCAGACAACGCCGGAAAGTCTGCGCATAAAAACCCCTTTACAACCTCCATGGACTATAATACTGTTCAAAGTAGTGTTTGGAATCTACATGTTGGTCTCAGTAGTTGTACTCATCAACTTGCTGATTGCTATGATGAGCGACACTTATCAGAGGATTCAGGCTCAATCGGACATTGAATGGAAATATGGTCTAGCTAAGCTCATTAGAAATATGCACCGTACAACTACCACGCCTTCACCTTTGAATTTGGTCACCACTTGGCTGGTTTATTTCTTACATTTGTGCAAACAGAAGATGGCTCAAAGGAAAAGACCAAGTTTAGTGCAGATGATGGGGCTGCAGTCTCGATCGAGAGTGAGCGCAAGAACTAAAATGGGCGCCAAATGGTTGGCCAAGGTTAAAAAAGGGCAAGTGGCTCCTAAAGAATCGGTAGCGCTGTCTGTGGTCCACCTGAGCCCGCTTGGATCGCAGGTGAGCTTCAACAATGCCAATCGGATAGAGACCATAGCCGACTGGGAAGGTATCGCCAAGAAGTATCGCGCCCTGATCGGCGAACAATCCGACCAGCACGAGCCAATAGTGGCGGACACAGCCAACACCATTTCATCGTCCGAAACCACTCTCACAGCTCATTGA

>PAIN-FX

ATGGACGAAAGTATACCGATGTTTGGCCGAGAATGGAATTCATCAATCTGCTCTGGAAATCTTTACGCTGAATTGCTTTTCGCTTTCGAGACAAATGATATTGCAAAATTTGATAGAATTTTATCTAATTCTGAAATTGATGCGGATTACAGATATGGCGACCCTAAATATGGTACATTACTAGACCTATGCACAATGCGAGGTAACTGCGCTGAACTCGTGAAGATCCTGATGAAGCATGAAGCTGACCAGAATTTAATTAATCCAACGAGGAAGAAAGCGCCAATTCACTTCGCAGCACAAAATCCAGATTTAGAATCGTTCAGAATCCTTGTGGAGCATCCAAGAACTAAAGTAGACATACTAGACAACAATGGGAATACTGCACTACATTATGCCGTAAAACATGCAGACTGTCTCACTGCAGTGAAATTACTAATAGACAGAAATGCCGATTTGAATGTAACTAACCGTAAGGGAATGACTCCGTTACATATTGCTCTTGACCAAGGCAATGAAGAGGTTGTCAAAATGTTAATCCATGCTGGTGCAGATTTAGCGTTAGAAGGTCCAGATTGTACAACATTGCTGGAAGATTTAACTGAACGATTTCCTCAAATCAAGATACCTGAGAGGATGCAGGAGAATTTAAAACAAAAACGGGATATGACACATGGAGAATTATTTCGTTTGCTCAGAGACAGAAGATTTAACGAATTCTTGACTGGAATTAAGACGAACAAGGATCTCCTGGAAGTCAATGATGGATGCCACACATTTCTGCAATATTGCTGCCAGTTCAATTTAGATAATATTGTTGAGGAATTGTTGAAACTCGGAGCGAATCCAAATATGACTCATGTGGCTTTGAAGGATACACCGATCATGATTTCTGCACGCAAAGGAAATGTAAAGATCCTGTCTAAGCTACTAGATCACAATGCCGATCAATTAGATTTCAGTGCGGTAAATGGAGAAACTGCATTGCATTGTGTCGTCGAAGGAGTGCAATCGATTGCACAGGCAGATCATGCAGATTATCTAGAATGCATGAATCTTCTGTTGACAGTGCCTAAAAGCAAGCTGAACATAAACCAACAAGATTTCAAAGGTAATACTGCATTGCATTATGCTGCAAAGATTAGGCAGCATAAGATGGTCCTGCTATTGTTGCAACATGGTGCTTATATTGGGGTGGAAAATAGGATGCAAGAAGCTGCATGTGCTGATATTAGCTATAGTACCTTTCTAGACCACCTAGACAATTGCATAGATTCAAATGATGCTCTACCACGTGAAGAGAATTATGAAATAACCTTTAACTACGACTTCCTATTACCATTTGATTGGTCATCGGAGCGGAAAGATTGCGATGATTATGAACTTAACAGGCCTAACAAACAATACCTGCAAGCTGAAACCAAGACGCTGCTACACATCGCGAACAATCGTGAATTACGAAGCCTATTGAAACATCCAATCTTCACTAGCTTTTTGCACCTAAAGTGGTACAAGATACGCTATTTTTTCTATATAAATCTATTATTTTATCTATTTTTTTGGTTATTTCTAACGATCTATATATTAGGTGTTTATAGCAATTTTAGAAATGATAGGAATGTGACGGTAGTGGATGCAATAGAAGAAGGAAAGCTTGTAGATAAAGAGAGTAGTGTTATTTGGTTTGTTGTTTTAATTTTGCTGTTGATCCTTGCTTTAAGGGAAATTTTTCAATTATACTTAGCACCTATTAAGTATGTTTTAAATTTGGAGAATTGGTTAGAAATATCTTTAATTGCAGCAACAATTATAATATTATGTTTTGATAATGTACAACTGAAGACGAAGCACCAAGTTTCAGCTGTTTGTATCATGTTAAGTTGGGCAGAACTTGTACTCTTGATTGGCCGACATCCAGCACTTTCTGTAAATATTGAAATGCTAAAAACTGTAACACTGAATTTTTTGACATTTTTAGCTTGGTATTCAATACTGATTATAGCATTTGCTTTAAGTTTTTATACTCTATTTAAAGACACTCAATACGAGGATACCGATGAAAATTATTTCTACAATCCAGGTATATCAATTTTTAAAACCATCCTTATGTTAACTGGGGAATTTGATGCCGGTACAATACCATTTGTAGAGTATCCGATAATTAGCCATCTCTTATTTGTGCTGTTTGTATTTCTAATAGCAATAGTATTGTTTAATCTATTAAATGGTCTAGCAGTTAGTGATACCCAAGCTATTAGATCTGATGCTGAATTAGTTAGTATAATATCAAGAGCGAAACTAATATACTATTTAGAGAATATCTTAATAAATCCTCGTAAACTGTTACTATTGTTGAAACTATTAAAATTCAGTAAATATTTTTCAATTAATTTAATAGACACATTAAGAACTGATCATAAAATCCATATATTAGTTAATAAAAATTCTGAATTAATTATTGAATCAAATCAATCAACAAGTAAACTAGATTCAAAAGCCCAGTCATGTACAAAACTGCTACTGAACATCTATATGGAACGTAGGATTGTGGAGAAAGCTGGAAATATTTTAATTGAAAAACAAAATAAATTGGAAAGATTGACAGAAAAATCTAAGCAATTACAATTTTATAATGAATTCGAAAATTTTAAACAATGTTTAGAGAATTTAAGATTAATGTTGAAAGAAAATAAAGAATTGTTGTTGAGTTTTTATGATAATAAAGAGAGGATCTGA

>TRPA1_ISOFORM_A-FX

ATGGTCTGGACCAGAAGACCTCCGGAAACTGTACCAATTACAGAGGGTGAATGTTTATTCGAAGACAAGAAGAATGGGATCAAGCCTGAGGAGAATAGAGGCCATCTATGGGAAAGCAGAGGTGGTCAGCGCAGAGGTCGGGGCTACAGAATGGAAGAGAAGTCAGAATTGAAAATTGCGATACCACCTGGCGAGGACAAACCTTCACTAAAAATTGAAGGGGAAGTCTGCCTTCTTACAGATAGCCCTTTCAGGATTTTGAGGGCGACAGAGAGTGGAAACCTAGAGGAATTCATGCAACTTTACGCAGCTGATCCAATTCGGCTTAATGTTCAAGATAGCAGAGGTAGGACTGCAATACATCTAGCTGCTTCCAGAAACAGATTAAATATACTTCAATTCATCTCGAATCATGGGGGAGATTTAAACTTGAAAGATAAACAAGGAAATACACCTCTCCACTTAGCAGTTGAACAGGACGCGCACGATGCCATTGATTTTTTATTACAAAATGGTGCAGATCCTAACATGTTAAACGAGAAGAAGCAAACTATCATTCATTTGGCTACTGAATTAAATAAAGTGAAAACTCTGGAAATATTGAAGCTTCATAAGGAAAAAATTTTTGTTGATGTTGAGGGAGAACACGGTAGAACTGCTCTACATATTGCTGCTATTTATGATCATGATGAGTGTGCCAGGCTTTTGATTACAGAATTTGATGCTTGTCCTAGGAAACCATGTAACAATGGTTATTATCCAATACATGAAGCAGCCAAAAATGCATCTTCTAGAACAATGGAAGTATTTCTACAGTGGGGCGAAGCACGAGGTTGTTCTAGAGAAGAAATGATTTCTTTCTACGATTCTGAAGGAAATGTGCCATTGCACTCCGCTGTTCACAGTGGTGACTTTAAGGCAGTAGAGCTTTGTTTAAAGTCTGGAGCAAAAATTTCTACACAACAACACGATCTATCAACGCCTGTTCATCTCGCATGTGCTCAAGGCGCTTTGGACATTGTAAAACTAATGTTCGGTCTTCAACCTGCAGAGATAATAACTTGTTTACAGTCTTGTGATGTACAAAAAATGACTCCACTTCATTGTGCAGCAATGTTTGATCGAGCTGAAATTGTACAGTACTTAATACAAGAGGGAGCTGACATAAACTGTCTTGATAAGGAAAAGAGATCACCACTTCTCTTGGCAGGTTCCCGCGGGGGTTGGAGAACTGTACTAACTCTAATTAAACTTAGAGCCAATATTCGTCTTAAAGACTCCAGTGACAGAAATGTCTTACATTTGGTGGTGATGAATGGCGGAAGGCTTGATGAATTTGCTGAGGAAGTTATCAAGGCTCAATCGAAACAAAGTTTGCAAGAACTATTAAATGAAAAGGATAATACTGGATGTTCACCACTTCATTATGCAAGCAGAGAAGGGCATATCAGAAGTCTCGAAAATCTTATCAAACATGGAGCAGTTATTAATCTCAAAAATAATAATAACGAGAGTCCTTTACACTTTGCCGCCAGGTACGGCCGATACAACACAGTAAAGCAATTATTAGATTCGGAAAAAGGTTCGTTTATAATTAATGAAACAGACGGAGAAGGGTTAACTCCTTTGCATATCGCTTCACAACAAGGCCACATTAGAGTTGTAGCTTTACTTCTAAATAGAGGAGCTTTACTTCATAGAGACCACAATGGCAGGAATCCACTGCATCTTGCAGCCATGAGTGGATATACTCAGACAATGGAATTACTACATTCGGTTCATTCTCATCTATTAGATCAAGTTGATAAAGATGGGAATACCGCCCTTCACTTAGCTACAATGGAAAATAAACCAAATTCAATAGCTCTCCTATTATCTATGGAATGCAAACTTTTATACAATGATTTTGATTTGAGTGCTATAGATTATGCAATATATTATAAATTTCCAGAAGCGGCTTTAGCTATGGTTACTCATGACAAAAGAGGCCAGGAAGTTATGGGTCTAAGATCAGATAAACATCCATGTGTAACTCTCGCTCTTATTGCATCAATGCCACGAGTATTTGAATCCGTACAAGATAAGTGTATAACTAAAGCAAACTGCAAAAAAGATTCCAAATCTTTTTATATCAAATACAACTTTTCGTGCCTTCAATGTACTCGTGTCTACTCCCCTGTGGATCAGAAGAATGGTGAAACTTTCTCTCAACTAAATTTAATTCCTCTTCCAGCTTTGAATGCAATGGTTCAACATGGCAGAGTAGAACTATTAGCACACCCACTAAGTCAAAAATATCTTCAGATGAAATGGAATACTTATGGGAAATATTTTCATATTGCACACTTACTGTTCTATTCCATATTTTTAATGTTAGTAACTTTATTTGTGTCGAATTTAATGGATTTTGCTTCATCAGTTAAAAATACAAATGTAACACTGAAAGGTACAATTAATGGAACAGTAATTCCTTGCAGTGACGCTGATACGAATGTAGAACGCAAAAGATCAGGATCAACATTTGAGTATATGACTGCATTAAGCATTCTATTTTATGTGGTATTGAATGGAGGTAGAGAAATAATACAGCTTTACCAACAACGATGGCAATATTTGTTGGATCCAACAAATTTAGTTACATGGCTGTTATATATATCTGCATTTTTTATGGTTGTGCCTGTATTTTCTGGGACTTATGTCTGTTTGCAGATTTCTTGCGCATCAGTTACAGTATTTCTGACTTGGTTTACTCTTTTATTAAATCTTCAGAGGTTTGATTTAGTAGGGATATATGTTGTAATGTTCTTGGAAATTTTACAAACTTTAATCAAAGTTCTGGTTGTTTTTTCAATTTTGATAATAGCATTTGGTCTAGCCTTCTTCATTTTACTTACAGGGGTTCGTGAACATAAATCATTTGTATCAGTACCAATGTCTTTAATGAGGACGTTTTCAATGATGCTTGGAGAAATGGACTTTCTAGGGACTTATGTTCATCCATACTTAGAACCTGGAAAATCAGGCCCTACCTTACCTTTTCCATTAACATCGTTCTTTATACTTGGTCTTTTTATGGTGCTGATGCCGATTCTGCTTATGAATCTACTCATTGGTTTAGCAGTTGGTGATATAGAGAGTGTAAGAAGGAATGCCCAACTGAAGCGTTTAGCAATGCAGGTAGTACTCCATACTGAATTAGAGAGAAAATTCCCACAAGTTTGTTTAGAAAAAGTTGATAAAAAGGAGCTTATAGAATACCCAAATGACAATAAAGGAAAATTAGGGTTTCTGGATCATCTTCTAAGAATGTGGTTTTGCAATCCTTTCTCTGAAGACGGTCTAGAAATGGTTTTAGAGAATAATGACGATTATACGACGGAAGAAATTACGAAGATGAAAAGAAAGTTAAAGGACATCTCAAAAACTTTAGATTCTCAAGGACAGCTTCTGAAGTTGATAGTGCAGAAAATGGAAATTAAAACGGAAGCTGATGAAGTAGACGAAGGGGTATCACCAAAAGAACTGCAAAGTGTACCTGGCGCTGGATGCAAATGGACTTCACCTAGAGTGAGAAATAAGTTGAGGTCAGCGCTAATTTTATCAAAAACCCAGTCGCTAAAGTAA

>TRPA1_ISOFORM_B-FX

ATGGTCTGGACCAGAAGACCTCCGGAAACTGTACCAATTACAGAGGGTGAATGTTTATTCGAAGACAAGAAGAATGGGATCAAGCCTGAGGAGAATAGAGGCCATCTATGGGAAAGCAGAGGTGGTCAGCGCAGAGGTCGGGGCTACAGAATGGAAGAGAAGTCAGAATTGAAAATTGCGATACCACCTGGCGAGGACAAACCTTCACTAAAAATTGAAGGGGAAGTCTGCCTTCTTACAGATAGCCCTTTCAGGATTTTGAGGGCGACAGAGAGTGGAAACCTAGAGGAATTCATGCAACTTTACGCAGCTGATCCAATTCGGCTTAATGTTCAAGATAGCAGAGGTAGGACTGCAATACATCTAGCTGCTTCCAGAAACAGATTAAATATACTTCAATTCATCTCGAATCATGGGGGAGATTTAAACTTGAAAGATAAACAAGGAAATACACCTCTCCACTTAGCAGTTGAACAGGACGCGCACGATGCCATTGATTTTTTATTACAAAATGGTGCAGATCCTAACATGTTAAACGAGAAGAAGCAAACTATCATTCATTTGGCTACTGAATTAAATAAAGTGAAAACTCTGGAAATATTGAAGCTTCATAAGGAAAAAATTTTTGTTGATGTTGAGGGAGAACACGGTAGAACTGCTCTACATATTGCTGCTATTTATGATCATGATGAGTGTGCCAGGCTTTTGATTACAGAATTTGATGCTTGTCCTAGGAAACCATGTAACAATGGTTATTATCCAATACATGAAGCAGCCAAAAATGCATCTTCTAGAACAATGGAAGTATTTCTACAGTGGGGCGAAGCACGAGGTTGTTCTAGAGAAGAAATGATTTCTTTCTACGATTCTGAAGGAAATGTGCCATTGCACTCCGCTGTTCACAGTGGTGACTTTAAGGCAGTAGAGCTTTGTTTAAAGTCTGGAGCAAAAATTTCTACACAACAACACGATCTATCAACGCCTGTTCATCTCGCATGTGCTCAAGGCGCTTTGGACATTGTAAAACTAATGTTCGGTCTTCAACCTGCAGAGATAATAACTTGTTTACAGTCTTGTGATGTACAAAAAATGACTCCACTTCATTGTGCAGCAATGTTTGATCGAGCTGAAATTGTACAGTACTTAATACAAGAGGGAGCTGACATAAACTGTCTTGATAAGGAAAAGAGATCACCACTTCTCTTGGCAGGTTCCCGCGGGGGTTGGAGAACTGTACTAACTCTAATTAAACTTAGAGCCAATATTCGTCTTAAAGACTCCAGTGACAGAAATGTCTTACATTTGGTGGTGATGAATGGCGGAAGGCTTGATGAATTTGCTGAGGAAGTTATCAAGGCTCAATCGAAACAAAGTTTGCAAGAACTATTAAATGAAAAGGATAATACTGGATGTTCACCACTTCATTATGCAAGCAGAGAAGGGCATATCAGAAGTCTCGAAAATCTTATCAAACATGGAGCAGTTATTAATCTCAAAAATAATAATAACGAGAGTCCTTTACACTTTGCCGCCAGGTACGGCCGATACAACACAGTAAAGCAATTATTAGATTCGGAAAAAGGTTCGTTTATAATTAATGAAACAGACGGAGAAGGGTTAACTCCTTTGCATATCGCTTCACAACAAGGCCACATTAGAGTTGTAGCTTTACTTCTAAATAGAGGAGCTTTACTTCATAGAGACCACAATGGCAGGAATCCACTGCATCTTGCAGCCATGAGTGGATATACTCAGACAATGGAATTACTACATTCGGTTCATTCTCATCTATTAGATCAAGTTGATAAAGATGGGAATACCGCCCTTCACTTAGCTACAATGGAAAATAAACCAAATTCAATAGCTCTCCTATTATCTATGGAATGCAAACTTTTATACAATGATTTTGATTTGAGTGCTATAGATTATGCAATATATTATAAATTTCCAGAAGCGGCTTTAGCTATGGTTACTCATGACAAAAGAGGCCAGGAAGTTATGGGTCTAAGATCAGATAAACATCCATGTGTAACTCTCGCTCTTATTGCATCAATGCCACGAGTATTTGAATCCGTACAAGATAAGTGTATAACTAAAGCAAACTGCAAAAAAGATTCCAAATCTTTTTATTCTTTTGAAAATTATCAATACTCACCTGTTCAAGTAAGTGCTCGTCAGAAAGTACTAGGAAACCCTAATTGGCGCCCAAGTCCTCTACCTGTAGTAAATGCAATGGTTCAACATGGCAGAGTAGAACTATTAGCACACCCACTAAGTCAAAAATATCTTCAGATGAAATGGAATACTTATGGGAAATATTTTCATATTGCACACTTACTGTTCTATTCCATATTTTTAATGTTAGTAACTTTATTTGTGTCGAATTTAATGGATTTTGCTTCATCAGTTAAAAATACAAATGTAACACTGAAAGGTACAATTAATGGAACAGTAATTCCTTGCAGTGACGCTGATACGAATGTAGAACGCAAAAGATCAGGATCAACATTTGAGTATATGACTGCATTAAGCATTCTATTTTATGTGGTATTGAATGGAGGTAGAGAAATAATACAGCTTTACCAACAACGATGGCAATATTTGTTGGATCCAACAAATTTAGTTACATGGCTGTTATATATATCTGCATTTTTTATGGTTGTGCCTGTATTTTCTGGGACTTATGTCTGTTTGCAGATTTCTTGCGCATCAGTTACAGTATTTCTGACTTGGTTTACTCTTTTATTAAATCTTCAGAGGTTTGATTTAGTAGGGATATATGTTGTAATGTTCTTGGAAATTTTACAAACTTTAATCAAAGTTCTGGTTGTTTTTTCAATTTTGATAATAGCATTTGGTCTAGCCTTCTTCATTTTACTTACAGGGGTTCGTGAACATAAATCATTTGTATCAGTACCAATGTCTTTAATGAGGACGTTTTCAATGATGCTTGGAGAAATGGACTTTCTAGGGACTTATGTTCATCCATACTTAGAACCTGGAAAATCAGGCCCTACCTTACCTTTTCCATTAACATCGTTCTTTATACTTGGTCTTTTTATGGTGCTGATGCCGATTCTGCTTATGAATCTACTCATTGGTTTAGCAGTTGGTGATATAGAGAGTGTAAGAAGGAATGCCCAACTGAAGCGTTTAGCAATGCAGGTAGTACTCCATACTGAATTAGAGAGAAAATTCCCACAAGTTTGTTTAGAAAAAGTTGATAAAAAGGAGCTTATAGAATACCCAAATGACAATAAAGGAAAATTAGGGTTTCTGGATCATCTTCTAAGAATGTGGTTTTGCAATCCTTTCTCTGAAGACGGTCTAGAAATGGTTTTAGAGAATAATGACGATTATACGACGGAAGAAATTACGAAGATGAAAAGAAAGTTAAAGGACATCTCAAAAACTTTAGATTCTCAAGGACAGCTTCTGAAGTTGATAGTGCAGAAAATGGAAATTAAAACGGAAGCTGATGAAGTAGACGAAGGGGTATCACCAAAAGAACTGCAAAGTGTACCTGGCGCTGGATGCAAATGGACTTCACCTAGAGTGAGAAATAAGTTGAGGTCAGCGCTAATTTTATCAAAAACCCAGTCGCTAAAGTAA

>WTRW

ATGGAGAAAGTTCCTCTGGACAGAGCCGTTAGTGTTAACTTAGAACAGAGACCAAAAGAGCCAAAGCCATCTCACAGTATAGTGTATCGTTGGAAAAGTTTTCGATCTAAGACTTTGCCATTTCACCGCCAGGAAGTTGAACTTAGGGAATCTTCATCCCTACCTCCGTTATTTAGTGAAAACGAAGAAGATGCTTTTGAGAAGATGGAAGACGATCTTACAGTTAAAATATGTAAAGATACACTTCGGCAGAATTTATTAGATGCGATGAGAGGGTCTTCTGGAGATGTCAAGCTATTATTAGAAGTGGAACAGGGTAAAGAATTAAAAAGGTACGAGATAGGACAGAATGAAATTGATATTCTCTTTTTATGGGCATGTTTTACTAAAAGAGTTGATTTATTGCCTGTATTAGCTGGCCAAGGTGCACAGATAAATTTTATTCTTTCTTCTGAAGGTATAGGTCCGCTACACTTGAGTGCTTTTTCAGGTTGTGTCGAGTGCTTGCGTTGGTTATTAAAAAATAATTGTGACATTAATTTTTCAAAGAATAAATATTCACCACTTCATTACGCAGTTTTGGGTAACTCGCCAGAAGCTCTTAAAATTTTATTGAATAACGGTGCAAACATAAATGAAACAGTTCTACATTCTGCTGTTTTGGCGAATTCAATTGAATGTTTAAAACTTCTTTTAGCAAGAAATATTAATCCCAATGCTCTAGATCCACACGGTTTTACAGCGCTACATGTAGCTGCTGACAGATGTAATGCATCAATTGTAAAGTTACTTCTAAGTTGTAGTAAAATCGACATAAACATGGCCACACCAGAGAAAGGTAATACTGCTCTTCACTTTGCAGCAGACGGTGGTGGTACTGAGTGTATATCATTGTTGCTGAGTAACGGAGGTGATGTAAATAAGAGGAATAAAAAATGGCAAACACCATTACATCTTGCTTCCAGGTCTCAGTGCACTGATTGTGTTGAATTGTTACTTAAAGCTGGATCTGATGTGACCGCTACTGATAATGAAAATAGAACACCATTACATGCTGCAGTTGGTAAAAATCTTTCTGCTTATAGTACAGTAGAACTGCTGATCAAATGGGGAGCTGATGTAAATAGCCGAGATAGGTATGGTTACACACCGTTACATGTTGCAGCTGTGAACGAATTGAGACACTGTGTGGATTTGTTAATAATGAATGGAGCTGACGTTAGTGCGCGCACTAAAGGAGGCTTAACAGCTTTAAGCATTGTTGGAAGAAAAACGCCTGCTTCGCTGAGAACTATAAGTCGAAGATTAGATGCTTCCATTTCCATAAATGATCCTGAACAGTCTTTTAAAGAGATTGAGCTGAAGTTAGATTTCCGTGCCTTACTGCAACATTCTTCTGGAGGAGAAGTAGGTCTTTTAAAGACCCTAATAGACGAGGGACAAAAAAATATGTTAGACCATCCACTGTGTTGCGCCTTTCTTTATATTAAGTGGCAAAAGATGCGTAGATTTTATTTTACGCGTTTGTTGTTAACTGGCCTGTTTGTGCTATTTTTAACATTGTATGTAATCACAGCTTTAGCTCATAATTGCTACAATGCAGCTCGCAATGTTACCAATTCTAACCATGAACATTGTGAAAAAAATTCCGCAGTTGGTGTTTTTCTTTTGAAGAATCCATTGCTTATGGAGGTCATTTGGTATGTTTTAGTTATTTTTACAGCTTTTGAAATGTCCAGAAAAATATTCGGTATAGCTGGCTATACGTCGGCCCATCAATATTTTGCCCAATGGGCAAATATCATAGAATGGTACACAATATTGAGTGTTTTCATTATTTCATTTGTGTACACCGGGAGAACTTATGAATGGCAAAATCATGTTGGTGCATTCGCTGTGCTTTGTGGTTGGACAAACCTAATGGTCATCGTAGGACAGTTACCATTGTTTGGTACTTATGTTGAAATGTTTACCAAAGTTCAAGCCGAATTCGCCAAACTTTTATTGGCCTACTCCTGTCTTATTATCGGTTTTACAATTAGTTTTTGTGTAATTTTTCCAACGTCCGAAGCATTTCAAAATCCTTTTATTGGCTTTGTAAAAGTGTTAGTAATGATGACTGGTGAACTTGATTTAGAACCACTCTTGGGTTCTAAGGATAGCAGTTCACTTCCACTAAACATTAGTGCTCATATTACATTTGTTTTATTTATACTGTTTGTTACCGTGGTGCTGATGAACCTTTTAGTCGGTATAGCTGTACACGATATACAAGGTTTACACAAAACTGCCGGTCTATCTAAACTAGTCAGACAAACGGAACTGATTTCATTTCTGGAATTGTCTCTTTTTCAAGGATACTTACCTAAACAAATCACAGAAATTCTTAAATGGAGTGCACTTATGTCACCTGCTGCCTATCGAGTTGTATTGCACGTAAAACCATTAAATCCACGAGAAAAGCGTCTACCAAGAGATATAATGAAGGCGGCTCATGAAATTGCGCGCCACAAGAGCCATTTTTATGCTTCTTCAGTCAGATCAACAGTTAGTAAAGCAGAAAAAAATGAAATGATTCTTGAACATATGCAAAGACTTGAAAGTATTCTGGAAAATCAAAGTGAGCTATTGAATCAAATATTATCATCAATGAAGAGGCCATTGCCAACGTAG

>TRPA5A

ATGACCAGGAAGAGAGAAAGGCTGAATACCGAATTATTAAATGCCATCATTTCTTCAAATTTAAGAGCGGTCAACAAACTTTTGGACGAAGGTGCCTGTCCTTGGGCAAGATGTCGGACAACAAAATCGAGCAGTGTCCACTTAGCAGCTCTGATCGGCCAAACGGACATTTTGACAGAACTAGTAAGAGCTGGAGCAGACCTGGGTGTGAGGGACGACAACGGTGTCCAAGCCGCTCACTTGGCAGTATGGGCTGGCCACATACATATTTTGAGAATCATATTCGATATTCAACCACAACTTATAAATTCACCAATAATGCCACCTGAAATTGAAAGTTTTCAAGATGAACTGGATTCTTGGGATCATGACCATCAGAGTATTGTTCAAATGATGCCTGTGTTTGAACTGGGCAGTACGGCTCTCCACATTGCTTGTAAGTTAAACAAAGTTGAAGTAGTTGAATACCTTCTATCAGAAGGAGCGGATGTGCACATGAGAGATTGTCAGGGTCTTACTCCGTTAGATGTAGCAGGAACTTGTGTTTCCGACCAACAACTAATAAACGATCTCAGCACTATTTTAGGCAAATCTAACAACTCTGCTGACCACTCTACTGATGAACCTGATGTTTCTATTACTGTTGATGTACCAGCAGAAGGTGTAGATCAAAATTGTACTGACAAAACAATTACTCGTCGTCCTAGTAAATCCATGTTCGATGTTGTAAATGCTCTATTTGAAGGAGGTGCTAGAATGCAAGATGATAACGATATATTTTTCCTGGGCAGACACGCTTGGAATTTATCAATTACAGCACTTCACACTGCTGTAACTAATGGAGATATTGATCTTATAGATTATCTTTTGAGTAAAGGAGCAAGCTTACTGACCAGAGATGAGCACGGTGAGACACCATTGCACTTGGCCGTCCGATTGACTTTGCTAGAGCCTCTAAAAGTCGTAATTAGCTGGTTAACATTAGATTTGATTGATATGAAGGATGGTAATGGAATGACTCCTTTACATCAAGCTGTTGTACAAGAATGGCCCAGCGGCATTGGACTTCTTCTTGAAGCTGGTGCTGATGTTACTATTCCTACAAGAGAAAATGAAACAGTTTTGCACATAGCTGCCAAAAAAGGTAATGACTTATTGCTTGAAGAACTGCTTTCTTTAGAGGATTCACTTCAGGTTATCAACACTATTGATATATGGGATTTCACTCCACTTTTCAGAGCCATTGAATCTAATTCTCCTATCTGTGTGAAAATGTTGATAAAACAAGGAGCTGATATGAGTATAAAATTACCCGGGAATAAAAGCCTTCTGCATTTGGCCGCTGATGTAATGTCATTACAGACTTTAGAAATTATAACAGAACACGAGAGCATTGTTAATGAAGACATCGACTTGCTATGTGATGGAGACGACGGGAAAATGTCCCCGTTACATATCACAGCTCAGAATGGAAGTGTTGACTGCACAAAACTACTAATTAAAACTGGGGCGAATATATTTCTTCGTATGACTGATATTTATGGTTCAACTGCAGCTCATCTGGCTGCCTGCGAAGGCCATTACGAGGTACTAACTGCTCTAATCTCACACAATTCAGCCTGTTTAGACGTACCAGACCTGCAAGGGTGGTATCCACTACATGTGGCTGCCCGGTATGGGCATGGACAGTGCGTTGTTCTAATGTTACGACATGGTGCTAATATTTCTGAAAACATACCCGACAATGCGGTCACAAAATCAGCATTGGATTTGATTGTAGAATATGTGCCCAAAGGAATAAATCGATTGGAAAATTTACTTGATTCCTACATTGATTTAGATTCATGTGCCGTTAAAGATCCAACCTGTTTAATAACTTTAAATTATACATTACTGTTAGGAAAAAATGAACAGTCAAATGATGATAAACAGCTTGCTTTCTTTGACACTCTGTTAAATAGCAACAATGTACAACTTAAAGAAACCTTACTTTTACATCCATTAACTGAATCGTTTCTATATTTCAAATGGAGGAGCATGAAAAATTTCTTTATCATTCTAATAAGTTTATACGTTTTGTATACTTTATCACTTACTTCATTTGCAATGAACACATATGTGTCATACGTTCCAGGATTCAGTGTAGCTTTCAGAATCATATCAATAATTTTACTCGTTCCTATTATAATAAGTGAATGTTTACAGGCTAGTACTCTACAGCGTTATTATTTTATCGACGATGAATCTTGGCTAAGATGGACAATGGCAATCAGTGGAGGATTAGTGTGCTTGTCGCATACAAACACAATTATTTCTAGGTATCTTGCCTCCTTAGCTGTATTTCTTTCTTGGACAGAATTATTATTTCTATTTGCTAGATTTCCCGCTTGGGGTTTACATGTGCTAATGTTCGAAAAAGTAGCTTCTCATGTGTTCCAGGTCCTCTCCAGATTTGTGTTTCTCATTTTAGGTTTTCTTTTTGCATTTATGATTTACTTCAGAGGTGCACCACCATTTAATAACTTTCTTGAAGCATTGTGCAAAGTTTTTATTATGATAATAGAATTAGATTACTCTGGAGCTTTTAACGACACCGAAGAGGTTCCTCTAACTTATCAATCAATGTTATATTCACTGTTTGGTCGGCTCATGTACGTTACATTTGTTGTACTTGTCGCTATGGTAATGATGAACTTAATAGTAGGTTTATGTGTAAGTGATGTTGCACTGTTAGAAGTGCAAGGGCGCACACAAAGATTGGCTAAACAAGCTGCTTTTCTCAGTTTTCTAGAAATGTCTGTGTACAACAAAAGTTTATTATTGTGGCTACCAACTTTATTGCAAGAAATAGTAAAAGACTTCAGAGCAGTACCGGTCTCATTTACAGTTTTCCCAAATGATCCCCTTTGCCCTTTACCAACCCGTCTTAAAAGAGCATTACTCAAAAGAGTTAAAGCTTCCAAGCCAACAAAACAAATAGCTGCAACTGTTGATCTCAAACTACAACGAATCGATTCTAATGTCAGTTTGTTGGCCCAAGATATGAATGAACTAAAATTATCTATTAAAAATATTAATTCAGTGCTGACTTCGTTAATCCAACACAAAAATGATTTAGAAGTTGTCAAATAA

>TRPA5B

ATGTACAGTCTAGTGCCTGCGATACAGTTAGAGGAAGTTCCTTTCATTGAGAGGAGATTCTCAGAAGATGCTCCCTCAACCATCAGCCTGTCTTCTAAAGATTCCAGTTGTCACATTACCCAAAGAAAAAGACACGACAGGAGAAAGGAACGACTTAACACAGAACTGCTTGGGGCTGTAATGGAAAGCTCTTTTAACGATGTCGTTAGGCTACTGGATGCTGGAGCCTCTCCTGATGCAACTTGCAGGCCCAGCAGAGTGTCGGCTCTACATATAGCTGCTTTGAAAGGTGATTGTACCACTTTAAGAGAACTGTTGAGAAGTGGAGCTAGTAGAACATCCAGAGACCGTGCAGGCCGTCAGGCAGTGCATTATGCAGCTTGGGCTGGACAAGATGGTACCTTGCAGATACTTATTAGAGCTGATCCTTCGCTAGTGAATTCCCCGGTGCATCCAAATTCAGAACCAAATCATGATATGGATTGTTTGGATTCTTGGAATCACGATCATGATCAGCTAAATCACTTGGTCCCAGACGCAGTAGATCTACTCAGCACACCTCTTCATTTAGCCTGTAAAACAATATGCTATGATACCGTAGATTTACTTCTTAGTCATGGAGCAGATGTAACTTCTAAAGATGCCAAGGGACTCACACCATTAGATGTTACTGGACTTCATGCGCCACAAAATGGACATGCAAAGAGCTCAGGCAGAAGTAAAGGACAAACGAACAAGAAAAATAGCCTTAAACAGGGGTCAGATGATAATGGACTACTAATTCGTGCCTTCGCTAAAATACCAGACACATTAGGTGATAAAAAACTTAACGATACAAATGAGGAAAAACCTTCTCATAAAATAGTGTGTGCTTTGATTAAAAAAGGTGCCAAAATGCCAAAAGGCAACGTAATATTAAAAGAGGGCTCTGTAATTAATCAAAATAAAATGCCAGTAACTACGTTGCATACAGCTGTAGCTAATGAAGAACTTGATTTGATTGAATGTCTTTTGAAAAACGGAGCGTGTTTAATGACATGGAACGAAAATGGGGAGACCCCGTTACATTTGGCAGTGAAAAAATGGTTAACAGAACCACTGAAGAAAATGCTTGCATGGGATAGCGGAGGGTGTGGTGGAAATAATGGGAATTCTTCTATTGTTGATGTAAGAGATAGCCGAGGGAGAACTCCTCTCCATCTAGCTGTGTTGCAAGAGTGGCCTAATGGTATTGCCTTGTTGCTGGAAGCCGGTGCTGACGTAGCGACTACTTCTAATGACAGTGAGACTGTTCTCCATCTGGCTGCGGAAAGAGGAAATAAAGCACTATTAGAAGAGCTACTGTCAATACCTGATGCTGCTAAGGTGTTAGAATACAGAAACCATTACTATCATACCCCCCTGTGTCGTGCAGTCGAAAGCAAGCATCTACCTTGCGTAGAATTACTGTGCCAATTCGGAGCTGATGCAACTATTGCAACACCAGGCGATGTTACATTGTTGCATATGGCTGCCTCACTAGACAGTCCCGATATTTTGGGTTATCTGTTGACTCGCGAAGCAGTCAAAGTTAAAGATGTGCGAAGTAGAGAAGTAAAAGGAGGCATGACAGCTCTGCATATAGCTGCTAAAGAAGGCTACACTGCTTGTGTTCAAGAATTACTACATGCTGGGTCAGACGTATTACTGGAATCTCAAGCTGAAGGGGACCGGCGAGGCTCAGCATTGCATCTGGCTGCTAAAGAGGGTCATTTAGAATCTGTAAAAAAAATTGTCAAACATGACTGTTCCACGTTAGAAGCTGTCAACATGGATGGATGGCAACCTTTACATATAGCAGCTGGTTTTGGAAGAAGTGCATGTGTCAGTAATATGTTAAAATCAGGAGCAGATATCTCAGCTTCTGTTTGGGATGAAACTGGTCGCAAAACAGCTTTGGATATAATCATGTTCTGTGTGCCTCAACCAGTAGACTTTTTAGAAAAAGTATTGGACTCCTACATGGTTGTAAACGATCATCCGCTAAGTGATCCAGACTGTGAAGTTGAACTAAAATACGACATTTTGGCACCTGGAGGCAGATCGTGTAAACAGTTAAAAGTATTAAATGCAATATTGAACAATGGAAATCAAAATCTTCAAGAAAGACTGCTACTTCACCCTCTAATCGAAACGTTTTTGTATTTCAAATGGAGAAAGTTGAGAATATTTTTCACGTTCATTATGGCTTTACATGTGATGCTCACCTTATCACTGACAATACTTGCTCATCTTTTGTATGTTGAAAAAGGACCGTCCATCATTCTATTTGTCTGTGCAGAGATAGCAAGAGTCGGTCTTTTCATCGCACTCTGCCCCATTATCATATTAGAGATAATCAACGCCAGTCAGTTACAGCGTTACTACATTAAGGACAGTGAATCTTGGGTGAAATGGTCGGTAATAGTAACTTCCAGTATAGTTGGAGTGGTCGACCCTGCCCAGGCTTGGCCGAGACATATTGCTGCAGTGGCTGTGTTACTGGCGTGGATAGAATTACTATTTTTACTGGCCAGATATCCCAAATGGGGATTTTATGTGCTTATGTTTTCCAGGGTTGCTTCGAATGTATTCAAGGTATTGGCCACATTTATCTTCCTTGTACTTGGATTTACATTTGCTTTTCTCATCCACTTCGAAGCGGCCGAACCATTTAGAAATTTATTAGATTCTTTTATTAAAGTGATTGTAATGATGTTGGAATTTGATTATGAAAATATGTTCGAACAATTTAAAGAAGTTAATGCATTCAGCGTCGTTGGCCGAATTATGTTTGTAAGTTTTGTAATACTAGTCGCTATGGTAATGATGAATCTGATGGTCGGCCTGGCTGTAAGCGACATTGCTCTTCTTGAAACACATGGTAAAACACAACGATTAGCCAAACAGACAGCTTTTCTTAGTCTTTTAGAACTGACGGTATATAATGAACGGTTACGCAAAATATTACCAAAAAAGTGGCATAAATATTTACAAAAAATGCGTGCTGTCCCACAATGGACAATTATTAAACCTGCAAGTCCTATTAACTCAGATGTGACAGATGTTCCTAAACATATGAAGGATACTGTTCTGAACAGAATATGGACTTCACATAAAAATAATGATGACCTCGAACTTACTATCAGTCTTCACGATATCAACTACAAAATTGAGCAACTAGCTGAAATAGTACATGATAAATTGACAGATGCTTCTTCATCTTCATCATCATCTTCATGTTGCTCTAAAGCTTCTAAATCAAGTGAAAATACCTTAATGGATAAAGGTACTTCTTCAATATTAGAAGAGATACTGAAAGAACAGAAATCTATTAAAGAGCAATTACTTTCATTAGCATTGAAATGTAATCAGCTACATCCAACAGTCTATCATAATCCGCCATCCACGAACGGCGCAACACATCCTAATTATGTTTAA

>TRPA5C

ATGAAACGCACAACAAGGAGTAAAGATGTAAGAAGGAAAGAGCGGCTAAATTCAGAACTACTATTGAGCACTCTGCAATCAAACATCGACAATGTCAGACGTTTATTGCAGGAGGGAGCTAATCCTAATGCAAGATGCAGAACGTCTTATGCATCCGCCCTACACTTAGCCGCACTAAGAGGCCAGACAGATATTGTAAGAGAACTAATTGATGCCAAAGCCAATTTGAAAGTGAGGGATCGAAATGGCTGCCAACCGATACATCTGGCAGCTTGGGCGGGATACGTCACAGTTCTGAAAGTTATTCTTCTGAATGATGCTGAACTAGTGAACTCTGTGGTCAAGAGTACAGACATAACAGTAGTATTGGAAGAACTGGATTCCTGGGATCATAATCATGCTGAAATTACCCAATTGTTACCAGCGATACATCCTGGCAGTAGTCCACTACATATAGCATCTTCGCTGAGCCGTAAACAAACAACAGAATTTCTGTTAAGTGTGAATGCAGATGTTAATTTAAAAGATTGTCGTGGGCTAACACCATTGGACGTGGCAGGTTTCAAAGCTGGAGGCTATTATAATTTTATAGATCACGATCAAAACGCCTCCGGTTCTTTGGCTGATCAAAATGCAGTCTTTCAAGAGATGTATCCTTCATTAGTTGTTAACCAGTTTGGTAGTCAAAAATTTACAAGCGGAGCAAATTCAGCCGTGTCATCTAGAAGGCAATCATTTAGTGAGAATCCTGACTTTCGTATACCAAATTTATATCCGTCAATATCAATGACTGAAAAAACAGAAGAAGAAGATGATGATGAATTCAACGAAGCAGAGTTTCATTCAACACAGGAAAGTACAACAACAACGTATAACGTAGTGAAAATGCTGATAGAGTTTGGCGCTAAGGTAAAAGATGAAAACTCCAAAAATGATAAAGATTCCATAGACTGCAGAAAAAATCCAACGACAACATTGCACACCGCCGTGTTAAATAATGATTTAAAATTAATAGACTGGTTACTACAGGAATGTCAATGTGATCACATGCTGATTACAGTTAATGACGATGGAGATACGCCATTGCATGTGGCCGTTAAAAGACGATATTTTGGTGCTCTTAGAAATATGATTAGTTGGCTAGATTTAGACACTTTGAATATGACTGACAAACAAGGAATGACAGCTTTATATTTAGCAGTTGATTTGGATTGGCTAAAAGGTGTTAGCATATTGCTGGAAGCAGGAGCCGATATTAAAATTAAAACCGACCAAAACGAAACAGTTATTAATTTAGCTGTACGAAAAAATAACCCAAAACTACTAGATGAGCTTCTTTCAGCTGAAGAGGAACCAGAATTGGCATTTAACGAAGTTGATTGTGATTATTTTACTCCTTTGTTTTTGGCAATACAAAGTGGTAATTTAGAATGCGTTGAATTGTTAATTATGCAAAATGTTAATGTGATCATTAAATTACCAGACAATACGAACATACTACATTTTGCCGTAAAATATTTTCCCAAAACTGAGAAAATAATTCATTGTATATGTAATAAGGGAATTCTCAGTCCGTATCAATTATGTGTACTAATCAATTCGTACAATGATGATGGGATGACACCTTTAGAAATGGCTTCCAGAGAAGGTTTGATAGAATGCGTGAAGGCAATGATCGGTGCTAGACCAAATCTATTACTTAGAAATAAAGGAGGTTCAACAGCGACACATATGGCTGCACGGTATGGTTACGTTGATACTTTAGAACAACTTATTAAATATGATCAAAGATGTCTAGAGGTAACCAATGTAAGAGGATGGAAGCCATTACATGTAGCAGCCAGAAGTGGCAATGGAAATTGTGTTATACTCATGTTACGATATGGGGCAGATGTTGCCGCCTGTGTTACAATAGACAATACTAATAAATCAGCTTTAGATCTAATTGTTGATTATGTGCCAATGGCGAAAGAGAAACTCATAAATCTGTTTGATTCATATGTTGTATCTACGAACTATGATAAGTCTGAAGACGATCATAGTATAGTTGAAATAGACTATGCATTATTACTTGGTACTGTAAGCACAAATAGAAACACACTTAAAGAATCTGTTCAAAGTCAACATGATGCTTCATTATTAATGGATGAAAAAGGAAGACAGACAATTTTTGTAAAAACGCTATTAAATTGTAAAAATGAAAATCTTAAAGAAACTTTATTGTTACATCCTTTAGTAGAAACTTATCTTTTTTATAAATGGGAGAATTTAAAATTTTTTTTTATCATCTTGTTTTTGTGCTATTCAATTTACACTGTAGTACTGATTTTATTTGCAACACTCAATTATCTGGCTGAAGTTACAACAATCAACTTTCTCTTAAGGCCAATCATATTCATTTTGCTATTGCCACTCGGACTTACGGAAATTATTCAAGCAAAAACTCTGCCGTATTATTACTTTCAAGAAATTGAATCCTGGCTGAAAATATTTATGTTTTTAACTGGATCAATGCTTGTCCTAGTAAGCAGTGGACTGTCTGCTTCCAGATACTTGGCCTCACTTTCTGTGTTAAACTCATCAGTAGTTATACTGTTCTTATTGGCAAGGCTTCCATCATGGGGTTTACATGTGCTAATGTTTGAAAAAGTTGCTTACAATGTTTTCGAAGTATTGTCAAGATTTGTATTTTTACTCTCTGGATTCCTTTTGGCTTTTATGATACATTTTGAAGGCGAACCACCATTTGGCAATTTCTGGGAATCATTGTGCAAAGTGTTGATCATGTTAGTAGAACTGGACTATTCAGGAGTATTTGGAGAAGGAACCGAAGTCAACCTACATAATGTTGTTGCTAGATTAATGTTTGTTGCATTCGCTTTAATAGTTGCCATGGTAATGGTAAATCTAATTGTTGGTATGTCAGTAAGTGATGTTACTCAATTAGAAATGCAAGGTAGAAGTCAACGGTTGAAAAAACAGGCTAGTTTTTTAATTTCTTTAGAGTTAATAGTTTACAATGACTGGATAGTGAAGTGTATTCCACGTTGTTTGCAAGATAATGTAAATAAATATCGTAAAATTGAAACAACAAAACTGGTCCATCCAGATGATCCAAGTTGCCCTTTTTCTGTGCAATGTAAAGAAGCATTATTACTTCACGGTTATAATGATAATTTACAGCCTCTTGAAGAATGCCTTTTGCAAATTCAAAATCTCTCTAAAGAAGTGAAAGAACTTAAAGGTTTAATAAAATCTTTGGGAAAAGCTGAATCAACGGGAAAACAGTTATCGATGGGCGAAGCCTGGTGTTCGGGAAGTGGAGTGTTAATTGAGAGCAAAGATCTCGTACTTCCAGGAGCTGGCAAGTCACTTGGGATGGGGAGTCTAGAAGGCTCTGGCCGACAAGAATCTTTGGGGAGATGA

>TRP-GAMMA

ATGTTGCAGAGGCACAGTATCCACGGCATGATGGAGGAGGAGAATGTCGTCCGGCCGCACCAAGAGATGGCGCAGTTGTCGCTCGATGAAAAGAAGTTCTTGCTGGCCGTGGAGCGAGGCGACGTGGCCACTACAAGAAGGATGTTGCAGAAGGCCATGGAAACAAACTACATCAACATGAACTGCGTAGATCCGTTAGGACGAACTGCGCTGCTGATGGCTATTGATAATGAGAATCCAGAAATGGTTGAATTGCTAATTAAACACCGGGTAGAAACAAAAGACGCTCTGCTGCATGCAATCTCTGAAGAGTTTGTCGAGGCTGTGGAGGCTCTCCTAGAACATGAAGAGTCTATTGCCAAACCTGGACAACCAGCGAGTTGGGAAGCGTTGCCCCCAGACACGGCCACGTTCACTCCGGACATCACTCCCTTGATACTAGCTGCTCACCGGGACAATTATGAAATAATCAAAATTCTGCTGGACCGAGGTGCCACGTTACCTATGCCGCACGCCGTCAGGTGTGGCTGCGATGAATGTGTTACCTCCAGGCACGAAGACTCTTTACGACATTCTCGTTCGAGGATACACGCTTACAGGGCCCTGGCCAGCCCTAGTCTGATTGCGCTCAGCTCTAAGGACCCCATTCTCACCGCTTTCGAGTTATCCTGGGAACTGAGAAGACTTTCATTTATGGAACATGAATTTAAGACTGAATACCAGGAACTTCGCAAACAGTGTCAGGACTTTGCCACGGCACTTCTAGATCATACGAGAAGTTCCTACGAACTAGAAGTACTGCTTAATCACGATCCTACAGGACCAGCGTTTGAGCATGGCGAGAGGATGCATCTAAACAGACTGAAACTGGCCATCAAATTCCGCCAGAAGAAGTTTGTCGCCCATCCAAACGTACAGCAGCTGTTGGCCTCTATTTGGTATGAAGGGCTGCCAGGCTTCAGGAGACAGAACATGGTCTTACAAGCTTTACAGATCATTAGAATAGGGACCATGTTCCCCATCTTCTCCCTCATGTACATTATTGCACCCCATTCCAAACCTGGACAAACTTTGAGGAAACCATTCATCAAATTTATTTGCCATTCCGCCTCCTACTTTACATTTCTATTCTTATTAATTTTGGCGTCGCAGAGGATAGAAACGCTATTAGATTGGACCACAAACGGAGAGAGCGGAAATCTGACGGAAAGCGTAATTAAATCGGAACCAACAAAGAGAGGCTCAATGCCAACCATCATTGAATGGCTGATATTGGGCTGGGTGGCAGGGCTCATGTGGAGCGAGGTGAAACAGCTGTGGGATGTAGGGCTGGAGGATTACCTGGCCGACATGTGGAATGTGATCGACTTCATCACCAATTCCCTGTATGTGGCTACGGTCGCCCTGAGAATCGTCTCCTACTATCAGGTTCAGAAGCATAGCGACCCCAGAGCTATGGACATCCCCAGGGAGGAATGGGATACGTGGGACCCCATGCTTATATCTGAAGGACTCTTTTCTGCTGCAAATATATTTAGTTCGTTAAAACTGGTGTACATATTCTCGGTAAACCCATATCTTGGACCGCTGCAAGTATCTCTTTCTAGAATGGTGCTGGACATCATGAAGTTCTTTTTCCTCTATGTACTCGTACTTTTTGCATTTTCCTGCGGTATGAACCAATTATTGTGGTATTATGCGGATATGGAGAAGAAAAGGTGCCCGAATGCGACAACAACGTTCGATCCACGGCATCCGAATGATCCGGATGCTTGTTTAGTTTGGAGAAGATTTGCCAATTTATTTGAAACTTCGCAGACACTGTTTTGGGCAGTGTTCGGCCTCATTGACCTAGACAATTTTGAATTGGCAGGCATCAAAACTTTTACAAGATTCTGGGGCATGCTCATGTTTGGAACTTACTCAGTCATCAATATAGTTGTGCTGCTGAACCTATTGATTGCCATGATGAATCATTCCTATCAGCTAATATCGGAGAGGGCAGATATCGAATGGAAGTTTGCCAGGTCTAGGTTGTGGATCAGCTACTTCGAGGAAGGAGGCACTGTCCCGCCCCCGTTCAACGTGCTTCCATCGCCTAAGAGTGTCTGGTACTTTTTAATGTGGCTGCACAGAAGGTTCTGTGGCCAAAGTCGAGCTGCTAAAAAAGAACATATGCGCACTATTAAGCGCAAGGCCAAGCAGGCCAGCGAGCGAGACTTCCGCTACCAAAGTATCATGAGGAACTTAGTGAGGCGATACGTGACAGTGGAACAGCGAAAGGCGGAGAATGAAGGTGTCACTGAGGATGACGTAAACGAAATCAAGCAGGACATCTCCGCCTTCAGGTGCGAGCTGATCGAGATCCTCAAGGAGTCTGGCATGAACACCACCACAGCCAGCGGCTCTGCCACTGGTGCCGGCGGAAAAAAGAACAGGCAGAAGGAGCGTCGGCTGATGAAGGGCTTCAACATCGCTCCGCCTCCCAGCTCGCCCAACTCTGGCGCCGGACTACCGCCTCCCGTCGCTGAATTCATTGCATCTCTGCAGAAGAGCTCCTCCAACGATAACCTCGTCAACTCGGCACGGTTAGGTAACTGTCTGTCCAGCTTCCAACCTCTCTCCAGCTCTTCTCCAAAGCAAACTAGTGAAAGAAAGCAAGCTTCTTTCGGACATAACACTTTTACCATGGATAATCTTAGTAGGCTTATGTTTAAAGGAAGTTCTGCCAGGAAACGCCGATGGGATAGCCTAATAGAAGTGGCAAGAAGTGGTAAAGTAGGTCGATTTATATCGCAGAGCCGATCGGAGGATTCTGTTTGCGGAGAAGGCGGGCCGCCGCCGCATGGAATTCACAATCACCAACAACGTAGTCCCAACTCGGAGGATACTACGGACAGCGCAGCCAGTGGAGCCACTTCAGCGGCCGAAAGAGCATTATCCGCTTTGAGGCGTAAACGTGAGAAGTTCCACAAGTCACGCTACACGCCCAACCAATCGTCTAGTGTGGATGTTGATGTGGTGGAGGAGCGATGTGAAAAAAAAGTGCTGAAACGTGCTTCCAGCGTACCTGTCCAAGGTAGGAGCGAACGGGATAGATCGGCCGATCTTCGGCCTTCATTAGGGAGCAGCGGGCCTGTTGGCATGGGTGATAGCGAGCCAGCGCTCTCTCACACCCGCACACAACTAGCAGAGTTTAAGGATAGAAACGGCATGGTGATGCCAACTTTACCTCCCGGCATACTACCAGTCTCTGGCCACAATGTACCTTCTGGCTGGCTTTGA

>TRPL

ATGAAGGAAGGCCCGCCAGTGACTATCGGGCCTAATGGAGAGGTAGAAGTGAACGTAGAAGACACTGGTAAAAAAAATGTGGGCATGCCAAGTTTACCAAAGCCGTTAACTCTTGAGGAAAAGAAGTACTTATTAGCGGTTGAGAGAGGTGATATGGCAAATGTTAGACGGATTCTACAACGTGCTCATCGAAAGAAGCACATAAACATCAACTGTGTGGATCCGCTGGGCCGTGGTGCTTTGTCTATAGCCATAGACGCAGAAAATCTTGAAATGGTGGAACTACTTGTAGTGCTGGGAGTCGAAACCAAGGATAGTCTATTGCAGTCGATAAATGTTGGCTTTGTTGAAGCAGTAGAATTATTACTCGAACATGAGGAACTTATACATAAAGATGGCGAACCTTATAGTTGGGAGAAAGTTGATTGGAATACAGCTCAGTTTACACCAGATATTACACCACTTATTTTGGCTGCTCACAGAAACAATTATGAAATTCTAAAAATATTACTCGATCGAGGAGCTACCTTACCAATGCCACATGAAATTAAGTGCGGTTGTGAGCAATGTATTAAAGAATCGGAAGAAGATTCGCTTCGTTTCTCCATGTCTCGTATTAACGAATATAAAGCACTTGCCAGTCCTTCATTAATAGCTCTTAGTTCCAGCGATCCAATATTGACAGCTTTTGAACTATCCTGGGATTTAAGAAATCTAGCTTTTGCTGAACAAGAAAGCAAGGCTGACTACATGGAATTACGAAGACAATGCCAACAACTGGCGGTAAGCCTCCTAGATCAGACACGTAGTTCTAGCGAGCTGGCAGTTATTCTCAACCATGATCCAGAGAGCCCTCCGTATGAAGAAGGGGAACACATGAAATTGGCAAGGCTGGAATTAGCAATTACATACAAACAAAAAAAGTTTGTTGCACATCCAAACATTCAACAATTGTTGGCTTCAATTTGGTACGAAGGACTGCCAGGTTTTAGACGCAAATCTTTATTAAAGAAAATGCTGAACTTGTCAATGATTTCTCTATTATTTCCTTTCTATTGTCTACTGTATATCGTTGCACCAAATACTTCCACTGGAAAGTTAATGAGGAAGCCGTTCATGAAATTTCTTATACACGCATCTTCTTATCTATTCTTTTTGCTCCTACTGATTCTGGTATCTCAAAGAGCAGAACTGGTCGCTGTTGAAGTAATGGTATCTATTTTTGGAAGTGACGAACAGAAACGTCGATTTGAGGAGCAACAGATGATACAACGAGGTAAAGGACCTATGATTTTGGAGTGCGTAGTCGGTTTCTACGTTCTAGGTTTCATTTGGGAAGAAACAATGGAAATTTGGATAGAAGGCATTAATAAATATCTGAGGAACATGTGGAATTTCATTGATTTCACCAGGAATGCCCTATATGTAGCTGTTTTTGTGTTAAGGTGTGCAGCATATATACAGCAAAGTCAGCAAATTTCAGCTGATCCAAGAACAGCTTACATACCAAGGGAAGAATGGGATGCTTTTGATCCTCAATTGATCGCAGAAGGGCTTTTCGCTGCAGCCAACGTTTTCAGTGCATTAAAATTGGTTCACATGTTTTCCATTAATCCACATTTGGGACCTCTTCAGATATCGCTCGGCAGAATGGTTATAGATATTGTCAAGTTCTTCTTCATCTACAGTTTAGTACTGTTCGCCTTTGCTTGCGGTCTGAACCAACTATTGTGGTATTTCGCTGAACTGGAGAGGCGAAAGTGTTACTCCTTACCTGGTGGTTTACCTGACTGGGACAATAAAGGAGATTCATGTACAAAATGGAGAAGTTTTGGAAACTTGTTTGAGTCTTCTCAGTCGCTGTTCTGGGCCAGTTTTGGTAACGTGGGTATAGATTCCTTTGAGCTAACAGGTATCAAAACTTACACCAGGTTCTGGGGACTGCTTATGTTTGGTTCTTACTCCGTCATCAATGTTATCGTTTTGCTCAACCTTTTAATTGCCATGATGTCCAACTCATACGCCATGATTGATGAACATTCTGATACTGAATGGAAATTCGCCAGAACTAAATTATGGATGAGCTACTTTGAAGAATCCGCTACTCTTCCACCACCTTTCAATATCTTCCCAACACCTAAACTTCTGCTAAAGTTTCTAGGCGTACGAGAGAAAGATAAGATGCGAAGGATGTCTACAAAGAGAAAGGAACGCAAAGAGAAGGAAAGAGACTACAGATACACGGCTGTGATGCGCTCCCTGGTCTGGAGGTACATCTCAGCCATGCAACGCCAGGCCGACGACAATCCAGTCACTGAGGATGATGTCAACGAGATTAAAGGAGAGATCACGACGATGAGATATGAACTGCTTGATGTGCTGGAAAATAATGGACTTGATGTATCTTCGGCAGCGAAGAAAGATAAAACGGCTGCTTTGGGAAAGAAGATGAAGGTCTGGGAAAGAAGACTTATGAAAGACTTCCACTTGGCGCCAGTAGCAGCTGAAGAAGAAATGGATTCACTTCTCCTTGAACCACCAGCTGATGAAGACATGCTTTCTAAATTTAGGCGAATTGCTAAGTTGGCTGTACTAAGCTCGGCACAACAAAAATGGGGACAAGTGGTCAGGAGCGCTTGTAAAGCATCTCAAATTGGACATTGCAATAGTAGAGAAGCATTCAAGAGTCAACAGACTTTGCAAAAAGCCATGGCTGAAGCACAAAAGTTAGTAACACGCAGTCCAAAAGCACAATCAAGAGCACCTTCACCGTCGATGAGCTTACCGGAGACCACAACTTCCACCATCATGGAGTTGCTAAAAGATTTTGAAACTGAAGATGATATTAGCAAATCATCACAGCTCCAGCCAGGAGAAATGTCTTTACCAGGTAGTGGCTCTACTAATGTGCTGTTAACATTAAAGAAAGGGTTGACCCCTCAAACCAGTCCGAGGATTTCCAAATCGCCAATTAAGCTTGCCATACCAAAAGATAAAGGATATTCTGGTGGATCAGGAAAATGTGAAGATATCATTGGAAAATCTGAGAAAAGTACAACTGAACGAAGATCTAGAGCAGCAACACCTGAGAAAACTATGGATGTGCCTTCTGTAGAAATTAGACCACCACCTTCTGAGCCACCAGAAAGTCCAAGTCCTGTTAGTTCACCGCCTCCAGCTGCAGCTTTATCGCCACGTTCAAGTGTCAGCTTTTCGCCAGACGTTATCCTCTGTACCAGCAGTGATAAATCAGACCAGCAGAGTCCTCCAAAATTAGATTCACCCGTAAAGCCAGTTCAAATTGAAGAAGATAAGCACATCATTGTGGTTATTTCAAGTACCAGCGAGGAGGTTATTTCTGCAGAGATACCCGCCGATCAGAAATCACCTTCAACATCTGCAGTAATGGAAGCTAAAGATACTCCTCCAAAATCACCAAAAAAAGAAAGTTCTACGTCGGTACTAACTCCTCCTAAGTCGCCTGCCAAATCAGCGTATGTTTCTATACCAGTAGATTCTACCAACAGTACTAATGTATATTCATTCTCTTCTTCAAGTCAAGAACCACTGATTCCACCAGAATCTCCAGAAGTGGTCAGGGGATTTACATCTGAATTCAAACCAATCAAGCGTCAACAGAAAGAGGGATGGCTGTGA

>PKD2

ATGGAGAATATCCCGGACTTGTGGGCTTACTTGACGCACCACTTTGCGCCCTTGATTACCTTGTACCATGTAAAAAATACAGATAAGTCGAAGTCGAAAGGAAATTTAGCACCTTTAATAAAGAGTAGCATTACCATGGGTCCTGCCAGAATCAGACAGGTACGAGTGGTCAACAGCTCTTGTGCAGTACCGGATGTATTCAGTGATTGGTACAAGTTCTGTTACACTTATTTTAATTATAAGTATGAGGATAAAAAATCTTTTGGTCCAAGTGAAAATAAAGAATGGTCCTGGAGGAAAACTGATCAAGTGCTTTATTATTTTGGAAAAATGGCAATGTACTCACCTCATGGATATTACACGCAAATACCCTCCCATTATGATGCTGCCAAAGTTCTGCTAGCTGATCTGGAAAAACGGAGATGGATAGATCGAGCCACTAGAGCAGTCTTCATTGACTTCACCGTTTACTCGCCGGATGTAGATCTTTTCTCAGCTGTTAAGTTAGTGATGGAACTGCCGCCTAGCGGGGGAGTCTTCCCATCTTCAAGATGTTATTCTATGAAATTAATAAGCCCCGAGGATTGGATGGATTTATTTAAAAAACCGACAGTTTTGAAGTTTTTATACTTAATTGTTGGAGTTTTATTTGCTGCTTTTATACTTTATTTCACACTGATAGTACTTTGGGATGTCTGTTATTTTGGCTGTTCATATTTACTAACATTCTGGGGATTTCTGGATGTCAGCATTCTTTTTCTTATCTTTGGTACATTAGCAATGTTTTGTGCAAAAGAGAGTTACAGCAAAAATGTTTTTTCAAAGTTGGCAGAGGGTGCTGAGAAGGACAAACATATGAGTTCAGACATTATGATCGCAATTAGCACAGTGTACAGTGCACTTTTAGGCATACTTGTTATACTTTGCTGGATTAAACTGCTCAAGTTTTTCGCTATGTTTACCAGGGTGTCGATGATGTTTACAGCTGTGCATCAGACTAGTAAAGAGCTAAAGACACTGCTTTTTATGGGATCTATCTTAATGTTTGGATTTGCCGTCTGTGGCCATTTATTTTTCGGCTTGCAG

>TRPM

TTGAGTTCAAAACTGGATATGGCGCCTACGCCTAAGCTCAGGGTTTCCTTAGTTCCTAAAATGAGAAAGCTTAAACCAGTCCCTAAATTGACCGTCGCTGGCAAACCGTCAGCAAGGAGTTGGATCGAAGCCACCTTTACAAAAAGAGATTGCGTGAAATTCATCGCCAGCGCTAAAGATGAGCACAGGTGCTGCTGCGGGTTATCGAAAACATTCCATTGTAGTTCTGGAACAAATCTAGAAAAAAGTGATAATGATGACATCTGGATTCCATCAAGGCATACTCTACCATCAGCCACTGATGCTTATGGTACGATTGAGTTCCAAGGTGGGCCACATCCAAGTAAAGCTCAGTACGTTAGATTGGCTTATGACACAAGACCTGAATTTATTCTACAACTGTTCACGCGAGAGTGGAACCTAGAGTTACCAAAACTCCTGATCACTGTACAAGGTGGAAAAGCAAATTTCGAACTGCAACCAAAATTGAAGAAAATTTTAAGGAAAGGCCTTCTGAAAGCTGCTAAAACTACGGGGGCCTGGATCTTTACTGGTGGAACTAATACAGGGGTAACCCGTCAGGTGGGCGATGCATTGCTGATGGAAAGATCGCAACGCAGTGGTCGTGTGATCAGTATCGGTATCGCGCCCTGGGGCATCGTCGAGAACAACCACGAGCTGATCGGACACAATAAGGATGTACCCTACCACTCAATCTCATCGCCAAGGTCCAAGTTTGCAGTACTTAACAACAGACATGCGTATTTTTTATTAGTAGATAATGGTACGGCAGGTCGATACGGAGCCGAGATCATACTTAGGAGGAAATTAGAAAAATATATATCAATTCAGAAGTTACATCCATGTACCCATTGTAGTACTCCAGTAGTGTGTCTAGTGATTGAAGGCGGCACGAACACAATCAGAGCCGTGCTGGAGTACGTGACCGACACTCCTCCAGTTCCGGTGGTGGTCTGTGATGGGTCTGGTAGAGCTGCCGATTTGTTGGCCTTTACTCACAAATACGCATCGGACTCTGAATCTGGAGAGCAGACGGTGCTGGAGAATATGAAGGAATACCTGATCGACACAATAGAGAGAACTTTTGAAGTGGGCAAGGAGCAGGCTGAGTGCCTCTACGGTGAACTGCTGCAGTGTACTCGAAAGAAGAATCTGATAACAGTGTTCCGGATCAGCGATCGTGCGGAAAATAACAACCAAGAACTTGACCAGACCATTCTTACTGCGCTGTTCAAGAGTCAGCATCTCTCGCCGTCGGAACAACTTAGTCTAGCACTCACCTGGAATCGAGTGGACATTGCCCAGTCAGAAATTTTTATATATGGCCAAGAATGGCCACCAGGTGCTCTGGATGAGGCAATGATGCAAGCGTTGGAACATGATCGCATAGATTTTGTGAAGCTTTTGTTAGAAAATGGCGTGAGCATGCGAAAGTTTCTTACAATACCTCGGCTAGAAGAGTTATATAATACTAAACAAGGTCCTGCGAACACGCTGGGATACATTTTGAGAGATGTGAGGCCTCACATTCCTCGTGGTTATGTGTACACACTGCACGATATTGGTCTAGTCATAAACAAGCTGATGGGCGGTGCATATAGATCGCAGTACACGAGGAGAAAGTTTCGTCTGATCTATGCAAAAGTTATGAAAAAGTCACCGCATGTACATCGTAACAGCTCATCATTTATACGGTATTATGGCAACGCAAACTTGACGCTGAGTCTGCTAGCTGAAACCCTTCCTGCCTCCAAGGAGATGCCCCTTTTTGACTATCCATTCAATGAACTTATGACATGGGCCGTACTGACGAAGCGCCAGGAGATGGCAATGTTGATGTGGCAGCACGGTGAGGAAGCGCTGGCCAAGGCGCTGGTTGCCTGTAAACTGTACAAGGCTATGGCCCACGAAGCCGCTGAAGATGATCTCGAAACGGAGATATACGATGAACTTCGCAGCTATGGCAAAGAATTTGAGACAATTGGTCTGGAACTCTTGGACTATTGTTACCGCCAAGATGACGATCAGACACAGCAACTGCTGACATGTGAGCTGCAGAACTGGAGCGGTCAAACTTGTCTCAGTCTAGCAGTGGCGGCGAATCACCGCCAGCTGCTGGCACATCCTTGCAGCCAAATCATCCTGGCCGACCTATGGATGGGCGGCCTTAGGACCAGGAAGAACACCAATCTTAAGGTGATATTTGGCCTACTGTTTCCTTTGTACATAGCGAAACTGGACTTTAAGTCCAAAGAGGAACTTCAGTTGATGCCTCAGACTGAGGAAGAACATCTGACCGGACTTGAAGAGGAGAATGACAGTGTTGACGGCAACAAAACTATTCTGGACCAGAATAAGACAAATTCCGGCCAGGATGTCGAGGTGCTAATACAGCAAGAATCGCAGACTCAGGTTCGAGACACAATAGTTACCGAGAATGGCCGTATTATAACTGACAATAGTAGCAGTTCTGACAGTGGAAGTCAGTTTCTTCCGCCACCTCTGGAATATTATGATCTGAAACCAACTAGACCTTTGCGATTGAAAAGGAAATTATATGAATTTTATACTGCACCAATAGCGAAATTTTGGGCCCATTCTGTTGCCTATGTTGCATTTCTACTAATATTTAGTTATGTAGTACTTGTTAGAATGAATCCCGACCCTTCTTGGCAGGAAATCTACTGCATTGCCTACATTTGTACTTTAGGATGTGAGAAAATAAGGGAAATTGCTTCATCTGAACCTGTGGCAATCAGCCACAAGTTCGCTGTATGGGCGTGGAATATGTGGAATCCATGCGATGCAGTTGCAATAATATTTTTTCTTATAGGCCTATCACTAAGACTGAAACCTTCATCGATGTCTGTGGGACGTGTCATGTTCTGTGTCGATATTATATATTGGTATCTAAGAATTTTAAACATTTTGGGTGTAAATAAATATTTAGGACCACTAGTGACAATGATGGGCAAAATGGTCAAGAATATGATCTACTTTGTTGTCCTATTGCTGGTTGTACTCATGAGTTTTGGAGTATGTAGGCAGGCGATACTCTATCCGGACTCAGATCCAAATTGGTCATTAGCCACAGAAGTATTTTATCAACCGTACTTTATGTTGTATGGAGAAGTATTTGCGGACTCTATCAACCCTCCGTGTGGAGATGAGCCCGGCATGGATGGCTGCCAGACAGGACGTTGGATTACAATCGTTGTCATGTCTACCTACCTGTTGGTCGCAAACATTTTACTAATAAATTTGCTTATTGCAGTCTTCAATAATATCTTCATCGAAGTTAATGCAATCTCACATCAGGTTTGGATGTTCCAGAGGTTTACTGTCGTCATGGAATATGAACAAAAGCCTGTACTACCTCCTCCATTTATAATATTATGCCATCTATATCTGTTGACTAAGTTTTTAAAGCGAAAAGTTCAAGGTGTCCAGGAAACCTACGATAATGGCCTCAAGTTATTCTTAGACTGTGAAGATCTAGAGAGACTGTATGATTTTGAGGAGGAAGCTGTTGAAGGCTACTTTAGAGAAAAGGAAAACAAACTTCATCAGTCGACCGATGAAAGAATTAAACAAACGACTGATAGAGTTGAAAACATGTACCAGAAGATTGAAGATATTAATCACAAAGAGAACTCACAAACGGCGTCCATACAGGCATTGGAGTTTCGTCTGCGGAAACTAGAAGAGCTGTCCGAACAAACTGCTTCTACACTTAGTGTTATCCACAGATTTATGACAGTGCATAGCCGTACTGGAGGTCCGAGTAACTTTGCAGCTGGAGGTGGTGGTGGAACAGGTGAGGAGGTTCAGTGCCCCAATGGCGCTTCCGTGGTGGTGGACAGACCTCGTCGAGCCAGCGAGCATTCTGACGTTCTTTCAGATATATCCGAGCAATTATCAGAAGAAAGTACAGAACAGACTGTGGGAGTGGGTAGCGGGATCACAGGCTCGCGGGAGAGTTCAATGTTGTTTACGATGGGCAGACAGCTGTCTCAGACCCACTCTGAGCCGGATACCGGCACTGGAGACTCGGCTAGCGGCGGTCCGGGCAGCAAGCATCTGGTGGAGCGCTCCGTAACTTGGGCTGAACCGAGAATCAAGGTGATACCGCCTGCATCGCACCCAAGGTCGGCACTACTGTCCATGCATCACGAGTACACAAGTATCACAGACGAACTGGAAACTGTATGTGGTCTTTTAAGTCCTCCCAAATCGCCCAGCGGTCTGTTGACGCCACTTGGCCATCGCACTGAACAGAATTACACGTCGCCCAGACGATCCCGTCATTATTCAGAGATGTCTAATCCAGAAATGGCTAGATATCTAGAAAAAGAGACGTTACGCGATGCAGAAGACTCTGATTACCAATTGATGGAAGGTCTAATTCAGAGGAGGTTGAAGTCAACAGACGAGGATAAATTCCACAATAACGCGTTCTTCTTGGCCACCACACCCGACCTAGAGCGCTATCAGGGCCGTCTTCGGCGAGCCAGTGCCATTGAAGGTGATTTTCCTTCTGGACATGTATCCGCTGTGGGCACGCCAGCCGGATTAAGTCCCGACGAAACCACAATCCATAGAGTGCCTAGTCAAACTGACCGAATTGACATGAGTGGGTCGTTGATGAACCAGCAAGCTACAACTAGCGGAGGAGAATCGTCTTCGAATTTTGGACAACCGCACACTGCAAACGATGTGACACCAATCTCAACCAACGAAACTGAGACACAAATTAGGCCAGCGTTTTCTGCTTCGAGAATTTTTAATAAAATGCAGAAAGAAGTTCAATTGTAG

>TRPML-FX

ATGATGGGAGAGGACGACCTTCTTATTGATGTTACGGCGACTCCTTGCCCTGTACAAGGCTCCACATCTTGCGTTGAAGACGAACCATTAGATATTAAATGTTATGAAGCAAAATTAAGAAGGAAGTTGAAATTCTTTTTCATGAATCCAATTGAAAAATGGCAGACCAAGAAAAGATTCCCATATAAATTCTTAGTTCAAATAATTAAAATATTGTTAGTCACTTTTCAGTTGTGCCTTTTTGCTCATAGTAGGTACAATCATGTGAATTACTCTTGGGATAATCGTATTACGTTTTCACATTTATTCCTCAAAGGATGGGACCCTACAAGAGAAGTTAACGCCTATCCGCCAGGACTAGGACCACTAGCACTGTATAATAAAAATGACTTCTACGAAACGATTGATTACGCCATTAGAGGATATAAAAACATTTCCGATGCTATCGGGCCATATTCTTATGCTAATGAAGAAAATAAAATTGTTCCTCCAGTGATCTGTTTACACCTCTACAAAGAGGGTTTAATTTTTGGATTCAATGAAAGCTATGTATTCAACAGTGAAATTACAGTTAAATGCTGCAATCTTTCCATTTTTGGTCATTCCAAGTTATCAGTTGGACTTTGTCTTAATAATTATAGTATCCAGTTCTCTGCTCTTGTCAAAGCAACTGTCAAATTTTCATTGAAAACAGTAAATTTTAAGACAGCTGGCCCATTATCACAACCCGATTGTTACAAATTCAATATAGTGATTTTATTTGATAATGGAGACATGGATGGACAGATGGTATTATCGCTTGATGCCGAACCTGTTAGGTTACAGTGTAAAGGTGATATTGAATATATTTCCGACAATAACATCGACTACATTTTACGAAGTATATTAAACTTCTTAGTTATACTAATCTGTGCATTATCTTTTTTACTATGTATCCGTGCAATAATAAGAGCTCAACAACTTAAGTATGAGACTGTTCATTTTTTCAGAAATTACTACAAAAAAGAACTTAGCCTAGAAGGTAGATGGGAATTCTGGAACCTATGGTATATTATGATCCTAGTTAATGATACTTTGATTATAATAGGCTCCTGTATTAAAGAAGAAATTGAAAGAAAACAGTTTGTGGGCGATCAGTGGAATACATGCAGTGTTTGCTTGGGCACAGGAAACCTACTAATTTGGTTCGGAGTACTCAGATATTTAGGATTTTTTAAAACGTACAATGTTGTGATATTAACGTTGAAAAAGGCTTTCCCTAAAGTTATAAGATTTTTATCTTGTGCAATACTCATATACGCCGGATTCACATTCTGTGGTTGGTTAATTTTAGGTCCATATCATATTAAGTTTCGCACACTAGCAAGTACGTCTGAATGTTTGTTTGCTTTAATTAATGGAGATGATATGTACGCAACCTTCTCAATTATGGCTGTGAAATCCTCAGTTCTATGGTGGTACAGTAGACTGTACCTATATAGCTTTATAAGTTTGTACATTTATGTTGTTTTAAGCTTGTTTATCTCAATTATAATGGATGCTTATGAAACTATTAAGCTATACTACAAGGAAGGTTTTCCGAAGAATGATTTACAAATTTTTATGACAGAAAGGGCTGATGAATTGTTTTTTAATCGTGATGATGATTTGTCTACATCACCATCAGATGTATCACTTAAAGCTTTAATTACTGGATTTTTCTGCTGTAAAAGGACGAACAGGGGCTCGTCAAGGGAATAA

>PPK-like7

ATGCTTAGAGTGTATCGTTTTGGTAAAACCAAATTTGGTAGATATATCTACGATTTTGGTGATGACACAACGCTGCATGGTCTGAGATATATACTGAAACCGAACAGCCATTTCGCTGAAACACTGCTGTGGCTAATCATGGTATTGCTGAGTCTATCCGGAGCGATATCACTGATTCTCAACTCTTGGAATCGCTATAACGCCAATCCAACTGTTATCTCTTTGGAGAAAGATTATCGAGACTGGAAGATAACCTTTCCTGCGGCTACCGCTTGCTTCTTACAACGGTTAAATGAGACTGCTGCAGTTGAATTAATCTATGATAAATGGGGCATTGATGAAAATTCAACGGAATTTAAAGAATATTCCCTATTTTTAAACACTGTAGCCAATTTAACATATGATAATTTAAAAGATTTTCAACAATTTATTGGCAACAATGATTTGAAAAAAATGACTGGAGATGAAATTTTAGAAATTGTAAAAAAGGTAACCACCGATATGGTATACCGTGCAAGCTTATACGACCGCAAACATAAAGATATTAAATTCCATGAAACTTTAACCGAAATGGGAATCTGTTATACCTTTGCTGGAGTGGTACCAAACTATATTACCCTGAAGAAGGATCCAGTGACTACAAATTTAAACTTACCCTACTGCAATTACCTGAACTCTTTGTGCTACGCTAGAATTGAAGATTTGCCTACACCAGTTAAGTATTATATTCACTCTCCGAATGAAATTCCTGACAGTTCTCACAAATATTTTGTAGTCTACGTTAACATGGAAAAAGATACTGGCTTTAGATTTCAGGAGACAGTTGCCTCACCGGAATTACGAAATCTTGATATACGTCAAAGGAATTGTAGATTTGTTGACGAACCTTTGCAAGGTGATAAGATCTATTCCTATAATCTTTGCATAATGAGGTGTCGCAAAAGATTGGCTATATCATTCTGTAAATGCCTACCCTTATTCTATACCAAAGAAGATGGTGTAGAAATATGTGGAGTTACTGGCCTGACATGTCTGTCCAAATATAGAGACAATATTCAACAACTTATCGATAGAAATGGTAAAAAAGTCGAATGCCCTTGTTTGTACCAATGTGAATCTATTGTCTATTATTTAGATAAAGATACTGAAAGAACATGGACATATCCGGTACCGAAGAATATCAGATTCCGTTGGTCAATTGAACGCTACAGCAAGACTAGATTTCGCAGAGATATCATCTTTGGAGTTGAAGATCTTCTTGTTTCGCTTGGTGGAATTGCAACTTTTTTTCTCGGATGCAGCGTCATTAGCTTCGTAGAATTAGTCTATTATTTCAGCTTGAGATTGTTTTGGTATGTAATGAGTTTGAAAAAAATAT

>PPK-like3

ATGATCACTCATTACAGAAAATTAAAAAGAAAATCAAAAATACAAAATTTTCTTACTTCAGCTTCAGATTTCTTTTTAATTTTTATGGAAACTTCTTCAATTCATGGCTTTAGCCACATTGTTACCCGCAAAAGACACCCATTAGAATATTTCCTGTGGATATTGACGGTCATCCTGGCCGGCACTGCTACTGTCCAATTGAATCTGTCGAAATGGCGGCAGTATCAAGACAATCCTACGGTAGTTTCCTTGGAAAGAAATTATCTAGATTGGAATACATCTCTTCCAGCTGTTTCCATTTGCCCTGAGGCGAAGTTGAATCCTAGCAAACTAGAAGAAGTTATTAAAAAGTTTTCCTACGTTAAGGATGAAAAAAGGCTGAAGAATTTCTTATTATCATTAGTTTTTGTTTCTTACAAGAATTTTGATGTTTTTATGGAAACATTCGACGAATTGAAAACAAGCGATTACTGGAAAGCTGTGGCTATAGTGAAACATGATTTAACCTACAGTGTGATCAACAGCAATGCAGAAAAATATGATATAACTACTATGTCCCCGTTTATGTCTGAATCTGGTATCTGCTATTCTTACAATTCAGTTGTCACTCAATATAATGATCCTAGCTATTGGGAGTCTAGAAATTGGAGTATTGTAGAAGCTCCTGCAGCATTTAGAGGGAGTCCACTAGATGGGGACATATTCGCCCAGATTATGAGTATGAACACAGGATATAGACTGTATATTCACGCACCTGCAGAATTCCCTGATGTTGCGCTAAGTAGCATGGTAGCTAAGAACAATTCATTCAAAGAGCTAGAAATACAAGCACTCAATATCTACAGCACTGAAGCCACCTTGCAATTGAGTATTAAACAGAGGAAGTGCAGACGTCCTGAGGAATCTAATTTATTGCTAAGTCCTATTTATACGTACAACATGTGCAGAATGGAATGCCGCATGAAGAAGTGCTGGGAACTGTGTGGTTGTGTCCCTTATTTTTATAGACCATTAGAAAACTTTAAGATATGTGATATTAAAGGGATGAAATGTCTTAGCAAACATAAAGAACTATTAGTCAAACTGAGAGACCCTAAAACCGAGAAGAAAATAAAATGTGACTGTCTTCCTCAGTGCGATGAAATCAACTATCTAATTGATAGCGAAAGTACAATGAATTGGGATTTTGGAACAACTCTAAAGTGGGGAGTGATCAGATATCCAAGACTGCGTTACAAACGAGACATAATTTTCGGCTTGTCTGACTTACTGGGAATGGAAGAGCTCGATGTCATCTGCTACTAA

>PPK-like4-FX

ATGAATCTCTTACTGAAAATGTTGCGCTCTATTTTCAATGCTTTAAGATATTATTGTATTAATACAAGTTTCCACGGTCTCAGATTTATTGCTGAAGGTGAAAGACACTGGACAGAAAGGCTCTTGTGGCTAACTTTGTGTCTGATATCTTGGTTAGGATCGGGAATGTTAATCTCTTCTTCATGGGATTCATTTCAAACGAATGCTGTCAGTTTTGTAGTTGAGACTAGTTACTTAAATACCAATACTACATTTCCCTCAATTTCTGTCTGTGAAGAAGGAAACATGCAAAAAATCTATGACATATCTCAAGAGATTTGGGGAGTTGATCATGATAATAATCTAGACGAAATACTGAAAGAACTAAGTTATTTTCGTGGAGAAGTTTATTACTTAAAAGAATTCTGTTTTTCAGGGGATCTTGATTGTCCTAAAGATAATTTCTATGATTTAATCAACAAGGCAAGAAGTAATTGTAGTGAAATATTCAACGCTTGTTTTTGGAAAAGCAAACCATTCGATTGCTGTACCCACTTTGTAAAAACAGAAACCGAATTAGGTCCGTGTTATATGTTCAGCTCTAATAATGAAAGGAAACTTTGGAAAAATGGTGATCCTTCTCACATTATAACTGTTTCAAGTATGAAATCAGGGTTAGCTATGTTATCTCTTCAAATCAACGTACCTGGTCAAATTTATATACATTCAGAATCAGAACTTCCTTATTTAAATACTTTATCAACAGATATATTAAAAATATTTCAAACCCCAAGAGATTATAAACATGCTTTTGCCGTAACTGATGTAGAAAATCCACCAGAAGTTAGAGAACTTAGTATTCATCAACGTAAATGTCGTTTTCCTGAAGAGAATTATTTAGAGATTGCTGATGTTTATGCTTACAGTTCTTGTGAGGTAGATTGTCGTAAAAAAGAACAATTACGTTTGTGTAATTGCTCTTCACATTTGATGCCCAAAACGCCAGAGTCCGAAAAATGTGACTTAGATGGTATACTTTGTTTAAATAATAATGTAGCTGAATATATCGGCCAAAAAACCAAGTGGGGAGACAACATTGGCCTGTTGTGTGATTGTTTACCAGCTTGTAATGATGCTCAGTACAATACAGTTACTTCAACTTCTAAAGAAAGTACAAACAATTATTCAACCATTGAAATATTTCTCGATCGTCTACCCAGTGAGCGTTACAAACGAAATGTGGTTAGAACACGACTTGATCTTGTTGTATCAATGGGAGGAGCAGCTGGTCTGTTTGTAGGAGCAAGTTTATTAAGTTTTGTTGAAATTTTCTATTTTTTCATTTTGCGCACTCACAAATCGGAAAAATATGATGATAATGATCAGACTAATGTTAAAACTGATTCGGAAGAAAATGTTTTAGCAAGATCAGATCAGTTTTCCTTCACTAGAACAGCTTCAAATTTGAATCGAAATGAATTAATAATCAGACATCCTTTTATACCTCAACAAACATTATTTCTTTAA

>PPK-like5

ATGTGGAATGGTACACCATTTGATTGCTGTTCATATTTTTTACCAATCACAACGGAAACAGGAGTGTGCTATTCGTTAAATTCTTTACATACAAAAGGAAAAAAATTAGATCTGGATTTAACTTCTAATCGAGCTACAGGACCTGGACGTGTGGATTTCATATTGTACGAAGCTGCCAAGGTGTATATTCACGCTCGTGAAGATGTACCAAATATTAATCATCCTCTGGAAGAGAATGCAGTATTGAACTGGGGTACCATTTTTACAATGAGATTTCAAGTAAACGAAATTGAAAATGATCCAATACTGAAGGAGGTATCAGTTGCTCAAAGAAATTGTAGATTTCCTACAGAAAATATTCTCAAAACTTATAATCATTATAGCTACTCTACATGTGTGGTAGATTGTAGAGCCAAGGCCCAAGTTGAGTTTTGCAATTGCACTCATCATTTTATGCCGAAAATTCCTGGTGTGAGTTCATGCACCACGACGGGACTTATTTGTCTAACCCAACATTCAGAAATACTTAGAGCTTTGAGAACAAAGGATAGTGACAAAAAGGGTTTGGTATGTGATTGTATGCCTAGCTGTGAGGAACCGGAGTATACAGTCATTTCAAAAATTCGATCTACCAATACTAATTATAATGAGAAAAATTATGGATCTAAAATATCTCTTTTTATGGACGCATTGCCAACGCAAAGATTCAAGCGCAATGTGGTAAGAACAAGGCTCGATCTCGTTGTAAGCATAGGAGGCACTACAGGTCTTTTTTTGGGCGCGAGCCTACTTAGTACTGCTGAATTTATTTACTTCATGTTTCTGCGCAAAAAGATTTTCATAGAAAGTATTGATGAGAAAAGTAAATCAGCAAACGATTCCAGGATATTTGTAAATTCAAGAGCGACAAGAAATAAATTAGATCCATCTAAATTTGTAGCAATGCAAAGAAACAGGTCGAAAAATAATCATGTACGTTTTTATCCGTACATTTCTTCAGAAAATTACCAATGA

>PPK-like9

ATGCTGTTTGAGTTTCCTAGTTCATTCAGGGCCAATCTGGAGTACCTTCAACGAAGCGTGGCAGTCCAAACTGGTATAAACTTTTTTTCAGGGATGCATCGTGCTTTAAGAAAACCATCTGTTTCCACTTACAAACTACTAAAAACTTCACTAAAATATCAAACAAAGGAATACTTCAACAATTCTACCCTACATGGTGTTCGATTTATCGCTGAGGAAGATAGACCAACTTTTGAAAGGTTGATGTGGTTTTCTTTTGTGTCAATCGGAGCAATCGTTACACTGATTATTATTACATCATTATGGGAGAAATTTCAAACAAATCCGACTATAACAGGACTTGATACCGATTTTCATAATTGGGATGTTCCATTTCCAGGAGTAACTGTTTGCCCTAAAAATAGAGCTTCTGATAAATTAATTAAAGCGTACATTGAAAAAAGATGGACGACTAGCGATCTTAGTGAAGAAAAAAAGAAATTTTACTATGATTATATCAATCTTATATCAAATTTAACCTACTCCAACTTAAAAGAAATAGATAAATTCAAGAATGATAAAACATTACCACAAGAAAATTTAAGAAGCGTACTACTAGCAGTTGTTATGCGATGCGAAGATCTCATTAACAATTGCGAATGGAAAGGTAGAAAATACGACTGTTGTTCAGGTTTTCTTCCAACTTTTACTGAACATGGTTTCTGCTATTCTTTCAATACTGTGCGCGCTGAAACTGACTGGCCATGGAAAGCGAAGAATTCATCTGTAATAAATCTTACTACACATTACATCTACGAAACTGACACCCGTTGGTCTGTATTGTTTAATGGAAATGCTAAGAAAAACCCTTACAGTATTTACATTCATTCTTCTGACGCACTTCCTAGTATAGAAATGCACCCACAGCATGTTTGGACGAAAACAATCAGCAAAATTCAGTTCGCATCCAAAACTACTTACACTACTGAGGAATCAAAGCAGTTGAGTGCTAAACAAAGAAAATGTGTATTCCCCTATGAAGTCAAGTTGGCAACAGCACCAGAATATTCATATTGGGCATGTATGTCAGAGTGCAGGATGAAATTCGCATTGAAAAAGTGTTCATGTGTACCATATTTTTACCATAAAATTGATGGATTTCCCTACTGTAATCTTGATGGGCTTGTGTGCCTTTCAAAATATACTGACGATTTCAAGAAATCTTTATATTGTCCTTGTGAACTTGGTTGCATGAACACCGTCTATGAAGTAGAAAAACTGGTGGACTCTAATGAAAAGGCTAAAAATCCGGTAGAAATTGGATTTGTGTCATGGCCAATGGTGAGATACAAGAGAGAAGTACTCTTTGGATGGGTAGATCTACTCGTTGCTTTCGGAGGGATAGCTGGCTTATTTCTCGGCTTTTCTTTGCTATCAGCTGTTGAAATCGTTTACTACTTCACAATGCGAGCTATGTGCATGATGTACAAAGATAAAAATAGGGTTCAGCAACTGGCCGAAGAGGAAGAAAACAGAAAAGTACCAAAAGTTGACCTTAGTCTCAGACCCAGCTATCAATTCTCCAAGAAATTTAATCAAATTAACCAACACTCTGTTAATAATAATTTACAAAGTTCTTCAACTAAGCAAAACATTATGACTTTACCATTTCTTAATTAA

>PPK-like10-FX

ATGCTTGCTAAGAAAGGAAGTAAGGGAAAAATAACGCCAAGCGGAAGTGTTAAACATCATTTTCAAGACTACTGTGCCAGCACTTCCATACATGGCGTTAAATATCTTGGTGAGGAGGAAAGACCTCTTGCAGAAAGAATATGGTGGTTATTGGTATTTATAATTTGCCTATCCGGCAACTTTTTCCTAATTGGTAAAGTATGGATTAAATGGCATGAATCTCCAGTAATTGTGTCATTTGCTGAAACAACGACACCTGTATGGCAAGTTCCGTTCCCCGCGGTAACGTTATGCAGTGAAACAAAATCTAGATCAACCTTGTTTAATTTTACTGATGCAGTGAATAGAAATTTAACTGAAGAAATCGATGGAGAAATGTTCAACAAAATGTCAGATGTTTCACTGCTTTGTGATAACCATGTAATTGTAGAAAATGGAAGTTTAACTACTAATGAATCTACTATTGAATTTCTATTTGAAGTAGCTCCACCATTTGAAGATACGGTACATATTTGCAAGTGGAAAGGTCCTGCCACACAAAATTGTAGTCATCTTTTTACGCCAATTATAACAGATGAGGGTGTCTGCTTTTCATTCAACATGCTTCCGACAGTTGAGCTATTTAGAGGGCAAGGGATTCCGTACTTTGAAGACAATGGCTACAGAACGGAAGGATGGACACTGGAGGGTGGATATCCGCCTGATGCCCCGCTAGATACCTATCCACATAGAGCACTGACGGCTGGGGCAAAAGCTGGACTTATTTTTGTTATGAAGGCAATGTGGAAGGATGCTGATTATTACTGTAAAGGGCCTGTACAAGGATTTCGAGTATTACTTCATAATCCAGCTGAGGTACCAAGTGTTGGAGAGCGCTATCTCAGAGCACCACTACGGCAAGAGATTGTAGCATCGATTCAACCAAAAATTATGACAACCAGTAAAGGTTTACGTTCTTATTCACCGGACAGAAGACAATGCTATTTTCCATCCGAACGCTATCTAGCTCATTTCAAAGTATATACACAGAGAAATTGTGAATTGGAATGCCTTACAAATTATACTTTAAAAAAATGTGGTTGCGTTGGATTACACATGCCAAGATCAGAATCAGTGAAAATTTGTGGTGGTGGAAGTAAAGAATGTATGTTGGAAGCTAATGATCTACTAAGACGTTACGAAATAGAATCCTCGTATAAGGAGACCAAACAAGAAACATGTGACTGTCTGCCCGCTTGCACTTCGATTCAGTATGATGCTGAAACTTCTCAAGCTGATTTTGAAGTTGAAAACGTATTTAGATCATATAATGACAATCTTACAGAAATTGTTGGAAGCAGCATGGCTAGAGTGTCAGTATTTTTTAAAGATATGCAGTTTACTACAAGTCGGCGAAGTGAACTTTTTGGCCTAGTCGATTTTCTAGCTAACTGTGGAGGTTTATTAGGTCTTTTTTGCGGGGTAAGTCTTTTAAGTTTAGTAGAAATCATTTATTATGTTACGCTTAGATTGTGGGGAAATATGAAGGCTACAAAGAAGACCAGTAGTAATGAAGCAGATATTGAAGCGGAACTGAGTAAACATGTCAACTTTAATAGTATAGCTTCTTCAAAATTAGTACATGAAGATTGA

>PPK-like8

ATGAACGTAAAAATAAAAAATATTAAGAAGCATTTAACTAAAACATTTCGTGCTTACATAAATTCATCAACATTTCATTGCATCAAATATATCGGAATATTAAATTTACATTTTATTGAAAGAATATTTTGGTTGACTGCCATTGCGCTTTTTTTTGCCTTATGTTGTACCGTGAGTTTTTTCATATGGGAAAAATACAATCAAATACCAACTGTGGTAAAAATTGAAGACCCTCATTTACCGTTTACAAGCGTGGCGTTTCCCGCAGTTACAATATGCCCGGCAAATAGATTAAAACCATCTTTGGCTTATGCTTATATCATCAGATATTTAAATAAAGCAACCACAAATAAAGAGGTAATAATGAAACATTTAGTAAGCGCTCTTTCGCTACTTCAATACCCGACATACATTCATATGGAATACCATCTAAAGAAAGTCATTGGTTACCTCCCATTATTCGATTCCTTAAATATCACTGACCTAATGGTAAAGTCTCTGCCCGCATGTGATGAAATAATGAAACATTGCTATTGGCAAGGAAAAAAAGAAAATTGTTGCAAAATATTTTCACTGCAAAGAACTGAAGAAGGATTTTGTTATTCATTTAATTCTATGTTATCAGAGGCCAATAAATTTAGCCCAGTTTATGTACCACCGATCATGTTGTACAAGGATGAAAATACAAAACATCATCGTGATCGTAGATGTAGGCCAAGACATACAACATCTTCAGGTGTTTTCTCTGGACTCACTGTAACCATGAAGGAGGATATTATTAAAGATAGGATTGGTCCCAATGGAAGCAAATATGAAAGAGGCGTAGTGGCTCAAATCCATCATCCACATGAGTTGCCAGAGCCTGGAGTTGGCATTCTTCTTCCCGTTGATAATCTTAATAAAGTTGATATGGCAATTAAAATTACTATTACAAAAAGTTCAAATGATATTCGGGGTCTTAAACTTCAAGACAGAGGATGTATATTTAGGAATGAAAGGTATCTGAACATTACAAAAATCTATTCTCAAAGATCATGTATAATTGAATGCCGTCTGAGATACATTTTAAAAAGATGTCATTGCTTGCCTTACTATTTTAATAATTATAACGAAGGTAGAAAGTTGCCAATTTGCAATTCCTTACGATTAAAGTGCATTGTGAGCTTAAATGAGGACTTAAGATTTTATAAGCCACCAAATATCCATATGGAACTACACGATACAGAAACACATAGCGCTCTGGATTGCACGCATTGTCTACCCACTTGTAATGAAGTAAATTATGAATTTGTATCAGAGCGAACAAAAAATTTCTACGTTACTAGTAATAATTTCGATGGGTGCTTCGATGTGTATTTTAAATCAGCTGATGCTGTTACATACCAGCGAGAAGTCAGTTTTAATGCCATGCAACTCCTAGTTAATCTTGGTGGCATTGGAGGACTTTTTTTAGGAGGAAGTTTGCTCAGTATTTTGGAGATTTTATTTTGGTTTTGTAAAGCTATTATTTCCCAGACATTTTCCGCAAAGAATTTAATTAATAAAAAGAAGAAGAAAGCGAAAGTTTCT

>PPK-like1

ATGTTGCATTTTCAGTTTTATCACTTCAAGTTCACTCCTGAATATTCCAACAAAGTAGAAGACAAGAAAGATATTCCATGTTTTTGGCGCAAATGTTTAAATGGGTTTATGAATTTCTGCGATGCAACAACGCTACATGGCTTCAGATATGTTTCAGAAGACGGCAGACCGAACTATGAAAGGGTTTATTGGCTAATAGCAATTCTGGCAGCTTATTGTCTTGTGACGTTGGTGGTCTACGATCAAATAGAACGTTTTTTCAAAAATCCTGTATTAATCAGTATGGAACCAATTCTCACTTCAATTTCTAAGATACCATTTCCTGCAATCACTTTGTGCCCTGACAGTCAGATTTTGAGTACAACATTTAATCTGAGTTGGGCCATTCAAGAGCCATTTAATTCAACAGTAAAACGGTACAGTCTGTTAAAAAATGCTTGTCAATACTTTCAACCAACTGTAACTTATTTTGGGGCTTGTTTAACGTTTAATATGCTACCCCAGGATCAAATATTCAAACTTCCAAATGTCACTGTGAGTATCTTCTGTTGTTCGAATTACCATCACGTTGGCTTTGTGCATAATCCTGCGGAAATACCCGGTAGTTCTCATCCAATTGTGTACACAGAAGCTGACCACACTCTTCTTATCACTCTTACACCAAAAATCCACTTTATAGATAAAAGGCTTGATCGTTGGAATGCACATGAAAGAGGTTGTTACTTTCAGAATGAACGCTATTTACAATACTTCAAAATATATACTCAAAGAAACTGTGAAGCTGAATGTGAGGCCAATATCACAGAACAAATGTGCAATTGTCGTGCCTTCTACCATCCACGTACACAAGATATTCAAGTATGTGGTCCAGGAATGGTTGATTGTGTACAGTCCAAAGTTGGTAAGGAATGGGATAAATCATCTTGCAAATGCCTGCCCTCTTGTTCAGAACTTAATTATGATACTTCTTCGTTGAGATTTAAGAGAAACTGGAGAAAGCATATTTTATACTCAACTCTGACCGACAAGAATCATTATACAGGAATTTACATGAAATACAGAGCGAGATATTTTTCAGGAACCAGGAGAGATTTAATGATCAGTTTAACTGATTTTATTGCTAATGTTGGCGGCCTTTTAGGATTATTCCTTGGATTCAGCTGTCTCAGTGTTTTCGAAGTGATATATTTCTTGACTTTTAGACATTATAGCGATGGTTGCAGAAAA

>PPK-like2

ATGATCGGTTTCGAGGAGAACATGGAAGCCTGGCCAGTGCCACGCTCTCTAACACAACCCTCCATTTGGAGAAGAATCATGAATAGTAATTGGCTGAATAGAATAATGGAGTATACTAAGATATCTAGTATGCATGGACTGAGATATATAGGTGATAGGAATCTTCACATTTTAGAAAGATTTTTCTGGATTGCATCTTTCTTAACGGCTGTAGCCACATCTGCCTACTTTATAACTAATCTGTTTGAGAAATACAACGAGATGCCAGTCATTATGTCGTTGTCCCCCAGACCTGTCATGTTCAATGAAGTACCATTTCCCGCTATCACCGTCTGTAATATGAATAACATGCGCAAATCTGAAGCTGAGAAGATATTCTACGCCGCAGAGCATGGTTTATACAATGAGGCTTTCATGGATAACAAACTCGTTTATGATTTTTGTGATCGAGATCCGCCAAAAAATTTGAAAAACAAATCTTTGGGCAACTCAGCTACTTGGGATTATATACAAAAGTTTATGATTAACATTAGTCAACCTTGTCATGAAATGTTCATGCATTGTGAATGGCATTCCGAAAAACAAGAATGTATGGATTTTTTTAATCCATCACTTACTGATGAAGGAATATGCTGTGTATTCAACAGACTTAAAAGAGAGCATATATTTAACAATCCACGAGATTTGTCCGACTTAAATCTAACTTTTCCTGGAGAAACATACGACTGGTCCCCAGAAAAAGGGTTTCCCAAGAATTTAAGCCCTGACACATTACCCAGAAGAGCGAAAGGTCCCGGATCTCAGTTGGGATTAAGTATAGTAGTGAATGCCGAACTAGAGGAATATTATTGTTCCACAGATAACAGTCAAGGTTTCAAGGTGATGATAAACAATCCACTGGAAACGCCTAAGGTATCCTCATTTGCATTGTCCGTGGAGCCAGGCAAGGAGACTAAAATTGTCCTAACACCAAAAGTGATAACAGCCACAGAACAGTTGAGGCGAATTGATATTTCCAAACGAGAATGTTATTTCGAAAACGAGAAACAACTCTTCTTCTACAGAACATACAGCCAAAGAAGTTGCCACTTGGAATGCGAAGCGAACTTCACCATCGAATTGTGCGGCTGTGTTCTTTACTATATGCCAAAAAATGCAACAACCAGAATTTGTGGCAAAATGGATAAACGATGTTACAATTTTGCTCAGAAAATATTGGAACAAAGTGCGAAAGAATTAACTGCTAACGCCGCAACGAAAAGATTAAAAACGTGTAATTGTTTACCAGCCTGCCATGAAATTTCCTATAGCTTCATGTTGACAACAAGTCGAATAACAGATTCATTGAAAATAGATCCAGTTATGTTGGGGAGGTTCAATACAACATACTTCAAACAAAACATGGCAATTATTCACTTCTATTACATTGAGAGACAGTTTACCAGCACTATTAGAGGTGTTCTTTTTGGATTTACTGAATTTCTATCTAACACTGGTGGGCTCCTAGGTCTTTTCTTCGGATTCAGTTTCCTCAGTGCTGTAGAAATTGTGTACTTCTTGATGCTCAGAGTATTTTGTGAACAACATAAGCGAAAAAGACCAGAAAATGATGTAGAAAAAAATGTAACAACCCAACTCTCGATAGCAGATCCTAAATTTTTTATAAAACCGCCATTACCTTTCATTAAATAG

>PPK-like6

ATGCATAAAACAAAAATAATCAGTTCATTTTTGAAACCAGTTGGTTATTGTATACTGCTATGTATAATAAAAATTAAATTATTACCTAAAAATTCAATTTACATCGACATTGACACTAATTATCTGCATTGGAGTTCACAATTACCAGCGTTAAGTGTTTGCCCAATATTTCTACATTCCCACAAACATGAAAAAAATTATCATAATTTCATTCAAAATTTATTAAGACCAGCTGATCACTATTGTTTCAATTGTTCTAGAAACAATAATTTGCCTGAAAATGGATATTTAAGAATTGTTGCAAAGAAACTTCTACCATGTAATAAATTGTTTTCAAAATGTTGGTTGAATGCTAAATTGTTTGATTGTTGTACGAGATTCAAGTATCTGCAAACAAAGGATGGTGTTTGTTATTCAGCCAATTCAGAAATGACGGAATCAGCTAAAAAATTTGAAGTGAGTTTTTTTTCTAAAGAAGCTAATATCGAAGTGGAAACAACAAAACCAATTCAAATATACCTTCATGAAGTGAGGGATGTAGCTTTTATTAAATTACAAAAGGGGATTATAGCACGAAGAGGATTTCAATTACAAGTTTTATTCTCTGTTAGAGAAACTAAGAGCAGTTCATCTATACGATGGTTAAGTATAAAACAACGGAAATGTTTATTACAAGGTGAAAAAGTTCTAAAAGGCTATAGAAGTTACAGTAGTTCAGCTTGTTTGTCGCAAATAGCGGCTGAGAAACAGAACTCCGTTTGTGGATGCACTCACCATCTATTAGCATCAAGAGGGACATTCAGAGATTGTGATATTGGTGGATTAAAGTGTCTAGATCAAAATAAAGAAAGATTACATGCTACTGACAAGAACATTTGTCTTCCCTCTTGCGAAGTGATAGAACCTTTTATTATATGGCATCGAATAATCAAAACTAAAGGTAATACTACAAAAGTAAGTTTTACTTTATCATCACCTCCAACAGAAAGATACATAATGAAATGTAAAACAGATCTTATTAGAAATTTAGCTGCTATTGCCTGCTTATCTCGACTATTTACTAAGCAAAGTATAGTCTCAGTAATCAAAAGCTTAATGCCAAGAAGACCTTCAAAGATTACAGTAATTCGATCGCTCAGGGAAACAAATCATAGCGCAATTTTATACTAG

>CSP1

ATGAATTGTTTGCTACCTATCTGTTTTCTAAGCATGCTCGCCGTAGCATGCAGTGCCACCTCCACCTACAGTACCTCCTACGACAACCTGGATCTGGACGAGATACTCAACAACAGCAGACTCTACACGAGGTACTTTCAGTGTCTCACCAACAAAGCCAGGTGCACTCCAGATGGCAAGGAACTGAAAGCCGTACTTCCAGACGCTTTGGCCACAGGCTGTGCCAAGTGTAATGAGAAACAGAAGCTGGGCTCCGAGAAGGTAATCAGATTTCTGTTGAAGAACAAACCCAGGGACTTTGAGGAGCTAGAGAAGATGTACGACCCGAACGGCACCTACCGTCACAAGTACGAACAAGAAGCCAAGAAGTTAGGAATTAAAGTGTAA

>CSP2

ATGTTCAGAACAACAATCTTCCTCACTCTATTGGTGGTAGGATGCGCCTGGGCGGCCAGCACCTACACCACCAAGTACGACAACATCGATCTGGACGAGATCTTGAAAAATCCAAGGATCTACAAGAAGTACTTTGACTGTCTGGCCCACAACATCCAATGTACTCCGGATGGCAAGGAGCTCAGGGACATCCTGCCAGACGCTCTGAAAACTGCCTGTTCCAAGTGCACTGAAAAGCAGAAGGTCGGTTCAGAGAAAGTTTTCAAATATTTGTTGGATAACAAACGCGACGACTACGAAGTTCTGGAGAGGCAGTTTGACCCAACCGGCATCTACCGCAAAAAGTACGCCCAAGAAGCTAAGAAACACGGAGTCCATGTTTAA

>CSP3-FX

ATGACCTCATCGGGCTTCTGGCGGTTGAGTGGGGGCAGTGCAGTGCAATTCGCTTTTCTGTATATATACCGAGACAATCACCGACCATCGCAAGTTCAATCAGTGGTCACAATGTTCAGGACAGTAGCTTTCCTCACTCTATTGGTGGTAGGATGCGCCTGGGCGGCCAGCACCTACACCACCAAGTATGACAACATCGACCTGGACGAGATCTTAAATAATCCGAGAGTCTACAAGAAGTATTTTGACTGTCTTGCCTACAACACCAAGTGTACTCCGGACGGCAAAGAACTCAGAGAAGTGCTGCCTGATGCGCTGAAGACCGGGTGTTCCAAGTGCAACGAGAAACAGAAGGCTGGTGCCGAGAAGGTCATCAAGTTCTTGCTGGAGAAGAGGCGCGCCGACTTCAACATTCTGGAGGAGAAGTACGACCCCAGCGGCACCTACCGCAAGAAGTACTCAGCCGAGGCCAAGAAACATGGAATCAATGTTTAA

>CSP4

ATGTTCAGAACAACAATCTTCCTTACTCTATTGGTGGTAGGATGCGCCTGGGCGGCCAGCACCTACACCACCAAGTACGACAACATCGACCTGGACGAGATCTTGAACAATCCGAGAGTCTACAAGAAATACTTCGACTGCCTTGCTCACGACGCCAAGTGTACTCCAGATGGCAAGGAGCTCAGAGACATCCTGCCTGACGCGCTGAAGACCGGATGTTCCAAGTGCAACGAGAAGCAGAAGATGGGCGCTGAAAAGGTGTTCAAGTTCTTGCTGGAGAACAAACGGGCTGATTTCGACATTCTGGAAGGGAAGTTTGATCCCAGTGGTATATACCGCAAGAAGTATGCCGCCGAGGCTAAGAAGCACGGAATCAATGTCTAA

>CSP5-FX

ATGCAAGGACTGGCACTAGTGCTGTTGTGCACTGTAGCCGCGACTCTGGCGGCCAACACCTACACCACCAAGTTCGATAACATCGACGTCGATGCGGTGCTGAGGAACGAGCGCGTCTACAAGACTTATATCAACTGTTTAACCAACAAGGGCAAATGTTCGCCGGAAGGCCGCGAACTTAAAGACAAACTGCCGGACGCCCTGAAGACAGCCTGTGCCCGATGTTCTCCTAAACAGAAACAAGGACTTGAAAAAGTTCTCAAGTTCCTGATGAACTCCAAGCCGAATGACTTTAAGGAACTGGAAAGGATCTACGACCCGCAAGGAGAGTACAGGAAGAAGTACGCCAACTTGGCAAAGAAGAAGGGCATCCGCCTTTAA

>CSP6

ATGACCTGTAAAGTAGTAATGCTGGCAGCGCTGGTGGCCTGCAGTCTGGCCGCGGACAGATACAACACCAAATATGACCATTTGGACCTAGACCAGATCCTCAACAACGAGCGCATTTACAAGAAGTACCTGGAGTGTTTGATTCGGCGTGGCAAGTGTACGCCGGACGCACGCGACCTTAGAGATGCTTTGCCTGATGCCCTGCAGAACGAGTGCACCAAATGTACACTGAAACAGAAACTTGGCTCAGAGAAGGTAATCAAATTCCTGCTGAAGAAGAAACCGAATGACTTCCACTTGCTGGAGAAGACTTACGATCCGAACGGAGAATACAGGAAGAAATATGGTGAACTAGCCAAGGCGCGGGGGATTGTCCTGTAG

>CSP7

ATGAGGACTTCTATGTTACTGCAGGGGTTGATTGTGCTAATGGCGATGTTTTGCTTGAGTTTGGCCGGAGAAGGAAGATCTGCTGTGTCGGACGAGGCATTAGAAGCGGCTCTCAAAGATAAACGATACCTGGCCAGACAGTTGAAGTGTGCACTAGGAGAAGGAGCTTGCGATCCGGTTGGAAGAAGACTTAAAACTTACGCCCCATTGGTGCTAAGAGGAGCATGTCCAAAGTGTACCCCAAGTGAGGTGAAACAGATTCAACAAGTTTTAGCTCACATACAAAGGCATTATCCAAAGGAATGGAGTAAAATACTCAAACAATACGCAGGCCAATGA

>CSP8

ATGGTCCGTTTCTGCTATCTGACCGCAGTCGTCCTCTTTTGCTCGCTGAACCTCGCGTTCACGCTCACACTTCAGCAGATACATAAGTCTTGGTTCCAGAGGCTATCTGTCATACAGGTGGAAGACGTACTGAATAACAAGAGAATACTAAACAAATATCTCGGCTGTTTGCTGAAAAGAACGGTCTGTGCACCAGAAGCAAGAGATTTTAGAATATTGCTGCCATCAATACTGAGGGCGCCATGCACCAACTGTACAGAACGCCAGAGAACATCATTAAAGAAAGTATTTGGGCATGTACATGAACTGCATCCGAATGAATGGCAACAAATAATGTCTATGTATGATCCAAAATCAGAACATCAAGAAAAAATCATTAACTTCATCACGAATCGATAA

>CSP9

ATGAGGGTGCCAATAATACTTACATTAGGGGTTGTTAGTGTACTGATAGGACAGAGCTTGGCTCAACAGAGCATCCTGAATGGACAGAATGTCAACGCACTTCTGGCTGACAGTGGCTTCATTCGCAAACAGATAAATTGCGTGCTTAGTAAAGGACCATGTGATATAACTGGTAATCAGTTGAAATTGGCCATTCCAGAAGTGGTTGGAAGAAATTGCAGATCTTGTACTAAACAACAGGCCGCAAGTGCTAGAAAAGTAATCAACTTCATTCGGACTAGATATCCAACGGAATGGTCTCAGATTCAAGCAAGATATGGGAGGGGTTAA

>CSP10

ATGGTTTTCATAAAGTTGGCAACATTGCTAATAATGTCAACTACTATTTGTATAGCAGATGAGGAATATGATAGATTATTCGATCCAGACATAGATGTTGATGCTGTCCTGGACAATGATCGAGTATTGAACGCTTATATGGCCTGTTTCTTCGATGAAGGACCTTGCGCTGAACGACCTAAACTTGTAAAGAGCAAAATTCGAGAAGTATTGGAATCCACTTGTGGTAAATGTAACGAGCGTCACAGACAGCGTCTGAAATATGTTCTGAACAAGTTCATCGAACGACGACCAGAAGACTGGAAACGTATTTTGGAAATTTACGATCCGGAGAAAGCTTTCAGAGGAAACGTGGAGAAATTAAGGCAAGGCTTACCCCCTTAA

>CSP11

ATGATGATGTTAAGACTGTGCTTAGCGTCGTTATTAATGTTTGGTATGGTTCTATGTCGGCCAGACAGTGATGAAGAAGCTTTCTATTATAAAGTATTTGAAGAGATCGATGTGGATGTCATTTTGGATAATGAACGTTTGCTAAGATCGTATCTAAGTTGTTTCTTCGACGAAGCGCCTTGTAGTTCTCACGCAGCTGCTGTTAAAGAATCAATTCCAGAAGTAATGAGCTCGGTTTGTGGTAAATGCAATGACAAACAGAAAGCCATCTACAAACACGCTTTGAACAAATTCATACCTACACATAAAGAAGACTGGGATCATATGTTGAGAATTTACGATCCAAAAGGTGAATACTGGCCTAATATTAAAGCATTTATGGAATCATGA

>CSP12

ATGAAGTGCCCATCAGCATTATTGGTATTTGTCCTGGCCCTGTGCGCTGGATATTGTCTGACAGCTTCTACCTACACTACCAAATATGACAATATAGATCTAGATGAAATCTTAAACAACGAGCGAATTTATAAAAAATACTATAATTGCCTACTAAATAGGGGTAGATGTACACCTGATGGAAAGGAACTAAAAGAAAATCTACCGGATGCTTTGCAGACAGCTTGTGAAAAGTGTTCAGAGAAACAAAAACAAGGTAGTGAAAAGGTGTTAAAATTCATTTTGGACCACAAACCAGAGGATTATCTAGCATTGGAAGAGATGTACGACCCAGATAAAAAGTACAGGCATAAATACGAAAAGGATGCCAAAGAAAGAGGGCTTGAATTCCCTGAACCTAAAGAATAA

>CSP13

ATGAAGAAAGCAATGCTTCTAGTATTTTTGACTATTTTGGCTGTATGTTTTGGCGCATCCACTTACACCACACAGTACGATAATATCGATCTTGATGAGATTTTAAACAATGATCGTGTTTATACTAAATATTTTCAATGTCTTATTGGTAAAGATCAATGTACTCCCGATGGAAAAGAATTGAAAGAAACACTACCGGATGCTCTACAGACAGGTTGTGCTAAATGTACTGAAAAACAAAAAGCAGGAGCTGAGAAAGTATTGAGATTTGTTATAGAAAATAAACCAGACCACTACAAGCAAGTAGAGGCTATTTACGATCCCAAAGGAATTTATAGACAGAAATTTGGTAATCAAGTTAAAGAGAAAGGAATTGAACTACCCAAATAA

>CSP14

ATGGCTGGCGGGAAAAAGAATGTATCGCTTCCAGATGCTTTGCGAACTAGATGTGAGAAGTGTTCAGAAAAACAAAAGATTGGAAGTGAGAAAATTCTCAGATTCTTATTGGAGAAAAAGCCTGCTGATTATTTAGAATTAGAGAAAATCTATGATTCTGATCGCAAATACAGGAACTTGTACAAAGAAGAAGCTGATAAAAGAGGTCTTAAATTTCCAGATAAATAG

>CSP15-NTE

TTCTTCTTTAGGATTATTATATTTGTGCCCAAATCGTCTAAATTAATATCTTCGAAGCATTCAACTACCTCGCTTCCAGATGCTTTGCGAACTAGATGTGAGAAGTGTTCAGAAAAACAAAAGATTGGAAGTGAGAAAATTCTCAGATTCCTATTGGAGAAAAAGCCTGCTGATTATTTAGAATTAGAGAAAATCTATGATTCTGATCGCAAATACAGGAACTTGTACAAAGAAGAAGCTGATAAAAGAGGTCTTAAATTTCCAGATAAA

>CSP16

ATGAATCTTTATATACTATCGTTATTTGCAACAGCCATCTTATTTGTTGGATGCATTGAAGCTGGCGAGGTATATACCTCAAAGTATGATAATATTGATGTGGATAAAATTTTAACTAACGACCGGATACTTAGTCAATACATCAAGTGTTTGATGGACGAAGGCAACTGTACCAATGATGGGAAAGAACTTAAAAAAACCCTGCCAGACGCACTAGCAACAGGTTGTAATAAATGTAGTGAAAAACAGAAATCACAGACTGAAAAAGTTTTAAGACATTTGATCAAAAATCGATCAAGAGATTGGGCCAGACTTAAAGGAAAATATGATCCAACTGGTGAATATTCTAAAAAATATGAAACTAAAGTATCATCTACACAAAAACAG

>CSP17-FX

ATGCTTACAGACATGTATACCAAAGTGTTGGTAATTCTAGCATTGACTGTTTATTTTTGTTATGCGGCGAAGAAAGTAACAACTTATACAGATAAATACGATAAAATTGATGTCGATGCCATATTAAATAACGAAAGAGTTCTAAAACGTTACATAGATTGTCTTATGGATAGAGCAAGATGTACCCCAGATGGAACTGAATTAAAAAAATACATACCAGAAGCTTTGGAAACAGAATGTGCGAAATGTACAGATGCTCAAAAAAGATTTGCTGGGAAAGTAATGTCTTTTCTACTGCTAAATAAACGTAATTATTGGAATCAGCTTTTAGGGAAGTATGATCCAAATGGAAAATTTAGAAAGAAGTACGAAGAGGCAACCCTTGAAGATGAAGATTATTCCTCTTTCTACAATGAATAA

>CSP18

ATGCTAAGATTATTTATATTAGTGCTATTGTCGTTGACAATACTGTTGCATTATTCAACAACAACACATATTGATGGATTATTTGAGGATAATATTGACTATGATTCAATATTGAAACAAGACTACTTACTGAAACCCTACATGGATTGTGTAATGGAACAGAGTAGATGTAACAAAGATGGGAAATATTTGAAAACGCTACCTTACATTATTAAAACTGAATGCCAATACTGCAATGTTGACCAAAAAAAGAAATTGACTAAGCTAATTGAAAAATTCAGAAAATATCAACCAACAGAATTTCAGAAATTATTAACAAAATATGATCCAAATAATTCTCATTGGAATGCATTTGAAAAACTGGTTGAAGAATACAAA

>CSP19

ATGTGTAAAAAAATGAAATTAATAATAGGTGTTTTTCTCACCTCCTTTTTAATCACAATTTATGCACAATATGATATTAATGTTGAAGAAATATTGAATAATAAAAGATTATTGGATGCTTATAGTAGATGTTACCTAGATAAAGGACCTTGCCCAGGACCGGCAAGAGAGAGCAAAAAAAAACTGGGAGAGGTATTTGCAACGAACTGTGCTAAGTGCAACAAGAAACAAAAACAAGATACCAGAGGTGCGATCAGGAAATTAAGAGAAAGAAAGCCACAACTTTTCTTGGAGATATTTGAAAAATACGATCCTGGCAGTAAACATTTGGATGGATTTCTTATTTGGTTGAAAAAAAATGATTGA

>OBP1

ATGAAGACCACACTTTCAGTTCTTGCCGTATTCCTAGTCATCGCTTATGTAAATGGAGCCGCCACACAAAGCCCTGTGAAAGAAAAAGTAGCTGCATTGGCCAAAAAATGTTCAGCTGATCACAAAGCAACCCCAGAACAAACCAAGTTGGTATTCTCACAGAAAGTTCCTTCTGATGAAGTAGAAAGGTGTTTGCTGGAATGTGTCTACACAGGAGTTGGAGTTATCCAAGGAGGTGAATTTAGTGCGGAAGGTAGCAAGAAATTGGCCACCTTGAGGTTCTCCGACCCAAAAGAACGTGAAACTGTCAACAAGTTGGTCGATACCTGTACTAAAGAAGTAACCAAAAATAAGGACGAGAAATGCTCACTTGGTAAATCTGTCAGGGAATGTTTTGTCAAACATGGTGAAAAGGTACATTTCTTCCCTTCAGCCAACTAG

>OBP2

ACAGAACAAGCCAAGGTGATATTGTCAAAGAAAGTTCCTTCAGATGAAGTAGAAAAGTGTTTGTTGGAATGTGTCTACCAAGAAGTTGGAGTTGTAATCGAAGGAGATGAATTCAGTGTGGAAGGTAGCAAGAAATTGGCCACCTCAAGGTACTCCGACCAAAAAGAACTTGAAACTGTCCACAAGATGATCGATGCCTGTACTAACGAAGTAACCAAAAATAAGGACGAGAAATGCTCACTTGGTAAATCTGTCAGGGAATGTTTTGCCAAACATGGTGAAAAGGTACATTTCTTCCCTTCAGCCAAC

>OBP3

ATGGCTAGTAAAACATCATTTCTAATTTTCACTTTTACTCTGGCAGTTGTGTTCAGTGTTTGCTTGGCACAGGACGAGTCATGTAGAAGTCCTCCTGCAGGATGGCCAAGGAGGCCTCCACAATGTTGTGATATTCCATTTCCATTAGAGAGTATGAAAAGACAATTTGGATCCTGCGTGAGACAGATTGGCAGACCCTCCTCTGCCGTTCCTACAGCTAAAGCAGTAATGGAGGCAAGATATTGCATTGAAGAATGTGTGTACAAAGGAATGGGATTGTTGGATGAAAGTGGTACAGCTTTAAATCATCAACGACTAACCCAGGAACTTAATCGAGCTGTAACCGGTGCTGGACTCTGGGAGTCAGCAATGCAACAGGCCATCACGTCCTGTTCTGGAGCAACTGGTCAAGGGAGCGATGATTCATCATGCAGCGAAATACCTCATGCGTTCACACACTGCCTAATGAGGCAATTATTTTTGAATTGTCCAGCCGACAAGTGGAACAACAGCGCTGAATGCAACCTGGTAAAAGACAGGATGCAAGTGTGTCCAAACATCCCGCCGCCGCCTCCAATACCGCCCCAGCAGAGATCCCAACAACAGAATTTTCAACCATAA

>OBP4-NTE

AATGCCCCTCTATGTCAACCTCCGACAGCTGCCCCTCACAAGCTTGAAAAAGTTATAGGACAGTGCCAGGAAGAAATCAAGTATGCGTTAATTCAAGAAGCGCTGAATGTATTAAGAGAATCAGTTGGACTGAGTCAAGCTCGAAATAGTGCAGCTCATTTGAGAAGTAAAAGACAAGCTTTCACTGGCGAAGAAAGGAGAATAGCTGGGTGTCTTCTCCAGTGCGTTTACCGCAAAATGAAAGCTGTTGATGAAAGCGGTTTTCCGGTGGCTACTGGACTTGTGAAAATCTATTCAGAAGGGGTGAAAGATAGAAACTATTACCTAGCTACAATACAAGCAGTACAACAATGTCTCTCCCGTGAAATACAGAATAAAAATAATGATCCAAAAATTGTAGAAGAAGGGTACACCTGTGATGTAGCTTACAATATGTTCAACTGCATCAGTGATCAAATAGAACTACTGTGCGGCAGTACACCG

>OBP5

Coding error

>OBP6

ATGGGTCCATCTAGGACAATTGTGCTAGTGACTACATTTACACTTACTACTCTGCTACTAATCACCAATACAAATGGGGCCATGTCTGAAGCACAAATGAAACAGGCGATGAAAACATTAAGAGGCATGTGCCTCGGGAAATCCGGTGTCACTACAGAAACTTTAGACAAAATGCAAGAAGGAGTCTTCAATGATGATGACAGAAATTTAAAGTGCTACTTAGGCTGTATTATGGGCATGTTGCAGGTGGTAAAAAATAATAAAATCAGCTTGAAGATGGTAAAATCTCAGGTAGCCAAAATGTTGGAGCCAGATATGGGTAAAAGATTAATAGCTGCTTTTGAAGGTTGCAAAGATACTGTTGGTGAAGATAACTGTGATTTGGCCTATAAATTTGCAAAATGCATATATGTATCCGATTCGAGTGCTTTTATTGTACCATGA

>OBP7

ATGTGTAAATTAGTGATTTTACTCACTGTTTTTGTTCTGCAGATGTATTTAATTTCTGCCCAAGAAGTGAATGAAGAATTATGGGGAAAAGCAGAAAAGAAATGCCAACAACTTTCCAAATTGAGTTCAATTGAAAGAGCATCAATTCTTACAAGTTTAGGGAACATATCTCGTACAGCTAAGTGTTATGTAAATTGTTTTTTTGAAGAAGTTGGATTGATGATTGGAGCAGCTGTAAATAGTACATTGTTGCATTCATGGTTGAAGGAAGAAGCTGAAATTTCAGAAAACAAAGAAGAATTATATAACAGAATTAAAACCTGTGTTGATGCAATTACAGTTGAAGATAAATGTGATAAAGCTTATGAACTGTATATTTGCTTCGAAAATAGATAG

>OBP8

ATGAAATCAATTTTGTTAACTGTTTTCTGCATATTCACAACTCATCTGGTTAGAGCCGAGATAGATCCAGCAGCAATAATGCAAGATATGGAAGTTTGCAAAAATGATACCAAAATGAGTGATGAAGAATTTTCTGAACTGATGGCACAACAAATGCCAAAATCACCTGAAGGAAAATGTTTCATATTTTGTATGATGCGATCAATGACCTTGATGTCAGAGGAAGGTGAATTGAATTCAGAAGGGTGCTCCGCGATGATTGCTGAAGCGCCAATTGATGATGACCAAAAAGAAAAGATGAAGGGCATGCCTGAGGAATGTGCTAAAGAAGTTAAAATAGTGAAGGAAGATCCATGTGAAACGGCTTACAGAATATCGCTGTGCTTTCAGAAGAAGGCGGAGGAGAGAGGAATCGAAGGGCCCATGTGA

>OBP9

ATGAATATGTTAACAATCCTAGCCACCTTTTGCTTTTTAATTGCTGCAGTAAATTGCAGGGTATTGAAACAGGAAGATGCTCTAGAAAAATGCAGCAAAGAAAATAGTATTGATAGAGAACTTGCACAAAAGATAATTTCACATCAAGCCCACGCCACAACTCCAAACGAAAAATGTTTGATTGCCTGCTACCTGAAAGTGAAAAGATATCTGCTAAATCACCAGATCGATTGGGTAGCGATTTATAAATCACATGCAGCTAGAATAACGGATGAAACGGAAAAAGAAAAAGTGAAGGCCATTTTCGAAGAGTGTGAGGCAAACTCCTATGAAAATAAAGATGACTGTCAAATAGCGTATGATGCTTACGAATGTAAGAAATTA

>OBP10

ATGAAGAACTTTTCTCTGATTATTTTTAATTGTTTTGTCATTTGTGGTGTGATTTGTGTGACCATAACGGATACACCAGCATTCTTTGAAAGCGTTGTGGAAAAATGCAGTGCTGAACACAGAATTGATAAGAATGAAGCCCGCAGAATGATCGGAAATGTAAGAAGATATGCACTAACTGAAGATGACAAATGCATGTTGAGCTGTTATATGAAAGAGAGGCAATATTTCGTTAATGGTAAAATAAATTGGCAAAGGATCTTTATTCCAGTTTCCATTATGCTTTCTACTGAAAGTGAAAGGAGTACAATGTGGGCCAATTTCCTGAATTGCGATAAATATCTCTCTTATGGAGTTCGAAATGATTGCCAGATAGCCTATGAAAGTCTCAGATGTTTTGAGGATGTAATGAATGCGTAA

>OBP11

ATGAAAACCATAGCTGTGATTGCTATCGTGTGCTACCTATTTGTAGCTACCGAATCTTTTCCTTCACACTCTCCGGAACTTGACACATGCAGCACTAAGCACCATTTATCAAAAGATAGGGCACGCCAATTAGCTATCCACAGTGTACAGGCCACCGATGATAATGAGAAATGCTTCCTGAGCTGCTATTTGAAAGAAAGGGGATATCTCGTTGATGGGAGAGTAGATTTTGACAAACTCTTAGAAAATCATAAGAAATTCCAACCAACACTTTTTGAAGAATTGAAGAAGACATTTGACGAATGTAAAAAGAATTTGGATTATAATGGCAAGAACGAGTGCGAAGTGGCGTACCAAGCGTACAGTTGTGCTTTCACCAGACCCTAA

>OBP12

ATGATGGTGCATTTCTATCCTCTGCTTATTGGTATATTGATTGTGAACAGCGTTTATAAAGTTGATTCAACGGGAGGAGAGACTGTAAAAAAAGCTCTGGAAGTTTTAAGTAAATGTCAAAAAGAATTTTCGGTAGACGATGGTACTCTGTCTAAATTGATGGACTTTAGTCGTGTTCCCCAATCTGCAGAAGCGAAGCGCATGGTGACATGTTGGATGAATGGAGTTGGATTTTTAGAGGATGGCAAACCATGTGTAGAGGAAATAAAAAGATGGCATAGGGTATTGTACACGTCTGAAGAACATCAAAAACTGGCTGATGAAAACGTAGACATTTGTAGCGCTAGCTTGAAAGCTAAAGAAGAAGGAGAATTGGCTTATGAGTTAACTAATTGTTTTGTGTCTCTCGCAGAAAAGACCGGACTACCACCTCCGAAACTAAGAATAGAATAA

>OBP13

ATGTTATTCTACGCAGCTCTAATTGTGGCCCATGCTATTTACTTTACAGATGCAGTGGCGGAATTAAAAGGATTAACTAAAAGTGAATTGCTTGATCTCGAGAAGAAAACAATGGCCGAATGTTTATCTAAACATGGAATTGAGAAAAGTGTGCATACAGATTATTTAAAGAACCAACCTCTTGATGTATCAAAGGAATTCAAGTGCGCAATTGGCTGCTACGTCGGAGAAGTCGGTTATGTCATAGATCGAAGGACACGATGGAGTGCCATAGAGGAAGTGCATGATATTGAGTACAAAAGTGAAGAGAATAAAGAAAAAGCTGCCCAAGTACTGAGAATTTGTAAAACTATTGTACCTGAAGTTGGTGAAGACTCATGCGAAGCTGGGTTCGCTCTATATTCTTGTTACATGAAAGAAGCAATAAAGATTGATTTGCATCCTTGA

>OBP14

ATGTTTCTCTACGCTGTCTTGCTTGTCACCTGTACTGTTCTTTGTACCTATGCAGCTCTAGATTTGAAAGGAATGACTAAAACTAAACTAGCAGAGCTTGAGGCAAAAAAAATGTCAGAATGTTTAAAAAAGTTTGGTGTAAATATAACTACGCTTTCTGGATATTTAAACGAAGAAAGTAATAATACTACAAAAGAATTCAAGTGTGTACTCGGCTGCTTTACTGAAGAAATGGGTTATGGTAAAGATAACAAACCTCTGTGGGACATTATGGAAGAAGTACATAAAATTGAATATGAAACTAAAGAAGATAAAGAAAAAGCTTTGAAAATTGTGGAAACTTGTAAAACTATTGTTCCCGAAGAAGCTGAAGATTCTTGTGAACTTGGATTCGCGATGCATTCATGTTATACGGCACAAGCAAGAAAGTTAGGTCTGACATTGGAGTAG

>OBP15

ATGAAAAAGGGAAAGTTCAATTCTAAAAACACCATGAAAATCGCTCACAAAGCTTATCCCAATGATGCAGAGAAAATCAATAAAGTAATTGAAATCCTGGACTCCTGTGGCACTAAAGAGAGCAAAAATGGAGCAGAAACTGTTCGGAGAGTGCTAAGAAAATTCGACTATCATGGTAGAGTAGCCTGGAAGAATCCTTTTGTCATGGTGTTTACATTCAACACATTTCTGGTAGAACAG

>OBP16

ATGCTTAATGCATGCTACGGAATCACTGCTGCTAGAAGTACATGGATCTTTCCAGTTGTAGAAAAAATATGGTCTGAATTTGTAAAACAGCTTCAAAGACCAAAGACAGGTGGACACCCTCAAAATAAAACTGCTTTACTTAAAGCAAGTTTTATAGAATCATCTAAATATTGCTCTGAAAGGCATGATGTGAATATGGTTCAAATGTTGGTCTTGTTTTCCGAAAAAGAAGTTAATGACTATAAAGGAAAATGTTACTTGCATTGCATGTTACAGAGATATAGATTGATGGATAAGGATGGAAATTACTTAAAAGATAGGTTTCAACCATTTATGGAGTACATCCCCATTTCTAACTTCCTGAAAACACTAAAAAAGAATCTTAATTATTGCCTAAATCAAGATATATGTTAA

>OBP17

ATGCATCCATTAACTCTTGTCTTGGGAGCCGTATTTTATACACTGTTTGAAGGAGCATACGCTGCAGTGGAATTATCAGATGATATGAAAGAAATGGCAAAGATGCTGCATGATCAATGTATAGATGAATCTGGTGTAAATGGAGCTTTGATTGAACCTTGTGCTAAAGGAGACTTTTCCGAAGATGGAAATTTGAAATGTTATTTTAAATGTATCTTCGCAAATATGGGCGCGCTCTCCGATGATGGTGAATTAGATGCTGAAGCTTTTGAATCAATTTTGCCACCTGATTTACACGATCCACTATCAAAAATGATCAACAGCTGCAAAGATGCCAAGGGCGCTAATGCTTGTGAAGTGGCATTCAACTTCAATAAATGTCTTTATAACGCAGATCCTGAGCACTTTTTGGTGATCTAG

>OBP18

ATGTATGTATTTCAAATGATTGTAGGTTTACCGTATAAAAGCAGTGAAATTAATGGATTAGTTTTTCATATTGTGAGAATCAATAGGATTGTAAACATGAACAGTTTTGTTGCATTTACCTTGTTCGCGTGCATTTCTGCGGCTAGTGTCACGGCCGAAGATAATGAAAAAATTTTACAAATTTTTAATAAATGCAGTGAAATGCACAAAATTTCAGATGATGAATTAGCAATGATCCAAAGTAAACAATCTGTTCCATCATCGGCTGAAGCTAAGTGTATGGCAGCGTGCATGCTTAAAGAAGGCAAACTTATAATTGGTACAACTTACATGAAGGATAACGCCTTAATGATCGCCGATGCACTTTATAAAGACAGTGCTGACATGGCTTCTAAAGCAAGAGAAGTTGTTGAACATTGTGCAACAGAAGTTGGCGTGGATACAGCAGGCGATGACTGTGAATTCGCTTACAAACTTGCTGTCTGCAGCGATAACCATGCTAAAAAGATCGGAGTTACCAGACCATTTTAA

>OBP19

ATGAGGTACTATTTGCCTGTTTTGTTAATTGCATTTTGCATTGCAGCAACCAAAGCGCTAGACGAAAACGTCAAGGACAAGATATTGGGGACATTCAGAAAATGCAGAAATCAACATCCAATCAGTGACAAGGAAGTCCAACAGATAAAGAATTTCGAATCAGTGCCATCATCCAGGGAAGCCAAATGTTTGGCAGCTTGTATGCTGAAGGAAGCAGGAATGTTAAGAGATGGTAAATATAATAAAGAAGTGGCAAAATTTGTGGCGGAAGGTGTTCATGAAGACCAGCAGGAAGTCGTCAAATCTAAAGAAATGATTGACCATTGCACAGCTAAAGTTGGAGAATTCGCTGGAGACGATGAATGCGAATTCGCGTACAGAATGGCGATCTGCTCTCATGACTATGCGAAAAAGGTTGGAATCAAGAAACTCTATTTTTGA

>OBP20

ATGAAAGGATTTGGAGCTATCTGTGTATTTGCCATCGCTATCACTTTATCGCTAGCGATCGATGAAGCTGCAAAAAAGAAGGCCATCAATACCTTCAATAAATGTAGAGAACAATACCCAATCACTGATGCTGAACTGGAAAAGCTCAAGAAACATGAGAGTTTACCAACCTCAGAAAATGCAAAATGTCTGGCTAAATGCATGCTTATGGAAGGAAATATGTTAAAAGATGGAAAATACAGGAAAGACATTGCAATCACCTTTGCTGAAACTTTGCATAGCGACAACGCCGAAGAAGCTGAAAAGGCTAGACAAGTGGTTGAATTCTGTGCCAATGAAGTGGGAACCGAAGTTAAAGGTGATGCTTGTGAACATGCGTACAAAATGGCTGAATGTAGCTTCAACAAGGCTAAAGAGATTGGACTGGAGAAACCAGAATGGGAGAATTGA

>OBP21

ATGAAAGCTTTACTTCTGATTGTATTTATGTCAGTCTCCATCAATAAGATTATCTGTGAAAATGCTACTGAATCTGATGAAGCAGATCCAGAAACTGTGCAATACCTTACGTGTATTCAAAAATACAATCTAACTGGCACAGAGGTTGCAACAAAAGGAAGTTATGCAGAAAAATGTGGTCACGCATGCGCAATGAAAGCTAAAGGTATGTTGTCAGATGATGGTGAATTCATTAAAGATCGGATGTTATTGCAAGGTTTTCCGCCGAACATGACTTACGTAGAAATGACTAGAATGCAAATGGCCATAGAAATGTGTGCAAGGCAAGTGACACAAGAGGAGGATGAATGTGAAAAAGTTTCCGCACTGATGGATTGCTTGCATACTAAGATGAAGGACCCCAGAAATCTCCGAAAGGTGAAGTAG

>OBP22

ATGTATTTGTTTTCTTTCATTTTAACGTTGTTCTGTATCTTTGTCACTATTTATTCTGGATTAGTGAACAAAAATATAACAAAAGAAGTACTGGAAGAGATAGAGATGATGGTGTTGGAAGAATGCAGAATTAAGTTTAACATTGCTGAAAAGGTAATTGATGACTACTTCAATGAAAGTGTACCGTTGCCTGTTAATGAAGATTTTAAATGTATGTTACAATGCCAGGATGAACGTTTGGGTTACATAAATAATCAAATTATTAACTGGGATCTCATAAGAGTGGTTCAAAATATATATTATAAAGATTATGATAATTCCAAAAAATATCTGGAAGTTGTAGACAACTGTGAGCAAAAAGGAGTTGTTGAAACCGAAAATAGCTGTGACATAAATTTTCAGATTACAGAATGTTTTAGAAAGCAGTACATTGAGCAAGGATTGCAGTGGTAA

>OBP23

ATGAAATACTTGTGTATAATTGTTAGTTGCTTACTACACTATGTGTATACTGCTCAACAAAATGTCACAGAACTATCTAAAAAGGAACTCTCTGATTTAGAGGATGAAGTTATGGAGAAATGTAGAGAGAAACACAATGTTGGTGAAGAGATTTTTTCCAAATTGGAAGCAATTTCTCAACATAAGGAGGAATTACCATCTTCCAAAGATTTCAAATGCATGTTGGGCTGTTTTGCAGAAGAAATGGGATATGCCAAAGACGCCCAGCCACAATGGGAAACAATGGTTGCAGTTCACAAAATAGAATATGAAAAAGAAGAGGATGCAAATAAAGCAATTCAAGTTGTAGAAACATGCAAAAAGTCTGTTCCTGAAAAAGAGGAAGATATCTGTGAACTTGGTTATTCTATGGCAACTTGTTATCTACTTGAATCTGAAAAGCTCGGACTGGACCTGGTTTAA

>OBP24

ATGGCACATTTTAAAAAAGTAATCTGCACTTTGACAGTTCTGTTCCTTGCATTGGTGTCAGGCTACAAGGAAGAATTAAGAGCAACTATGAAAAGCTGCAAAAATGGCCAAGACGTTACTGATGCTGAAATTGAGGAATTCATGAAACCATTGATCCCCAAAACGGATGAAGAAAAGTGTCTAATGGCATGTGTATTCAAAGCCTTCAACGTGATTGATAATGGCCATTACGATCCAAAAATTGCTCTAGCTGTTGCTTCAGACCTGATGAAAAACGAGCCTGAAAAACTTCAAAAAATTAAAAACATCATAGACCATTGTGGTGATGATGTACCTAGGAAGATGGATAATGAATGCGAACTTGCTTCTGAAATAATGCAATGTGTTGTCAAATATGAACGTGAGGTCGGAATTGCTTGA

>OBP25

ATGTATTTCAAATGTTCAGTATTTACAACAGTGTTTACTTTGCACATAATGTTGGCCACCTGTGGGCCATTGCAAACTTATGTAAGGTGTCGCTTGGAAAGTAACATCAGTATGAGTGAAATTTCACGATTAGAATTATTGCAGTTACCTGTTAGCCAAGAAGGAAAGTGTTACATTGCCTGTTTATTGAGGAGCTCAGGATTAGTAAACACAGAAGGCGATATTGATCCTTACAATGTTATTAGTCTGGGTAGAAAATATGCTGTTGATCGAAGTAAACTATTGCGTATGGAAACAGTTGTGTTAGAATGTGGTAACCAAGGTAATTTTCATTTCATTTCTTATTTTATTCAATAT

>OBP26

ATGTCAAAATTGTATTTTTGTTTAGTTTTACTGACAGCATTTTACTTTGCACAATCACACGTGCAAAGAAGAGAAGTCCAAAATGAAAAGGTTGTGTTAAGCAACTTGACACCAAAGCAATTGGATGATCTTGAGAGGAAAGTCTATGAAGAATGTGCTGTTAAATTAAATGTTGACAAAGGTTATGTTGAAAAGTATTTCAGAAACGATACCGGAGTACCAGATGATGTTGGATTCAAAAAAATTACTGGCTGTTATGGAGAAAAAATGGGATATGTCTCGGGAACAAAACCAAATTGGGAGAGGTTAAGAGAAAGCTACGAAGTATATTACAAAGATAACAAGGAAGATCTTGAAACAGCTTTAAAAATTGTGGATGAATGCCAGAAAATTCCTCTTGATAACTTGGATGGAAACGAAGTCAATTATACTCTAGCGAAGTGTACTAAAGAAGGATACCTAAAGTCTGGCCTAGAATGG

>OBP27

ATGCATTTATTCGGCTATATTTTGGTCGGTCTGGCTGTAGTAGATCTAGTTCTATCAATTCAAGAAATTAAAGGGCTTAACAAAAAGGAAATACAAGAGCTTGAACTAAGATCCCTTGAAGAGTGTCGAGTTGAATATAATGTGGACAAAAGTGAAGTACAGAACTACTTAGATATGGATAAGGTTATACCCGATAACGACGGCTTCAAGAAAGTATTGGGATGTTATGCTGGAAAAATGACGTTCGTTGATGACACTCGAATCAATTGGGATAAAGTTAGATTAGCGGCCGAAATATATCACGAGAATGAAAAGGAATATCGAGATAAATCTTTGAAATTGATCGATGAATGTGAAAAAAAATGTAAGTTATCATACGAGGGACTTAGTCATAACGAAGTAAGCTATGTAGTTGCGAAGTGTATTAAAGATGGCTACGTACAGAGAGGCATAAAATGGTAA

>CHEB1-FX

ATGAAAACGCCAGGGAAACCTACGCAGAATATAGTTTTATTTTTATTGCTTTCATTAATTTGGAATTTGGAATGCAGAGAAATGGCAGGCCCTTCTATGAATATATTAAAGAAGATTGAGCAATGTGACTCGACAGAAGGTCATGCTATAAAGCTAGACAAGTATTGCATAAACAGATACAATCAAACCGTTTATACCTACGACGGCATCATGGAACTCCAGACAGAATTGACGAACGAGGACTCAGAAGTAAATATCATGGTGGAAACGTGGGTTAGTGGAGGCTGGAAACGTACTTTGCAGAAGAGAATGGAGCATATATGTTCTACATTTGAAAAATATACACCAAATTATATCCAATCTATTATGAAACAAGCAAACGTAACTAGTTGTCCAATACCTGCGGGCATTTACGAATTTAAAAATTTAGTGACCAACGTGGACATTACAATTCCAATTCCTTACGGACTGTACAGAAGTCAAATGGATTTATTTAAAAACGGAACACTTATTTTCTGTATTAGAAGTATTCTCGACGTTGTACCGTTGGATTAA

>CHEB2

ATGAAAACGCCAGGGAAACCTACGCAGAATATAGTTTTATTTTTATTGCTTTCATTAATTTGGAATTTGGAATGCAGAGAAATGGCAGGCCCTTCTATGAATATATTCAAGAAGTTTGAGAAATGTGATTCAACAGAAGGCCATGCTATAAAGATTGACAATTATCGTTTTAACAGATACAATAGAACAGTTTATACCTACAACGGCAACATTGAACTCCAGACAGAATTGAGGGACGAGGACTTAGAAATGAATATCATGGCGGAAAAGTGGGGTAATGGAGGCTGGAAACGTACTTTGCAGAAAAGATTTCCAAACGCATGTTCTTCAGCTGAAAAATTTACACCAAATTATTTCCAGTCTATTATGAAACAAGGAAACTTAACTGGTTGTCCAATACCAGCGGGCCATTACGTATTTAAAGATTTAGTGATTAACGCGGACTTTACAATTGCAATTCCTTACGGACTGTACAGAAGCCAAGTGGAATTGATTAAGAACGGTACAATAATTTTCTGTATTACATGTATTATCGACGTTGTACCGTTGGATTAA

>CHEB3

ATGAAAACGCCAGGGAAAAATACGGAGATTATAATTTCATTTTTATTGCTTTCATTAATTTGGAATTTGGAATGCAGAGAAATGTCAGGCCCTTCTATGAATTTAGTAAAGAAGATTGAGAAATGTGACTCGACCGAAGGCCATACTATAAAGGTTGAGAAGTATCGCATAAACAGATACAATCAAACCGTTTATACCTACGACGGCAACATTGAACTCCAGACAGAATTGAGGGACGAGGACACAGAAATAAATATCATGGTGGAAAAGTGGGGTAATGGAGGCTGGAAGCGAAATTTCCTTAGAAGAATTCCAAACGCTTGTTCTTCAGCTGAAAAATTTGCACCAAATTATGTCCAGTCTATTATGAAACAAGCAAACATTACTGGTTGTCCAATACCAGCGGGCCATTATGAAGCAAAAGATTTAGTGGTCAACGTGGACGTTGCATACCCTATTCCTTACGGACTGTACAGAAGCCAAGTGGATATGTTTAAGAACGGTACAATTATTTTCTGTATTAGATTTATTCTTGATATTGTACCGTTAGATTAA

>CHEB4

ATGAAAACGCTAGGAAAAATTACGCAGAATATAGTTTCATTTTTATTGCTGGCATTAATTTGGAATTTGGAATGCAGAGAAATGTCAGGCCCTTCTATGAATTTAGTAAAGAAGATTGAGAAATGTGATTCGACCGAAGGCCATACTATAAAGTTTGAGAATTATCGTTTTAACAGATACAATAGAACCGTTTATACCTACAACGGCAACATGGAAATCCAGACAGAATTGAGGGACGATGACACAGAAATAAATCTCATCGTGGAAAGGTGGGGTAATGGAGGCTGGAAGCGAACTTTGCAGAAAAGAATTGGACATATGTGTTCTTCACTTGAAAAATTTGCTCCAAATTATTTCAAGACTGTTCTGAAACAAGAAAACGTAACTGGTTGTCCAATACCAGCGGGCCATTATGAATTTAAAGATTTAGTGATGAACGTGGACGTTGCAATTCCAATTCCTTACGGACTGTACAGAAGCCAAGTGGATTTGATTAAGAACGGTACAATTATTTTCTGTATTATATTTACTCTCGACGTTGTACCGTTAGATTAA

>CHEB5

ATGAGGCCACCCTCCGATAGTTTCGTTTTAAAACTCTGTTGGATATTCTTTATTATAGTTGGGATTGTAGAGAATAAAACGATGTCAGGCCCATCCCAGTATGTAATAAAATCATTAGAGAAATGTGAATCTACAGAAGGCCACAAGTTAATGCTGGACAAATACAGAATTACCAAGTTCAATAGGACTGTTTATACCTACAATGGCAATATGACCCTCATTGAAGAATTAACAAATGAAGGCACAGAGATAAACTTCTCTTTCGAGGTTTGGGGTAATGGAGGTTGGAAAAAAAATTTCTTTAACAAAAGATTTAAAAATACTTGCAGTGTTCTAGAACAATATTCGCCAAAATATGTTGAAGCTATTAAAAAACAAACGAACATAACTGGTTGTCCATTACCTGTGGGTACTTATGAATTTAAAGATTTAGTGATTAATTTGGATATGGCAGTTTCAATGCCTTACGGACTGTACAGGACTACTGTGGAGTTCTTTAAAGATAGTGAAATATTTTTCTGTATGAGACTTATTCTTGATGTGTTACCAGTTAAATAA

>NMDAR1-FX

ATGAAAGTAATATTCATCCACAGTTCTGATACAGATGGTAGAGCGATTCTTGGAAGATTTCAGACAACATCTCAGAGTCATGAAGATGACGTGGAAATGAAACTTCAGGTGGAATCAGTTATTGAATTTGAACCTGGTTTGGACAATTTTACTTCTCGACTAATGGACATGGATTACGCTCAAGCTCGAGTGTACCTCGTTTATGCTAGCAAACAAGATGCTTTTGTTATATTCCGTGATGCTGCTTTTTTAAATATGACTGAATATGGCCACGTTTGGATTGTCACCGAACAGGCTCTTGACACAAGAAATGTGCCTGTTGGTACAATAGGGCTAAAACTTGTTCATGCCACTAATGAACGAGCTCATATACAGGATAGTATATATGTCCTTGCTTCTGCACTTCGCGAAATGAACCAGACAAGAAATATAACACAAGCTCCGCAAGATTGTGATAATTCTGGATCTATTTGGGAAACTGGAAAAATATTATTTGAATACATTAGAAAACAAGTCCTCTTGACAGGTGCAACTGGAAAAGTAGCTTTTGATGACAATGGTGATAGAATAAATGCTGAATATGACGTAGTAAATTTACAAGCTCCTGGAAAACATATATCTATTGGTCATTATTTTTATAATAGTGATCTAGATCGCATGCGTTTAAAGGTGAATGATGACAAAATTATCTGGCCTGGAAGAAGAAGAATTAAGCCTGAAGGTGTGAAGTTACAAACGCATTTAAAGGTTCTAACCATAGAAGAAAAACCTTTTGTTTATGTTAGACATTTGAAGGATGAAGTAACATGTGCTCCAGACGAAATTCTATGTCCACATTTTAATAATTCAGCACGAAATGATGGTCAAATGTATTGCTGTAAAGGTTTTTGCATGGATCTTCTCAAAGAATTATCACGAGTGATTAATTTTACTTTTGAACTAGCACTCTCTCCTGATGGACAATTTGGAAATTATGTTATAAAAAATACTTCTGGGGGCAAGAAGGAATGGACTGGATTAATAGGTGAGCTTGTATATGAAAGGGCTGATATGATAGTGGCTCCGTTAACTATTAACCCAGAAAGAGCAGAATTTATTGAATTCAGTAAACCATTTAAGTATCAAGGGATTACAATTTTAGAGAAAAAGCCATCAAGATCTTCGACGTTAGTGTCATTCTTGCAACCATTTTCTAACACCCTATGGATTCTAGTGATGATATCTGTCCATGTTGTAGCTCTGGTGCTTTATTTATTGGACAGGTTCTCTCCATTTGGTAGATTTAAGCTTGCTAACACAGACGGAACTGAAGAAGATGCTCTGAACTTATCGAGTGCAATTTGGTTTGCTTGGGGTGTCTTGCTCAATAGTGGTATTGGTGAAGGAACACCAAGAAGTTTCTCGGCCAGAGTATTAGGAATGGTTTGGGCTGGTTTTGCAATGATTATTGTTGCATCCTATACTGCCAACTTGGCTGCTTTTCTAGTGTTAGAACGTCCAAAAACTAAACTATCAGGCATTAATGATGCAAGATTGAGAAACACAATGGAAAATCTGACTTGTGCAACAGTAAAGGGCTCCGCTGTGGACATGTATTTCAGGCGTCAAGTAGAATTGTCGAATATGTACCGCACAATGGAAGCCAACAATTATGATACGGCTGAAGAAGCAATCCATGATGTTAAAATTGGGAAGTTGATGGCTTTTATATGGGATAGTTCTCGATTGGACTTTGAAGCCGCCCAAGACTGTGAATTAGTGACAGCTGGAGAACTGTTTGGTAGAAGTGGGTACGGAATTGGTCTTCAAAAAGGATCACCGTGGGCTGACAAAGTTACATTAGCAATTTTGGATTTTCATGAAAGTGGTTTCATGGAAAGTCTTGATAATAAATGGATATTGCAAGGAATTTTACAACAGTGTGAACAGTACGAGAAAACTCCAAATACTCTTGGCCTTAAAAACATGGCTGGAGTGTTTATACTGGTTGCTGTTGGTATTCTTGGAGGCATTGGTCTTATAATAATAGAAGTGGCATACAAAAAACACCAAATACGAAAGCAAAAAGAAATGGAATTGGCAAGACATGCTGCAGATAAATGGCGAGGAGTTGTAGAGAAAAGAAAAACTGTAAGAGCTGCAATAGCTGCCCAGAGGAGATTAAAAGCAAATGGCATCAATGAAACAGGAGTAAGTCTGTCTGTTGAAGCCCTTCCACGAGGTGTCCTAGATTTACCGAGCCCTGTAAGAGCCTGGCCGGGTGCCTGTCCTATTAGACAACGAACTCCAACAGATTCACCTAACCTGCCTGCATGA

>NMDAR2A

ATGGTCGCCCCGGGTTTGTTGTTGGCGGCGGTACTGTGTCTATTGTCGGTGTCGCCACCACAACGAGGCGGCGTCACCGGACAACGCAGGGGCGGGACCGCGTCCAGGCCGGCACAATCTCGTCCCGGCACCCCAGGCCATTCTGGGTCCAACATTACAATAGGTTTAATATTACCTAAAACATTGTTCGGCGCCAGAAGCTACAATCGTGCAATGATTGAAGCAGTTAATGGATTGTATAAACCTAGAGGTCGTAAACTTAGTTTCCTCAGAAGATATGCGTTCACCGTTGGACAGGTCAACACACAAATGATGGAGCTTACACCAAGTCCTACAATGTTAGGAAAAGTATTAAGATGTATTTCAAAATTCATTAAAGTTGAAATAACTATATTGAATTCTTTGTGCAAAGAGTTCTTGTCGGTGAATGTATCTGCCATTCTGTACCTGATGAACTATGAAAAATATGGCCGCAGTACAGCGTCAGCTCAATACTTTCTCCAGCTGGCCGGCTATTTAGGAATTCCAGTCATTGCGTGGAATGCGGACAATTCAGGCTTGGAAAGGCGCGCCTCTCAATCAAGCTTACAACTCCAGTTGGCTCCATCTCTGGAACATCAAACTGCTGCCATGTTATCAATTTTAGAGCGATATAAATGGCATAGATTTAGTGTGGTTACTTCGCAGATTGCTGGACATGATGACTTTATCCAAGCCATTAGAGAAAGAATAACCGAAGTACAAGACAGATTCAACACCATTTTAAACTCCGTTCTTGTGACCCAGTCATCTGATCTGATGGAACTAGTCAACTCAGAATCTCGAGTCATGCTGATGTACGCCACACGAGAAGAAGCTGTACATATTCTGGCCGCTGCGCAAGATTTTGGCATTACTGGAGAAAATTACGTTTGGGTTGTCACGCAGTCTGTCATTGAGAAGCAAATGAATCCTGCACATCAATTTCCAGTCGGCATGCTCGGTGTCCATTTCGATACATCTTCAGAACGTTTGCTGAGTGAAATAACCACTGCCATCAAGGTATATGCAAATGGAGTTGAAGACTTCGTCAACGATCCTAAAAACGCCGACTTCTCGTTGAACACGCAACTATCTTGTGAAGGCGGAGCAGGAGATTCAAGATGGAAAACCGGAGACAAGTTTTATAGGTACCTAAGAAATGTGAGCGTGGAAGCAGAACAGGGAAAACCGAATGTAGAATTCACAGCAGATGGAGTTTTGAAAGCAGCAGAACTGAAGATAATGAACCTTCGACCGGGAGTAAGCAAACAACTAGTGTGGGAAGAGATAGGAGTGTGGAAATCTTGGCAAAAGGAAGGACTGGACATAAAAGACATAGTATGGCCAGGTAATTCTCATACGCCACCACAAGGAGTGCCAGAGAAGTTTCATCTTAGGATTACCTTCCTAGAAGAACCACCTTATATCACCCTTGGACCGCCAGACCCTGTCACTGGGAAATGTTCAATGAACAGAGGAGTAACTTGCCGAGTAACCACAGAAACGCAAGTTCAAGAGTTATCACTTCTATGCGATCCAGGAGAAGGATATCTCCTGGGGGTAGGTGATCGCATTCTAGATCTCCTTCTTAATCTATCTGGCGATAATCTTTCATCTGAACTATCATCATACCTGCCAATACTTGAACGAGATCTTTTGCTCCTAATGGGCGGAGAGTCCCTGCTGGAACTTCTTCTCTTCATTGATAATTTTGCTTTATCACTTCTACCGCTCATATATAGCTCTTTAAAAACTATGAAAAAT

>NMDAR2B-NTE

GCTTCGCACATCAATGGAACTCAAAATCGTTGTTGTTCTGGATTCTGTATTGATCTTCTTCAGAAGTTTGCTGAAGAGCTGGGCTTCACTTATGAGTTAGCCAGAGTTGAAGACGGTCGATGGGGCACATTAGAGAATGGTAAATGGAACGGACTTATTGCTGACCTAGTCAATCGAAAGACAGATATGGTAATGACATCATTGATGATAAATTCTGAAAGGGAAGCGGTGGTTGATTTCTCTGTTCCATTCATGGAGACTGGTATTGCAATTATTGTCGCCAAAAGGACCGGTATCATTTCACCCACTGCTTTTCTAGAACCTTTTGATACAGCGTCTTGGATGCTTGTTGGTGTTGTCGCCATACAAGCTGCTACGTTTACCATATTTCTGTTCGAGTGGCTAAGTCCAAGTGGTTTCAACATGAAGGCGAGTTCGGCTACGAGTCACAGGTTTTCCCTGTTCAGAACCTATTGGCTGGTTTGGGCTGTTCTGTTTCAGGCTGCGGTACATGTTGATTCGCCGAGAGGATTTACATCTAAGTTTATGACAAACATGTGGGCAATGTTTGCTGTAGTTTTTCTGGCCATCTACACTGCTAATCTGGCTGCTTTCATGATCACTAGGGAAGAGTACCACGAGTTTTCTGGCATAGACGATAACCGAGTATCACAACCCTATAGTCATAAGCCAATGTTCAAATTTGGTACTATTCCATGGAGTCATACAGATTCAACATTAAAGAAATATTTTAAAGAAATGCATTCTTATATGAAGATATACAACAAATCTTCAGTTACTTCAGGAGTGGAAGCAGTTGTCAGCGGGGAACTGGATGCTTTCATTTATGATGGAACAGTCCTTGATTATCTTGTTGCCCAAGATGAAGATTGTCGACTTTTGACAGTCGGCTCTTGGTATGCCAAAACTGGATATGGGCTTGCTTTTACTAGAAATTCCAAGTATCTCCCTATGTTTAACAAAAGACTTTTAGACTTGAGGGAAAACGGTGACTTAGAAAGATTGCGTAGATACTGGATGACAGGTACATGTAAACCAGGTAAACAAGAGCACAAATCTTCTGATCCATTGGCTTTGGAACAATTCTTGTCAGCTTTTTTGTTATTGATGGCTGGAATTCTATTAGCAGCTATATTGCTTCTCTTAGAATACCTGTACTTCAGGTATGTTAGAAAACACCTGGCTAAAACTGATCGTGGAGGATGTTGTGCCCTAGTTTCGTTGAGCATGGGAAAGTCCTTGACGTTTCGTGGAGCAGTTTATGAAGCGCAAGATATATTACGTCACCATCGTTGCCATGATCCCATCTGCGACACTCATCTTTGGAAAGTTAGGCGAGAACTAGACATGGCAAGATTAAGAATAAGACAGTTAGAGAAAGAAATGGAATCACATGGCATTAAACCTCCTAACAGGTTGGCTTGTTATGACCGTTATATTACAAATTCTGAAATGCTCAGACCTAGAGACATGACAAGGAATCATATCACCACTACAGAGTTTGAAGGAAGGTTCTGTGCATCAACTCCTCAACTCTATAGGTTTGTAATTTTAAGGAAAAATTATTTTAA

>CHLORIDE_CHANNEL_B-FX

ATGGGTGAACAAGCAGAAAACGAACCGTTACTGAAAGGTAACAATAGTACGCAAAAATCGTATGGCATCAACGTTGATGTAATTACAAAATGTAACGAGGTATCTCCAGAAACTTCGGCCAAAACCCGCCCGAGAAGAAGGGCTAAGTTATCTGAAACAATTGTGGTTGGTACGTTGGACGTACTTTCTGCTAAATATGAGAGTTTGGACTATGATACCTGTGAAAATCATTTACTGCTTGAAGAGGAACGAAAAAAAGGTTATCAATTTTTCGTTAAAAAAACCATGGCCAGATGGATCATATTTCTTTTCATTGGCGTGATTACAGCATTAATTGCTGTTTGTATAGATATATCCATTGACAAGATATCAAGTTTAAAATTCAAACTTATTAAGGAATACATTGACCATTGTATGTCTGAAGACTGTTTGTACGTGCCTTATTTGGTTTGGTTAGGTTTAAATGTTGGAATGGTATTGGTCGGCTCGTTCTTGGTATCATATATTGAGCCAATTGCAGGAGGCAGCGGTATACCACAGGTCAAATGCTATCTGAATGGCGTCAATGTGCCAAGACTTGTCCGAATTAAGACACTCGTTGTCAAGGTGATTGGCGTGATATCAACCGTTGTTGGCGGATTGGCCGGTGGAAAGGAAGGCCCCATGATACACGCTGGGGCAGTCGTAGCCGCTGGTATCTCACAAGGAAAGAGTACTTCACTTAATAAAGATTTTGGAATTCTACGCTACTTCAGAGAAGACCATGAAAAGAGGGATTTTGTGTGCGGCGGAGCTGCAGCTGGTGTTGCTGCGGCCTTCGGAGCTCCGGTAGGTGGCGTATTGTTCAGTTTAGAGGAAGGAAGTAGTTTTTGGAATCAAAGTTTGACTTGGCGAATATTCTTTGCCTCTTCAGTTTGTACATTTACTCTAAACATCATTCTAAGTACATACCATGGACATCCAGGCGAGCTTACATATTACGGTTTACTGAACTTCGGCCATTTTGAATCATTAAACTATGAACTATTTGAATTGATTTTCTACGCCATCATGGGTGTAATCGGTGGTGTTTTAGGATCATTTTATACTTATGTTAACTATAAACTAACAGTATTCAGAATGAGATATATCCGTGCAAGATTTCTTAAAGTATTCGAAGCCTGTCTAGTGGCTGCAGTCAGTGCAACTGTTGGTTTGCTCATGATTTTTGCACTCAACGATTGTAAACCTTTAGGACAGGATCCTACCCAATATCCCGTACAGATGTATTGTGGAGATGGTGAGTATAATTCTGCGGCTGCTATTTGGTTACAAGTACCAGAAGCAAGTGTTCGTTCATTGTTCCATGATCCTCCAGCATCTCATAAAGTAAGCACTCTGTTTCTGTTCATGATTGCTTATTTGTTCCTTTCAATTTGGACGTATGGTTTAAGTGTGTCCGCTGGAATATTCATACCTTGCCTTTTGACTGGTGCTGCTTGGGGTAGAGTAGTTGGAATATTGATTGGCTCCGTATTACCGAATACTTCCTGGGTAGATCCTGGTAAATACGCTCTAGTGGGAGCCGCTGCTCAATTAGGTGGAGTAGTCCGAATGACAATAAGTCTGACGGTGATCTTGATTGAAGCTACTGGAAATATTACCTTCGGTTTACCATTGATGATCACCCTGATGACTGCCAAATGGGTTGGTGAATTTTTTTGTGAGGGTTTCTACGATCTACATATTCAACTAAGCGGTGTGCCATTACTCGGATGGGAGCCTCCACCATTATCGTCCACCTTGTACGCAAGCAAGGTTATGAGTTACCCTGTGCTGGCTTTCGCTCCGGTAGAATCAGTTCGTAACATTGTAGAAGCGCTCACCAATCAAACTTTTAACGGATTTCCAATTGTGGATAGCGTTCATCATCTAGAATGCCCGGCTGAGGTTAATTCAGTCGGCCGTTTACGAGGCCTAATACTACGATCTCAGCTTATAGTTTTATTACATAATAAAGTGTTCGTTGAACGAACTGAAGACACTGACCATGAAGTTACTGTAAAGACTTTTAGAGATAAGTACCCAAGGTATCCTGAGATTAAGGATATTTATATTACTGAAGAGGAAATGGATTGTACTATTGATCTTACTCCATTTATGAATCCGTCTCCGTATACAGTTCAACAGGTCGCATCTTTGCCACGCATCTTTAGACTTTTCCGAGCTTTAGGACTTCGACACTTAATTATAGTCAATGAAGTTAATGAGGTCGTTGGAATGGTAACCAGAAAAGATTTGGCCAGATACCGTGTATGGAAACATTTTGGTAATATGGGCATGGAAGAGCTGAAGATCAGTAAAGGGTTGTAA

>CHLORIDE_CHANNEL_B2-FX

ATGTTAACTGTGGTGGATAATACACACATCTTTGCATTCATGTCTGGGCTATTTAATATGGCACAAGGAAAACTAAGCAGGTTCTTCAATTATAAGAACCCATCGTCATCTGCTAGTTCTTCCTCATCAAGATTACGCACAGTAAGGGCAAAATCCGGTTCATTAAGCTATATTTCATCCAAATATGAGAGTCTGGACTATGATACCTGTGAGAATCAACTACTAGTAGACGAAGAGAGGACAAGAGGGTATACCTACATTGTTCAGACCGATGTTGCGAGATGGTTCCTTTTCTTTTTGATAGGCATCTTTACTGCAGCCCTTGGTGTTTTCACTGATGTTTCCATCGCCTACCTCGCTGATCTCAAATATAATATTCTTAAATACTATATGGATAAATGTCTCATTGAGCAATGTATATATGTTACGTACGGCATGTGGTTGACATTAGTTATGGTGCCTGTACTAGTAGCATCAATTCTGGTAGTTTATATCGAGCCTGCAGCATCAGGAAGCGGTGTGCCTGCTGTAATAGGATATTTAAACGGAGTGAAAATGCCGCACGTTGTGGAAGTGAAGGTGTTGATTGTGAGGATGCTGAGCACGATCGCCACATGTGTAGGCGGATTGGCCGGGGGCAAGGAGGGTCCAATGATACATTCAGGCGCAATAGTAGGCGGATCGCTGGCCACATTATGGATACCTGGTTTAGGACCACATTCTGTACTGCACTACTTTAGGCAAGATCATGAGAAAAGAGATTTTGTAGCCGGAGGATCAGCTGCAGGGGTTGCTGCAGCATTTGGAGCGCCAGTTGGAGGTGTTTTATTAAGTTTAGAAGAAGGAACCAGCTTTTGGAGTTTATCCCTGATCTGGAAAATATTTCTATCATCTATGGTGGCAACATTTACTCTAAATCTATCGCTAAGCGCCGTTGAAGGGCATCCAGGGCAATTAACCCATCCCGGATTGTTGTTTTTTGGAAAGTTGAATGATACAATAACAAAGTATGAAGTATTTGAACTGCCCATCTTCATCCTGATGGGCGTAATTGGAGGTCTTCTTGGCGCTGTTTTCGTTGCAATCAATATGAGACTTATGGTCTTCAGAATGAAGCACATCAAGAAGAAATGGGTTAAACTATTGGAAGCTCTTACTGTGGGTTTTTTGACTGCATCTTGCGGAATGGCTTCAATATATTCACTCAATTATTGTAGAGACTATACAGAAGCTAGCCCAAAGTTTGCTGTTCAGATGTTTTGCAGCGAGAGCAGACATAATGAACTTAGCGCTTTTTGGTTTCAAAAATCAGAAGACACACTTAGATCTCTCTTTCATGATCCCAAAGGCTCATATAGTGTAGTTCCTTTAGTAGTTTTCTTCCTCATGTATTTCTGCATGTCAATCATGACAACTGGCCTAAGTGTGAGTGCAGGAGTGTTTGTACCCGCTTTACTTTCTGGTGCAGCCTGGGGCCGGCTTATAGGAATTGGCATACAACATGTATTCCCTGAAGCTACGTGGGCTGAACCAGCGAAGTATGCAGTAATAGGAGCAGCAGCGCATCTTGGTGGAATTTGTAGGATATCAATATCATTAACAGTAATGATGATAGAAGCAACTGGCAATATTACATTCGGTCTCCCTTTAATGTTAACTCTTATCACTACAAAATGGATTGGAGATTTTTTTACAGAGGGAGTATACGAAATGCAAATTTATTTGAATGGTGTGCCATTACTGCCCTCAGATCCACCACCTTTATCTTCTGATATCAAGGCAACAGATGTGATGTCTGCCCCTCCTGTAGTTTTCCCCTCTAAAGTTAAAGTTGCCCGTATAATAGATACTTTAGATAATGTCCCTCATAATGGTTTTCCCATCGTTGACCCCTCTATTTCCACTAGCCATGGTGTATTAAAATCAGCTGGTAGACTGAAAGGTCTCATATTGAAATCTCAGCTATTGATTTTATTGCACAGTAAATCATTTAATGAGCTTGCCCCAACGACCAGTGATCAATTACGGAAAAAATTGCATATGTTCAGAGAAGCTTATGCTAAATATCAAAAAATTCAGTTACGTTCAGGATGGATAGAGGATGTGCAGATAACAGAGGAAGATTACAATCAGACACTGGATCTGCGGCCATATATGAATCCTTCTCCTTATACTGTTAAATTGGATGCATCACTGCCAAGAATTTTTAGGCTTTTTCGAGGATTAGGACTTAGGCATATAATTGTGGTGAACGATATAAATGAAGTAGTTGGTATAGTTACAAGAAAGGACTTAGCAAGGTACCGCACGTGGAGACATGCAGGTGCGATGGGCCTAAAAGAATTGAGAGTACAAGTATGA

>MRITYU-FX

ATGGCAAAACCTAAAGACAGGCATAATTATGATAGGGACACCAGTAGTGACACTGAAGATTATCATGATCCAGAAGAACACCGAAGAAGAGTATTCAAAAATCGAATGAGGTCTCGGACCAATCACCCCATGTGTCCAGCAAAGAACCAGATGAATCAGCAAAGCCAGACATCAGCAAGTACCAGTAATAATGTCAGTGAAGAGAGAATTACTCTTGTGGTTGATAACACTCGATTCATTCTTGATCCAGCCATTTTCACTGCTCATCCAAACACCATGCTTGGAAGGATGTTCAGTTCGGGGCTAGAATTCACACATCCAAATGAACGAGGTGAATACGAAGTTGCAGAAGGAATTTCAGCTACAGTTTTTAGATCAATATTAGAATACTACAAAGGAAAGATGATCAAATGTCCTCCTACCGTTCCAGTGCAAGAACTAAGAGAAGCTTGTGACTACCTCCTTGTGCCTTTTGATGCATCCACCGTGAAGTGCCAGAACCTCAGAGGTCTTTTGCATGAACTATCTAATGAAGGTGCAAGGTGCCAATTTGAAGTATTTCTAGAGGAATTAATACTTCCGTTGATGGTGAATTCTGCTGAACGTGGAGATCGTGAATGCCATGTTGTAGTTTTGTTGGATGATGATTCTGTTGATTGGGATGAAGAATATCCACCGCAAATGGGAGAAGAATATTCTCAGACGGTTAACAGCACAGCCATGTATAGATTTTTCAAATATATAGAAAATCGGGATGTCGCAAAACAAGTAATGAAGGAACGTGGCCTGAAAAAAATTCGGCTTGGTATTGAAGGTTATCCGACGCACAAAGAAAAAATAAAAAAACGGCCTGGAGGCCGAGCAGAAGTAATTTACAATTATGTACAACGTCCGTTCATACACATGTCTTGGGAAAAAGAAGAAGCTAAAAGTAGACATGTTGATTTTCAGTGCGTAAAATCCAAGAGTGTAACGAACTTGGCGGAGGCAACAGCTGATCCAGTACTAGAGTTAGATGCTGGAGGAAATCCAATTGGTGGAGGTCCGGGTGCAATGGTCGAAGTACTTCCACAGGCCGTTCCACAAGATTTGGAAGACGCAGCTTTTGGGGCAGTTGAACAAGCACCGCCCTTACCACCTCCATTTCCACCAGAGTAA

>PIEZO-FX

ATGACGCGGTGGTTAATTTCTCTTCTTTTACAAAGGATTCTCCTACCTTTAGCTCTGGTAGCTGGCGTGGCGATCAGGCAAAATGGATTATCCGGAATCTATCTACTTCTTCTGCTTATTCTCCCTTTCATACCAGTGCCTACCTCGAAAACCATGTCTGGTCACGCTGGACGTTACCAGAAATGTGTAATAACACTGACTATTTTAAATATAATTAGTGTAGTCGCATTTCAATTCGTTTTAATCGCTTATCCTCCGTATGGCCGATTAGTTAAACAGCAAGGTAGTTTTTTGGAGAAATACCTGAGATACGCTGGTTTGGTACGTTTAGACGGACTGAATTGGTTAGATTGTCTAAAAATACTTCTACCAGAAGCTTTAGTTTTAATTGCAGCCATAATTACGTATGTTGTTTCTCTACGACTTAATCCGGCTAGCCTAGAAGATCAACTACTTCCAGTAGCTGGACCACATAGTTCACATCCTCCTCAAACAGAAGGCGATAAAGCAGCTGCGCAAAGGAAACTGTCATTACTTACTTCATTTGGAAAATATGTGTGCCTAGTTTCGCTATGTTTTGCTGGGATATTCCGTCCATCAGTGCTATCAGGAATCTATTTCCTTGTCTTTCTTGGAAGTATGACCTGGTGGGGTCTGAACAAGACTCTTGGCAATCCATTTGCATGGATTTGTAGAAGTCTATTGATTGTTTTGGCTGGTCATATTATAGCTTTATTTCTATATCAGATGGAGTGGGCTCAACTACTTCTTCCTAGTGATCATCCGGTTGCCAGATATTTAGGATTAAGTGCTATTATGGTAACCGATACAGAGGATCCTAGAATAGTTAACATTTCATCTCTTGAATGGGATTCATTTGTTAATCCCTTTACCTTAATTTGGCTGTATTTCATTATCTGCTACGAATCTAATCTGATAATAAATGCACCCCTAATTCGGGGTAGAGAATCAATATATAGAAGAGGTACCTCTAGAAGAGGATTAATTCAAGATTCCATGGGAAGTGTGACAATTACTGAAGTTCCTGAAGAAACGATTAAACAAGAAATTACTCCCGAAGAAGAAACAGGTCCTGGTGTACTGGAAATAGTATTAAATGCTGGAATAACAATATCACAATTGCTGGCTCAATCTTCTTATATTGCTACAAATATTATTATGATGGCGTGGAGTATAACTTATCACAGTTGGTTGACGTTTATCTTGCTTCTGTGGGCTAGTATAATGTGGTTGATGCCAAACCAACGGAAAGCCATGTTACGATCATCACCTTTTCTAGTTTTTTACGCTACATGTCTACTTTTAGTCCAATATGTTTATGGTATGAATCTTACAAACAGTGAACTACCGCAGATCATCCAGGGAATTAATCTGAAACAAATCGGTTTAGAGAAGATTCTACACTTTCCGTTCAAACCATTGATAATAAAGATACTTTTTACTGTAATGTTTTGGATAACATTGAAACAATATAACCAAGAAAAATGGGAAGCGAGGAACCAGTCCGCTCTTGCCGATATTGCGGCTCCTTTACAAATTGGAGTAGGCACGGCCACAGGTGCTGCTGGAGAATCGCAGACCAGAACTAATCTATTAGTTAAACAACTTGGTACAATCTGCCGGTCAGTTTTAACCAAATTCTGGATATGGATAGTTGCAATCATACTCTTTACGATTGGTATATCAGGAGATCGTGTGACCATTTTTAGGATCATTTATATGGCACTGGCATTAATATTTATTGTGACATTCCAGTTATCCTGGACATTTTGGAGGAAAATGATGTATGGCTTCTGGTTGGTACTAATTCTATATTCTATGCTCATATTGGTAATGACGTATACATACCAATTTGATAATTTTGATACATATTGGGAACAATATCTTCATACACCAAAACAATTGCAAAAAGATATTGGTCTTGAACGTTACGAGACCACAGAACTATTTGTTAAACTTCTTACACCGACATTTTTTGTCGTAATCACTGTGATCCAATTGTATTATTTTCATAATGACTTTTTGGCCATCTCCGATATAAAAAGCAGAGGAAGTTCAATAAGACCTCGTATTTCGGATGTAAGTAATTTACCTCCAAGATCAGATTTCAGTAGTTCAGCTGGGCCGGAGTTAGTTACTCCAATAGACAAAAATGATGACATAAATTCTATAACGGAAAAACCAAAGAAAAAACGCATAAGTATACGAAAAGCCATAACTGAAGTGCGTCTTAAAGAGTTTACTTCATTTTTATTAAGGCAAACCGTGGCGATAATAGAACTAGTATGGCTATTTCTTGAACTTCATATGTTAAAGTTAGTTTTGTTAGCTTTAATGGTACTATCGGTTTATGATGTATGTGTACTGCATATTGCTTTCGTAGCATTAGTTGTCGCGGGAACAATGTTTGGCACTAGAATACAAACACTGGTCGTCCATACTTCCTCTTTCCTTATTTCCATATTGTTTTTATCGAAAATGATCTATCAGATTAATTATATCAATCACGATTATTGGAATGTGACATGTCCTACGGATAATTACACTTATAATAGTGCAGAATGGATTGGGTATTTTAAAGTCAATGATACTACAACTCTTCCAATGTTGCTCAGAAGTTACATAATTGTGATGCTGGCAATAACTTTTAATTCAGTAATTATTAGACGGCAGAAGTATCAACGCCACTTGAGAGGACGTCAATTGAGTAGGCCTTTTATAATGTTCCCAAGAGTAAATTACAGAGATGTAGATACAGATCTGTTGAGTTGCATAAAATATCTATTCAATTATGGTTTCTACAGATTTGGTGTTGAGATTTGTTTAATCGCTATAGTTACTCTAATTGGACTGAGAATGGATTTTTATGCAGTGCTGTATGCATTTTGGATTTGCCTTCTAATTACGCCCAATAGAAGGGTATTATCAAGTATATGGCCATTTTTTACACTTTTTATAGTAGTAACAATACCATTTCAGTATGCTCTAGCCGTTGGCTTTCCACCTTCGTTGTGTTTCGTTTATCCTTGGGATCGTTCTGAATCTCTCGAAAAGATGCAAGAGTGGATGTTCTTTCCGGACTATCAACACCCACCGCCTGCCTATAAGCTATTATGTGATTTTGTAGTATTATTACTAGTATGCCGACAAGGTGTGGTTTTCAGAATTGAAAAACGTCATAGAAACATGGATTACCCTGGTGGGACGAATAAGAGTATTATTCATCGATTTGAGGAGCCCAACTTTGCAAATCCCACACCAGATTTTGTATCCTTTACAAGATCATGGCTGGATGTGGCTAAAAGAATAATCCTTTCTGCATTCATCTGGATAACATTAGCAATAGTATTTCTTGCTGGAACTAATAGGGTTAATCTATTCTCTTTGGGATATTTAATTGGTGCTTTTATATTTCTTTGGCAAGGAAATGATTTCTACCTTCGCCCAATTCGTTCAATATTAAAATGGTGGAATTTCCTAATTGGTTATAACGTTTCAGTTATTTTGATAAAAGCACTGTTGCAAATTTTAGGATGCATTTATTTCAAGGAAATACAATCGCAAGCTTGTTGGGCAGTTCAGATTTTAGGAATCGTTTGTATACAAAAATTTTCTCCTGGATCAAAAGAATCTTTTGATTCAGCAGATTGTTCATTGCCGCGTGAAGACGCTGGCCTAGCTTGGGACGGTTGTTGTTTCTTTTTCCTCATCTTGCAAAGAAGACTCTTCAATAGTTATTATTTCTTTCATATTGTTGATGAAACCAAGGCAATGACGGTTCTTGCCTCTAGAGGAGCTGAACTTATTGAGGACTTAGCACGCAGACAAGTAATGGAACAACAAGAATCTGAGAAGAAAATACTTGAGAAAATTAAAAATAAAATGGATAGAATTAAAGCTTCACAGAAAAGAATTCAAGGGCCTAATTTTCAAGAACCCACTAGCCATTATGTTGCAATCAGATCGGGAGATTACTACATGTTTGAAGATGTTGAAGAGGAAGTTGATTTACTATTGGATGAAGAGAGTTCTGAATCTTCAACAGAATTGAGTGATGAAGCGATTGATCATAGAAGATCTAATGTGGGGCAGTTGCTAAAGGCTGCATTGAAGACAGATTTACGTAAAGCAGTTGAAATAGCTACCGAACAGAATCGTGAAGCAGACAAAACACTTAGAAGAGATCGCCACTCTAGGGATGGCGATACGCTCAGACGAAGACGCAATCTTTCGTTGATGATTCCTCCGACTCATGAACGTGAACGATGGAGAAGAAGCGCCACTTCATTGCAAGAAACTTCTCAAATTCAAGCAATTGCTGGACCTAGTGGTATTACTCCATACCAAGATAGAGAAGATGATGAAGGGGATGATGGAAGTAGAGAAGATGAAGATCGTAAAAAAGAAGTAGAAGTTGAAAAAAAAGATGAAGCAATTGAAGAAACAGACGAACCACAACCTCCGTGGAAAGAAAAATTAATAACCTTCTTGAAATTTCTTTGGGCCTTCATTGACTCATTGATGGTTTCCATCACCAATACATTGAATAAATTCTCTAGAGATTATCGTTATGTAATGCGAACTTTGTCTGTTGAAAAGAAACAGTTAAAGACAAGCAAAGATTTTGGAATCGGTTTGAGACCAAGTCCTGGCCATATATGGCAGCCTCTACCATTAGCAGAACTGTCTAAACCAAAATCAAGCCCAGTATCAATAGGAGAATTCGAGGAACAAGGAGAACTTACTGCTTCTGAACAGCCGCCATTCTTTCGTCTCATATTGGCCATCTGGTATGCCGTACTCTCTCATTCGGAGTTGGTCTGCTATTTTACTATATTTCTCCTTCAGATACGTTCGCCAACTATACTTTCATTGCCGCTGCCTTTAATGGTGTTTCTATGGGCTACGCTAACAGTACCCCGGCCGTCCAAGAAGTTCTGGGTGACAATAATCGCATATACTGAGGTAGTGGTGGTTGTAAAATGTATGTTTCAATTTGAAATATTACCTTGGAATCAAAAATTGATCCCTGACAATGCTCCATTTGCACTACCTAGAATCATAGGTATTGAAAGAAAACCTGGAATGTATGCCTATTACGACTTATTCTTACTTCTTGTAGTCTTTTTTCACAGATCTATGCTCAAATCTCTGGGTCTATGGAAAGAGACTGCCCAAAATTTTCCTGTAAAAAGTAGCAAAGATAGTCCATCTTCTGAAACTACAGAACTTAGCGGAAGTGAAAATACTCAAGCTAGTAAAAAGGATAATTCAAGTAAAGATACTACTGTGAAGGATTTGGTATTAATTGGTGATACTGGTTCAACAGGGCCACGAGAGTATTTACCACCTTTACCATTAAAGAGAAATCAAATATGCCGACCTTGTCATAAATTCTTTGGCAATTTAATCGAATCAAAAGGTCGCGTTACTACAGATGTATATGCGTACATGTTTTTCTGTGATTTCTTCAATTTCCTTGTTGTTATCTTTGGATTTGCAGCATTTGGGTCTCAACAAGGTGACGGAGGTGTTTCTCAATATTTTGAGGAGAATAAAGTTCCTATAGCTTTTCTGGTTATGTTAATACTCCAGTTTGGACTGATTATTATTGACAGAACTTTGTATTTAAGAAAATTTATATTAGGAAAAATAATATTTCAATTTGTATTAGTAATTGGCATACATGTGTGGATGTTCTTCGTCTTGCCAGCAGTCACAGACAGACAATTTAATGCAGCACTGCCACCACAAATGTGGTACATGGTAAAATGTTTCTATTTATTACTCTCGGCCTACCAGATCAGATGTGGATATCCTACTCGCATCTTTGGAAACTTTCTCTGCAAGAGTTACAACTACATAAACATGTTCCTGTTCAAGGTTTTTATGGCTGTACCATTTGTATTCGAATTAAGAGCATTGATGGATTGGATTTGGACGGACACCTCAATGACATTATCTGATTGGTTGAAAATGGAAGATATATTTGCTCATGTCTTTCAGCTCAAGTGTCAAAGAAGAGCCGAAAAAGATTATCCCCAACCACGAGGAGAAAAGAAGAACAACTTAATCAAATACCTAGTGGGAGGAAGTTGTCTTATCGGCATAATAGCAGTAATCTGGTTTCCACTTGTACTGTTCGCTCTGGGAAACACTGTGGGTCAACCAAATATTCCTACAGAAGTGACACTGTCTCTCAGTATTGGAGCTTACACACCTATTTACCGTGGTACAGCTCAGAATGATTCCATTTTCAACTTAGAAAAAGAAATGTGGGACAACATGTTGAATGTATATAAGAAATCGCGAGCAGCACAGACATTTCTGTCAAATTATGAGTATGATGATGTCGGCGTGGCTATACTTGGATCCCATTCTACTGTTGTGTGGACGATATCGCCACCGGACAAACTTTCTTTAATTAATGATTTAAGATCAGGTGCACCAATGTCAATCAAATTAGAATGGACGATAAGTAGAAAGTCGACAATCCCTGATCAGCCGGCTATGGTTTCAAACAGCATACAGTCTTTATTGCCCATCGGTCCGAATAGAGAGATCATAGCTGACTTTATAACGGGCAATGAAACTGAAAATATGGCGATCCTGCCTAATCTGCTACCAAAATTTGTGAAAGTAACCAGCCGAGGAACGGCAAATGTTATTAATCAGCTTATGTGGTTCGATGACGATGCCCAGGCGGAAGATCCTTATCGACCACTTACCGCAAAACTTATTGGCAAGGCTGAACAATTTTGGTGGGAAGTAGAAGAAAAATGCGGTGATCCAGTTTATGAAAATTACCTCTCTAAATTGAGTTACATAAATTGTAACCGACTCGTTATGTATACTTTTAATGATAAAGCTTTTCCAGCAACTTTAAATATTATTTCTGGAAAAGGGATCATTGGACTGTACACTACATTTGTGATTGTAATACACTCTGTTATACGTGGTTTCTTTACTGGAATATCGTTCAAGATAATGTTTGATGATATGCCAAACGTGGACCGTTTACTACAGCTTTGCTTGGATATTTATTTGGTGCGAGAAAGTGGTGAACTAGATTTAGAAGAAGATCTATTTGCCAAATTGGTATTCCTTTATCGTTCACCGGAAACGTTGATCAAATGGACACGACCGCCTGAAGAAGTTGGTGACGATGAAGTGGACGCCCAACCAGAACTGAATAATTAA

>NARROW_ABDOMEN

ATGATGCTTGGCAGAAAACAGAGTTTAAAAGGGGATCAAATCCTTGCTGATTATGGTCCCGAAGAATCTTTGAACGAAAGTGCAGATATTGAATGGGTTAATAAAAGATGGGTACGAAGGTTGATGAGATCTTGTGCTTTAGTAAGTCTAGTATCAGTTAGCTTAAATACTCCTAAAACATTTGAGAGATTTCCTCCACTTCAGTATGTGACCTTCTGTTCAGATTTGATTATAACATTTCTATTTACTACTGAAATGATTGCTAAAATGCATATTAGAGGAATACTAAAGGGTGATGTGCCATATTTAAAAGATCATTGGTGTCAATTTGATGCAAGTATGGTGTTCTTCTTGTGGGTATCAGTAATATTACAAATGTTTGAGTTGCTGAGCATCGTTCCAAAGTTTAGTTATTTATCGTTTCTGCGAGCTCCTAGGCCATTAATTATGATTCGCTTTATAAGAGTATTTCTTAAATTTTCAATGCCCAAATCTAGAATTAATCAGATATTCAAGCGATCAAGTCAACAAATCTACAATGTAACTCTATTTTTTTTATTCTTCATGTCACTTTACGGTCTTCTGGGTGTTCAGTTATTTGGCGAATTGAAAAATCACTGCGTTTTGAACACCACTGAGCCTGAGTATATAACGTTAAATAGTTTGGCAATTCCTGATACATTTTGCTCTACTGATCCAGATTCTGGATATCAATGTCCGGCTGGAATGAAATGTATGAAACTAGAGCTAGATAGATATGTAATGGGGTTCAATGGTTTTGATGAATTTGCTACAAGCATTTTTACGGTTTACCAGGCTGCTTCGCAAGAAGGTTGGGTGTTCATTATGTATAGAGCAATAGACTCGTTACCCGGTTGGCGAGCTAGTGTTTACTTTAGTACTATGATCTTCTTTCTTGCATGGCTGGTGAAAAATGTATTTATTGCCGTCATCACGGAGACATTCAATGAAATTCGAGTACAATTTCAGCAGATGTGGGGCATACGTGGACACATGACCAATAAATCAGCTTCTCAGATTTTAACTGGAGACGATCTAGGATGGAAATTGATAACATTAGATGAAAATAAGTACAGTGGTTTAGCACCACCAATTTGTCAGACTATTCTACGAAGTGCTTCATTTAGATTGTTAATGATGGGAGTTATTTTAGCAAATGGTATTGTTACGGCAACTATGCATTTCAAACACGATGGTAGACCGCGACATGTTTTTTATGAAAATTACTATTATATTGAGGTTGCATTTACCATATTTTTGAATCTGGAAGCAATATTTAAAATTTGGTGCCTTGGCTTTAGAGGATATTTCAATCAATCTTATCATAAATTTGAACTTTTATTATCCATTGGTACAACTTTGCACATAATACCTTCACTATATCTGTCGCCATTAACATATTTTCAGGTACTACGCATTTTACGATTAGTGAAAGCATCACCGATGCTTGAAGATTTTGTGTATAAAATATTTGGTCCTGGCAAAAAATTAGGGAGTTTAATTATATTTACAATGTGTTTGTTGATTATATCATCTAGTATATCGATGCAATTATTTTGTTTTCTTTTGGATTTTACAAAATTTGAAAGTTTCCCAGAAGCTTTCATGTCGATGTTTCAAATTTTGACACAAGAAGCTTGGGTAGAAGTTATGGATGAAACCATGTTAAGAACAAATGAAACACTGGCACCATTAGTTGCAATTTACTTCATACTTTACCATCTGTTTGTTACTCTGATTGTGCTGAGTTTGTTTGTAGCCGTAATTTTAGATAATTTAGAACTTGATGAAGATATAAAAAAATTAAAACAGTTAAAATTTCGTGAACAAAGTGCAGAAATTAAAGAAACCCTACCATTCAGATTGAGGGTTTTTGAAAAGTTTCCAGATAGTCCACAAATGACTATACTTCATAAAGTTTCTTCTGAATTTATGTTGCCCAAGGTTAGAGAAAGTTTCATGAGGCAATTTGTACTTGAAGTGGAAGAAGATGAGAATGATAGTTTGAAAAAAATTTGCGAACCGTACGATACAAAAATTGTTTATCGCAAACAGCGTAGCATTAAATTGCTGAATCCGCTTGTTAAAGTGCGATATATCAGTACTGATCTGCGCAAGGCAGCAATTACAAATATTATCAACGATTCAAACAACCAGAGACTTATGCTTGGTGATTCAGCAGTGATTCCTGTTCCTGGCGGTAAGGCTGCTTTACACTCGCAGGGTACAATAAGCAGTGCTAAGCAACTCAGCTGTCTGAATCGATCAATACGCAGAAGTGTACGCAGCGGTTCCATTAAACTAAAACAAACTTATGAACACTTAATGGAAAATGGAGACATAGGGGCCATGAATAGAATGAGTTCATCAAGAAATAGACCTCACGATCTAGACATTAAATTATTACAAGCTAAGAGGCAGCAAGCTGAAATGAGAAGAAACCAACGAGAAGAAGATCTGAGGGAAAACCATCCTCTGTTCGACACTCCTTTGTTTATGGTGCCTAGAGAAAGTAAATTCAGAAAAGCGTGTCAGATGATTGTCTACGCAAGGTATGATGCTCGATTAAGAGATCCACTCACTGGCAAAGAAAGAAAAGTAAAATACAAAAGAATGCACAATTTCTTGAGTTTGGTTACATACCTTGATTGGGTAATGATTAACGTTACTACACTTTCATGCATTTCCATGATGTTTGAAACACCTACATACAGAGTCATGTCGACACCAGTTCTACAAATTGCAGAATATTTCTTTGTCATATTTATGAGCATTGAATTAACCTTGAAAATATTAGCAGATGGTATATTTTTTACACCTAAAGCCTATATGAAAGATGTAGCATCCATCCTGGACGTTTTTATTTATGTGACCAGTTTAGTATTTCTGTGTTGGATGCCAGAAAATGTACCTCCCAATTCTGGTGCGCAGCTTCTAATGATCTTACGCTGTGTAAGACCCTTAAGGATATTCACATTGGTACCGCACATGCGTAAAGTAGTAGACGAATTGTGCCGTGGTTTCAAAGAAATTTTATTGGTATCAATATTGTTGATAGTGCTTATGTTTGTATTTGCAAGTTATGGTGTCCAATTGTTTGGCGGCCGTTTAGCTCGATGTAATGATCCAAAAATCACAAGAAGAGAGGATTGTGTGGGAACATTTGAACGTAAAGTTTTCGTCACAAAAATGAAACTTCTCCCAGGACCTGGAGAAGATTACCCATCAATGGTTGTACCTAGAGTGTGGGCCAATCCAAGGAGATTCAATTTTGATAACATAGGCTATGCTATGCTGGCACTATTTGAAGTACTATCTTTCAAAGGTTGGCTTGATGTAAGGGATATTCTCATCAAAGCCCTTGGTCCTATACATGCTATTTATATCCATATTTACATATTTCTCGGTTGTATGATTGGATTGACTTTATTTGTGGGAGTGGTTATTGCTAATTATTCCGAGAACAAAGGCACTGCGTTGTTGACTGTTGATCAGAGAAGATGGTGTGATTTGAAGAAAAGATTGAAAATTGCCCAGCCATTGCATTTACCACCAAGACCAGATGGTAAACGTTTCAGAGCGTTCATCTATGACATTACACAAAATATATATTTCAAGAGGTTTATAGCAGGAATGGTTCTGATAAACAGTAGTCTTCTTTGTGTTTCTTGGAGAGAGGAAGAGTTTCATACTGGACCATTAGCTCTTGTAAGTACGATACTTACCTTGGTATTCGTAGTTGAAGTTGTGATGAAAATCATCGCGTTCACTCCCAGAGGATATTGGCAATCAAGAAGAAACAGATACGATCTTTTAGTAACAGTATTGGGAGTTAATTGGATATTTATTAATTCTACTTTTCATAACAACTTATCTTACACAATTGGTTACATTGTAGTTATATTCAGATTTTTTACTATTACTGGAAAACATGCTACATTAAAAATGTTAATGTTAACAGTGGGTGTATCCGTCTGTAAAAGTTTTTTTATCATATTTGGAATGTTTTTGCTAGTATTCTTCTACGCCTTAGCTGGTACCATTCTTTTTGGTACGGTTAAATATGGCGAAGGTATTGGCAGGCGAGCTAATTTTGAAACTCCAGTGACTGGTGTAGCCATGTTGTTTCGTATAGTTACTGGTGAGGACTGGAATAAAATAATGCATGACTGTATGATACAACCGCCGCATTGTACGCCTGCGGACAATTATTGGGAAACTGATTGTGGCAATACTGTAGCATCATTAATTTATTTCTGTTCTTTCTACGTTATAATTACTTACATTGTGCTAAATTTGCTTGTAGCTATTATTATGGAAAATTTTTCATTATTCTATTCAAATGAAGAGGATGCCTTACTTTCTTATGCAGATATCAGAAATTTTCAGAACACTTGGAATGTAGTTGATATCCATCAACGAGGTGTTATACCAGTCAGAAGGGTGAGATTCATACTAAGATTGCTGCGGGGACGTTTGGAAGTTGACCCACAGAAGGATAGACTTCTATTTAAACATATGTGCTACGAATTGGAAAGGCTTCATAATGGTGAAGATGTCACTTTTCATGACGTTCTGAATATGCTTTCTTACCGATCTGTAGATATAAGAAAAGCCTTACAGTTAGAAGAATTATTGGCTAGAGAGGAGTTCGAATATATTATTGAAGAGGAAGTAGCCAAACAGACGATTAGAACTTGGTTGGAAGGGTGTCTAAAAAAGATTAGAGCTACCAGCGGTAAGCAACAAAACAGTTTGATAGCAGGATTGCGAGCCACAAATGAATTTAATGTACAACAGGATTTACAGGAGGATAAAACAAAGGAAAACAATGTGGAAAGGGAAGCGGAAACAGAACCAAAGGAGCAAGAAGGTTTAAGACAGAGAACAAAACCAAAACCAACTTCCGCAGCAATACCTAGATCTGACAGCGTCGGCAGCGCTTCTGGTAGAAAATATCTTGCGCCCACTTTGTCTGATCCATCCACAAGACCGGATAAGGAAAGAAATAATAATAAAAAAAGAGCAAACAGAATATCCGAAAGTTTGGAGATGTCCAGAACAATGGCCCAACACAGAGAGACGGTGAATAACAAGATAAACATTACCAAAAATACAAACATTACGTTGGAAGTACGTGACTGGTGGGATGAACAATTGGTACATAATTCTGATTCAAGTGACGATGAAACCGGCGGACTCTAA

>SNMP1A

ATGGCAGCCCCTTTCAGACTTGGAGTAGCTGGAGTTGCTATTTTCCTTGTTTCTGCGATTTTTGGTTTTTGGGGATTTGAAAAATTTCTCTATTACAAAATTGGTGAAAATGTAGCCCTGAGGAAAGGTGGTGATATGAGGAAAGCATGGTCCAAATTTCCAATAGCCGTAGAATTTAGAATATTTATATTCAACGTAACAAATCCACTTGAAGTACACCAAGGGGCCAAACCTAAAGTTCAAGAGATTGGCCCGTATTTTTTCGATGAATGGAAAGAGAAAGCTCTATTTGAGGATGACGCTAAGGAGGACACAGTTGCTTTTAATCAGAAAACAACATGGATTTTTCAGAAAAGTCAATCTGGAGGACTGACTGGCGAAGAAATGATCACCATTCCACATGCAGCCTTATTGGGAATGGTGCTCATGGTGGAACAGCAAAAACCAGGAGCGCTTCCCATGATTGCTAAAGCCTTGCCAGCTCTTTTCAACGGTCCAGAGACTGTTTTCTTGACGGCCAAAGCTATGGATATATTATTTGACGGCGTGCCAATTAATTGTACCTCCAAAGAGTTTGGACCGAAAGCAATTTGCACAATGATCAGACAGAATCCAAAAGGTCTGAAAAAACAAGGAGACGATATCTTCTTATTCTCCTTTTTTGGAACAAAAAATGGCACAATCGATGACGGGAGGTTCAAGGTGAAAAGAGGTATAAACAATCCCAAAGAGGTTGGGATGATGTTCAGTTTTAATGGGAAAACAGTACAAGAAGTGTGGTCCGGTCCAGAATGTAACGCTCTCAGAGGCACAGATTCAACTATCTTCCCGCCGTTTATCACACCTGACGAAGAGATAGTTTCCTTTGCACCTGATTTGTGCAGATCTCTGGGAGCCAAATTTCAGTACCATATAAATTACCGAGGAATTCCTGGAAACCATTACACTGCAACACTGGGAGATATGTCTGCCAATGAAGACGAAAAATGTTTCTGTCCCACTCCGAGTACATGTCTGAAAAAAGGAGCGTTTGACATTACTAAATGTGTTGGAGCTCCTATAATATTAACCTTGCCACATTTCTACGAAGCAGATCCAAGTTATTTAGAAGAAGTTGATGGACTTCATCCAAACAAGGAAAATCATCAAATTTTTTTGAATTTTGAGCCGATTACTGGTACTCCATTGGGTGCTAGGAAGAGGTTGCAGTTCAATATGAGAATCCACGCTATTAAGAAAGTACCGTTAATGAAGAACTTACCAGATGCTATGATACCGTTGTTCTGGGTAGAAGAGGGATTGGAGTTATCTCAAGAATTCATAGACCTGCTGGATGCCAGCCTGTTTCGCTCCATGAGGATAGTAGGCGTAAGCAAATGGGTGCTGATGCTTGTCGGACTGGGCATGATTGCTGGTGGATATCTTCTACACTACAATCGCACCAAAGGACTGGGTGACAAACCGCCAGTCAACGAGAAGCCCAAATCCATTCAAGTTGCATCGGTTACGCCGCCAAAATTTTAA

>SNMP1B-FX

ATGAGCTCCCCAAAGAAGGATTTTACAAATCTAATGGAGAAGATCAAGCAGATGCCAACTAAGGTGAAACAGGCACCGCCTCAAAAATTTGGCAAATGGGGAGCCGGCCTCGTGCTTGGGGGCATTGGATTTGGATGGGTGGCTTTCCCCTACATTTTGTCATTCGCCATTTCAAAGATGGTAAACCTACAAGAAGGAAATGATATAAGAAATGTGTGGAAAGCGATACCATATCTATTCAATTTCAATATTTACGTATTCAACATAACAAATCCAATGGAAGTGCAAAACGGAGCCACCCCTATTGTGAAAGAAATAGGGCCGTATCGATACAAAGAATTTAAGGAGAAAGTGGATCTGGTGGACGATGTTGACGAGGGAACCATCACCTACAGCAATATGAATACCTGGTACTTTCAGAAAGAAAAAACTTTACCCTTGACTGGTGATGAAGTTGTCACAATTCCACATTTACCACTTTGGAGTATGCTGTTAGTGGCAGAATCAGATTTTCCATCACCTTTGCTGAGTGTGGTCAATGCAGGTTTACCTAAAATATATGGTAAACTGAAAACCGTATTTATGACCGCTACAGTAAATGAATTATTATTTAAAGGTGTACTAATTGATTGTAGCGTTAAAGATTTTCTACCCAAAATTTTGTGCGTGGCCATTAAACAAAACAGTAAACCGCTACAGAAACTCGGAAACAACCGATATCTATTTTCGGTTTTAGGACCTAGGAATGCTACACCCGAAAGCTCCAGAGTAACCGTTAAGAATGGTGTAGGAGACGCATACAGTATTGGCAAAGTTATAAGGGTGAATGGTAAATCGGCAAATTCAATGTGGCTGGAGGGAGATTGTAACAGATTTGCTGGAACGGACGCTACTATATTCCCACCATTCAGGAGGCCTGATAATAACAGCGTAGTGGCATATTCAACTGATGTATGTAGAAGTATATTTGGAACATATGAAGGAGATGGAGAGTACAGAGGAATTCAAGGGCACAAATATGTTTTTTCTTTGGGTGACACTCGAAAGAATCGCAAAGATTCATGTTACTGTCTTAGAGATGACGCTTGTCCTAAGAAAGGAGCCATTGACTTGATGAAATGCCAAGGTGCCCCATTGGTTGGAACGTTTCCTCATTTTTATGACAGTGATGAAAGTTTTTTGAGAGGAGTTATCGGCTTAAAGCCCATCAAGGAGAAACATGAATTATCTTTCCTAATGGAGCCGACAAGTGCTACACCACTGGTAGCAAGAAAACGTTTCCAATTCAATCTGCCCTTACATCCAATTCGTTACGTCAATGTGACTAGAAAAATTAAACCAACTCTGATGCCAATTCTATGGCTTGAAGAGAACTTGGATCTCGATGGTGAACTGATGGATTTTTTAGAAGCAAATTTATTATCAAATCTACGATTGGCTAACGGTGTAAAATGGACTCTTATAGTACTTGGAGTTAGCCTGTGTTTTGTAGGAGTGTTTATGCACCAGAAAAAGAAACTAAATGATCGACCAGTAATCAGAGTAACTCCTTCACCGAGTGGTTCTGTTCAGACTAATCTAAGCAAGAGTACCGCAGATTCCAGAGAACAACTTATTTCACCTTTTTCAAAAGGAGACAGTCCATTTCAGGCTGGTGTTGTTTTAGAACCATCTTTGTTAAAAGAAGGATCTGGAGGATATCTCAGTAGAAAATCTGGTAGCAGCGAAGATCCAGAAAACGTTAGCAGATTTGCTGGAAAACTGTACGAACGATTAAGTCCGCCTTCAACTAGCCCTCCCGTTGAAACGATATCTCGTCGACTACAACATGAACCGCAAATTATAATGTCGCCTGTACCTAGTGATCTACCGCCAATAGAGACTCCTTTGTTACAACAAAGGAAAACTCCTCCTCAGCCGTCTGCCGAAGAAAGTGGCGTATTCAGAATCCGCGGCACTGAAGAATCCACAGTGATACCTATAAAAAGTACTCATGGTACTGCAGAGTCTAGAAAATCTGAGCGAAGAAAAGCCGAGTCTAGATTATCAGAACACAGTGAAGCAGAGTCTTTACCAAAAGAAAAACAAGATATTCCACATTAA

>SNMP1C-FX

AGCATGCTGTTAGTGGCGGAAGGAGGATTTCCACCACCTTTTCTGAGCTTTATCAGTGCGGCTGTACCTAAAATTTATGGAAAACTGAAAACAGTGTTTATGACCGTTAAAAAGAATGCAACACCTGAAAACAACAGAATAACTGTTAAAAATGGTGTAGGAGATGCTTCCACCATCGGCGAAGTGGTTAGGATAAATGGTAAAACAGAAAACACAATGTGGCTGGACGGCGAATGTAATAGATTTGCTGGCACGGATGCCTCTATATTTCCACCCTACCGGACACCTGATAACAATAGTATAGTGGCGTATTCTACAGATATATGTAGCCATGATGGATCAATAATAATATCTAGAGACCCTTGCTCATATCTGATAAGTCATTCTTTGTTTTTCTGTTGGTTGAAGTCATCAAGTTTTAGTAACAGCAAAAAAAGTGAATCATATCACTTTGGAACTCATGTTGTAAATTCATTAAGAGTGCAAATCATTCTAAAAAAAACTCCGAAATTTATCAGTAGATTACACTCTAGTGAACAGCTAGAACGCTTTAAAAGAATCATATATTGTATCGAAGAGTTGTTCGAAGCTGACAATATTAGTGCTGCACCGTTGATAGCGAGAAAGCGTTTCCAGTTCAATATACCTATACATCCAGTGCAATATGTCGGCGTTACTAAAAAACTAAAATCAACCATGATGCCAATTGCATGGGTTGAAGAGTCGTTGGATCTGGATGGAGAACTGATGGACCATCTGGAAGCCAACTTATTATCAAATCTACGAATGGTTAATTTCATAAAGTGGACCCTTATACTGCTTGGAGCCGGCCTCTGTTCGTTTGGAATGTTTATGCACAAGAAAAAGAAGCAAAGGGAACCTTCAGTAATAAGAGTTACACCCACGCCCAGTGGATCTTTTGAGTCTAATCTAAGCGGGAGTACCGTTACTTCAAAAGAACAACTTATTTCACCTTTATCAAAAAGAGGCAAACCTTTGCGGGCTGGTATAATTTTAGAACCATCTGTATTAAAAGAAATTTCTGGAGGGTACGCCATTAAAAAATCTCATGCTAGGAATGATCCAGAATATGTTAGCAAATTTGCAGGGAAACTACAGGAACGATTAAACTATCCTTCTTCTAGTGCACCACTTGAATCAATATCTCATCGTTTACAGCAACAACCACAGATCATTATGACGTCTGTTCCGCCAATACAAACTCCTGAGATAAGGGAGGCGCGACTGGTTGGTAAAAGAGAATTACTCCAAATCCATAGTACCGAAGGGTTACCAATGAGATCAATGCGCAATCACAGAAAATCAGAACTTAGAAGCTCTACGTAG

>SNMP1D-FX

ATGTCTGGCAATTCTGCTCCGACAAGCACATATATAGTTAAGGATTTATTATTTGATGGTTTCTTAATTACTTGCAACAAAAGTACTAATGCCTCTTCATTAGCTAACTTGTTTTGCTCATCGCTCAAGCATTTTCCTAATATTAGAATATTAAGCGAAACTGAGGATGGCAATCTTCTTGGAGCACTCTTTCGTTATAAAAATACTTCTTATGATGGCCTCTTTGAGGTAAATAGAGGAGTAAAAGAATTTGCATCCATTGGTAAAATTGTAAGTGTGGATCACAAGACAGCACTAAACAATTGGAAGGGAGATTTTTGCAATAAATTTGCAGGAAGTTACGATTTTACCTTACTTCCTCCATTTGTTACTAAGGATTCAAGCTTCAAGATATTTAGCACTGATATCTGCTCATCTGCACCAGTGGTATATTCTGGTAAAGCGGAATATCGTGGAATAAGTGGTTTAAGATTTAAAATAGCTCCAGAATTCATGGGTAATACGACTAAATATCCTCAAAATTCTTGCTATTGTTCTGGTTCTATTGGAAATCTTACTAGTTCTGGAGAATGTCCGCCGTTGGGCACTTTGAATCTTGCTCCCTGTATGAATATTCCTGCTTTGGTATCATATCCCCACTTACTTCATACAAGTCCACTCTACCAATCTCTTGTGGATGGTTTAAAACCAAATAAGACGTTGCATGAAACTTTTTTTGATATTGAACCAACCACTGGAGTTCCTCTAAAGGGATATAAACGATTCCAGCTAAATGTACTAGCAAAACCCTCTTCAGCCATTCGGGTTTTAAAAGATACAAAATTAGCCTTGTTTCCAATATTATGGATGGAAGAAGGCGCAGAAATAGGTGAAGATCAAGTCAATATTCTTCGTGATCAATTACTCAAAGTATTGCACATTGCAGAGATAATAAAATGGGTTTTGATATCTTGCGGTATTAGCATGGCTATAATTGGAGCAATTATAGCAATCTGGTTGGTCAGAAGAAGAGTTCACCAACATCCTGATTAA

>SNMP2-FX

ATGGCTCATCTGTGTGTACATAAAGTGTCCTTGGCTTCCGGCGTTGGAACTGTGATTGTCATCCTAGGTATTGTACTTGGATGGTATGGATTTCCGTATGTGTTTAATAAAAATGTAGCCCAGCGCATCAGTCTATTTCCTGGCTCTGAAGCCATGCAGAGATGGATGGAAATACCATTTCCACTAGAGTTTAAAGTCTATTTCTTTAACGTAACCAATGCTGATCGTATACAAGAAGGCGAACGTCCTGAACTACAAGAAGTTGGACCATATGTTTTTGACCTTTATAAAACTAAAGTCAATGTTTCTTACAATAAAGTAAACGATACTTTGGACTACTACACACAGAATAAATACTTTTTTAATCATAAGAAATCTTTTCCACGTAAAGAGAAAGATATATTAGTATTAGTCAATGTGCCATTAATGGCAACCGCAACTATGGTTGAAAGGCTGTTTCCAGTGGGACTGAGTTTTCTCGATAAGGCTGTACCATATTTATTCCCAAATATAACTAATATGTTTATAACAGCATCGGCAAAAGAATTTATGTTTGACGGAGTATTGGTGAACTGCAGTTATGCTGTAGGACCTGCCATGCCAGTATGCAACGGTATGCGAGGCAAATTACCACAAACTGTTGTACCAGTACCAGACTCTAAGGACTTTAAGTTTTCCTTCTTTAAACATAAAAACGAAACATTAGAAGGACCATTTAAAATCTTTGGTGGAAATAAAGATATATTTAAAATGGGACAAATTATTGAATACAAGTCTAGTAACAATTTAATAATGTGGGAATCAAATAGTACGTGCAGTCAACTGAAAGGAACAGATTCTACAGTATTTCCGCCGATATCCGATCTAAACAATGATATATTCATTTACATTCCCGATATCTGTTTATCATTGGCAGCTATTTACAAAAACAAAACCATCATTAACGACATGACAATGTATCGATATGAGAGCAGTGATAAGAATTTCGCTGATGAAAATTCTGTCCCTGAAAATCAATGCAGATGTAGACAAAATGAAGAAGGCAGTGGACCTCCTGTATGTTTGAAGCAGGGTGCTATTGATGCCTCTAACTGTCAAGGTGCTCCTGTAATATTTACCCAACCTCATTTCCTGGACGCTGATCCTGAATACACACAGTATCCAGAAGGATTATCTCCTGACAGAGAGAAGCATTTAACCTTTGTTATACTTGAGCCGAAAACTGGAGTCCCTGTTATCGGCAGGAAAAGGATGCAAATGAACATATTTCTTCGTAAAATAGAAGACATAACACTACTAACTAATATTAGCGAAGGATTACTTCCAATTGCTTGGGTAGAAGAGGGTGGTGAGTTAAATGAAGAATCTCTCGAAAAATTAGAAGACATGTATTACATTTTACATTCATTGGACGTCATCAAATGGATAATCATAAGTATTGGCATACTAGTATTATTTGCCGCGATGATCTTGTACGCTAAATATCGTGGTTTCTTCTGTTTCAATGGTAATCAAGTGTCTGAATTCAGTTATGGCGTGAACACATTCCACGTAGAAGGTAAAATGAATCATGATCGTACAAAATCCTCAGTATTTGGCTTGCCAACCACTAACAGAAACGGAGGATTAGAAAGTGGTGATGATATCCAAACGATATCTAGTAATATAATACATCCTGATGGAAAATTAAATGAAAATCTATCGAATTCGGTGCTACCAAGT

>Group2a

ATGTCAGCTGGTGTGGTAATTTCAAAAGTTATATTATGGGGTCTTGGAGGAGTAATTCTAGTATTGTGTGGTACTATGAGTGGATTTTGGCCAGTTATATTGAATTATCAATTAAAAACTGAATTAGTATTAACTAATGATTCCAAAAGTTACAACCTATGGAAGGAAACGCCAATTCCAATGTACTTAGAAATATATATGTTCAATTGGACAAACCCTATAGAAACGTTGGAAAATAATGTAAAACCTAACTTTTTAGAATTAGGTCCATATGTGTTTAGTGAGCATCACATGAAAAAAAATCTAACATGGAACACAAATGATACGATAACGTTCAAGCAAAGAAGAAGATGGTATTTTATACCGGAAATGTCTAAAGGAACACTCAATGATGAAATTACCAATATAAATGTTGTTGCCATGACTATGCGAAATTTGGTAGTCAAATATGTGCCTAAAAAATGGTATCCAGTAGCGAACATCATATTAAAGGTAGAAGAGAAAAAGGTATTTGTAACTAAAACCGTAAGAGAATTACTTTTTGATGGTTATTCTGATGAGCTGTTGAATCTGACGAAAAAGCTGGAGAGAATTCTTAAAATCAAGATACCTTTTGATAAATTTGGTTGGTTTTATAATAGAAATGACTCTATTAATTACGATGGCGTTTTTAACATGAAGACCGGAGCAGGAGGTCTGGATAGTTTAGGAAATATAGATCGATGGAATTACAATTCTACAACAACCGCATATCCAGGACCTTGTGGCAAAGTGAAAGGTAGTTCTGGAGAATTATGGCCAATTAATACTGCTGCAAAACAGCATGCTGAGATCTTTGTTACTGATATATGCAGTTCACTGAAATTGAAAGAAGATGGTGAAGATGTAATCAATAATGTGAAAGGAAGCAGATTCACGGCGGACAGTTTGATTTTCGATAATGGAATTCAAGATCCAAACAACATGTGCTACTGTTCGAAACCTCCTTGTCCGGCAACAGGTGCCAGGGACATCTCGAGCTGTCGTTACGGAGCACCAGCATTCATATCTTTGCCACATTTCTACTTGGCTGATCCATCCTATACGGCCAACATTAGTGGCATGTTTCCTTCGCCTGAATTGCACAACTTCCATATACTGTTAGAGAAGGTAACTAGCATTCCTTTAGAAGTGCACGCAAGGTTGCAAATTAGCATCCGCTCAGAGCCTGTTCCCGGTATCAAATTTTATGAAAGGATTCCGACCGCTTACATCCCGATGATCTGGTTTGATGAAAAAGCTGCAGTTACACAATCTTTAGCCTCTGAACTGTCTACCTTAGTAGCTGTAGCCACCTACATTGAACCACTTCTCATTGGACTGGCAGTACTAGGAGCGGTAATGATAATCGCAGGGTTGATACTATGGCGTTACCACTTCTTTACCAAAAGAGATACACCCCTTCTGGAATCTACTTCATCCTAA

>Group2b-a

ATGGTTTCAATTCAACAACAGGAATTAAAATTATCACCAGGCACTAAAAGTTTTCAACACTGGGAGAAAACTCCAGTACCTATGTACATAGATATTTATTTCCACAATTGGACCAACGCTAACAAAGCGTACACGGAAAAGCCAAGGTTCAATCAAATGGGACCATATAGATTTTATGAGCAAAGAACTAAAGTGAACCTGACTTGGAATGAGAATGGTACTGTGAGCTATAGACAGACACGCCACTGGTTTTTCGACCCGGAACACTCTAACGGTACCCTGGAGGATAAAGTTACTACTATCAATATGATCCCTGTGATAGTGGCCCACATCGCTAGGTTTCGCAGCAGAAGAGATTTGATGACTTTATCCTGGGCGTTCACCGGGTTAAACATACGTCTGGATGTTGAGAAAACTGTCAGTGAATTACTGTTCGATGGTTACCAAGATCGATTGCTCAGCATTCTACAATTCATACCTGGACTGACGAAAATGAAGATAACTGATCGTTTTGGATGGTTTTATAAGAGAAATGGTTCAGAAATAGTGGACGGACTGTTTAACATGGATACTGGCACAAAAGATCTGAATCAAGTGGGTAAGATTCGTTCATGGAATTATAAAAACAAGACTAACTATTTCCGTGGACAGTGTGGCGATGTGGCCGGCACTTCTGGCGAGGTGTTTCCGCCTTGGTTAACGTCTAAAGACCGGATTGGTCTGTTCAGCGGTGATTTGACCATAAATCTTGATTACGAAGAAGACAGATCAGTAAACGGGATACATGGACTGAAGTTCACCGGCGGCACAGATCTGGTGGATTCTGGTTTGAAAGATTCGCAAACAGCATGTTATAGAAATGGTGAACAAGCACCGCTTGGTTTGTTGAACATCACTGAATGTAGATACGGTGCTCCGATATTTGTATCGTATCCACATTTCTATCTGGCTGACAAAGAGGTGCAGACGCAAGTGGATGGCATGAACCCAAGAAAAGAGGAACACAGTTTTGATTTGACCGTAGAACCGACTTTTGGCGTACCATTGGATGTAAAAGGACGATTCCAGATCAATATTATGGTAGAGCCGGCAAAGGATATTCTCATCCTAAGAAATCTACCCAAACGATATTACATGCCAATCCTATGGTTTGATCAACGTGCTTCACTCACTGAACCACTGGCCAATGAAATTGGCTTTATGGTAACATTATACCATGTCTGTAATACCATAGCATTGGCTTTATTAGTGATTGGCATATTGAGCGCCACTGTTGGCATAACTATGGCATGTAGAGAA

>Group2b-b

ATGGCCATGCCGCATGATATCAATGTGAAGCTGTACATTTTCAGTGCAATCGGTGCATTATTCTGCATATTTGGTACAACTATATTCTTCACGTGGCCAGAAATTTTTACGAATATACTGCACAGTCAAATGCAAATCATCCAAGGTTCGCACACATTCGCACTCTGGCGGGACACTCCAGTACCGATGTACATGAACTTCTACTTTCACAACTGGACAAACCCGGATGAGCTGAAGCTCAATAAACCGAGATTTCAACAAATGGGTCCATACAGGTTCAATATAGAAGAGAAAAAGACGCATATTGTGATGAATCCAAATGGCACTGTTACTTTCAAAATTTCAAGAAAATGGTTTTTTGATAAGGAGTCCTCCAACGGAACGCTGGATGATATTGTGACGACATTGAATGCAGTTGCTATGAGTGCCGCAACCACTGCTAAAGATTGGTCCAGGCTGATGACTAGCCCCTTATCCATCGCCATGATACAAACCGGGCAAAAGATTTACATACAGAAGAAAGTGGGCGATCTATTGTTCTTTGGATATAAAGATCCGCTCTTAGATATAGCCGATTCCTTGCCAAAGTTCATCGGCGTTGATATTCCATTCGATAAGTTTGGATGGTTTTACAAGAGAAACGAATCTAGTGATTTCGAAGGTGTATTTAATATGAACACAGGTGTTAATGATATATACCAATTAGGAAGATTACATAATTGGAATTACTATAATTTTACCAATTTTTATGACGAGCCTTGCGATATGGTGAACGGATCGGCTGGTGAATTGTTTCCACCGAATCAAACTAAAGAAAAGGCAATAGAATTTTATTCGGCAGATTTGTGCAGATCACTAAAGTTGACCTTTAAAGAAGAAGAAGTTATCCACGGCATTACCGGATATAAATACACCGGAAATGAATATACTTTCGATAATGGAACGCTGGATCCGAGTAATGAATGTTTTTGCAATGGTGCCTGCAGTCCAGCTGGTGTACTGAATGTAACATCGTGCCGTTATGGTGCTCCAGGATTTGTTTCGTTTCCGCATTTTTATTTAGCCGATAAATTCTATCTGGATCAGGTGGAGGGTATGAGACCGGATGCTGCGAAGCATACTCTATTCATTACTTTGCAACCGGAAACCGGCATTCCCCTGGATGTAGCTGCAAGATTTCAAATCAACATTTTGCTATCCCCAATAGAAAAGTTTAGTTTATTTAAAAATGTTCCTACGATATATTTCCCGATGATATGGTTTGAACAACGTGCTACGATCACCAGCCGTTTAGCTTGGCAAGTGAAGTTCGCGTTGGCCGCACCTTTGATCGGACAGATTGTGTCATTTTTATTTATAGTCGGAGGAGTCGCAGTGATGGCTTACTATCTCTATCTGATTGTTCCATACAGCAGGAGATCCAAGGAGACGATAATCGATTTGAAAGAAGGACCAATAATAAAACCGTTGATTCATAGTGACAGACCGATACAGATCGCAACTATGCAAGAATTGTTACCAAAAACCATGAATGGAGTCAGAATTGATGAGAAACTAGAATGCTAA

>SCRB3-FX

ATGGGCTTGACGAAACAATATTTACGAGTCGGCCAGTCGGCCAAAAATCGTCTGTTCGGAATACCGCCAGTAAGGAGAGGACCGTCACTAAAACCAGATAGAACACCTTTACAGATGCTAGTCTCACAGCAAGGCAACATAAACCATGGTCGATTAGCCCTTGTGATGATAGGACTGCTTACACTAGCTTTGGGTATCATCATGTCTACAGTACCGTGGGTTGATTATATTATTTTAAAGCAATTGAAATTATGGAATGGCTCTTTAAGTTATTATTACTGGCGACAACCAGGCGTTGTCAGATTAACCAAAGTTTGGGTGTTCAACGTGACAAATCCAGATGCATTTCTAAACGGACAAAAAGCTAAATTACAAGAAGTTGGTCCCTTTGTTTACAAGGAAGACATGGAGAAGGTGAACATTAAGTTTCATGATAATGGCACAGTTAGCTTCCAACACAAAAAAATTCTACAGTTTCTTCCAGAAAGATCTTTACATCCAAGAAATACAAAATTAATAGTCCCTAACATTCCATTGCTGACATTGACTTCTAAGCTGGCTAATTTGAACGCGCTTATGAGGATTGGGGTATCAGTAGGTTTGAAATCATTCTATGGTATGTCAGTATTTAATACTCTTACACCTGATGAACTTATGTTTGGTTATGAAGAAAAATTAACTAAAATTGCCAGCACCATATATCCGCGAGAAAAAAGGCCTCCTTCAAAGATGGGACTATTAATAGGGAGAAACAGTTCTTTATTAAACGACGTAGAAACAATTTATACTGGTGAAAAAGGTATGGAGAATTTTGGATTACTAGATAAATTAAATGGACTGGATCATCTTCCTTATTGGAACAGCCTACCATGCAATAACATCAGAGCTTCTGAAGGTTCATTGTTTCCCCCAAGAGATTTGACAAAAGAAGATGTAGTCCATGTCTTTGACAAGGACTTATGTCGCACTTGGCCATTGAGATATCGTTGGAATGAAGTAAAAGATGGTATTACTGTTGGACGGTATACTCCTGACGATAATGCGTTCACTTACAGTGACCGTAACAGTAATAATAAATGTTTCTGCCCGGGAAGGCAAAAATGCCCACCTGATGGATTGCAAGATATCAGTCCCTGTCAGTTTGATGCACCAGTTTTCTTATCATTTCCTCACTTCTATGGGGTTAAGGATAAAGAAATTACCGAAGCCGTAGAAGGATTAAATCCTGATAAAGAGAAACATGAAACCTTCCTCAAAATTCAGCAGAAACTTGGTGTGCCATTAGAAGTACGAGTTAGAGTACAGCTTAATCTAAAAGTGACCCATTCTAATTATGGTATTTCAAGAAGATTTCCCAGCATAGTATTTCCAATTGTATGGGTTGAAGAAGGAGCTGAAGAATTACCAGATTATATAATCAGATGGATCTACCTGGCTACAACGCTATCATATTGGCTAGTTCCTATTTTCACATATGGAACTATAGTGCTTGGTGCTTTTTTCCTTGTGCTAGCTTTTATTGGTGCATACAGGAATGTTGTATTTACTCGTGAAAATATAGAACGTGGTAAAGAACAATTTAGAAGAGGTTCTAGTTTCTTAGTAAACGGACAACATCGTTTGTTAATTATAAGAGATTCTTATACACTCTTGCACAATCAACCAACTGTGGCAGAAACATCATTTGATCCTGACCCATAA

>SCRB5-FX

ATGAGATTTAACAATTTTTTCGGGATACAAAGAGGTGGTGAATATAGTGCTGTGAGACAAAGTGACAGAGTGTACGAACCAACCACAGAACCGAATCGTCAATCTTTAAGTCCAACTCAAGAAGTCTTCAATAAATTCTTAGGCGGCCGTCTTTCAAGAGGAAATTCCAAGTCTAAATGGGTATATACTACTACAGTCTGCGGCCTGTGTGCGATCATCCTAGGTTCCATAATTGTTGCTGTAGATCCTTATCAGATAGTATTTAAAATGAAAGTGATCTTCAGCGAAGGTAGTGAAACTTTTCTGATGTGGGAAAAACCGGAAGTAGATCTGTTGTTGAAAGTTTATCTCTTCAATATCACTAATAAGGATGCATTTCTCAAAGGAACTGAAAAATTAAAAGTGCAAGAAGTGGGACCTTATGTTTATAAGGAAAGTATGGCTCATGAAAATGTAACATTCAATGAAAATGGAACGATCACAGCGATCCCTGTACATCCGTTAACATGGGTGCCAGAATTGTCCAATGGCACAGAAGATGATATTTTAGTATTGCCAAACATTGCTCTATTAAGTTTTGCTCAAGTGATGTCCACATCTTCAATGCTAACCAGACTTGGAGTGAATGTGCTGATCAGACAGACGCAGACAAAGCCGTTAGTATCGATGACTGCTAAGGAATTTATGTTTGGCTACAAGAGTACTTTATTAACACTTGGAAACAAATTAATGCCATCTTGGATTAGTTTTGGCAAACTGGGACTTATAGATAGGATGTACGATTTCGATGGCGACATAAGCACTACATTTTCTGGTACCACAGATATCCATATGTCCGGTTTGTTAGACAATTATAACAGTCTGAACTATCTACCACAATGGGAAGCTCCGTGTAACAATGTGAGCGGCGCAAGTGATGGTACCAAATTTAATTCACTTATCAGTAAAGATCAAGACTTGCTGTTCTTTAGGAAATCACTTTGCCGTTCTATGAGTTTGGTTCGTGTTGGTGAAGAAGTAGTTAAAGGTATCCATGGTTATAAATATGAATTCAAAAACAATTCCATGGACAATGGATTTGTCGACCCGAATAATAAATGTTTCTGCCATAAAAAGTGTTTACCAAAAGGAATATTGGATGTGCATGAATGCTACTATGGTTTCCCCATCGCTTTATCATATCCTCATTTTTATGAAGTTGATGAAGAAGTACAAAATTCAATTGAAGGAATGACTCCAAATGAAGATTTGCATAAAACTTTTTTCATAATCAATGAGGAATCTGGTTTACCTTTAAATTTATCGGTTAGAATGCAAATCAATATGGCTTTTACAGACCTATCAACCATGGCCAATGTCCAGCGATTCAGTAATATGATTTTGCCTATGTTGTGGACAGATATATCTATGCCAGGTCTACCCGCAGGAATGAACACAAAATTCTACATCTACCTCACTCTTGGACCAATCTTACAAGTGTTGGCCACATATCTATTTCTAGTTGGAGGTGTAGCATTCATATTATTGTCGCTGGCAAGTGCCTTATTGATACCAAAGATCAATTTAATATCCAGCAATAGAGGAAATACAAACAACGGCAATGCTAACTTGAGAAATGCGCACAATGAAGATTGGGCGGTGGACATGTCGACCGGATTGGCTAGCAATAAGAAACGTCATCCGCCTTCATTGTCGAAAAAGAATAGCACATCCAAGGAGATGGACTTGTATTATTGCTCATTGTTGGCGGTACAAAATGAAGAATGTTGA

>SCRB6-FX

ATGGCGTTAATTAGTCTCATATCAGCGAGTAGGAATTTAAACACCTGGAGCCAACTAACCCTTTCGATTACCTTGAAAAGCGTCCTGAAATATTCTCATGCAATGATTAAAGTAAAAGCCCATGAATTTTTCTTTGGCTACGATGATAAACTGGTCAAAGTTTTCACCAATTTAGCGAAGATCCTGCAACAGCCTTTACCATTTGAGAAATTTGGAATTTTAGCGCAGAGGGCAGCCTCTACGGACGAAACCACAACGGTGAGAACGGGCACAAATGATGTTGGACGAATCGGCATAGTCACAGAGATCAATGGACTGGACAAGCTGGACGCCTGGAATGACGATGAATGTAATCGGATTGACGGCAGTGATGGGGCTTTCTTCCCAAGACATACCATCAATGAATCAGCAACCTTGTATCTGTTTCACAAAGATATGTGTCGGCGGGTGCCGTTCGTCTATGATAAGGAAGTTGATTTTCAAGAAGGGGTTATGGCAATGAGATTTCGTCTAGCACCAAACGCCTATAACACCACTGGTTCATGTTTTTGTACTGAATCTGGATGTGCTCCGGTTGGAGTCTTTGATATTTCTCCCTGCGCAATGGGTGTTCCTATAATGATGTCTTATCCACATTTCCTAAGAGGAGATCCATTGCTGAGTGAACCATTTGAAGGACTTCATCCAAACCCAGAGAAACACGAATTTTATATTGATGTTCAAAGGCTTTTAGGCTTTACATTGGGCACAGTATCCCGTTTGCAGCTAAATATCCAGATAAACAAGGCCAAGAATATGGTGCTACTTAAAGAATTTTCTGACAAATTAATACTTCCAATAGTGTGGCTACAAGTTTCTGCTGAGAAAATGCCACCCGACTTATTCAACATTATCTATCACGCCACCTTCACCGTCAGAAGAATGCAAGTAGCTTTAATGTGGGGTTTCTTAATACTGACAATAATTTTAGGTTTTCTAATTTACCGAACTAGTAGACAATTAGAATCTCATAAAGTTCAAGAAGATAATGAAAATAAAAATGATGTAGCAGCCAGTGAAGAAATGCTGCCAGTCAAAATTCTCAGAGTATCTTGA

>SCRB7-FX

ATGACAGAGAGACTTAAAATGATGAAAGGATTGCCAGCATATGATTGGTGGCAAAATCCACCTGATGAAGTACTGCTCAGAGCTCATGTATTTAATATAACTAATGGTGAAGAATTTATGAAAGGGAAAGCTGACAAACTTATTGTACAAGAAGTTGGGCCTTACATATTTAGAGAAAAACTGTATCATACGAACGTCATATTTAATGATAATGGGACACTTACTTATACAGCTACAAGAAAATTAATTTATCTACCAGAATTGAACACCCTCTCTTTAAATGATACAGTATTTGTTGCAAATTTAGCAATAATGGGGGCTGCATCATATTTGAGCAATGCACCGTATGTTACTAAAATTGCCTATAACTTATTTATCAGCAGATTTGGAACAAAACCATTTGTTCGGTTATCGGTAAACGATTATTTCTGGAATTTTACCGACCCATTATTAGAATTTGGCAGATCATTTGCACCATCTTTAGTTCCAGAAGATAATATGGGGATTTTGAACCAGATTTATAAAGATTTTACGGACGTTGTCACCGTGCACATGGGTGTAGAGAGTGGGCCTAGAAATTTTTTCAAAATTGACAAATACAATGGTGCAAACGGTCTACAGGCTTGGAGTAATGAAACTTGTGATTCGGTGCTAGGATCGTCGGAAGGTGTTTCTTATCACCAAAATGTGTTCAAAAATGATACCGTCAAATATTTACGGAAGACCATATGTAGGGCTCTCCCATTATACTATGGAGGCGATGTAGAAATGTTTGGGATGACTGGTTATAGATTCAATTTGCCTAACAACACCTTTTCAAGATCTGAAAATGAAAATGAAGAATGTTACAGTGATCCTAGTTATCCTTTGCTACCATCAGGGCTGAGTGATGTATCACCTTGCTATTACAATTTGCCAATTGCTTCATCGTTTCCCCATTTAATGTTCGCAGAACCGAAAGCGACAGATAAATTACAAGGTCTCACACCAGATTGGGACAGACATGGTTCAGCGGCAATAATAGAACCTAATACTGGGGTGCCTTTCACAGCATGGGCAAGGAGTCAATGTAATTTAATTATGCACAGTATGTCTGGATTTCCAAAATTAAAAAGATTTAGCAATACGGTTATTCCAATGTTTTGGTTAGAATATAAACAAATGGGTCTTCCAGGCTATATTTCAACATTGATGTACTTCAGCGTTGTTATACTGCCTTCAATACAACTTTATATTAGTTTAAGTTTGTTAATTATAGGTTTTATATTTTATTTAATATCGATGGTAAGATTGTGGAAATCAGTTTCTTATAAACGCGCAAAATATAGTTTAATAAAATTATTTAATTCTACTAGAATCGAAACAACAGAACATGTTTGA

>SCRB8a-FX

ATGGGCTCCATTCTGTTAGGCCAGCCAGCTGGTGGTGGGGGAGGGGCCAAGGACAAGACTGGCCTAGGGGTTTGGGCAAGACTCAAGGCAGGACTACTGCGCCTGGGACCCAGTAGGCGTCATCAGGCGCCCATTTTGCCTGAGGCCGCCTATCCGCTGAAAGTAATAATCGACTCAGAACTAAGCGGCGTAATTTACAGTGATATAGGTGCCAACTATGAACACCCAAGATACTTTGCGCCATCTTGGCCTTACAAAGGGATAACAATTGCAGCAATACTTACCTCGATAAGCCTGATTCTGACTACGCTGATGTGGCTCACAAATATCTATGGCAACGCTATTCTTAAGTTGGTCTCGATCCGCAATGATATCGACATGGTATTCGAGTTGTGGCGAGTGCCGCCCACACGGCCTACTCTGTCCGTTTATATCTTCAACTACACAAACCACAGAAAGGTGTTGGAAGGCAAAGAGAAGCCACATGTGCAAGAAGTTGGCCCTTATGTATTCAGGGAAAAAATGGAACGAATCAATGTCATTTTCAACCCAAATGGTACAGTATCGTTTCAAGAAAATCGCACCATTGAACCCGACATGGAAAGAACAAGTGGTAGTATGCTTGATAGGGTTATCGTGCCGAATGTACCGTTAATTACAGTCTTAAAAACTGTGAACTCCTTGTTTTATCTGGATCAACGAGTGTTGACACAAGTTTTAAACTCCGTCAATTCGCAACCATTTCAAAATTTGAGTGTGAACGAGTTTATATGGGGTTATGAGGATGCTTTCTTCAAGATTGTAAAAAAAATAGTCAATCTTTTAACTCAAGAGGATACTAAAGGATTTGGGTTTCTAGATAAGAGACGTGGCGTTCATCATGACGTGGTTACCATGCACACAGGTGAGTACGATCTAGAAAACATTGGACAGATAACGCGTTGGTCAGGACAGGACAGAATCGGGTGCTGGGGTGACACGAAGTGCGACCAGATGACAGGAAGTGATGGCAGTATTTTTCCAGCACGAGCCACCAAAGATGGCAGGCCTATGTACATATATTCGCACAGCATGTGTAGGCGTTTACCGTTACACTTCGCAAAAACCACTTTGAGTAAAGATGGATTCCCCGTCCAAGAGTACAATGTAGCGCATGATCTGTTTGATTTTAGTCATAATAATACGGAAAACCAATGTTACCAATACAAAGGTGCCTATCCACCATCGGGTGTCTTCAATACAGGGCCTTGTCAGAATGACAGGCCAATCTACGTTTCATTTCCACATTTTTGGAAAGCTGGTCATGAATTAAAGCAGAGTATCTCAGGTTTAATGCCAGAAGAGTCCAGACACAAGTCTTACTTCCAGATACATAAAAAATTAGGCATATGTCTGAAGGCGAAAACAAGATTTCAAATGAACATAATGCTTCACAGAGCCAAATTTGTGGATCAAACATCGAAGCTACAAAATGAAACTATTATATTGCCTATAGGATGGTTAGACTATGACTCCGGTAATTTGCCAGAACACTTTGTTAACGTACTTCATCATCTAACTTTTACTGCTGCCTACATTGAAGAGACACTGAAATGGATCTTAGTGCTGTTTACACTGTCATGTTTAGTTTACTTTGCTGTCAATGTTCGCCGCTTAATTGTTCAACCATCTTGA

>SCRB8b-FX

ATGGCGTTAATTAGTCTCATATCAGCGAGTAGGAATTTAAACACCTGGAGCCAACTAACCCTTTCGATTACCTTGAAAAGCGTCCTGAAATATTCTCATGCAATGATTAAAGTAAAAGCCCATGAATTTTTCTTTGGCTACGATGATAAACTGGTCAAAGTTTTCACCAATTTAGCGAAGATCCTGCAACAGCCTTTACCATTTGAGAAATTTGGAATTTTAGCGCAGAGGGCAGCCTCTACGGACGAAACCACAACGGTGAGAACGGGCACAAATGATGTTGGACGAATCGGCATAGTCACAGAGATCAATGGACTGGACAAGCTGGACGCCTGGAATGACGATGAATGTAATCGGATTGACGGCAGTGATGGGGCTTTCTTCCCAAGACATACCATCAATGAATCAGCAACCTTGTATCTGTTTCACAAAGATATGTGTCGGCGGGTGCCGTTCGTCTATGATAAGGAAGTTGATTTTCAAGAAGGGGTTATGGCAATGAGATTTCGTCTAGCACCAAACGCCTATAACACCACTGGTTCATGTTTTTGTACTGAATCTGGATGTGCTCCGGTTGGAGTCTTTGATATTTCTCCCTGCGCAATGGGTGTTCCTATAATGATGTCTTATCCACATTTCCTAAGAGGAGATCCATTGCTGAGTGAACCATTTGAAGGACTTCATCCAAACCCAGAGAAACACGAATTTTATATTGATGTTCAAAGGCTTTTAGGCTTTACATTGGGCACAGTATCCCGTTTGCAGCTAAATATCCAGATAAACAAGGCCAAGAATATGGTGCTACTTAAAGAATTTTCTGACAAATTAATACTTCCAATAGTGTGGCTACAAGTTTCTGCTGAGAAAATGCCACCCGACTTATTCAACATTATCTATCACGCCACCTTCACCGTCAGAAGAATGCAAGTAGCTTTAATGTGGGGTTTCTTAATACTGACAATAATTTTAGGTTTTCTAATTTACCGAACTAGTAGACAATTAGAATCTCATAAAGTTCAAGAAGATAATGAAAATAAAAATGATGTAGCAGCCAGTGAAGAAATGCTGCCAGTCAAAATTCTCAGAGTATCTTGA

>SCRB8c

AATATCCTTTCTAGATTTCTTTCTTCTTTATTTTTAGGATTTTACGTAACTATTATTTTCTTAATTGTCAGCTCTATTGGTTTAATACTAATTTGGGGAACTAATATTTATGATAATGCAATAGTGCAGTTTTTAACAATAAAAGATAATGGTGCATTTTTTAATATTTGGCGAGATCCAAAAATTAATTTAATCGTTCGAATCTATCCATTTAACTATACAAATATCGAAGCTGTTTTTGCTGGTAAGGAGAAACTGAGAGTACAAGAGATTGGTCCATACGTCTTCAGAGAAAACAATGTTAAATCAAATGTAAGATTCAGTGGAAAGGAATATATAACATTTTCTTACAACAGGACCCTAACATATCTAAGCAAACTATCCAAGGGTTCACTAAATGATACAATAATTACCCCCAATGTTATATCTATTTCAGCATTAGACAAGGTGTCGAAAGAAAATTTAAAAGAAATGGGATTACGCTTCATATTGGCATTATTTAAGATCAAGCCGTTCAGCACTGTTTCTTCCTATGAATTTATAATGGGATATGATGATAATTTCTCCTTGATGTTGAGAAAGATAAATACATTACTGAAAAAAGAAGTACATCCACCTTTTGGAATTTTAACGAAGAGAAAAGGATTAAGTGCAGACAATATAACGATTGGTACTGGAGTTCCAGAAAGTAGTCACATTGGCATAATTAAAAAATATAATGGAAAAGAAAAGCTAGATTTTTGGAACGATACCTCATGCAACAACATTACCGCATCAGATGGCTTTCTCTTTCCTCGAAGTTTTGCGTCAGATAGCGATGTAATATACATATATGCAAAAGACTTGTGCAGAAGGATACCATTGAAATTTCAGAAAGAATTCATTGATGTGGATGGTTACCCAGTGAGAAGATTTGTACTTCCTCAAAATGTATATTCCACTCCGGACATTAATCCAGAGAATTCCTGCTTTTGTTACGAATCTTGTCCACTTTCTGGCATTCACAACGTGTCGTCCTGTTTTTATGATGCTCCACTTATAGTATCTCAACCCCATTTTTTGTACGGGGACCCAATACTGTTTGAAAACATAAAAGGACTTGATCCACAGCCAGAAAAGCATTCATACTACATGGATTTACATAAAGATTTTGGTTTCCCATTACGAGGACACACTCGAGTGCAATTAGGCATTGTGGTTAAGAAAGGTTCACTTTTGTCACGTTTAAAGTCTTACAGCAATGAAACGGTTCTGCCAATTACATGGATGGATTTTGTAAGT

>SCRB8d-FX

ATGGCTCATCTGTGTGTACATAAAGTGTCCTTGGCTTCCGGCGTTGGAACTGTGATTGTCATCCTAGGTATTGTACTTGGATGGTATGGATTTCCGTATGTGTTTAATAAAAATGTAGCCCAGCGCATCAGTCTATTTCCTGGCTCTGAAGCCATGCAGAGATGGATGGAAATACCATTTCCACTAGAGTTTAAAGTCTATTTCTTTAACGTAACCAATGCTGATCGTATACAAGAAGGCGAACGTCCTGAACTACAAGAAGTTGGACCATATGTTTTTGACCTTTATAAAACTAAAGTGAATGTTTCTTACAATAAAGTAAACGATACTTTGGACTACTACACACAGAATAAATACTTTTTTAATCATAAGAAATCTTTTCCACGTAAAGAGAAAGATATATTAGTATTAGTCAATGTGCCATTAATGGCAACCGCAACTATGGTTGAAAGGCTGTTTCCAGTGGGACTGAGTTTTCTCGATAAGGCTGTACCATATTTATTCCCAAATATAACTAATATGTTTATAACAGCATCGGCAAAAGAATTTATGTTTGACGGAGTATTGGTGAACTGCAGTTATGCTGTAGGACCTGCCATGCCAGTATGCAACGGTATGCGAGGCAAATTACCACAAACTGTTGTACCAGTACCAGACTCTAAGGACTTTAAGTTTTCCTTCTTTAAACATAAAAACGAAACATTAGAAGGACCATTTAAAATCTTTGGTGGAAATAAAGATATATTTAAAATGGGACAAATTATTGAATACAAGTCTAGTAACAATTTAATAATGTGGGAATCAAATAGTACGTGCAGTCAACTGAAAGGAACAGATTCTACAGTATTTCCGCCGATATCCGATCTAAACAATGATATATTCATTTACATTCCCGATATCTGTTTATCATTGGCAGCTATTTACAAAAACAAAACCATCATTAACGACATGACAATGTATCGATATGAGAGCAGTGATAAGAATTTCGCTGATGAAAATTCTGTCCCTGAAAATCAATGCAGATGTAGACAAAATGAAGAAGGCAGTGGACCTCCTGTATGTTTGAAGCAGGGTGCTATTGATGCCTCTAACTGTCAAGGTGCTCCTGTAATATTTACCCAACCTCATTTCCTGGACGCTGATCCTGAATACACTCAGTATCCAGAAGGATTATCTCCTGACAGAGAGAAGCATTTAACCTTTGTTATACTTGAGCCGAAAACTGGAGTCCCTGTTATCGGCAGGAAAAGGATGCAAATGAACATATTTCTTCGTAAAATAGAAGACATAACACTACTAACTAATATTAGCGAAGGATTACTTCCAATTGCTTGGGTAGAAGAGGGTGGTGAGTTAAATGAAGAATCTCTCGAAAAATTAGAAGACATGTATTACATTTTACATTCATTGGACGTCATCAAATGGATAATCATAAGTATTGGCATACTAGTATTATTTGCCGCGATGATCTTGTACGCTAAATATCGTGGTTTCTTCTGTTTCAATGGTAATCAAGTGTCTGAATTCAGTTATGGCGTGAACACATTCCACGTAGAAGGTAAAATGAATCATGATCGTACAAAATCCTCAGTATTTGGCTTGCCAACCACTAACAGAAACGGAGGATTAGAAAGTGGTGATGATATCCAAACGATATCTAGTAATATAATACATCCTGATGGAAAATTAAATGAAAATCTATCGAATTCGGTGCTACCAAGTTGA

>SCRB8e-Fx

ATGCCTATCGAAGAGGAAGTGTCTAACCTACTGTGGACCAAGGATGCACTGTACCTCTGTCTGGACGAGGCCACCAAGAGTTCGGCTGACAGCAACTGCTCTACTGCACATGTAAAGCCGATCCATATTACAAAAAAAGGCAAGCGGATCAAAGCTGATGAGGATTACAGAGCTTCGAGTCCGGCGTTTCTCCGATATTTCCTTCTGAGACTGAAGAATGCCAAGAAACAGGAGAGTGTCTTCAGATGTTGCGGTTTCTATGTGACATCTGTATTCACCATTTTGAGCTTCATTGGATTGGCGATGTTGTTTGGGAGTGACTTGTTTGAAACTGGAATACAAAAGATTATTACCCTGTCCGAGAATGGGTCTATTTATAATTTATGGTTGGATCCAAAATTAGATATACTTGTACAACTTCATCCATTTAATTATACCAATATTGAGGCAGTGTTAGCTGGTAAAGAAAAGCCAAAAATTAAAGAACTCGGCCCCTATGTTTTCAGGCAGAGGATAACTAAAGCAAACGTGGAATTCCATGGCAGAGAGACATTAACCTACTCTGAGAACATTACCAACGAGTTCTTGAGTGATCTCACAAATGGTAATCTGGAAGATGACACCATTATCACGCCAAATGTAGTCATTTTTGCTGCCATAAAGCAAGCTAGGAAAATGAACATGCTGTCACAGTTCACTTTGTCCGCGGTCTTGTCCCGCTTAAAGACCAAACCGTTCATAAAGATATCCGCCCGTGAATTTCTTTATGGCTACGATGACGACTTATCTTCCATCCTAAGAAAGTTCGTCAATTTTGTAGACCACGAATCTCTGCCATCTTTTGGTTTTTTAGCATCAAGAGATGGTCTGATGTCCGATAGACTGACTATAGGAACTGGTGTACCAAATCTTAATAATCTGGGCATGATCCAGGAATACGATGGGTACAAAGACCTAGAAGAAGGTTATGGAGAAGACTGCGACAATGCCAGTTCTTCAGATGGTTTTCTTTTTCCGGCTGAATTGATAGAAACCAGTGAAACACTTCATATTTACAGGAAATTTGTTTGCAGAAGTATACCGATGGAATTTCACAAACAAATCTTTACTCAGCAAGGATTTCCTGCCAAGAGATATTTACTTCCACGGAATATTTTTACTTCTGGAGATATTAATCCTGCTAATAAATGCTACTGCGAAGAATCTTGCCTACCGTCAGGAGTTGTTAGCGTTGCACCTTGCTACTATGATGCGCCATTGGTGCTTTCGCAGCCACACTTTCTAAATGGTGACCAAATTTTACTGGACAACGTAATCGGACTTAAGCCGGATGCTGCAAAACATACTTCTTACATAGACATACATAAAGTGGGTGTTCCCGTGGGAGGCGCAGCACGAATACAGTTAAATGTAGTGGTAAAGAAATCTCGGTATGTATCCCATCTGAAAGGACTGACAGAAGGCATGGTACTTCCAATTGGCTGGGCCAATCTGCAAATTGCTAATCTGAGTGGGATGCCCGAGAAACTTGTCTACCACGTTTCTTTCACCGCACCTCTTATCAAGATTGGGCTGCAGCTTACATTTTCTGCTTTATTCCTAATGTTCCTAACTAGATTATTTGTATATATGTTTCTTAGGTTGTAA

>SCRB9-FX

ATGTTCGGTTTGGAATTGGCTTATTGTATTATAGAAGAGGAATACAAATCTATTGACAACCTTGACGACGAACTAAGGAAGACAACTAACGACGAGAGACTTAAAATGATGAAAGGATTGCCAGCATATGATTGGTGGCAAAATCCACCTGATGAAGTACTGCTCAGAGCTCATGTATTTAATATAACTAATGGTGAAGAATTTATGAAAGGGAAAGCTGACAAACTTATTGTACAAGAAGTTGGGCCTTACATATTTAGAGAAAAACTGTATCATACGAACGTCATATTTTAA

>Ammonium_Transporter1-FX

ATGGCTGTACCTGAACGAAACTCCACTCTAGTCATGGAGGATTTAAAGAAAGACATGGACGACTTTTTCTTACTAACCAACGCGATATTTGTATCATTTATGCAGGCTGGATTTGCATGTTTAGAATCAGGTGCAGTTAGAACGAAAAATTCATTAAATATTATTATGAAGAACCTCTTAGATTTACTTATTTGTGCAGTATTCTATTATATAACTGGATACACTTTAGCTTATGAAGATGGCAATTCGTTCCTAGGATATTCTCGATGGGCAGGTTATGGTTTTGAAGAAAGTAAATTAGCTTACTGGTTTTTTCAATTTATTTTTGCTGCTACTGCTGCAACTATTATTTCTGGAGCAGTTGCCGAAAGGTGTAATTTTGTAGCATATATTGTTTACAGTGGACTAATATCAGGAATAGTATATCCAATAGTAAGCCACTGGGCATGGCATCAAGATGGTTGGCTTCGGAACATGGGCTATTCAGACTTTGCTGGTTGTGGAGTTGTCCATGCTTTGGCCGGAGTATGTTCATTTGTAGCCGCTGCACTTCTTGGTCCGAGAATTGGAAGATTTTCAAATGGTAAACCTAAAGAAATTCCTGGACATTCCTTGCCGCTAGTTGGAGTCGGAGCCTTGCTGTTAATATCAGGCTTTTTAGCATTTAATGGAGGTTCTCTTGGACATATAACTCAACCAGGAGATAGTGAAACAGTTGCAAGAAGTATGTTTAGTACAATAGTTGGTGGAAGTGGAGGAGCTACTGTATCTTTAATTCTTGGAAGAGTTGGAATGTATGGACCAAGTCCGTGGCCATTTTCGCCAACTCTTAATGCTGCACTCGCTGGCATGGTTAGTGTATGTGGTGCACCAGAAGCATATGCAACTTGGGCTGCATTTCTTTGCGGAGCAATAGGCTGCCTTATATACTTTGCTCTACATCGTTTGGTACTGTTTTTAAAAGTTGATGATCCTTTGGATACAGTAGCTGTGCATTTTGGAGCAGGAGTATGGGGTGTCTTCGCAAGGGCATTACTTTCACAAGAAGGGCTTATTTATGCAGATCCTAACGCTTTAAATTTACTATTGCACAATGCAATTGGTGTCTTTGCAATCATATGTTGGTCAGCAGTAACCAGTATAATTCTTTTTGGAAGTTTAAAGTTACTTGGGCTGTTGAGAATATCGGAAGAAGAAGAGTTGAAAGGTCTTGATATAAGTAAGCATGGTGAAACATCGTACCCATCATCAGCTTGGGGTTGGTATAATGACACAGTGGAATTCTATCCCGAAAATGGAGGAACTTTCAAAATGAAGAGAGAATATGATAACCATGCATTTCAATTAGAAAAACGAAATCAAGAAATTTAA

>Ammonium_Transporter3-FX

ATGTCTTCAAATATAAGTGATTTTAATGAAACTGATTTCCAGAAAGAAAATGATATACCATCTATGTATAATATCACAATTGAGGATGCTAGCTGGATAATGACTTCGGCTTTTATTATTTTTACCATGCAAACAGGTTTTGGAATGCTTGAGTCTGGTTGTGTATCTACAAAAAACGAAATGAACATAATGATGAAGAATATAGCTGATATATTTCTTGGAGGTGTGACATATTGGGCAGTAGGATTTGGTTTCAGTTTTGGGACTTATTGGACGTGCCCTGTATTGGCTATAGGCGATTTCGCTTTGGACCCTACTCCAGAAGAAATGGGCCCAAAATTCACAGCATTTTTATTTCAATTATCTTTTGCAACAACTGCTACAACTATTGTTTCTGGTGCGATGGCTGAAAGATGTAACTTCAAAGCATATTGTGTATTTTCATTTATGAACACAGTGGTATATTGTATACCTGCAGGATGGGTTTGGGGTGACCACGGATTTCTGAAACAAATGGGAGCGGTGGATATCGCTGGAAGTGGACCTGTTCATTTAATTGGAGGAGTCTCTGCATTTGCTTCCGCTATGATGCTTGGACCAAGATTAGGCAGATATGAGAGTGGAAATTCGTCACTTCCTCTTGGTACTCCAGTCAATTGTGCGATGGGACTTTTTGTACTCTGGTGGGGATGGCTAGCTTTTAATAGTGGTAGTACCTACGGACTTTCCGGTTTAAAATGGGCTTACTCAGCAAAGGCTGCTGTAATGACTATGCTATCCTCATTTGGTGGAGGAATTGTAGGTTTGACGTACACATTAATAATAAATCGAGGCAATGCTAACATTCTAGACATAATAAATAGTGTACTGGGATCACTTGTGGCAGTAACAGCTGGCTGTTTTCTGTACAGTTCATGGCATGCTCTTTTAATAGGATGTATAGGCGGGCTTGTAACTTCCTTTACGACACCTTTATTTGACAAAATCAAAGTTGACGATCCAGTCGGAGCTTCAGCTGTCCATGGCATGTGTGGTATTTGGGGTGTAATAGCTGTTGGCTTATTTGCTGAAGATCCGTATCCACTAACTACCACAAATCGCAGGGCTGGACTCTTTATAGGTGGTGATTGGTATCTACTTGGTGTGCAAGCTCTGAGTGCATTATGTTTGGGTTCATACAGTTTTATTGTGACTGTTGTACTTTTATGGAGTATTAATAAAGTTATACCTATAAGAATGCTTGCTCATGAAGAATTACTTGGGGCAGATTTAGCTGAACATTTAATTAGGCATAGAGGTGTTGGCATATCAAGAGTTGTATCAGCCTTAAATTTAATTGATGAATTTAATGTTGAACTTGATAAAGTAAGGACTACTGGGACCAATCCTGGGCATGAACATTACTTGGATGAATATGTCAAGAAAAAATTACAGAAAAATAATTTAAATCCAACTGAAAAAGTTTTTACTTTGTTGAATAATATGAAATTTAATAACGAGCATCCACAATTAGCGTGGCTAGATTAA

>Ammonium_Transport_Rh50-FX

ATGACGTTGGGCAAGAGAACAGGAATCATTCTTATTATCTTCCAACTTGCTCTGATAGTTGCCTTCGCATTTGTTGCCACTTATGGTGAAGAAGCTGACTCCAAGAATATTTTACCAAACATTTCAACTAATATATATTATCCAATGTTTCAGGATGTTCAGGTAATGATACTGGTCGGCTTCGGCATGATAATGACGTTCCTGAAGCGATACAGCTATAGTGGTGCAGGACTGAACTTTCTCGTTTCTGCAATCACCTATCAGTATGGCTTGTTGCTGCAAAGAATGTTTGGACCATTGAGAATAGGCATGAAAAATTTAATAAATGCAGATTTAACTTCAGCCGCCATTCTAATATCAATGGGAGCAGTTCTTGGCAAGACTACACCTCTCCAAATGTTACTGATGGTACTTGTTGAGGTACCTATATGCATGGGTAATCAATACCTGTCCACATTTCTCCAGGTGGCTGATATAGGTGGTTCAGTATCTGCGCACACATTTGGAGCATATTTTGGTTTAGGGCTATCCAAAATTCTAGGTAAACCGAAAGATACTTCACTTGAAGGTCCATCTTATCAAACTGATTTATTTTCTATCATTGGTACATTATTTCTTTGGGTATTCTGGCCAAGCTTCAATGGGGCGCTCAGCTGGGAAGATGGACAACAGAGAGCAGTTATCAATACTTATCTTGCGCTCATCGCTTCCTGTGTTATCTCATTCGCTGTCTCTACACTTACTCATCATTCCAATAAATTTACCATGGTGCATATACAAAATGCAACTTTAGCTGGTGGAGTTGCCATAGGAACAGTGGCCGACTTAATGGTGATGCCATATGCCGCCCTACTAATTGGTGGACTGGCTGGCGCAGTTTCTACACTTGGCTACGTTTATTTACAGCCTTGGCTACTTCATCATTTGGATCTTCACGATACTTGTGGAGTAAACAATCTACATGGAATGCCATCATTAATAGCGGCTACTGTGGGAATTATTGCAGCATCCCAAGCCTCCTTAGAAATGTATGGACCAAAACTGTACTTACTATTTCCTGCTCGAGCACCATTCCCTTCTTCTAAAGAACTGAAAGACATCCAGGTAATAGAAGAAAAAGTAATACCAGGTGAGAACAGAAGTGCAAATAGACAAGCTCTCTATCAACTATTAGCGCTCCTATTCACCTTTACTATAGCAACAATTGCAGGAATCGTTACAGGAGGTCTACTGAAACTATTCTCTCCTTGGATTGGTAATGTTTCTGACGAAGACACTTTTGATGACAGATTTATGTGGTCTGTGTCTGATGAGGGTGATTTAGATGATACCAAAAAATTTAAATCTGAAACAAATATCCAACCACTCTCTAACCATAATCATCATCAAATTGGTATTGTTTAA

CYP450 CYP4 clade from Mesquita et al., 2016

>CYP4G

GTGCTGTTTGTCTGTGCCAGGATGATGGAGCAAGAGTCTATACAGAGTGCTTTCAGTGCTACTGGCATGTTGATGACGCTGCTTGTGCCAGTCATGCTGCTCTACTACCTCTACTACAGGAAACAGAGGAGCCGTCTTTACGAACTGGCAGACAGAATACAAGGACCTCCTGGGTATCCATTTATTGGCAATGCTCTGGAATTTATTGGCGGAGCTGATAAAGTGTTCAACAACGTTATGAGCCACAGTTATGTGTACGAGAACGTGATCAAGGTGTGGGTCGGGCCCAGACTGCTCATCTTCCTCACCAACCCACTCGACATTGAGTTGATACTAGGCAGTAATGTGCATATCGACAAGGCACCCGAGTACAGGTACTTCGAACCGTGGCTTGGCAACGGTCTACTCATCAGCACTGGTGCCCATTGGCGAGCTCATAGAAAATTGATCGCACCTACCTTTCATCTGAACGTGTTGAAGTCATTTATTGGGCTGTTCAACAGAAACAGCCAAGCGGTTGTGCAGAAGTTGAAGAATGAAGTGGGCAAGCAATTCGATTGTCACGATTATATGAGCGAGACGACTGTAGAGATCCTGTTAGAAACTGCAATGGGCGTTAATAAGAAAACACAAGAGAGCGGTTTTGAATACGCCATGGCCGTCATGAAAATGTGCGACATATTACATCTTAGACAGACGAAACTTTGGCTGAGATCTGATGTAGTATTCCATTTTACCAAGTATGGCAAGTTGCAGAAGAAATTACTTGATACTATTCACGGACTAACCAAGAAGGTGTTAAAGATACGAAAAGAGGAGTATGTCAAGAATGGTAATTCTCTGAGAGTAACCCAAGAACCAGATAATATCAAAGTAGATTCCAATGAAAATGAAAAACTGGCCAGCGATCAGAACTTTTCATTTGGGCATTCTAAAGGACTTAAGGATGATCTGGACGTGGATGACAACGATGTCGGTGAAAAGAAACGTTTAGCTTTCTTGGACTTGATGATTGACAGCGCTCAAAATGGAGTCGTACTTACTGATGAGGAAATACAGAACCAAGTAGACACAATCATGTTCGAGGGTCACGATACGACGGCTGCCGGGAGCAGTTTCTTTTTGTGCATGATGGCGGCAAGGCCGGACATTCAAGAGAAATGTATGCAGGAAATTGACAGCATATTTGGAGACTCAGACCGTCCGGTTACATTCCAAGACACCCTGGAGATGAAGTACCTAGAGAGGTGTATCATGGAAACATTGAGGCTATACCCACCTGTGCCCATCATAGCCAGGGAGCTGCAGAAGGAACTCAAACTAGCTTCTTGTGATGTAATCATTCCCGCAAAGTGCACTGTAATCATTGCTACCTACAAGCTGCACCGAAGAGAAGACATATATCCGAATGCGGAACATTTCGACCCGGACAACTTCCTACCGGAGAGATCAGCAAATAGGCATTATTATTCATTTATTCCATTCAGCGCAGGGCCCAGGAGTTGTGTTGGTCGCAAATATGCAATGCTGAAATTGAAAGTACTGTTGGCCACTTTCTTGAGGAACTACAGAGTATTGCCTGGAAAACCGCAGAAAGACTGGGTATTACAGGCTGACATCATTTTGAAGAGAACCGATGGTTTCCAAATCCAAGTTGAACCTCGCCGGCCTACAATTAAAACTGTTTAATTGTGTTTACTTCAATTCCAACTTTAGTTTTAGATTTAATCACAATATTTACTTGTGTAGGGATGTTGGGTGACTTAGAATTTTGTAAATAATAATATATGTATGTATGTTTGTATATTTATTGTTTAAAAAACTTAGTTTTTAGCAAGAGTTCGTTAAATGTTAAAAGATTTGTAATGGAAAAATATATAAATATTTAATTGTAGATAGAA

>CYP4P

ATGAAGGCTACAGAGAAGAGAAACCTAGTTAAAGCATATAGTTATTGTAGAGTGTTAAAAATTGTGAAGAATATACCAACGCTTCCATCAATACCATTTTTAGGTGTTGCCTTACAATTCTACAAAATTAAGTCACCAAATGGTATGATAGAGGTATTGTCACCTTGTCTACAATCATCACCTCAGAAAATTATAAAATGTTGGATAGGACCAAAAATAGTTGTGTGCTTACTGAATCCAAAATATATAGAGGTACCTATTGTTTTATCAAGTAAAGATGCTTTGGATAGAGATTCTGTTTATAAAATTTTGCACATTTATGGATCAGGACTTTTCACTGAAAATGGTAAAAAATGGAAAGAATTGCGTAAACCATTGCACAAGGTATTAAGTTCAAAAGCAGTCGAATCATATTTCCATTTTTTTCAAGAATCAGCCGTAAAATTTTGTAAATTTTTACAACAGTACGCTGTTACTGGGGAAAAATTAGATTTGAAGCATGGAATAACAAATTTTAGTGTTGATTTATTATCAGCATGTACTTTAGGATATGAAACCAATGAAATGTTTAATTCAAAATTAACAGTTGGCAAATGTGCTAGTGAAATTTTATATTCAGCTACAAAAATGATGCATACACCGCATTATATTGTGTTTAATTCGTTGATTCCTTATGGAGCCGATGGACGCAATTTAATCAAATCCTCTAAAGAGATGTGGTCTTTTATTTTGAAGGGTAGAATGAACGAAATTGTTTCGCCAGAAGAAAATGACATCACAAATCCCCAATTTTACTCGGACTTTTTAATACAAAAAGGAAAAGAATATGGTTTATCACATGAGGAATTAGCACGCCTAGCAACAGATTATGTAATGGCAGGATTCGACGCGCCTGCTGTTATGAGTGCAGCTACTCTGATGATGTTAGCCATGAATCCTGAACATGAAGAAGCTGTTTATCGTGAACAAGTTGAAATCCTTGGCGATGATTTATCAGTGTTGCCTTCAGAAAATGATTTATCAAAAATGCATTATTTGAACAGAGTCATAAGGAAACTTTACGTATTTTTAGTCCAGTACTATTTAGGACTACCAAGAATGATATTATTCTTGATGGTAAATAATATTCTAATAGCTTCTGTTGTCTATGTTTGTGGAGAACTGAGCAGTATTGTGAGAGAGTATATCAATCAATTTATTAGCATTTCAACTAAGTCCGGAGTCTATTTAAGGTTAAAGTTTGATAATGACGTAACAATTGAAATTTTTCAAGTAGATGATAGAATTAGTACGAATTATTTATATTTTATCATTATTATATTTTATGCAGAACAGTTCACATTGCCAAAAGGTTGTTCAGTATATATCTCGATACACGATTTACACAGAGATCCTAGCAATTGGTCACATCCACATGAATTCTATCCTGACCATTTTCTACCTAAAGAAGTCGCTAAAAGACCTAAAGGAGCGTACATTCCATTTAGCTGGGGCCCAAGATCATGTCCAGGTCCACAATTTGCCAACGTTGAGATGAGAGTACTTCTATCTCGAGTGATTAGAGAGTATAAATTTGAAACTGATCTAAAATTGGATAAATTAAGTTATAAATATTCTTTATTAATCGAACCTGCAGAAGGATTTATGATGAAAGTTGTATCAAGAAATCTGCAAGAAAAGAAGAAGTAATATAAAAAACTGTTTAAAACTTATGAGCGAATTTTTGAGTGTATTTTGTATGTACTAATAAGTGAACTTACTGTATATTAATTCAATTTTTCAAAGATGGAACTTGTGTCAAAAATATATTGTAAAAGCTAAGGGAATGAATATGTATATGCATTTTT

>CYP4GG

TTGATCGTAGGGATGTTGCTGATACTCTCATTGTTGGTGGCGACCGCCGCCCTTGTGTACTACGTGAACTGGAGGATGTCCAGGAGAAGACTGTACGAGCTTGCGGACAAGATACCAGGGGAGAATGGTCTGCCATTCATTGGCGTCATGTTGCGGTTTGCGGTCAGTCCTAACAGAATTCTAGAAACGCTGTATGAAATGAGTTTCCGTGAAGAGTATCAGGATTTAGGCAAGGCGTGGATGGGCCCCAGACTGTACATTGCGCTGATCAACCCGAAAGATATCGAAATAATTCTGAGCAGTTCAGTGCACTTGAAGAAAGCGCGGGACTATAGTTTCTTTGAGCCGTGGTTCGGCAACGGTCTGCTCATCAGCAATGGCGATACGTGGAAGTCGCACAGGAAAATGATCGCCCCGACATTTCATTTAAATGTGTTAAAGAGGTTCATGGACGAATTCAATAAAAATTCTAAATGCGTCATCGACAGAATGGCTAAAGAGAACGGCAAATCTTTCGACTGTCACGATTACTTGAGTGAATGCATGGTGGAGACTCTTCTGGAGACGGTTATGGGAGTGAAGCAGGCGAACCAGGGCCGCAACTGCCTCACATATGCCTTGTCAGTGATGGATATGTGCGACATTCTTCATTCACGTCAGACTCGACTGTGGCTGAGACCAGACTCCATCTTCAGATGTACCAACTTGGCAAAAGAATACGACAGAAACCTGGGCATCATCTTCAATCTGACAAATAAGGTGTTCAAGAAGAAGAAGGAGGACTATGACCAACACAAAGTGAGCGACTTCCAGGAAAAAGAAGAACACCTACTTCCGGACCTCAAAGAACTGGCTACCAATGATAAATTCTCTTATGGAAAATCTACTGGCATCAAGGATGATCTTGATGTTGATGAAAACGAAATTGGTGAAAAGAGAAGACTTCCTTTTTTGGAATCAATGATAGAGAGGGCTCAGAACGGCGATGGACTGACTGACCAAGAGATCAAGAACCAAGTGAATACCATCATGTTCGAGGGGCACGACACAACGGCAGCGGGCAGCAGCTTCTTCCTTTGCGTAATGGCGGCCAGGCAAGAGATCCAGGAGCGGGTGCTGGAAGAAATTGACGCCATTTTCGGAAATTCCGACCGAGCAGTCACTTTCCAAGACACCCTCGAGATGAAATACCTGGAAAGGTGTATCATGGAAACTCTGCGGCTCTTCCCTCCGGTGCCAGTGATCGGCCGGGAAATCGAGCAGGAAATTAAATTAGCTTCCAGGGATCTGTCCATACCAGCCGGTTGTTCTGTTCTAATTCCCACATTCAAGCTACATCGCAGGCCTGACATCTATCCCAATCCAGAGGAATTCGATCCCGACAGATTTTTGCCAGAGAACTCGTCGTCCAGACATTATTATGCTTACATCCCGTTCAGCGCCGGACCTAGAAGCTGTGTCGGTCGTAAATATGCTATGCTGAAACTTAAAATTTTATTGGCAAACATTTTAAGAAAATTCAAAGTGAAACCTGGCAAAGCAACCAAGGAGTGGAAACTTCAAGGCGATATCATCCTGAAAAGAACTGACGGATTTGAAATTTCATTGGAATGTAGGAATACAAGAAAGGTGCCAATTTAATGGGCACATTCAGTTTTCTTGAACCAAATTGTGGTCGTCAGCAGTTTGGCATTGCTTTTCCCTCTCAATCCAATTACTTCAATTGGCTGAACTGATGTGATTTAATTAATTGGAAAGTAATTATTTATTATTGAACATCTATCCTACTCTTATTGTCTATCTCATTTTGTGAAGTTACGAATATTAATTAACAAATGCTTAGTAAATATTCTGTTTCTAATGTTGTAATATAGTGATTATTTATTGT

>CPY4U

ATGATTCCATTGAAGGATATACGACACCGTAGCCCACCTATACCACGATGGGACGCTTACAATAAGTTTTTCGATCAATTGAACTATCTGAAAGTGCCACATTTTGTAGAACTAACATCACCTGTATTAAAGACTGCACCTGTCAAAATGTGCAAATCTTGGTTTGGTCCGAAAATAATAATAACAGTACTTGAGCCGAAGTACATTCAGACTATTCTATCAAGTAAAGACGCATTGGATAAAGATTCAATTTATAACATTTTACAAACTGGTGGTTCTGGCGTGTTTAACCAAAATGGTAAAACTTGGCATGAATTACGTAAACCTTTAGATAAATTTATGAATATAAAGTTGATTGAAAATTATTTCGACAATTACCAAAAATCTGCCGTTCAGATATGTGATAGTATAGGATATTATGCTAATACTGGGGAATACATAGATCTGAGACATTGGATGGGAAACTTTACTATGGACGTGTTATCAGGGGCCATTTTTGGATACGAAACTAAACTAGCTTTAAGTAAAGACTGTTCAATTGGAAAGAAGACAGAACAGTGTCAGCCATCATCAAAGAGGTTGATTAAACAATTTAGTGCAGAGAATTTGACTTCTTCTTGCGGCTCACACAATAAACTTGCTGACTCAGTGAGAGAGGTTTTCATTAACCTCATCAGTTTAGCAATGAAATTGATATTTAAGCCACATTTAATGGTCTGCCAAGCGTTGCTTCCTTTGTGTGAGGATGGACGGGAATACTTGAACGGCGCTAAAGAGTTCTGGTTGTTTGTAGGAGACATATTACGAGGAAGAATTAATCAGATTGGTTCTTCAGAAGACTTTAAACCAAAATTTTACTCCGACATACTAATCCAAAGGGCTGAGAAAGCTGGACTATCACACCAGGAATTGTCTCGTCTTGCCACTGACTTAATAATAGCAGGATTCGATGCTCCAGCGCTGATAACAGCATCTGTTATCATGATGTTAGCCATGTTTCCAGAACACGAAGAAGCAGTCTATCGTGAACAAATGGAAATTATAGGAGAAGATCTGTCCATCATGCCATCTTGGAATGATTTTTCTAAAATGCATTACCTGAACAGAGTTTTGAAAGAAACTATACGTATCTTCTGTCCAATAGGGATTGTGAGGTATCTGAAGAAGGATATTAAACTGGATGATAAGTTTACACTTCCCCAAGGTTGTTCAACATATTTATCATTTTACGATCTGCACAGAGATCCTAAGTATTGGTCACATCCACATGATTTCCATCCTGATCATTTTCTACCTGAAGAAGTAGCGAGAAGACCAAAAGGAACATATCTTCCATTTAGCTGGGGACCAAGAAGTTGTCCAGGTACACAATTTGCTAATGTTACAATCAAGGTAATTCTTTCTCGTCTTATTAGAGAGTTTAAATTTCTAACTGATTTGAAATTTGAGGAACTACGATTCAAATATGCTTTATTCTTGGAACCAGAAAATGGATTTTTAATGAAAATACAACGGAGGATACAGTCGTCAAACATGAGTTAATAAAAAATATTTGTTTTAAGCAATTTTTTTTTAGT

>CYP4E

ATGTTGCTGGAATTAGTGCTATTATTGTTGACGATTATTTCATTAATGGTCATCTTGCTGGTACCGCTGGCAGATATCAATCTAAGACCAAAGACTATTCGGAAAATGTCTAAACTTCCAGGCCCAAGATTGTTGCCACTTTTCATTGGATTTGCCTATAAGCTTGTAACACTTGCTGAAACTGAAATGTTGCAATATTTGACTTCTCTTCATCAAGATTTTCCTCGCATGGCTGCTATTTGGATTATTGGCATACCGGAAGTATTGCTATTCGATCCTGACGACTTGGAGGTAATTATGGGCAATATTCAGCATATCCAGAAAGGTTTGGAATATTATTTTCTGATTCCGTGGCTAAAAGAGGGACTTCTCTTAAGTAATGGTGAAAAATGGCATCAAAGGCGTAAACTTTTGACTCCGGCATTTCATTTTAAAATTTTGGAAGACAGTATGCAAAGCTTGAATAAACATTCCAGGCTATTATTACGCAATATGTTGAACAGAAATGGTGAACCTTTCTTTGCTGAAGAGATGATTATTCCATGTACCTTGGACATTATCTGTGAAACAGCTATGGGACATTCCTTAAACTCTCAAGAAAATAGTGGCAACACTGATTATCTGAAAGCAGTAAAAAGTGCTTGCAATTTAATGTTCCGAAGATGTGTAAAACTTATCTATGGTAATGAGTGGATATACGCATGCACTGTGGATGGAAGAAATTTCCACAAAGACCTAAATTATCTGCATACTTTCTCAGAAAATATTATTCGAAAAAGAAAAATGAATTATTTAGCTGAGAGTCAATATTCTGCAGAAAAATCAATTGAGGAAGAAAATTTCTTTAATAATAAAAAAAAGAAAGCTTTCCTAGATATTCTTATTGAAACTGATCGGAAAACTGGGAACACATTTTCCGATAAAGATATTAGAGAGGAAGTGGACACCTTTATGTTTGAGGGACATGACACTACTTCGACTTGTATACAGTTCGCTCTCTACTTACTTGGAAGAAATCCTCATGTACAGGAAAAAGCATACGAAGAATTGCATGAAATCTTTGGCGACAGTGACCGTGCAGCTACCAACAAAGATGTGCATGAAATGCATTACTTGGAAATGATAATTAAAGAGACTCTGAGGCTGTATCCAAGTGTACCGTATATCTCAAGATTGCTCACCCAAGATCTTCAGCTAAAAGACGACACAGTGATACCAAAAGGAGCAAATGTTTGTATCATTCCATTTATTTTGCACAGAGATTCCAAATATTTTCCAAATCCAGAAGTATTTGATCCAGAAAGATTTACTGTGGATAATTGTAAGAAAAGACATCCATACGCTTATATACCGTTCAGCGCTGGACCCAGGAATTGTATCGGACAAAAGTTTGCCATGATGGAGTTAAAAGTGGTACTTTCAACAATTTTGAGATTCGCCAAAATCGAAAGCGTCAATGAAATTGATGATACAAAATTAATTCCTTCAGCCTTATTACGATCTAAAGATCCAATTCAAATTAAGTTAATATCAAGAGACTGATCTTTTTTTTTTTTAATGAATTAAAATTGATTACTTTTCTAATTGTTAATTATTGTAAATAGGTATTGTAAAATTTTCTATTAATAATC

>CYP4D

ATGATTGTGGAGTTTCTATTACTTATTTTATTAATGGCTATTCTATTAATGGCCGCCGCATTACCACTTCATTTAATAGACTTAAGACCAAAGACGATCTGGAAAATGTCTAAACTTCCTGGACCAAGAGGTCTTCCATTTTTCATCGGATTAGCTATTCGATTAGCAAAACTACGTGATATAGATATATTAGCGTTTTTCCAGAATATTCATCAAGAATATGCTCGTCTTGTAGCGTTCTGGCTCGCTGGCATACCCACGGTGTGGCTATTTGATCCGGATGACTTGGAGGTAATTTTGGCAAGTTCCCAAGCCATTCAAAAAGGAAATGAGTATCACTGTTTGTTCCCTTGGATGCAAGATGGTCTTGTATTAAGTAAAGGTAAAAAATGGCATCAAAGGAGGAAACTTCTAACACCTGCTTTTCATTTCAAAATTTTGGAAAATAATATGCAAAGTTTAAACAAAAATGCTAGATGTCTTTTGCGAAATATGTTGAAGAAAGAAGGTAGACCGTTCGTTACTCAAGAACTGGTCGTACGCTGCACTTTGGACGTTATTAGTGAAACTGCTATGGGACATTCGTTGAATTTGCAGGAGCGTGAAGAAAACAATGATTACCTAAATGCTGTCGAAAGGGCATGCAGCTTAACGTTTGATAGAAGTAAAAACGTTTTTTATTCGAACGATTGGGTGTATTTTCTGACGTTAGACGGTAGAAAATTTTCCAAAAATTTAAAATATCTTCACAACTTTTCTGAAAACATTATTCAAAATAGAAAATTGAAATATTTACTTGAAAAAAAATATTCAGAAAATAACATTGAAGAGGATAACAACTTTGGAAAGGGAAAAAAAGCTTTCCTAGATATACTCATAGATTTGGAAGACAAATCTAAGGGCACACTTACAGATAAAGATATCAGAGAAGAGGTTGATAATTTTATGTTTGCAGGACATGACACAACTGCAAGCTGTATAATGTTCACCCTTTACTTGCTTGGGAGACACCCTCATGTACAGGAAAAAGCATTCGAAGAATTGTATGAAATCTTTGGCGAAAGTGACCGAGAGGTTAACAACAAAGATCTGCATGGAATGCATTATCTGGAAATGATAATCAAGGAGTCTATCAGGATTTATCCTCCTGCTCCTTATTTTTCAAGGAATCTTATCCAAGATTTAGTGCTAAAAGACAAAACAGTGTTACCGGAAGGAGCAAATGTTGGCATCTTTGCATTTATTATGCACAGAGATCCCAAATATTTTCCAAATCCAGAAGTATTTGATCCGGAAAGATTTTCTGCGGAAAATTGTAAGAAAAGACATCCATACGCTTATTTACCTTTCAGCGCTGGACCCAGGAATTGTATCGGGCAAAAGTTCGCCATGATGGAGTTAAAAGTGGTACTTTCAACAATTTTGAGATTCGCCAAAATCGAAAGCGTCAATGATGAAATTAGTGCTAGAAGTTTAACACCATTGATATTGCTAAAACCGTGTGATCCTATACGCATTAAAGTATTTTCAAGATTTTAAAAAATAATTTTATGTCAAGGAATAAAAGAAAATGTAACTTGAATACTTATACTCAAATTTCCTCATACTGTAATCACCTAAGTCCCAACCTATGCTTTTCCTTCAAACTCAGAATTCCTGTTAAGTCAAAATCACATAATACAGATTGTAAAACT

>CYP4A

ATGCTGGTTGTCATAATTATAATTTTGTTTATATCTGGATTAATGTTGTGGTCACAATGGAAACTTGATAAACGTTTTGTTGTGCTCGGAAATACTATTCCTGGTCCATCTGGAATTCCATTAGTTGGGAATTTGTTCAGTGTACCACACAGCGGACCAGCTGGACTGAAATATCGACGTGTTCTGGTGAAAAAGTACGGTCATATTAATCGATTATGGGTGGGAAATATGCTCGCAGTATGTTTGAGTGATGCTGAGGATTGTGAAATAGTTTTGAAAGATACAACGCATTTGACTAAATCAGTTATTTATAACTTGCTACACTCGTGGTTGGGAACAGGATTATTAACGAGCTCTGGAGAGAAATGGCACAAACGTCGCAAAGCAATAACCCCTGCCTTTCATTTCAAAATATTAGAAGAATTTATAGATATTTTCAACAAAAATGGAAACATTCTCATTGAATGCTTAAGAAATCATTGTGATGGCTCTACATTTAATGCCCAACCATTGATATCGAGATACGCATTAGATGTTATTTGTGAAACTGCAATGGGAACTGAAATTAATGCACAACTTCATCACAACTCTAATTATGTTCAAGCAGTAACAAAAATGTGCGAACTTATTTCTGAACGAATTAGAAAACCTTGGTTGCAGAACAATTTTATTTATTTTTTAACTGGAAAACACAAGCAAGAACAAAAACTAATCAAAATTCTTCACGATCAGACTAATAAAGTTATTAAAGAAAAAAGTCATCAAATGCGAGAAGATATTCACGTTGGATCGTTTAGAGATGAAGAAGGTGTTAAAAGAAAAGTAGCGTTTTTAGAATTACTGTTAAAGATGAAGATGTCAGGCAATTCAACGTTTCAATCAGATAATGATATTAGAGAAGAAGTGGATACTTTCTTGTTTGAGGGCCATGACACTACAACAGCTGCTATTGGCTTTGCGCTCATTCTGCTTTCTAGACATAGAGAAGTGCAGGAAAAAATATATGAGGAATTGGTAGAAGTAATGGATTTACAGGACTCACAGAAGATCTCTTATGAAAACTTACAATTGATGAAATATTTGGAATGTGTCATTAAGGAATCTCTTCGACTCTATCCAAGTGTTCCATTAATAGGAAGAGAAATTTTTGAAGACTTACATCTTCCTAGTGGTTACACCGTCCCAGCAGGGACAGTGTTGCTGATAAGCATTTATTTTTTACACAGAAATGAAAAATATTTTCCTAATCCAGAAGAGTTTAATCCAGATAATTTTCTGCCTAAAAATTTAAGTAAAAGGCATCCATATGCTTATGTTCCATTCAGCGCAGGCCCAAGAAATTGTATTGGGCAGAAATTTGCTATGTTAGAACTGAAATCTGTTTTGTCAAAAATTATTTTTAACTTCATAATAGATCCATCTTCCGATGTTTGGGATGTAGAAGAAGATCCAAGTTTGGTCTTTCGGTGTGTAGGAGGACATAAAATTAAACTAAGACCAAGAAAATAA

>CYP4Y

ATGGCAAGGAAGAAAGGAAAAAGTTTTCTTAGCATTTTGGAAATGACTACTCCTAAATACACAGAATATGAATTCATCAAGTTATTACGGTCGATAACCGAACCTAAGAGGTGCTCGTTACAGGAATACTTTTCATGGGAATGCAAAAAAGCTTTAGAAGTCGTGTACCGCATGTGTAATGAATACTGTTTGCCAAGAGAAAGGATATTTAAATTTTGGTTTGGTCCTGTACTTGGAATAGTTGTTACAAATCCAAAATACATAGAGGCAATATTGACTAGTCCAGATGCTGCGGATAAACATTATGTCTATAAAGTCGTTGAATTAGTTGGAAATGGTTTATTCACTAGAAATGGTAAAGAATGGGAAGAACTTCGCAAACCTTTGGATAAATTATTAACCAAAAAGATGGTAGAATCAAATTTGGGCATGTTTCATAAGAAATCTATAAAATCGTGTAATGTGTTAAAAAAGTATGCTGATACTGGCGAATGTTTTAATATTAGACATTACATCACAAACTTTACATTAGATACTGTTTGTGTTTCAATTTTTGGATACGATTTGAATGAAATAGAAAATGATAATCATAATATCATGGAAACAATGGAAAGTATTGTGGAGGCTACTGTAAAATTTGTGTCCAATATTCTGACCTTGTTATATTTTCCATTTGGAGAGAAATTTTCTAAAGGTAGCCGTTGTATGAGGAACTTGGCTAAGTCATTTTGGAAATTATCTAGTGAGATTTTGCAGGGTAGACTACAAGCTAGGACAAAACTAGGAGAAAGTATAGATGATAAACCAATTTTCTATTCAGACGTCCTACTACAAAAAGCTAAAAAGTATAACCTATCTTGGGAAGAGGCTGGAAAACTTACTACTGATATGCTTGTAGCTGGAATTGATACATCTGCTGTGACAATTTCCTGTATTCTACTGTTATTAGCCATGAATCCGGAACATCAAGATGCTGTATACCGAGAACAATTAGAAATATTTGGAGATGATCTAGAAGTGTCACCTACTTGGCAACAACTATCCAAAATGGCTTATCTTACTAGAGTGATTAAGGAAGTGATGCGCCTTTTTGGCCCAATTGGAATATTTAGGAAAATATCAAATGATTTAGATTTAGGTGGAGACTACAAACTACCTCAAGGATGCGCAGCTTTCATTTTATTGTATTACCTACACAGAGATCCAACTCTATGGTCACATCCAAACGAATTCTATCCAGATCATTTTCTACCAGAAGAGTGTGCTAAAAGGCCGAAGAATACATATTTCCCATTCAGTTGGGGACCAAGATCTTGTCCAGGAAGTGTTTATGCTATGGCTTCGCACAAAATATTCATATCTACAGCGATTAGAAGATACAAATTTGAAACAGATTTAAAATTTGACAAGCTAGAATACAAGTACTCCTTATTATTGGAGGTCAGCCAAGGATATATGGTCAAGATAAAACCAAGAAATTGAAATAAGAAACATTTCATTACTCTCAATCATTAATAATGTTTACTTAAGCCACCACAGTCAATTTGATTATAGTTTTCAATGTAATTTAAGTTAAAATAAATTGAAATTTATT

>CYP4H

ATGATTCCAATTGCGGTAGTTTTTCTACTAGGTCTATTGTTTGTTGTAACATTGTTCGTACCTTTTGATCATGTGAACCTAAAATGGAGGACCATCAGAGCCATGTCAAAATTATCTGGACCATTTAGTATTCCATTTTTTGGTTTGGGACTTCTTATGGCGGTAATACGTGATAGCGAAATGGTATCAGTGCTCACTTATTACTACAAGAAATATCCAAGAATTTTAGCAACCTATATTGTTGGCATGCCACTCATCCTTTTACATCAACCTGACGATCTAGAGATATTGCTAGGAAGTGCGACTCACATCAAGAAAGGATTCGAATACGAAATTCTTAAATCATGGCTAAACGAAGGACTACTACTTAGCACGGGTGAAAAATGGCGACATCGGAGGAAACTGCTCACTCCTGCATTTCATTTTCGTATTTTAGAGGACAATTTACAAAGTTTAAATAAACATGCAAGATACCTGTTGCGTAATATTTTGGAACAAGACGGTAAACCGTTTGCAGCCGACCAATTGGTCACACTGTGCACACTTGACGTTATCTGTGAAACCGCCATGGGAATTTCTCTGAATACGCAGGATAATCAAGCCTTAGAATATGTAGATGCCGTGAAAAGATTACCTAGTCTAGTTGTGGACAGGATACGTAAATTTTGGAAAAGAAAGAACTGGTTATATTATTTAACGATATCCGGAAGGAAATTTCATAAATCTTTAACAACTTTACACAACTTTACAGAAAAGATAATTCAAGAAAGAAAACTCAGTTACAGTACTGAGATACCCAGAAACAACCAAAACACAGAAAGTGAGGAATTATTGCTGCCTAAAAAGCGAAAAGCATTTTTAGATAGTCTATTAGAACTTGATCTGAAATATCCAAATTTATTCAGCGATCTAGACATAAGGGAAGAAGTGGATACCTTCATGTTTGAGGGACACGATACTACCTCAGTGACCCTTACTTTTGCTCTATATCTTCTCGGACTTCATCCTGAGATTCAGGAGAAAACGTTTGAAGAGCAGTATAATATTTTTGGTGATAGCGACAGAGCTGCCACACGTCCAGATTTAAACGAAATGCACTATCTTGAGATGGTTATCAAGGAAACGTTAAGGCTATACCCCAGCGTACCGTATATCTCAAGATCAATCGCTGAAGACTTGATATTGCCTGGAAACGTAGTCCTTCCTGCCGGAGCGAATGTTCTTGTACTTCCGTACTTCCTTCATCGAGATGTTCGCTACTTCCCCGATCCAGAGGTTTTCAATCCAGAGAGATTTACAATAGAAAACTGCAAGACCAGGCATCCATATTCTTACATACCGTTCAGTGCTGGACCTAGAAATTGCATAGGACAGAAATTTGCTATGATGGAACTAAAGGTTGTTCTTTCGACAATTATTAGGTTTGTAAAAATAGAATCAGTCACCAAACCGGAAGATATAAAAGTATTTCCTTTTGTAATACTTAGA

>CYP4F

GTACTGGTAGGCTTATTGATTTTGGTGGCATTCATTCCCTTTGACTATGTGGACATCAGATGGAGGACGATTAGGATCATGTCAAAACTACCCGGCCCGCTGGCAATTCCAATTATTGGATTAGGTATCTTCATTGCACTTCTCCGTGACAATGAATTAGTGAAAACTATTTCCTCTTACTACTACCGTAAGCAATATCCTCGTTTGTTCGTAATGTATATAGTTGGCATCCCGATTATAGTTTTGAAAGATCCTAATGATCTAGAGCAATTACTTGGAAGTGTGAATCATATCAAAAAAGGACTTGAATATAAACCACTTCAAACATTTCTAAACGAAGGATTACTGACAAGTTCGGGTGAAAAATGGCGTAAAAGAAGGAAACTGCTCACAACAGCATTTCATTTCAATATTTTAAAGGACAATTTACCAACTATAAATAAACATGCCAGATACCTTTTGCGTAACATTTTGAAGAAAGATGGTAAACCATTTGAGGTCGATCATCTGGTCACACTGTGCACTCTCGACACTATTTGTGACATTAGAGTAATCTTAAAAATTAAAATGTTATCATTCATGTGTCCAGAGACCGCCATGGGGATAACGCTGAACTCGCAAGATAATGAAGCCTCGAAATATGTGGATGCAGTAAGAAGATTACCTGAGCTGGTGGTGGATAGAATTCGTAAATTTTGGAAGGGAAAAGATTGGTTATACCGTTTATTGCCTTCTGGTAAACAATTTTATAAATCTATAAGAACTTTGCACAACTTTTCTGAAAAGATAATTAAAGAAAGAAAACTTAGTTACAGTATCGAGATGTCTAGAAACTGCCCGAAAACAGAAAATGAAGAAGATTTGTTGGCACAAAATCCTAAAGCATTTTTAGACAGTCTGTTGGAACTTGATCTGAAATATCCAAATTTATTCACCGATGTGGATATAAGAGAAGAAGTGGATACCTTCATGTTCGAGGGGCACGATACTACCTCAGTCGCCCTCACTTTTGCACTATACATGCTTGGATGTCATCCGGGTATCCAGGAGAAAACATTTAAAGAACAATACGCAATTTTTGGTGAAAGTGATCGAGCTGTGACCAGTACAGATTTACATGAGATGCACTATCTAGAGATGGTCATCAAGGAAACATTAAGGTTATATCCCAGTGTTCCTTATATATCAAGATTACTCACCGAAGACTTAAAACTACATGGGAATTTAGTATTACCTGATGGAATAAATGTGATCGTACTTCCGTATTTCCTTCATCGAGACGCTCAATACTTCCCCGATCCTGAGGTTTTCAATCCAGAGAGATTTACAATCGAAAACTGCAAGGCTAGACATCCATTTTCGTATATACCGTTTAGTGCTGGACCTAGAAATTGTATAGGGCAGAAATTTGCCATGATGGAAATAAAAGTCATCCTCTCGACAATTATTAGATCTGTTAAAATTGAACCTATCACCAAACTGGAAGATATCAGCGTATTTCCGTTTTTAGTGCTTAGAAGTAATTCTGTGATGATTAAATGTGAACCAAGGATTACTGTCAAGTAATATTATCAATAGATAAATAAAACTGGTACTTACATTA

>CYP4EM3

ATGGATGATGTTAAAATAGTGAAAGACTTGATCAGGATAATTGAATACAGAAAATCTACAGAAGGATTTATTAGAGAAAGTGAAAAAAAGTTTGATTACCTAAAGAGCTTGAAACAAAAGTATCCTCGCCTAACAGCATTGTGGATAGTGGGAATTCCTGTTGTTATCATACATGAGCCAGAAGATTTAGAGGTAATAATGGGTAGCCCACATCATATAACCAAAGGACATGAATATGAAGCAATTCTTCCCTGGCTTCAAGAAGGATTATTAGTGAGCACTGGCGAAAAATGGCATAAACGTAGAAAACTTCTGACACCAACTTTCCATTTTCGTATACTAGAAGATAATTTACAGAGTATCAATAAGCATTCAAGAAATCTTGTCAGAAACATGTTAAATACAAAAGGAGAACCAATTGTAGCAGAAAATTTTGTAACACTGTGCGCATTGGACATTATTTGTGAAACTGCCATGGGATTTCCACTGAACAGTCAGGACAACGAAGGAAAGGAATATGTTCATGCTATAAAAAGGATCTCATCTTTAGTGGTTACAAAGATTTTGAAAATTTGGTTAAGAAGAAATTTCATTTTTTATTTAACTAAATCTGGAAAAGAATTCCATAAACGTTTGACCTTTTTACACAATTTCACAGAAAAGATTATCAGAGAAAGAAAAATCACTTACTTGGCTGAACAAGCGAAGCAAAAAAATAACCAAAATTACGACGATGAAGAAATTTACTTACCTAAAAAACGAAGAGCATTTTTGGACAGTATGATCGAATTGGACTTAAAAGATCCTAACTTATTCACTGATAAAGATATTAGAGAAGAAGTTGATACATTTATGTTCGAAGAAAAAGTTTTTAACGAAGTATTTGAACTATTTGGTGACAGTGAACGAAATGCCACACTGAAAGATCTCCAAGAAATGCACTATTTAGAAAAAGTTATCAAAGAGACTTTAAGAATGTATCCCAGTGTACCGTATATGTCTAGGGTATTGTCTCAAGACTTGAAACTTAGTGGGGATGTTATCGTGCCGACAGGAGTAAATGTTGTTATTGTCCCATATTTGGTGCATAGAAATCCTAGATTCTTTAAAAATCCAGAAGTATTTGATCCGGACCGGTTTTCCCAAGAAAATTGTAAAAACAGGCATCCATTCTCTTATATACCATTTAGCGCTGGCCCAAGGAATTGTATAGGTCAAAAATTCGCCATGATGGAACTGAAGGTAGTCATTTCAACAATTGTCAGATTTTCCAAAATAGAAACAGTCACCAAACCTGAAGAACTAGCTTTAGCACCCTTACTGATACTTAGATCGGTTACACCAATCAAAATAAAAGTTACCCCGAGAAATTATTAA

>CYP4EM6

ATGTTTTACGAACTATTCCTTATCGGAGCGGCTGTTTGCCTTTTTATTTATTTCCTACTGCCTGTGGAACATATGAATTTCGAGGAAAGAACTGTAAGCCTGATGAACCAGCTTCATGGACCAAAAGGCATTCCATTGCTTGGGATGGCAATCTATACAACCACATTATCAGAAGAAGCCTATTTACCATTCTTAAAGAGTATAATGGATCAATATCCAAGACTAACCGGACTATGGATTGTGGGTATGCCGTTTGTTCTGATGCAAGATCCAAACGATATTGAGGTTCTATTAAGTAGTCTAAATCACATTACCAAAGGAATTGAATATGAACCAATTACGCCATGGCTTCGACAAGGATTGCTCAACAGAGAAAAATGGCGTAGACGGAGAAAAACATTAACACCTGCTTTTCATTTTAATATCTTACAAGATAATCTACCATGTATCAACAAACATGCTAAACTACTCTTAAGAAATATGTTTAATGAAAAAGGAAAACCTTTGGTTGTTGAAGAATACATAACTTTATGCGCACTTGATGTGATCTGCGAAACTGCAATGGGATATTCAATGAATGCTCAAGACAATAATGCCAAAGAATATATCCAAGCTGTGAAAAGAATACAGATGATGGTAGTAAAACGGGTGATGAGTTTCTATTTAAGAAAAGATTGGTTCTTCAACTTGATGCCGTTCAGTAAAGATTTCTACAAAGATTTAAATACTGTGCACAGTTTTACAGCCAAGGGTCATGATACGACATCAAATGTGGCCACATTTACTTTGTACGAACTTGGTCGTAATCTATCTGTACAAAAGAAAGTTTTTGAAGAAATACAAAATATTTTTGGTGATAGCCAACGTGAACCAACCAGAAAAGATCTACATCAGATGCATTATTTGGACTGTGTGATTAAGGAAATCATGAGGATATATCCCAGTGTTCCCTACATTTCTAGAAAATTAATCCAGGATTTAGAACTCAATGATGGTACAATCCTTCCTAGAGGAGCTAACATTGCAATTATTCCATACTTTCTACACAGATCGCCCAAGTACTTTCCTAATCCAGAGATATTCGATCCGTCTAGATTTAGTTTGGAAAATTGCAAAAGGAGACATCCTTTTTGTTATCTACCATTCAGTGCAGGACCAAGAAATTGTATAGGGCAAAAGTTTGCATTGATGGAATTGAAGATTATAATCTCAACATTAATAAGGTATGCAACTGTAGAAACAATCACTAAACCAGAAGATTTCACAATATTACCGATGTTAATCATAAGACCATCTTCACCAATAAAAATCAAAGTCATGCCAAGAAAATGA

>CYP4T

GGTTTTGGTTTCATAACTGGTCTATTCAAAGAATACAGTTTATCACCAGGACTGGTTAAACTCTGGTTTGGACCACGGCTTGTGGTATTTGTTATGAATCCGAAGTACATTGAGACAGTGCTGACAAGTACAGAAACATTGTACAAAGATGGTTTTTACGGTTTTATTGGATTAGTCGGAAATGGGTTATTCGTTAGAAATGGTCAAAGATGGGAAGAATTACGAAAACCATTGAATAAATTGTTAACTAAAAAGATGATTGAATCAAATATTAATATGTTCCATGAGAAATCGCTGAAGCTTTGTAAAGTGCTGAAAAAATATTCTAATACCAAAGAAAGTTTAAATTTCAGACACTATTCAACAAATTTCGCATTAGATACACTTTGTGTTTCAAATTTTGGTCATGACTTAAACGAACTAGAAAAGGATAATCGCAATATTTTCGAACATATGGAAAGGATTGTAGATGAGATGACTAAAGTACTGATTAACATTATTCATTATTTCTACTTTCCATGGGTGAATTATTCGAGGAGTGGTAGAAATGTACAGAAACTGAGCGCATATCTTTGGAAATTATCTTGCGAGATTTTGAAATCTAGAATAGAAACTAGAAAAGAACTGGGAGAAGAAACAGACGGGCAACCAACCTTCTACTCAGATGTGGTCATACAAAAAGCGAAAGACTATAAACTATCTTGGGAAGAGACTGGAAAATTTGCTACCGATTTTCTTGTAGCTGGATTCGACACATCAGCTGTGACCACATCCTACATTATATTGATGTTGGCTATGTTTCCTGAACATCAAGAAGCTGTCTATCAAGAACAACTTGATATTTTAGGAGAAAATCCTGAAATGGCACCAACATGGGAACAATTATCCAAAATGGTTTATTTGACTAGAGTGGTAAAAGAAGTGATGCGTCTCTATTGTCCACCTTCAATATTGAGGCATGTTAGAAATGATTTAGATTTAGGACAAGACTACAAACTTCCTAAGGGATGCACAGTTCTTATCTCATTCTACTCTTTACATAGAAACCCAACTTTTTGGTCGCAACCAGAAAAGTTCTATCCAGATCATTTTCTGCCAGAAGAGTGCGCAAAAAGGCCGAAAAATGCATACTACCCATTCAGCTGGGGACCAAGGTCCTGCCCAGGTAGTGTTTATGCCATGATAGCAAATAAAACTCTTATATCAACTTTAATAAGGGAGTATAAATTTGAAACTGACTTAACGTTCGAGAATCTGGATTACAAGTATTCTTTGTTAGTTGAGGTCAGTCAAGGATTTATGGTGAGGGTCAAACCAAGAAAATGA

>CYP4V

CCACTGGTGATGGTATATGCGCCAGAAGCTGTGCAGCTAGTTCTAAGCACAAAGCAGAAACATTTAGATAAGGGTAAAATCTACGACACGTTGTTACCTATGCTAGGAGAAGGTTTGATCACCTGTAAAGCGGCCAAATGGTCAAGAAGACGTAAACTACTTACACCAGCTTTTCATTTCAACATTTTGGAGACTTTCTTTCAAGTGTTTCAAATTCGCTCTAATGATTTCATTGAAGAACTAAAGAAAGGTGGTATAACCAAACAGTACAAGGATATATGTCCTTTTGCTAAAAATCTTACATTGAACATAATCTGTGAAACGGCCTTGGGACTTCCGAATGAAGAAAGAAAAGAACAAATTGAGATCGTCAAAGCTATGCAAAGATTGGAGGAGATAGCGATGTATCGATGTATCTACCCATGGTTGCTATGTGATTGGGTTTTCAAATTAACTAGAGCCTACAGAACTCATGAAAGGAAAATACACATACTGAACAACTTCACTGACAAGGTAATTAAGAAACGGCAAGAAGTATTGCAAACTGAATTGGAGTTAGAAAACGACAATACAGATGGAGGTAAATATTGTTCAAAAATTATCTCAAAGAAAAATGCAATACTGATAGATATATTACTGAAATTAAATTCAGATGGTCAAAGTTTGAGAAATGAAGATATCCGTGAAGAAATTAAAACTTTTATGTTTGCCGGACATAATACTACGTATTTAGCAATCAGCTACTGCATATACCTACTTGGAAGGTATCCAGAAGTGCAGAAAAACGCCATCGAAGAAGTAGATACGATATTTGGCGATTCCAAGAGGGATCCAACTATGGACGATTTAAAACAATTGAAATATTTAGATCGATGTATTAGAGATACTTTACGATTGTATCCAAGTGTTCCTGTGATTGGTAGAAGAAGTTCAGAAGATCAGCCTATTGGCGAATACGTGATCCCAAAGAATTCAGATGTGATAATTATTCCTTACATAATACACCGTAATCCAAAACAGTTTCCAGATCCAGAAAGATTCAATCCTGACAACTTTCTTCCAGAAAACGTGAAAGGAAGACATCCATATAGTTACATACCGTTCAGTGCCGGCCCTAGAAACTGTATAGGCAAAAGATTTGCAGATCTTGTCATGCTTATTGCGATCAGCTGGGTACTGAGAGAGTTCACAATACATTCTCTACATAAACAGGAAGATTTGAAAGTATTGCCGAAAACAATACTAGAACCAGTGCATGGACTTCAAGTGAAATTGACACCACGTAGATAATTTGTGACGAAATTTATTATGTTTTAACACTAATATTAGATTGTTTTGAATTATTAATTTTAGTTTAATAGAATTTTTTGTTGAATGTGATGACAGTTACTTTAACTTAACACCTATTATTATAAAATAAATATTGCATTGTTATATATTTGTTTACACT

>CYP417A

ATGCTTGCATTGCTGCAAGAGGGCAACATTTTGAACAATATTTACATTAGTTCAGGAAAAAGATGGGAAGAATTGCGCAAACCGTTGAATAAAGTGCTTACTAAAAAGATGATAGAATCAAATCTGAACATGTTTCATGAAAAATCTATTAGATTGTGCAATGTGTTGAAAAAATATGCTGATGGTGGCGAAACTTTCAATTTGAGAAATTACGTAACAAACTTCACTGCAGATACACTGAGCCGTAAGTACTTTTCAAACTTTGGATACGATTTAAATGAGATCGAAAATAATAATCACAGATTATGCGACCGTGTTGAAGGAATTGTGGAAACTATAATAAAATTGGCATCTAACTTTCTACACATAATTTATCTTCCATTTTCGAAATTTTCGAAGAACGGCAGATGTTTGAAGGAAATCGGTAAACTATTTTGGAAATTATGTTGCACGGTAAGCAGTTTTAAACATAAGATTCTGCAAGCCAGAATAGACACCAGAAAAAAGCTTGGAGAGAATATGGATGATAAACCAACTTTCTACTGTGATGTTCTCATCAGTAAAGCCAAACAATACAAACTCTCTTGGGAAGATACTGGAAAACTTGCAACTGACTTCTTTATTGCTGGCTTTGATACATCAGCTGTGATAGGATCATACACTTTATTGATGCTGGCCATGTTTCCTGAACATCAAGAAGCTGTCTATCAAGAACAATTGGAGATTTTAGGAGAAAATCCTGATATAGTGCCACAATGGGAACAATTATCCAAAATGACTTACTTAACTAAAGTCATCAAGGAAGTAATGCGCCTTTATAGCCCGCCTGGAATATTTAGGAAAATTACGAAAGATATAGATTTAGGAGAAAACTATAAGCTTCCTGAAGGATCAACTGCCTTCATTATATTTTATTACCTACATCGAGATCCTGTTTTCTGGTCACATCCGGATAAATTCTATCCAGATCATTTTCTACCAGAAGAATGTGAAAAAAGACCAAAAGGATCATACTTTCCATTCAGCTGGGGACCAAGATCTTGTCCAGGAAGTGTTTATGCGATGACATCAATTAAAGTACTCGTTTCTACCGCAATTAGATATTATAAACTTTATACAAATTTAAAATTTCACGAATTGGAGTACAGATATTCCATAATGCTTGAAGTGAACGAGGGTTATATGGTCAAAATCAAACCAAGAAATTGA

>CYP4C

ATGAATGACTACATAAGCGGTATTCGCACTGGGGATCTCCCACTTACAAAGCCATGCTCTAGACCACTAGACCAACACCCGCCTCGCGCTGGCAGAGAATGGCATGAATTACGCAAACCTTTGGATAAATTGATCACTAAAAAAATGGTCGAATCAAATATTAATGTGTTCCATAAGAAAGCATTGAAAATGTGCCATATGATAAATAAGCATGCAGAGACAGGGATTGAGTTTGTTTTAAGAGGTTATATTGTGAATTACACTATTGAAGTTTTGTGTGTTACGAGTTTTGGATGCGATATAAATTTGAATGAAACTGATGGAATTAAATTAAAAGAAGCTTTAGAAAGCAAGGAAATCAAGTTGTGTTCGCTGTGTAATTTAGAAAACAGAGAAGAAAGTGTATTTCATTTTATAGCGGTATGCCCAATATTAAGAGAGTTTAGGTCCATATATTTTAAGAAGGATTGTTTAAATGAGCTGGAGTTTATGGACTATTTAAATGGTAAGTGTTGGAAAAGCTTGGTGAATTATTGTAAAGCGGCATGGGCATATAGATACGAACTGCTCCTCCAAGCCAGATTAAGGAATAGGACACTATTAGAAGGGTTCGATACATCAGCTGTAACATTGCATTATATATTGTTTTTATTGGCCATGTTTCCTGAACATCAAGAAGCTGTCTATCAAGAACAGATTGATATTTTTGGAGATAGTCCTGAAGTAGAACCTACTTGGGAACAACTCTCCAAAATGGAGTATTTGACCAGAGTATTGAAGGAAGTTATGAGAATTTATTGCCCAATTGGTATTTTCAGGAAACCAACAAATGATATAGACTTAGGTGATTATAAACTGCCTAAGGGATCTACATTGTTCATCATGTTTTACAGCCTACATAGAGATCCAAGACTTTGGTCACATCCAAATGAATTTCATCCAGATCATTTCCTACCAGAAGAGTGTGCTAGAAGACCAAAAGGAAGTTATCTTCCATTTAGCTGGGGTCCAAGATCTTGTCCAGGAAGTGTCTACGCAATGGCATCTTTAAAAATAGCCGTCTCTACAGCAATTAGAAGGTACAAGTTTGAAACCGGTGTGAAATTTGAAGAATTGGAATATAAATATGGGTTTTTATTGGAGCCAAAACAGGAGTATTTAGTCAGGATCACAAATAGAGCACCATTGCCATGA

>CYP4I

ATGGTTGAATCAAATTTAGAATTCTTCCATGAAACAGCTTTAAAAGTTTGTAAAGTAATAAGTAAACATGCTAAAAATGGAGAAACTTTCAATTTTAGAACATACGCAACCAATTATTCTGCAGATACTGTTTGTGTAACAAACTTTGGATACAATTTGAATGAAGTTGAAAAAGAACAATACAAGTTGTTAGATGTGTTTGAAAAGCTGCAAGCAATATTCGTAAAAATGGTTTTCCATGTACCAAATCACGTTAGCTTACTATATGCTGGTTTAACTTCAGATGGAAGACGTATAAAGAATATCGCCAGATCGTACTGGGATTTATCTTGTAAGATATTAAAGCAAAGAATTGATGATAGGAAAATGCTAGGCGAGGATATTGATGCAAAACCTGCTTTCTATTCTGACGTACTGGTACAAAGAGCGAAACAAGATAAATTGTCTTGGGAGGATACTGGAAGACTTGCTACGGACTTTCTCGTTGCTGGATTTGATACATCTGCTGTAACAACATCATACATTTTTCTAATGCTAGCCATGTATCCTGAACATCAAGCAGCTGTCTATCAAGAACAGATAGATATTTTAGGAAAGGATGCTGAGGTTTCCCCAACCTGGGAACAGTTATCGAAAATGATATATTTAACAAGAGTAATTAAAGAAGTTATACGATTATTTGGTCCAATCGCTATATTTAGGAAACTTGCCAATGATGTAGATCTAGGTGAATATAAACTTCCAAAAGGGTGCATGGCGTTGGTAGCATTTTCTTTATTACATAGAGACCCAGATATATGGACTCATCCTGATGAATTCTATCCTGATCATTTTCTGCCAGAGGAGGCAGCTGCTAGACCAAAAGGATCTTATTTCCCTTTCAGCTGGGGACCAAGATCTTGCCCAGGAAGTGTTTATGCGATGGCATCAATCAAAGTGATCGTATCCGCATTGATAAGAAATTATAAATTTGAAACGGATTTATTATTTGACCAGCTTGAATACAGGTATTCCTTACTACTTGAGGCCATACAGGGCTATATGGTCAGGATTAAACCAAGAAATTAA

>CYP4C75

ATGCCATTTAAAAATGTTCTTTTGAAGTTTCCCCGTGGATTCATATTTTTTAACTTTCATAATCATAATGTAATTAAAGAGAAGCGAGCAGAATTAAAAAAAGTCAAAGAAGGAAGACTGAATTATAATTCGAGTAATGACGAAGAAACTAAGGGAATTTTTGTACTAGATTATGGAGTAAAGAAACGATTAGCCTTTCTTGACCTGTTATTGAAAACTGCTGAAGAGGAAAAAGTACCGTTGAGCGATGAAGATATCCGTGAAGAAGTGGATACTTTCATGTTTGAGGGTCACGACACAACTTCGGCCGCACTTTGTTGGTTACTGTTTCTTCTGGGATCAAATCCTAAAATTCAGGAGAAAGTATTTAATGAGCAGCAAAGTATATTCGGTGATAGTGGCCGAAATCCTACAACTCAAGATTTGACCAAGATGAAGTACTTAGAATGTTGTATAAAAGAGGCTTTGCGATTGTATCCGAGTGTGCCCATGTTTGCTAGATTACTCACATCAGATGTCAAAATTGATAAATTTTTGATCCCTGCTGGTACGACGGCCATGATTGTAGCTTATCAGCTGCATCGTGATCCAGAAGTGTTTCCACAACCAGAAAGGTTCATCCCAGAAAGATTTCTTGACAGGGATTCAACGAGAAATCCGTTTGGTTATGTACCATTTAGCGCTGGTCCTAGAAATTGTATAGGCCAGAAATTCGCTTTGATGGAAGAAAAAGTTATTGTTTCTTGGATTCTGCGAAAATTTAAAATAACTTCAGATGATAAAAGAGAAGATTTGGTTTTATTAGCAGAATTAATTTTAAGACCTAAAAATGGCATTAGAATCAGTATTGAACCACGAAGATGA

>CYP3093A2

ATGATAAAGTACATTTTATGGACTCTTCGTGCAGCAAATATGATAGCCAACATACCTGGGCCTAAACCATTGCCAATCATTGGAAACGCTTTGATGTTTGCTAGGATTAACACTTTAGAAGAGCTATTTAATGTGGTCGACTCATTGGTTAAAGAATATGGAACTCAAGATGCAATTTTTAGATTATGGCTAGGTCCAAAACTGGTCATCTCTCTCGGGAATCCAAAACATATAGAGAAAATACTTTCAAATCCAGACGCTTTGCAGAAAGATGATATTTACGAACGCATAGGATTGGTTGGAAATGGTTTGTTTGTCAGAAACGGCAGAGAATGGCATGAATTACGCAAACCTTTAGATAAATTGATCACTAAAAAAATGGTAGAATCAAATATTAGTGTGTTTCAAGAGAAAGCATTGAAGATATGTAATATGATGAAGAAGCATGCTGAGACAGGCCTGGAATTCAATTTAAGAGGTTTTACTGTAAATTTCGCTATTGAGGTTTTGTGTGCTACAAGTTTTGGATGTGAGATAAATTTGAATGAAAATGATGGAATTAAATTAAAAGAAACTTTAGAAAAGTGTTTGGAATTGATTGTGAAATTTATTATTCAAACTCCTTACAATATAATCTTGACATGTGCGAAATTTTCTATGGATGGCAGACGTTTAAACCAATTTATAAAACCATTTTGGATCTTATGTAACGAGGTAGCAGTGCTAGGAAGGTTGCATCAGAAAAAAGTGGAACCAGTTTCCCTCCTTAGCAAAGATGACCAATCATTACCTCAGTACTACTCAGACGTGTTGTTAGAAAAAGCTACAAAAGACAAACTCTCTTTTGAAGACACTGGAAAGCTGGCAACTGACTTTTTCATTGCTGGATTCGATACATCAGCTGTAACATTGCATTATACATTGTTTTTATTGGCCGTGTTTCCTGAACATCAAGAAGCAGTCTATCGAGAACAAATGGATATTTTTGGAGATGGTCCTGAAGTAGAACCTACTTGGGAACAACTCTCCAAAATGGAGTATTTGACCAGAGTATTGAAGGAAGTTATGAGACTTTATTGCCCAGTTGGTATTTTCAGGAAACCGTCAAGTGATATAGACGTAGGAGATTATAAACTGCCCAAGGGTGCTACACTCTACATTACCTTATACAGCCTACATAGAAATCCAAAAATTTGGTCACATCCCAATGAATTCTATCCAGATCATTTCCTACCAGAAGAATGTGCTAGAAGACCAAAAGGATGTTATCTTCCATTTAGCTTGGGTCCAAGATCTTGTCCAGGAAGTGTCTACGCTATGGCATCCTTAAAAACAGCAGTGTCTACAGCAATTAGAAGGTACAAGTTTGAAACCGGTGTGAAATTCGAAGAATTAGAATATAAATATGGGTTTTTATTGGAGCCAAAACAGGAGTATTTAGTCAGGATCACAAATAGAGGTCCACTGCCATGA

>CYP3093A7

ATGGTAGAATCAAATATTAGTGTGTTTCAAGAGAAAGCATTGAAGATATGTAATATGATGAAGAAGCATGCTGAGACAGGCCTGGAATTCAATTTAAAAGGTTTTACCGTAAATTTCGCTATGGAGGTTTTGTGTGCTACAAGTTTTGGATGTGAGATAAATTTGAATGAAAATGATGGAATTAAATTAAAAGAAACTTTAGAAAAGTGTTTGGAATTGATTGTGAAATTTATTATTCAAACTCCTTACAATATATGCTTGAAATGTGCCAAATTTTCTATGGATGGCAGACGTTTAAACCAATTTATAAAACCATTTTGGATCTTATGTAACGAGCTTCTCCAAGGCAGATTAAGGAATAGGAAACTAATGCAAGAGGATGTCCACTTATTACCAAAATACTACTCAGACGTATTGTTGGAAAAAGCTACAAAGGACAAACTCTCTCTTGAAGACACGGGAAAGCTGGCAACTGACTTTTTCTTTGCAGGATTCGATACATCAGCTGTAACATTGCATTATACATTGTTTTTATTGGCCATGTTTCCTGAACATCAAGAAGCTGTCTATCGAGAACAGATTGATATTTTTGGAGATGGTCTTGAAGTAGAACCTACTTGGGAACAACTCTCCAAAATGGTTTATTTGACGAGAGTATTGAAAGAAGTTATGAGACTTTATTGCCCAGTTGGTATTTTCAGGAAACCAACAAGTGATATAGACTTAGGAGATTATAAACTCCCTAAGGGTGCTACCCTCTTCATAACCTTATACAGCCTACATAGAAATCCAAAAATTTGGTCACATCCCAATGAATTCCATCCAGATCATTTCCTACCAGAAGAAAGTGCTAAAAGACCAAAAGGAAGTTATATTCCATTTAGCTTGGGTCCAAGATCTTGTCCAGGAAGTGTCTACGCTATGGCATCCTTAAAAACAGCAGTCTCTACAGCAATTAGAAGGTACAAGTTTGAAACCGGTGTGAAATTTGAAGAATTGGAATATAAATATGGCTTTTTATTGGAGCCTAAACAGGAGTATTTAGTCAGGATCACAAATAGAGCACCATTGCCTTGA

>CYP3093A8

ATGCAAGAAGATGACCAATCATTACCTCAGTACTACTCAGACGTATTGTTAGAAAAAGCTACAAAAGACAAACTCTCTTTTGAAGACTCAGGAAAGCTAGCCACTGACTTTTTTATTGCAGGATCCGATACATCGGCTGTAACATTGCATTATACATTGTTTTTATTGGCCGTGTTTCCTGAACATCAAGAAGCAGTCTATCGAGAACAGATTGATATTTTTGGAGATAGTCCTGAAATAGAACCTACTTGGGAACAACTCTCCAAAATGGAGTATTTGACCAGGATATTGAAGGAAGTTATGAGACTTTATTGCCCAGTTGGTATTTTCAGGAAACCAACAAGTGATATAGACTTAGGTGATTATAAACTGCCTAAGGGTGCTACACTCTTCATTATATTGTACACTCTACATAGAAATCTAAAACTTTGGTCACATCCATATGAATTCTATCCAGATCATTTCCTACCAGAAGAGTGTGCTAGAAGACCAAAAGGAAGTTATATTCCATTTAGCTTGGGCCCAAGATCTTGTCCAGGAAGTGTCTACGCAATGGCATCCTTAAAAATAGCAGTCTCTACAGCAATTAGAAGGTACAAGTTTGAAACCGGTGTGAAATTCGAAGAAATAGAATATAAATATGGCTTTTTATTGGAGCCAAAACAGGAGTATTTAGTCAGGATCACAAATAGAGCACCATTGCCTTGA

>CYP3093A9
[truncated: 12,342 more chars]
